# Controlled masking and targeted release of redox-cycling *ortho*-quinones via a C–C bond-cleaving 1,6-elimination

**DOI:** 10.1038/s41557-022-00964-7

**Published:** 2022-06-27

**Authors:** Lavinia Dunsmore, Claudio D. Navo, Julie Becher, Enrique Gil de Montes, Ana Guerreiro, Emily Hoyt, Libby Brown, Viviane Zelenay, Sigitas Mikutis, Jonathan Cooper, Isaia Barbieri, Stefanie Lawrinowitz, Elise Siouve, Esther Martin, Pedro R. Ruivo, Tiago Rodrigues, Filipa P. da Cruz, Oliver Werz, George Vassiliou, Peter Ravn, Gonzalo Jiménez-Osés, Gonçalo J. L. Bernardes

**Affiliations:** 1 Yusuf Hamied Department of Chemistry, University of Cambridge, Cambridge, UK; 2 Center for Cooperative Research in Biosciences (CIC bioGUNE), Basque Research and Technology Alliance (BRTA), Derio-Bizkaia, Spain; 3 Instituto de Medicina Molecular João Lobo Antunes, Faculdade de Medicina da Universidade de Lisboa, Lisbon, Portugal; 4 Biologics Engineering, R&D, AstraZeneca, Cambridge, UK; 5 Wellcome-MRC Cambridge Stem Cell Institute, Department of Haematology, University of Cambridge, Cambridge, UK; 6 Division of Cellular and Molecular Pathology, Department of Pathology, University of Cambridge, Cambridge, UK; 7 Department of Pharmaceutical/Medicinal Chemistry, Institute of Pharmacy, Friedrich Schiller University Jena, Jena, Germany; 8 Department of Chemical Engineering and Biotechnology, University of Cambridge, Cambridge, UK; 9 Ikerbasque, Basque Foundation for Science, Bilbao, Spain; 10 Present Address: Department of Biotherapeutic Discovery, H. Lundbeck A/S, Valby, Denmark

**Keywords:** Drug delivery, Targeted therapies, Drug discovery and development

## Abstract

Natural products that contain *ortho*-quinones show great potential as anticancer agents but have been largely discarded from clinical development because their redox-cycling behaviour results in general systemic toxicity. Here we report conjugation of *ortho*-quinones to a carrier, which simultaneously masks their underlying redox activity. *C*-benzylation at a quinone carbonyl forms a redox-inactive benzyl ketol. Upon a specific enzymatic trigger, an acid-promoted, self-immolative C–C bond-cleaving 1,6-elimination mechanism releases the redox-active hydroquinone inside cells. By using a 5-lipoxygenase modulator, β-lapachone, we created cathepsin-B-cleavable quinone prodrugs. We applied the strategy for intracellular release of β-lapachone upon antibody-mediated delivery. Conjugation of protected β-lapachone to Gem-IgG1 antibodies, which contain the variable region of gemtuzumab, results in homogeneous, systemically non-toxic and conditionally stable CD33+-specific antibody–drug conjugates with in vivo efficacy against a xenograft murine model of acute myeloid leukaemia. This protection strategy could allow the use of previously overlooked natural products as anticancer agents, thus extending the range of drugs available for next-generation targeted therapeutics.

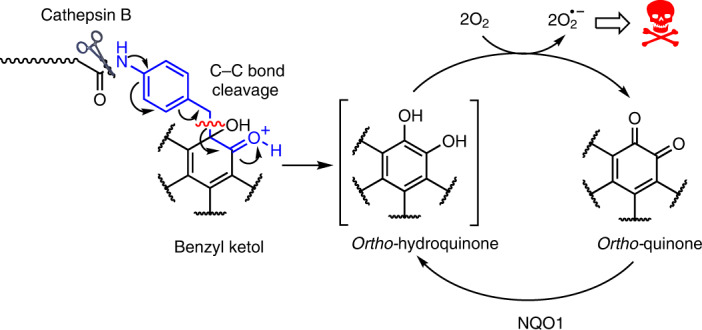

## Abstract (web summary)

A strategy for protecting redox-active *ortho*-quinones, which show promise as anticancer agents but suffer from redox-cycling behaviour and systemic toxicity, has been developed. The *ortho*-quinones are derivatized to redox-inactive *para*-aminobenzyl ketols. Upon amine deprotection, an acid-promoted, self-immolative C–C bond-cleaving 1,6-elimination releases the redox-active hydroquinone. The strategy also enables conjugation to a carrier for targeted delivery of *ortho*-quinone species.

## Main

The application of new chemical entities with innovative mechanisms of action is necessary for the development of next-generation therapeutics. Natural products are often a source of anticancer agents or act as an inspiration for rational molecular design^1,2^. However, many natural products identified with medicinal value in vitro remain underexploited due to dose-limiting toxicity in vivo^3^. Toxicity can stem from low cancer-cell specificity, off-target reactivity or, sometimes, built-in metabolic reactivity of functional groups. Such liabilities can be addressed with prodrug strategies that can mask pharmacophores to prevent secondary pharmacology or accelerated metabolism and can widen the therapeutic window^4^. Drug targeting strategies can also be useful. For example, antibody-mediated delivery for cancer-cell discrimination has facilitated the use of auristatins, maytansinoids and calicheamicins^5^. However, some medicinal compounds contain a reactive moiety that cannot be effectively protected by existing chemistry or prodrug strategies. Furthermore, only highly functionalized molecules that contain amine or hydroxyl groups tend to be suitable for conjugation to carriers. Toxic molecules that do not contain such amenable functional groups are often discarded if a carrier cannot be attached without laborious synthetic derivatization. Therefore, improvements in protection of functionalities and attachment of drug carriers to alternative functional groups will extend treatment options with already-discovered compounds^6,7^.

**Fig. 1:**
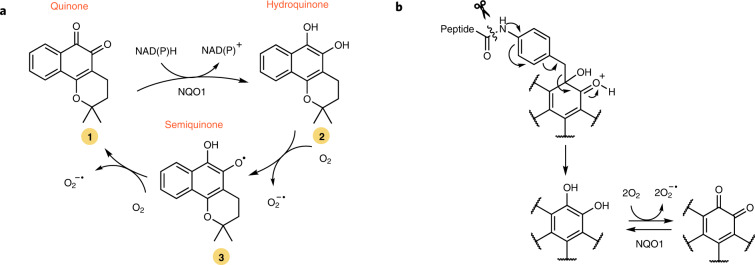
Protection strategy for *ortho*-quinone-containing compounds. **a**, Metabolism of *ortho*-quinones, for example, β-lapachone, **1**, which redox cycles between hydroquinone **2** and semiquinone **3** forms. **b**, This work: acid-dependent self-immolative release of *ortho*-quinones following enzymatic peptide cleavage. 1,6-Elimination of an aminobenzyl linker results in cleavage of a C–C bond to release an unstable hydroquinone intermediate, which auto-oxidizes into a quinone.

One underused family of cytotoxic natural products is the *ortho*-quinones. Several low-molecular-weight compounds that contain an *ortho*-quinone group, such as β-lapachone^8^, tanshinones (I, IIA, IIB and crypto)^9^, mansonones A–G^10^, dunnione^11^, miltirone^12^, salvicine^13^ and caryopteron A^14^, display wide antiproliferative effects in vitro. For example, β-lapachone (**1**, Fig. 1a), a natural product from Brazilian lapacho tree bark^15^ exhibits good efficacy against leukaemias^16^ and NQO1+ cancers, such as breast^17^, non-small-cell lung^18^ and pancreatic^19^. However, untargeted *ortho*-quinones have dose-limiting toxicity and metabolic liabilities because of their NQO1-dependent redox-cycling behaviour that results in the formation of reactive oxygen species (ROS; Fig. 1a)^20^. ROS disrupt the function of proteins^21^ and can lead to irremediable DNA oxidation, PARP1 hyperactivation and cell death^22,23^. Although this mechanism is of use against cancer cells, systemic propagation of ROS is undesirable and may lead to anaemia and methaemoglobinemia, as observed in clinical trials of **1** (refs. ^24,25^). Additional *ortho*-quinone toxicity can result from their ability to react as electrophiles with critical cellular proteins, peptides, nucleic acids or glutathione, which interferes with redox homeostasis^26^.

We hypothesized that if delivered to target cells without systemic side effects, *ortho*-quinones would have substantial potential as a monotherapy or in combination with mainstay anticancer agents^27,28^. Their metabolic interference may be a potential avenue to tackle malignancies^29,30^, and a strategy to overcome cancer drug resistance^31^. Some tumour selectivity of **1** has been reported, as overexpression of oxidoreductase enzymes (for example, NQO1) can increase redox-cycling rates selectively in the tumour^32^. However, no therapeutic *ortho*-quinone has yet reached the clinic due to generalized ROS-mediated systemic toxicity. In contrast, *para*-quinones, such as doxorubicin (Adriamycin), geldanamycin, mitoxantrone and mitomycin C, have lower redox-cycling rates and are successfully used in the clinic^22^. If the side effects of *ortho*-quinones could be prevented through selective protection and targeting strategies, this may lead to new cancer treatment options.

Prodrug approaches to mask redox activity and limit side effects of *ortho*-quinones, such as **1**, have been described. Boothman and co-workers developed a number of approaches, including formation of esterase-cleavable hydroquinone alkyl esters^33^, and pH-sensitive aryl imine, acyl hydrazone, ketal^34^, aminoalkyl alcohol and amino aromatic phenol prodrugs^35^. However, the disadvantages of these strategies have prevented their widespread adoption. For example, esters and hydrazones are often too labile under physiological conditions for effective targeting, whereas ketals are not sufficiently labile in tumours^34^. Modern prodrug strategies and linkers, including those that connect targeting antibodies and their payloads, use enzymatically activatable trigger groups and release drug functionality by means of self-immolative spacers. For example, *para*-aminobenzyl carbamate linkers release amines and *para*-aminobenzyl ether linkers release alcohols upon specific protease-triggered hydrolysis^36^. We hypothesize that a similar strategy is possible for *ortho*-quinones.

Here we present a modular strategy that uses self-immolative benzyl linkers for protection and controlled release of *ortho*-quinones. The self-immolative 1,6-elimination of a *para*-aminobenzyl linker attached at the quinone carbon as a benzyl ketol enables release of the hydroquinone. The released hydroquinone then oxidizes spontaneously to give the desired quinone by using the redox-cycling ability of the payload (Fig. 1b). The strategy is compatible with peptide linkers, such as those activated by the cysteine protease cathepsin B^37^. In this work, we explore the mechanism and properties of this C–C bond-cleaving elimination and prospectively apply it to protect and target **1** as an antibody–drug conjugate (ADC) for treatment of acute myeloid leukaemia (AML).

## Synthesis of self-immolative quinone models

**Fig. 2:**
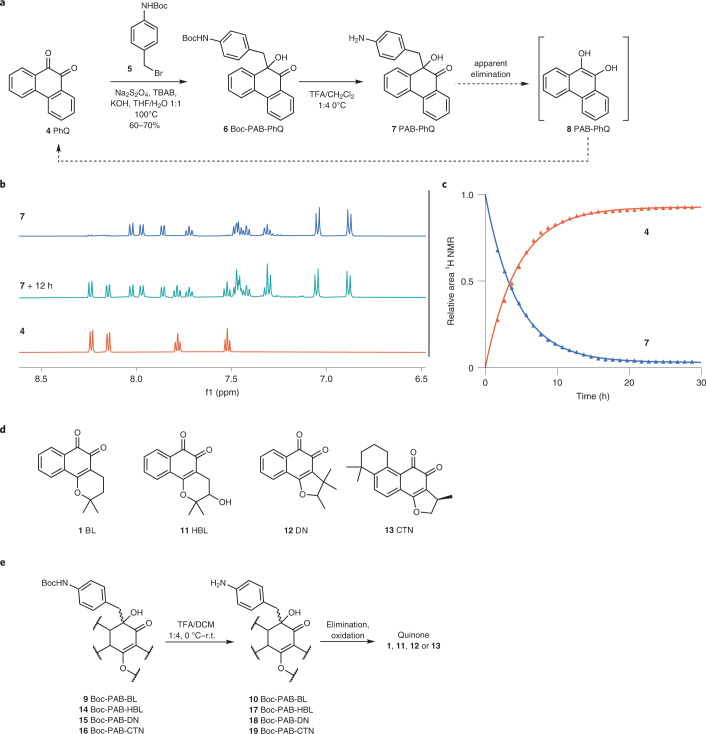
Synthesis of *para*-aminobenzyl phenanthrene-ketol and its elimination characteristics and protection of medicinally relevant *ortho*-quinones as *para*-aminobenzyl ketols. **a**, Sodium-dithionite-mediated reductive alkylation of PhQ, **4**, with Boc-*para*-aminobenzyl bromide **5** in a two-phase water/THF system generates benzyl ketol product **6** in 60–70% yield. Upon deprotection of *N*-Boc, compound **7** is unstable and eliminates to release quinone **4** and a linker by-product via the hydroquinone intermediate **8**. TBAB, tetrabutylammonium bromide. **b**, Kinetics of consumption of **7** and formation of **4** were measured by ^1^H NMR spectroscopy in moderately acidic solution (pH ≈ 6). Example ^1^H NMR aromatic region of **7** following 12 h at rt is shown, compared to freshly formed **7** and to **4**. **c**, Elimination reaction followed by relative area of ^1^H NMR peaks over 30 h at rt. **d**, Structures of β-lapachone (BL) **1**, 3-hydroxy-β-lapachone (HBL) **11**, dunnione (DN) **12** and cryptotanshinone (CTN) **13**. **e**, Protection of *ortho*-quinones **1**, **11**, **12** and **13** as benzyl ketol derivatives. Upon deprotection the derivatives eliminate to reform the quinones.

Initially, the synthesis of model derivatives of quinones that fragment upon removal of a protecting group was investigated using 9,10-phenanthrenequinone (PhQ), **4**, as a model compound (Fig. 2a). Phenolic alcohol-containing drugs are typically protected as *para*-aminobenzyl ethers^38^. However, when we investigated reductive alkylations of **4** with Boc-protected *para*-aminobenzyl bromide linker **5** (Fig. 2a), with a view to forming *O*-benzyl derivatives, we instead obtained the *C*-benzyl derivative Boc-*para*-aminobenzyl phenanthrene-ketol (Boc-PAB-PhQ), **6**, in good yield (60–70%). Indeed, *O*-benzyl derivatives of hydroquinones have been reported to be unstable and to rearrange to their corresponding *C*-benzyl isomers^39,40^. Intrigued by **6**, we tested its stability following *N*-Boc deprotection to give *para*-aminobenzyl phenanthrene-ketol (PAB-PhQ), **7**, which to our surprise appeared to be unstable, and as it was consumed, formation of quinone **4** was observed (Fig. 2b and Supplementary Figs. 1–6).

Complete consumption of **7** and formation of **4** was confirmed by ^1^H NMR spectroscopy. Appearance of **4** proceeded with a half-life (*t*_1/2_) of 2.94 ± 0.08 h at 25 °C in a moderately acidic methanolic solution with complete release within 20 h. Data fitting suggested that release followed first-order kinetics and the rate of consumption of **7** (*k*_obs_ = 6.18 ± 0.12 × 10^–5^ s^–1^) was consistent with the rate of formation of product **4** (*k*_obs_ = 6.55 ± 0.22 × 10^–5^ s^–1^; Fig. 2c and Supplementary Figs. 4–6). Through ^1^H NMR spectroscopy, we identified the presence of an aminobenzyl side product from the spontaneous reaction of methanolic solvent with an aza-quinone methide generated by self-immolative elimination (Supplementary Figs. 2 and 18). This, and the necessity for aniline deprotection for quinone release, suggests a self-immolative elimination mechanism. Under basic conditions, appearance of **4** was slower (*k*_obs_ = 1.16 ± 0.18 × 10^–6^ s^–1^) and not complete after 40 h (Supplementary Figs. 4–6), which suggested a pH dependence for the elimination reaction.

We anticipated that self-immolative release might generate an unstable hydroquinone intermediate (**8**) that could auto-oxidize to the corresponding quinone. 9,10-Phenanthrenediol hydroquinone **8** was not observed when elimination was performed under standard, oxygenated conditions as a result of its instability to auto-oxidation (Supplementary Fig. 2). However, in degassed solvent, intermediate **8** was observed (Supplementary Figs. 16 and 17), which confirms that the transient hydroquinone intermediate exists. Elimination from **7** was also followed by ultraviolet–visible spectroscopic analysis (Supplementary Figs. 20–22). Elimination from benzyl ketols, such as **7**, in this manner has not been previously documented and thus prompted further investigations.

## Generality of C–C bond elimination reaction to *ortho*-quinones

We next investigated whether the observations described above also applied to additional *ortho*-quinones. From β-lapachone **1** (Figs. 1a and 2d), *N*-Boc-protected *para*-aminobenzyl β-lapa-ketol (Boc-PAB-BL) **9** could be synthesized under identical conditions in similar yield. *N*-Boc deprotection of **9** gave (PAB-BL) **10**, which was observed to be unstable under acidic conditions and eliminated to reform **1** via hydroquinone intermediate **2** (Fig. 2e and Supplementary Figs. 8, 9 and 20).

**Fig. 3:**
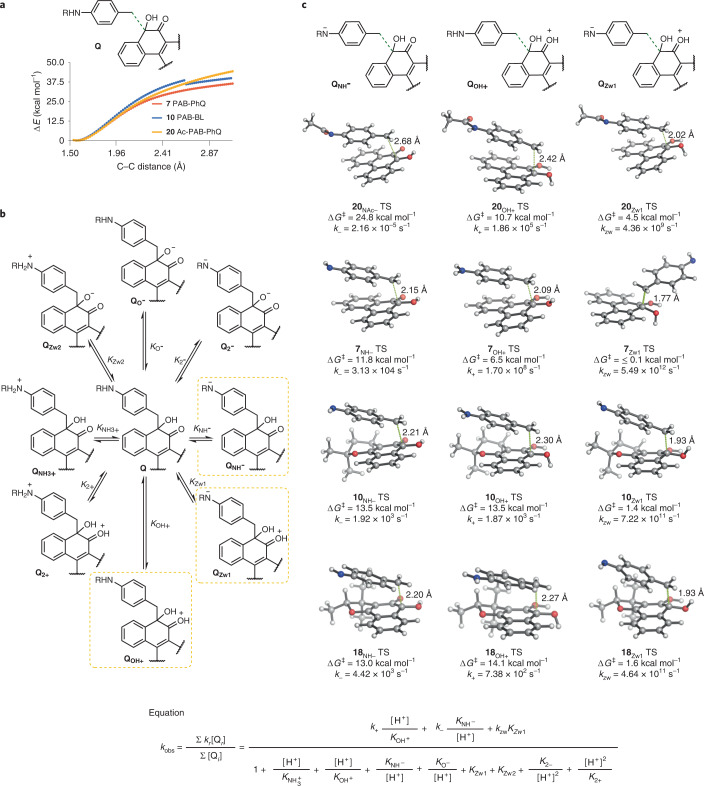
Modelling of the mechanism of elimination of *para*-aminobenzyl ketols. **a**, Theoretical potential energy surface calculated with PCM(H_2_O)/M06-2X/6-31+G(d,p) for the elimination of species **7**, **10** and **20** along the breaking C–C bond (green). **b**, General chemical scheme of nine different species denoted as Q (where subscript indicates neutral, cationic, anionic and zwitterionic) that are in equilibrium for the protected and unprotected *para*-aminobenzyl ketol derivatives in an aqueous solution. Species inside a dashed box (Q_NH-_, Q_OH+_ and Q_Zw1_) are those for which an elimination TS structure was found. The equation describes the effect of pH on the observed kinetic rate (*k*_obs_) and its derivation is described in the Supplementary Information. **c**, TS structures for the elimination of reactive species calculated at the same level of theory. Green dashed lines show interatomic distances (in angstroms) for the breaking C–C bond. Colour blue denotes nitrogen, orange denotes oxygen, grey denotes carbon and white denotes hydrogen. Intrinsic first-order kinetic constants (*k*_–_, *k*_+_ and *k*_zw_) were calculated from the theoretical activation energies (Δ*G*^‡^ in kcal mol^–1^) at 310 K using the Eyring equation. Source data

Three additional medicinal orthoquinones, (±)-3-hydroxy-β-lapachone (HBL) **11**, (±)-dunnione (DN) **12** and cryptotanshinone (CTN) **13** (Fig. 3a), were also successfully converted into their corresponding PAB-ketol analogues as diastereoisomeric mixtures (**14**–**16**; Fig. 2e). In the case of HBL, an intermediate hydroxyl protection and the use of an indium(0)-mediated Barbier reaction were required to achieve Boc-PAB-HBL **11**. In all cases, the removal of the Boc protecting group in acidic media led to reformation of their respective *ortho*-quinone precursors (**17**–**19** to **11**–**13**; Fig. 2e and Supplementary Figs. 10–15). These investigations validated the generality of the methodology for a number of medicinally relevant *ortho*-quinones with structural similarity to **4**.

## pH-dependent elimination profiles of benzyl ketol derivatives

The mechanism and kinetics of the elimination of model compound PAB-PhQ **7** were investigated. Initially, deprotected (R = H) derivative **7** and a theoretical protected (R = Ac) derivative **20** were compared (Fig. 3a). The energy profile along the breaking C–C bond (green) was calculated quantum mechanically at the PCM(H_2_O)/M06-2X/6-31+G(d,p) level of theory with the purpose of locating the transition state (TS) structures for the elimination. However, no maximum was detected, indicating that neutral species **7** remained unreactive and did not eliminate, contrasting with the observed release of **4**. Comparisons were made with different heteroatom-based leaving groups for the elimination of similar neutral *para*-amino benzyl species (Supplementary Fig. 106). This showed that reagents with chloride or activated alcohol (mesyl or triflate) leaving groups had low enough activation barriers (7.8–16.9 kcal mol^–1^) to eliminate. A carbamate leaving group was calculated to have a higher activation barrier (28.1 kcal mol^–1^). However, carbon-based leaving groups needed three electron-withdrawing groups (CN or NO_2_) or protonated carbonyl groups to have acceptable activation barriers (15.3–28.1 kcal mol^–1^) for elimination.

These results suggested that charged species might be necessary to promote the C–C bond elimination reaction. In aqueous solution, several charged species are in equilibrium albeit in various proportions. We considered nine possible charged states for **7** (Fig. 3b). Three of the nine species (**7**_**NH–**_, **7**_**OH+**_ and **7**_**Zw1**_) were reactive enough to allow an effective flow of electronic density from the *para*-amino group to the ketone to promote elimination of the hydroquinone, by either making the former more nucleophilic (NH^–^) or the latter more electrophilic (C = OH^+^). The activation barriers calculated for these species (Δ*G*^‡^_NH–_ = 11.8 kcal mol^–1^; Δ*G*^‡^_OH+_ = 6.5 kcal mol^–1^; ΔG^‡^_Zw1_ ≤ 0.1 kcal mol^–1^) suggest fast to extremely fast intrinsic elimination rates (Fig. 3c; *k*_–_ = 3.13 × 10^4^ s^–1^; *k*_+_ = 1.70 × 10^8^ s^–1^; *k*_zw_ = 5.49 × 10^12^ s^–1^). However, the global rate depends on the concentration of these reactive species in solution, which are exceedingly low due to the high p*K*_a_ values for aniline deprotonation (≥16) and low p*K*_a_ for carbonyl protonation (≤−2), values well beyond the practical range in aqueous solution. Hence, the theoretical global kinetic constant (*k*_theo_) can be modelled as a function of the intrinsic kinetic constants for each reactive species (*k*_r_), the equilibrium constants for all species in solution both reactive and non-reactive (*K*_i_), and pH; this expression for the theoretical rate constant can be related to the experimentally observed rate constant (*k*_obs_) (Fig. 3b (equation) and Supplementary Information).

It became apparent that despite many attempted approximations to cancel out some of the terms in the equation, *k*_theo_ calculated at different pH values was exceedingly dependent on the equilibrium constants involving the extremely low populated, but very reactive species (*K*_NH–_, *K*_OH+_ and *K*_Zw1_), which resulted in erratic pH dependency profiles depending on the numbers used. With no experimental values available for some equilibrium constants, and because tabulated^41,42^ and calculated values by using different programs such as Marvin 19.19.0 (2019), ChemAxon (https://chemaxon.org) or Epik (Schrodinger Suite)^43,44^ were not reliable (Supplementary Fig. 109), we decided to examine the elimination reaction kinetics for **7** at different pH values to derive more realistic equilibrium constants by using experimental data.

For *N*-protected derivative **20**, Ac-PAB-PhQ, we also considered nine different species. We found three of them to be reactive, but with higher elimination activation barriers (Δ*G*^‡^_NAc–_ = 24.8 kcal mol^−1^, Δ*G*^‡^_OH+_ = 10.7 kcal mol^−1^, Δ*G*^‡^_Zw1_ = 4.5 kcal mol^−1^; Fig. 3c, Supplementary Fig. 111 and Supplementary Table 5) relative to those calculated for the unprotected (R = H) derivative **7**. The activation barrier for the negatively charged acetanilide (**20**_NAc–_) was substantially increased with respect to the deacetylated analogue due to the large decrease in nucleophilicity of the nitrogen lone pair delocalized along the acetyl group. The dramatic obstruction of this reaction channel causes the global elimination rate to slow down by 2–5 orders of magnitude at pH 3–10 (Supplementary Fig. 110). Therefore, as for the widely used *para*-aminobenzylalcohol linkers^45^, elimination is self-immolative and faster with free aniline.

For comparison, we also modelled the elimination of the aminobenzyl ketol species derived from the medicinal natural product of most notable interest, β-lapachone, PAB-BL **10** and dunnione, PAB-DN **18** (Supplementary Fig. 111 and Supplementary Table 5). Similarly to PAB-PhQ **7**, the neutral species of PAB-BL **10** and PAB-DN **18** were unreactive to elimination. We considered the possible reactive species in a similar manner. An additional species—arising from protonation of the cyclic vinyl ether group—was considered but was calculated to be unreactive. In comparison to unprotected phenanthrenequinone derivative **7**, slightly higher activation barriers were obtained for the three charged reactive species of PAB-BL **10** (Δ*G*^‡^_NH–_ = 13.5 kcal mol^−1^, Δ*G*^‡^_OH+_ = 13.5 kcal mol^−1^, Δ*G*^‡^_Zw1_ = 1.4 kcal mol^−1^) and PAB-DN **18** (Δ*G*^‡^_NH–_ = 13.0 kcal mol^−1^, Δ*G*^‡^_OH+_ = 14.1 kcal mol^−1^, Δ*G*^‡^_Zw1_ = 1.6 kcal mol^−1^) and were noticeably higher for cationic **10**_**OH+**_ and **18**_**OH+**_. This increase is due to the reduced aromaticity around the carbonyl groups in **10** and **18**, which in turn affects their protonation ability (*K*_OH+_). Given the calculated differences in the intrinsic kinetic accessibility to the different reaction channels, and the expected thermodynamic differences in the relative populations of each reactive charged species, it was likely that both PAB-BL **10** and PAB-DN **18** had a slightly different pH-rate dependence relative to **7**.

**Fig. 4:**
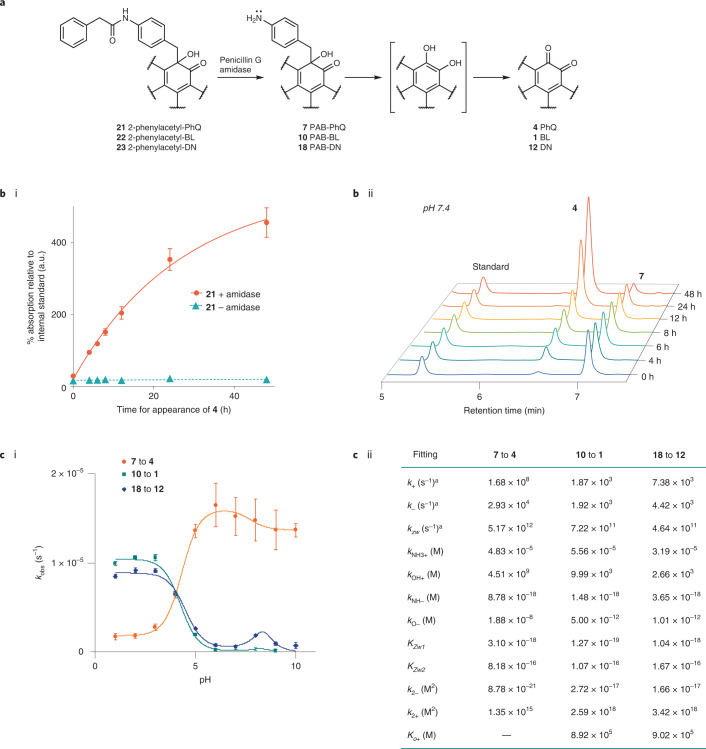
Experimental analysis of kinetics and pH-rate dependence of elimination. **a**, Amidase-releasable models to test elimination kinetics in buffered aqueous solution of protected and unprotected benzyl ketol species. **b**, i: Formation rate of **4** from **21** with and without penicillin G amidase incubation at pH 7.4 and 37 °C. Data are presented as mean values ± s.e.m., *n* = 3, from one representative experiment. ii: Example HPLC trace showing **7** and **4** after **21** was treated with penicillin G amidase at pH 7.4 and 37 °C. Time = 0 h time point taken 5 min after enzyme addition. **c**, i: Experimental elimination rate *k*_obs_ versus pH dependence of elimination of PAB-PhQ **7**, PAB-BL **10** and PAB-DN **18**, with data presented as calculated *k*_obs_ value from representative experiment ± 95% confidence interval. Lines represent fitting of the experimental data to theoretical models for *k*_obs_. ii: From this fitting, values for intrinsic kinetic (*k*_i_) and equilibrium (*K*_i_) constants were derived. ^a^Intrinsic rate constants (*k*_i_) calculated from the theoretical activation barriers were fixed during the fitting. Source data

To experimentally validate predictions and determine the pH-rate dependence of the elimination and the critical reaction parameters in aqueous solution, we synthesized models protected with a penicillin-G-amidase-cleavable phenylacetamide group^46^. Addition of penicillin G amidase to the protected models triggered amide hydrolysis and formation of the desired *para*-aminobenzyl ketol in neutral aqueous solution (Fig. 4a).

With this strategy, we showed that at physiological pH and temperature (pH 7.4, 37 °C), formation of PhQ **4** occurred at rate *k*_obs_ = 1.15 ± 0.66 × 10^–5^ s^–1^ following addition of the enzyme to **21**. Intermediate **7**, PAB-PhQ, could be identified (Fig. 4b and Supplementary Figs. 24–26) and elimination of **7** occurred at rate *k*_obs_ = 9.72 ± 2.88 × 10^–6^ s^–1^. Without added enzyme, **21** was stable and formation of **4** negligible. As mentioned above, hydroquinone **8** was not observed by NMR spectroscopic analysis of non-degassed solutions, suggesting that in oxygenated aqueous solution the intermediate has a very short *t*_1/2_ relative to that of species **7**.

As enzyme-mediated amide hydrolysis was fast (<5 min), immediate kinetic analysis of the subsequent elimination rate was possible without use of acidic or basic deprotection reagents, thus allowing control of pH. Focusing on the phenanthrenequinone, β-lapachone and dunnione derivatives, elimination from **7**, **10** and **18** generated enzymatically from **21**, **22** and the (*R*,*R*/*S*,*S*) pair of enantiomers of **23**, respectively, was followed across a range of pH values at 37 °C (Fig. 4c and Supplementary Figs. 27 and 38). Reactivity of the quinone products under basic conditions precluded analysis of product formation at pH >10. As predicted computationally, the reaction rate was dependent on pH. Compound **7** tends to show acid-promoted elimination, which peaks at pH 5–6 (*k*_obs_ pH 6 = 1.7 ± 0.3 × 10^–5^ s^–1^). The elimination rate of PAB-BL **10** was even more acid-dependent relative to **7** (Fig. 4c and Supplementary Figs. 33–36). Negligible elimination resulting in formation of **1** occurred at pH ≥6 within 72 h (*k*_obs_ pH 6 = 2.1 ± 0.8 × 10^–7^ s^–1^), and the rate peaked at pH 3 (*k*_obs_ pH 3 = 1.1 ± 0.1 × 10^–5^ s^–1^). For the PAB-DN derivative **18**, elimination displayed a very similar rate to that of PAB-BL **10** (*k*_obs_ pH 6 = 7.4 ± 1.1 × 10^–7^ s^–1^; *k*_obs_ pH 3 = 9.1 ± 0.2 × 10^–6^ s^–1^; Fig. 4c and Supplementary Figs. 37 and 38).

The *k*_obs_ derived at different pHs were fitted into the equation shown in Fig. 3b; the calculated *k*_i_ were used as fixed parameters and the individual *K*_i_ values were left as adjustable parameters. A good fit of *k*_obs_ to the theoretical kinetic model was obtained (**7**, *χ*^2^ = 0.270, *R*^2^ = 0.998; **10**, *χ*^2^ = 4.577, *R*^2^ = 0.998; **18**, *χ*^2^ = 8.726, *R*^2^ = 0.994), despite the different pH-dependent profile obtained for quinone derivatives **7**, **10** and **18**, which reflects the high quality of our kinetic model. The empirically derived equilibrium constants were all within expected ranges, except for the unusually facile deprotonation of the tertiary alcohol (*pK*_O–_ ≈ 8–12) (Fig. 4c(ii)).

With the calculated rate values, we became interested in the potential applications of the acid-dependent release. Such a profile is unusual because drug release from related *para*-hydroxybenzyl ether linkers is promoted in basic conditions^36^.

## *C*-benzylation prevents quinone redox activity

**Fig. 5:**
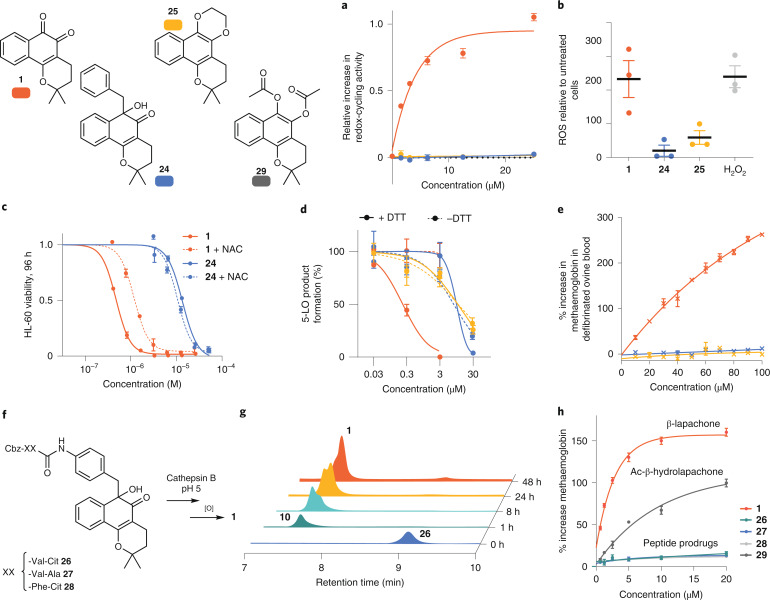
*C*-benzylation prevents redox-cycling of β-lapachone; cathepsin-B-cleavable β-lapachone prodrugs. **a**, In vitro redox-cycling capability of **1**, **24** and **25** measured by phenol red/horseradish peroxidase reporter assay. Relative increase in redox-cycling ability calculated by (*A* – *A*_0_)/*A*_0_, where *A* is absorbance at 610 nm for the test compound and *A*_0_ is absorbance at 610 nm for a sample with PBS added only. The redox activity of **1** saturates the assay above 10 μM, but **24** and **25** show no measurable redox activity up to 25 μM. **b**, Generalized ROS production by compounds **1**, **24** and **25** in HL-60 cells measured with the dye 2′,7′-dichlorodihydrofluorescein diacetate. **c**, Toxicity to AML cell line HL-60 with and without the ROS quencher NAC (600 mM). IC_50_ of **24** decreased slightly from 13.6 ± 1.3 µM to 10.5 ± 0.9 µM due to NAC. IC_50_ of **1** doubled from 0.47 ± 0.26 µM to 1.18 ± 0.18 µM with NAC. **d**, In vitro 5-LO enzyme target inhibition with and without DTT (1 mM). IC_50_ of **1** without DTT > 30 µM, with DTT 0.24 ± 0.13 µM. IC_50_ of **24** without DTT 8.0 ± 2.5 µM, with DTT 10.4 ± 4.1 µM. IC_50_ of **25** without DTT 11.3 ± 1.1 µM, with DTT 11.5 ± 2.2 µM. **e**, In vitro methaemoglobin generation measured by absorbance at 630 nm after 1 h incubation with test compounds. **f**, Cathepsin-B-activatable prodrugs release β-lapachone by linker elimination following enzymatic amide-bond cleavage. **g**, Release of **1** from **26** at 254 nm after in vitro dipeptide cleavage by protease cathepsin B (MES 20 mM buffer, pH 5). Peaks for PAB-BL **10** and β-lapachone **1** overlap. **h**, Concentration-dependent methaemoglobin generation by dipeptide prodrugs after 4 h incubation in ovine blood. Methaemoglobin was measured by absorbance at 630 nm following treatment with compounds relative to DMSO control. For **a**–**e** and **h**, data show mean ± s.e.m. from one representative experiment (*n* = 3).

**Extended Data Fig. 1:**
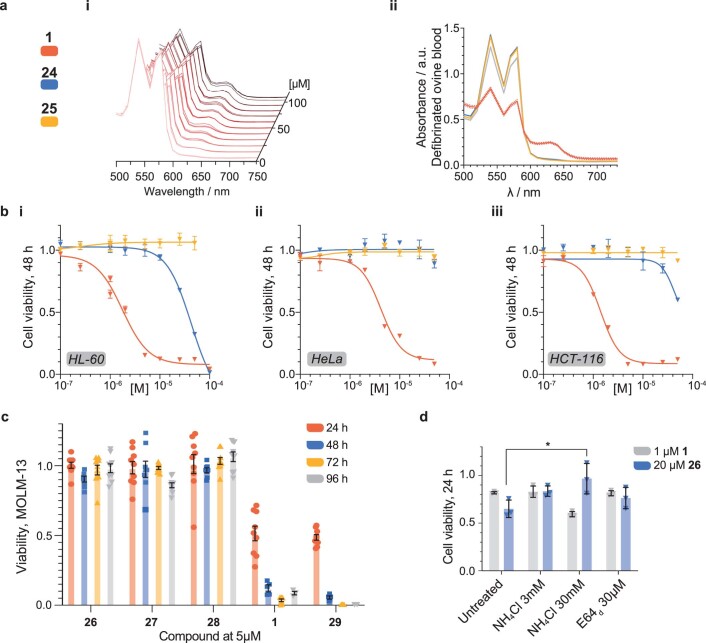
Additional *in vitro* assays of the benzyl ketol protection unit. **a**, (**i**) Concentration-dependent *in vitro* methaemoglobin generation by **1**, (**ii**) appearance of methaemoglobin peak at 630 nm for samples at 100 µM. Line represents mean values, with dotted borders representing ± SEM (n = 3). This experiment was repeated once with similar results. **b**, Toxicity of **1**, **24** and **25** to cancer cell lines: HL-60 (acute myeloid leukaemia), HeLa (endocervical adenocarcinoma) and HCT-116 (colon carcinoma). Data are presented as mean values fit to 4-PL model with error bars representing ± SEM (n = 3). These experiments were replicated once with similar results. **c**, Viability of leukaemia cell line MOLM-13 at 24 h timepoints following treatment with peptide prodrugs at 5 µM compared to compounds **1** and **29**. Values are mean normalised cell viability from three biological replicates ± SEM (n = 9). **d**, Lysosome deacidification and inhibition assay in HL-60 over 24 h. Significant difference marked: paired two-tailed t test for **26**, untreated vs. NH_4_Cl 30 mM treated viability: *p* = 0.0199. Bar height displays mean values from one representative experiment with error bar displaying ± SEM (n = 3).

The toxicity of the benzyl ketol pharmacophore generated upon quinone derivatization was investigated to determine the usefulness of the protection strategy for prodrug generation. *C*-benzylation disrupts the quinone scaffold, so it was expected that the redox-cycling ability of an *ortho*-quinone and associated undesirable systemic toxicity would be lost upon derivatization. We focused our attention on β-lapachone, the most well-studied medicinal *ortho*-quinone. Redox activity of a stable, non-releasable model of benzyl β-lapa-ketol, **24**, which lacks a *para*-amino-group necessary for self-immolative release, was compared to parent drug **1**, and control non-redox cycling protected 1,4-dioxine derivative **25**. Compound **24** was found to be non-redox active in an in vitro redox cycling assay (Fig. 5a and Supplementary Fig. 39) and, unlike **1**, did not generate detectable ROS in AML cell line HL-60, a cell line in which H_2_O_2_ generation by **1** has been previously reported (Fig. 5b)^47^. Compound **24** displayed decreased toxicity by >20-fold to a range of cancer cell lines, including leukaemia, breast, colon and cervical cancer (Extended Data Fig. 1b and Supplementary Fig. 42). The IC_50_ of **24** against HL-60 was unchanged by addition of antioxidant *N*-acetyl cysteine (NAC; Fig. 5c**)**. This is in stark contrast to **1**, for which the IC_50_ value more than doubled upon NAC addition as NAC alleviated ROS-mediated toxicity. These results together confirm that **24** does not redox cycle.

In previous work, we showed that **1** is, cumulatively, an reversible allosteric inhibitor of the 5-lipoxygenase enzyme (5-LO also known as 5-LOX or ALOX5), an enzyme that catalyses the oxidation of arachidonic acid, and that its anticancer activity was dependent on 5-LO expression^48^. Activity of compounds **24** and **25** against 5-LO was tested. β-Lapachone **1** inhibited 5-LO activity in the presence of the reducing agent dithiothreitol (DTT) that promotes redox cycling and formation of hydroquinone **2**. Unlike **1**, the inhibition profiles of **24** and **25** were not impacted by addition of DTT (Fig. 5d). Therefore, benzyl ketol β-lapachone derivatives are not strong inhibitors of 5-LO, unlike **1**.

Methaemoglobin formation, a major side effect of *ortho*-quinones in vivo, did not occur for the protected compounds **24** and **25** in an in vitro ovine blood model. In contrast, β-lapachone **1** caused dose-dependent methaemoglobin formation detectable at dose concentrations as low as 10 µM after 1 h of incubation (Fig. 5e, Extended Data Fig. 1a and Supplementary Fig. 40). Haemolysis of haemoglobin, related to anaemic side effects, was also lower for the protected models (Supplementary Fig. 41). These promising results indicated that the benzyl protection unit, while intact, has the capacity to mitigate the blood-borne redox-mediated side effects of quinones.

## Cathepsin B can trigger the release of β-lapachone from dipeptide prodrugs

Confident that the protection strategy produces redox-inactive derivatives, it was next investigated whether the self-immolative linker strategy was compatible with enzymatic cleavage of cathepsin-B-labile dipeptides. Dipeptide units^49^ Cbz-Val-Cit-, Cbz-Val-Ala- and Cbz-Phe-Cit- were added by sequential amide coupling from deprotected aniline intermediate **10** (**26–28**, Fig. 5f). After incubation of **26–28** with cathepsin B (Fig. 5g and Supplementary Figs. 53–55), compound **1** was observed. Aniline **10** was identified as a common intermediate and release of **1** from **10** occurred as previously observed. The Cbz group was not necessary for cathepsin B action (Supplementary Fig. 56). This demonstrated that the dipeptide-*para*-aminobenzyl ketols are compatible with cathepsin B, in an identical manner to dipeptide-*para*-aminobenzyl alcohol and carbamate linkers. Like benzyl model **24**, dipeptide prodrugs **26–28** generated less methaemoglobin in blood in an in vitro model relative to **1** (Fig. 5h). Additionally, they generated less methaemoglobin than an acyl-hydroquinone β-lapachone prodrug **29**, previously described by Ma et al.^33^ due to the increased stability of the dipeptide over labile ester bonds.

Cbz dipeptide prodrugs **26**–**28** were fully stable in human serum (Supplementary Figs. 58 and 59) and did not exhibit toxicity to AML cell line MOLM-13 at 5 µM concentration, unlike **1** and derivative **29** (Extended Data Fig. 1c and Supplementary Fig. 57) that achieved complete cell death at this concentration. Compound **26** exhibited 10-fold higher IC_50_ relative to **1** in AML cell lines HL-60 and MOLM-13 (Supplementary Fig. 57). After lysosomal deacidification (NH_4_Cl)^50^ or inhibition (E64d)^51^, **26** was less toxic, suggesting that acidic compartments help promote toxicity of the prodrug (Extended Data Fig. 1d). It was interesting that with NH_4_Cl at 30 mM, an increase in toxicity of **1** was seen, a result previously described for menadione^52^ (Supplementary Fig. 60). Prodrugs **26**–**28** may have suitability for treatment of NQO1+ solid tumours with concurrent overexpression of proteases, for example, cathepsins^53,54^ and acidic pH^55^ in the extracellular malignant environment.

## β-Lapachone is a promising treatment for AML

In our work, we were particularly interested in testing quinone **1** for the treatment of AML, a cancer for which **1** displays potent toxicity, as described in ref. ^48^ and confirmed by us on a panel of AML cell lines (Supplementary Fig. 43). AML is the most common form of acute leukaemia among adults^56^. It is characterized by immature myeloid cell proliferation and bone marrow failure and is a cancer for which new treatments are urgently needed. AML has a poor 5 yr survival rate of ∼20% and a large proportion of patients relapse^57^. Interference in redox homeostasis is an appealing treatment angle for AML^58^. Recent observations note that despite differences between the mechanisms of action of clinically used AML therapeutics, most share oxidative stress as a mediator of the cytotoxic effect^59^. For example, anthracyclines and arsenic trioxide induce rapid ROS accumulation^60,61^.

Quinone **1** also has relevant protein targets in AML. As mentioned above, in previous work^48^, we show that β-lapachone strongly inhibits enzyme 5-LO, an enzyme that is a candidate target for therapeutic targeting of the stem-cell-like blasts in AML^62,63^. In our experiments, 5-LO gene knock-out (KO), using CRISPR-Cas9 gene editing, impaired proliferation of leukaemia cell line HEL (Supplementary Figs. 44–47). Experiments also suggested that KO or shRNA-mediated knockdown (*K*_D_) may make AML cells more resistant to **1** (Supplementary Figs. 45–52). Both redox sensitivity and 5-LO target relevance made β-lapachone a valuable experimental drug for AML treatment. For AML, the data strongly supports the development of an intracellular targeting strategy to enable application of the quinone prodrugs.

## ADCs release β-lapachone in CD33 + AML cells in vivo

Antibody conjugation can enable targeting and assisted delivery of payloads inside AML target cells. We have shown that an *ortho*-quinone protection strategy can eliminate redox toxicity from **1**, and indeed, protection should therefore prevent any potential redox damage to the antibody carrier.

We reasoned that cell killing should depend on effective cellular trafficking of a conjugate to a low-pH cellular compartment inside target cells because pH 4–5 is required for the efficient release of **1** in vitro. Furthermore, in the system described, the slow release of **1** from the quinone protection unit PAB-BL **10** at physiological pH should limit systemic drug release, that is, any linker deconjugated from the antibody while in circulation should not release drug, even if a protecting dipeptide unit is broken. This can limit the toxicity profile of the *ortho*-quinone to that produced by non-specific cell uptake.

**Fig. 6:**
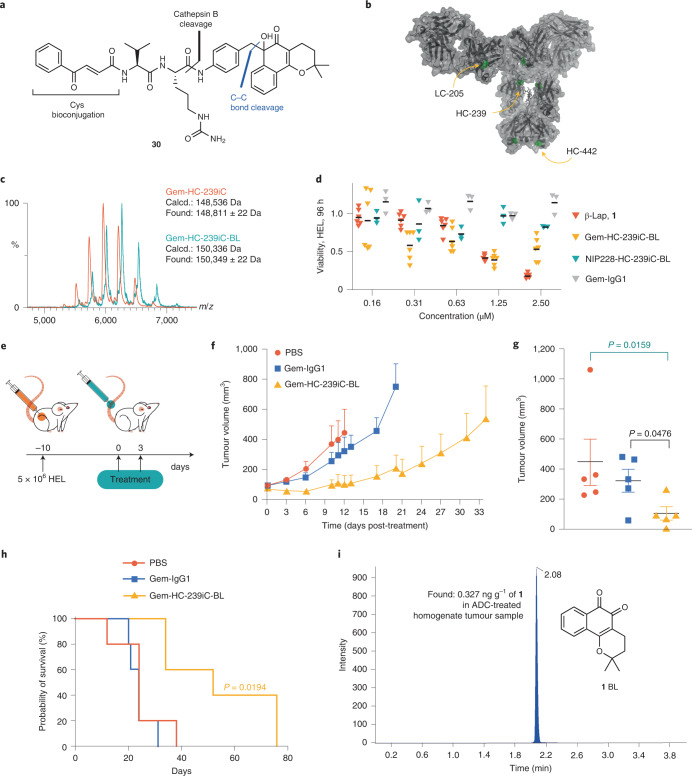
Gem-HC-239iC-BL has an effect on subcutaneous AML tumour growth and prolongs survival of mice. **a**, Structure of payload **30** for attachment to antibody through engineered cysteine residues on the antibody. **b**, IgG1 antibody containing a gemtuzumab variable domain with the three different cysteine mutation sites annotated. See Supplementary Information for details. In this study we decided to use Gem-HC-239iC. **c**, Native MS of refolded and conjugated ADC (green) in comparison to refolded unconjugated antibody (orange), indicates intact, homogenous conjugates. **d**, Toxicity to HEL after 96 h, measured by counting of number of live cells relative to control using the method of trypan blue exclusion. Line represents mean cell viability (one experiment, *n* = 6 or *n* = 3). Data suggest Gem-HC-239iC-BL shows increased toxicity compared with non-internalizing control ADC NIP228-HC-239iC-BL, also containing payload **30** in DAR 2, and to native Gem-IgG1, which showed no toxicity at 2.5 µM. **e**, Representation of the timeline of the HEL tumour cell inoculation and ADC therapy. **f**, Tumour growth curve. Data are represented as mean ± s.e.m. (*n* = 5). A two-way ANOVA indicates a significant effect of time versus treatment (*P* = 0.0089). **g**, Tumour volume at day 12 (*n* = 5). Data are represented as mean ± s.e.m. and a Mann–Whitney test was used to test statistical significance. P value has been deleted. **h**, Overall survival over time (*n* = 5). Log rank test indicates a statistically significant difference between DAR-2 ADC Gem-HC-239iC-BL treatment versus PBS and versus native antibody Gem-IgG1 treatment (*P* = 0.0194). **i**, Gem-HC-239iC-BL-treated (200 mg ml^−1^) xenograft tissue homogenate sample.

To test this strategy, we designed IgG1 antibodies for payload conjugation with a binding region based on the well-characterized CD33 targeting antibody gemtuzumab^64^ and synthesized payload linker **30**. Linker **30** is equipped with a Val-Cit cleavable moiety, as used in brentuximab vedotin^65^, and a 3-benzoylacrylic acid conjugation moiety, as previously developed by our group^66,67^ for site-specific cysteine conjugation (Fig. 6a). By using variants with engineered cysteine mutations, we generated homogeneous conjugates with a drug-antibody ratio (DAR) of 2. Three single-cysteine mutation sites were compared, one in the light chain and two in the heavy chain (Fig. 6b). Complete conversion was achieved for all conjugates. The positioning of the engineered cysteines on the antibody influenced the ease of conjugation. A Gem-HC-239iC mutant with a cysteine introduced in the hinge region of the heavy chain at position 239 offered favourable reactivity over Gem-LC-V205C and Gem-HC-S442C mutants with cysteine residues in more exposed positions on the light chain (Val to Cys mutant at position 205) and heavy chain (Ser to Cys mutant at position 442), respectively. With Gem-HC-239iC, complete conversion was achieved using 20 equivalents of linker per cysteine for 6 h to generate conjugate Gem-HC-239iC-BL. Gem-HC-V205C and Gem-HC-S442C mutants required more equivalents of linker (40 and 30 equivalents per cysteine, respectively) to achieve complete conversion. These conjugates may also be termed antibody–prodrug conjugates (APDCs)^68^ because they contain a protected payload.

The ADCs were characterized under reducing conditions using liquid chromatography–mass spectrometry (LC–MS) analysis (Supplementary Figs. 70–73), with the linker mass identified as an addition to the chain with the engineered cysteine in each case. Native-MS analysis confirmed that the ADCs had retained the bonding pattern of their light and heavy chains (Fig. 6c and Supplementary Figs. 74–84). All three conjugates retained a good CD33 binding profile with similar binding to their non-conjugated parent antibodies (Supplementary Fig. 64) and offered good conjugation integrity following incubation tests at physiological pH and temperature (phosphate-buffered saline (PBS), 37 °C) and in the presence of 10% human serum, over 48 h (Supplementary Figs. 85–90). The conjugates demonstrated stability to storage, retaining payload when stored at 4 °C for ∼1 month (Supplementary Figs. 91–96). Compound **1** has an IC_50_ against CD33 + HL-60 cells of ∼1 µM, and the conjugates displayed toxicity to HL-60 at 0.4 µM, a concentration at which unconjugated antibodies were non-toxic (Supplementary Figs. 97–99). Gem-HC-239iC-BL was selected as our favoured conjugate because of favourable reactivity with payload **30** and stability.

## DAR 2 ADC inhibits AML tumour growth in mice

We evaluated the antileukaemic effect of DAR 2 ADC Gem-HC-239iC-BL in a subcutaneous mouse model of human AML. NOD-SCID immunocompromised mice were inoculated with HEL cells to induce tumour growth and were treated intravenously with either ADC Gem-HC-239iC-BL, the native antibody Gem-IgG1 or PBS. In vitro, against HEL, Gem-HC-239iC-BL had similar toxicity to **1** while non-targeted NIP228-HC-239iC-BL and Gem-IgG1 showed no apparent toxicity (Fig. 6d). In vitro toxicity results were promising although variable. Results varied depending on the cell-counting method used and potentially because of conjugate aggregation arising from the hydrophobic^69^ linker and drug. In the in vivo model, a strong antitumour activity was observed with the administration of only two doses of ADC at 7.5 mg kg^−1^. The ADC-treated mice had decreased tumour volumes relative to the controls (Fig. 6e–g). Moreover, the antileukaemic activity of the ADC significantly improved the survival of the ADC-treated animals (mean of 52 days versus 24 days for control; Fig. 6h). Importantly, two out of the five ADC-treated animals were alive 76 d post-treatment, and no signs of toxicity or metastasis were seen in the liver, spleen, kidney, brain, bone marrow, heart and lung of control mice and mice treated with the ADC as demonstrated by histopathological analysis (Supplementary Figs. 100 and 101 and Supplementary Table 1). To verify drug release, we tested samples of fixed tumour tissue of mice treated with ADC Gem-HC-239iC-BL by LC tandem MS, and in ADC-treated mice we were able to detect and quantify **1**, which was present at 0.327 ng g^−1^ (Fig. 6i, Supplementary Figs. 102–105 and Supplementary Tables 2 and 3). These data provide evidence for the successful in vivo release of **1** through the acid-dependent C–C bond-cleaving self-immolative 1,6-elimination mechanism reported in this work. Together, these results demonstrate that the ADC antitumour activity in vivo has great potential to be further developed as an antileukaemic treatment.

Alternative payload classes are an important strategy to identify new treatments and combat drug resistance in the fight against cancer. Here we leverage prodrug chemistry and antibody-mediated delivery to develop a method for intracellular targeting and release of *ortho*-quinones from a protected redox-inactive form. This work begins to solve a fundamental problem of application of *ortho*-quinone therapeutics: the reactivity of *ortho*-quinones with cellular metabolism. Through protection and targeting we aim to increase the concentration of redox-active quinone in the target tissue and decrease the concentration in healthy tissues, thereby lowering associated dose-limiting toxicities.

Unlike previous approaches, our *ortho*-quinone protection strategy is truly modular and could be adapted to any peptide pro-moiety. The self-immolative 1,6-elimination mechanism described is unprecedented because it cleaves a C–C bond between the benzyl linker and quinone carbon. The elimination is triggered within an acidic pH range, depending on the protected quinone. We demonstrate the generality of the methodology, which we applied to protect model 9,10-phenanthrenquinone and four additional *ortho*-quinones. β-Lapachone, (±)-dunnione and cryptotanshinone are all *ortho*-quinones of medicinal interest. 3-Hydroxy-β-lapachone adds additional possibilities for functionalization compatible with the protection strategy.

As an example of the application of the *ortho*-quinone protection strategy, we synthesized cathepsin-B-cleavable prodrugs of β-lapachone, followed by ADCs using the protection chemistry. For β-lapachone, **1**, minimal release of the payload from the quinone-protection unit occurs at physiological pH even after peptide deprotection, which for an ADC means the chemistry offers a built-in protection against premature in-circulation drug release upon linker deconjugation. The rapid cell death triggered by the ADC relative to the untargeted prodrug suggests that released protected quinone species **10** reaches a low pH compartment (∼4) necessary for its fast elimination. In many cells the FcRn receptor will recycle the antibody to the cell surface directly from the endosome (pH ∼5.5–6) and prevent access to more acidic compartments^70^, but our results suggest that our ADC does not undergo notable endosomal recycling^71,72^. The activity of β-lapachone is believed to be independent of endosomal/lysosomal escape, (that is, it should give a toxic effect as soon as it is released from **10**).

This work also highlights the relevance of β-lapachone for the treatment of AML and verifies the relevance of 5-LO as a respective target. ADC therapy is well established in the AML disease area, with reapproval of CD33-targeting Mylotarg (gemtuzumab ozogamycin) for relapsed or refractory AML^73^ and with many more ADCs in development^74^. Our protected β-lapachone payload is efficacious in a xenograft AML model following ADC targeting and can effectively expand future treatment options upon further development and optimization. It should also be noted that β-lapachone is a lower-potency payload than those commonly used in ADCs; for example, most marketed ADCs have subnanomolar-potency payloads^5^. However, the concept that ADCs must contain a subnanomolar toxic payload has been recently challenged and efficacious cancer-cell killing has been obtained with lower-potency payloads^75^. Side effects, and hence the high attrition rate of ADCs in clinical studies, often result from payload-mediated toxicity; thus a lower-toxicity drug that is masked while in circulation with built-in deconjugation toxicity protection may be advantageous^76^. In this respect, β-lapachone or other *ortho*-quinone derivatives offer opportunities for the development of ADCs with a distinct mode of action.

The protection strategy described here is expected to be applicable to other *ortho*-quinones and may encourage the development of improved synthetic routes to, or new derivatives of, these high-value natural products. Derivatives of β-lapachone with higher toxicity that retain the quinone pharmacophore have been reported^77^. New prodrugs, drug-targeting strategies and combinations of the two will enable the application of compounds previously deemed inappropriate for medicinal use. Here, we have developed enabling chemistry that facilitates the integration of bioactive *ortho*-quinones into these strategies.

## Methods

### Synthetic procedures

All synthetic procedures are described in full in the Supplementary Information.

### Solvents, reagents and materials

All non-aqueous reactions were performed in oven-dried glassware under a positive pressure of nitrogen, unless otherwise stated. All reagents, unless otherwise stated, were purchased from commercial suppliers and used as received without further purification. Anhydrous solvents were used for non-aqueous reactions unless otherwise stated. Anhydrous dichloromethane and Et_2_O were freshly distilled over CaH_2_ under an atmosphere of argon. Anhydrous dimethylformamide (DMF) was obtained from commercial sources and used directly. Water used experimentally was deionized and prepared on site. Merck Silica gel 60 was used for flash column chromatography. Analysis of reactions was performed using thin-layer chromatography (TLC) silica gel 60 F254 plates. Analytical TLC plates were visualized by ultraviolet at 254 nm or by staining with known indicator solutions.

### Characterization of synthesized compounds

NMR spectra were recorded on Bruker 400-AVIII, DPX-400, 500-AVIII HD Smart Probe or Avance-600 BBI spectrometers as appropriate. NMR spectra were recorded at 25 °C and 400, 500 or 600 MHz for ^1^H and 100, 126 or 150 MHz for ^13^C. The solvent is specified for each spectrum. The residual solvent peaks were used as an internal reference for chemical shift (^1^H NMR CDCl_3_ *δ* 7.26 ppm, dimethylsulfoxide (DMSO) *δ* 2.50 ppm, MeOD *δ* 3.31 ppm; ^13^C NMR CDCl_3_ *δ* 77.0 ppm, MeOD *δ* 49.0 ppm, DMSO *δ* 39.5 ppm). Splitting patterns are presented as follows: chemical shift (ppm), multiplicity (s, singlet; d, doublet; t, triplet; q, quartet; qu, quintet; sextet; hept, heptet; m, multiplet (denotes complex pattern); br, broad; dd, doublet of doublets; dt, doublet of triplets; td, triplet of doublets), coupling constant (*J*) and relative integration value. Chemical shifts are given in ppm (*δ* scale) relative to the resonance of their respective residual solvent peaks, with coupling constants (*J*) in Hz. Structural assignments are made with the aid of correlated spectroscopy and heteronuclear single quantum coherence. H1/H2 denotes a signal which may correspond to H1 or to H2. For signal processing, MestReNova software v.8.0.0 or 14.2.0 was used. Absorbance and fluorescence spectra of small molecules were recorded on a MiniMax i3x imager in 96well clear plates or clear bottom black plates. High-resolution mass spectra of small molecules were obtained with a Thermo Fisher Orbitrap or Waters Xevo LC-MS and ionized by electrospray.

### General procedure to synthesize benzyl ketols 4, 9, 15 and 16

A mixture of orthoquinone (1 equiv.), sodium dithionite (5 equiv.) and tetrabutylammonium bromide (5 equiv.) in THF/water 1:1 was heated to 80 °C for over 10 min. Potassium hydroxide (20 equiv.) in water was then added, followed immediately by Boc-*para*-aminobenzyl bromide **5** (5.5 equiv.) in THF. The reaction was refluxed for 4.5 h. Water was added, then the product was extracted into ethyl acetate, washed with water, dried (magnesium sulfate) and solvent was removed in vacuo. The crude product was purified by flash column chromatography.

### General procedure to synthesize benzyl ketol 14

Boc-*para*-aminobenzyl bromide **5** (2.9 equiv.), NaI (8.5 equiv), *O*-protected hydroxy-β-lapachone (1 equiv.) and indium(0) powder (2.3 equiv.) were added to anhydrous DMF. The solution was heated to 40 °C and sonicated for 3 h, while monitoring by TLC. Water and 1 M HCl (drops) were added to quench the reaction and the product was extracted with ethyl acetate. The organic layers were combined and washed with brine, dried (sodium sulfate) and solvent was removed in vacuo. The product was purified by flash column chromatography on silica gel. Pure *O*-protected benzyl ketol (1 equiv.) was dissolved in THF and methanol and stirred at 0 °C. A separate solution of lithium hydroxide monohydrate (2 equiv.) in water was prepared and added dropwise to the solution over 5 min. The mixture was stirred at 0 °C until completion and then acetic acid (2 equiv.) was added to quench the reaction. The solvent was removed in vacuo, and the residue was redissolved in ethyl acetate. The organic layer was washed with water, brine, dried (sodium sulfate) and the solvent removed in vacuo, affording benzyl ketol **14**.

### ^1^H NMR kinetic analysis of elimination of PAB-PhQ

Kinetics of the release of PhQ **4** from PAB-PhQ **7** were monitored by ^1^H NMR at 25 °C. Analysis was performed in MeOD due to poor solubility of the compound in H_2_O. To form the active eliminating species, Boc-PAB-PhQ **6** was dissolved in dichloromethane/trifluoroacetic acid 4:1 at 0 °C and stirred for 30 min, upon which the reaction appeared complete by TLC. Product **7** was dried in vacuo before being dissolved in MeOD. The basicity of the solution was altered by addition of triethylamine (drops). pH was estimated using pH paper. pH values are only indicative because analysis is performed in MeOD. Elimination rates were determined from the aromatic region. Kinetics were measured by monitoring the integral of one peak, normalized to the number of protons present (that is, the integral of 1H), divided by the total integral of the aromatic region (*δ* = 6.2–8.6 ppm, that is, the integral of 12 H). Analysis was performed using Mestrenova v.14.2.0 processing software and rate constants were calculated using GraphPad Prism v.8.0 software.

### Penicillin-G-amidase-mediated kinetic analysis of elimination at physiological pH

Stocks of 2-phenylacetyl-*para*-aminobenzyl ketol were made up in DMF at a concentration of 10 mM. For a reaction, 17.5 µl of ketol stock was added to 17.5 µl of a 10 mM stock of internal standard in DMF and 310 µl of PBS at pH 7.4. To initiate a reaction, 5 µl of a suspension of penicillin G amidase (PenG, Sigma-Aldrich enzyme 76427, 5–10 U mg^−1^) was added. In control samples 5 µl of PBS was added in place of the enzyme. Test and control samples were then incubated at 37 °C with shaking. At the recorded time points, 20 µl aliquots of the reactions were analysed by HPLC (254 nm, ThermoFisher U3000). HPLC column: Phenomenex Kinetex C18, 5 µm, 50 × 4.6 mm, 100 Å; flow, 1 ml min^−1^. Gradient and solvent system for assay with 2-phenylacetyl-PAB-PhQ **21**: A = H_2_O, B = acetonitrile, *t* = 0–1 min 0% B, *t* = 1–10 min 0–100% B, *t* = 10–11 min 0% B. Gradient and solvent system for assay with 2-phenylacetyl-PAB-BL **22** and 2-phenylacetyl-PAB-DN **23**: A = H_2_O + 0.1% formic acid, B = acetonitrile + 0.1% formic acid, *t* = 0–1 min 0% B, *t* = 1–10 min 0–40% B, *t* = 10–16 min 40–50% B, *t* = 16.1 min 100% B, *t* = 16.1–18 min 100% B, *t* = 18–20 min 0% B. Peaks were identified by LC–MS analysis and comparison with pure compounds. Values described represent peak area (mAU × min) divided by peak area of internal standard. Recorded values are averages of three independent reactions and error bars display standard errors of the mean. Fitting was performed using GraphPad Prism 8 software. Rate and half-life measurements were calculated by consumption of intermediate *para*-aminobenzyl ketol and formation of product quinone.

### pH-rate aqueous kinetic analysis of fragmentation

pH-rate analysis was performed by adaption of the PenG assay. To obtain deprotected *para*-aminobenzyl ketol species, 2-phenylacetyl protected ketol was dissolved in PBS with 33% DMF to a concentration of 3.3 mM. Then 20 µl of a stock solution of PenG was added to 600 µl of this stock and incubated for 20 min at 37 °C, after which 20 µl of a 100 mM stock of internal standard in DMSO was added. Reactions were then set up immediately. For a reaction, 10 µl of the aminobenzyl ketol/standard stock was added to 190 µl of citrate–phosphate buffer at the specified pH to obtain an aqueous solution containing 1.8% DMSO. Test samples were incubated at 37 °C with shaking. At the recorded time points, 20 µl aliquots of the reactions were analysed by HPLC (254 nm, ThermoFisher U3000). HPLC conditions: column Phenomenex Kinetex C18 5 µm, 50 × 4.6 mm, 100 Å; solvent system A = H_2_O + 0.1% formic acid, B = acetonitrile + 0.1% formic acid; flow, 1 ml min^−1^. Gradient for assay with PAB-PhQ **7**: *t* = 0–1 min 0% B, *t* = 1–10 min 0–100% B, *t* = 10–11 min 0% B. Gradient for assay with PAB-BL **10** and PAB-DN **18**: *t* = 0–1 min 0% B, *t* = 1–10 min 0–40% B, *t* = 10–16 min 40–50% B, *t* = 16.1 min 100% B, *t* = 16.1–18 min 100% B, *t* = 18–20 min 0% B. Values described represent peak area (mAU × min) divided by peak area of internal standard. Where necessary, peaks were deconvoluted using Origin fitting software. Recorded values are averages of three independent reactions and error bars display standard errors of the mean. Values were fitted using GraphPad Prism software. Rate and half-life measurements were calculated based on consumption of the *para*-aminobenzyl ketol species.

### In vitro redox-cycling ability analysis

The in vitro redox-cycling capability of compounds was assessed by phenol red/horseradish peroxidase (HRP) assay by adaption of a previously described protocol^78^. Briefly, stocks were prepared as follows: (1) DTT to a concentration of 2.4 µM in PBS pH 7.4 buffer; (2) compound stocks in PBS pH 7.4 buffer to 3× the desired concentration by 1,000× dilution of DMSO stocks; (3) a stock of phenol red/HRP detection reagent containing 300 µg ml^−1^ phenol red and 180 µg ml^−1^ HRP enzyme in PBS pH 7.4 buffer; (4) a stock of 1 M NaOH in water; (5) a stock of 100 µM H_2_O_2_ in PBS prepared by addition of a 30% solution of H_2_O_2_ in water into 1,000 µl of PBS. Into each well of a 96-well clear flat-bottomed plate was then added 40 µl of compound stock in PBS followed by 40 µl of DTT stock, followed immediately by 40 µl of phenol red/HRP reagent. A positive control with 100 µM H_2_O_2_ added in place of compound was included. The plate was incubated in the dark at room temperature for 15 min. Following this, 20 µl of 1 M NaOH was added to each well to terminate the reaction. Absorbance was immediately measured at 610 nm (MiniMax i3x Imager, Molecular Devices). Absorbance of treated wells was normalized to control wells treated with PBS only (zero redox cycling).

### Cellular ROS detection

To determine ROS generation by compounds in leukaemia cells, HL-60 cells were washed with PBS, then incubated in serum-free media (10 ml of 1.6 × 10^6^ ml^−1^ cell concentration) with 2,7-dichlorofluorescin diacetate at 20 µM for 30 min at 37 °C. Following this, the cells were washed with PBS and resuspended in Fluorbrite–DMEM media (ThermoFisher) (10 ml). Then, 450 µl of the cell mixture was seeded into wells of a clear flat-bottomed 12-well plate, to which 50 µl of a 10× stock of the compounds in media had been already added, to achieve a final compound concentration of 50 µM. The 10× media stocks were made by 100-fold dilution of DMSO compound stocks, to ensure a final in-plate DMSO concentration of 0.1%. DMSO 0.1% only was used as a negative control. H_2_O_2_ was used as a positive control, for which 10 µl of a 30% aqueous solution was dissolved into 1 ml DMSO, then diluted 100-fold into media for plating, to give a final H_2_O_2_ concentration of 1 µM. After plating the cells were immediately incubated at 37 °C in the dark for 15 min before the fluorescence of the wells (excitation, 485 nm; emission, 535 nm) was recorded (MiniMax i3x Imager, Molecular Devices).

### Methaemoglobin analysis

Defibrinated Oxoid sheep’s blood (ThermoScientific) was diluted to a 5% v/v suspension in PBS pH 7.4. In a 96-well microtitre plate, 150 µl of blood suspension was added to 50 µl of serially diluted compound in PBS containing a 4× stock of each compound of interest. Serial dilution of compounds from DMSO stocks into PBS was performed to achieve an in-plate DMSO concentration of 0.1%. PBS buffer alone was used as a negative control. The plate was incubated for the specified time at 37 °C. The plate was then centrifuged at 3,500 r.p.m. for 5 min. The supernatant was removed and cells lysed by resuspension in 100 µl of a solution of Triton-X-100 (1%) in PBS. Following this, 80 µl of lysed cell contents were transferred to a second microtitre plate for ultraviolet measurement. Ultraviolet absorbance was measured immediately at 630 nm (MiniMax i3x Imager, Molecular Devices). Methaemoglobin increase was determined by $\left( {{{A}}_{630} - {{A}}_{630}^0} \right)/\left( {{{A}}_{630}^0} \right) \times 100$, where *A*_630_ is the absorbance of the test well at 630 nm, and ${{A}}_{630}^0$ is the absorbance of the negative control at 630 nm.

### Haemolysis analysis

To analyse haemolysis, defibrinated Oxoid sheep’s blood (ThermoScientific) was diluted to a 5% v/v suspension in PBS pH 7.4. In a 96-well microtitre plate, 190 µl of blood suspension was added to 10 µl of serially diluted compound in PBS containing a 20× stock of each compound of interest. Serial dilution of compounds from DMSO stocks into PBS were performed to achieve an in-plate DMSO concentration of 0.2%. PBS buffer with 0.2% DMSO was used as a negative control. Triton-X (1% v/v) was used as a positive haemolysis control. The plate was incubated for 6 h at 37 °C. Following incubation, the plate was centrifuged at 3,500 r.p.m. for 5 min at room temperature. Cell pellets were lysed by resuspension in 100 µl of a solution of Triton X-100 (1%) in PBS. Then, 80 µl of lysed cell contents were transferred to a second microtitre plate for ultraviolet measurement. Ultraviolet absorbance was measured immediately at 540 nm. The percentage of haemolysis was determined by $\left( {{{{\mathrm{A}}}}_{540}-{{{\mathrm{A}}}}_{540}^0} \right)/\left( {{{{\mathrm{A}}}}_{540}^{{{{\mathrm{total}}}}}-{{{\mathrm{A}}}}_{540}^0} \right) \times 100$, where *A*_540_ is the absorbance of the test well at 540 nm, ${{{\mathrm{A}}}}_{540}^0$ is the absorbance of the negative control at 540 nm and ${{{\mathrm{A}}}}_{540}^{{{{\mathrm{total}}}}}$ is the absorbance of the positive haemolysis control (100% haemolysis at 540 nm).

### Expression, purification and cell-free activity assay for recombinant human 5-LO

*Escherichia coli* (BL21) was transformed with pT3-5-LO plasmid, and recombinant 5-LO protein was expressed at 30 °C as described^79^. Cells were lysed in 50 mM triethanolamine/HCl pH 8.0, 5 mM EDTA, 1 mM phenylmethanesulfonyl fluoride, soybean trypsin inhibitor (60 μg ml^−1^) and lysozyme (1 mg ml^−1^), homogenized by sonication (3 × 15 s) and centrifuged at 40,000*g* for 20 min at 4 °C. The 40,000*g* supernatant (S40) was applied to an ATP–agarose column to partially purify 5-LO as described. Aliquots of semipurified 5-LO (0.5 μg) were diluted with 1 ml ice-cold PBS containing 1 mM EDTA. Samples were preincubated with the test compound or vehicle (0.1% DMSO) with or without 1 mM DTT, as indicated. After 10 min at 4 °C, samples were prewarmed for 30 s at 37 °C, and 2 mM CaCl_2_ plus 20 μM arachidonic acid was added to start the formation of 5-LO products. After 10 min, the reaction was stopped by addition of 1 volume of ice-cold methanol, and the formed 5-LO products were analysed by reverse-phase HPLC as described^80^. 5-LO products include the all-*trans* isomers of LTB4 (trLTB4 isomers) and 5(*S*)-hydroperoxy-6-*trans*-8,11,14-*cis*-eicosatetraenoic acid and its corresponding alcohol 5(*S*)-hydroxy-6-*trans*-8,11,14-*cis*-eicosatetraenoic acid.

### In vitro cleavage of peptide–lapachone prodrugs by cathepsin B

Cathepsin B cleavage of Cbz-dipeptide-protected prodrugs was tested in vitro as follows: cathepsin B (Abcam ab151914) was preactivated by dilution of enzyme stock (20 µl, 0.44 mg ml^−1^, 37 kDa, 12 µM) into activation buffer (45 µl, MES 25 mM pH 5 + 10 mM DTT). The reaction was incubated at 37 °C for 20 min. Reactions were made up containing the prodrug of interest (15 µl of a 5 mM stock in DMSO) and internal standard acetophenone (0.75 µl of a 100 mM stock in DMF) in MES 20 mM pH 5 buffer (114.25 µl) resulting in a total reaction volume of 150 µl at pH 5 with 10.5% DMSO, 500 µM prodrug and 0.5 µM of cathepsin B (1,000 equiv. of substrate compared to enzyme). A 20 µl sample of each reaction was removed immediately at *t* = 0. Following preactivation, activated cathepsin stock (20 µl) was added to test reactions. Cathepsin activation buffer only (MES 25 mM pH 5 + 10 mM DTT, 20 µl) was added to negative control samples. Samples were then incubated with shaking (500 r.p.m.) at 37 °C. Subsequent 20 µl samples were removed at specified time points and analysed by HPLC (254 and 430 nm; instrument: ThermoFisher U3000; column: Phenomenex Kinetex C18, 5 µm, 50 × 4.6 mm, 100 Å; solvent system A = H_2_O, B = acetonitrile; flow, 1 ml min^−1^; gradient: *t* = 0–1.0 min 0% B, *t* = 1.0–5.0 min 0–40% B, *t* = 5.0–12.0 min 40% B, *t* = 12.1–14.0 min 100% B, *t* = 14.1–15.0 min 0% B). Spectra at 254 nm were normalized to the height of the internal standard; spectra at 430 nm were unnormalized.

### Antibody expression and purification

The variable heavy-chain and light-chain sequences for gemtuzumab were identified as previously described^81–83^. Strings of the variable domains were produced from Geneart, Invitrogen and cloned into mammalian expression vectors encoding either standard human IgG1 constant regions or site-specific cysteine variants such as Gem-LC-V205C, Gem-HC-239iC and Gem-HC-S442C using NEBuilder assembly from NewEnglandBiolabs. Antibodies were transiently expressed in Chinese hamster ovary cells under serum-free conditions as previously described^84^. Cleared culture supernatant was loaded directly onto MapSelect SuRe column equilibrated with PBS (pH 7.2). Antibodies were step eluted with 0.1 M glycine pH 2.7. Pooled fractions were buffer exchanged into PBS.

### Antibody–linker conjugation reactions

Antibodies in PBS pH 7.2 + 1 mM EDTA buffer were refolded prior to reaction. The refolding procedure was performed as follows. First, antibodies were reduced with tris(2-carboxyethyl)phosphine hydrochloride (20 equiv., to a final concentration of 10% DMF) for 30 min at 37 °C. Antibodies were desalted with Zeba spin desalting columns (0.5 ml, 7 kDa molecular weight cut-off, size exclusion columns, ThermoFisher) into PBS pH 7.2 + 1 mM EDTA. (l)-Dehydroascorbic acid (20 equiv., to a final concentration of 10% DMF) was then added and the antibodies were incubated for 3.5 h at 25 °C. Antibodies were desalted with Zeba spin desalting columns into NaPi 20 mM pH 8. Refolding and reactivity was assessed by reaction of a sample of each antibody (10 µl containing 10 µM antibody) in NaPi 20 mM pH 8 with *N*-ethyl benzoylacrylic acid (5 equiv. per cysteine + 1 µl of a stock in DMF) for 30 min at 37 °C, according to a published protocol^66^. Antibody–drug linker conjugations were performed by incubation (with shaking) of refolded antibody stock with the specified equivalent of linker for the specified time period in a buffer of NaPi 20 mM pH 8 and a final concentration of DMF of 10%. After completion of the reaction, antibodies were desalted with Zeba spin desalting columns (ThermoScientific) into PBS pH 7.4. Following reaction, a sample of conjugate was subject to reduced LC–MS analysis to assess conversion. The concentration of the conjugates was determined by ultraviolet absorbance at 280 nm (*A*_280_), as measured using a SpectraDrop reader (MiniMax i3x Imager, Molecular Devices). Ultraviolet absorbances were corrected by the expression *A*_280_ − (1.929 × *A*_330_) to account for light scattering.

### Antibody–conjugate integrity analysis

The stability of the antibody conjugates to deconjugation of payload at physiological temperature was measured by incubation of conjugate (10 µl of 20 µM stock) in PBS pH 7.4 buffer at 37 °C with shaking. After 48 h, 10 µl aliquots were taken and analysed for percentage conjugated to unconjugated antibody by reduced LC–MS analysis. Conjugate stability to storage at 4 °C in PBS pH 7.4 buffer over an extended period (3–4 weeks) was assessed. Stability of conjugate to deconjugation of payload in human serum was assessed by incubation of conjugate (10 µl of 20 µM stock) with 1 µl of human serum in PBS pH 7.4 buffer at 37 °C. After 48 h, 10 µl aliquots were taken and analysed for percentage conjugated to unconjugated antibody by reduced LC–MS analysis.

### General cell culture conditions

Cells were incubated in a humidified 10% CO_2_/90% air atmosphere at 37 °C. HL-60 cells were cultured in RPMI medium (Gibco) with 10% heat-inactivated fetal bovine serum. HEL cells were grown in RPMI media with 20% heat-inactivated fetal bovine serum. Leukaemia cells were maintained at a density of 1 × 10^6^ cells ml^−1^. Cells were split every second day to keep them in the exponential growth phase. SKBR-3 and HCT-116 cells were grown in McCoy’s 5A Modified Medium (Gibco) with 10% heat-inactivated fetal bovine serum. MCF-7 cells were grown in Dulbecco’s Modified Eagle Medium (Gibco) with 10% heat-inactivated fetal bovine serum. Adherent cell lines were passaged by addition of trypsin–EDTA (0.25%) (Gibco). HL-60 was kindly donated from the group of Prof. Bruno Silva-Santos, iMM Lisbon. HEL was kindly donated from the group of Prof. Bruno Silva-Santos from iMM, Lisbon (in vivo studies) or Dr Isaia Barbieri, Department of Pathology, University of Cambridge (in vitro ADC studies). HeLa, SKBr3 and HCT-116 were purchased from ATCC. MCF-7 was kindly donated from the group of Dr Sérgio Almeida, iMM Lisbon.

### Viability assays with small-molecule lapachone models

Assays assessing cytotoxicity of β-lapachone and derivatives were performed by CellTiter-Blue assay (Promega) according to the manufacturer’s instructions. Briefly, adherent cancer-cell lines were plated at 20,000 cells per well in 96-well plates or suspension cell lines were plated at a concentration of 2 × 10^5^ cells ml^−1^ in 24-well plates. Compound stocks in media were made by dilution of DMSO stocks containing the compound of interest to obtain a final in-plate DMSO concentration of only 0.1%. Incubation was performed for 48 h. Following incubation, media was replaced with media containing CellTiter-Blue (Promega) in 1:10 dilution and the plates were incubated for 1.5–4 h. The fluorescence of the plates was recorded (excitation, 555 nm; emission, 585 nm; MiniMax i3x Reader, Molecular Devices). Cell viability was calculated by division of the fluorescence intensity of treated wells by that of the calculated average fluorescence intensity of replicate negative control wells containing cells with 0.1% DMSO only. IC_50_ values were calculated using GraphPad Prism 8 software.

### *N*-Acetyl cysteine assay

For assays involving cytotoxicity of β-lapachone derivatives with and without antioxidant *N*-acetyl cysteine, HL-60 cells were plated into 24-well plates at a concentration of 2.5 × 10^5^ cells ml^−1^ in 450 µl. To each well was then added 25 µl of drug diluted in PBS to obtain the correct in-plate drug concentration, and 25 µl of PBS or a stock of *N*-acetyl cysteine at 12 mM in PBS. DMSO was maintained at an in-plate concentration of 0.1%. After 96 h incubation, toxicity was assessed by counting of live cells using the trypan blue exclusion method with a Countess II Automated Cell Counter (ThermoFisher Scientific) according to the manufacturer’s instructions. Cell viability was determined by dividing the average live cell number of treated wells by the average live cell number for control wells treated with 0.1% DMSO. Viability for wells treated with *N*-acetyl cysteine was assessed by comparison with control wells treated with *N*-acetyl cysteine and 0.1% DMSO. IC_50_ values were calculated using GraphPad Prism 8 software.

### Viability assays with small-molecule prodrugs and ADCs using the trypan blue exclusion method

For assays assessing cytotoxicity of β-lapachone versus small-molecule prodrugs, antibodies and ADCs, suspension cells were plated into 24-well plates at a density of 2.5 × 10^5^ cells ml^−1^ in 240 µl. Stocks of ADC or antibody in PBS were normalized to 12.5 µM. Serial dilutions of compound were made up in PBS. Then, 60 µl of each stock of either PBS or compound in PBS was added to the cells to achieve the desired final concentration. After the specified incubation time, toxicity was assessed by counting of live cells using the trypan blue exclusion method with a Countess II Automated Cell Counter (ThermoFisher Scientific) according to the manufacturer’s instructions. Cell viability was determined by dividing the average live cell number of treated wells by the average live cell number for control wells treated with PBS only. IC_50_ values were calculated using GraphPad Prism software. Note: for the assay performed over 96 h, 5% human serum was added to media instead of 10% fetal bovine serum, to limit non-specific antibody internalisation. This had little effect on ADC toxicity.

### Lysosomal inhibition assay with small molecule prodrugs

Assays to assess the effect of lysosomal activity on prodrugs were performed as follows: HL-60 cells were plated into 96-well plates at a concentration of 2.5 × 10^5^ cells ml^−1^ in 180 µl media. Cells were treated with 10 µl of a stock of NH_4_Cl in PBS (to obtain an in-plate concentration of 3 or 30 mM), 10 µl of a dilution of a DMSO stock of E64d in PBS (to obtain an in-plate concentration of 30 μM), PBS only or a dilution of DMSO in PBS. Cells were incubated for 30 min. Following this, 10 µl of a stock of test compound made by dilution of a DMSO stock into media was added. DMSO was present in-plate at a maximum of 0.2%. Cells were incubated overnight for 16 h. Following incubation, media was replaced with media containing CellTiter-Blue (Promega) in 1:4 dilution and the plates were incubated for 6 h. The fluorescence of the plates was recorded (excitation, 555 nm; emission, 585 nm; MiniMax i3x Reader, Molecular Devices). Cell viability was calculated by division of the fluorescence intensity of treated wells by that of the average fluorescence intensity of the relevant negative control wells.

### Protein mass spectrometry

LC–MS to analyse protein samples was performed on a SQ Detector 2 connected to an Acquity UPLC system using an Acquity UPLC BEH300 C4 column (1.7 μm, 2.1 mm × 50 mm). Flow rate was set at 0.2 ml min^−1^ with eluents of solvent A (water with 0.1% formic acid) and solvent B (71% acetonitrile, 29% water with 0.075% formic acid). The gradient was from solvent A/B (72:28) to 100% B over 25 min, followed by solvent B for 2 min and then up to solvent A/B (72:28) over 18 min. A capillary voltage of 2.0 kV and a cone voltage of 40 V were used for the electrospray source. The desolvation gas was nitrogen at a total flow of 850 l h^−1^. Mass spectra were reconstructed using the MaxEnt algorithm preinstalled on MassLynx software (Waters) from the ion series. To obtain the ion series described, the major peak(s) of the chromatogram were selected for integration and further analysis.

### Native protein MS

Native MS evaluations were performed on a Synapt High Definition Mass Spectrometer (Waters). All protein samples were buffer exchanged into 200 mM aqueous ammonium acetate buffer solution in water using Zeba spin desalting columns (0.5 ml, 7 kDa molecular weight cut-off, size-exclusion columns, ThermoFisher). The samples were further diluted to 2–10 µM before native MS evaluation. Sample aliquots of 2.5 µl were transferred to a borosilicate emitter (Thermo Scientific) before being introduced into the mass spectrometer. The instrument was set up for the detection of high mass complexes. The conditions used for these experiments were: capillary voltages, 1.6–2.5 kV; sample cone voltages, 100–180 V; source temperature, 80 °C, extraction cone voltage, 3–8 V; nanoflow gas pressure, 0.02–0.5 bar; backing pressure, 3.5–3.9 mbar; trap pressure, 3.2 × 10^2^ mbar, ion mobility spectrometry (N_2_) pressure, 6.1 × 10^–1^ mbar; and time-of-flight pressure, 7.1 × 10^–7^ mbar. Spectra were calibrated externally using caesium iodide. Data acquisition and processing were performed using MassLynx 4.1 (Waters).

### In vivo AML xenograft study

The AML cell line used in this study, HEL cells, were a kind donation of Prof. Bruno Silva-Santos, iMM, Lisbon. The cells were maintained in a humidified incubator at 37 °C under 5% CO_2_ and grown using 1× RPMI 1640 medium without l-glutamine (Invitrogen, Life Technologies) supplemented with 10% heat-inactivated fetal bovine serum (FBS) (Gibco, Thermo Scientific), 1× MEM NEAA (Gibco, Thermo Scientific), 1× sodium pyruvate (Gibco, Thermo Scientific), 1× GlutaMAX (Gibco, Thermo Scientific), 200 U ml^−1^ penicillin and 200 µg ml^−1^ streptomycin (Gibco, Thermo Scientific) and 10 mM HEPES (Gibco, Thermo Scientific). All animal experiments were conducted at the Instituto de Medicina Molecular João Lobo Antunes (Lisbon). Animal work was performed in strict accordance with Portuguese law (Portaria 1005/92) and European Guideline 86/609/EEC and follows the Federation of European Laboratory Animal Science Associations guidelines and recommendations concerning laboratory animal welfare. Furthermore, all animal experiments were approved by the Portuguese DGAV and the IMM Animal Ethics Committee (authorization AWB_2017_11_GB_AMLeukemia). A localized model of AML was established in 8-week-old female NOD-SCID mice (purchased from Charles River) by inoculating 5 × 10^6^ HEL cells subcutaneously in the flank. Tumour growth was monitored over time, by performing bilateral vernier caliper measurements every 3–4 d and mean tumour volumes were calculated using the formula (length × width^2^)/2. Treatments were initiated when tumours reached approximately 100 mm^3^ (approximately 10 d after tumour induction), with the mice having been randomly assigned to receive DAR-2 ADC or native antibody and PBS as controls. Treatments were administered intravenously in two injections with a 3 d interval. Animals were observed every 3–4 d; tumours were measured as described before and mouse weight was evaluated throughout the study. Once tumours reached 1,000 mm^3^ animals were killed, and maximal tumour burden was not exceeded. No signs of animal suffering or discomfort were observed, including no weight loss. The light/dark cycle was 14 h light/10 h dark (lights on at 07:00; lights off at 21:00). The temperature was 20–24 °C and the relative humidity was 55 ± 10%. The type of food was autoclaved diet pellets RM3A (P), from SDS Special Diets Services (product code 801030). Food was placed in a grid inside the cage and provided ad libitum to animals. The type of water was sterile water treated by reverse osmosis. Water was provided ad libitum to animals through bottles with a capillary hole. The data collected were analysed using GraphPad Prism8. Once the mice had been killed (by isoflurane overdose) comprehensive necropsy was performed. Macroscopic findings were recorded, and heart, lung, left and right kidney, liver, spleen, central nervous system and primary tumours were collected for histopathology. Samples were immersion fixed in 10% neutral buffered formalin, routinely processed for paraffin embedding, sectioned at 4 µm, and stained with haematoxylin and eosin. Lesions were classified according to previously published criteria (International Harmonization of Nomenclature and Diagnostic Criteria for Lesions in Rats and Mice) and scored according to a six-tier severity scale: 0, absent; 1, minimal; 2, mild; 3, moderate; 4, marked; 5, severe. Distant metastasis were scored according to a five-tier severity scale: 0, absent; 1, minimal; 2, mild; 3, moderate; 4, marked. Representative haematoxylin and eosin pictures were obtained using NDP.view2 software (Hamamatsu) in slides digitally scanned in the Hamamatsu NanoZoomerSQ (20× for heart, lung, liver, spleen, kidney and primary tumour; and 10× for the central nervous system).

### Quantitative LC–MS/MS of β-lapachone in formol-fixed xenograft mouse tumour and formol supernatant

Concentrations of β-lapachone in formalin-fixed tumour and the respective formol supernatant were quantified using reversed-phase ultra-high-performance liquid chromatography tandem mass spectrometry as previously described^85^. Care was taken during sample handling to minimize exposure to light, and amber glass/plastic and foil wrapping were used where possible. Tumour tissue was homogenized to a concentration of 200 mg ml^−1^ with PBS. An aliquot of homogenate or formol supernatant sample was transferred to a fresh tube with cryptotanshinone (internal standard) in ethyl acetate for liquid–liquid extraction. Samples were mixed, centrifuged and the ethyl acetate layer transferred to a 96-well plate, evaporated and reconstituted in 0.1% formic acid in acetonitrile:water 70:30. PBS was used as the surrogate matrix for calibration standards and quality-control samples. The calibration ranges were 0.25–250 ng g^−1^ (homogenate) and 0.05–50 ng ml^−1^ (formol). The analytical batches included quality-control samples at low, medium and high concentrations. The analysis was performed on a Shimadzu Nexera X2 UHPLC coupled to a Sciex TripleTOF 6600 mass spectrometer. The extracted samples were injected onto a Phenomenex Kinetex EVO C18, 1.7 µm, 50 × 2.1 mm analytical column, at 35 °C. Gradient elution was performed using 0.1% formic acid in water and an increasing percentage of 0.1% formic acid in acetonitrile at 0.5 ml min^−1^, with a total run time of 4 min. The mass spectrometer was operated with positive electrospray ionization and enhanced high-sensitivity product ion scans of 100–500 Da, from precursor ions *m*/*z* 243.0 (β-lapachone) and *m*/*z* 297.0 (cryptotanshinone). Extracted ion chromatograms were produced from product ions *m*/*z* 187.0358 (β-lapachone) and *m*/*z* 251.1411 (cryptotanshinone), with a range of ±0.025 Da. The peak area ratios from the integrated analyte and internal standard chromatograms were used to back-calculate the concentrations of β-lapachone against a linear 1/*x*^2^ weighted calibration curve.

### Quantum mechanical calculations

Full geometry optimizations were carried out with Gaussian 16 (ref. ^86^), using the M06-2X hybrid functional^87^ and 6-31+G(d,p) basis set in combination with ultrafine integration grids. Bulk solvent effects in water were considered implicitly through the IEF-PCM polarizable continuum model^88^. The possibility of different conformations was taken into account for all structures. All stationary points were characterized by a frequency analysis performed at the same level used in the geometry optimizations from which thermal corrections were obtained at 313.15 or 298.15 K. Frequency analyses were carried out at the same level used in the geometry optimizations, and the nature of the stationary points was determined in each case according to the appropriate number of negative eigenvalues of the Hessian matrix. The quasiharmonic approximation reported by Truhlar and co-workers was used to replace the harmonic oscillator approximation for the calculation of the vibrational contribution to enthalpy and entropy^89^. Scaled frequencies were not considered. Mass-weighted intrinsic reaction coordinate calculations were carried out using the Gonzalez and Schlegel scheme^90,91^ to ensure that the TSs indeed connected the appropriate reactants and products. Single-point energies were alternatively calculated on the optimized geometries using combinations of different density functionals (M06-2X and ωB97x-D^92^), basis sets (6-31+G(d,p) and 6-311++G(2d,p)) and implicit solvation models (IEF-PCM and SMD^93^)) (Supplementary Table 4). Gibbs free energies (Δ*G*) were used for the discussion on the relative stabilities of the considered structures. The lowest-energy conformer for each computed stationary point was considered in the calculation of the elimination activation barriers; all the computed structures can be obtained from authors upon request. Computed molecular structures were depicted using open-source PyMol 2.3 (https://pymol.org). Electronic energies, entropies, enthalpies, Gibbs free energies and lowest frequencies of the calculated structures are summarized in Supplementary Table 5. Cartesian coordinates of the lowest-energy structures calculated with PCM(H2O)/M06-2X/6-31+G(d,p) are shown in Supplementary Table 6. Theoretical kinetic and equilibrium constants derived from fitting of experimental and computed data were calculated using Microcal Origin Pro 2020b.

### Reporting summary

Further information on research design is available in the Nature Research Reporting Summary linked to this article.

## Online content

Any methods, additional references, Nature Research reporting summaries, source data, extended data, supplementary information, acknowledgements, peer review information; details of author contributions and competing interests; and statements of data and code availability are available at 10.1038/s41557-022-00964-7.

## Supplementary information

Supplementary Information Synthetic procedures, Supplementary Figs. 1–112, Schemes 1–10, Tables 1–6 and Discussion.

Reporting Summary

## Data Availability

Data supporting the findings of this study are available within the paper and its Supplementary Information. The Supplementary Information reports experiments described within the manuscript in greater detail, and describes synthetic procedures and characterization data. Requests for materials should be addressed to G.L.J.B. and G.J.O. Source data of HPLC traces used to calculate the kinetics of PAB-PhQ, PAB-BL and PAB-DN elimination in aqueous solution are provided with the manuscript. All computed geometries, energies and fitting data can be accessed through the Zenodo repository (10.5281/zenodo.6325898). No restrictions on data availability apply. Source data are provided with this paper.

## Source data

Source Data Fig. 3 Excel used to generate 3a.
Source Data Fig. 4 Excel containing all experimental data for 4c.
