# Supplementary material for: Controlled masking and targeted release of redox-cycling ortho-quinones via a C–C bond-cleaving 1,6-elimination
Source: Nat Chem. 2022 Jun 27;14(7):754–65. doi: 10.1038/s41557-022-00964-7 (PMC9252919; doi:10.1038/s41557-022-00964-7)
Supplement: Supplementary file 1 — Synthetic procedures, Supplementary Figs. 1–112, Schemes 1–10, Tables 1–6 and Discussion. [file 41557_2022_964_MOESM1_ESM.pdf]

nature portfolio

https://doi.org/10.1038/s41557-022-00964-7

Supplementary information

Controlled masking and targeted release of redox-cycling *ortho*-quinones via a C–C bond-cleaving 1,6-elimination

In the format provided by the authors and unedited

Supplementary Information for

Controlled masking and targeted release of redox-cycling *ortho*-quinones via a C–C bond-cleaving 1,6-elimination

Lavinia Dunsmore[1], Claudio D. Navo[2#], Julie Becher[1#], Enrique Gil de Montes[1#], Ana Guerreiro[3#], Emily Hoyt[1], Libby Brown[1,4], Viviane Zelenay[4], Sigitas Mikutis[1], Jonathan Cooper[5], Isaia Barbieri[6], Stefanie Lawrinowitz[7], Elise Siouve[4,8], Esther Martin[4], Pedro R. Ruivo[3], Tiago Rodrigues[3], Filipa P. da Cruz[1], Oliver Werz[7], George Vassiliou[5], Peter Ravn[4,10], Gonzalo Jiménez-Osés*[2,9] & Gonçalo J. L. Bernardes*[1,3]

[1]Yusuf Hamied Department of Chemistry, University of Cambridge, Lensfield Road, CB2 1EW Cambridge, UK.

[2]Center for Cooperative Research in Biosciences (CIC bioGUNE), Basque Research and Technology Alliance (BRTA), Bizkaia Technological Park, Building 800, 48160 Derio-Bizkaia, Spain.

[3]Instituto de Medicina Molecular João Lobo Antunes, Faculdade de Medicina da Universidade de Lisboa, Av. Prof. Egas Moniz, 1649-028 Lisboa, Portugal.

[4]Biologics Engineering, R&D, AstraZeneca, Milstein Building, Granta Park, CB21 6GH Cambridge, UK.

[5]Wellcome-MRC Cambridge Stem Cell Institute, Department of Haematology, University of Cambridge, CB2 0AW Cambridge, UK.

[6]Department of Pathology, University of Cambridge, Division of Cellular and Molecular Pathology, Addenbrooke's Hospital, CB2 0QQ Cambridge, UK.

[7]Department of Pharmaceutical/Medicinal Chemistry, Institute of Pharmacy, Friedrich Schiller University Jena, Philosophenweg 14, 07743 Jena, Germany.

[8]Department of Chemical Engineering and Biotechnology, University of Cambridge, Philippa Fawcett Drive, CB3 0AS Cambridge, UK.

[9]Ikerbasque, Basque Foundation for Science, 48013 Bilbao, Spain.

[10]Current address: Department of Biotherapeutic Discovery, H. Lundbeck A/S
Ottiliavej 9, 2500 Valby (Denmark)

[#]These authors contributed equally.

*e-mail: gb453@cam.ac.uk; gjoses@cicbiogune.es

Contents

1. List of abbreviations and compounds **3**
2. Synthetic procedures **5**
3. Elimination from benzyl ketol species: characterisation and kinetic analysis **42**
4. Assays with non-fragmentable benzyl β-lapa-ketol **89**
5. β-lapachone and AML **92**
6. Assays with dipeptide prodrugs **99**
7. Antibody and ADC QC characterisation data **115**
8. *In-silico* modelling **169**
9. *C*-benzylation of *ortho*-quinones **202**
10. ^{1}H and ^{13}C NMR Spectra **204**
11. References **239**

1. List of abbreviations and compounds

**ALOX-5**	5-lipoxygenase
**Boc-PAB-BL**	Boc-*para*-aminobenzyl β-lapa-ketol, **9**
**Boc-PAB-CTN**	Boc-*para*-aminobenzyl cryptotanshi-ketol, **16**
**Boc-PAB-DN**	Boc-*para*-aminobenzyl dunni-ketol, **15**
**Boc-PAB-HBL**	Boc-*para*-aminobenzyl 3-hydroxy-β-lapa-ketol, **14**
**Boc-PAB-PhQ**	Boc-*para*-aminobenzyl phenanthrene-ketol, **6**
**BL**	β-lapachone, **1**
**CTN**	Cryptotanshinone, **13**
**DN**	Dunnione, **12**
**DTT**	Dithiothreitol
**h**	hours
**HBL**	3-hydroxy-β-lapachone, **11**
**HRP**	Horseradish peroxidase
**HQ**	Hydroquinone
**MES**	2-(N-morpholino)ethanesulfonic acid
**min**	minutes
**PAB-BL**	*para*-aminobenzyl β-lapa-ketol, **10**
**PAB-CTN**	*para*-aminobenzyl cryptotanshi-ketol, **19**
**PAB-DN**	*para*-aminobenzyl β-dunni-ketol, **18**
**PAB-HBL**	*para*-aminobenzyl 3-hydroxy-β-lapa-ketol, **17**
**PAB-PhQ**	*para*-aminobenzyl phenanthrene-ketol, **7**
**PenG**	Penicillin G Amidase
**PhQ**	9,10-Phenanthrenequinone **4**
**ROS**	Reactive oxygen species
**2-Phenylacetyl-PAB-BL**	2-phenylacetyl-*para*-aminobenzyl β-lapa-ketol, **22**
**2-Phenylacetyl-PAB-DN**	2-phenylacetyl-*para*-aminobenzyl dunni-ketol, **23**
**2-Phenylacetyl-PAB-PhQ**	2-phenylacetyl-*para*-aminobenzyl phenanthrene-ketol, **21**
**5-LO**	5-lipoxygenase

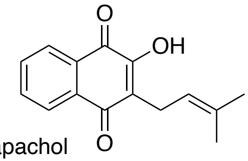
Lapachol

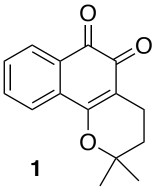
1

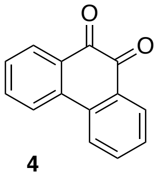
4

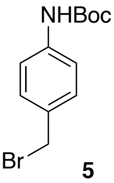
5

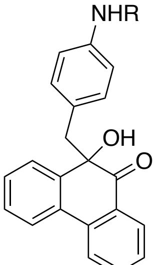
6 R: Boc
7 R: H
21 R: Phenylacetyl

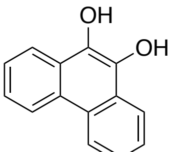
8

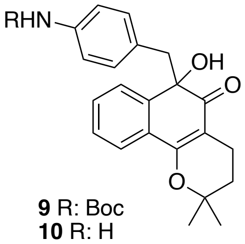
9 R: Boc
10 R: H
22 R: phenylacetyl

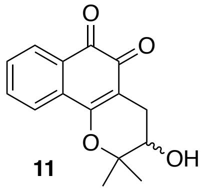
11

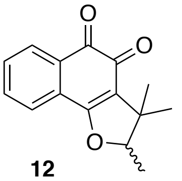
12

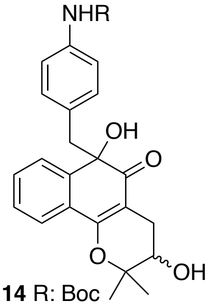
14 R: Boc
17 R: H

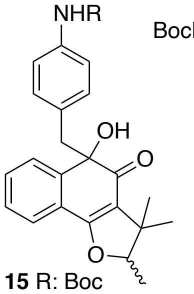
15 R: Boc
18 R: H
23 R: Phenylacetyl

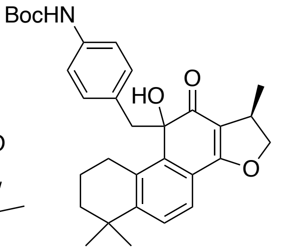
16 R: Boc
19 R: H

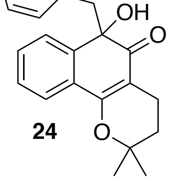
24

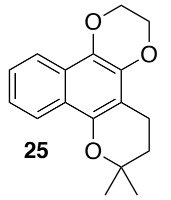
25

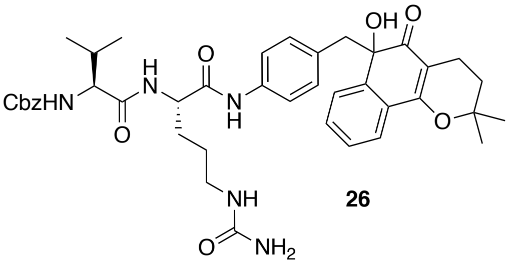
26

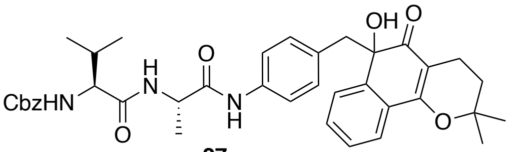
27

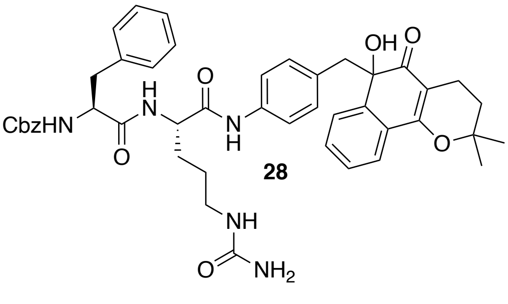
28

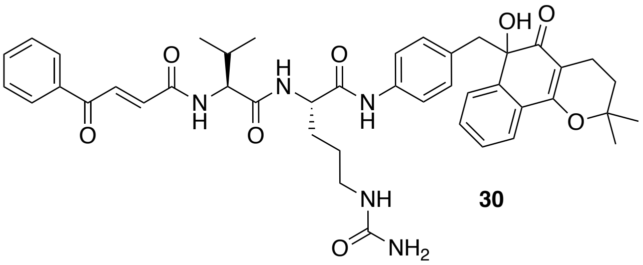
30

2. Synthetic procedures

Synthesis of β-lapachone 1

β-lapachone **1** was synthesised by the following synthetic route, from lawsone, via lapachol. This route was inspired by two known procedures (Inagaki *et al.*[1], Bian *et al.*[2]).

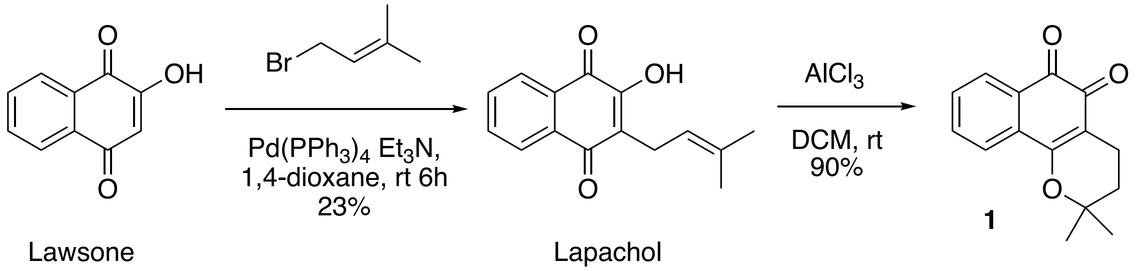

Scheme 1. Synthesis of β-lapachone.

A fast synthesis of β-lapachone **1** was also developed, avoiding purification of lapachol.

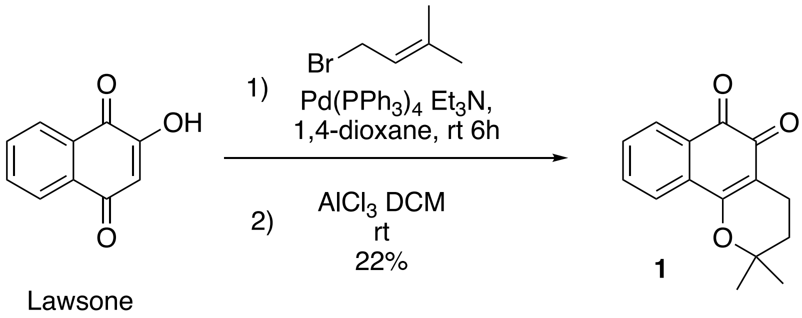

Scheme 2. Synthesis of β-lapachone, without purification of lapachol.

Lapachol

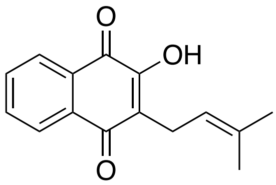

To a mixture of lawsone (2.00 g, 11.4 mmol), tetrakis(triphenylphosphine)palladium(0) (26 mg, 0.02 mmol) in dioxane (50 mL), 1-bromo-3-methyl-2butene (1.60 mL, 13.8 mmol) and triethylamine (1.92 mL, 13.8 mmol) were added. The reaction was stirred at room temperature for 6 hours. Then, water (40 mL) was added, and the crude was extracted into dichloromethane. Organic layers reunited were dried over $MgSO_4$ and filtered. The organic solvent was removed *in vacuo* and the resulting crude was purified by silica column (diethyl ether/hexane 1:8) affording lapachol (647.8 mg, 2.67 mmol, 23%) as a yellow powder. ^{1}H NMR (400 MHz, $CDCl_3$) δ 8.12 (d, *J* = 7.7 Hz, 1H), 8.07 (d, *J* = 7.6 Hz, 1H), 7.75 (t, *J* = 7.6 Hz, 1H), 7.67 (t, *J* = 7.5 Hz, 1H), 7.30 (s, 1H), 5.21 (t, *J* = 7.5 Hz, 1H), 3.31 (d, *J* = 7.4 Hz, 2H), 1.79 (s, 3H), 1.68 (s, 3H).

β-lapachone 1

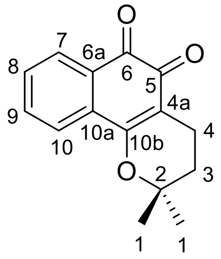

Synthesis from lapachol: To a solution of lapachol (638.8 mg, 2.63 mmol) in anhydrous dichloromethane (13 mL), under inert atmosphere, $AlCl_3$ (1.78 g, 13.3 mmol) was added. The reaction was stirred at room temperature for 1 h. Then the mixture was cooled at 0ºC and water (20 mL) was added dropwise. The crude was extracted into dichloromethane and organic layers reunited were dried over $MgSO_4$ and filtered. The organic solvent was removed in vacuo and the crude was purified by flash chromatography (Petroleum ether/ Ethyl acetate 4:1) to afford **1** (574.3 mg, 2.37 mmol, 90%) as a red solid.

Synthetic method avoiding purification of intermediate lapachol: To a solution of lawsone (1.00 g, 5.75 mmol) dissolved in 1,4-dioxane (25 mL) was added tetrakis(triphenylphosphine)palladium(0) (0.2 mol%, 13 mg), triethylamine (960 µL, 6.90 mmol) and 1-bromo-3-methyl-2-butene (800 µL, 6.90 mmol). The reaction mixture was stirred under a nitrogen atmosphere for 4 h. Following this, water (10 mL) was added and the reaction was extracted into dichloromethane (3 × 10 mL), dried (anhydrous magnesium sulphate), filtered, and solvent was removed *in vacuo*. The crude product was then redissolved in anhydrous dichloromethane (30 mL) and anhydrous aluminium trichloride (3.80 g, 28.5 mmol) was added. The reaction was stirred for 6 h at room temperature before being quenched by addition of water (30 mL). The crude product was isolated by extraction into dichloromethane (3 × 30 mL). The organic phase was dried (anhydrous magnesium sulphate), filtered and solvent was removed *in vacuo*. Flash column chromatography (4:1 petroleum ether/ethyl acetate) afforded β-lapachone **1** as a red solid (300 mg, 1.24 mmol, 22%). R_f 0.25 (4:1 petroleum ether/ethyl acetate, UV). ^1H NMR (400 MHz, $CDCl_3$): δ 8.03 (d, J = 7.7 Hz, 1H, H7), 7.79 (d, J = 7.8 Hz, 1H, H10), 7.62 (t, J = 7.6 Hz, 1H, H9), 7.48 (t, J = 7.6 Hz, 1H, H8), 2.55 (t, J = 6.6 Hz, 2H, H4), 1.84 (t, J = 6.7 Hz, 2H, H3), 1.45 (s, 6H, H1). ^{13}C NMR (101 MHz, $CDCl_3$) δ 179.9 (C6), 178.6 (C5), 162.1 (C10b), 134.9 (C9), 132.7 (C10a), 130.7 (C8), 130.2 (C6a), 128.6 (C7), 124.2 (C10), 112.8 (C4a), 79.4 (C2), 31.7 (C3), 26.8 (C1), 16.3 (C4). HRMS (m/z): $[M + H]^+$ calcd. For $C_{15}H_{14}O_3$ 243.1016, found 243.1008.

Synthesis of dunnione 12

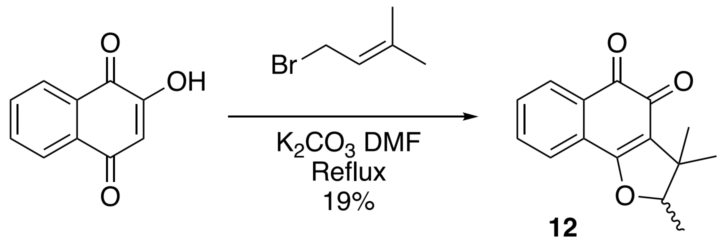

Lawsone

Scheme 3. Synthesis of dunnione.

The synthesis of Dunnione was carried out inspired by a previously described methodology.[3] A solution of Lawsone (2.00 g, 11.50 mmol) in DMF (15 mL) was added to a suspension of K_2CO_3 (1.60 g, 11.50 mmol) in DMF (55 mL). The mixture was stirred for 15 min. Then, a solution of 1-bromo-3-methyl-2-butene (3.30 mL, 28.70 mmol) in DMF (5 mL) was added dropwise at room temperature over 30 minutes and then, the reaction was refluxed for 8 hours. Following this, the reaction was cooled, filtered and poured into dichloromethane (30 mL). The crude was washed with water (5x25 mL), and the organic layer was dried over $MgSO_4$, filtered and the solvent was removed *in vacuo*. Flash column chromatography (Petroleum Ether/Ethyl Acetate 10:1→8:1) afforded (±)Dunnione, **12** (524 mg, 2,16 mmol, 19%) as a red solid. ^{1}H NMR (400 MHz, $CDCl_3$) δ 8.05 (d, *J* = 7.6 Hz, 1H), 7.66 – 7.61 (m, 2H), 7.59 – 7.50 (m, 1H), 4.67 (q, *J* = 6.6 Hz, 1H), 1.47 (d, *J* = 6.6 Hz, 3H), 1.45 (s, 3H), 1.26 (s, 3H). ^{13}C NMR (101 MHz, $CDCl_3$) δ 181.6, 175.5, 168.3, 134.6, 131.7, 130.9, 129.3, 128.0, 124.6, 123.4, 93.0, 44.2, 25.9, 20.5, 14.7. HRMS (m/z): $[M+H]^+$ calcd. for $C_{15}H_{14}O_3$ 243.1016, found 243.1026. NMR are in accordance with previously reported literature.[4]

3-hydroxy β-lapachone 11

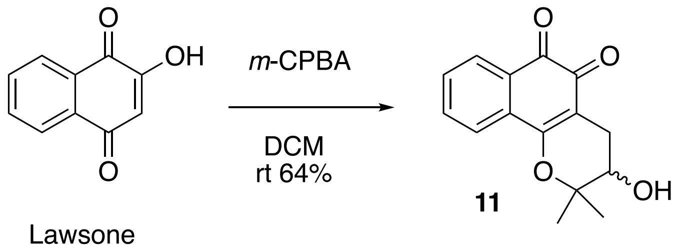

Scheme 4. Synthesis of 3-hydroxy-β-lapachone.

To a solution of lapachol (110 mg, 0.45 mmol) in anhydrous dichloromethane (20 mL) was added 3-chloroperoxybenzoic acid (145 mg, 77% (w/w), 0.65 mmol) and the mixture was stirred at room temperature for 24 hr. The reaction was quenched by addition of sodium hydrogen carbonate (20 mL), washed (water, 2 × 10 mL), dried (magnesium sulfate) and solvent was removed in vacuo. The crude reaction mixture was purified by flash column chromatography (1:1 dichloromethane/ethyl acetate) to obtain 3-hydroxy β-lapachone **11** as a red solid (74 mg, 0.29 mmol, 64 %). ^{1}H NMR (400 MHz, CDCl3) δ 8.04 (dd, *J* = 7.6, 1.4 Hz, 1H), 7.83 (dd, *J* = 7.9, 1.2 Hz, 1H), 7.64 (ddd, *J* = 7.7, 1.4 Hz, 1H), 7.50 (ddd, *J* = 7.6, 1.2 Hz, 1H), 3.93 (t, *J* = 5.2 Hz, 1H), 2.80 (dd, *J* = 17.7, 4.9 Hz, 1H), 2.61 (dd, *J* = 17.7, 5.4 Hz, 1H), 2.29 (s, 1H), 1.74 (s, 1H), 1.51 (s, 3H), 1.45 (s, 3H). ^{13}C NMR (101 MHz, CDCl3) δ 179.7, 178.9, 161.7, 135.0, 132.2, 131.1, 130.2, 128.9, 124.5, 110.6, 81.7, 68.4, 25.5, 25.2, 22.2. HRMS (m/z): $[M + H]^+$ calcd. for $C_{15}H_{16}O_4$, 259.0965 found 259.0960.

9,10-phenanthrenequinone 4

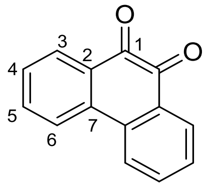

Characterisation of compound obtained commercially. ^{1}H NMR (600 MHz, MeOD) δ 8.25 (d, *J* = 8.1 Hz, 1H, H6), 8.16 (d, *J* = 7.8 Hz, 1H, H3), 7.80 (t, *J* = 8.0 Hz, 1H, H5), 7.54 (t, *J* = 7.4 Hz, 1H, H4). ^{1}H NMR (600 MHz, $CDCl_3$) δ 8.14 (dd, *J* = 7.8, 1.5 Hz, 1H, H6), 7.99 – 7.95 (m, 1H, H3), 7.69 (td, *J* = 7.7, 1.5 Hz, 1H, H5), 7.44 (td, *J* = 7.5, 1.1 Hz, 1H, H4).

9,10-phenanthrenediol 8

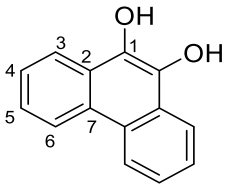

9,10-phenanthrenequinone **4** (1.00 g, 4.81 mmol) was dissolved in water/ether 1:1 (50 mL) and sodium dithionite (2.10 g, 85%, 10.25 mmol) was added to the solution. The reaction was stirred while being degassed with nitrogen for 1 h. Water (50 mL) was added to the reaction and the product was extracted with ethyl acetate (3 × 30 mL), dried (magnesium sulfate), filtered, and solvents were removed *in vacuo* to yield phenanthrene-9,10-diol, **8**, (860 mg, 4.10 mmol, 85%) as a brown-white solid. ^{1}H NMR (400 MHz, DMSO-d_6) δ 8.72 (s, 2H, OH), 8.35 (d, *J* = 8.3 Hz, 2H, H6), 7.90 (d, *J* = 8.2 Hz, 2H, H3), 7.30 (dd, *J* = 7.6 Hz, 2H, H5), 7.20 (dd, *J* = 7.6 Hz, 2H, H4). ^{13}C NMR (100 MHz, DMSO-d_6) δ 134.6, 127.7, 126.6, 126.2, 124.4, 122.9, 121.5, 39.5. HRMS (m/z): $[M – H]^-$ calcd. for $C_{14}H_{10}O_2$ 209.0603, found 209.0610.

Synthesis of *tert*-butyl (4-(bromomethyl)phenyl)carbamate 5

Linker *tert*-butyl (4-(bromomethyl)phenyl)carbamate was synthesised from 4-aminobenzyl alcohol by the following synthetic route.

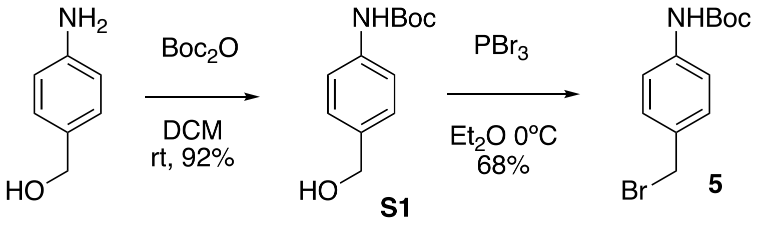

Scheme 5. Synthesis of *tert*-butyl (4-(bromomethyl)phenyl)carbamate.

***Tert*-butyl (4-(hydroxymethyl)phenyl)carbamate S1**

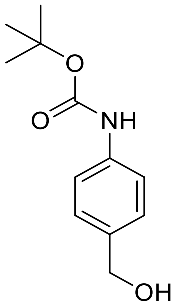

To a solution of *para*-aminobenzyl alcohol (1.00 g, 8.13 mg) in anhydrous dichloromethane (30 mL) under nitrogen was added di-*tert*-butyl dicarbonate (1.95 g, 8.94 mmol) dissolved in anhydrous dichloromethane (10 mL) and the solution was stirred at room temperature for 16 h. Following this, methanol (10 mL) was added and the reaction stirred for 10 min. Solvent was removed *in vacuo* to give the crude product. The crude product was purified by flash column chromatography (40–60 petroleum ether/ ethyl acetate 1:1) to yield *tert*-butyl (4-(hydroxymethyl)phenyl)carbamate as a yellow solid (1.67 g, 7.52 mmol, 92%). ^{1}H NMR (400 MHz, $CDCl_3$): δ 7.32 (d, *J* = 8.3 Hz, 2H), 7.25 (d, *J* = 8.6 Hz, 2H), 7.08 (s, 1H), 6.64 (s, 1H), 4.59 (s, 2H), 1.51 (s, 9H). ^{13}C NMR (100 MHz, $CDCl_3$): *δ* 152.9, 137.9, 136.0, 128.0, 118.8, 80.7, 77.5, 77.2, 76.8, 65.0, 28.5.

***Tert*-butyl (4-(bromomethyl)phenyl)carbamate 5**

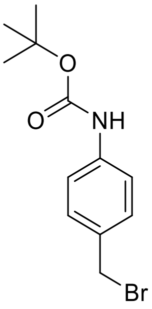

A solution of *tert*-butyl (4-(hydroxymethyl)phenyl)carbamate (1.04 g, 4.65 mmol) dissolved in anhydrous ether (20 mL) was cooled to 0 °C before addition of phosphorous tribromide (1.0 M in dichloromethane, 1.86 mmol, 1.86 mL) dropwise over 30 seconds. The reaction was stirred at 0 °C for 30 min, when thin-layer chromatography indicated completion of the reaction. The solution was poured onto ice cold water (10 mL) and extracted with ether (3 × 20 mL). The organic phase was dried (sodium sulphate), filtered, and solvent was removed *in vacuo* to yield the crude product *tert*-butyl (4-(bromomethyl)phenyl)carbamate as an off-white solid (900 mg, 3.15 mmol, 68%). The product was used without further purification. ^{1}H NMR (400 MHz, $CDCl_3$): *δ* 7.35 – 7.28 (m, 4H, H2 & H3), 6.58 (s, 1H, NH), 4.47 (s, 2H, H5), 1.52 (s, 9H, H8). ^{13}C NMR (100 MHz, $CDCl_3$): *δ* 152.7, 138.7, 132.3, 130.0, 129.6, 118.7, 80.9, 33.8, 28.4. HRMS (m/z): [M – Br] calcd. for $C_{12}H_{16}NO_2$ 206.1181, found 206.1176.

Boc-*para*-aminobenzyl phenanthrene-ketol 6

tert-butyl (4-((9-hydroxy-10-oxo-9,10-dihydrophenanthren-9-yl)methyl)phenyl)carbamate

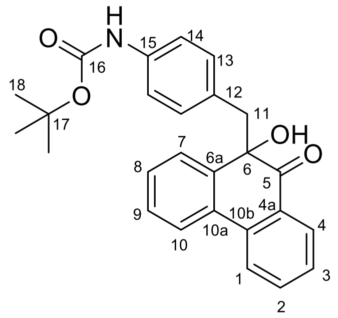

9,10-phenanthrenequinone **4** (500 mg, 2.40 mmol), sodium dithionite (2.50 g, 85%, 12.2 mmol) and tetrabutylammonium bromide (1.80 g, 5.6 mmol) were dissolved in a solution of tetrahydrofuran/water 1:1 (36 mL). Potassium hydroxide (2.70 g, 48 mmol, 20 equiv.) in water (2 mL) was then added, followed immediately by Boc-*para*-aminobenzyl bromide (1.40 g, 4.90 mmol) in tetrahydrofuran (2 mL). The reaction was then refluxed for 3.5 h. Water (60 mL) was added, and then the product was extracted into ethyl acetate (3 × 30 mL), washed (water, 1 x 30 mL), dried (magnesium sulfate), and solvent was removed *in vacuo*. The crude product was purified by flash column chromatography (petroleum ether/ethyl acetate 4:1) to yield Boc-*para*-aminobenzyl phenanthrene-ketol **6** (610 mg, 1.46 mmol, 61%) as a yellow solid. ^{1}H NMR (600 MHz, $CDCl_3$) δ 7.94 (d, *J* = 8.0 Hz, 1H, H1), 7.88 (dd, *J* = 7.6, 1.4 Hz, 1H, H4), 7.85 (dd, *J* = 7.8, 1.6 Hz, 1H, H10), 7.70 (td, *J* = 7.5, 1.5 Hz, 1H, H2), 7.61 (dd, *J* = 7.6, 1.6 Hz, 1H, H7), 7.44 (td, *J* = 7.4, 1.0 Hz, 1H, H3), 7.40 (td, *J* = 7.4, 1.6 Hz, 1H, H8), 7.36 (td, *J* = 7.5, 1.4 Hz, 1H, H9), 7.17 (d, *J* = 8.2 Hz, 2H, H14), 6.77 (d, *J* = 8.4 Hz, 2H, H13), 6.50 (s, 1H, NH), 4.01 (s, 1H, OH), 3.00 (d, *J* = 13.7 Hz, 1H, H11), 2.93 (d, *J* = 13.7 Hz, 1H, H11), 1.51 (s, 9H, H18). ^{13}C NMR (151 MHz, $CDCl_3$) δ 202.7 (C5), 152.8 (C16), 140.2 (C4a/C6a/C10a/C10b), 137.7 (C4a/C6a/C10a/C10b), 137.4, (C12), 135.2, (C2), 130.9 (C13), 129.3, (C9), 129.3, (C15), 129.2 (C4a /C6a/ C10a/C10b), 128.8 (C8), 128.6 (C4a/ C6a/C10a/C10b), 128.3, (C3), 127.49 (C4), 126.4 (C7), 124.1 (C10), 123.4 (C1), 117.8 (C14), 80.5 (C17), 79.4 (C6), 50.6 (C11), 28.5 (C18). HRMS (m/z): $[M – H]^-$ calcd. for $C_{26}H_{25}NO_4$ 414.1705, found 414.1706.

Boc-*para*-aminobenzyl β-lapa-ketol 9

tert-butyl (4-((6-hydroxy-2,2-dimethyl-5-oxo-3,4,5,6-tetrahydro-2H-benzo[h]chromen-6-yl)methyl)phenyl)carbamate

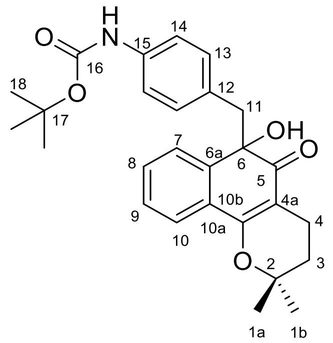

β-lapachone **1** (200 mg, 0.83 mmol), sodium dithionite (85%, 830 mg, 3.52 mmol) and tetrabutylammonium bromide (1350 mg, 4.04 mmol) were dissolved in a solution of tetrahydrofuran/water 1:1 (16 mL). The solution was heated to 80 °C over 10 min. Potassium hydroxide (900 mg, 16.0 mmol, 20 equiv.) in water (2 mL) was then added, followed immediately by Boc-*para*-aminobenzyl bromide (1300 mg, 4.56 mmol) in tetrahydrofuran (2 mL). The reaction was then refluxed for 4.5 h. Water (30 mL) was added, then the product was extracted into ethyl acetate (3 × 30 mL), washed (water, 1 x 30 mL), dried (magnesium sulfate), and solvent was removed *in vacuo*. The crude product was purified by flash column chromatography (petroleum ether/ethyl acetate 4:1) to yield Boc-*para*-aminobenzyl β-lapa-ketol **9** (270 mg, 0.60 mmol, 72%) as a yellow solid. ^{1}H NMR (400 MHz, $CDCl_3$): δ 7.65 (d, J = 7.8, 1H, H10), 7.59 (d, J = 7.8, 1H, H7), 7.43 (t, J = 7.6, 1H, H8), 7.32 (t, J = 7.6, 1H, H9), 7.06 (d, J = 8.1 Hz, 2H, H14), 6.51 (d, J = 8.5 Hz, 2H, H13), 6.38 (s, 1H, NH), 3.96 (s, 1H, OH), 3.01 (s, 2H, H11), 2.53 (dt, J = 17.3, 5.6 Hz, 1H, H4), 2.15 (ddd, J = 17.3, 8.6, 6.7 Hz, 1H, H4), 1.71 –1.60 (m, 2H, H3), 1.49 (s, 9H, H18), 1.35 (s, 3H, H1a/H1b), 1.12 (s, 3H, H1a/H1b). ^{13}C NMR (100 MHz, $CDCl_3$): δ 200.6 (C5), 162.3 (C10b), 152.7 (C16), 141.6 (C6a), 137.2 (C12), 130.2 (C8), 130.1 (C13), 129.7 (C10a), 127.6 (C15), 127.5 (C9), 125.8 (C7), 123.1 (C10), 117.7 (C14), 106.9 (C4a), 80.5 (C17), 78.3 (C6), 78.1 (C2), 53.9 (C11), 31.8, (C3), 28.4 (C18), 27.7 (C1a/C1b), 25.7 (C1a/C1b), 15.7. (C4). HRMS (m/z): $[M + H]^+$ calcd. for $C_{27}H_{31}NO_5$ 450.2275 found 450.2275.

Boc-*para*-aminobenzyl dunni-ketol 15

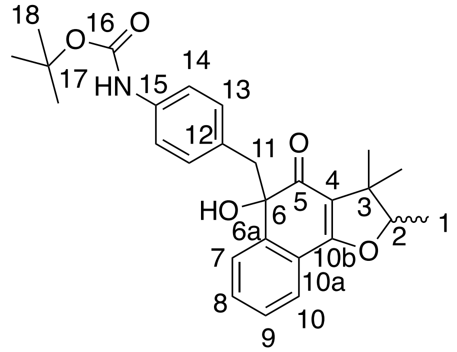

(±)Dunnione (75 mg, 0,31 mmol), sodium dithionite (85%, 258 mg, 1,26 mmol), and tetrabutylammonium bromide (417 mg, 1.50 mmol) were dissolved in a solution of THF/water 1:1 (5 mL). The solution was heated to 80 ºC over 10 min. Potassium hydroxide (337 mg, 6.00 mmol) in water (2.50 mL) was then added, followed immediately by Boc-*para*-aminobenzyl bromide **(5)** (479 mg, 1.68 mmol) in THF (2.50 mL). The reaction was then refluxed for 1.5 h. Water (15 mL) was added, then the product was extracted into ethyl acetate, washed with water, dried over $MgSO_4$, filtered and the solvent was removed *in vacuo*. The crude product was purified by flash column chromatography (Petroleum Ether/Ethyl acetate 6:1) to yield Boc-*para*-aminobenzyl dunni-ketol **15** (91.60 mg, 0.20 mmol, 64%) as a yellow foam. ^{1}H NMR (500 MHz, $CDCl_3$, mixture of diastereoisomer 2:1) δ 7.59 & 7.57 (2 ddd, *J* = 7.8, 1.3, 0.5 Hz, 1H, H10/7), 7.53 (ddd, *J* = 7.7, 1.4, 0.5 Hz, 1H, H10/7), 7.48 & 7.46 (2 td, *J* = 7.6, 1.4 Hz, 1H, H8/9), 7.38 & 7.34 (2 td, *J* = 7.5, 1.3 Hz, 2H, H8/9), 7.09 & 7.07 (2 d, *J* = 8.1 Hz, 2H, H14), 6.68 – 6.59 & 6.59 – 6.52 (2 m, 2H, H13), 6.41 & 6.38 (2 s, 1H, NH), 4.52 & 4.35 (2 q, *J* = 6.6 Hz, 1H, H2), 3.04 & 3.02 (2 d, *J* = 12.8 Hz, 1H, H11a), 3.01 & 2.98 (2 d, *J* = 12.8 Hz, 1H, H11b), 1.50 & 1.49 (2 s, 9H, H18), 1.38 & 1.16 (2 d, *J* = 6.6 Hz, 3H, H1), 1.35 & 1.30 (2 s, 3H, CH_3), 1.22 & 1.10 (s, 3H, CH_3).^{13}C NMR (126 MHz, $CDCl_3$, mixture of diastereoisomers) δ 197.6 & 197.1 (C5), 169.0 & 168.5 (C10b), 152.7 (C16), 145.0 & 144.9 (C6a), 137.2 (C12), 131.04 & 131.00 (CHAr), 130.9 & 130.7 (C13), 129.5 & 129.4 (CAr), 127.7 & 127.6 (CAr), 126.7 & 126.6 (CHAr), 123.70 & 123.4 (CHAr), 123.69 & 123.60 (CHAr), 117.9 & 116.9 (C4), 117.6 (C14), 92.6 & 91.9 (C2), 80.6 (C17) 79.8 & 79.4 (C6), 53.21 & 53.12 (C11), 44.0 & 43.8 (C3), 28.46 & 28.46 (C18), 28.1 & 24.2 (CH_3), 27.7 & 19.9 (CH_3), 15.9 & 13.7 (C1). HRMS (m/z): $[M+H]^+$ calcd. for $C_{27}H_{32}NO_5$ 450.2275, found 450.2285.

Boc-*para*-aminobenzyl 3-hydroxy-β-lapa-ketol 14

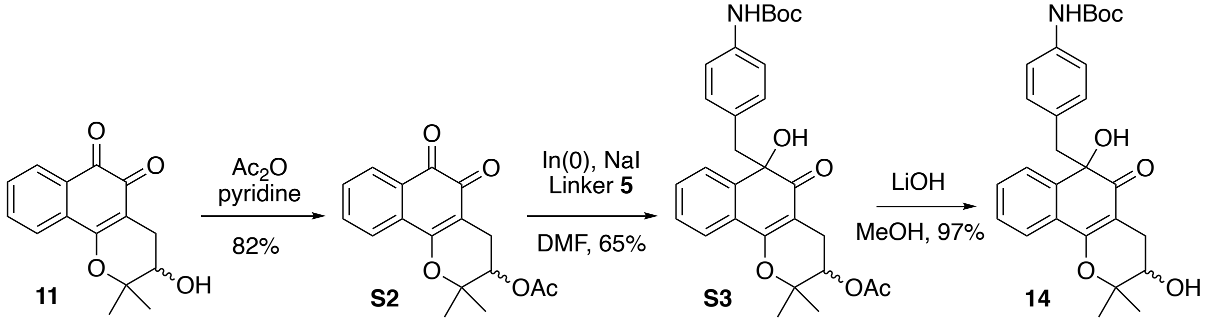

Scheme 6. Synthesis of Boc-PAB-HBL **14**.

(±) O-Acetyl-3-hydroxy-β-lapachone (S2)

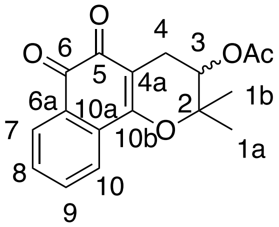

To a solution of hydroxy-β–lapachone **11** (85 mg, 0.33 mmol) in anhydrous pyridine (1.00 mL) acetic acid (0.50 mL) was added at 0ºC. The mixture was stirred at room temperature for 5 h. Then the pyridine was removed *in vacuo* and the resulting crude was purified by silica column (Petroleum ether/ ethyl acetate 1:6) to afford **S2** (79.90 mg, 0.27 mmol, 82%).^{1}H NMR (400 MHz, $CDCl_3$) δ 8.08 (d, *J* = 7.6 Hz, 1H, H7), 7.84 (d, *J* = 7.8 Hz, 1H, H10), 7.67 (td, *J* = 7.7, 1.4 Hz, 1H, H8), 7.53 (td, *J* = 7.6, 1.3 Hz, 1H, H9), 5.13 (t, *J* = 4.6 Hz, 1H, H3), 2.89 – 2.75 (dd, *J* = 18.2, 4.9Hz, 1H, H4), 2.75 – 2.61 (dd, *J* = 18.2, 4.2 Hz, 1H, H4), 2.07 (s, 3H, CH_3 Ac), 1.48 (s, 3H, H1), 1.43 (s, 3H, H1). ^{13}C NMR (101 MHz, $CDCl_3$) δ 179.5 (C6), 178.7 (C5), 170.2 (*C*$OOCH_3$), 161.3 (C10b), 135.0 (C8), 132.1 (C6a), 131.1 (C9), 130.2 (C10b), 129.0 (C7), 124.4 (C10), 110.2 (C4a), 79.8 (C2), 69.2 (C3), 25.0 (C1), 23.3 (C1), 22.8 (C4), 21.1 (COO*C*H_3). HRMS (m/z): [M+H]+ calcd. for $C_{17}H_{16}O_5$ 301.1021, found 301.1101.

($\pm$) Boc-*para*-aminobenzyl O-acetyl-β-lapa-ketol (S3)

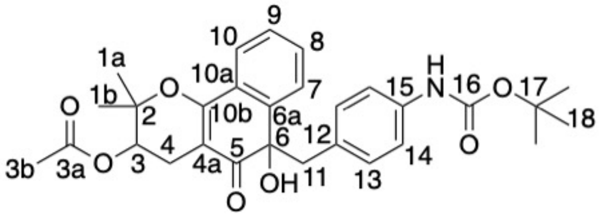

Boc-*para*-aminobenzyl bromide **5** (0.140 g, 0.49 mmol, 2.9 eq), NaI (0.210 g, 1.4 mmol, 8.5 eq), **S2** (0.050 g , 0.16 mmol, 1 eq) and indium(0) powder (0.043 g, 0.38 mmol, 2.3 eq) were added to anhydrous dimethylformamide (4 mL). The solution was heated to 40°C and sonicated for 3 hr, while monitoring by thin-layer chromatography (1:1 ethyl acetate: petroleum ether). Water (10 mL) and 7 drops 1M HCl were added to quench the reaction and it was extracted with ethyl acetate (3 x 150 mL). The organic layers were combined and washed with brine (10 mL), dried (sodium sulfate), and the solvent removed *in vacuo*. The product was purified by flash column chromatography on silica gel 60 (20-30% ethyl acetate: petroleum ether) to give ($\pm$) Boc-*para*-aminobenzyl O-acetyl-β-lapa-ketol **S3** as an orange solid (0.054 g, 0.1 mmol, 65%). ^{1}H NMR (500 MHz, $CDCl_3$, mixture of diastereoisomers A:B 1:2) δ 7.70 (dd, *J* = 7.9, 1.3 Hz, 1H, AH7), 7.67 (dd, *J* = 6.4, 1.4 Hz, 1H, BH7), 7.66 (dd, *J* = 6.2, 1.4 Hz, 1H, BH10), 7.58 (dd, *J* = 7.7, 1.3 Hz, 1H, AH10), 7.48 (td, *J* = 7.6, 1.3 Hz, 1H, BH8), 7.45 (td, *J* = 7.6, 1.4 Hz, 1H, AH8), 7.35 (tdd, *J* = 7.6, 5.0, 1.3 Hz, 1H, AH9/BH9), 7.10 (d, *J* = 8.1 Hz, 2H, AH14), 7.05 (d, *J* = 8.1 Hz, 2H, BH14), 6.57 (d, *J* = 8.5 Hz, 2H, AH13), 6.46 (brs, 1H, A/B N$\underline{H}$), 6.45 (d, *J* = 8.5 Hz, 2H, BH13), 4.96 (dd, *J* = 6.7, 5.4 Hz, 1H, AH3), 4.92 (dd, *J* = 4.8, 3.4 Hz, 1H, BH3), 3.03 (d, *J* = 2.1 Hz, 2H, BH11), 3.01 (s, 2H, AH11), 2.87 (dd, *J* = 17.3, 5.4 Hz, 1H, AH4), 2.61 (dd, *J* = 18.0, 3.4 Hz, 1H, BH4), 2.36 (dd, *J* = 17.9, 4.8 Hz, 1H, BH4), 2.19 (dd, *J* = 16.7, 6.1 Hz, 1H, AH4), 2.08 (s, 3H, AH3b), 1.99 (s, 3H, BH3b), 1.49 (s, 9H, AH18), 1.48 (s, 9H, BH18), 1.35 (s, 3H, AH1a/1b), 1.33 (s, 3H, BH1a/1b), 1.16 (s, 3H, AH1a/1b), 1.01 (s, 3H, BH1a/1b). ^{13}C NMR (126 MHz, $CDCl_3$, mixture of diastereoisomers A:B 1:2) δ 200.6 (BC5), 200.4 (AC5), 170.3 (BC3a), 170.0 (AC3a), 161.5 (BC10b), 161.1 (AC10b), 152.6 (A/B C16), 141.9 (AC6a), 141.5 (BC6a), 137.3 (A/B C12), 130.5 (BC8), 130.4 (AC8), 130.3 (AC13), 130.0 (BC13), 129.4 (BC15), 129.4 (AC15), 127.6 (BC9), 127.6 (AC9), 127.1 (BC10a), 126.6 (AC10a), 125.9 (BC10), 125.9 (AC10), 123.4 (AC7), 123.1 (BC7), 117.8 (A/B C14), 104.6 (AC4a), 104.4 (BC4a), 80.5 (A/B C17), 78.6 (A/B C2), 78.4 (BC6), 78.3 (AC6), 70.2 (AC3), 69.2 (BC3), 54.0 (BC11), 53.5 (AC11), 28.4 (A/B C18), 25.3 (AC1a/b), 24.2 (BC1a/b), 23.5 (BC1a/b), 22.2 (AC4), 22.0 (BC4), 21.5 (AC1a/b), 21.0 (A/B C3b). HRMS (m/z): [M+H]+ calcd. for $C_{29}H_{34}NO_7$ 508.2330, found 508.2324.

Boc *para*-aminobenzyl 3-hydroxy-β-lapa-ketol (14)

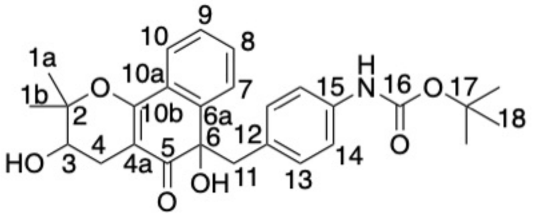

S3 (0.054 g, 0.11 mmol, 1 eq) was dissolved in tetrahydrofuran (2 mL) and methanol (2 mL) and stirred at 0ºC. A separate solution of lithium hydroxide monohydrate (9 mg, 0.21 mmol, 2 eq) in water (2 mL) was prepared and added dropwise to the solution over 5 min. The mixture was stirred at 0ºC for 1.5 hr until completion and then acetic acid (12.3 μL, 0.21 mmol, 2 eq) was added to quench the reaction. The solvent was removed *in vacuo*, and the residue was redissolved in ethyl acetate (25 mL). The organic layer was washed with water (10 mL), brine (10 mL), dried (Na_2SO_4), and the solvent removed *in vacuo* to give Boc-*para*-aminobenzyl 3-hydroxy-β-lapa-ketol **14** as a pink solid (0.048 g, 0.104 mmol, 97%). ^{1}H NMR (500 MHz, $CDCl_3$, mixture of diastereoisomers A:B 1:2) δ 7.65 (dd, *J* = 4.3, 1.5 Hz, 1H, A/B H7), 7.64 (dd, *J* = 4.3, 1.3 Hz, 1H, A/B H10), 7.46 (qd, *J* = 7.4, 1.3 Hz, 1H, A/B H8), 7.33 (qd, *J* = 7.3, 1.3 Hz, 1H, A/B H9), 7.03 (dd, *J* = 13.5, 7.8 Hz, 2H, A/B H14), 6.60 (brs, 1H, A/B N<u>H</u>), 6.46 (d, *J* = 8.5 Hz, 2H, AH13), 6.43 (d, *J* = 8.5 Hz, 2H, BH13), 3.64 – 3.59 (m, 1H, A/B H3), 3.02 (s, 2H, A/B H11), 2.72 (dd, *J* = 17.1, 5.3 Hz, 1H, AH4), 2.53 (dd, *J* = 17.4, 4.6 Hz, 1H, BH4), 2.34 (dd, *J* = 17.4, 4.7 Hz, 1H, BH4), 2.10 (dd, *J* = 16.9, 6.8 Hz, 1H, AH4), 1.47 (s, 9H, A/B H18), 1.36 (s, 3H, BH1a/1b), 1.32 (s, 3H, AH1a/1b), 1.09 (s, 3H, AH1a/1b), 1.01 (s, 3H, BH1a/1b). ^{13}C NMR (126 MHz, $CDCl_3$, mixture of diastereoisomers A:B 1:2) δ 201.1 (BC5), 200.9 (AC5), 161.9 (BC10b), 161.8 (AC10b), 152.9 (A/B C16), 141.7 (AC6a), 141.6 (BC6a), 137.3 (AC12), 137.2 (BC12), 130.5 (AC8), 130.5 (BC8), 130.0 (AC13), 129.9 (BC13), 129.6 (BC15), 129.4 (AC15), 127.7 (AC9), 127.6 (BC9), 127.1 (BC10a), 126.9 (AC10a), 125.9 (AC10), 125.9 (BC10), 123.3 (AC7), 123.2 (BC7), 118.0 (A/B C14), 105.2 (AC4a), 104.9 (BC4a), 80.5 (A/B C17), 80.3 (A/B C2), 78.4 (BC6), 78.3 (AC6), 69.0 (AC3), 68.1 (BC3), 53.9 (BC11), 53.8 (AC11), 28.4 (A/B C18), 25.3 (AC1a/1b), 24.8 (AC4), 24.7 (BC4), 24.5 (BC1a/1b), 22.6 (BC1a/1b), 20.7 (AC1a/1b). HRMS (m/z): [M+H]+ calcd. for $C_{27}H_{32}NO_6$ 466.2224, found 466.2217.

Boc-*para*-aminobenzyl cryptotanshi-ketol 16

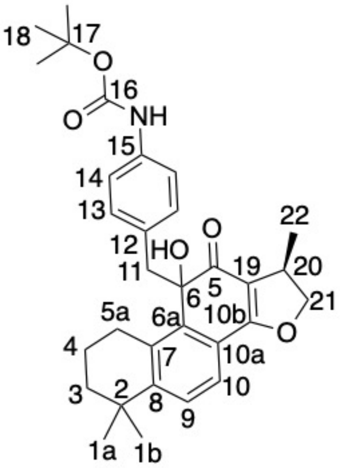

Cryptotanshinone **13** (152.6 mg, 0.51 mmol), sodium dithionite (85%, 439 mg, 2.14 mmol), and tetrabutylammonium bromide (708 mg, 2.55 mmol) were dissolved in a solution of THF/water 1:1 (5 mL). The solution was heated to 80 ºC over 10 min. Potassium hydroxide (572.3 mg, 10.2 mmol) in water (3.00 mL) was then added, followed immediately by Boc-*para*-aminobenzyl bromide **5** (807 mg, 2.83 mmol) in THF (3.00 mL). The reaction was then refluxed for one hour. Water (15 mL) was added, then the product was extracted into ethyl acetate, washed with water, dried over $MgSO_4$, filtered and the solvent was removed *in vacuo*. The crude product was purified by flash column chromatography (Petroleum Ether/Ethyl acetate 8:1) to yield Boc-*para*-aminobenzyl cryptotanshi-ketol **16** (206.9 mg, 0.41 mmol, 80%) as a yellow foam. ^{1}H NMR (500 MHz, $CDCl_3$, mixture of diastereoisomers A:B 1:2) δ 7.41 – 7.35 (m, 2H, A/B H9/10), 7.10 (d, *J* = 8.2 Hz, 2H, AH14), 7.07 (d, *J* = 8.2 Hz, 2H, BH14), 6.67 (d, *J* = 8.5 Hz, 2H, AH13), 6.58 (d, *J* = 8.5 Hz, 2H, BH13), 6.43 (s, 1H, ANH), 6.40 (s, 1H, BNH), 4.78 (dd, *J* = 10.0, 9.0 Hz, 1H, AH21), 4.52 (dd, *J* = 9.0, 9.0 Hz, 1H, BH21), 4.20 (dd, *J* = 9.0, 4.2 Hz, 1H, AH21), 4.09 (dd, *J* = 12.0, 9.2 Hz, 1H, BH21), 3.52 – 3.43 (m, 1H, AH20), 3.39 – 3.25 (m, 2H, A/B H5a, BH20), 3.23 – 3.13 (m, 2H, A/B H5a, A/B H11), 3.10 (d, *J* = 13.1 Hz, 1H, AH11), 3.02 (d, *J* = 12.9 Hz, 1H, BH11), 1.90 – 1.82 (m, 1H, A/B H4), 1.78 – 1.61 (m, 3H, A/B H4, A/B H3), 1.50 (d, *J* = 1.2 Hz, 9H, A/B H18), 1.37 (s, 3H, A/B H1a,1b), 1.30 (s, 3H, A/B H1a,1b), 1.29 (d, *J* = 6.8 Hz, 3H, AH22), 1.23 (d, *J* = 6.8 Hz, 3H, AH22). ^{13}C NMR (126 MHz, $CDCl_3$, mixture of diastereoisomers A:B 1:2) δ 197.1 (A/B C5), 171.9 (AC10b), 171.5 (BC10b), 152.8 (AC16), 152.7 (BC16), 151.3 (AC8), 151.2 (BC8), 141.6 (AC6a), 141.2 (BC6a), 137.4 (A/B C7), 137.1 (AC15), 137.0 (BC15), 130.9 (AC13), 130.4 (BC13), 129.9 (BC12), 129.5 (AC12), 127.0 (AC10), 126.9 (BC10), 121.8 (AC9), 121.6 (BC9), 121.4 (BC10a), 121.1 (AC10a), 117.5 (A/B C14), 112.9 (BC19), 112.5 (AC19), 82.4 (BC6), 81.8 (AC6), 81.0 (BC21), 80.8 (AC21), 80.5 (A/B C17), 49.9 (BC11), 49.1 (AC11), 38.8 (A/B C3), 35.4 (AC2), 35.3 (BC2), 34.6 (BC20), 34.4 (AC20), 32.6 (AC1a,1b), 32.6 (BC1a,1b), 29.4 (BC5a), 29.3 (AC5a),

28.4 (A/B C18), 20.0 (A/B C4), 19.4 (BC22), 18.0 (AC22). HRMS (m/z): $[M+H]^+$ calcd. for $C_{31}H_{38}NO_5$ 504.2744, found 504.2705.

***para*-aminobenzyl phenanthrene-ketol 7**

10-(4-aminobenzyl)-10-hydroxyphenanthren-9(10H)-one

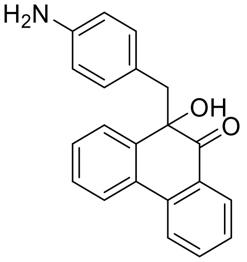

Boc-*para*-aminobenzyl phenanthrene-ketol **6** was dissolved in 4:1 dichloromethane/ trifluoroacetic acid at 0° C. The deprotection was followed by thin-layer chromatography until completion (30 min to 1 h). The residue was dried to offer *para*-aminobenzyl phenanthrene-ketol **7**. ^{1}H NMR (600 MHz, MeOD) δ 8.01 (d, J = 8.0 Hz, 1H), 7.96 (d, *J* = 7.8 Hz, 1H), 7.86 (dd, *J* = 7.7, 1.4 Hz, 1H), 7.72 (td, *J* = 7.7, 1.5 Hz, 1H), 7.50 – 7.44 (m, 2H), 7.42 (td, *J* = 7.6, 1.4 Hz, 1H), 7.31 (t, *J* = 7.5 Hz, 1H), 7.09 (d, *J* = 8.4 Hz, 2H), 6.90 (d, *J* = 8.4 Hz, 2H), 3.10 (d, *J* = 13.3 Hz, 1H), 3.02 (d, *J* = 13.3 Hz, 1H).

***para*-aminobenzyl β-lapa-ketol 10**

6-(4-aminobenzyl)-6-hydroxy-2,2-dimethyl-2,3,4,6-tetrahydro-5H-benzo[h]chromen-5-one

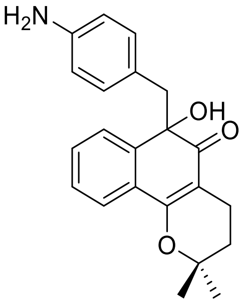

Boc-*para*-aminobenzyl β-lapa-ketol **9** was dissolved in 4:1 dichloromethane/trifluoroacetic acid at 0° C. The deprotection was followed by thin-layer chromatography until completion (30 min to 1 hour). The residue was dried to offer *para*-aminobenzyl β-lapa-ketol **10**. ^{1}H NMR (600 MHz, MeOD) δ 7.66 (d, *J* = 7.7 Hz, 1H), 7.62 (d, *J* = 7.9 Hz, 1H), 7.50 (t, *J* = 6.9 Hz, 1H), 7.37 (t, *J* = 7.7 Hz, 1H), 7.07 (d, *J* = 8.4 Hz, 2H), 6.70 (d, *J* = 8.4 Hz, 2H), 3.21 (d, *J* = 12.3 Hz, 1H), 3.10 (d, *J* = 12.4 Hz, 1H), 2.46 (dt, *J* = 17.3, 5.9 Hz, 1H), 2.16 (ddd, *J* = 17.3, 8.2, 6.4 Hz, 1H), 1.70 – 1.64 (m, 2H), 1.36 (s, 3H), 1.12 (s, 3H).

***para*-aminobenzyl 3-hydroxy-β-lapa-ketol 17**

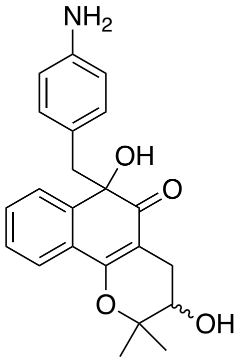

Boc-*para*-aminobenzyl 3-hydroxy-β-lapa-ketol **14** was dissolved in 4:1 dichloromethane/ trifluoroacetic acid at 0° C. The deprotection was followed by thin-layer chromatography until completion (30 min to 1 hour). The residue was dried to offer *para*-aminobenzyl β-lapa-ketol **17**. ^{1}H NMR (500 MHz, MeOD, mixture of diastereoisomers) δ 7.66 (td, *J* = 7.8, 1.3 Hz, 1H), 7.59 (dd, *J* = 7.8, 1.3 Hz, 1H), 7.49 (dtd, *J* = 16.6, 7.6, 1.4 Hz, 1H), 7.38 (tdd, *J* = 7.6, 3.2, 1.3 Hz, 1H), 7.07 (dd, *J* = 8.5, 7.2 Hz, 2H), 6.76 (d, *J* = 8.4 Hz, 1H), 6.71 (d, *J* = 8.5 Hz, 1H), 3.68 (dd, *J* = 8.0, 5.5 Hz, 1H), 3.63 (t, *J* = 4.9 Hz, 1H), 3.21 (d, *J* = 12.3 Hz, 2H), 3.10 (dd, *J* = 12.4, 3.0 Hz, 2H), 2.73 (dd, *J* = 17.0, 5.5 Hz, 1H), 2.63 (s, 1H), 2.43 (qd, *J* = 17.4, 4.9 Hz, 2H), 2.18 (s, 1H), 2.07 (dd, *J* = 17.0, 8.1 Hz, 1H), 1.36 (s, 3H), 1.25 (s, 3H), 1.08 (s, 3H), 1.04 (s, 3H).

***para*-aminobenzyl dunni-ketol 18**

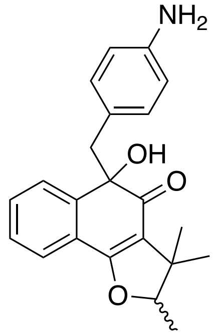

Boc-*para*-aminobenzyl dunni-ketol **15** was dissolved in 4:1 dichloromethane/trifluoroacetic acid at 0 ºC. The deprotection was followed by thin-layer chromatography until completion (30 min to 1 hour). The residue was dried to offer *para*-aminobenzyl β-lapa-ketol **18**. ^{1}H NMR (400 MHz, MeOD, mixture of diastereoisomers) δ 7.76 – 7.64 (m, 1H), 7.61 – 7.46 (m, 1H), 7.44-7.36 (m, 3H), 7.08 (m, 2H), 6.83 – 6.70 (m, 2H), 4.28 & 3.61 (2q, *J* = 6.7 Hz, 1H), 3.28 – 3.20 (m, 1H), 3.13 (m, 1H), 1.37 (d, *J* = 6.6 Hz, 3H), 1.31 (s, 3H), 1.19 – 1.08 (m, 3H).

para-aminobenzyl cryptotanshi-ketol 19

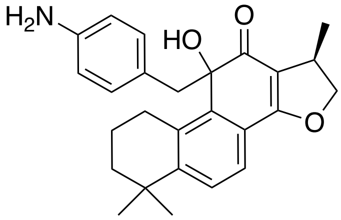

Boc-*para*-aminobenzyl cryptotanshi-ketol **16** was dissolved in 4:1 dichloromethane/ trifluoroacetic acid at 0° C. The deprotection was followed by thin-layer chromatography until completion (30 min to 1 hour). The residue was dried to offer *para*-aminobenzyl cryptotanshi-ketol **19**. ^{1}H NMR (500 MHz, MeOD, mixture of diastereoisomers) δ 7.51 (m, 1H), 7.43 – 7.38 (m, 1H), 7.15 – 7.02 (m, 2H), 6.86 – 6.71 (m, 2H), 4.83 – 4.80 & 4.54 – 4.47 (2m, 1H), 4.25 (dd, *J* = 9.2, 4.7 Hz, 1H), 4.10 – 4.06 (m, 1H), 3.58 – 3.42 (m, 2H), 3.30 – 3.13 (m, 3H), 1.92 – 1.66 (m, 4H), 1.42 (m, 3H), 1.34 (m, 3H), 1.23 (2d, 6.8 Hz, 3H).

Acetyl-β-lapa-hydroquinone 29

2,2-dimethyl-3,4-dihydro-2H-benzo[h]chromene-5,6-diyl diacetate

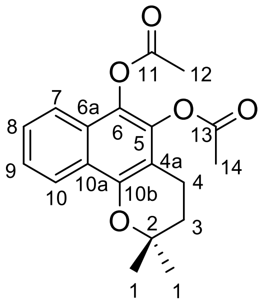

β-lapachone **1** (116 mg, 0.48 mmol), zinc (157 mg, 2.42 mmol) and sodium acetate (19.7 mg, 0.24 mmol) were refluxed in acetic anhydride (15 mL) for 2.5 hr. The reaction mixture was filtered free of solids, water was added (10 mL), and the product was extracted into ethyl acetate (3 × 10 mL), dried (anhydrous magnesium sulphate) and solvent was removed *in vacuo*. The crude product was purified by flash column chromatography (5:1 petroleum ether/ethyl acetate) to deliver acetyl-β-lapa-hydroquinone **29** as a white crystalline solid (131.8 mg, 0.402 mmol, 84%). To note, the flash chromatography was performed quickly as the compound is unstable to silica. ^{1}H NMR (400 MHz, $CDCl_3$): *δ* 8.21 (d, *J* = 7.3 Hz, 1H, H7), 7.68 (d, *J* = 7.5 Hz, 1H, H10), 7.45 (m, 2H, H8 & H9), 2.69 (t, *J* = 6.7 Hz, 2H, H4), 2.43 (s, 3H), 2.36 (s, 3H), 1.88 (t, *J* = 6.7 Hz, 2H, H3), 1.43 (s, 6H, H1). ^{13}C NMR (101 MHz, $CDCl_3$) δ 169.0 (C11/C13), 168.3 (C11/C13), 148.1 (C6), 138.6 (C5/C10b), 130.1 (C10a), 126.9 (C8/C9), 126.4 (C5/C10b), 125.3 (C8/C9), 124.4 (C6a), 122.3 (C7), 120.8 (C10), 109.5 (C4a), 75.0

(C2), 32.0 (C3), 26.8 (C1), 20.6 (C12/C14), 20.6 (C12/C14), 18.1 (C4). HRMS (m/z): $[M + Na]^+$ calcd. for $C_{19}H_{20}O_5$ 351.1203, found 351.1193.

β-lapa-hydroquinone 1,4-dioxine 25

7,7-dimethyl-2,3,5,6-tetrahydro-1,4,8-trioxatriphenylene

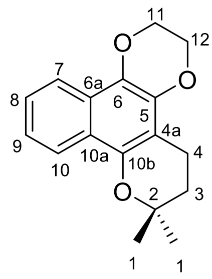

To β-lapachone **1** (70 mg, 0.28 mmol) dissolved in a solution of 1:1 water/tetrahydrofuran (10 mL) was added sodium dithionite (150 mg, 0.86 mmol) and tetrabutylammonium bromide (31 mg, 0.10 mmol). The reaction was stirred for 5 min at room temperature before addition of potassium hydroxide (130 mg, 2.32 mmol) dissolved in water (0.5 mL), followed immediately by 1,2-dibromoethane (176 μL, 2.04 mmol). The reaction was then refluxed at 100 °C. Further potassium hydroxide (95 mg, 1.70 mmol) was added after 10 min of reflux. The reflux was continued for 4.5 h after which the solution was cooled to room temperature. Water (30 mL) was added and the crude product was extracted into ethyl acetate (3 × 10 mL), dried (anhydrous magnesium sulphate), filtered and solvent was removed *in vacuo*. The crude product was purified by flash column chromatography (4:1 petroleum ether/ethyl acetate) to obtain 7,7-dimethyl-2,3,5,6-tetrahydro-1,4,8-trioxatriphenylene **25** as a white solid (34 mg, 0.13 mmol, 44%). ^{1}H NMR (400 MHz, $CDCl_3$): δ 8.12 (d, J = 8.4 Hz, 1H, H10), 7.96 (d, J = 8.4 Hz, 1H, H7), 7.42 (dd, J = 8.3, 6.8 Hz, 1H, H8), 7.32 (dd, J = 8.3, 6.8 Hz, 1H, H9), 4.38 (m, 4H, H11 and H12), 2.75 (t, J = 6.8 Hz, 2H, H4), 1.87 (t, J = 6.8 Hz, 2H, H3), 1.40 (s, 6H, H1). ^{13}C NMR (101 MHz, CDCl3): δ 143.2 (C10b), 137.4 (C5), 129.7 (C6), 125.5 (C8), 124.6 (C10a), 123.2 (C9), 121.5 (C10), 121.1 (C6a), 119.7 (C7), 107.4 (C4a), 74.0 (C2), 65.1 (C11/ C12), 64.3 (C11/C12), 32.4 (C3), 26.8 (C1), 17.5 (C4). HRMS (m/z): $[M]^+$ calcd. for $C_{17}H_{18}O_3$ 270.1251, found 270.1249.

Benzyl β-lapa-ketol 24

6-benzyl-6-hydroxy-2,2-dimethyl-2,3,4,6-tetrahydro-5H-benzo[h]chromen-5-one

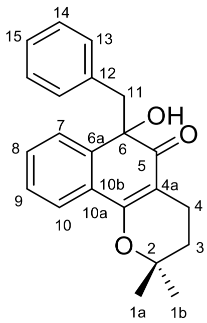

To β-lapachone **1** (30 mg, 0.12 mmol) dissolved in a solution of tetrahydrofuran (4 ml) was added sodium dithionite (107 mg, 0.61 mmol) and tetrabutylammonium bromide (198 mg, 0.61 mmol). The reaction was heated to 80 °C before the addition of potassium hydroxide (138 mg, 2.46 mmol) dissolved in water (6 mL), followed immediately by benzyl bromide (300 µL). The reaction was then refluxed at 100 °C for 4.5 h after which the solution was cooled to room temperature. Water (30 mL) was added, and the crude product was extracted into ethyl acetate (3 × 10 mL), dried (anhydrous magnesium sulphate), filtered, and solvent was removed *in vacuo*. The crude product was purified by flash column chromatography (4:1 petroleum ether/ethyl acetate) to obtain benzyl β-lapa-ketol **24** as a pale brown oil (16.8 mg, 0.05 mmol, 41%). ^{1}H NMR (400 MHz, $CDCl_3$): δ 7.65 (d, J = 7.8 Hz, 1H, H10), 7.62 (d, J = 7.8 Hz, 1H, H7), 7.45 (t, J = 7.6 Hz, 1H, H8), 7.34 (t, J = 7.6 Hz, 1H, H9), 7.08 (m, 3H, H14 & H15), 6.60 (d, J = 6.6 Hz, 2H, H13), 3.96 (s, 1H, OH), 3.07 (s, 2H, H11), 2.54 (dt, J = 17.3, 5.8 Hz, 1H, H4), 2.15 (dt, J = 17.3, 7.4 Hz, 1H, H4), 1.63 (dd, J = 7.4, 5.8 Hz, 2H, H3), 1.35 (s, 3H, H1a/H1b), 1.09 (s, 3H, H1a/H1b). ^{13}C NMR (101 MHz, $CDCl_3$): δ 200.6 (C5), 162.2 (C10b), 141.6 (C6a), 135.2 (C12), 130.1 (C8), 129.8 (C13), 127.7 (C15), 127.6 (C10a), 127.6 (C9), 126.8 (C14), 125.8 (C7), 123.0 (C10), 106.9 (C4a), 78.2 (C6), 78.0 (C2), 54.5 (C11), 31.7 (C3), 27.6 (C1a/C1b), 25.9 (C1a/C1b), 15.7 (C4). HRMS (m/z): $[M + Na]^+$ m/z calcd. for $C_{22}H_{22}O_3$ 335.1642, found. 335.1636.

2-phenyl-acetyl-*para*-aminobenzyl phenanthrene-ketol 21

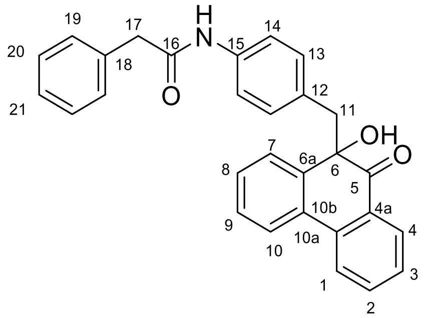

Boc-*para*-aminobenzyl phenanthrene-ketol **6** (70 mg, 0.169 mmol) was dissolved in dichloromethane/trifluoroacetic acid 4:1 (2 mL) at 0° C. The deprotection was followed by thin-layer chromatography until completion (30 min to 1 h). The residue was dried to offer *para*-aminobenzyl phenanthrene-ketol **7**, which was subsequently dissolved in anhydrous dichloromethane (5 mL). Phenyl acetic acid (114 mg, 0.838 mmol) was added followed by HATU (318 mg, 0.838 mmol) and triethylamine (1 mL) to achieve a reaction pH of 10. The reaction was stirred for 30 min. Following this, water was added (30 mL) and the product was extracted into ethyl acetate (3 × 10 mL), dried, and solvents were removed *in vacuo*. The crude product was purified by flash column chromatography (dichloromethane/methanol 9:1) to obtain 2-phenyl-acetyl-*para*-aminobenzyl phenanthrene-ketol **21** (23.9 mg, 0.055 mmol, 33%). ^{1}H NMR (400 MHz, $CDCl_3$) δ 7.94 (dd, *J* = 8.1, 1.0 Hz, 1H, ArH), 7.86 (ddd, *J* = 7.8, 3.8, 1.5 Hz, 2H, ArH), 7.71 (td, *J* = 7.7, 1.5 Hz, 1H, ArH), 7.58 (dd, *J* = 7.6, 1.5 Hz, 1H, ArH), 7.48 – 7.30 (m, 8H, ArH), 7.23 (d, *J* = 8.5 Hz, 2H, H13), 6.96 (s, 1H, NH), 6.78 (d, *J* = 8.4 Hz, 2H, H14), 3.95 (s, 1H, OH), 3.73 (s, 2H, H11), 2.99 (d, *J* = 13.6 Hz, 1H, H17), 2.92 (d, *J* = 13.6 Hz, 1H, H17). ^{13}C NMR (101 MHz, $CDCl_3$) δ 202.7, 169.0, 140.1, 137.7, 136.7, 135.2, 134.5, 131.0, 130.9, 129.7, 129.5, 129.34, 129.2, 128.7, 128.7, 128.4, 127.9, 127.5, 126.4, 124.2, 123.42, 119.1, 50.6, 45.1. HRMS (m/z): $[M + H]^+$ m/z calcd. for $C_{29}H_{23}NO_3$ 434.1756, found. 434.1757.

2-phenyl-acetyl-*para*-aminobenzyl β-lapa-ketol 22

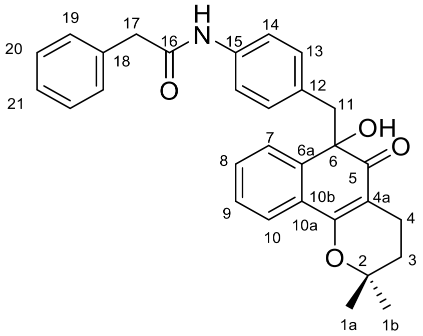

Boc-*para*-aminobenzyl β-lapa-ketol **9** (25 mg, 0.050 mmol) was dissolved in dichloromethane/trifluoroacetic acid 4:1 (2 mL) at 0 ºC. The deprotection was followed by thin-layer chromatography until completion (30 min to 1 h). The residue was dried to offer *para*-aminobenzyl β-lapa-ketol **10**, which was subsequently dissolved in anhydrous dichloromethane (5 mL). Phenyl acetic acid (38 mg, 0.279 mmol) was added followed by HATU (105 mg, 0.276 mmol) and triethylamine (1 mL) to achieve a reaction pH of 10. The reaction was stirred for 30 min. Following this, water was added (30 mL) and the product was extracted into ethyl acetate (3 × 10 mL), dried, and solvents were removed *in vacuo*. The crude product was purified by flash column chromatography (dichloromethane/methanol 9:1) to obtain 2-phenyl-acetyl-*para*-aminobenzyl β-lapa-ketol **22** (4.1 mg, 0.009 mmol, 18%). ^{1}H NMR (500 MHz, MeOD) δ 7.74 (dd, *J* = 7.8, 1.3 Hz, 1H, H10), 7.59 (dd, *J* = 7.8, 1.3 Hz, 1H, H7), 7.52 (td, *J* = 7.6, 1.4 Hz, 1H, H8), 7.37 (td, *J* = 7.6, 1.3 Hz, 1H, H9), 7.32 – 7.30 (m, 4H, ArH), 7.27 – 7.22 (m, 1H, ArH), 7.20 (d, *J* = 8.5 Hz, 2H, H14), 6.36 (d, *J* = 8.5 Hz, 2H, H13), 4.59 (s, 1H, OH), 3.60 (s, 2H, H17), 3.13 (d, *J* = 12.0 Hz, 1H, H11), 3.02 (d, *J* = 12.0 Hz, 1H, H11), 2.40 (dt, *J* = 17.2, 5.6 Hz, 1H, H4), 2.06 – 1.96 (m, 1H, H4), 1.60 – 1.55 (m, 2H, H3), 1.30 (s, 3H, H1a/H1b), 0.85 (s, 3H, H1a/H1b). ^{13}C NMR (126 MHz, MeOD) δ 202.1, 172.0, 163.5, 143.7, 138.9, 136.9, 131.9, 131.2, 130.6, 130.0, 129.6, 129.6, 128.7, 127.9, 127.3, 123.7, 120.0, 109.3, 79.1, 78.9, 53.9, 44.8, 32.4, 27.8, 25.7, 16.6. HRMS (m/z): $[M + H]^{+}$ m/z calcd. for $C_{30}H_{29}NO_4$ 468.2175, found. 468.2178.

2-phenyl-acetyl-*para*-aminobenzyl dunni-ketol 23

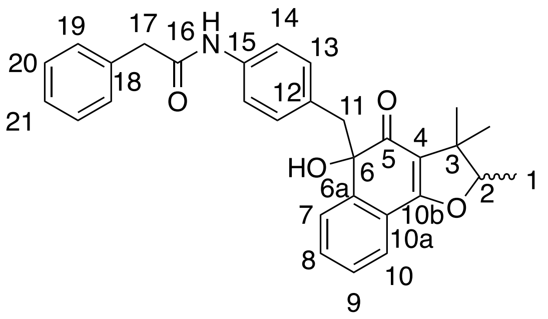

Boc-*para*-aminobenzyl dunni-ketol **15** (40 mg, 0.09 mmol) was dissolved in dichloromethane/trifluoroacetic acid 5:1 (1 mL) at r.t. The reaction was followed by thin-layer chromatography until completion (1 hour). The residue was dried to afford *para*-aminobenzyl dunni-ketol **18** which was subsequently dissolved in anhydrous dichloromethane (2.00 mL). Phenyl acetic chloride (13.20 mL, 0.10 mmol) and triethylamine (34 mL, 0.24 mmol) were added at 0 ºC. The reaction was stirred at room temperature overnight and then washed with 10 mL of 1M aqueous HCl. The organic layer was dried over $MgSO_4$, filtered, and the solvent was removed *in vacuo*. The crude was purified by flash chromatography (Petroleum ether/ Ethyl acetate 4:1→2:1) to obtain 2-phenyl-acetyl-*para*-aminobenzyl dunni-ketol **23** (23.40 mg, 0.05 mmol, 56%) as a yellow foam. In order to facilitate the kinetics studies, a portion of the mixture was purified by HPLC to provide the major isomer (racemic mixture of *S,S* and *R,R*) as white solid. HPLC purification conditions: YMC-triart C18 5mm 10 x 250 mm; solvents A = H_2O B = Acetonitrile; flow rate = 3 mL/min; gradient t = 0.0-5.0 min B=50-100%, t = 5.0-10.0 min B = 100%, t = 10.0-11.0 min B = 50%, t = 10.0-13.3 min B = 50%. Product **23**: t = 6.36 min. ^{1}H NMR (500 MHz, $CDCl_3$) δ 7.55 (dd, *J* = 7.8, 1.4 Hz, 1H, H10), 7.52 (dd, *J* = 7.7, 1.4 Hz, 1H, H7), 7.45 (td, *J* = 7.6, 1.4 Hz, 1H, H8), 7.40 (t, *J* = 7.2 Hz, 2H, Ar*H*), 7.36 – 7.30 (m, 4H, Ar*H*), 7.16 (d, *J* = 8.5 Hz, 2H, H14), 6.96 (s, 1H, N*H*), 6.65 (d, *J* = 8.5 Hz, 2H, H13), 4.36 (q, *J* = 6.7 Hz, 1H, H2), 3.72 (s, 2H, H17), 3.02 (d, *J* = 13.0 Hz, 1H, H11), 2.97 (d, *J* = 12.9 Hz, 1H, H11), 1.38 (d, *J* = 6.7 Hz, 3H, H1), 1.34 (s, 3H, C*H*$_3$), 1.09 (s, 3H,C*H*$_3$). ^{13}C NMR (126 MHz, $CDCl_3$) δ 197.5 (C5), 169.01 (C16 or C10b), 168.99 (C16 or C10b), 144.9 (C6a), 136.5 (CAr), 134.5 (CAr), 131.0 (CHAr), 130.8 (CHAr), 129.7 (CHAr), 129.4 (CHAr), 129.2 (CHAr), 128.4 (CHAr), 127.8 (CHAr), 127.6 (CHAr), 126.5 (CHAr), 125.4 (CAr), 123.6 (CHAr), 123.3 (CAr), 118.9 (CHAr), 117.9 (C4), 91.9 (C2), 79.7 (C6), 53.0 (C11), 45.0 (C17), 44.0 (C3), 24.2 (*C*H$_3$), 20.7 (*C*H$_3$), 13.7 (C1). HRMS (m/z): [M+H]+ calcd. for $C_{30}H_{30}NO_4$ 468.2169, found, 468.2180.

Synthesis of dipeptide prodrugs

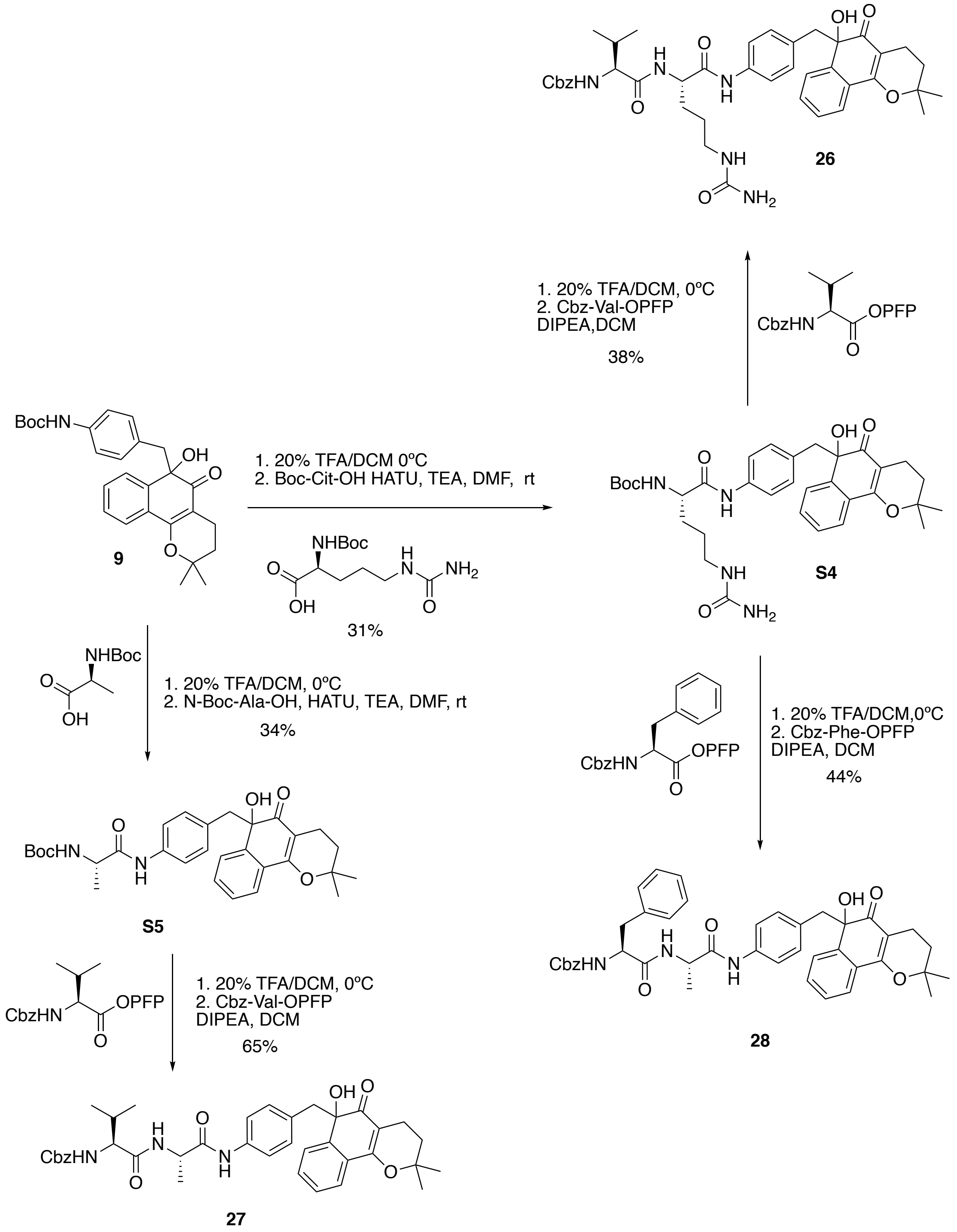

Scheme 7. Synthesis of dipeptide prodrugs **26–28** containing β-lapachone protected as a benzyl ketol.

Boc-L-Cit-OH

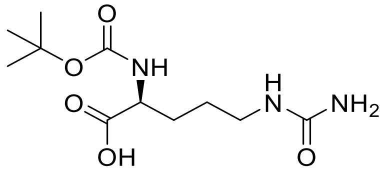

L-citrulline (1.00 g, 5.71 mmol) was dissolved in a solution of 2:1 acetonitrile/water (27 mL). Sodium hydrogen carbonate (720 mg, 18.9 mmol) and di-*tert*-butyl dicarbonate (1.87 g, 8.14 mmol) were added. The reaction was stirred for 48 h. The reaction was then washed with ether (1 × 10 mL) to remove excess di-*tert*-butyl dicarbonate then acidified to pH 3-4 with 1 M citric acid. The product was extracted with a chloroform-isopropanol / water system to yield the product Boc-L-Citrulline (426 mg, 1.55 mmol, 27%). ^{1}H NMR (400 MHz, DMSO-d_6): 1.38 (s, 11H), 1.51 (m, 1H), 1.64 (m, 1H), 2.92 (d, J = 6.3 Hz, 2H), 3.81 (m, 1H), 5.37 (s, 2H), 5.96(s, 1H), 6.98 (d, J = 7.9 Hz, 1H). ^{13}C NMR (100 MHz, DMSO-d_6): δ 26.8, 28.2, 28.4, 39.7, 53.4, 77.9, 155.5, 158.7, 174.2. HRMS (m/z): [M + Na]$^+$ calcd. for $C_{11}H_{21}N_3O_5$ 298.1379 found 298.1372.

Perfluorophenyl ((benzyloxy)carbonyl)-L-valinate

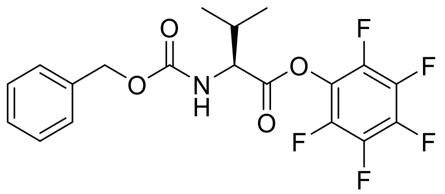

Cbz-L-valine (700 mg, 2.79 mmol) was dissolved in anhydrous dichloromethane (10 mL). The solution was cooled to 0 °C. *N*,*N*-diisopropylethylamine (1.40 mL, 8.04 mmol) was added, followed by pentafluorophenyl trifluoroacetate (575 µL, 3.35 mmol) and the solution was stirred for 1 h at room temperature. Following this, solvent was removed *in vacuo* and the crude product purified by flash column chromatography (dichloromethane/methanol 9:1) to yield perfluorophenyl ((benzyloxy)carbonyl)-L-valinate (1.206 g, 2.60 mmol, 93%). ^{1}H NMR (400 MHz, $CDCl_3$) δ 7.42 – 7.27 (m, 5H), 5.61 (d, J = 8.9 Hz, 1H), 5.17 (s, 1H), 4.71 (dd, J = 8.9, 4.7 Hz, 1H), 2.40 (dq, J = 13.1, 6.6 Hz, 1H), 1.11 (d, J = 6.8 Hz, 3H), 1.05 (d, J = 6.9 Hz, 2H). ^{13}C NMR (100 MHz, $CDCl_3$) δ 168.5, 156.4, 136.0, 128.6, 128.3, 128.2, 67.5, 59.2, 31.2, 18.8, 17.2. HRMS (m/z): [M + H]$^+$ calcd. for $C_{19}H_{16}NO_4F_5$ 418.1078 found 418.1068.

Perfluorophenyl ((benzyloxy)carbonyl)-L-phenylalaninate

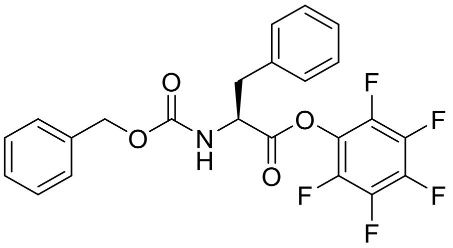

Cbz-L-phenylalanine (700 mg, 2.34 mmol) was dissolved in anhydrous dichloromethane (10 mL). *N*,*N*-diisopropylethylamine (1.22 mL, 7.00 mmol) was added, followed by pentafluorophenyl trifluoroacetate (482 μL, 2.81 mmol) and the solution was stirred for 1 h at room temperature. Following this, solvent was removed *in vacuo* and the crude product purified by flash column chromatography (dichloromethane/methanol 9:1) to yield perfluorophenyl ((benzyloxy)carbonyl)-L-phenylalaninate (928 mg, 2.00 mmol, 85 %). ^{1}H NMR (400 MHz, $CDCl_3$) δ 7.33 (td, *J* = 9.8, 9.2, 6.0 Hz, 8H), 7.22 (d, *J* = 6.5 Hz, 2H), 5.27 (d, *J* = 8.2 Hz, 1H), 5.13 (s, 2H), 5.03 (d, *J* = 7.8 Hz, 1H), 3.43 – 3.20 (m, 2H,). ^{13}C NMR (100 MHz, $CDCl_3$) δ 168.2, 155.7, 136.0, 134.7, 129.4, 129.0, 128.7, 128.5, 128.3, 127.7, 67.5, 54.8, 37.9. HRMS (m/z): $[M + H]^+$ calcd. for $C_{23}H_{16}NO_4F_5$ 466.1078 found 466.1075.

Perfluorophenyl (((9H-fluoren-9-yl)methoxy)carbonyl)-L-valinate

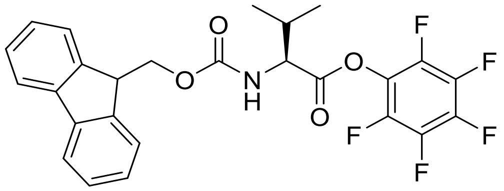

Fmoc-L-valine (600 mg, 1.77 mmol) was dissolved in anhydrous dichloromethane (20 mL). The solution was cooled to 0 °C. *N*,*N*-diisopropylethylamine (920 μL, 5.28 mmol) was added, followed by pentafluorophenyl trifluoroacetate (364 μL, 2.12 mmol) and the solution was stirred for 1 h at room temperature. Following this, solvent was removed *in vacuo* and the crude product purified by flash column chromatography (dichloromethane/methanol 9:1) to yield perfluorophenyl (((9H-fluoren-9-yl)methoxy)carbonyl)-L-valinate (853 mg, 1.69 mmol, 95%). ^{1}H NMR (600 MHz, $CDCl_3$) δ 7.77 (d, *J* = 7.5 Hz, 2H), 7.61 (dd, *J* = 7.6, 3.5 Hz, 2H), 7.41 (t, *J* = 7.5 Hz, 2H), 7.32 (t, *J* = 7.6 Hz, 2H), 5.29 (d, *J* = 9.4 Hz, 1H), 4.69 (dd, *J* = 9.2, 4.8 Hz, 1H), 4.48 (d, *J* = 7.0 Hz, 2H), 4.26 (t, *J* = 6.9 Hz, 1H), 1.10 (d, *J* = 6.8 Hz, 3H), 1.05 (d, *J* = 6.9 Hz, 3H). ^{13}C NMR (151 MHz, $CDCl_3$) δ 141.5, 127.9, 127.2, 125.1, 120.2, 120.2, 67.4, 59.1, 47.3, 31.4, 17.5. $[M + H]^+$ calcd. for $C_{26}H_{20}NO_4F_5$ 506.1385 found 506.1387.

Perfluorophenyl (*E*)-4-oxo-4-phenylbut-2-enoate

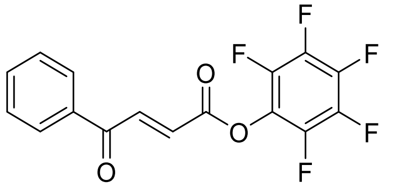

(*E*)-4-Oxo-4-phenylbut-2-enoic acid (300 mg, 1.70 mmol) was dissolved in anhydrous dichloromethane (20 mL). *N*,*N*-diisopropylethylamine (900 µL, 5.1 mmol) was added, followed by pentafluorophenyl trifluoroacetate (357 µL, 2.00 mmol) and the solution was stirred for 2 h at room temperature. Solvent was removed *in vacuo* and the crude product purified by flash column chromatography (dichloromethane/methanol 9:1) to yield perfluorophenyl (*E*)-4-oxo-4-phenylbut-2-enoate as a yellow solid (460 mg, 1.30 mmol, 76%). ^{1}H NMR (600 MHz, $CDCl_3$): δ 8.17 (d, *J* = 15.6 Hz, 1H), 8.04 (d, *J* = 7.2 Hz, 2H), 7.67 (t, *J* = 7.4 Hz, 1H), 7.56 (t, *J* = 7.8 Hz, 2H), 7.11 (d, *J* = 15.6 Hz, 1H). ^{13}C NMR (151 MHz, $CDCl_3$) δ 150.9, 102.7, 98.6, 96.9, 91.6, 91.5, 91.1. HRMS (m/z): $[M + H]^+$ calcd. for $C_{16}H_7O_3F_5$ 343.0394, found 343.0380.

Boc-L-Cit-*para*-aminobenzyl β-lapa-ketol (S4)

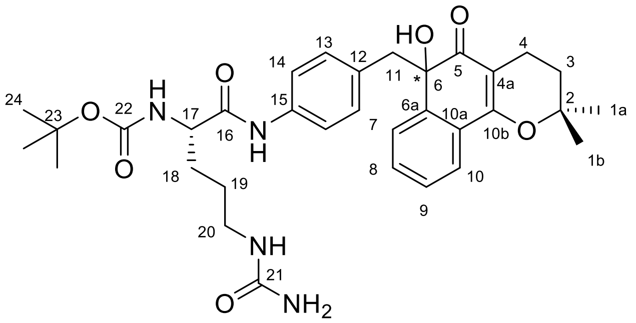

Boc-*para*-aminobenzyl β-lapa-ketol **9** (200 mg, 0.445 mmol) was deprotected by addition of 20 % trifluoroacetic acid in dichloromethane (5 mL) at 0 °C for 30 min. The deprotected residue was dried *in vacuo*. Meanwhile, Boc-L-citrulline (610 mg, 2.22 mmol) and HATU (200 mg, 0.526 mmol) were dissolved in anhydrous dimethylformamide (8 mL) and stirred for 20 min. The crude amine salt was dissolved in anhydrous dimethylformamide (2 mL) was then added to the mixture, immediately followed by triethylamine (1.2 mL). The pH of the reaction was 9–10 as estimated by indicator paper. The solution was stirred for 30 min. Following this, water was added (30 mL) and the product was extracted into ethyl acetate (3 × 10 mL), dried, and solvents were removed *in vacuo*. The crude product was purified by flash column chromatography (9:1 dichloromethane/methanol) to obtain Boc-L-Cit-*para*-aminobenzyl β-lapa-ketol **S4** (82.7 mg, 0.136 mmol, 31%). ^{1}H NMR (500 MHz, DMSO-d$_6$) δ 9.75 (d, *J* = 6.9 Hz, 1H, NH), 7.61 (dt, *J* = 7.9, 1.4 Hz, 1H, H10), 7.53 – 7.45 (m, 2H, H7, H8), 7.39 – 7.31 (m, 1H, H9), 7.26 – 7.20 (m, 2H, H14), 6.93 (dd, *J* = 8.1, 3.3 Hz, 1H, NH), 6.31 (d, *J* = 8.3 Hz, 2H, H13), 5.77 (s, 1H, NH), 5.39 (d, *J* = 8.4 Hz, 2H, NH$_2$), 4.04 – 3.95 (m, 1H, H17), 3.01 (d, *J* = 12.1 Hz, 1H, H11), 3.00 – 2.88 (m, 2H, H20), 2.86 (d, *J* = 12.2 Hz, 1H, H11), 2.31 (dt, *J* = 17.2, 5.6 Hz, 1H, H4), 2.03 – 1.90 (m, 1H, H4), 1.65 – 1.43 (m, 4H, H3, H18), 1.36 (s, 9H, H24), 1.27 (d, *J* = 3.2 Hz, 5H, H1a/H1b, H19), 0.90 (s, 3H, H1a/H1b). ^{13}C NMR (126 MHz, DMSO-d$_6$) δ 199.8 (C5), 199.8 (C5'), 171.6 (C16), 171.5 (C16'), 160.4 (C10b), 160.4 (C10b'), 159.4 (C21), 159.3 (C21'), 155.9 (C22), 155.9 (C22'), 143.0 (C6a),138.0 (C12), 130.0 (C10a), 130.0 (C7/C8), 129.7 (C13), 128.1 (C15),127.7 (C9), 126.8 (C10), 126.8 (C10'), 122.4 (C7/C8), 118.3 (C14), 108.2 (C4a), 108.2 (C4a), 78.5 (C23), 77.7 (C2/C6), 77.7 (C2'/C6'), 77.6 (C2/C6), 77.6 (C2'/C6'), 55.0 (C17), 55.0 (C17'), 52.8 (C11), 39.1 (C20), 31.4 (C3), 29.7 (C18), 29.7 (C18), 28.7 (C24), 27.7 (C1a/C1b), 27.7 (C1a'/C1b'), 27.2 (C19), 25.4 (C1a/C1b), 25.3 (C1a'/C1b'), 15.8 (C4). HRMS (m/z): [M + H]$^+$ calcd. for $C_{33}H_{42}N_4O_7$ 607.3125, found 607.3123.

Boc-L-Ala-*para*-aminobenzyl β-lapa-ketol (S5)

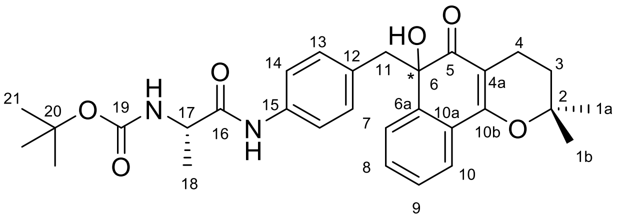

Boc-*para*-aminobenzyl β-lapa-ketol **9** (120 mg, 0.260 mmol) was deprotected by addition of 20% trifluoroacetic acid in dichloromethane (5 mL) at 0 ºC for 30 min. The deprotected residue was dried *in vacuo*. Meanwhile, Boc-L-alanine (100 mg, 0.529 mmol) and HATU (121 mg, 0.318 mmol) were dissolved in anhydrous dimethylformamide (8 mL) and stirred for 20 min. The crude amine-TFA salt was dissolved in anhydrous dimethylformamide (2 mL) then added to the mixture, immediately followed by triethylamine (1.2 mL). The pH of the reaction was 9–10 as estimated by indicator paper. The solution was stirred for 30 min. Following this, water was added (30 mL) and the product was extracted into ethyl acetate (3 x 10 mL), dried, and solvents were removed *in vacuo*. The crude product was purified by flash column chromatography (2:1 40-60 petroleum ether/ethyl acetate) to obtain Boc-L-Ala-*para*-aminobenzyl β-lapa-ketol **S5** (44.5 mg, 0.086 mmol, 34%). ^{1}H NMR (400 MHz, MeOD) δ 7.73 (d, *J* = 7.8 Hz, 1H, H10), 7.60 (dt, *J* = 7.9, 1.6 Hz, 1H, H7), 7.52 (td, *J* = 7.6, 1.4 Hz, 1H, H8), 7.37 (td, *J* = 7.6, 1.3 Hz, 1H, H9), 7.21 (dd, *J* = 8.5, 1.2 Hz, 2H, H14), 6.38 (d, *J* = 8.1 Hz, 2H, H13), 4.13 (d, *J* = 7.0 Hz, 1H, H17), 3.14 (d, *J* = 12.0 Hz, 1H, H11), 3.02 (d, *J* = 12.1 Hz, 1H, H11), 2.81 (s, 1H), 2.42 (dt, *J* = 17.3, 5.6 Hz, 1H, H4), 2.11 – 1.98 (m, 1H, H4), 1.61 (dd, *J* = 7.8, 3.6 Hz, 2H, H3), 1.44 (s, 9H, H21), 1.39 – 1.29 (m, 6H, H18 and H1a/H1b), 0.96 (s, 3H, H1a/H1b). ^{13}C NMR (100 MHz, MeOD) δ 202.1 (C5), 163.5 (C10b), 143.7 (C6a), 138.6 (C16/C19), 131.9 (C16/C19), 131.2 (C9), 130.7 (C13), 129.6 (C10a), 128.7 (C9), 127.3 (C10), 123.7 (C7), 120.1 (C14), 120.1 (C14'), 109.3 (C4a), 109.3 (C4a), 80.6 (C20), 79.1 (C2), 78.9 (C6), 53.9 (C11), 52.1 (C17), 32.5 (C3), 28.7 (C21), 27.8 (C18/C1a/C1b), 27.8 (C18'/C1a'/C1b'), 25.9 (C1a/C1b), 18.4 (C18/C1a/C1b), 16.6 (C4). HRMS (m/z): $[M + H]^+$ calcd. for $C_{30}H_{36}N_2O_6$ 521.2652 found 521.2653.

Cbz-Val-Cit-*para*-aminobenzyl β-lapa-ketol 26

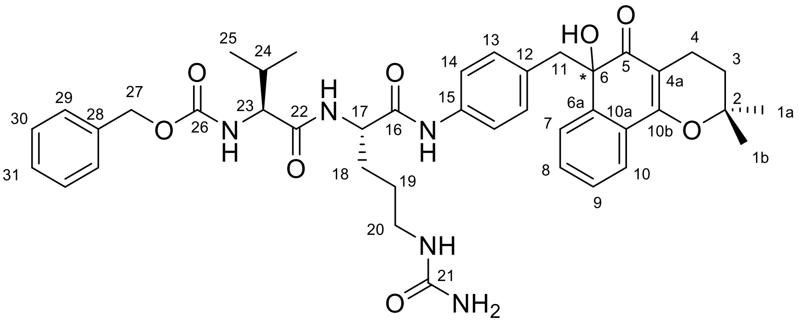

Boc-L-Cit-*para*-aminobenzyl β-lapa-ketol **S4** (30 mg, 0.0495 mmol) was deprotected by addition of 20% trifluoroacetic acid in dichloromethane (2 mL) at 0 ºC. The deprotection was followed by thin layer chromatography and upon completion (30 min) the amine salt was dried *in vacuo*. Perfluorophenyl ((benzyloxy)carbonyl)-L-valinate (48 mg, 0.140 mmol) was dissolved in anhydrous dichloromethane (15 mL) at 0 °C, to which was added *N*,*N*-diisopropylethylamine (100 µL). The deprotected amine dissolved in dichloromethane (5 mL) was added to the reaction dropwise. The pH of the reaction was 9, as measured by universal indicator paper. The temperature of the reaction was slowly raised to room temperature and the reaction was stirred for 6 h. Solvent was removed *in vacuo* to obtain the crude product, which was purified by flash column chromatography (dichloromethane/methanol 9:1). The product was further purified by semi-preparative HPLC to obtain pure Cbz-Val-Cit-*para*-aminobenzyl β-lapa-ketol **26** (13.7 mg, 0.019 mmol, 38%). HPLC purification conditions: column: Agilent Zorbax SB-C18 80Å 5 µm 9.4 x 250 mm; solvents: A = H_2O + 0.1% formic acid, B = acetonitrile + 0.1% formic acid; flow rate = 5 mL/min; gradient t = 0.0 – 1.0 min B = 0%, t = 1.0 – 10.0 min B = 0 – 90%, t = 10.0 – 15.0 min B = 90 – 100%, t = 15.0 – 18.0 min B = 100%, t = 18.0 – 20.0 min B = 0%. Product **26**: t = 11.9 min. ^{1}H NMR (400 MHz, MeOD) δ 7.73 (dt, *J* = 7.7, 1.6 Hz, 1H, ArH), 7.64 – 7.55 (m, 1H, ArH), 7.51 (ddd, *J* = 8.4, 7.4, 1.3 Hz, 1H, ArH), 7.33 (tdd, *J* = 10.1, 6.3, 2.9 Hz, 6H, ArH), 7.22 (d, *J* = 8.1 Hz, 2H, H14), 6.37 (dd, *J* = 8.4, 1.5 Hz, 2H, H13), 5.08 (s, 2H, H27), 4.44 (t, *J* = 7.1 Hz, 1H, H17), 3.94 (d, *J* = 6.8 Hz, 1H, H23), 3.13 (d, *J* = 12.0 Hz, 1H, H11), 3.07 (m, 2H, H20), 3.01 (d, *J* = 12.1 Hz, 1H, H11, H11), 2.46 – 2.35 (m, 1H, H4), 2.04 (hept, *J* = 7.3 Hz, 2H, H4, H24), 1.86 – 1.76 (m, 1H, H18), 1.73 – 1.65 (m, 1H, H18), 1.63 – 1.49 (m, 4H, H3, H19), 1.31 (d, *J* = 1.5 Hz, 3H, H1a/H1b), 0.99 – 0.90 (m, 9H, H1a/H1b and H25). ^{13}C NMR (101 MHz, MeOD) δ 202.1, 174.3, 171.9, 163.5, 162.3, 162.3, 158.8, 143.7, 138.5, 138.2 133.6, 132.4, 132.0, 132.0, 131.2, 130.7, 129.9, 129.6, 129.5, 129.0, 128.9, 128.7, 127.3, 123.7, 120.2, 120.1, 109.4, 109.3, 79.2, 78.9, 69.1, 67.8, 62.3, 62.3, 54.9, 53.8, 40.2, 32.5, 31.9, 31.6, 30.5, 30.1, 27.8, 25.9, 25.8, 25.0,

24.0, 19.8, 18.7, 16.6, 14.4, 11.4. HRMS (m/z): $[M + H]^+$ calcd. for $C_{41}H_{49}N_5O_8$ 740.3659 found 740.3687.

Cbz-Val-Ala-*para*-aminobenzyl β-lapa ketol 27

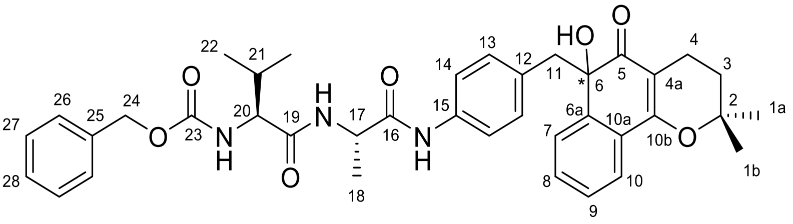

Boc-L-Ala-*para*-aminobenzyl β-lapa-ketol **S5** (10.5 mg, 0.020 mmol) was deprotected by addition of 20% trifluoroacetic acid in dichloromethane (2 mL) at 0 °C. The deprotection was followed by thin layer chromatography and upon completion (30 min) the amine salt was dried *in vacuo*. Perfluorophenyl ((benzyloxy)carbonyl)-L-valinate (25 mg, 0.060 mmol) was dissolved in anhydrous dichloromethane (15 mL) at 0 °C, to which was added *N*,*N*-diisopropylethylamine (100 µL). The deprotected amine dissolved in dichloromethane (5 mL) was added to the reaction dropwise. The pH of the reaction was 9, as measured by universal indicator paper. The temperature of the reaction was slowly raised to room temperature and the reaction was stirred for 6 h. Solvent was removed *in vacuo* to obtain the crude product, which was purified by flash column chromatography (dichloromethane/methanol 9:1). The product was further purified by semi-preparative HPLC to obtain pure Cbz-Val-Ala-*para*-aminobenzyl β-lapa-ketol **27** (8.8 mg, 0.013 mmol, 65%). HPLC purification conditions: column: Agilent Zorbax SB-C18 80Å 5 µm 9.4 x 250 mm; solvents: A = H_2O + 0.1% formic acid, B = acetonitrile + 0.1 % formic acid; flow rate = 5 mL/min; gradient t = 0.0 – 1.0 min B = 0%, t = 1.0–10.0 min B = 0–80%, t = 10.0 – 25.0 min B = 80 – 95%, t = 25.0 – 25.1 min B = 95 – 100%, t = 25.1 – 28.0 B = 100%, t = 28.0 – 30.0 min B = 0%. Product **27**: t = 14.5 min. ^{1}H NMR (600 MHz, MeOD) δ 7.73 (ddd, *J* = 7.8, 2.6, 1.3 Hz, 1H, H10), 7.59 (ddd, *J* = 9.9, 7.9, 1.3 Hz, 1H, H7), 7.52 (td, *J* = 7.6, 1.4 Hz, 1H, H8), 7.39 – 7.26 (m, 6H, H9), 7.23 (d, *J* = 8.1 Hz, 2H, H14), 6.38 (dd, *J* = 8.6, 1.8 Hz, 2H, H13), 5.09 (s, 2H, H24), 4.59 (s, 1H, NH), 4.42 (q, *J* = 7.2 Hz, 1H, H17), 3.94 (dd, *J* = 6.7, 3.9 Hz, 1H, H20), 3.14 (dd, *J* = 12.2, 1.4 Hz, 1H, H11), 3.02 (dd, *J* = 12.2, 1.3 Hz, 1H, H11), 2.41 (dt, *J* = 17.2, 5.6 Hz, 1H, H4), 2.12 – 1.99 (m, 2H, H4 and H21), 1.64 – 1.52 (m, 2H, H3), 1.38 (d, *J* = 7.2 Hz, 3H, H18), 1.32 (s, 3H, H1a/H1b), 1.01 – 0.91 (m, 9H, H1a/H1b and H22). ^{13}C NMR (126 MHz, MeOD) δ 202.1, 174.0, 172.7, 163.6, 163.6, 158.8, 143.7, 138.5, 138.5, 138.2, 132.0, 131.9, 131.2, 130.7, 129.6, 129.5, 129.5, 129.0, 128.9, 128.7, 128.7, 127.3, 127.3, 123.7, 123.7, 120.2, 120.1, 109.4, 109.3, 79.2, 79.2, 78.9, 67.8, 62.2, 62.2, 53.9, 53.8, 51.0, 32.5, 31.9, 27.8, 25.9, 25.8, 19.7, 19.7, 18.5, 18.1, 18.1, 16.6. HRMS (m/z): $[M + H]^+$ calcd. for $C_{38}H_{43}N_3O_7$ 654.3179 found 654.3198.

Cbz-Phe-Cit-*para*-aminobenzyl β-lapa-ketol 28

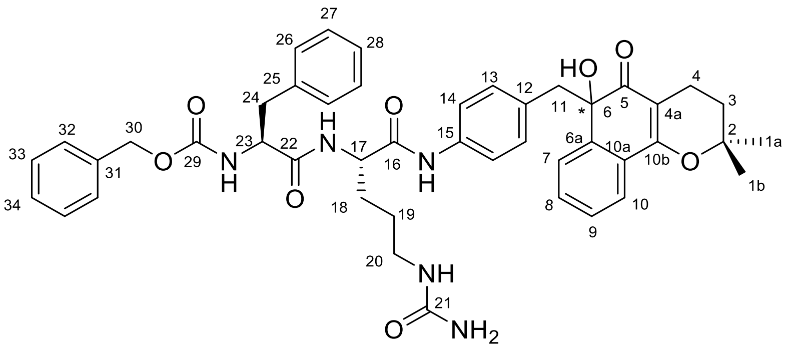

Boc-L-Cit-*para*-aminobenzyl β-lapa-ketol **S4** (15 mg, 0.025 mmol) was deprotected by addition of 20 % trifluoroacetic acid in dichloromethane (2 mL) at 0 °C. The deprotection was followed by thin layer chromatography and upon completion (30 min) the amine salt was dried *in vacuo*. perfluorophenyl ((benzyloxy)carbonyl)-L-phenylalaninate (23 mg, 0.049 mmol) was dissolved in anhydrous dichloromethane (15 mL) at 0 °C, to which was added *N*,*N*-diisopropylethylamine (100 µL). The deprotected amine dissolved in dichloromethane (5 mL) was then added to the reaction dropwise. The pH of the reaction was 9, as measured by universal indicator paper. The temperature of the reaction was slowly raised to room temperature and the reaction was stirred for 6 h. Solvent was removed *in vacuo* to obtain the crude product, which was purified by flash column chromatography (dichloromethane/methanol 9:1). The product was further purified by semi-preparative HPLC to obtain pure Cbz-Phe-Cit-*para*-aminobenzyl β-lapa-ketol **28** (7 mg, 0.011 mmol, 44%). HPLC purification conditions: column: Agilent Zorbax SB-C18 80Å 5 µm 9.4 x 250 mm; solvents: A = H_2O + 0.1% formic acid, B = acetonitrile + 0.1% formic acid; flow rate = 5 mL/min; gradient t = 0 - 1 min B = 0%, t = 1 – 10 min B = 0–90%, t = 10 – 15 min B = 90 – 100%, t = 15.0 – 18.0 min B = 100 t = 18.0 – 20.0 min B = 0%. Product **28**: t = 12.5 min. ^{1}H NMR (400 MHz, MeOD) δ 7.76 – 7.71 (m, 1H, H10), 7.62 – 7.56 (m, 1H, H7), 7.55 – 7.48 (m, 1H, H8), 7.39 – 7.32 (m, 1H, H9), 7.32 – 7.11 (m, 7H, ArH), 6.39 (d, J = 8.4 Hz, 2H, H13), 5.01 (d, J = 2.3 Hz, 2H, H30), 4.59 (s, 2H, NH_2), 4.40 (td, J = 9.0, 4.5 Hz, 2H, H17 and H23), 3.14 (dd, J = 12.0, 2.3 Hz, 1H, H11), 3.11 – 3.05 (m, 3H, H24 and H20), 3.02 (d, J = 11.9 Hz, 1H, H11), 2.91 – 2.82 (m, 1H, H24), 2.41 (dd, J = 17.4, 3.5 Hz, 1H, H4), 2.11 – 1.97 (m, 1H, H4), 1.80 (d, J = 5.2 Hz, 1H, H18), 1.72 – 1.54 (m, 3H, H3 and H18), 1.48 (q, J = 7.1 Hz, 2H, H19), 1.31 (s, 3H, H1a/H1b), 0.94 (d, J = 5.8 Hz, 3H, H1a/H1b). ^{13}C NMR (101 MHz, MeOD) δ 202.2, 174.2, 171.9, 171.9, 163.6, 162.4, 158.4, 143.8, 138.4, 138.2, 132.1, 131.3, 130.7, 130.4, 129.6, 129.5, 129.0, 128.8, 128.8, 128.7, 127.8, 127.4, 123.8, 120.3, 109.4, 79.7, 79.3, 79.0, 78.9, 78.9, 67.7, 58.0, 54.9, 53.9, 40.3, 39.0, 32.5, 30.6, 27.9, 27.7, 25.9, 16.7. HRMS (m/z): $[M + H]^+$ calcd. for $C_{45}H_{49}N_5O_8$ 788.3659 found 788.3694.

Synthesis of ADC linker 30

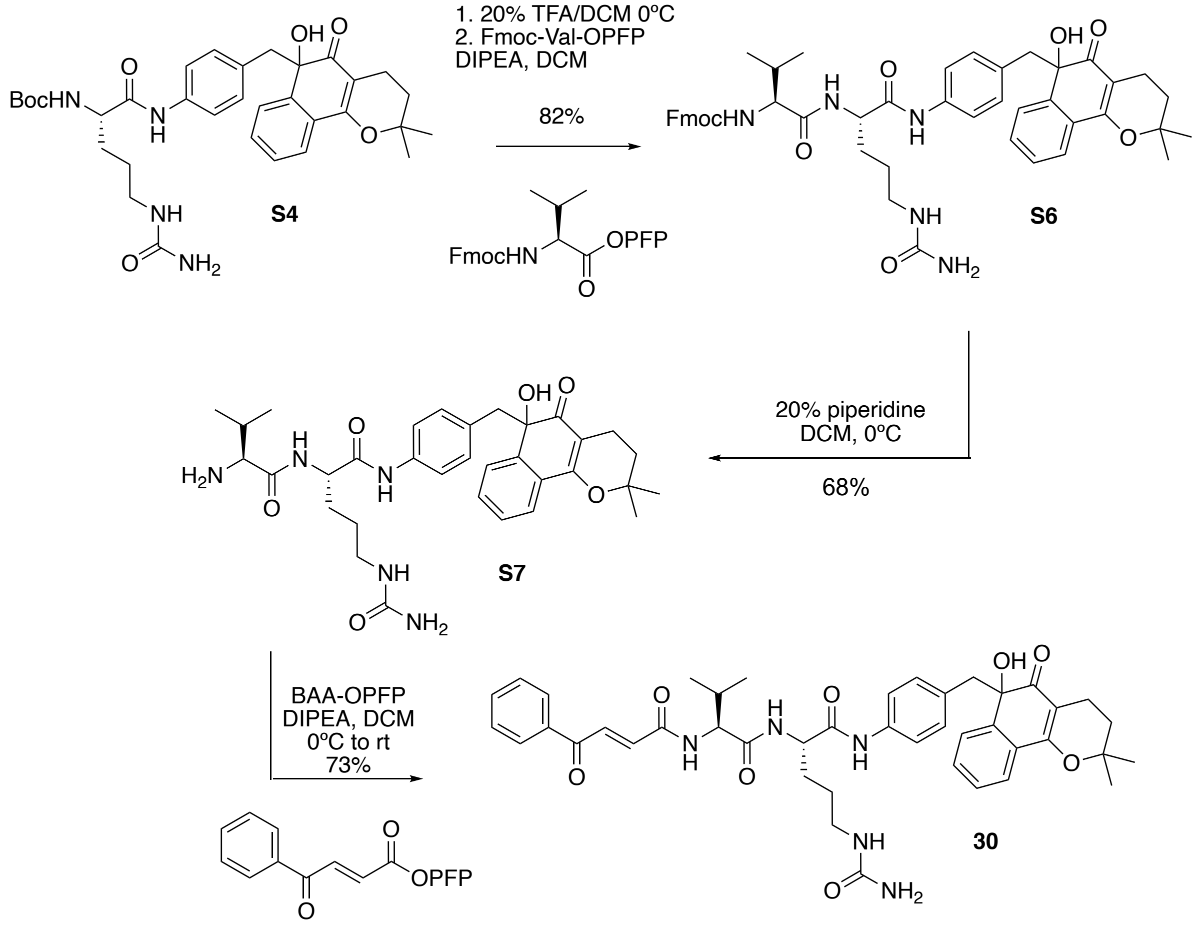

Scheme 8. Synthesis of ADC linker **30**.

Fmoc-Val-Cit-*para*-aminobenzyl β-lapa-ketol S6

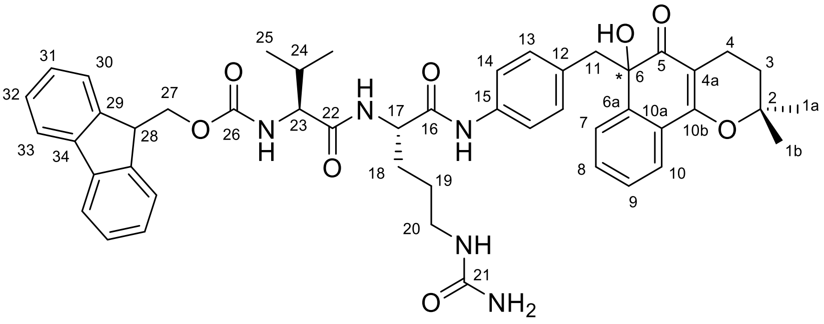

Boc-L-Cit-*para*-aminobenzyl β-lapa-ketol **S4** (75 mg, 0.124 mmol) was deprotected by addition of 20% trifluoroacetic acid in dichloromethane (2 mL) at 0 °C. The deprotection was followed by thin layer chromatography and upon completion (30 min) the amine salt was dried *in vacuo*. Perfluorophenyl (((9H-fluoren-9-yl)methoxy)carbonyl)-L-valinate (125 mg, 0.248 mmol) was dissolved in anhydrous dichloromethane (15 mL) at 0 °C, and to this solution *N*,*N*-diisopropylethylamine (50 μL) was added. The deprotected amine in dichloromethane (5 mL) was then added to the reaction dropwise. Further *N*,*N*-diisopropylethylamine (50 μL) was added to the reaction following amine addition to obtain a reaction pH of 9, as measured by universal indicator paper. The temperature of the reaction was slowly raised to room temperature and the reaction was stirred for 6 h. Solvent was removed *in vacuo* to obtain the crude product, which was purified by flash column chromatography (dichloromethane/methanol 9:1) to obtain Fmoc-Val-Cit-*para*-aminobenzyl β-lapa-ketol **S6** (84 mg, 0.102 mmol, 82 %). ^{1}H NMR (600 MHz, MeOD) δ 7.80 (d, J = 7.6 Hz, 2H, ArH), 7.73 (d, J = 7.5 Hz, 1H, ArH), 7.67 (dd, J = 11.4, 6.5 Hz, 2H, ArH), 7.58 (t, J = 7.3 Hz, 1H, ArH), 7.52 (t, J = 7.6 Hz, 1H, ArH), 7.42 – 7.33 (m, 3H, ArH), 7.31 (td, J = 6.9, 3.2 Hz, 2H, ArH), 7.22 (dd, J = 8.2, 5.9 Hz, 2H, H14), 6.35 (dd, J = 8.5, 2.9 Hz, 2H, H13), 4.45 (dt, J = 9.8, 5.3 Hz, 1H, H17), 4.43 – 4.31 (m, 2H, H27), 4.23 (t, J = 6.7 Hz, 1H, H28), 3.94 (d, J = 7.0 Hz, 1H, H23), 3.22 – 3.10 (m, 2H, H11 & H20), 3.07 (dt, J = 13.7, 6.7 Hz, 1H, H11), 3.04 – 2.96 (m, 1H, H20), 2.39 (dt, J = 17.1, 5.8 Hz, 1H, H4), 2.11 – 1.95 (m, 2H, H4 & H24), 1.83 (s, 1H, H18), 1.69 (d, J = 9.4 Hz, 1H, H18), 1.64 – 1.45 (m, 4H, H3 & H19), 1.30 (d, J = 8.3 Hz, 3H, H1a/H1b), 0.99 – 0.92 (m, 6H, H25), 0.91 (d, J = 6.7 Hz, 3H, H1a/H1b). ^{13}C NMR (126 MHz, MeOD) δ 202.1, 174.3, 171.9, 163.5, 162.3, 158.8, 145.3, 145.2, 143.7, 142.6, 138.4, 132.0, 131.2, 130.6, 129.6, 128.8, 128.7, 128.2, 127.3, 126.2, 123.7, 121.0, 120.1, 109.4, 79.2, 78.9, 68.1, 62.3, 54.9, 53.9, 49.9, 40.2, 32.5, 31.8, 30.5, 27.8, 25.8, 19.8, 18.8, 16.6. HRMS (m/z): [M + H]$^{+}$ calcd. for $C_{48}H_{53}N_5O_8$ 828.3972 found 828.3958.

NH_2-Val-Cit-*para*-aminobenzyl β-lapa-ketol S7

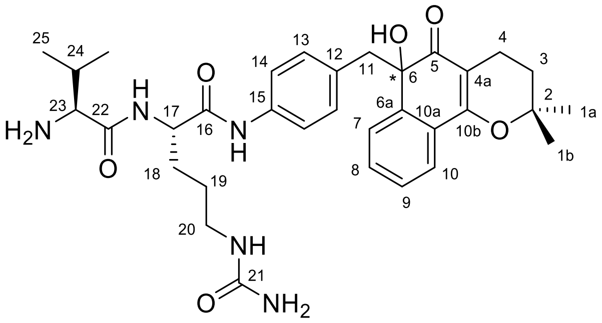

Fmoc-Val-Cit-*para*-aminobenzyl β-lapa-ketol **S6** (18.5 mg, 0.02 mmol) was deprotected with a solution of 20 % piperidine in dichloromethane at 0 ° C for 30 min. Then, the solvent was removed *in vacuo* and the mixture was purified by semi-preparative HPLC to yield intermediate NH_2-Val-Cit-*para*-aminobenzyl β-lapa-ketol (8.2 mg, 0.014 mmol, 68%). HPLC purification conditions: column: YMC-Triart-C18 80Å 5 µm 10.0 x 250 mm; solvents: A = H_2O + 0.1% formic acid, B = acetonitrile + 0.1% formic acid; flow rate = 3 mL/min; gradient t = 0.0 – 5.0 min B = 0%, t = 5.0 – 10.0 min B = 0 – 45%, t = 10.0 – 22.0 min B = 45 – 70%, t = 22.0 – 22.1 min B = 70 – 100%, t = 22.1 – 26.0 B = 100%, t = 26.1 – 30.0 min B = 0%. Product **S7**: t = 13.4 min. ^{1}H NMR (600 MHz, MeOD) δ 8.36 (s, 2H, NH_2), 7.74 (d, *J* = 7.8 Hz, 1H, H10), 7.64 – 7.56 (m, 1H, H7), 7.53 (td, *J* = 7.6, 1.3 Hz, 1H, H8), 7.38 (t, *J* = 7.6 Hz, 1H, H9), 7.25 – 7.14 (m, 2H, H14), 6.40 (dd, *J* = 8.5, 1.7 Hz, 2H, H13), 4.52 (dt, *J* = 9.2, 5.1 Hz, 1H, H17), 3.69 (d, *J* = 5.8 Hz, 1H, H23), 3.21 – 3.07 (m, 3H, H11 and H20), 3.02 (d, *J* = 12.1 Hz, 1H, H11), 2.42 (dt, *J* = 17.2, 5.6 Hz, 1H, H4), 2.24 – 2.17 (m, 1H, H24), 2.10 – 1.99 (m, 1H, H4), 1.89 – 1.86 (m, 1H, H18), 1.77 – 1.66 (m, 1H, H18), 1.65 – 1.51 (m, 4H, H3 and H19), 1.33 (d, *J* = 2.0 Hz, 3H, H1a/H1b), 1.05 (ddd, *J* = 16.6, 6.9, 4.1 Hz, 6H, H25), 0.95 (d, *J* = 2.9 Hz, 3H, H1a/H1b). HRMS (m/z): $[M + H]^+$ calcd. for $C_{33}H_{43}N_5O_6$ 605.3292 found. 606.3303

BAA-Val-Cit-*para*-aminobenzyl β-lapa ketol 30

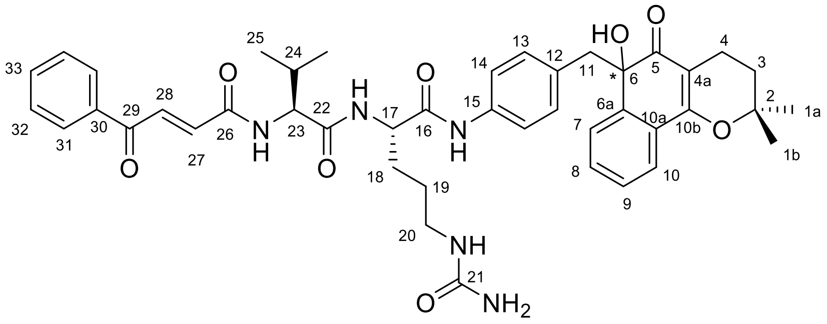

Perfluorophenyl (*E*)-4-oxo-4-phenylbut-2-enoate (2 mg, 0.0058 mmol) was dissolved in anhydrous dichloromethane (2 mL) under a nitrogen atmosphere at 0 °C. *N*,*N*-diisopropylethylamine (300 µL) was added. NH_2-Val-Cit-*para*-aminobenzyl β-lapa-ketol **S7** (2.4 mg, 0.0040 mmol) dissolved in anhydrous dichloromethane (2 mL) was added to the solution dropwise. The reaction was stirred for 2 h at 0 °C and followed by thin-layer chromatography. Solvent was removed *in vacuo* and the crude product was purified by semi-preparative HPLC to yield BAA-Val-Cit-*para*-aminobenzyl β-lapa-ketol **30** (2.2 mg, 0.0028 mmol, 73%). HPLC purification conditions: column: Agilent Zorbax SB-C18 80Å, 5 µm, 9.4 x 250 mm; solvents: A = H_2O, B = acetonitrile; flow rate = 5 mL/min; gradient t = 0.0 – 10.0 min B = 0 – 60%, t = 10.0 – 20.0 min B = 60 – 65%, t = 20.0 – 25.0 min B = 65 – 100%, t = 25.0 – 27.0 min B = 100%, t = 27.0 – 27.1 B = 100 – 0%, t = 27.1 – 30.0 min B = 0%. Product **30**: t = 16.2 min. ^{1}H NMR (500 MHz, MeOD) δ 8.55 (s, 2H, NH_2), 8.06 – 7.98 (m, 2H, ArH), 7.87 (d, *J* = 15.3 Hz, 1H, H28), 7.74 (dd, *J* = 7.8, 1.3 Hz, 1H, ArH), 7.70 – 7.64 (m, 1H, ArH), 7.62 – 7.48 (m, 4H, ArH), 7.37 (tdd, *J* = 7.6, 4.9, 1.3 Hz, 1H, ArH), 7.24 (dq, *J* = 9.1, 2.6 Hz, 2H, H14), 7.17 (d, *J* = 15.3 Hz, 1H, H27), 6.38 (dd, *J* = 8.5, 1.6 Hz, 2H, H13), 4.59 (s, 4H, NH), 4.46 (ddd, *J* = 8.7, 5.3, 3.3 Hz, 1H, H17), 4.31 (d, *J* = 7.3 Hz, 1H, H23), 3.14 (d, *J* = 12.1 Hz, 1H, H11), 3.09 (m, 2H, H20), 3.02 (d, *J* = 12.1 Hz, 1H, H11), 2.47 – 2.37 (m, 1H, H4), 2.20 – 2.09 (m, 1H, H24), 2.04 (dtd, *J* = 16.7, 9.2, 6.8 Hz, 1H, H4), 1.91 – 1.80 (m, 1H, H18), 1.78 – 1.67 (m, 1H, H18), 1.64 – 1.41 (m, 4 H, H3 and H19), 1.32 (s, 3H, H1a/H1b), 1.01 (ddd, *J* = 6.6, 3.5, 2.6 Hz, 6H, H25), 0.94 (d, *J* = 3.0 Hz, 3H, H1a/H1b). ^{13}C NMR (126 MHz, MeOD) δ 200.1, 200.1, 191.6, 173.4, 172.0, 166.9, 163.6, 163.6, 162.3, 143.7, 138.5, 138.5, 138.3, 136.3, 134.9, 134.6, 132.0, 132.0, 131.2, 130.7, 130.1, 129.9, 129.6, 128.7, 127.3, 123.7, 123.7, 120.1, 109.5, 109.5, 79.2, 79.2, 78.9, 68.9, 61.0, 61.0, 55.0, 55.0, 53.9, 53.9, 33.1, 31.8, 30.8, 30.5, 30.4, 27.8, 27.8, 25.9, 25.8, 19.8, 18.9, 18.9, 16.6. HRMS (m/z): $[M + H]^+$ calcd. for $C_{43}H_{49}N_5O_8$ 764.3654, found 764.3641.

3. Elimination from benzyl ketol species: characterisation and kinetic analysis

Proof-of-concept of 1,6-elimination by ^{1}H NMR

Boc-protected benzyl ketols were dissolved in dichloromethane/trifluoroacetic acid 4:1 at 0 °C and stirred for 30 min, upon which the reaction appeared complete by thin-layer chromatography. The product was dried *in vacuo*. ^{1}H NMR spectra in $CDCl_3$ and MeOD solvent were measured immediately and at subsequent 24 h intervals. Acidity remaining from the TFA-deprotection promoted the elimination in each case. Release rate is dependent on compound and the acidity of the environment, as explored in subsequent figures.

PAB-PhQ **7** ($CDCl_3$)

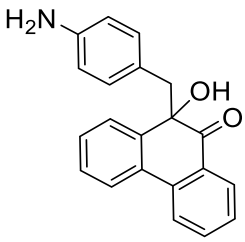

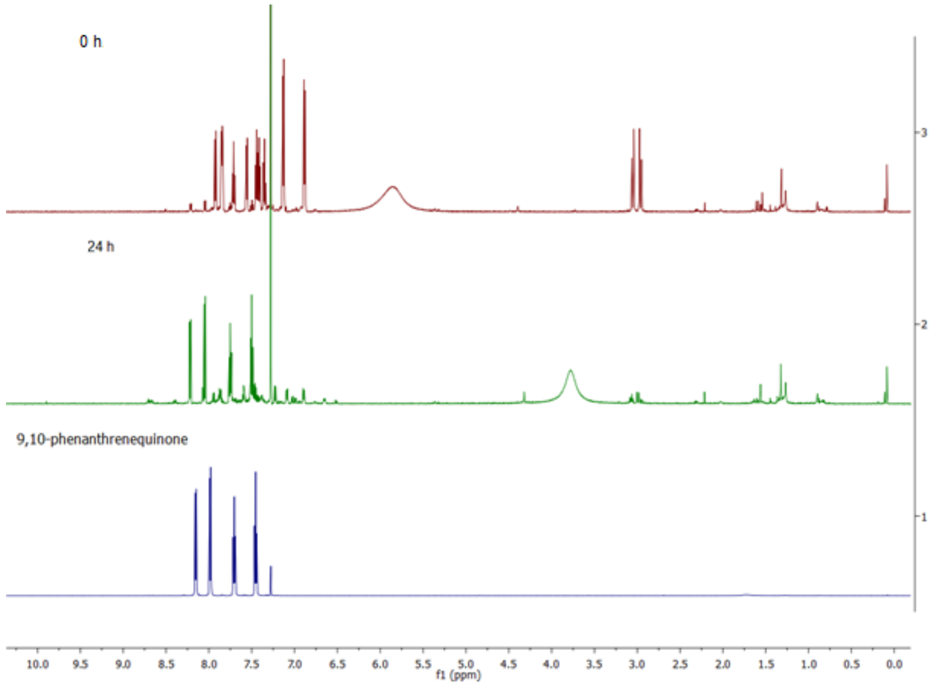

Figure 1. Proof of concept of elimination from PAB-PhQ 7 under acidic conditions by ^{1}H NMR in $CDCl_3$. Emergence of quinone peaks apparent. Note: a substance precipitated out of the solution, correlating with the disappearance of the aniline aromatic peaks. Replicate experiments displayed similar results.

PAB-PhQ **7** (MeOD)

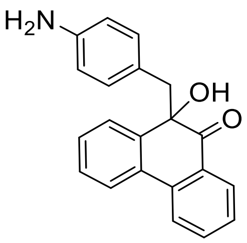

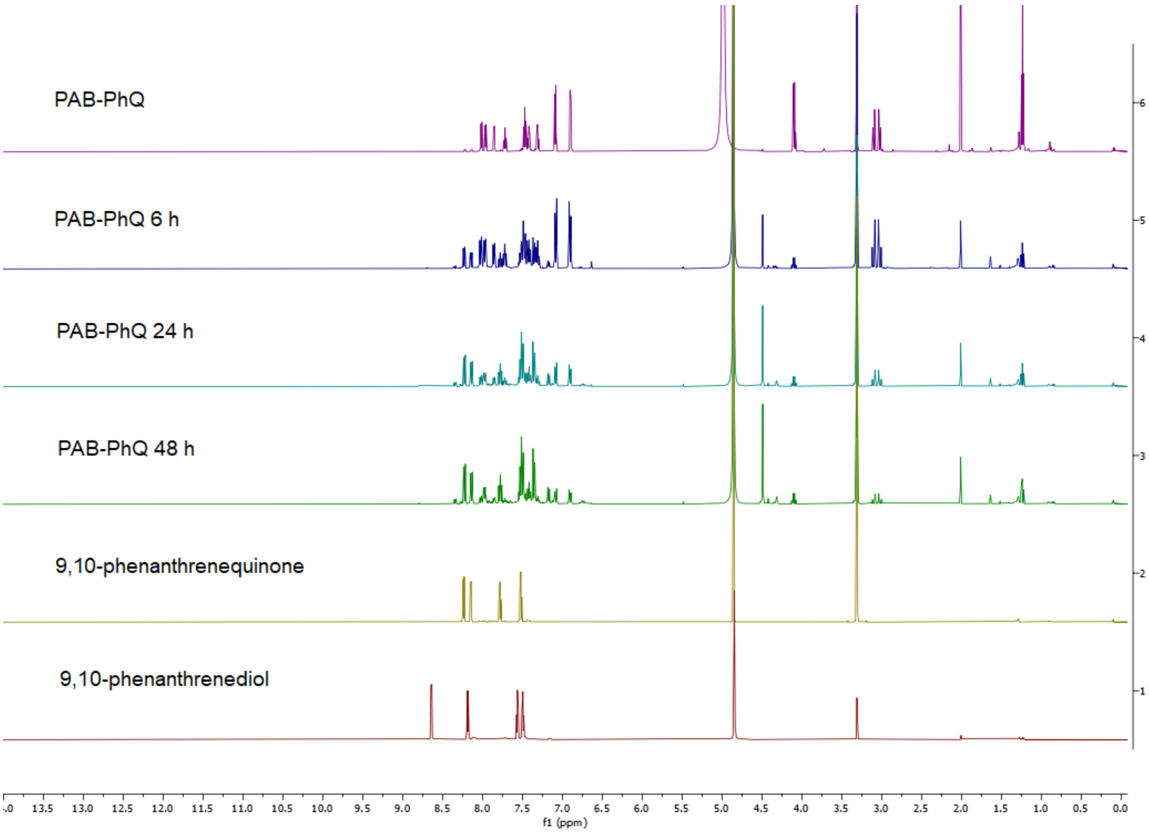

Figure 2. Proof of concept of elimination from PAB-PhQ 7 under acidic conditions by ^{1}H NMR in MeOD. Emergence of quinone peaks apparent. ^{1}H NMR of unstable phenanthrene-9,10-diol **8** recorded immediately after its synthesis in deuterated methanol also compared. These peaks are not observed. Replicate experiments displayed similar results.

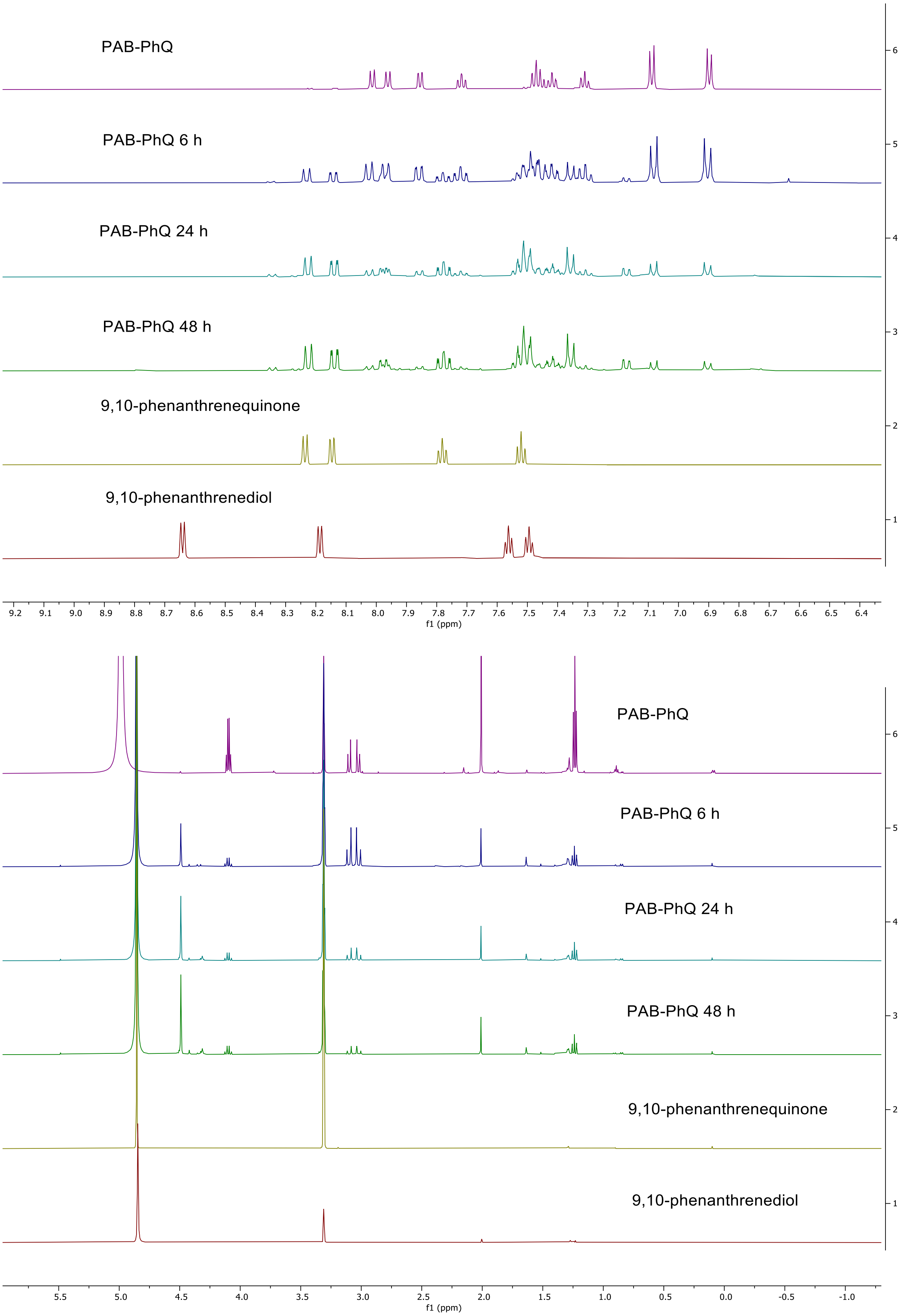

Figure 3. Zoom of spectra in Figure 2.

Kinetics of elimination from PAB-PhQ 7 measured by ^{1}H NMR

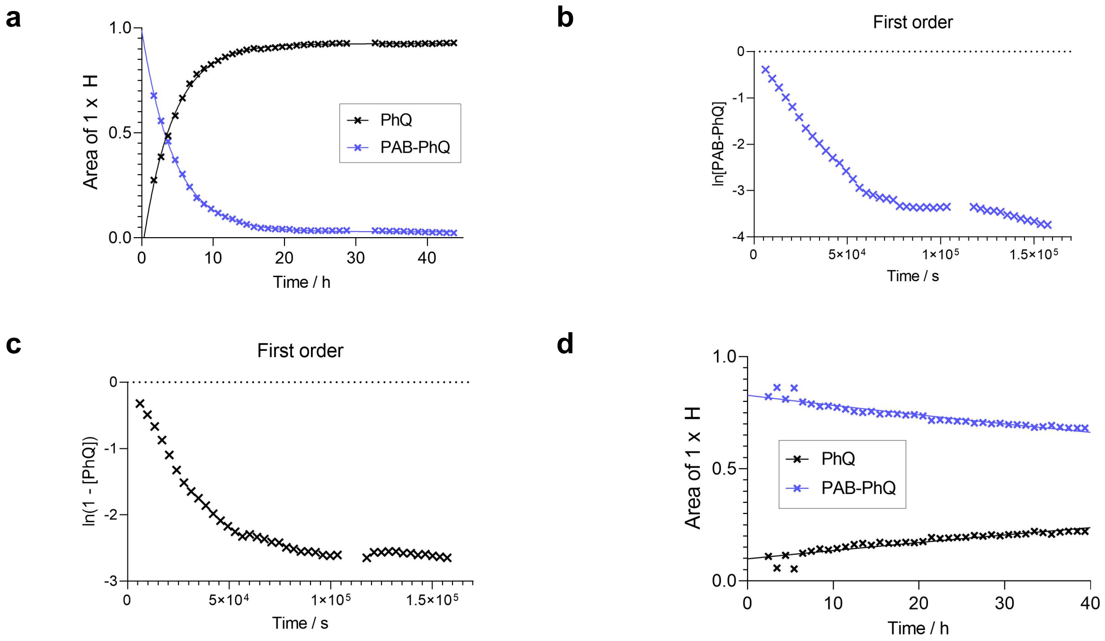

e

	Estimated pH	K_{1st} / s^{-1}	95 % CI	Half-time / h	95 % CI	R^2
**PhQ appearance**	~ 6	6.55×10^{-5}	$6.39 – 6.77 \times 10^{-5}$	2.94	2.84 – 3.03	0.98
	~ 9	1.16×10^{-6}	$9.98 \times 10^{-7} – 1.33 \times 10^{-6}$	165.5	145 – 192	0.85
**PAB-PhQ disappearance**	~ 6	6.18×10^{-5}	$6.07 – 6.30 \times 10^{-5}$	3.11	3.06 – 3.17	0.99
	~ 9	1.55×10^{-6}	$1.37 - 1.72 \times 10^{-6}$	124.5	112 – 140	0.90

Figure 4. Kinetics of elimination from PAB-PhQ 7 measured by ^{1}H NMR at 25 °C at acidic and basic pH. a, Formation of PhQ **4** and consumption of PAB-PhQ **7** at pH ~ 6. **b**, First-order kinetics fit for PAB-PhQ **7** consumption at pH ~ 6. Linearity is observed in the exponential part of the curve suggesting a fit. **c**, First-order kinetics fit for PhQ **4** formation at pH ~ 6. Linearity is observed in the exponential part of the curve suggesting a fit. **d**, Formation of PhQ **4** and consumption of PAB-PhQ **7** at pH ~ 9. **e**, Calculated rate constants and half-lives of PhQ **4** formation and PAB-PhQ **7** consumption at pH ~ 6 and pH ~ 9. This experiment was performed once. Similar experiments are displayed in **Supplementary Figures 1–3** and **6**, demonstrating proof-of concept of the elimination. The gap in data for the acidic pH sample in **a**–**c** is due to a machine fault at these timepoints.

a ~ pH 6

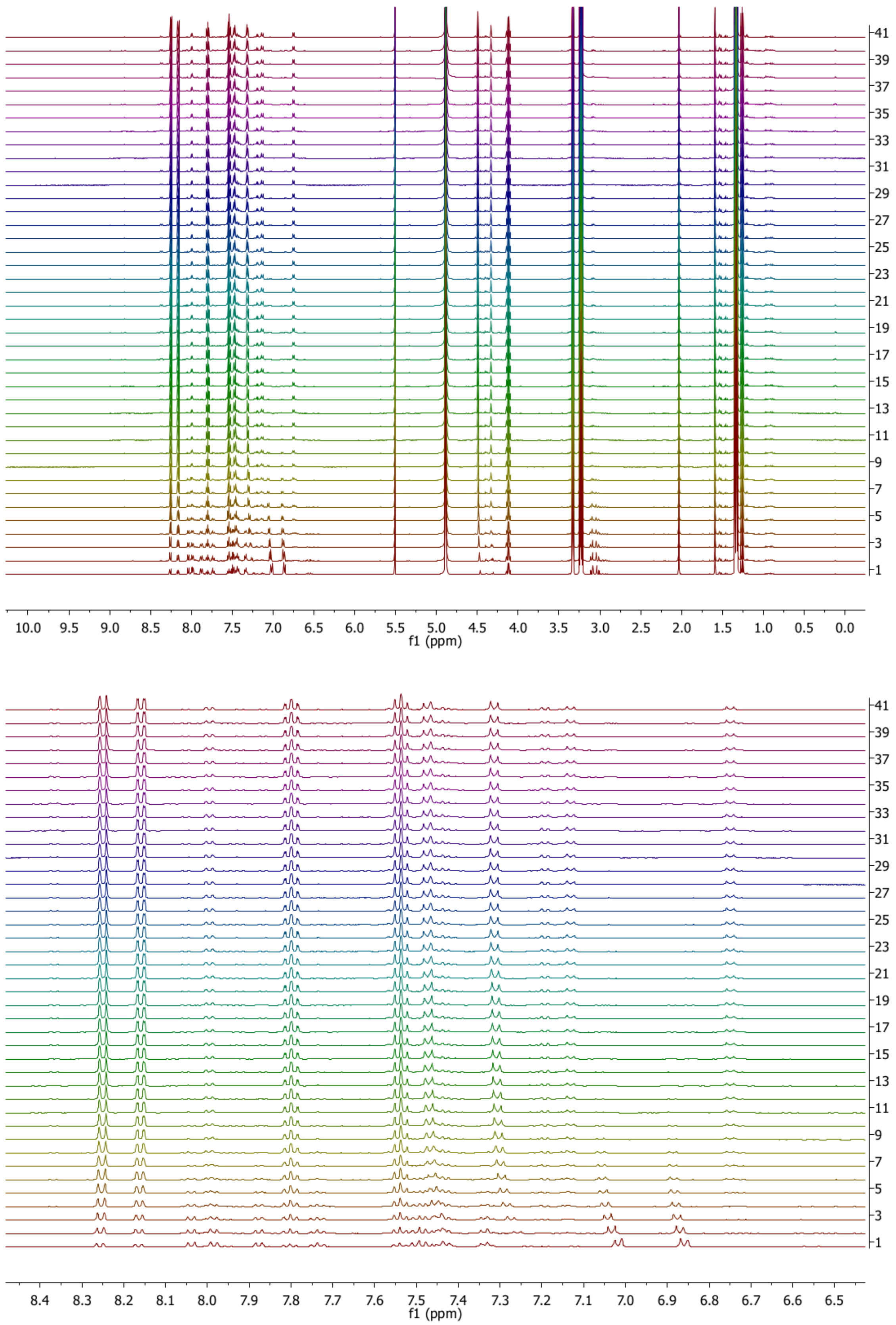

b ~ pH 9

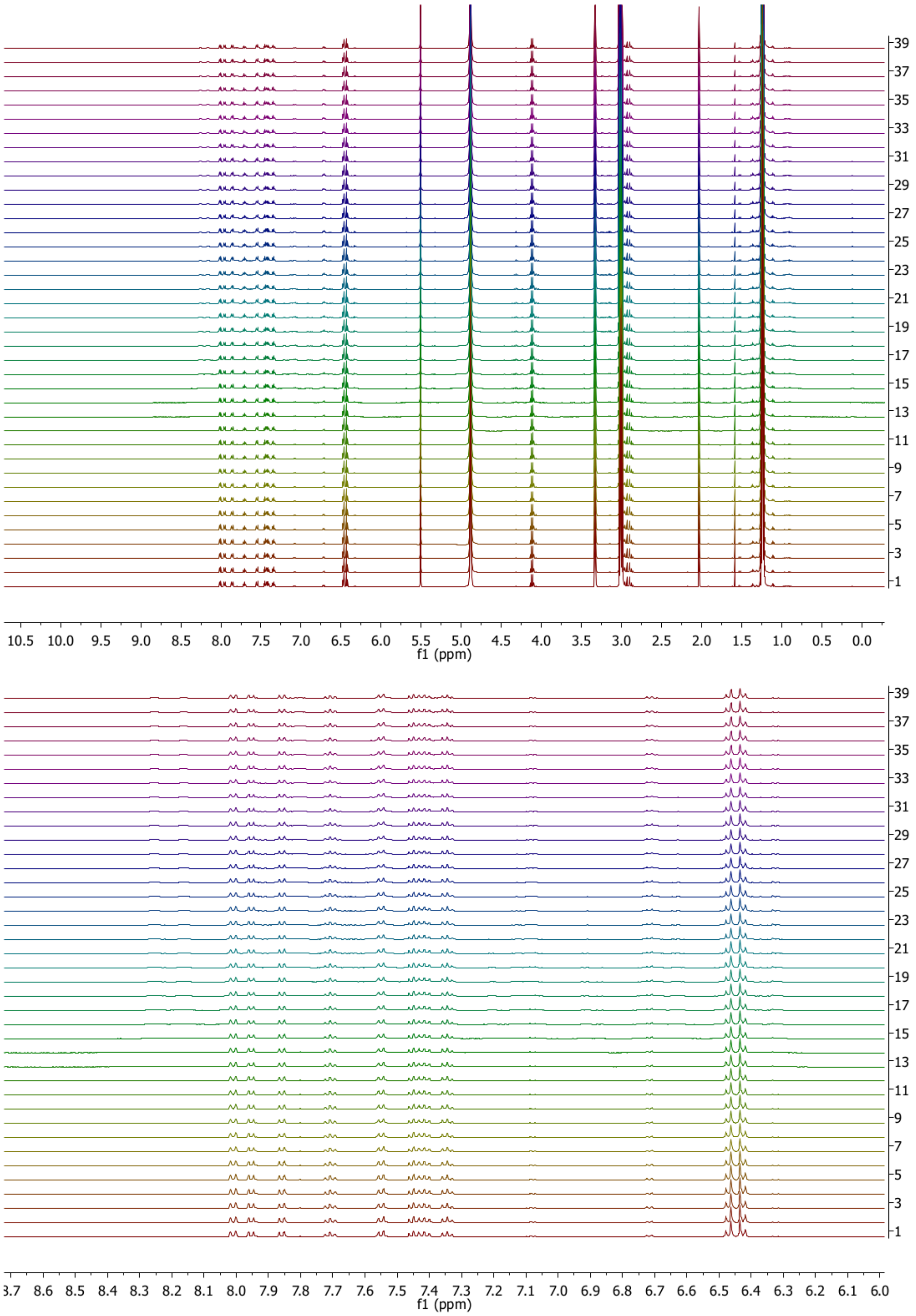

Figure 5. Raw data for kinetics of elimination from PAB-PhQ 7 measured by ^{1}H NMR at 25 ºC. a, pH 6. **b**, pH 9.

Initial rate of elimination from PAB-PhQ 7

Formation of **4** from **7** was monitored by ^{1}H NMR at 25 °C, over 12 h.

Method: Analysis was performed in MeOD due to poor solubility of the compound in H_2O. To form the active fragmenting species, Boc-PAB-PhQ **6** was dissolved in dichloromethane/trifluoroacetic acid 4:1 at 0 °C and stirred for 30 min, upon which the reaction appeared complete by thin-layer chromatography. Product **7** was dried *in vacuo* before being dissolved in MeOD. Basicity of the solution was altered by addition of triethylamine (drops). pH was estimated using pH paper. It should be noted that pH values here are indicative only as analysis is performed in MeOD. Elimination rates were determined from the aromatic region. Elimination was measured by monitoring the integral of one peak, normalised to number of protons present (*i.e.* integral of 1 × H), divided by the total integral of the aromatic region (δ = 6.2 – 8.6 ppm, *i.e.* integral of 12 × H).

a Formation of 9,10 phenanthrenequinone **4** and consumption of PAB-PhQ **7** over 12 h

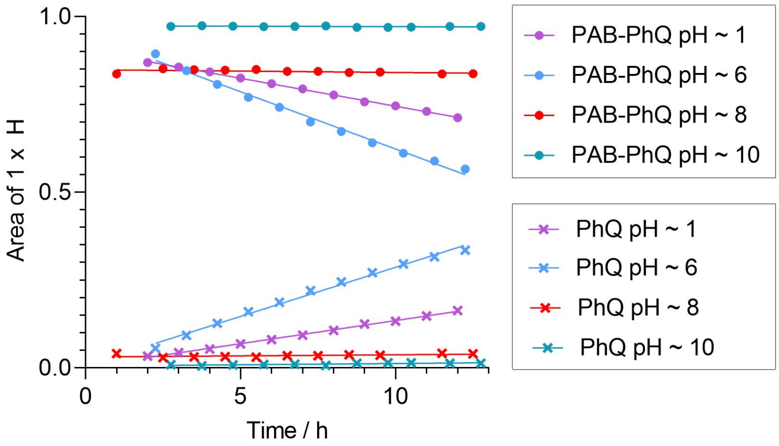

b Colour of NMR tubes at t = 12 h. Left to right: pH ~ 1, pH ~ 6, pH ~ 8, pH ~ 10.

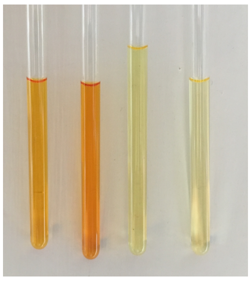

c pH ~ 1

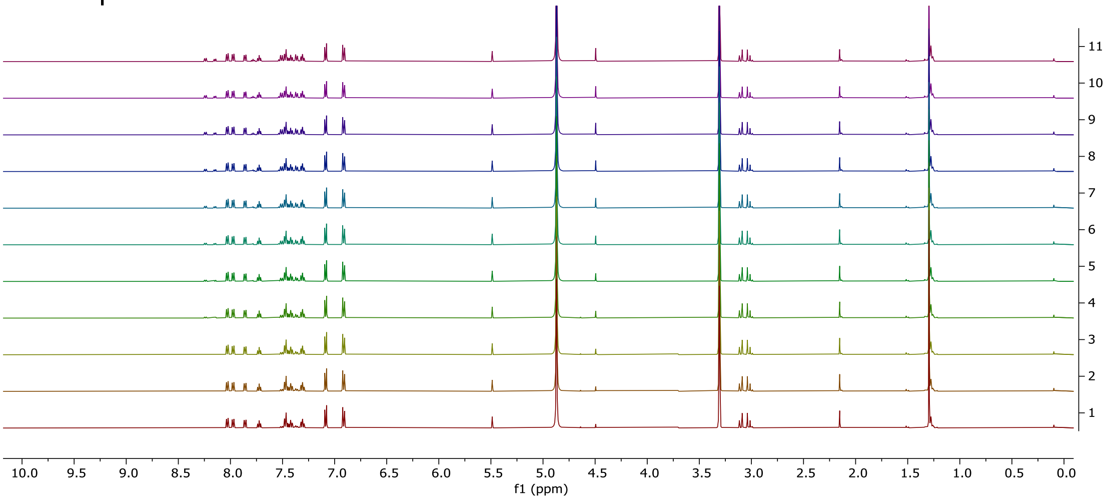

d pH ~ 6

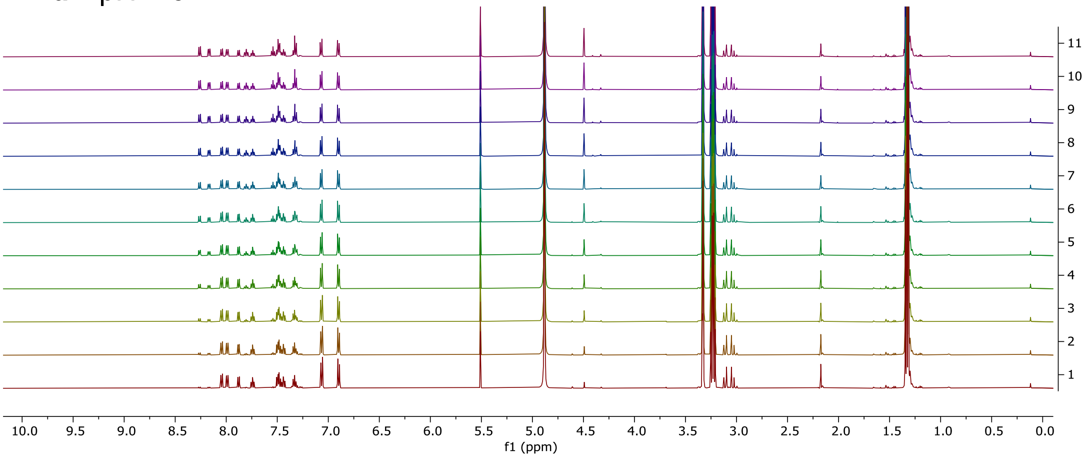

e 9,10-phenanthrenequinone

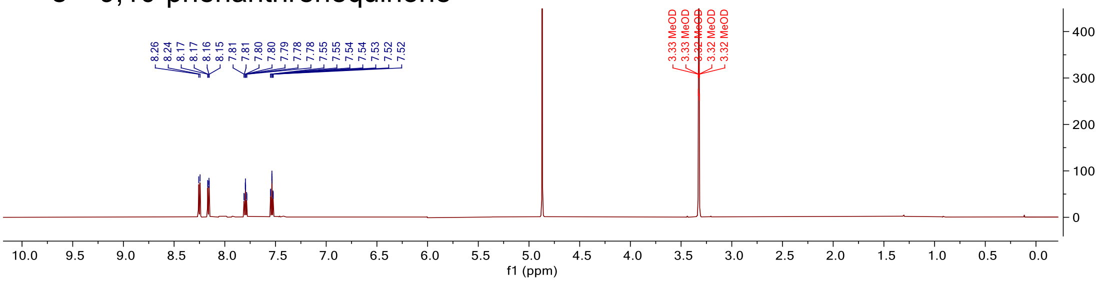

f pH ~ 8

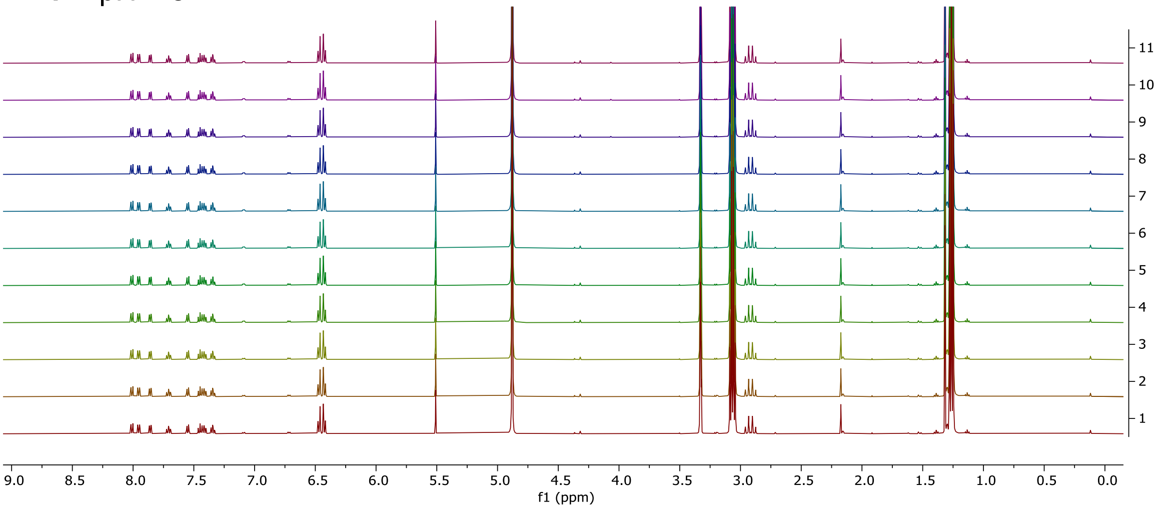

g pH ~ 10

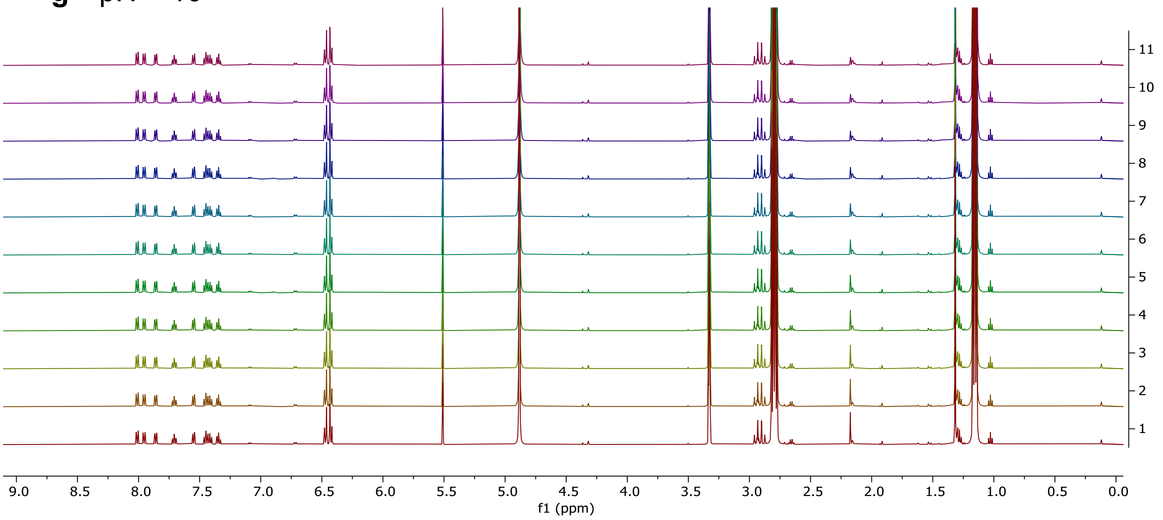

Figure 6. Analysis of the elimination rate of PAB-PhQ 7 over 12 h. This experiment was performed once. Similar experiments are displayed in **Supplementary Figures 1–5**, demonstrating proof-of concept of the elimination.

Proof-of-concept of 1,6-elimination by ^{1}H NMR

PAB-BL **10** ($CDCl_3$)

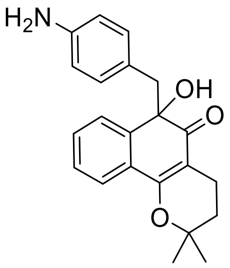

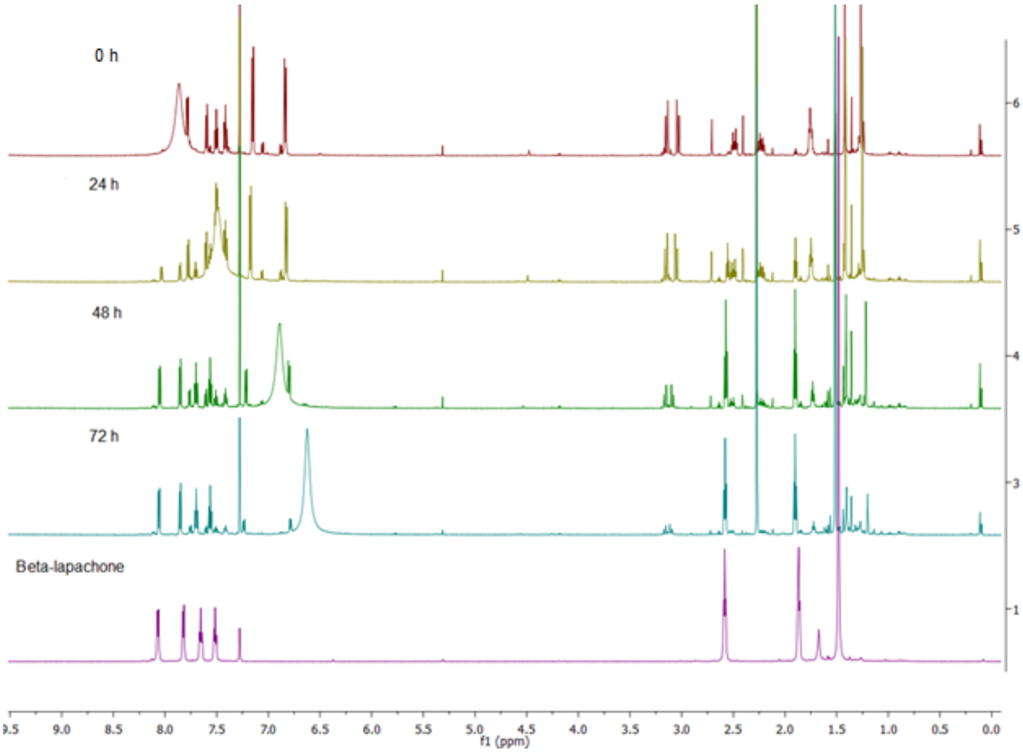

Figure 7. Proof of concept of elimination from PAB-BL 10 under acidic conditions by ^{1}H NMR in $CDCl_3$. Emergence of quinone peaks apparent. Note: a substance precipitated out of the solution, correlating with the disappearance of the aniline aromatic peaks. Replicate experiments displayed similar results.

PAB-BL **10** (MeOD)

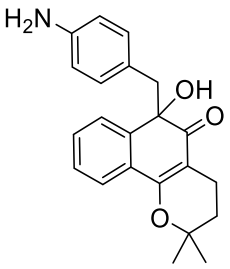

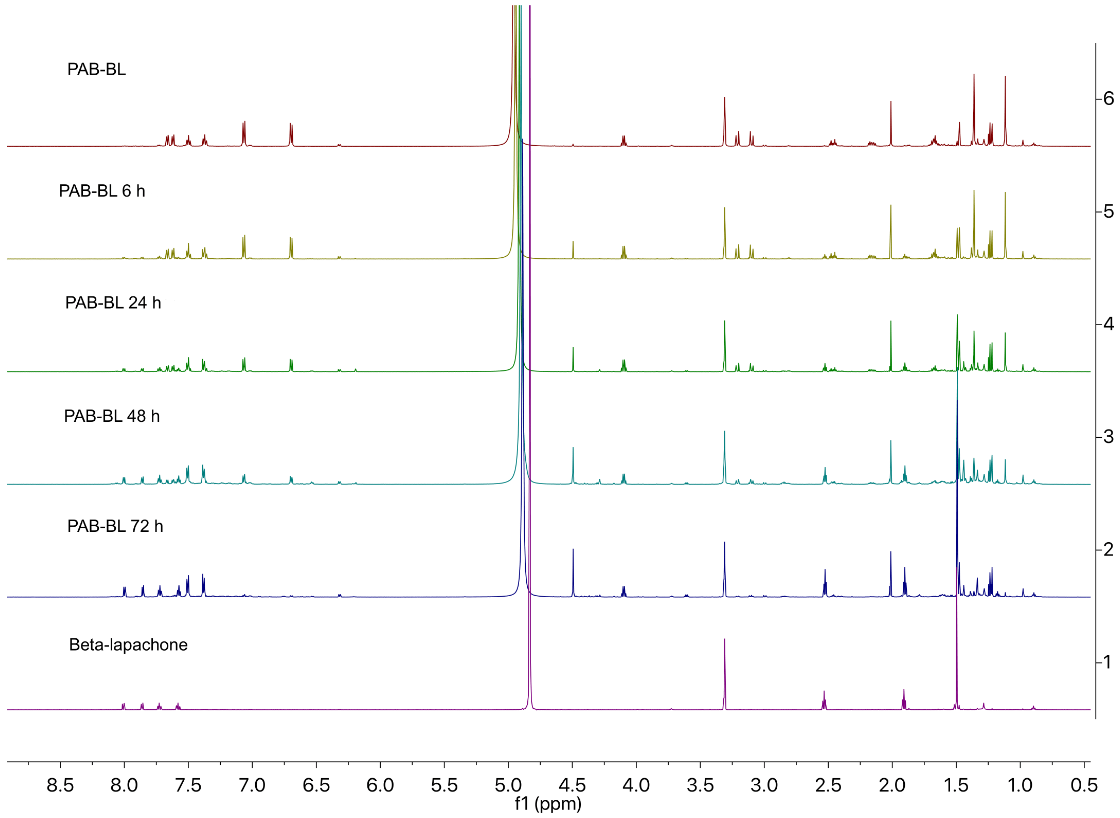

Figure 8. Proof of concept of elimination from PAB-BL 10 under acidic conditions by ^{1}H NMR in MeOD. Emergence of quinone peaks apparent. Replicate experiments displayed similar results.

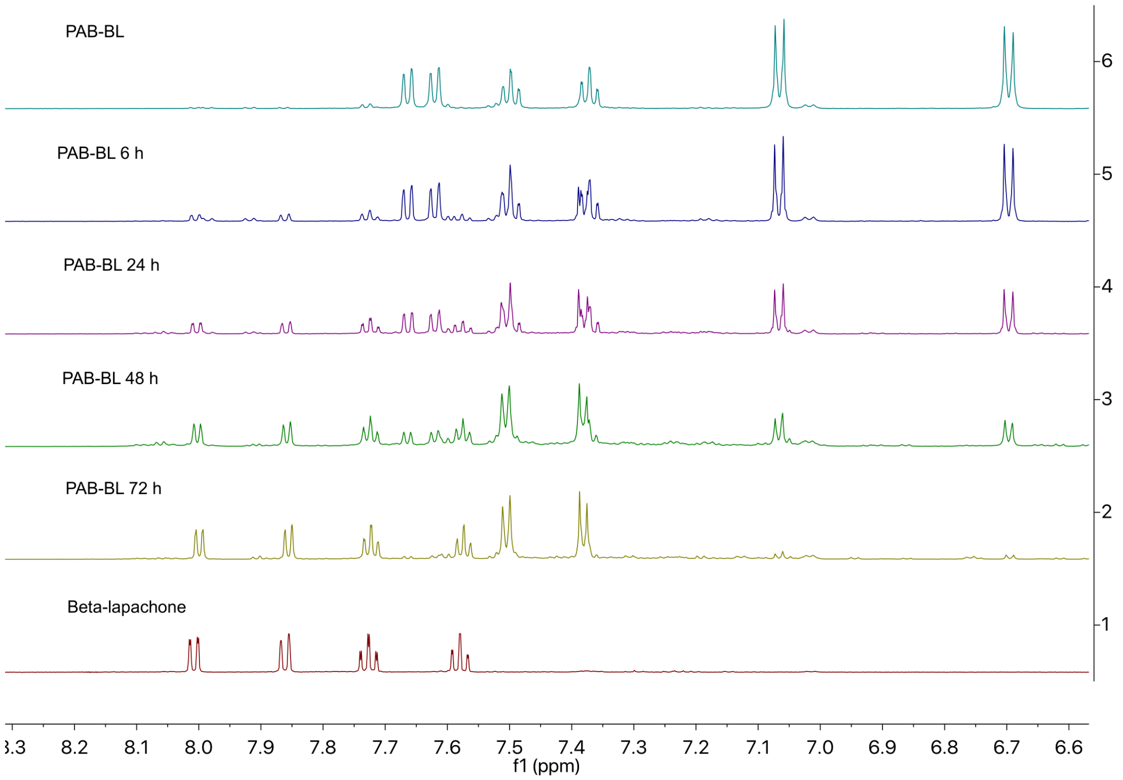

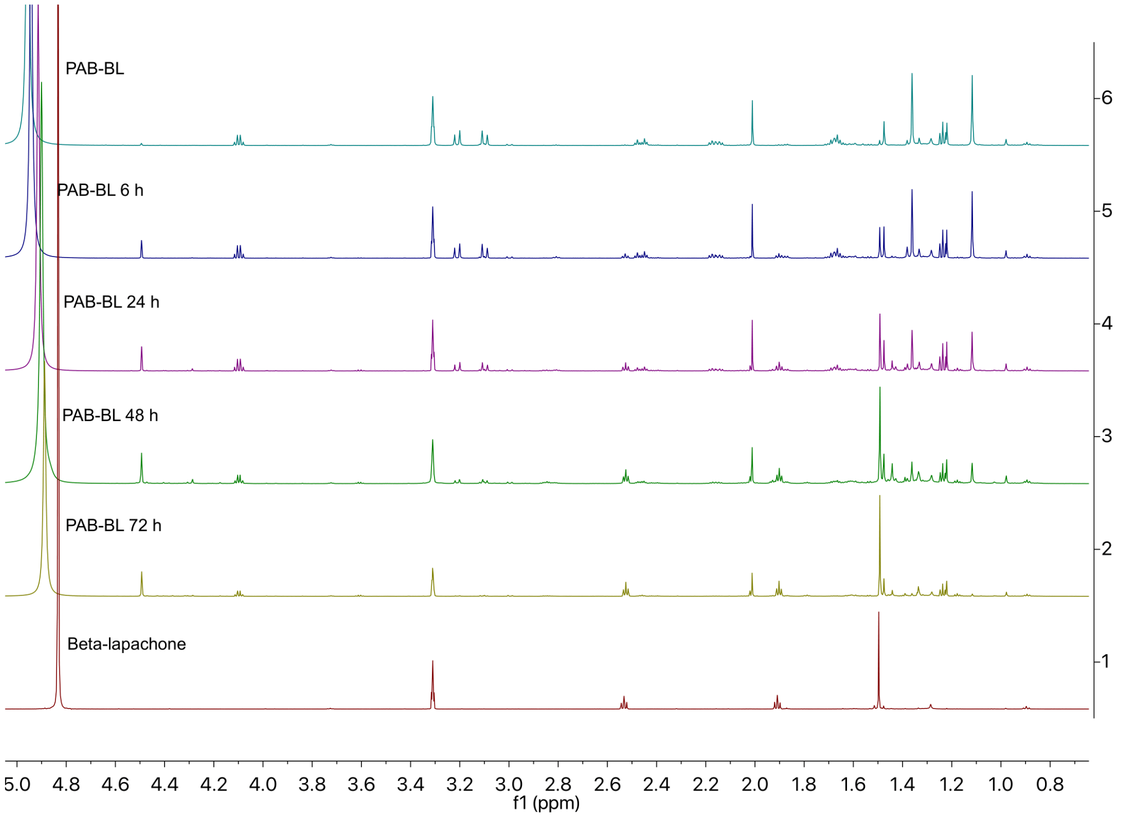

Figure 9. Zoom of spectra in Figure 8.

PAB-HBL **17** (MeOD)

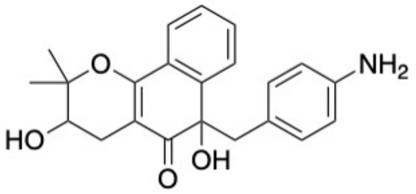

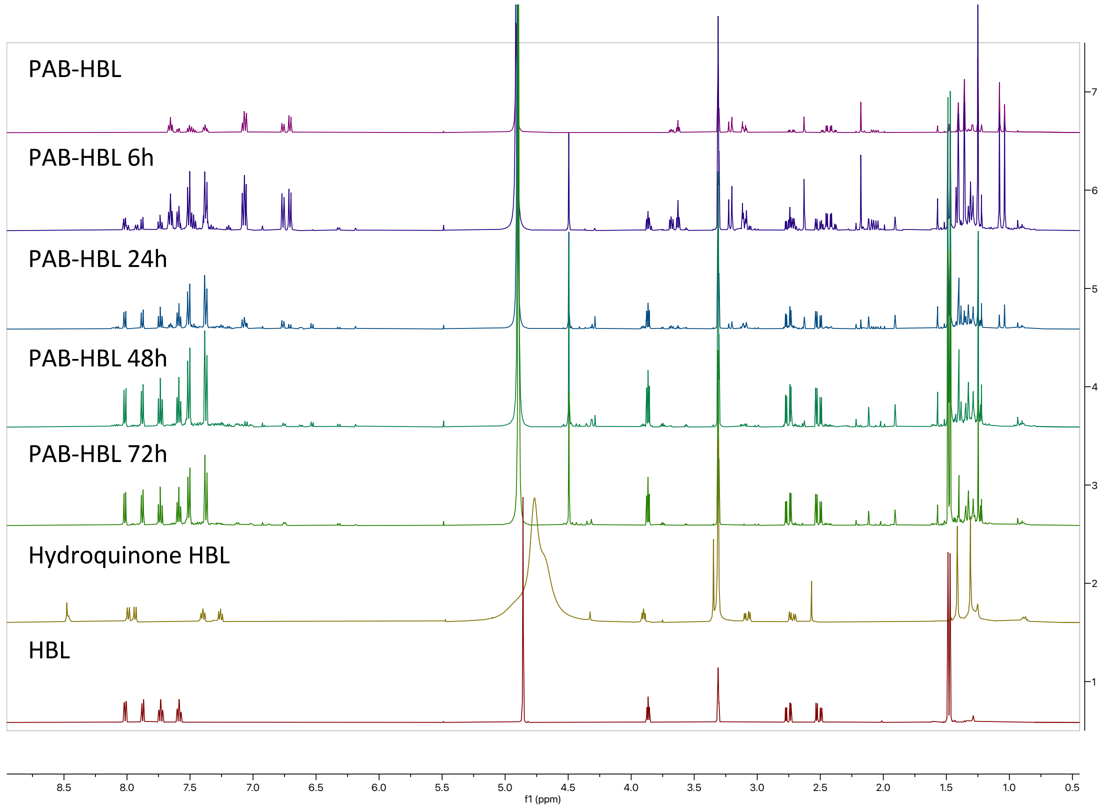

Figure 10. **Proof of concept of elimination from PAB-HBL 17 under acidic conditions by ^{1}H NMR in MeOD.** Emergence of quinone peaks apparent. Emergence (6h) and disappearance (24h) of hydroquinone HBL peaks also apparent. Replicate experiments displayed similar results.

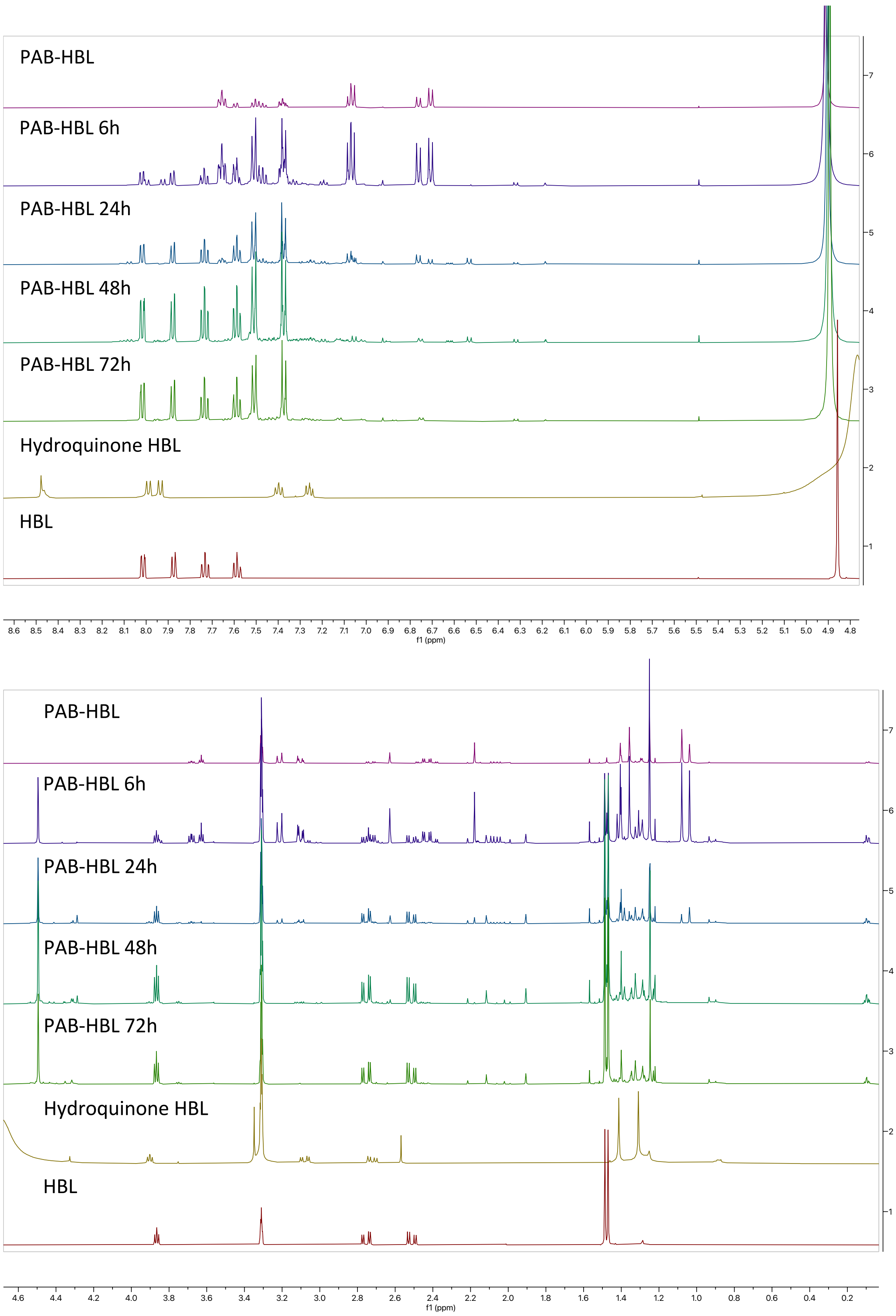

Figure 11. Zoom of spectra in Figure 10.

PAB-DN **18** (MeOD)

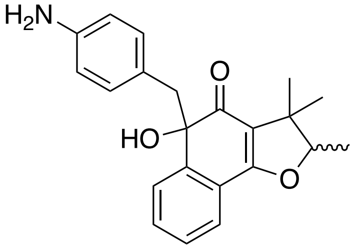

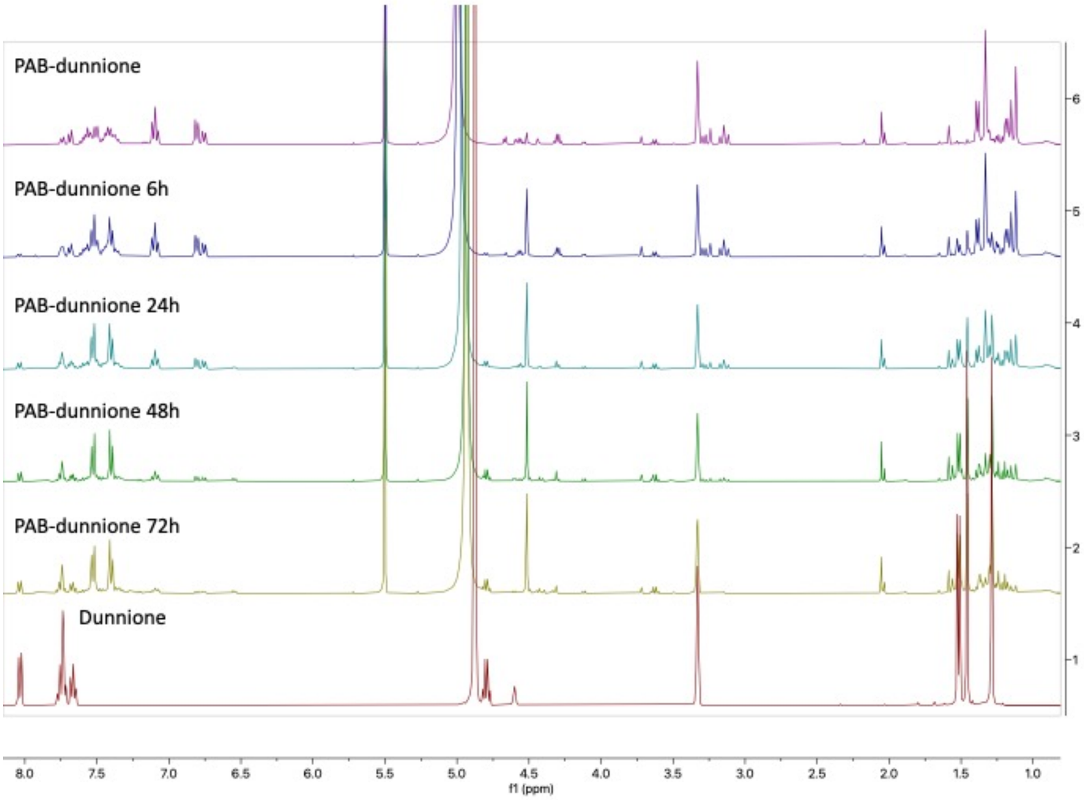

Figure 12. Proof of concept of elimination from PAB-DN 18 under acidic conditions by ^{1}H NMR in MeOD. Emergence of quinone peaks apparent. Replicate experiments displayed similar results.

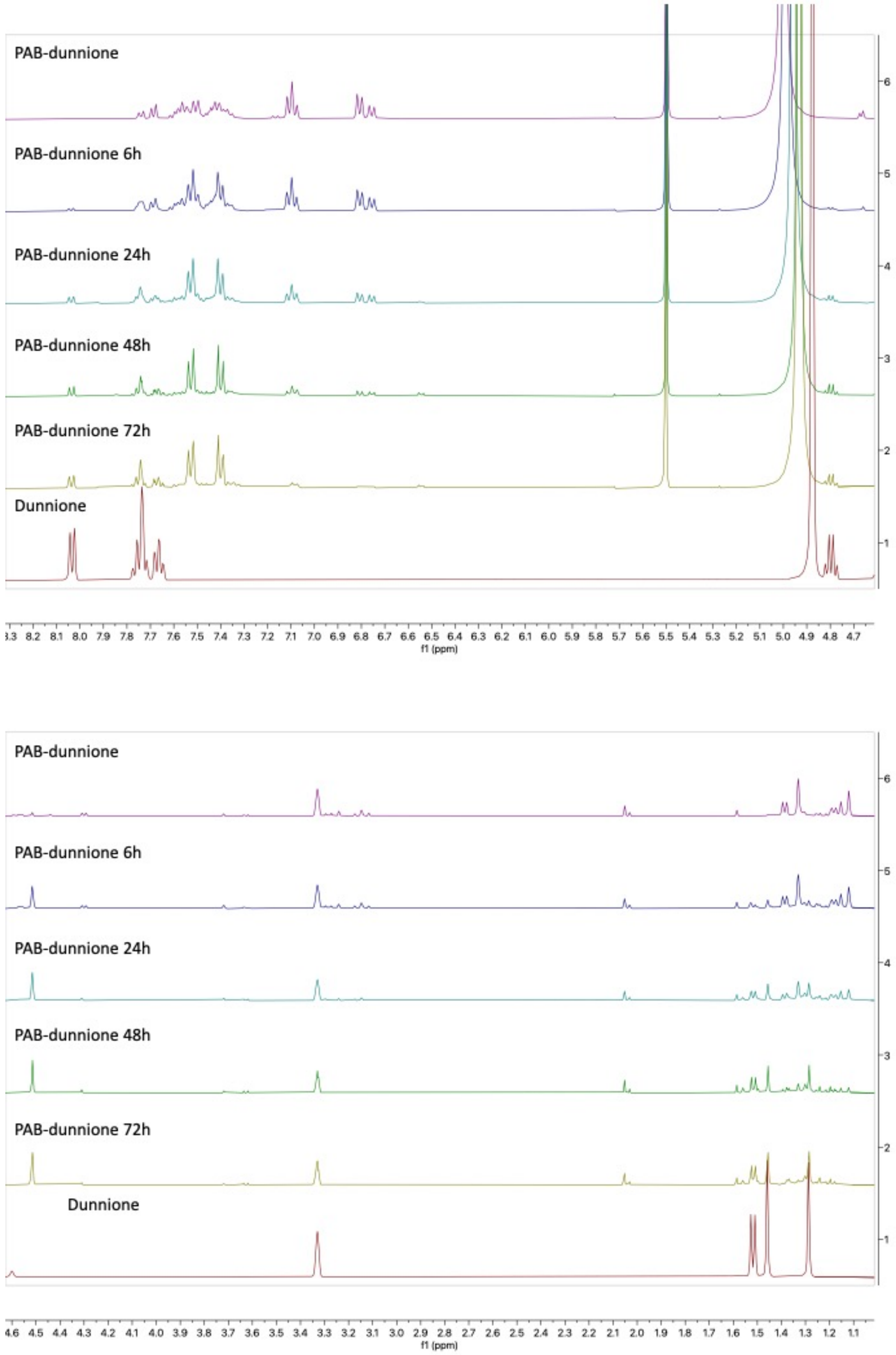

Figure 13. Zoom of spectra in Figure 12.

PAB-CTN **19** (MeOD)

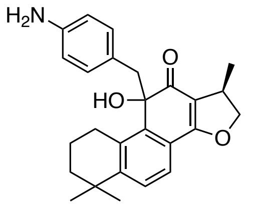

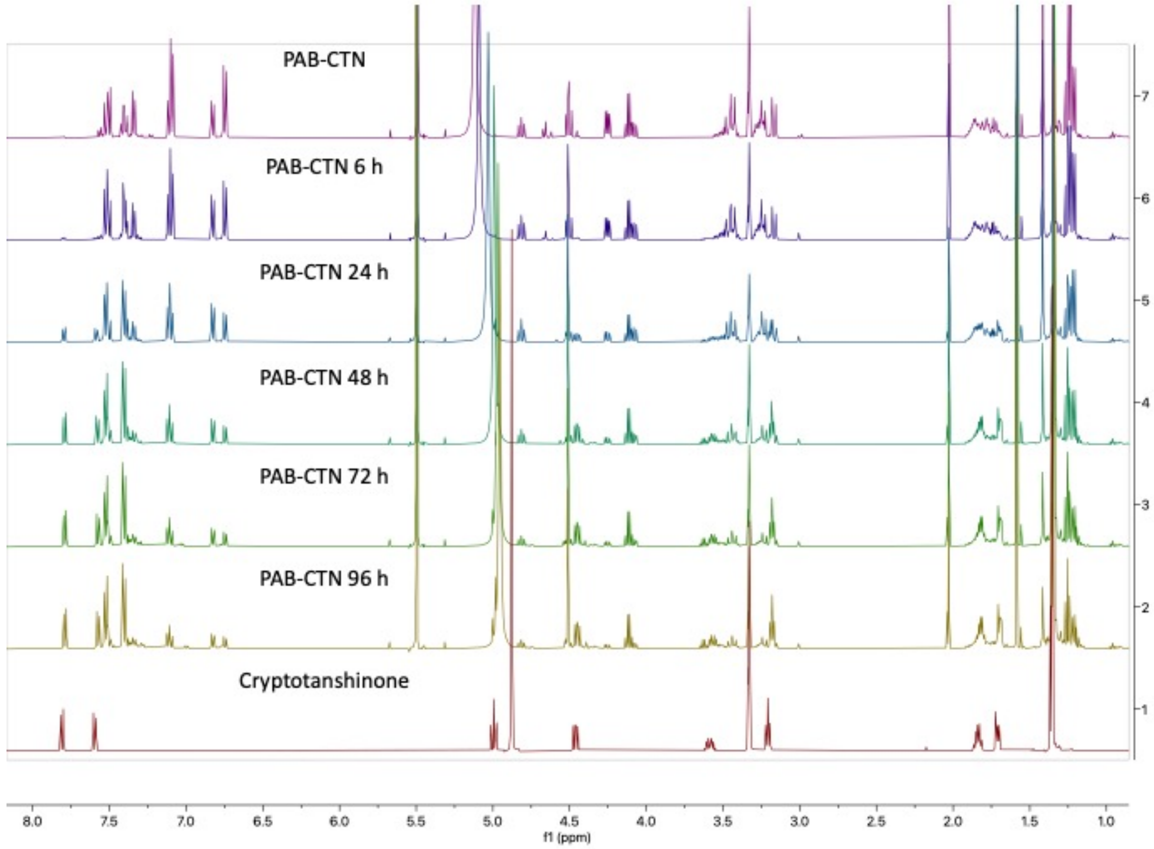

Figure 14. Proof of concept of elimination from PAB-CTN 19 under acidic conditions by ^{1}H NMR in MeOD. Emergence of quinone peaks apparent. Replicate experiments displayed similar results.

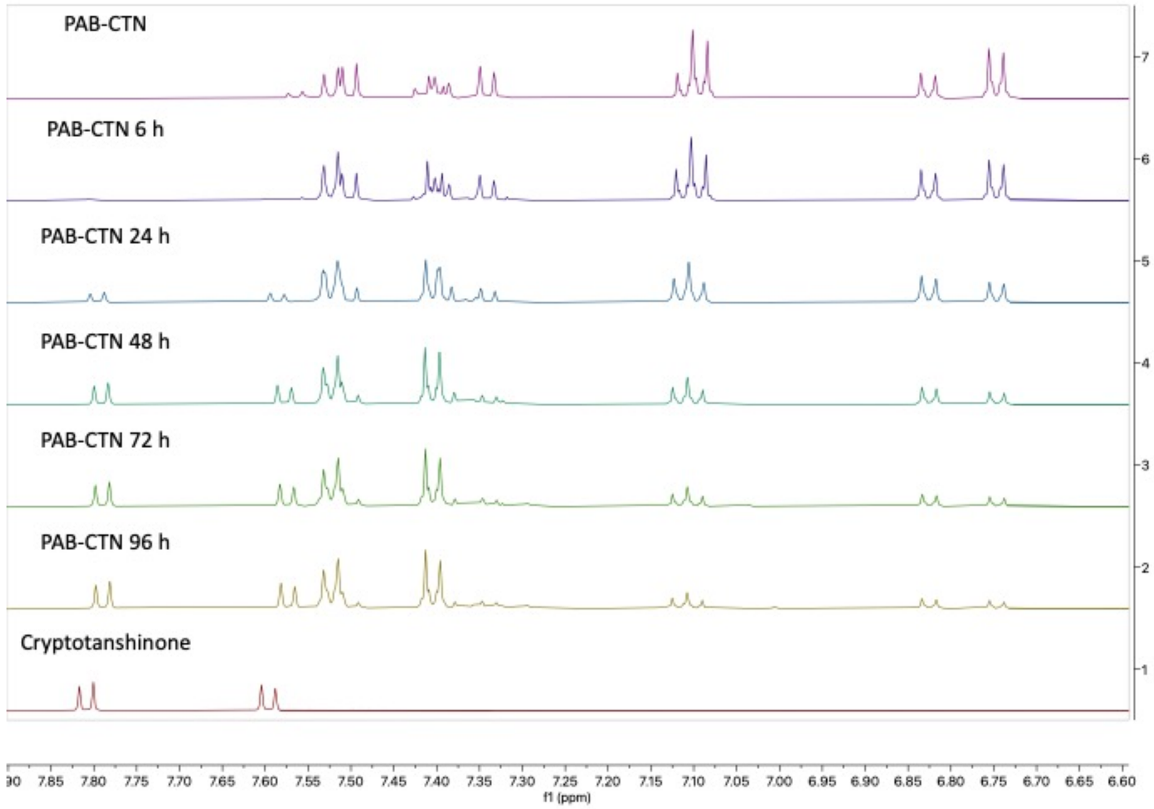

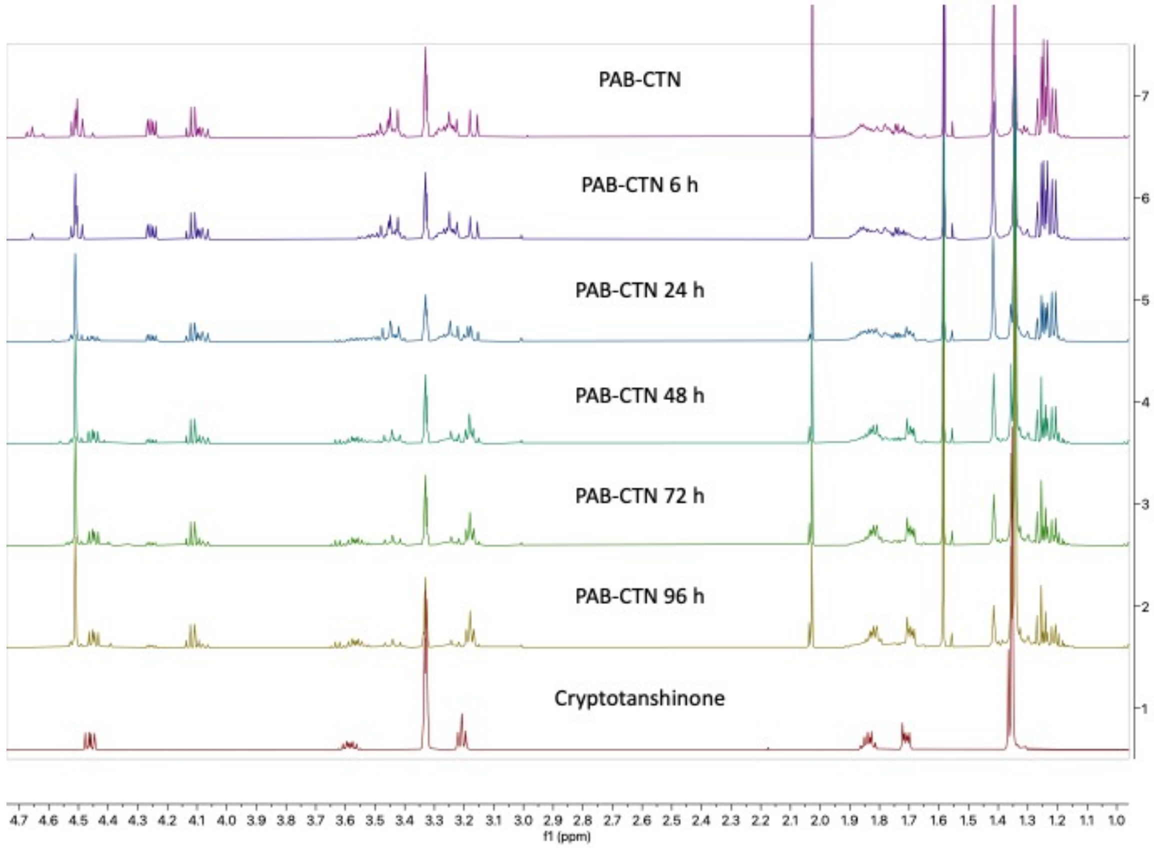

Figure 15. Zoom of spectra in Figure 14.

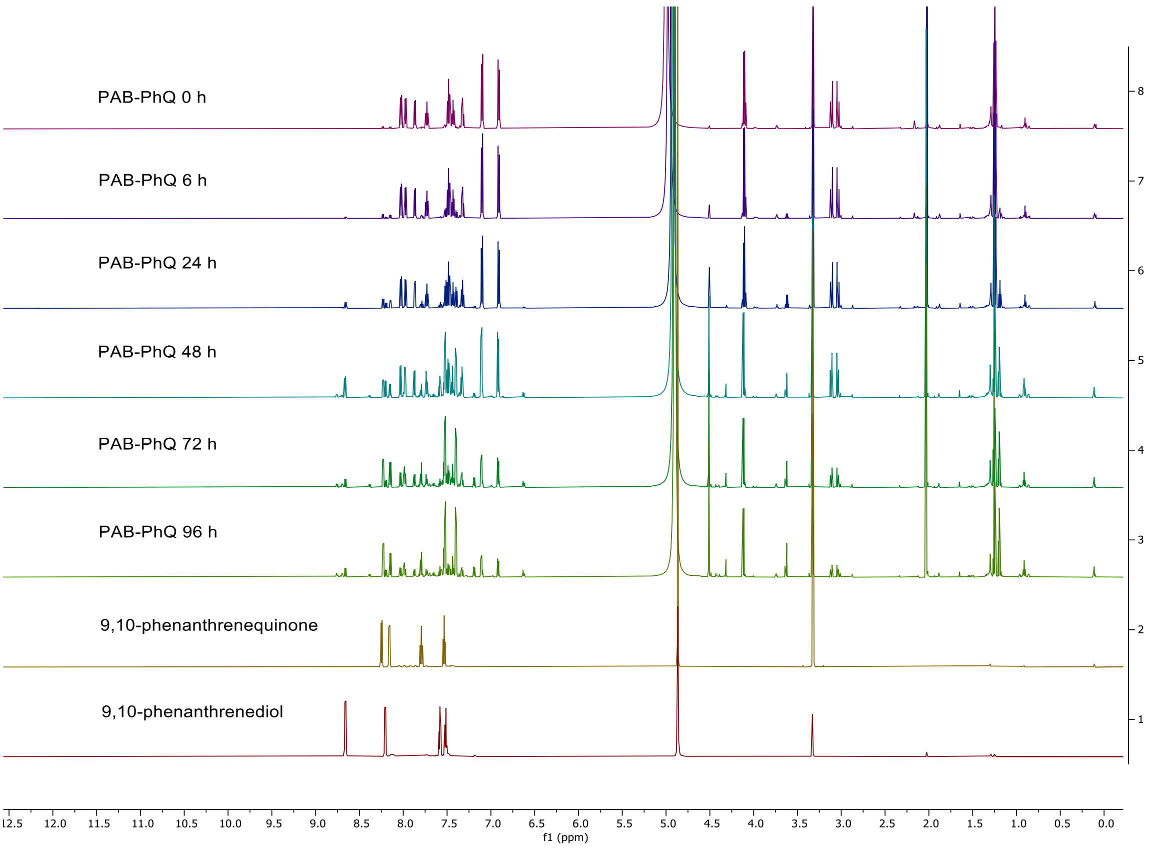

Figure 16. Elimination from PAB-PhQ 7 in degassed MeOD. ^{1}H NMR experiment was performed at neutral pH in degassed solvent. Hydroquinone **8** is observed. NMR solvent was degassed by nitrogen bubbling prior to placement in the NMR tube. The NMR tube was not deoxygenated and over time the solution oxygenated. This procedure allowed observation of the unstable hydroquinone intermediate, which can be observed to oxidise to the quinone at later timepoints. This experiment was performed at neutral pH (~ 7). Replicate experiments displayed similar results.

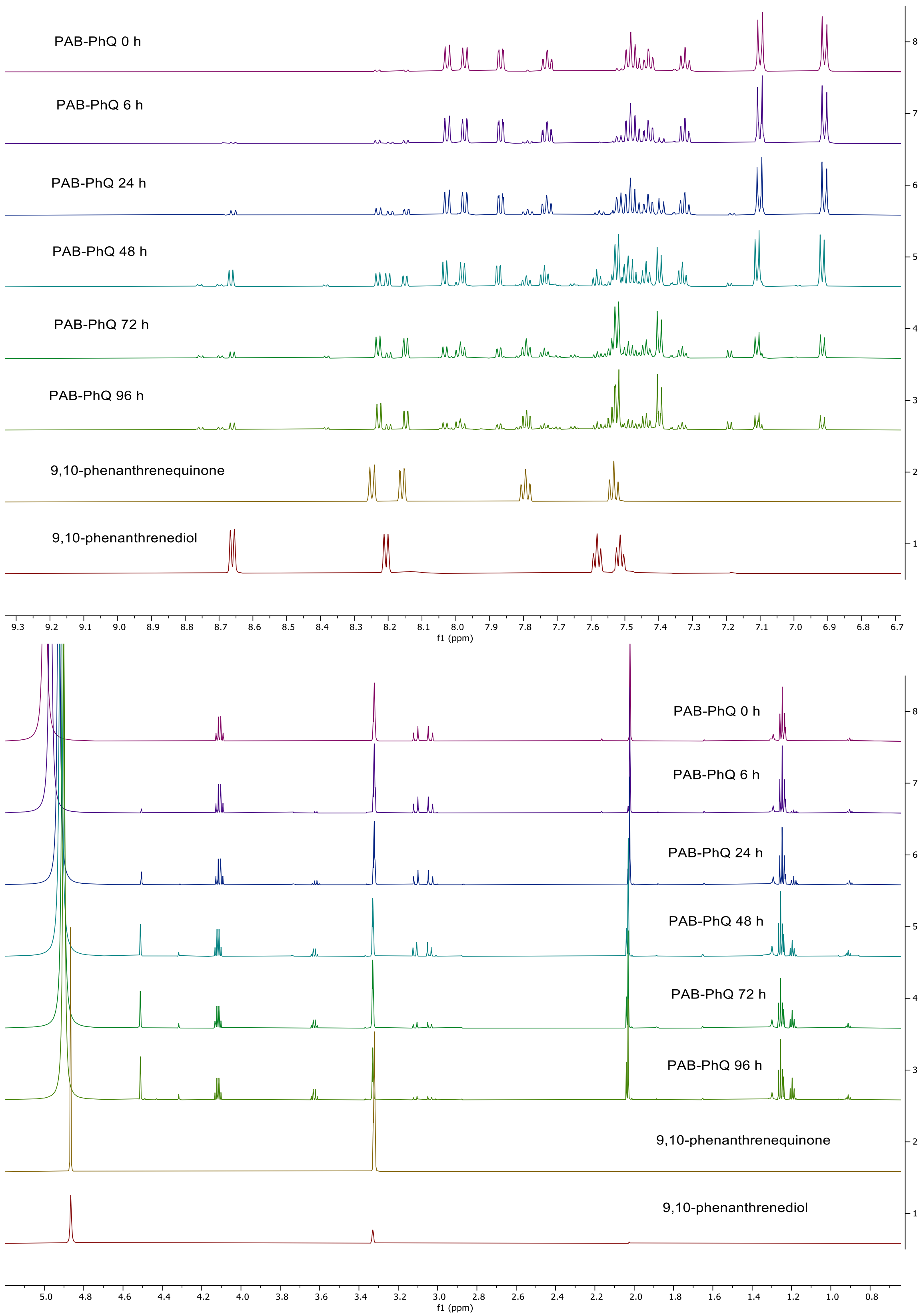

Figure 17. Zoom of Figure 1616.

Elimination side product

In eliminations performed in methanol, an additional product was identified, characterised by an emergent benzylic CH_2 peak at $\delta = 4.5$ ppm. The CH_2 peak integrated correctly in relation to the released quinone product. The side product was identified to be an aza-quinone methide condensation product that was either 4-(methoxymethyl)-aniline formed by condensation of the methanol solvent or *para*-aminobenzyl alcohol by analogous condensation of water. The CH_3 peak of 4-(methoxymethyl)-aniline overlaps with the solvent peak of the methanol solvent, and as the aromatic aniline and benzylic peaks shift depending on the proton environment of the sample, so ^{1}H NMR cannot distinguish between the two possible condensation products in this instance.

a

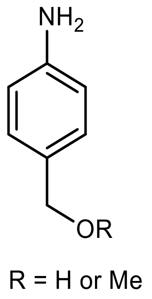

b

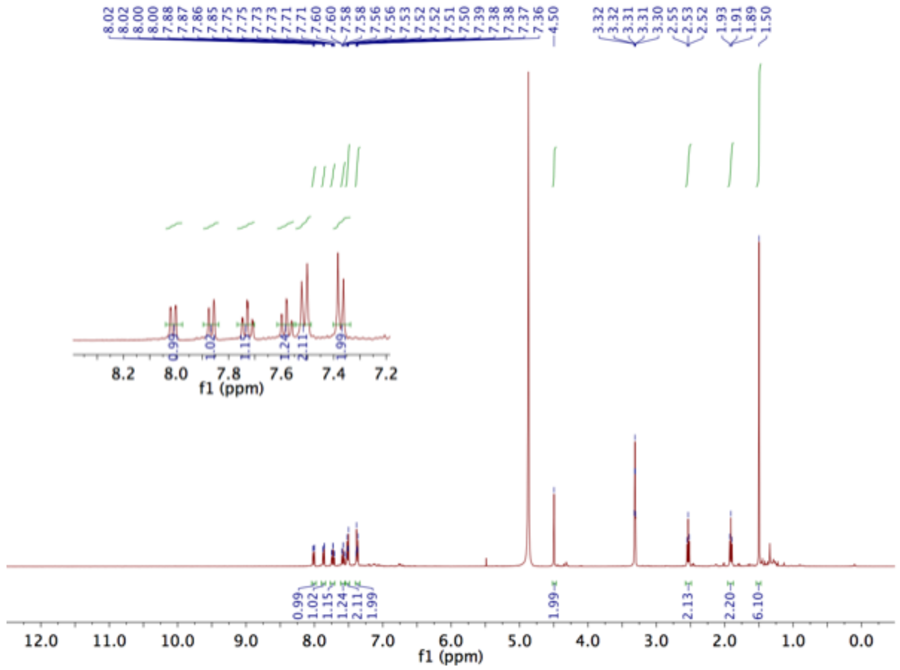

Figure 18. Side product. a, Structure of proposed product. **b**, ^{1}H NMR of products present after PAB-BL **10** elimination in acidic solution. Similar results to those displayed were obtained for all elimination experiments performed in methanol.

Alfa-ketol rearrangement

It was analysed whether PAB-BL **10** could undergo an alfa-ketol rearrangement to an isomeric form in base by migration of the benzyl substituent to the adjacent ketone. However, only one species was observed under basic conditions, so it was concluded that this migration does not occur to any significant extent.

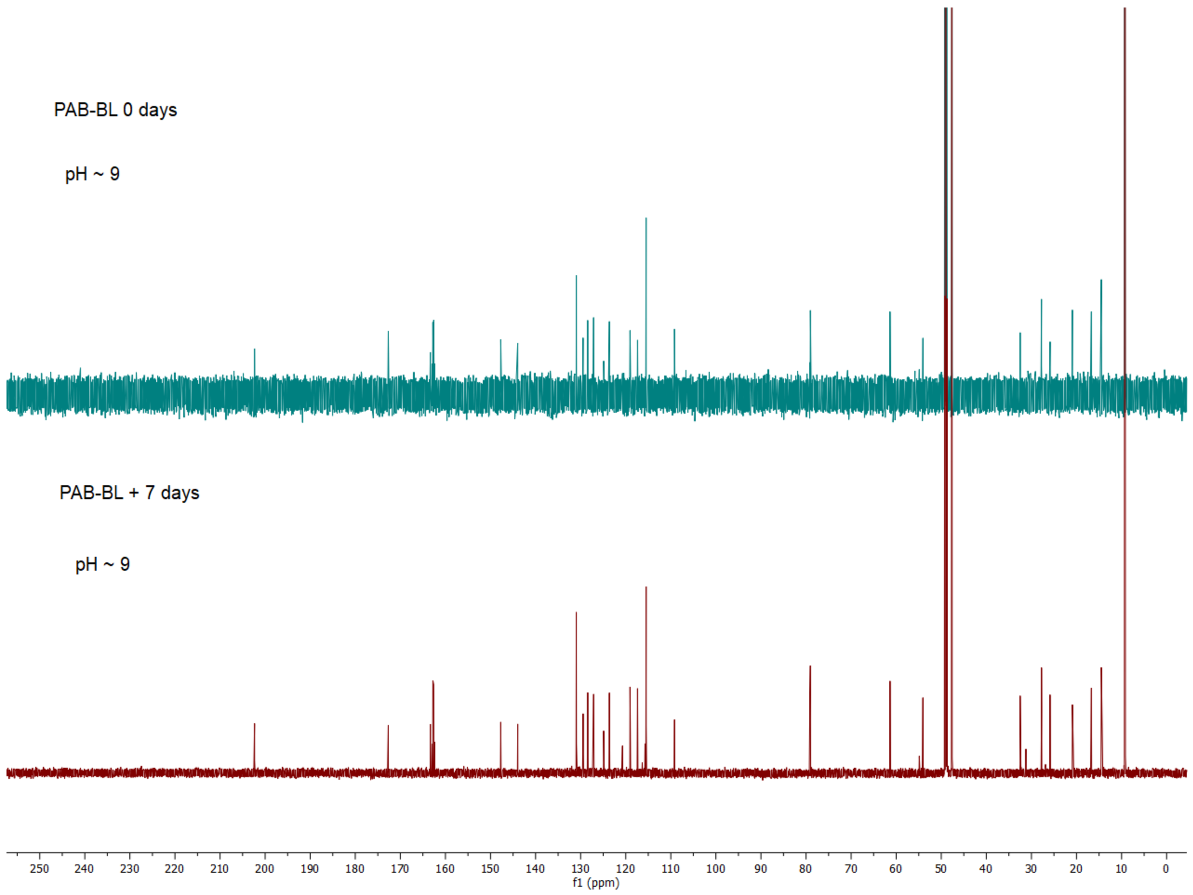

Figure 19. ^{13}C NMR of PAB-BL 10 at basic pH (~ pH 9) in MeOD. pH was adjusted to ~ 9 by addition of triethylamine. This experiment was performed once.

Proof-of-concept of elimination by UV/Vis

UV/Vis analysis of the benzyl-ketol elimination was performed.

a

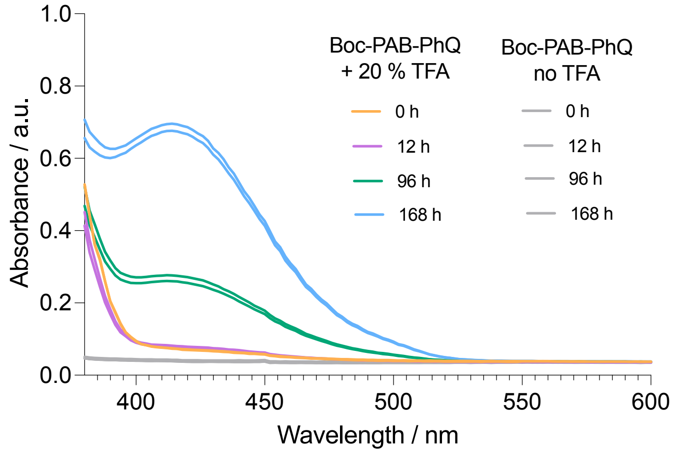

b

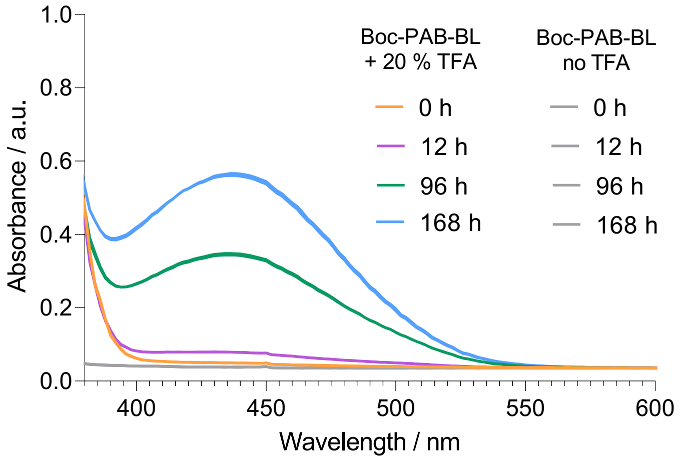

Figure 20. Elimination of benzyl ketol species PAB-PhQ 7 and PAB-BL 10 in acidic solution followed by UV/Vis. a, Formation of 9,10-phenanthrenequinone **4** from Boc-PAB-PhQ **6**, with 20% TFA (~ pH 1). **b**, Formation of β-lapachone **1** from Boc-PAB-BL **9**, with 20 % TFA (~ pH 1). This experiment was performed once and complements NMR analysis described above.

Method: Boc-*para*-aminobenzyl ketols were dissolved in 3:1 DMSO/H_2O to a concentration of 5 mM. 160 µL of compound stock was then added to wells of a 96 well plate. 40 µL of trifluoroacetic acid (TFA) was then added to the plate well to achieve a 20% TFA concentration and effect Boc-group deprotection at ~ pH 1. The UV/Vis absorption spectrum was read immediately and at specified time intervals. In between readings the plate was sealed and stored at room temperature in the dark.

a

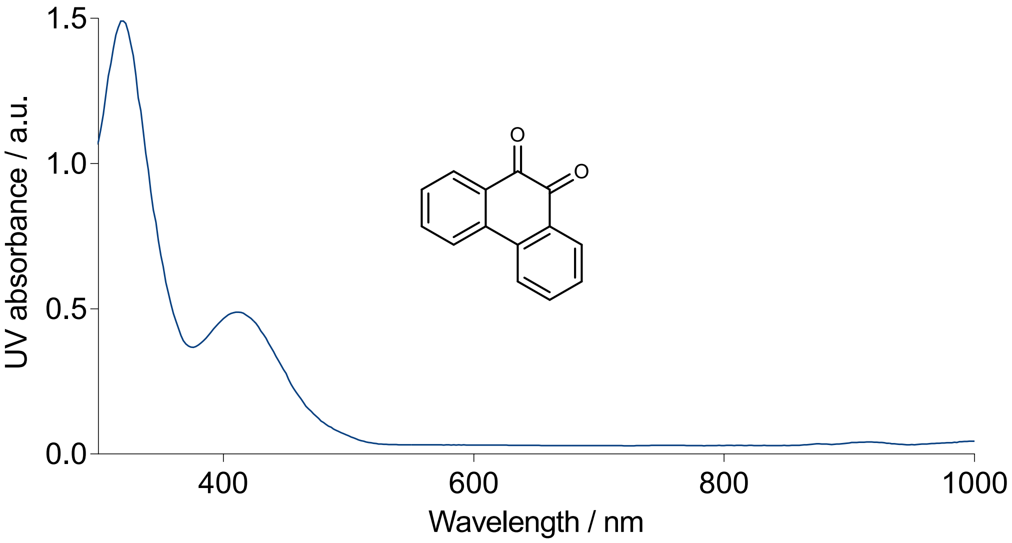

λ$_{max}$ observed at 320 nm, 410 nm.

b

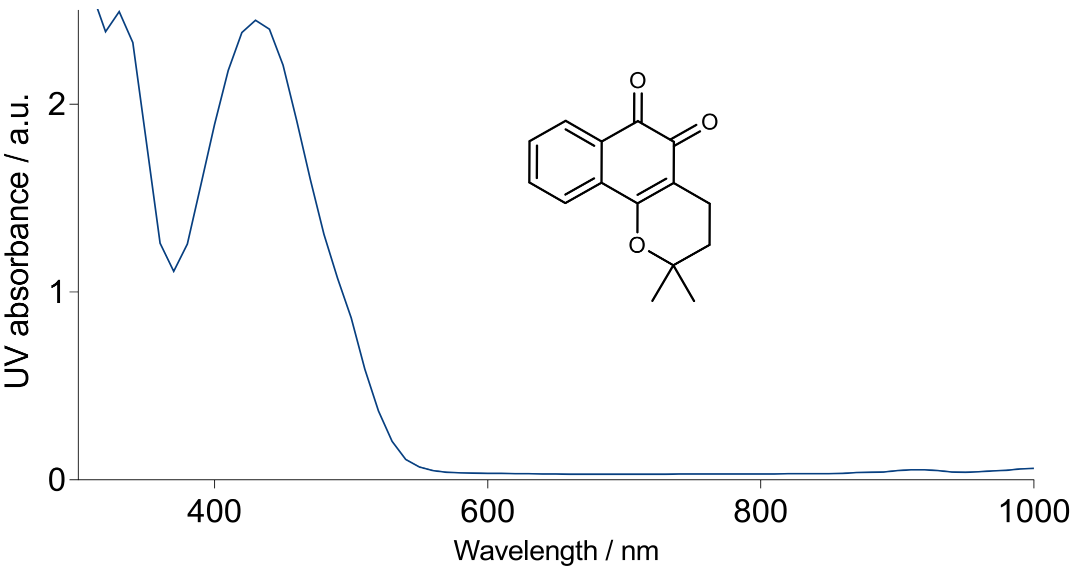

λ$_{max}$ observed at 330 nm, 430 nm.

Figure 21. **UV/Vis absorption profiles of 9,10-phenanthrenequinone 4 and β-lapachone 1 above 300 nm. a,** 9,10-phenanthenequinone **4** UV absorption spectra. **b,** β-lapachone **1** UV absorption spectra.

a

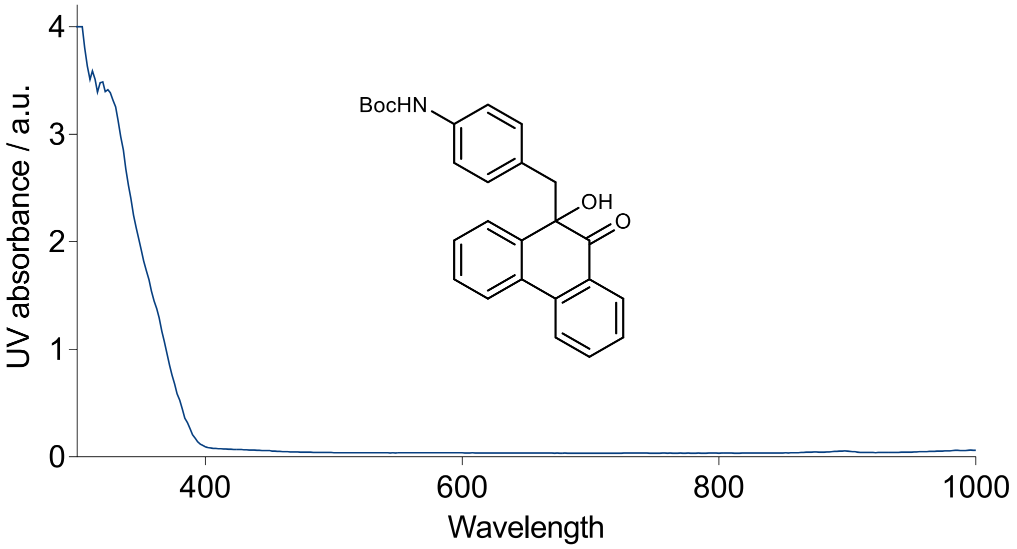

b

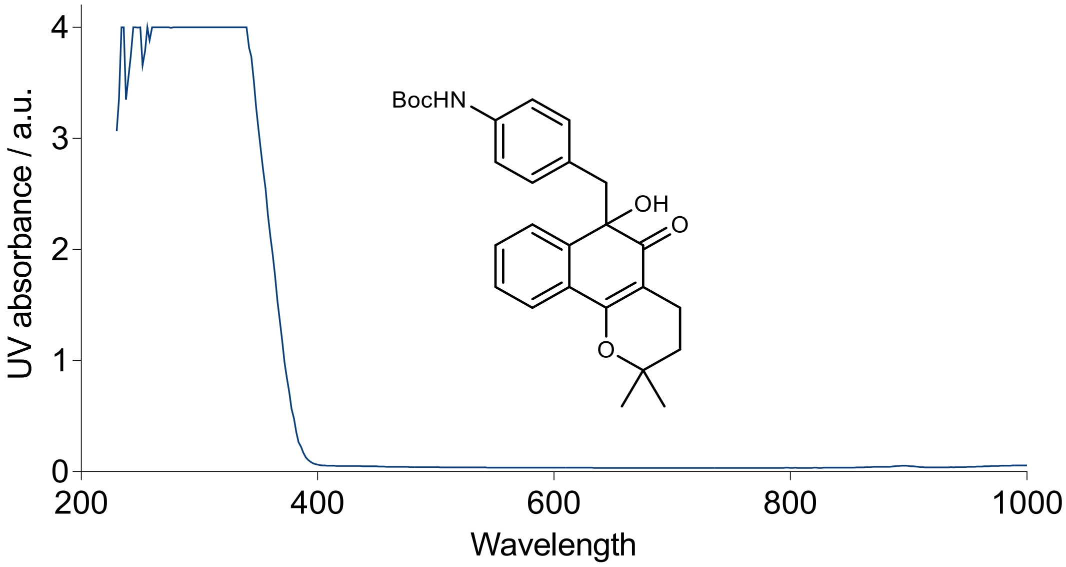

Figure 22. **UV/Vis absorption profiles of Boc-PAB-PhQ 6 and Boc-PAB-BL 9 above 300 nm. a,** Boc-PAB-PhQ **6** UV absorption spectra. **b,** Boc-PAB-BL **9** UV absorption spectra.

Kinetics of release of quinone PhQ 4 from 2-phenylacetyl-PAB-PhQ 21 upon amidase trigger at pH 7.4

Hydrolysis of 2-phenyl acetamide group in **21** occurred rapidly upon Pencillin-G amidase addition (< 5 min) and allows analysis of the rate of fragmentation of **7**. Addition of enzyme to test samples hydrolyses the amide bond leaving the amine group free to fragment.

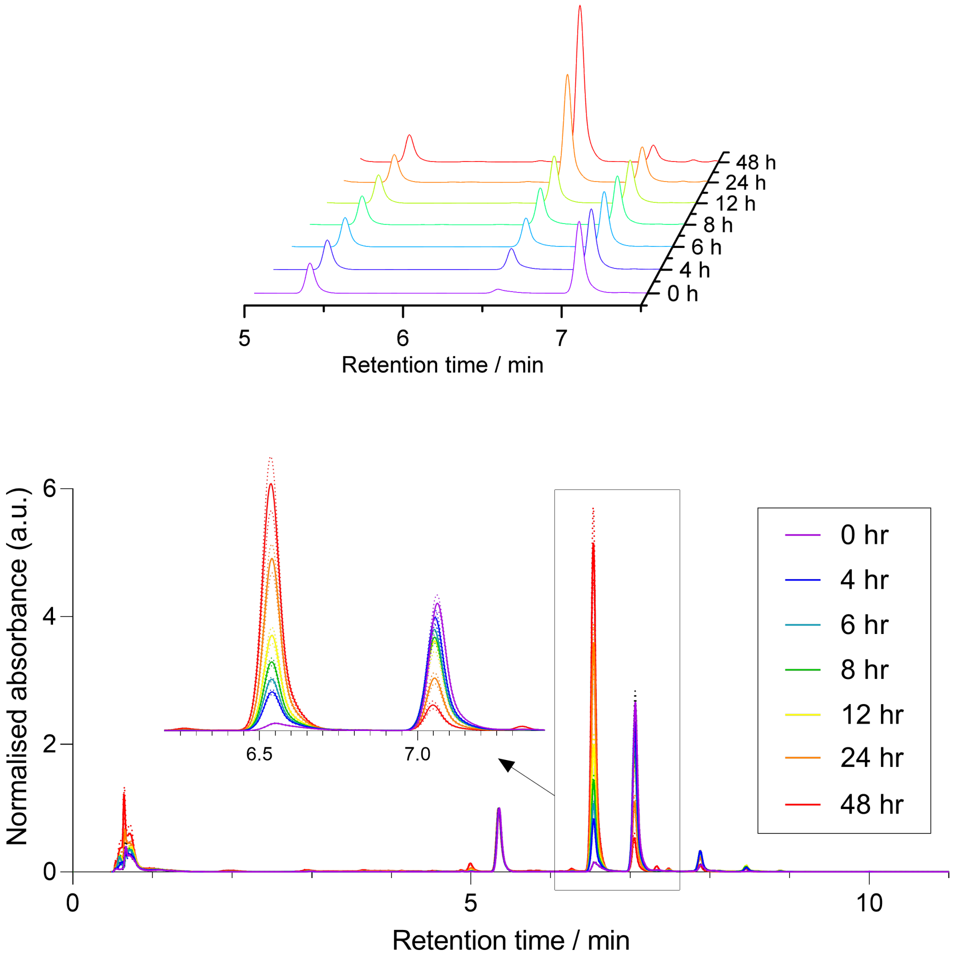

Figure 23. Penicillin G amidase assay to measure elimination of PAB-PhQ 7 at pH 7.4, 37 °C. HPLC analysis of formation of PhQ **4** (6.53 min) following elimination of PAB-PhQ **7** (7.05 min). PAB-PhQ formed by addition of Penicillin-G amidase enzyme to 2-phenylacetyl-PAB-PhQ **21**, and incubation for 15 min at 37 °C. Spectra are normalised to acetophenone internal standard (5.34 min). Displayed spectra are the averaged trace of three independent reactions. Note consumption of 2-phenylacetyl-PAB-PhQ (7.05 min) **21** even in the first timepoint t = 0, taken < 5 min after amidase enzyme addition. This experiment was performed as described in the materials and methods with 0.1% formic acid not added to the solvent system. A replicate experiment displayed similar results.

Control experiment without addition of amidase to 2-phenylacetyl-PAB-PHQ 21, monitoring stability of 21 to pH 7.4, 37 °C

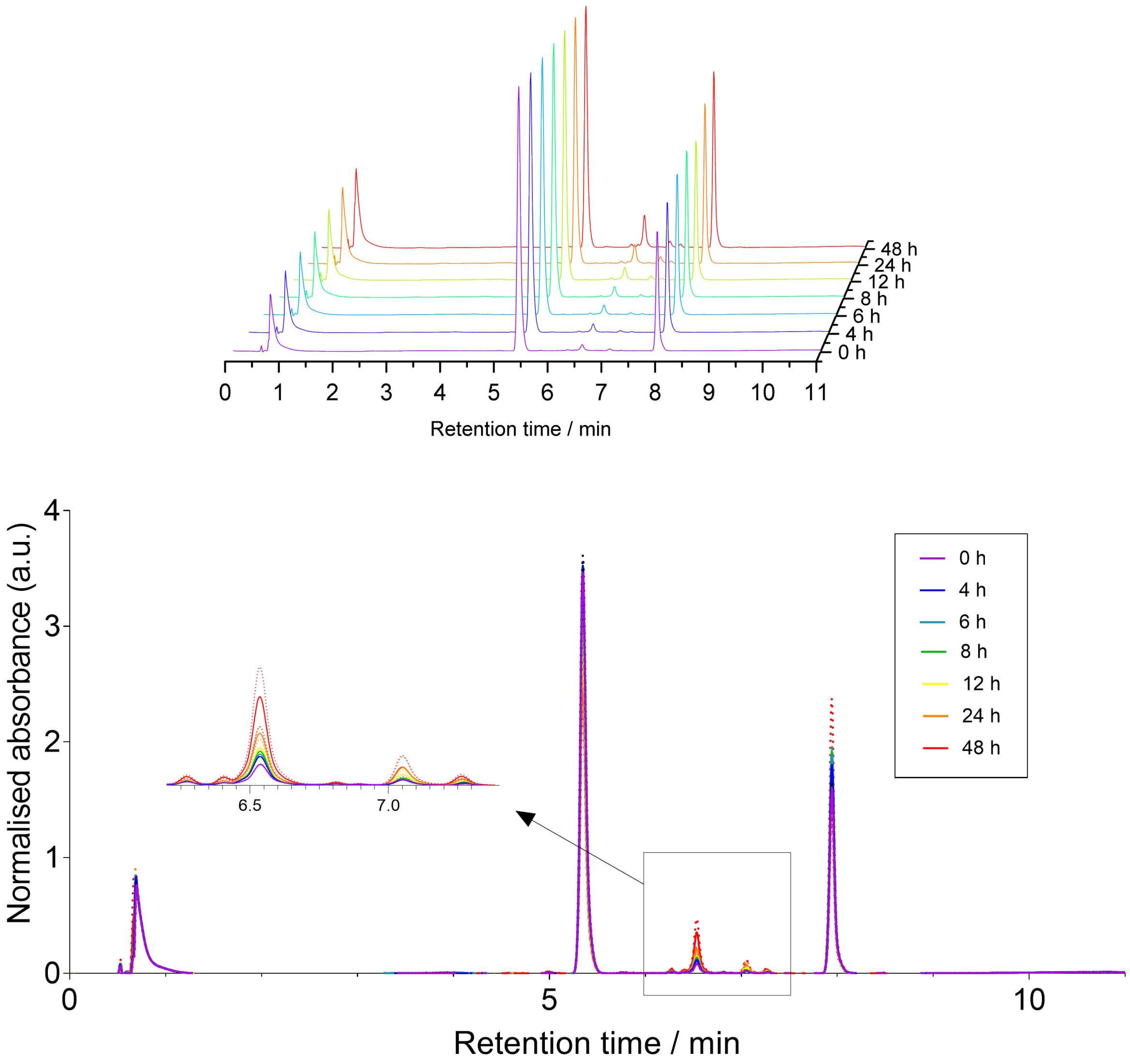

Figure 24. Testing release of PhQ 4 from protected 2-phenylacetyl-PAB-PhQ 21 in the absence of Penicillin-G amidase. Spectra are normalised to acetophenone internal standard (5.34 min). The PhQ **4** peak (6.53 min) displays very small increase over 48 h. Displayed spectra are the averaged trace of three independent reactions with standard error of the mean plotted as a dotted line. This experiment was performed as described in the materials and methods with 0.1% formic acid not added to the solvent system. A replicate experiment displayed similar results.

Peak assignment

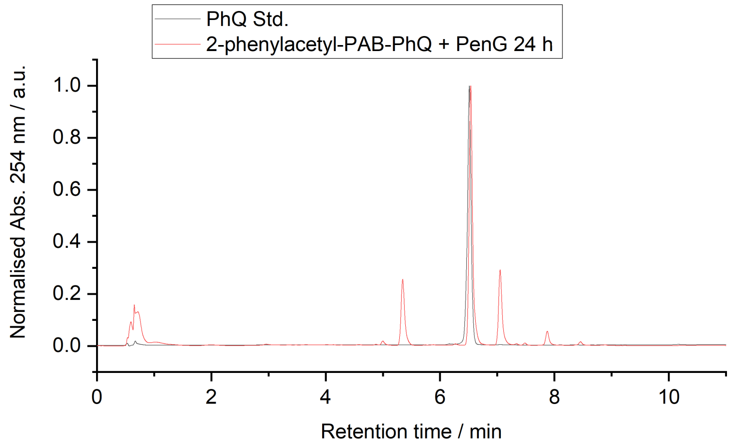

Figure 25. Assignment by overlay of released product from amidase assay with 2-phenylacetyl-PAB-PhQ 21 with PhQ 4 standard. Assignments: acetophenone internal standard 5.34 min, PhQ 6.53 min.

Analysis of PAB-PhQ 7 elimination rate at pH 7.4 from amidase assay data

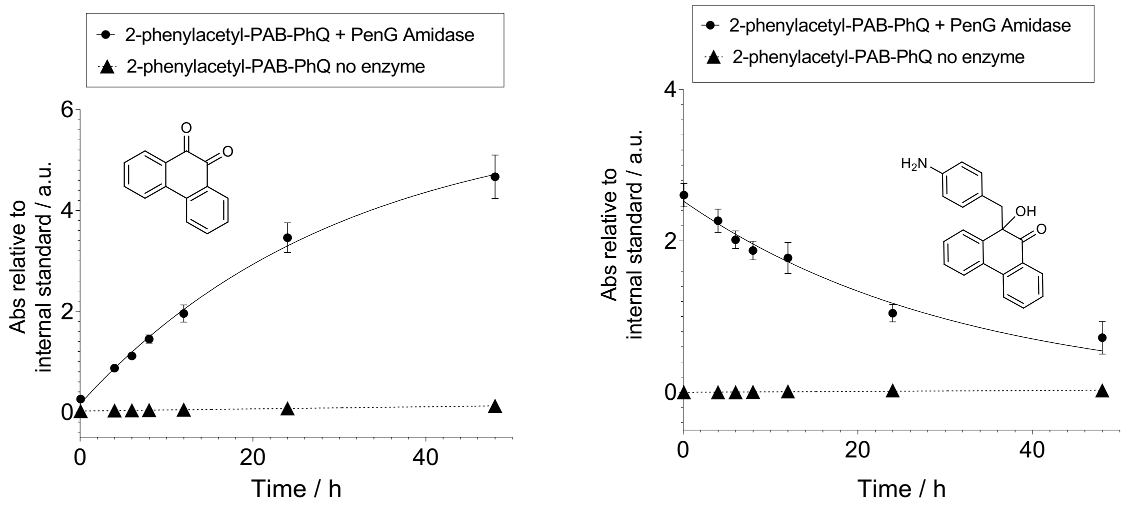

Compound	Buffer	pH	Half life $t_{1/2}$ / h	95 % CI / h	k / s^{-1}	95 % CI / s^{-1}	R^2
PAB-PhQ (consumption)	PBS	7.4	18.7	13.1 – 30.9	9.72×10^{-6}	6.84×10^{-6} – 1.33×10^{-5}	0.89
PhQ (formation)	PBS	7.4	20.9	13.8 – 40.8	1.15×10^{-5}	6.49×10^{-6} – 1.68×10^{-5}	0.94

Figure 26. First order kinetic analysis of elimination of PAB-PhQ 7 at pH 7.4, 37°C. Fitting performed to mean values of n=3 independent samples tested in one experiment. A replicate experiment displayed similar results.

Kinetics of release of β-lapachone 1 from 2-phenylacetyl-PAB-BL 22 upon amidase trigger at pH 7.4

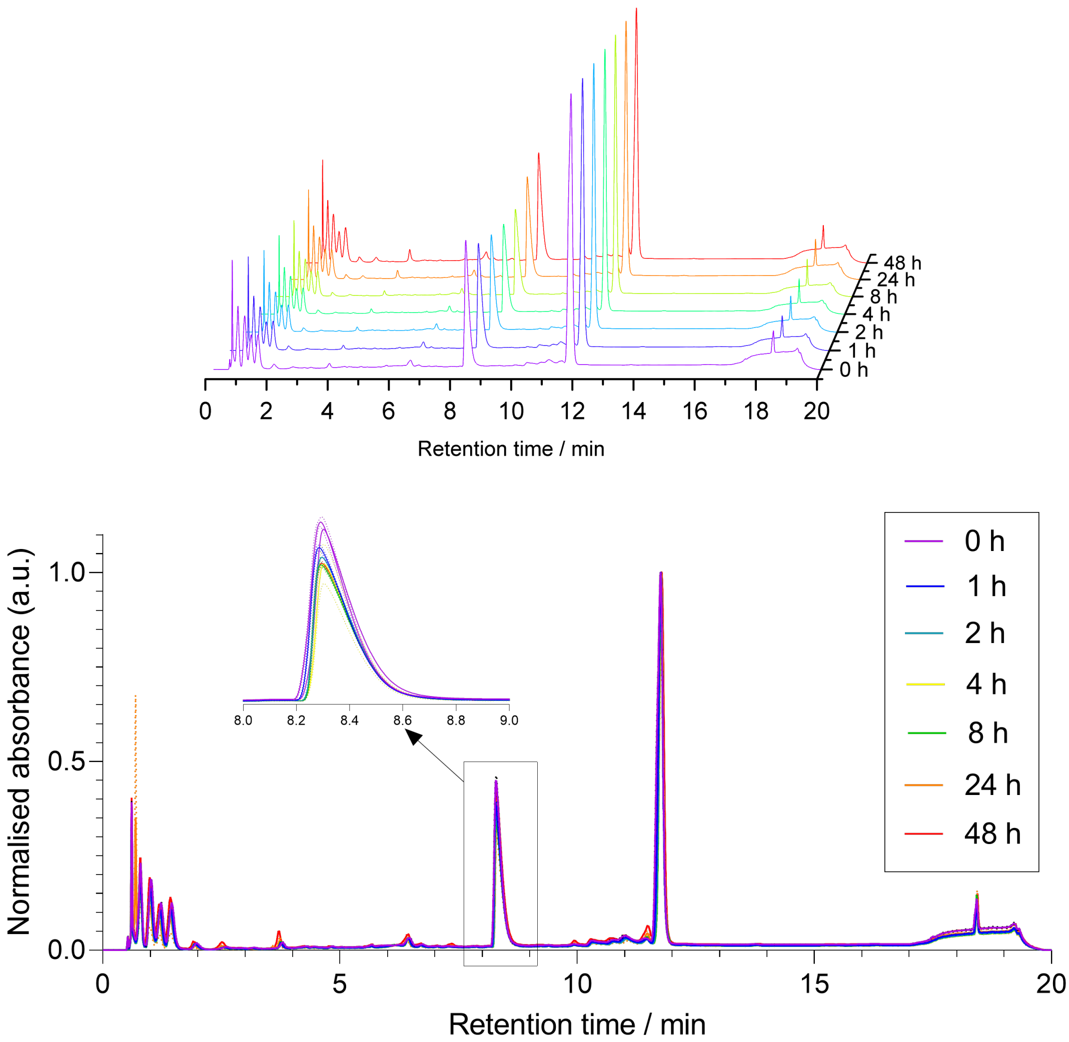

Figure 27. Elimination of PAB-BL 9 at pH 7.4, 37 °C. Hydrolysis of 2-phenyl acetamide group occurred rapidly upon enzyme addition and allows analysis of the rate of fragmentation of intermediate PAB-BL **9**. At pH 7.4, PAB-BL **9** does not appear to fragment. Retention times: β-lapachone **1** (11.5 min) PAB-BL **9** (8.3 min). Spectra are normalised to warfarin internal standard (11.8 min). Note consumption of 2-phenylacetyl-PAB-BL (14.3 min) **22** even in the first timepoint t = 0, taken < 5 min after amidase enzyme addition. Absorbance measured at 254 nm. Displayed spectra are the averaged trace of three independent reactions with standard error of the mean plotted as a dotted line. These results were replicated in the pH-dependent kinetic study described in **Supplementary Figure 35**.

Control experiment without addition of amidase to 2-phenylacetyl-PAB-BL 22, monitoring stability of 22 to pH 7.4, 37 °C

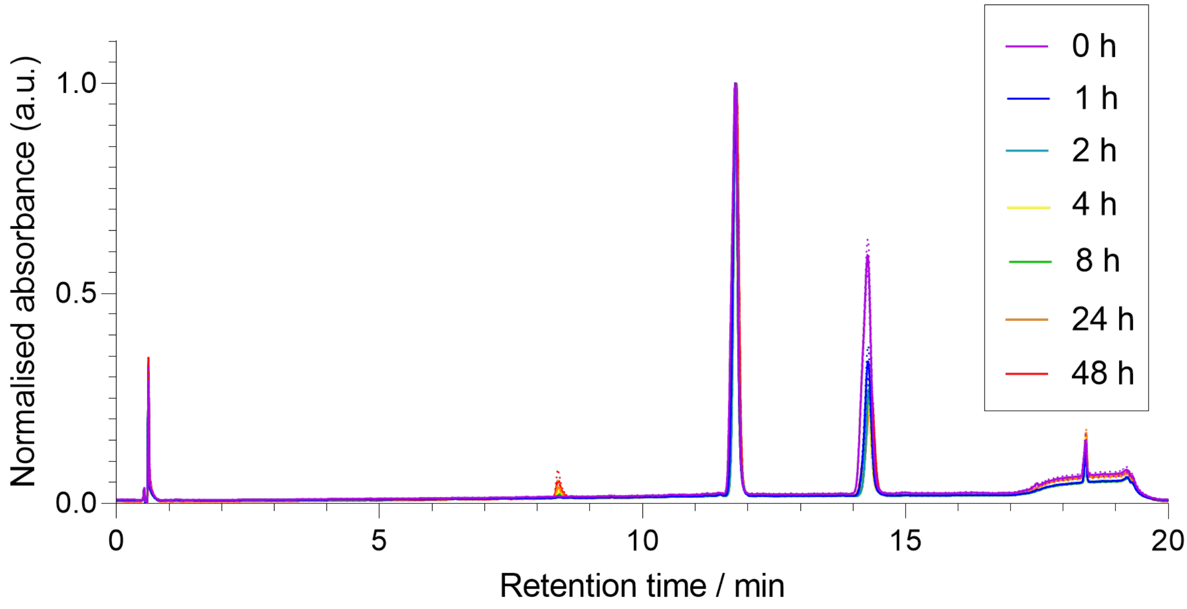

Figure 28. 2-phenylacetyl-PAB-BL 22 incubated in PBS, pH 7.4 at 37 °C without Penicillin-G amidase. Retention times: β-lapachone (11.5 min) PAB-BL **10** (8.3 min), 2-phenylacetyl-PAB-BL **22** (14.3 min). Spectra are normalised to warfarin internal standard (11.8 min). Absorbance measured at 254 nm. Displayed spectra are the averaged trace of three independent reactions with standard error of the mean plotted as a dotted line. Some degradation of compound **22** appears to occur.

Peak assignment

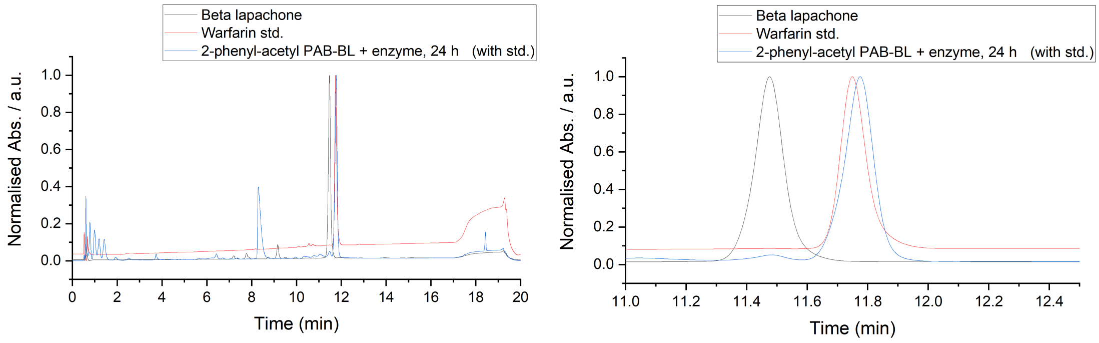

Figure 29. Assignment by overlay of released product from amidase assay with 2-phenylacetyl-PAB-BL 22 with β-lapachone 1 standard.

Kinetics of release of dunnione 12 from 2-phenylacetyl-PAB-DN 23 upon amidase trigger at pH 7.4

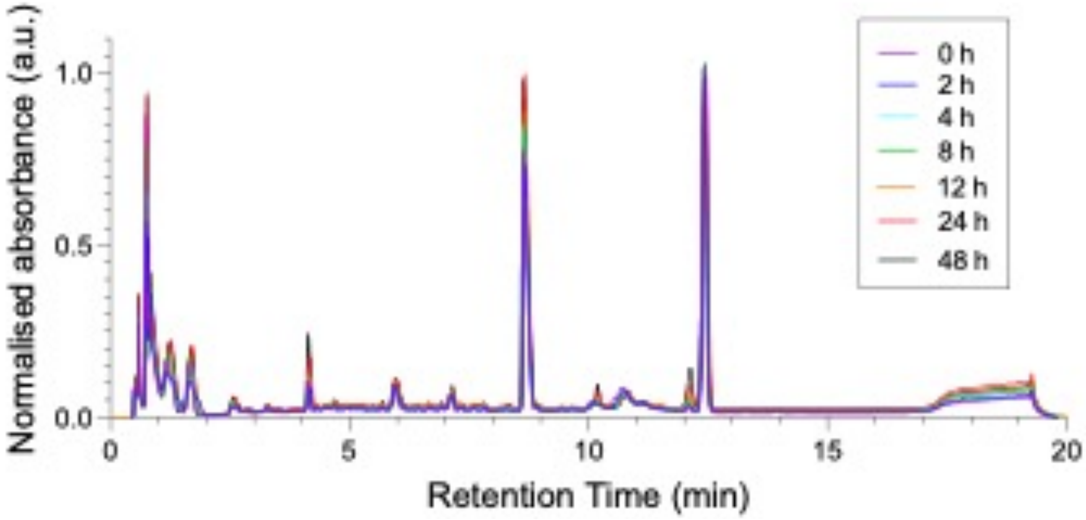

Figure 30. **Elimination of PAB-DN 18 at pH 7.4, 37ºC** Absorbance was measured at 254 nm and spectra are normalised to standard (warfarin, 12.4 min). Displayed spectra are the averaged 2 to 3 of three independent reactions. PAB-DN **18** was formed *in situ* by addition of Penicillin-G amidase to 2-phenylacetyl-PAB-DN **23**, and incubation for 15 min at 37 ºC prior to the t_0 HPLC timepoint. Experiment shows the elimination of PAB-DN (8.6 min) is slow at pH 7.4 with minimal dunnione (DN, 12.2 min) visible at 48 h. These results were replicated in the pH-dependent kinetic study described in **Supplementary Figure 37.**

Control experiment without addition of amidase to 2-phenylacetyl-PAB-DN 23, monitoring stability of 23 to pH 7.4, 37 °C.

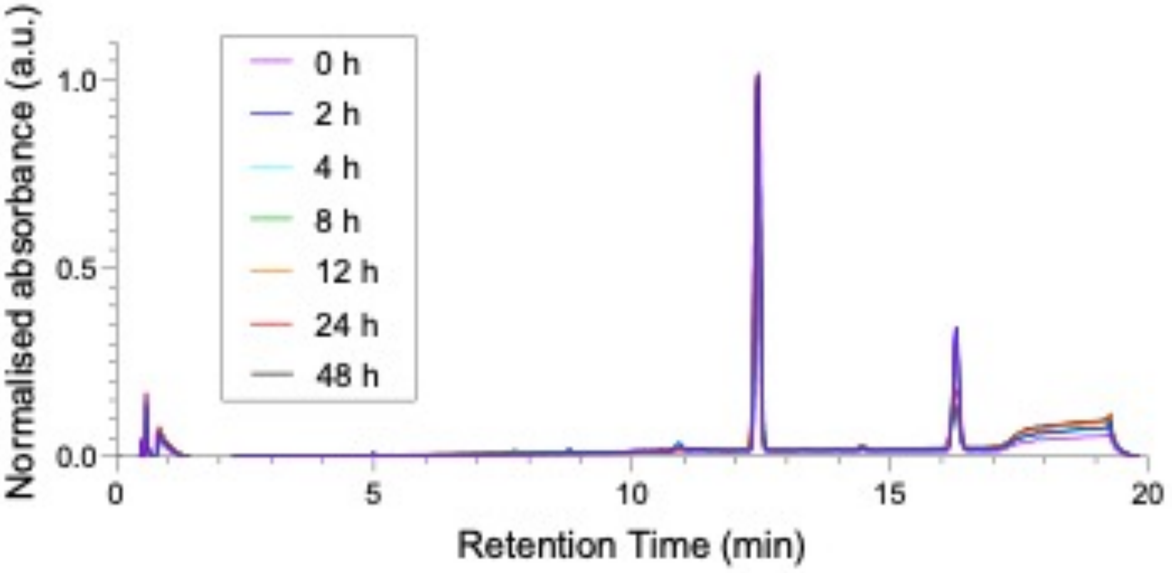

Figure 31. 2-Phenylacetyl-PAB-DN 23 (16.3 min) incubated in PBS, pH 7.4 at 37ºC without Penicillin G amidase. Spectra are normalised to warfarin internal standard (12.4 min). Absorbance measured at 254 nm. Displayed spectra are the averaged trace of three independent reactions with standard error of the mean plotted as a dotted line. Some degradation of compound **23** appears to occur.

Peak assignment

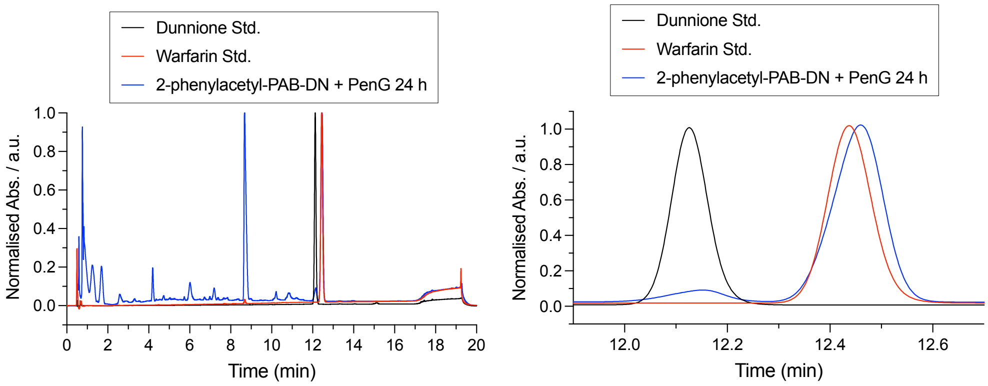

Figure 32. Example spectrum showing elimination product, overlayed with DN 12 (black line, 12.2 min). Sample contains warfarin internal standard (red line, 12.4 min).

pH dependence of kinetics of formation of quinone PhQ 4 from PAB-PhQ 7

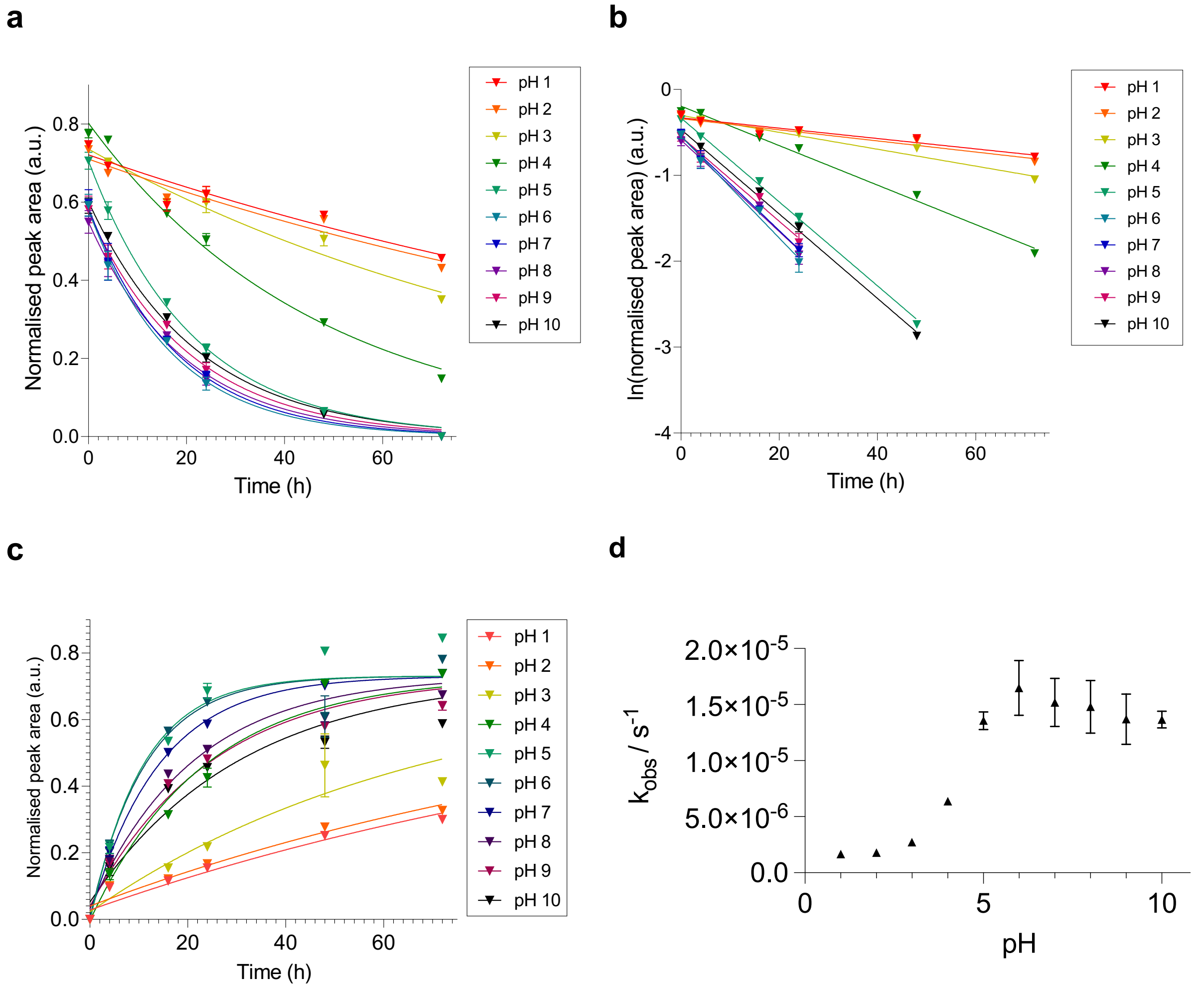

e

pH	k_{obs} / s^{-1}	95 % CI
**1**	1.68 × 10^{-6}	1.34 × 10^{-6} – 2.02 × 10^{-6}
**2**	1.80 × 10^{-6}	1.53 × 10^{-6} – 2.06 × 10^{-6}
**3**	2.73 × 10^{-6}	2.40 × 10^{-6} – 3.07 × 10^{-6}
**4**	6.38 × 10^{-6}	6.08 × 10^{-6} – 6.69 × 10^{-6}
**5**	1.36 × 10^{-5}	1.28 × 10^{-5} – 1.44 × 10^{-5}
**6**	1.65 × 10^{-5}	1.40 × 10^{-5} – 1.89 × 10^{-5}
**7**	1.52 × 10^{-5}	1.30 × 10^{-5} – 1.73 × 10^{-5}
**8**	1.48 × 10^{-5}	1.25 × 10^{-5} – 1.72 × 10^{-5}
**9**	1.37 × 10^{-5}	1.15 × 10^{-5}– 1.59 × 10^{-5}
**10**	1.37 × 10^{-5}	1.29 × 10^{-5} – 1.44× 10^{-5}

Figure 33. pH dependence of PAB-PhQ elimination. Normalised HPLC peak areas are mean of n=3 independent samples tested in one experiment. **a,** Normalised area of PAB-PhQ **7** peak with fitting of an exponential one-phase decay model, with a shared zero plateau. **b,** Linearised fit of PAB-PhQ **7** peak area. **c,** Normalised area of PhQ **4** peak with fitting of an exponential one-phase association model, with plateau a shared value. **d,** pH dependence of estimated k_{obs} of elimination, measured via linearised fit of normalised PAB-PhQ **7** peak area in **b**, error bar 95% CI of k_{obs} value. **e,** Numerical values in **d**.

HPLC traces PAB-PhQ 7 fragmentation

a

pH 1

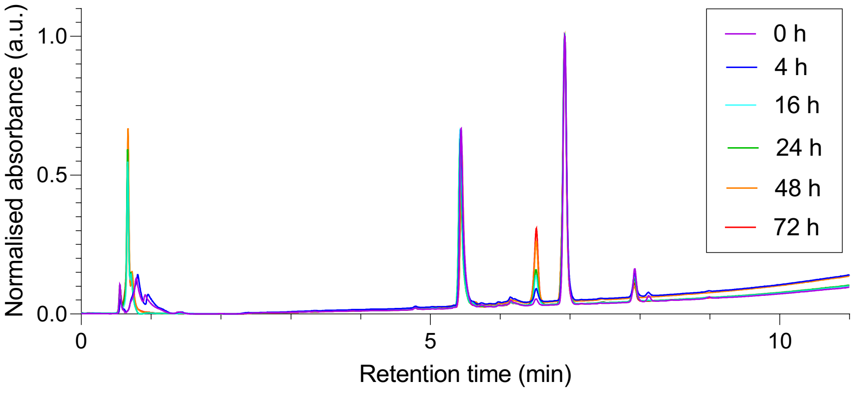

pH 2

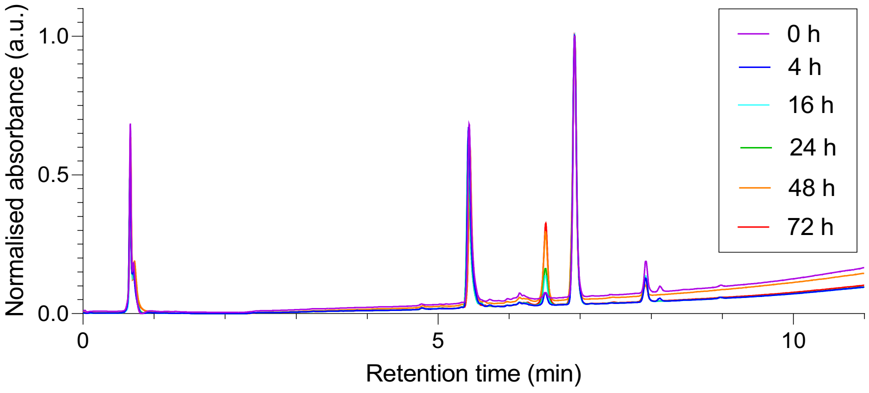

pH 3

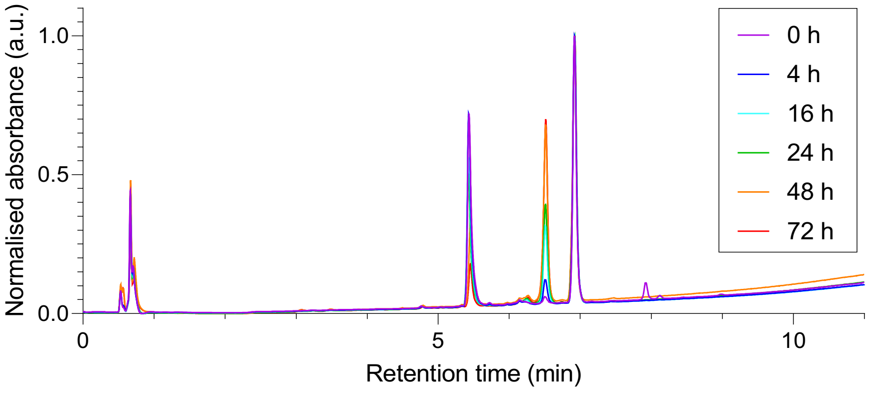

pH 4

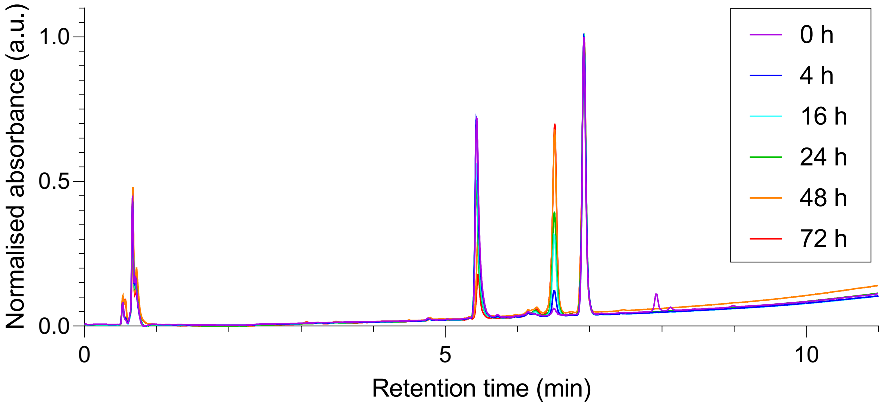

pH 5

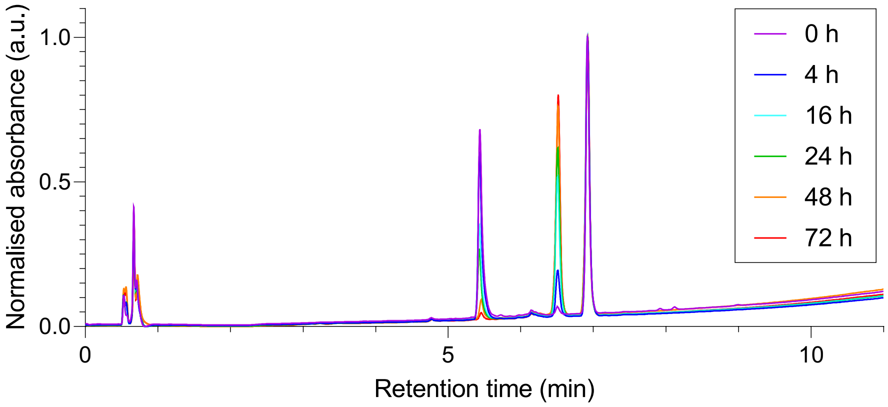

pH 6

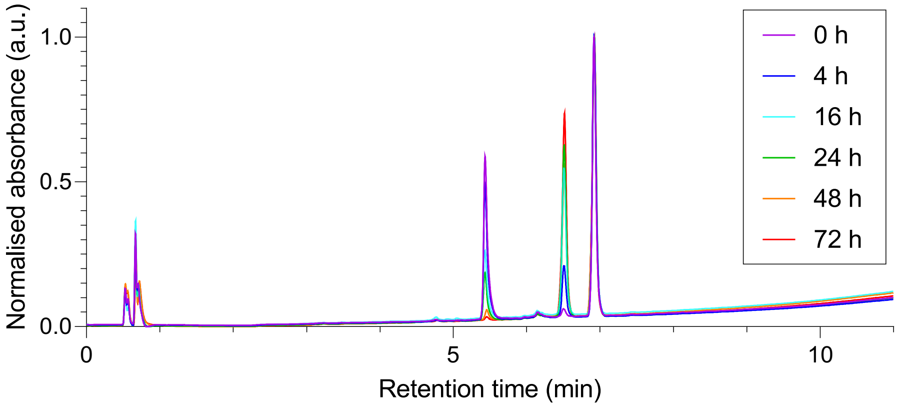

pH 7

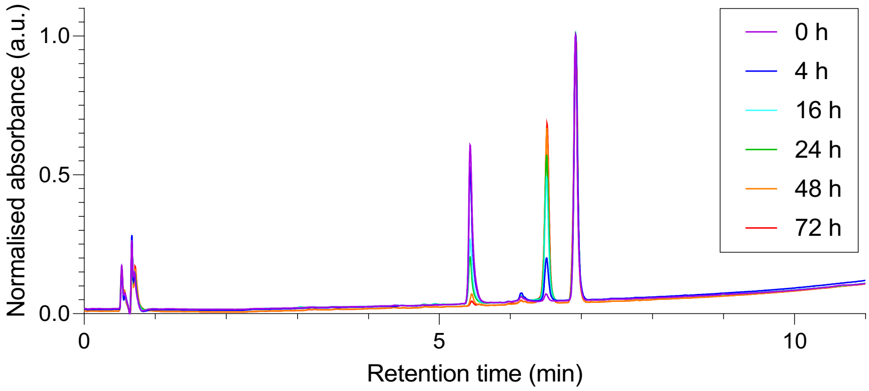

pH 8

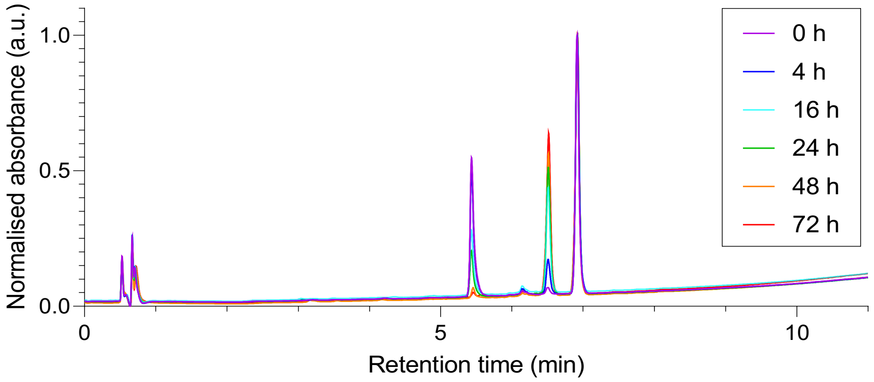

pH 9

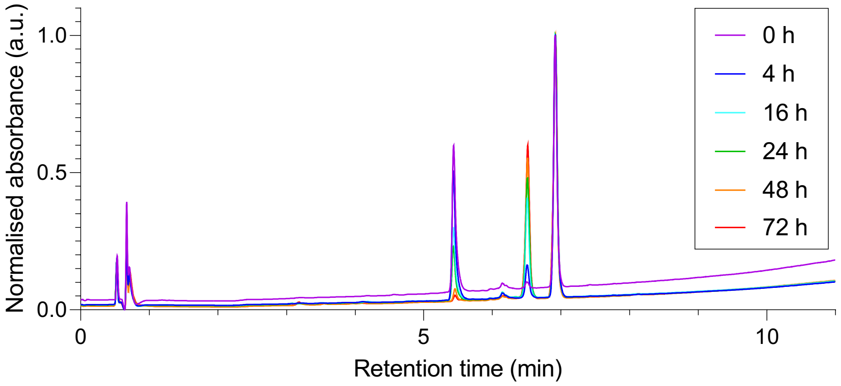

pH 10

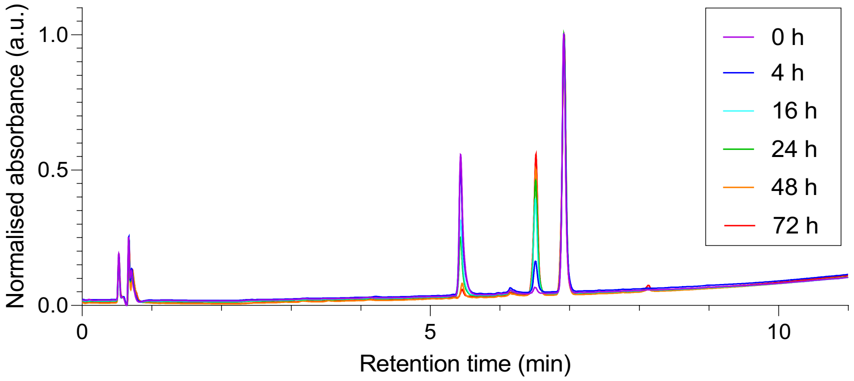

b

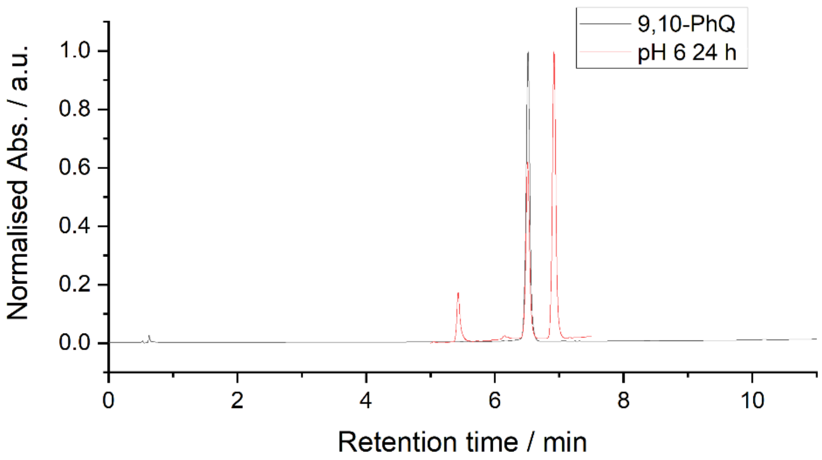

Figure 34. Normalised HPLC traces for rate vs pH experiment for elimination of PAB-PhQ 7 to release PhQ 4 . a, Spectra show elimination of PAB-PhQ **7** (5.4 min) leading to formation of PhQ **4** (6.5 min), normalised to the warfarin IS (6.9 min). **b,** Assignment of PhQ peak by overlay with a standard (black line, 6.5 min).

pH dependence of kinetics of formation of β-lapachone 1 from PAB-BL 10

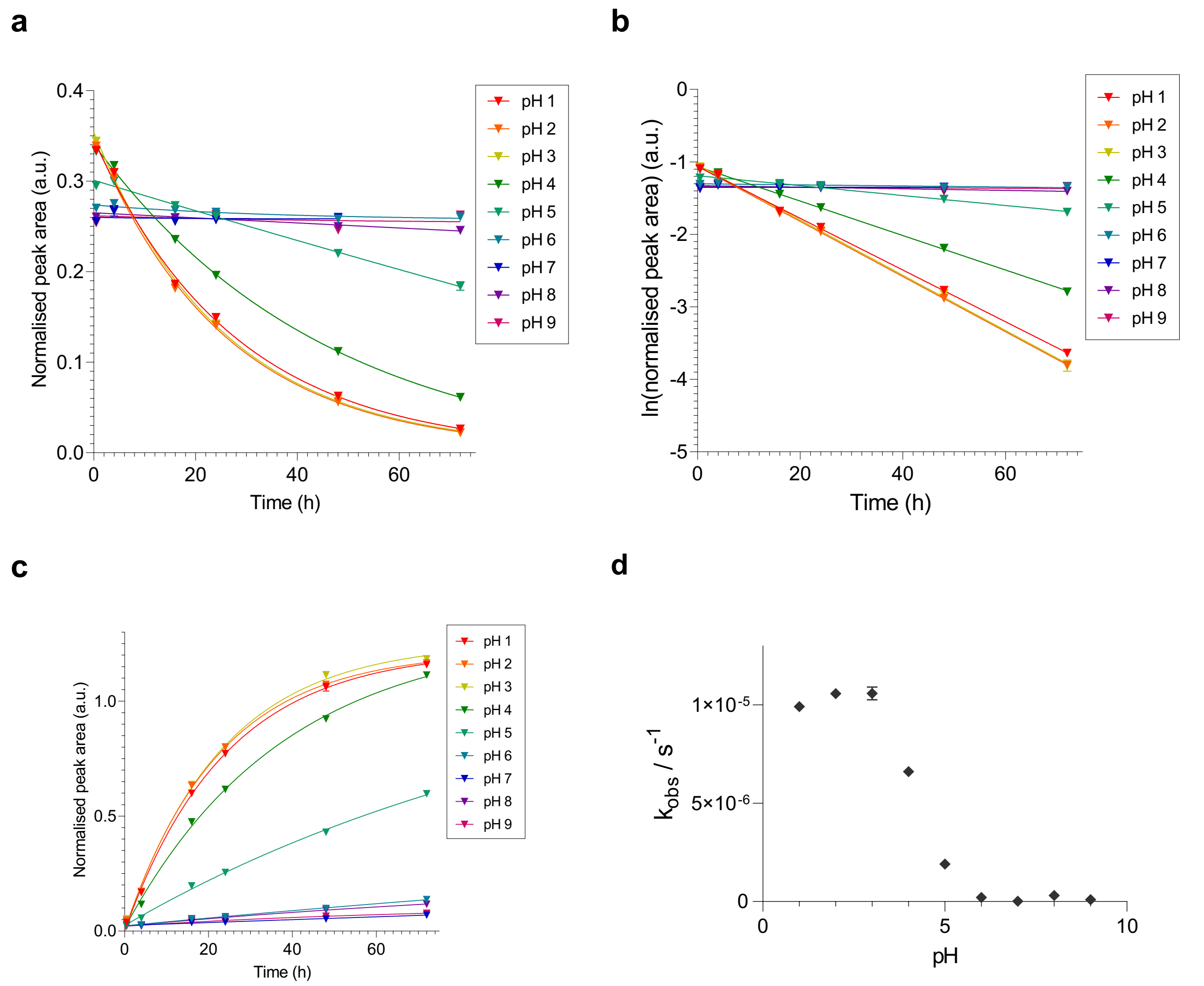

e

pH	k_{obs} / s^{-1}	95 % CI
**1**	9.92×10^{-6}	$9.72 \times 10^{-6} – 1.01 \times 10^{-5}$
**2**	1.06×10^{-5}	$1.04 \times 10^{-5} – 1.07 \times 10^{-5}$
**3**	1.06×10^{-5}	$1.03 \times 10^{-5} – 1.09 \times 10^{-5}$
**4**	6.61×10^{-6}	$6.49 \times 10^{-6} – 6.73 \times 10^{-6}$
**5**	1.90×10^{-6}	$1.77 \times 10^{-6} – 2.04 \times 10^{-6}$
**6**	2.07×10^{-7}	$1.05 \times 10^{-7} – 3.09 \times 10^{-7}$
**7**	1.41×10^{-8}	$0.00 – 2.44 \times 10^{-7}$
**8**	3.07×10^{-7}	$2.00 \times 10^{-7} – 4.06 \times 10^{-7}$
**9**	9.33×10^{-8}	$0.00 – 2.81 \times 10^{-7}$

Figure 35. pH dependence of PAB-BL elimination. Normalised HPLC peak areas are mean of n=3 independent samples tested in one experiment. **a,** Normalised area of PAB-BL **10** peak with fitting of an exponential one-phase decay model, with a shared zero plateau. **b,** Linearised fit of PAB-BL **10** peak. **c,** Normalised area of BL **1** peak with fitting of an exponential one-phase association model, with plateau a shared value. **d,** pH dependence of estimated k_{obs} of elimination, measured via linearised fit of normalised PAB-BL **10** peak area in **b**, error bar 95% CI of k_{obs} value. **e,** Numerical values in **d**.

HPLC traces PAB-BL 10 fragmentation

a

pH 1

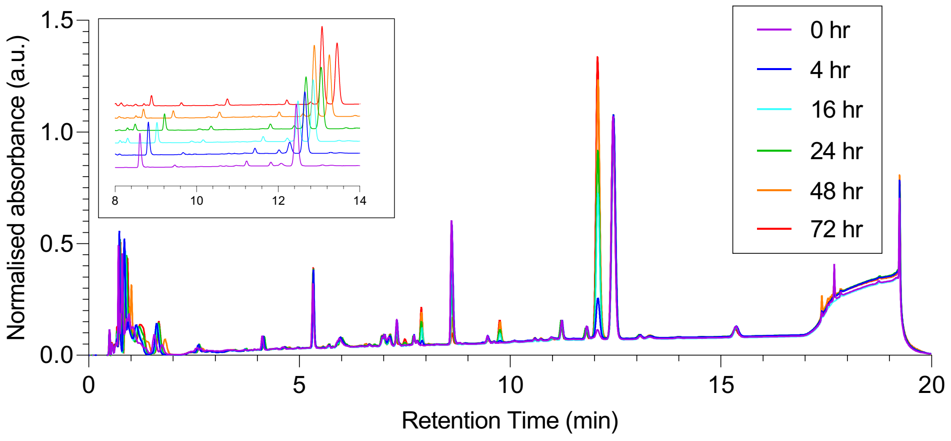

pH 2

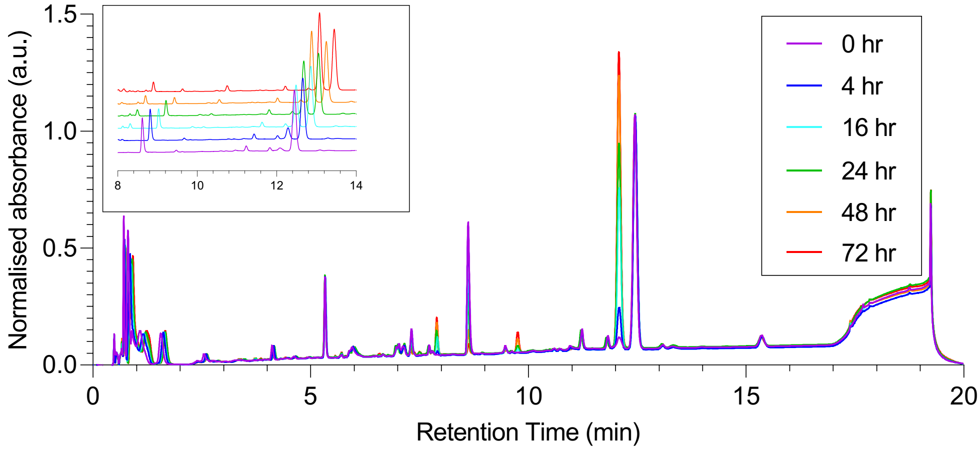

pH 3

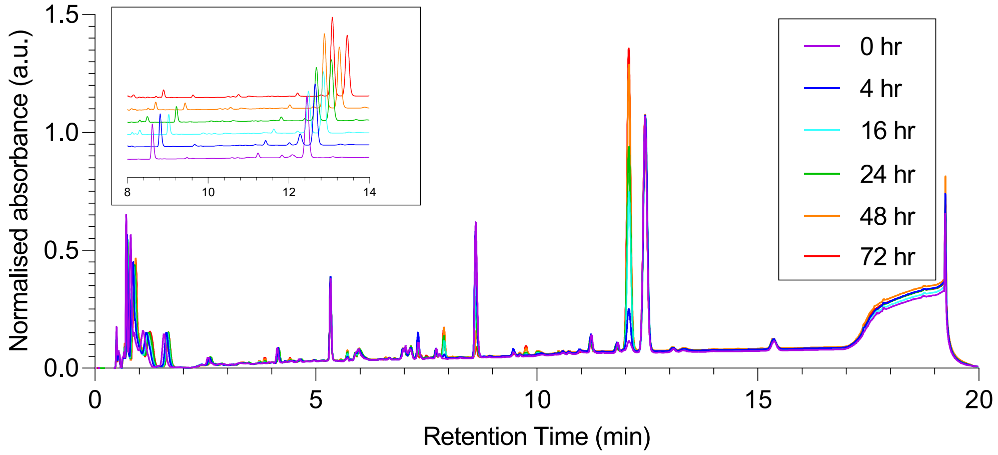

pH 4

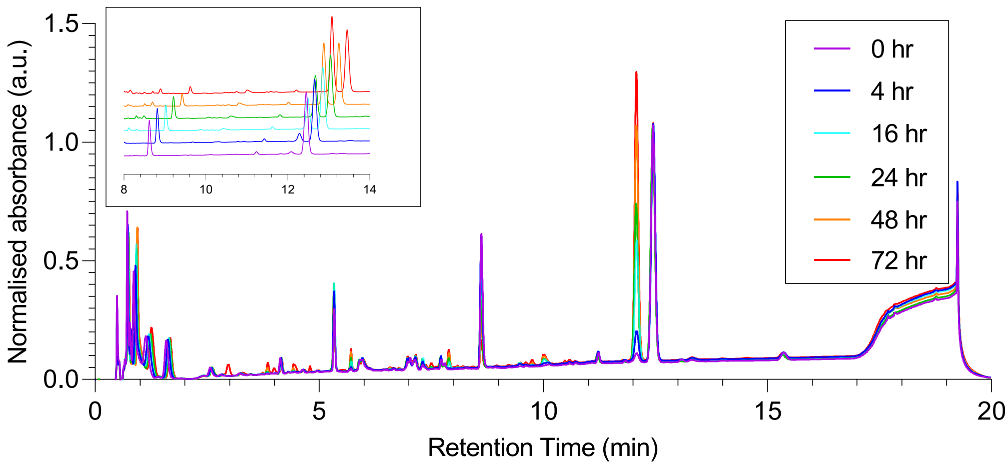
Normalised absorbance (a.u.)
Retention Time (min)
0 hr
4 hr
16 hr
24 hr
48 hr
72 hr

pH 5

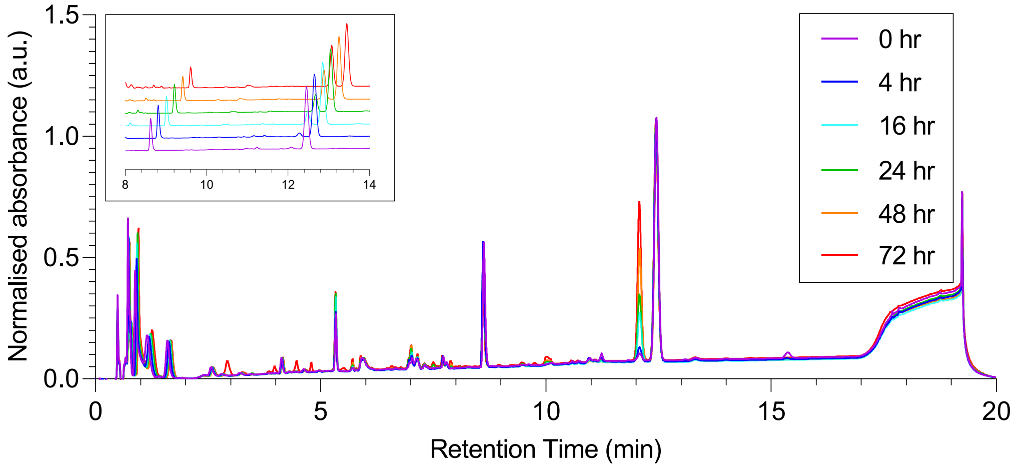
Normalised absorbance (a.u.)
Retention Time (min)
0 hr
4 hr
16 hr
24 hr
48 hr
72 hr

pH 6

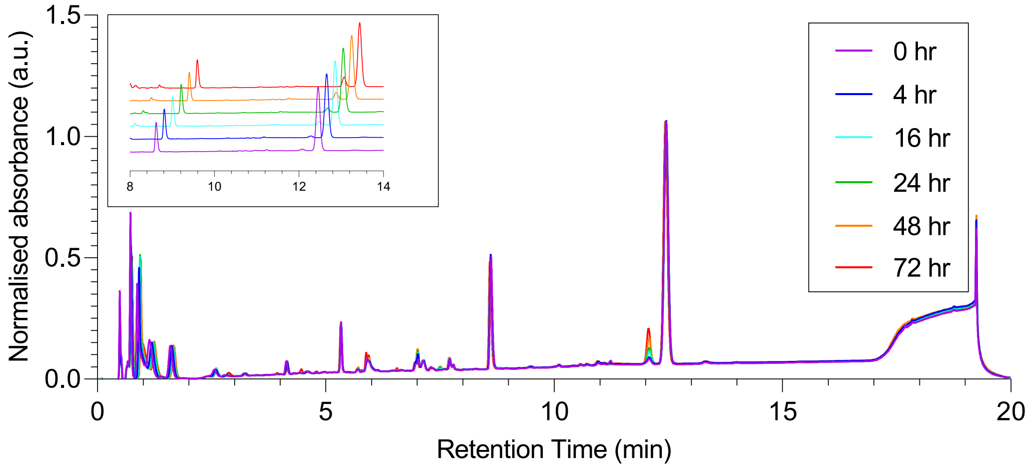
Normalised absorbance (a.u.)
Retention Time (min)
0 hr
4 hr
16 hr
24 hr
48 hr
72 hr

pH 7

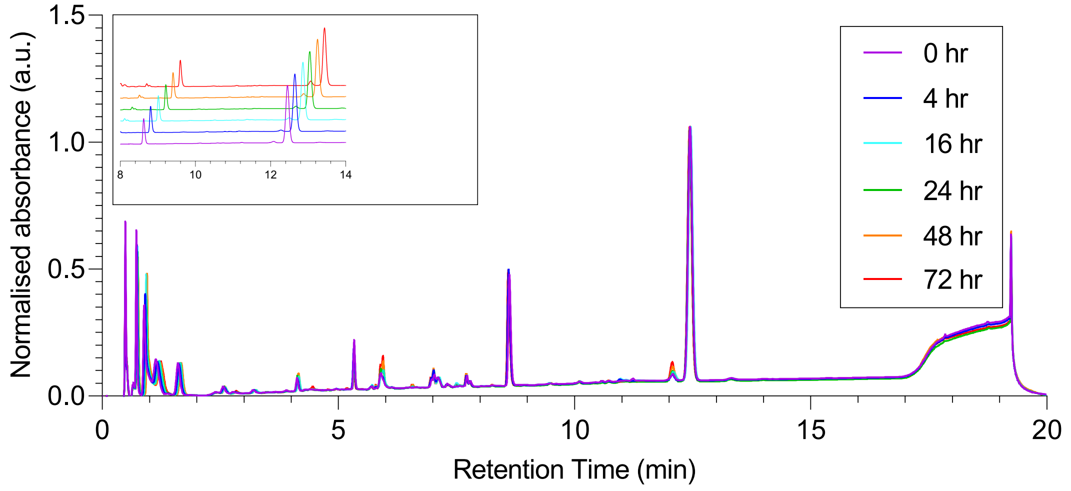

Normalised absorbance (a.u.)
Retention Time (min)
0 hr
4 hr
16 hr
24 hr
48 hr
72 hr

pH 8

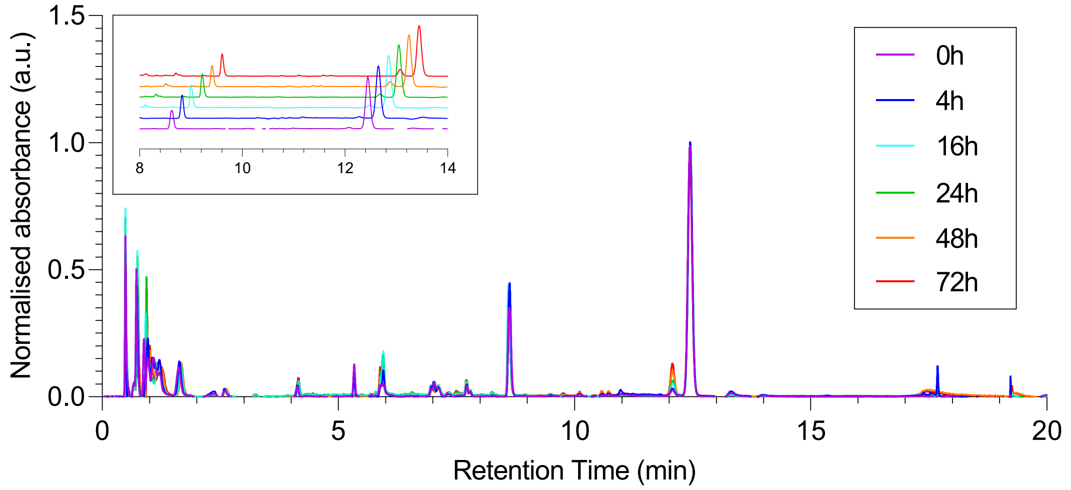

Normalised absorbance (a.u.)
Retention Time (min)
0h
4h
16h
24h
48h
72h

pH 9

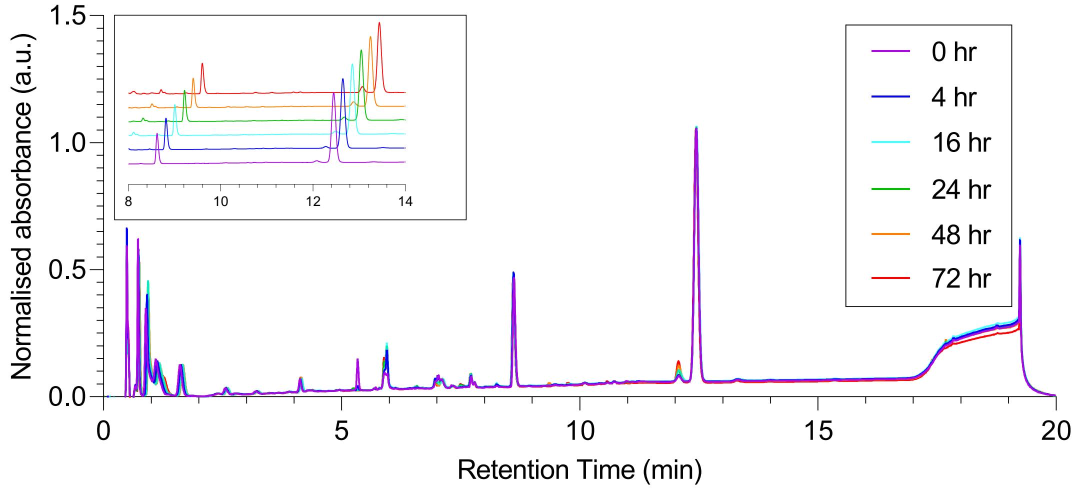

Normalised absorbance (a.u.)
Retention Time (min)
0 hr
4 hr
16 hr
24 hr
48 hr
72 hr

b

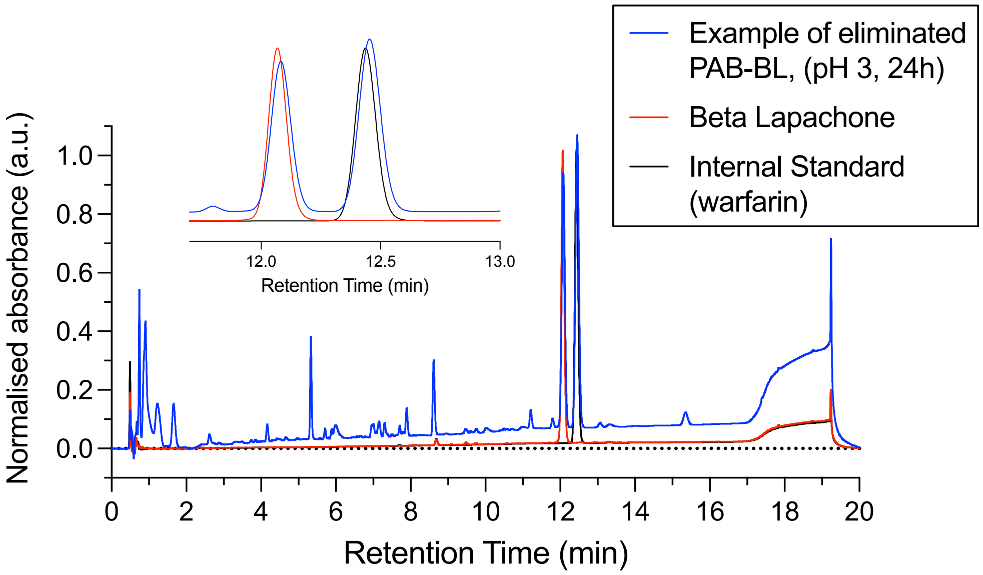

Figure 36. Normalised HPLC traces for rate vs pH experiment for elimination of PAB-BL 10 to release β-lapachone (BL). a, Spectra show formation of β-lapachone **1** (12.1 min) following consumption of PAB-BL **10** (8.6 min) Spectra are normalised to warfarin internal standard (12.4 min). **b,** Assignment of BL peak by overlay with a standard.

pH dependence of kinetics of formation of Dunnione 12 from PAB-DN 18

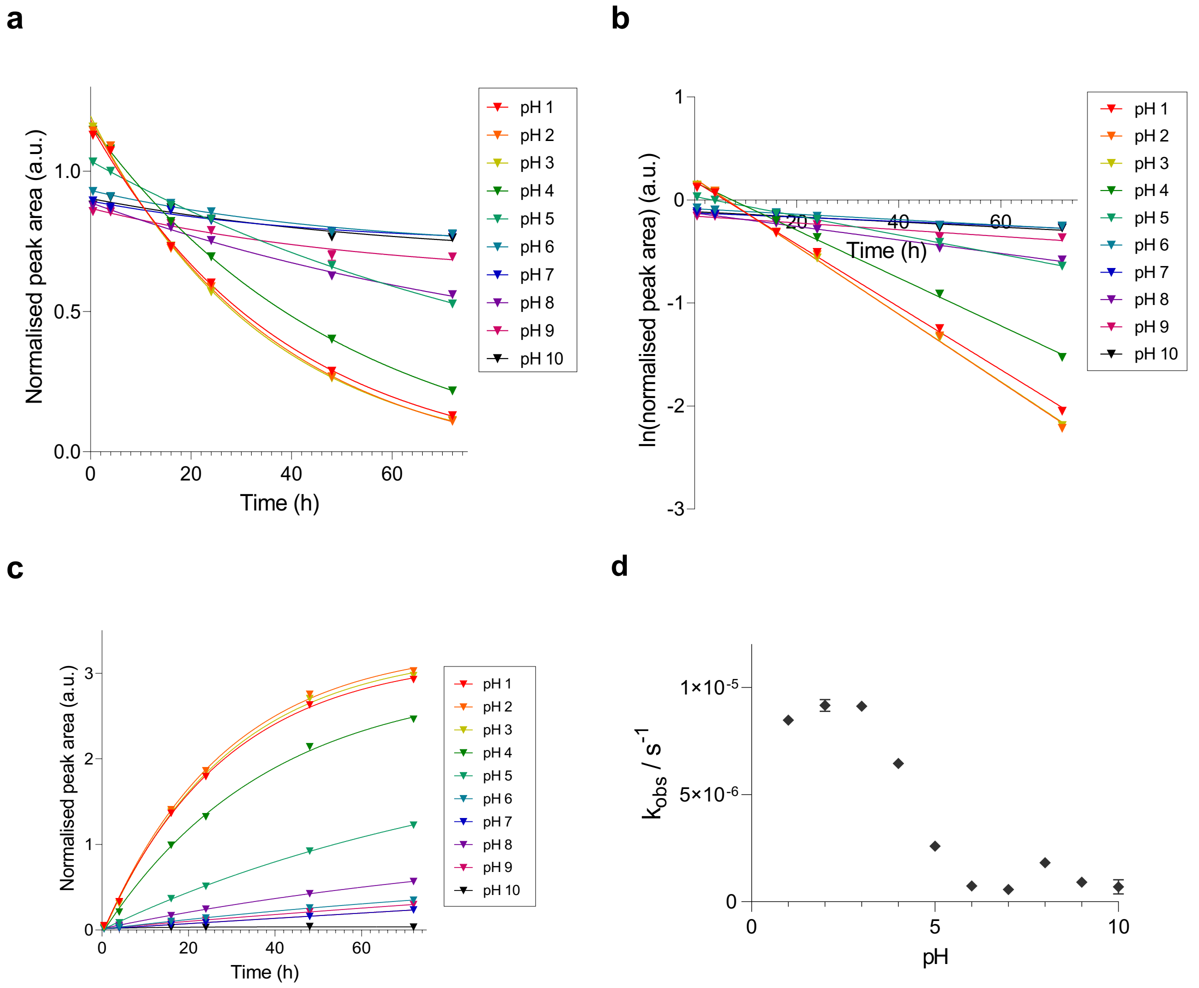

e

pH	k_{obs} / s^{-1}	95 % CI
**1**	8.47×10^{-6}	$8.26 \times 10^{-6} – 8.68 \times 10^{-6}$
**2**	9.16×10^{-6}	$8.88 \times 10^{-6} – 9.43 \times 10^{-6}$
**3**	9.12×10^{-6}	$8.93 \times 10^{-6} – 9.30 \times 10^{-6}$
**4**	6.44×10^{-6}	$6.30 \times 10^{-6} – 6.59 \times 10^{-6}$
**5**	2.60×10^{-6}	$2.52 \times 10^{-6} – 2.68 \times 10^{-6}$
**6**	7.35×10^{-7}	$6.25 \times 10^{-7} – 8.46 \times 10^{-7}$
**7**	5.71×10^{-7}	$4.65 \times 10^{-7} – 6.78 \times 10^{-7}$
**8**	1.82×10^{-6}	$1.70 \times 10^{-6} – 1.93 \times 10^{-6}$
**9**	9.13×10^{-7}	$7.28 \times 10^{-7} – 1.10 \times 10^{-6}$
**10**	6.96×10^{-7}	$3.63 \times 10^{-7} – 1.03 \times 10^{-6}$

Figure 37. pH dependence of PAB-DN elimination. Normalised HPLC peak areas are mean of n=3 independent samples tested in one experiment. **a,** Normalised area of PAB-DN **18** peak with fitting of an exponential one-phase decay model, with a shared zero plateau. **b,** Linearised fit of PAB-DN **18** peak. **c,** Normalised area of DN **12** peak with fitting of an exponential one-phase association model, with plateau a shared value. **d,** pH dependence of estimated k_{obs} of elimination, measured via linearised fit of normalised PAB-DN **18** peak area in **b**, error bar 95% CI of k_{obs} value. **e,** Numerical values in **d**.

HPLC traces PAB-DN 12 fragmentation

a

pH 1

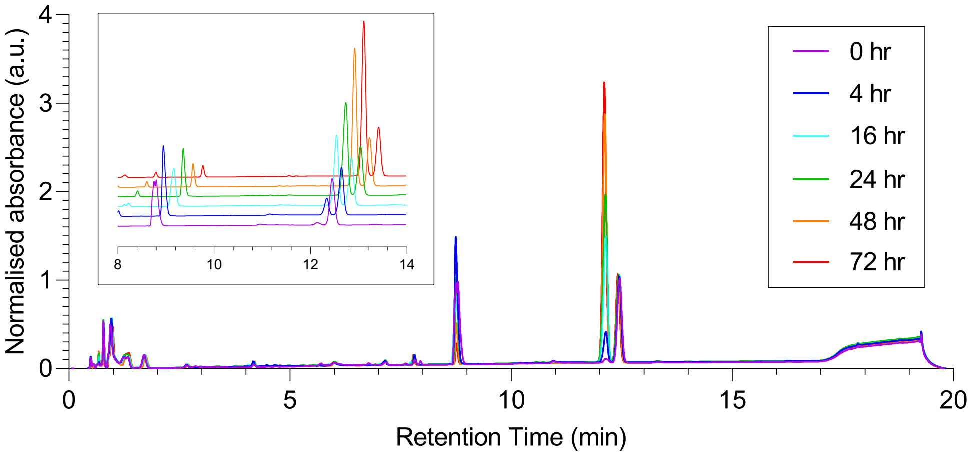

pH 2

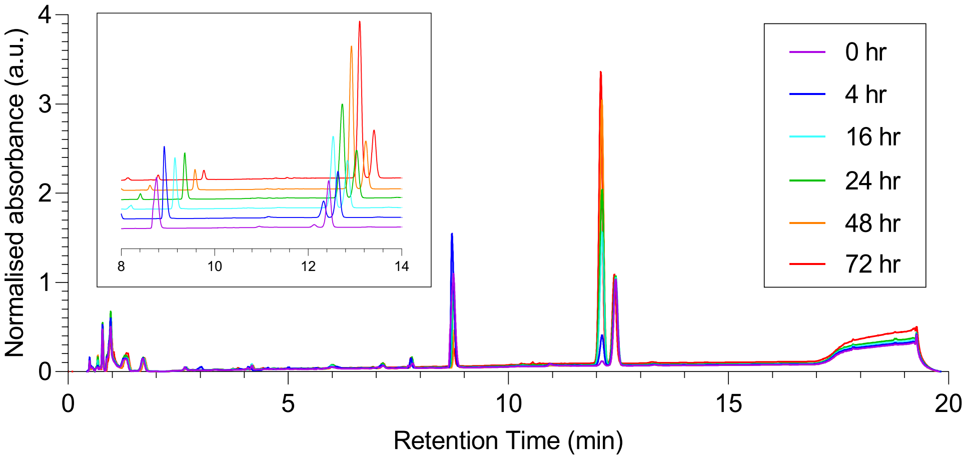

pH 3

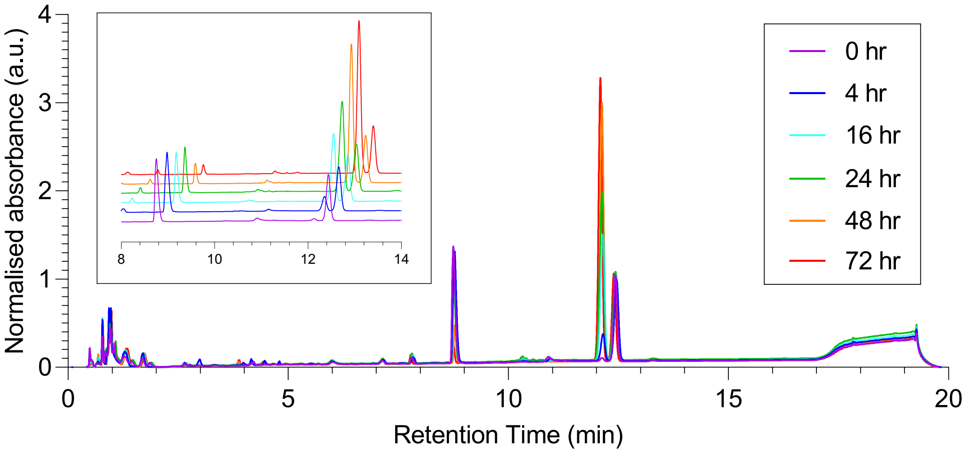

pH 4

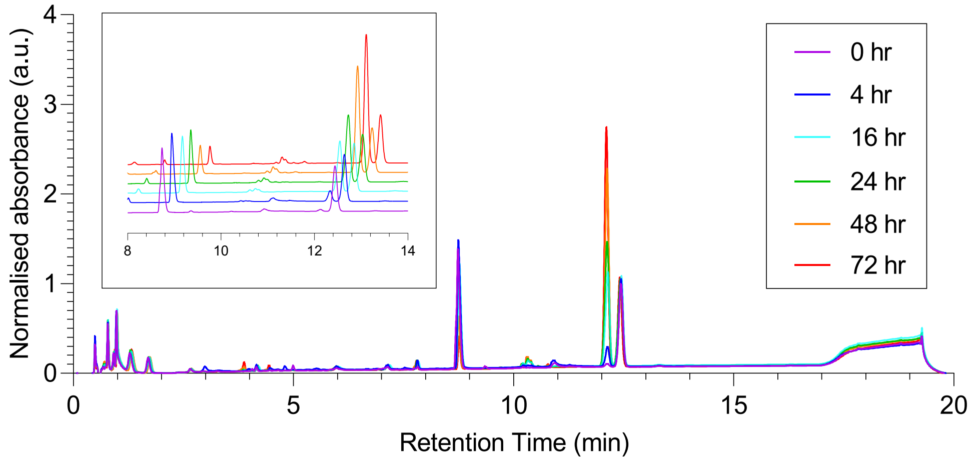

Normalised absorbance (a.u.)
Retention Time (min)
0 hr
4 hr
16 hr
24 hr
48 hr
72 hr

pH 5

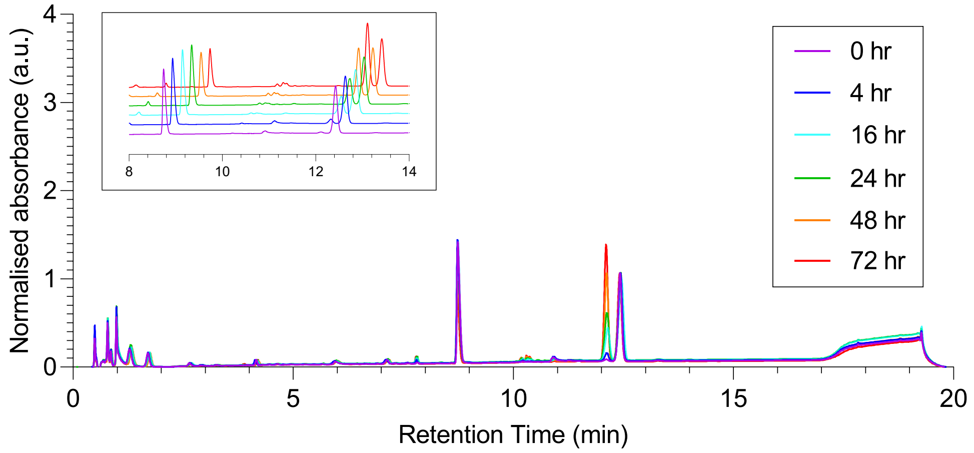

Normalised absorbance (a.u.)
Retention Time (min)
0 hr
4 hr
16 hr
24 hr
48 hr
72 hr

pH 6

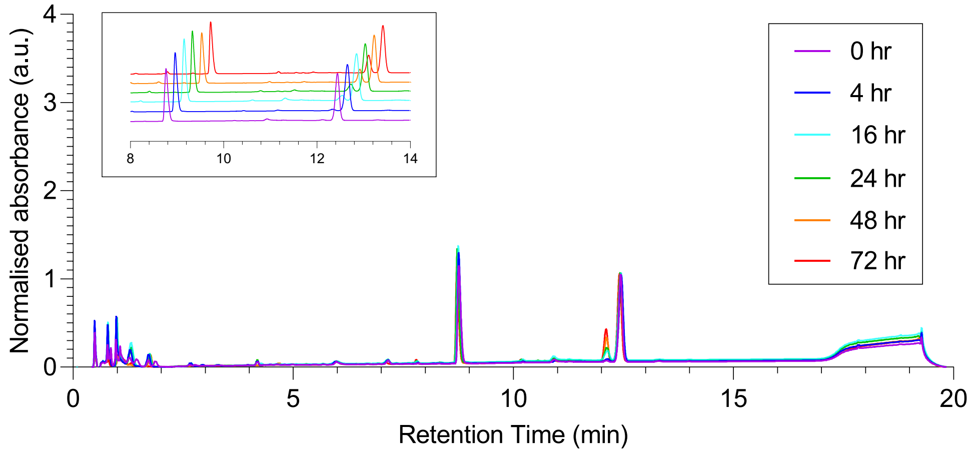

Normalised absorbance (a.u.)
Retention Time (min)
0 hr
4 hr
16 hr
24 hr
48 hr
72 hr

pH 7

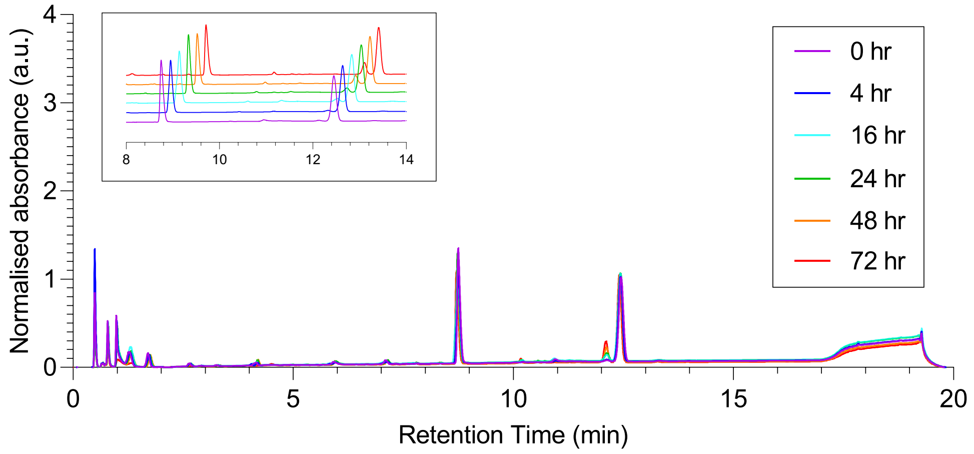

Normalised absorbance (a.u.)
Retention Time (min)
0 hr
4 hr
16 hr
24 hr
48 hr
72 hr

pH 8

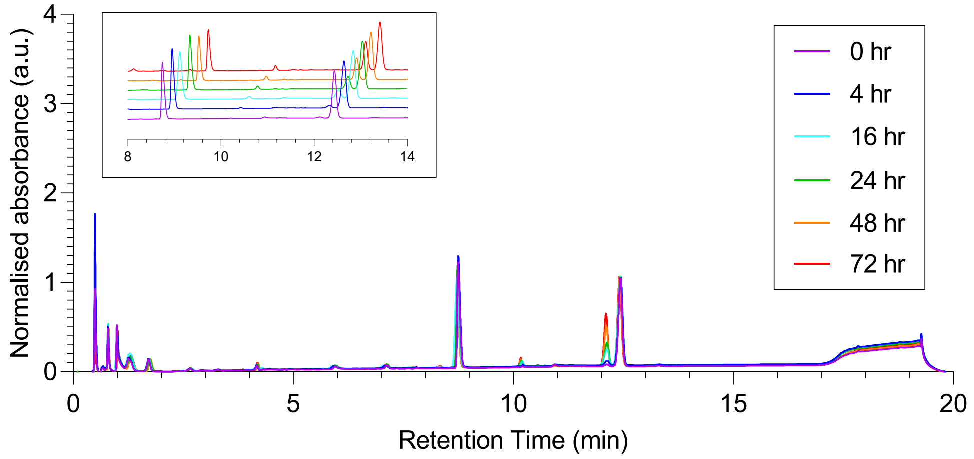

Normalised absorbance (a.u.)
Retention Time (min)
0 hr
4 hr
16 hr
24 hr
48 hr
72 hr

pH 9

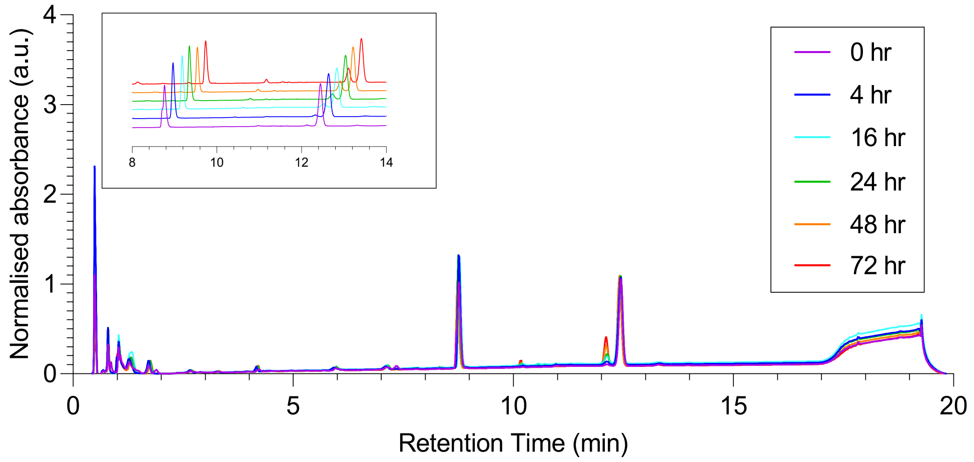

Normalised absorbance (a.u.)
Retention Time (min)
0 hr
4 hr
16 hr
24 hr
48 hr
72 hr

b

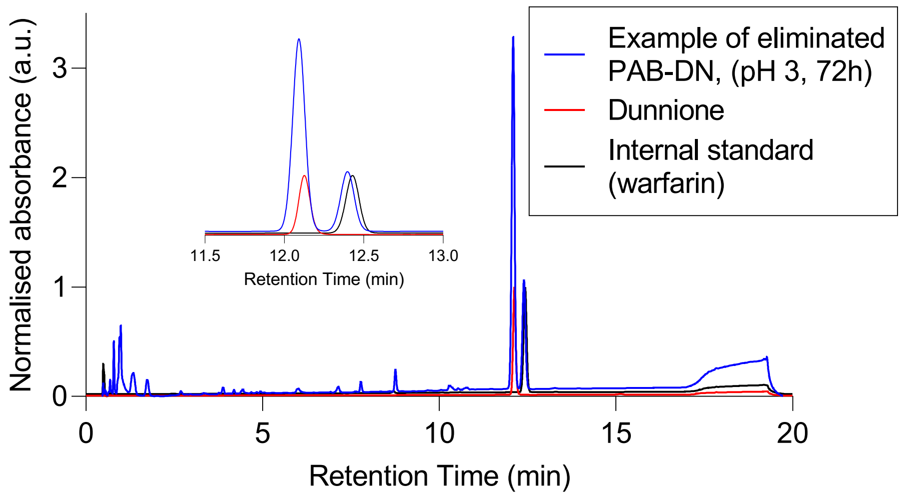

Figure 38. **a,** Normalised HPLC traces for rate versus pH experiment for elimination of PAB-DN **18** to release of DN **12**. **b,** Assignments by overlay with a DN **12** standard. Example spectrum showing elimination product, overlayed with DN **12** (red line, 12.2 min). Sample contains warfarin internal standard (black line, 12.4 min).

4. Assays with non-fragmentable benzyl β-lapa-ketol

In vitro redox-cycling ability

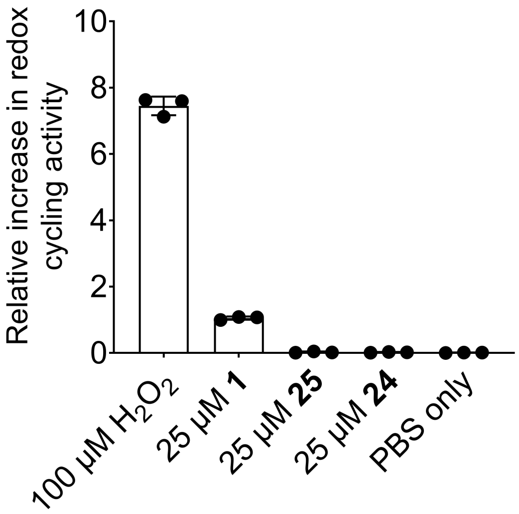

Figure 39. Relative *in vitro* redox cycling compared to H_2O_2 control. Data are presented as normalised mean values from one representative experiment ± SEM (n=3). This experiment was repeated once with similar results.

Methaemoglobin analysis

a

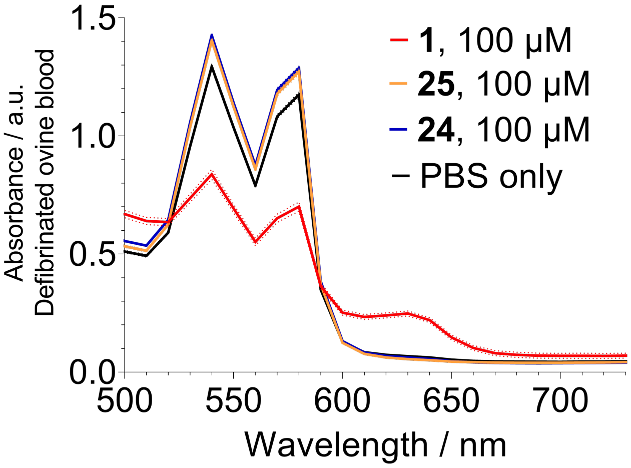

b

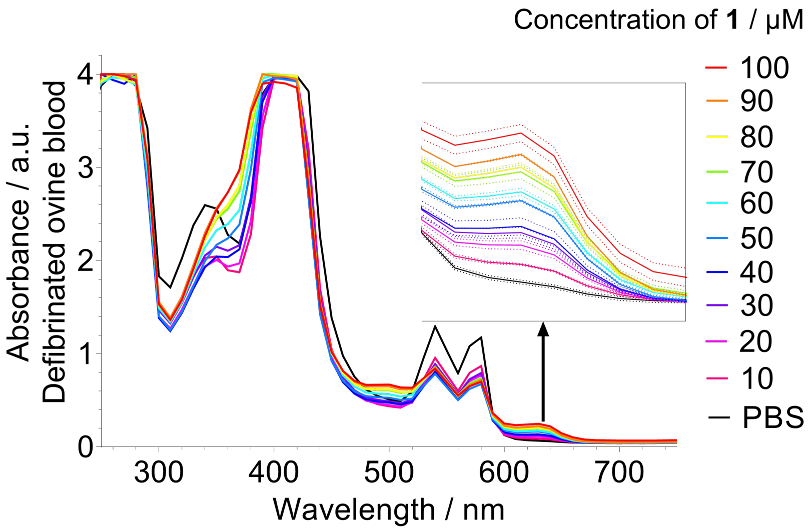

Figure 40. Methaemoglobin observed by UV/Vis after 1 h incubation of compounds with defibrinated ovine blood. a, Appearance of methaemoglobin peak at 630 nm for compounds at 100 µM concentration. **b,** Full UV spectrum showing methaemoglobin generation by increasing concentrations of **1** in ovine blood. Line represents mean values, with dotted borders representing ± SEM (n=3). This experiment was repeated once with similar results.

Haemolysis Analysis

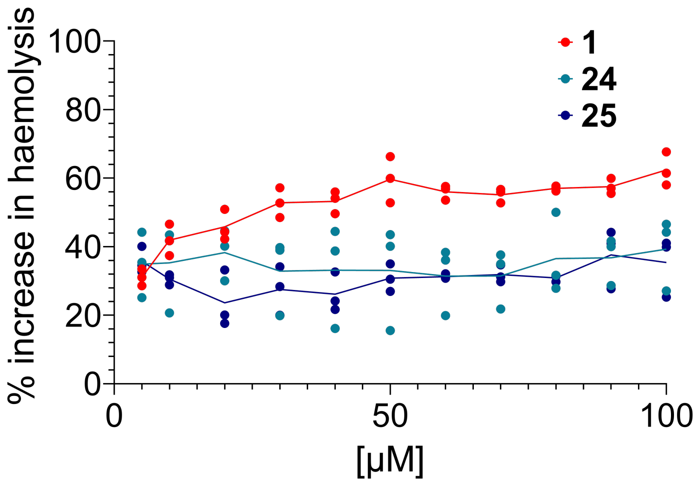

Figure 41**. Haemolysis of ovine blood by β-lapachone and derivatives. Haemolysis of ovine blood by β-lapachone and derivatives.** Line connects mean % increase in haemolysis at each tested concentration (n=3). This experiment was performed once, but methaemoglobin experiments described in ***Supplementary Figure 40*** also suggest haemolysis by changes in UV absorbance at 540 nm.

Cytotoxicity to cancer cell lines

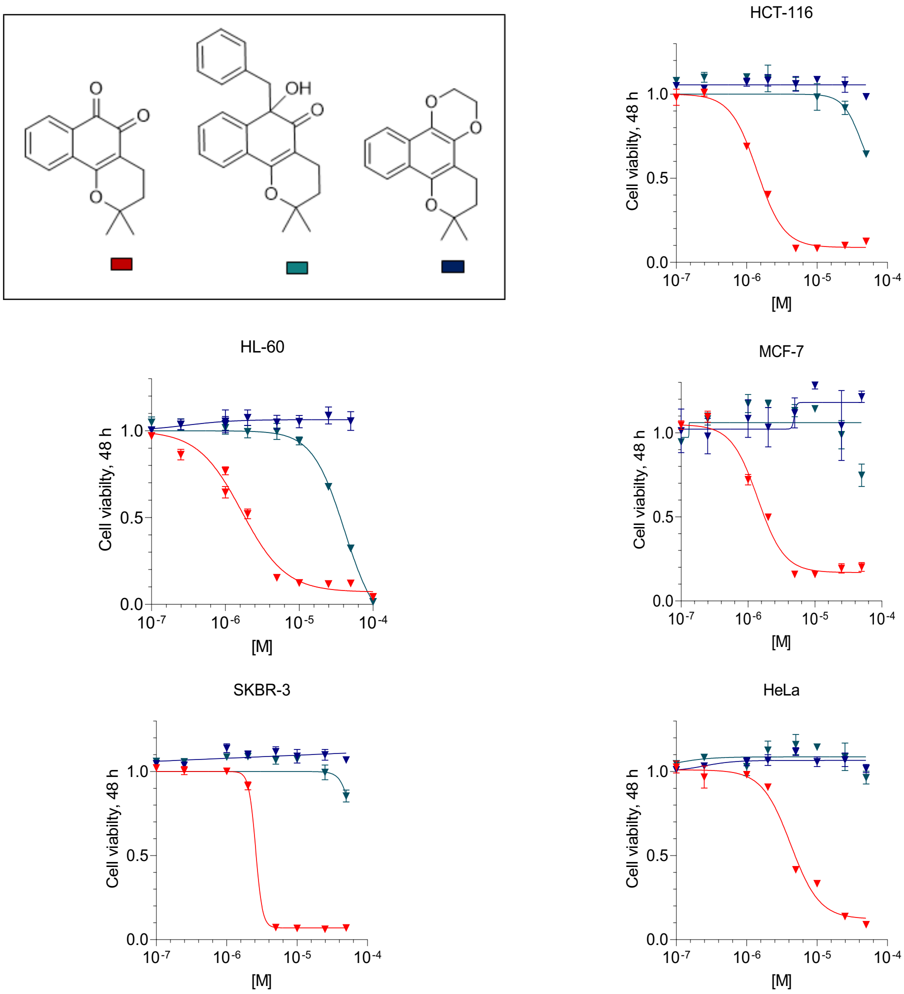

IC$_{50}$ / μM

	HCT-116	HL-60	SKBR3	MCF-7	HeLa
**1**	1.1 ± 0.5	1.4 ± 0.2	2.8 ± 0.4	1.9 ± 0.2	4.2 ± 0.9
**24**	> 40	32.4 ± 14.9	> 50	> 50	> 50
**25**	> 50	> 50	> 50	> 50	> 50

Figure 42. Cytotoxicity of β-lapachone and derivatives to cancer cell lines. Data are presented as mean values fit to 4-PL model with error bars representing ± SEM (n=3). These experiments were replicated once with similar results.

5. β-lapachone and AML

Cytotoxicity of β-lapachone to AML cell lines

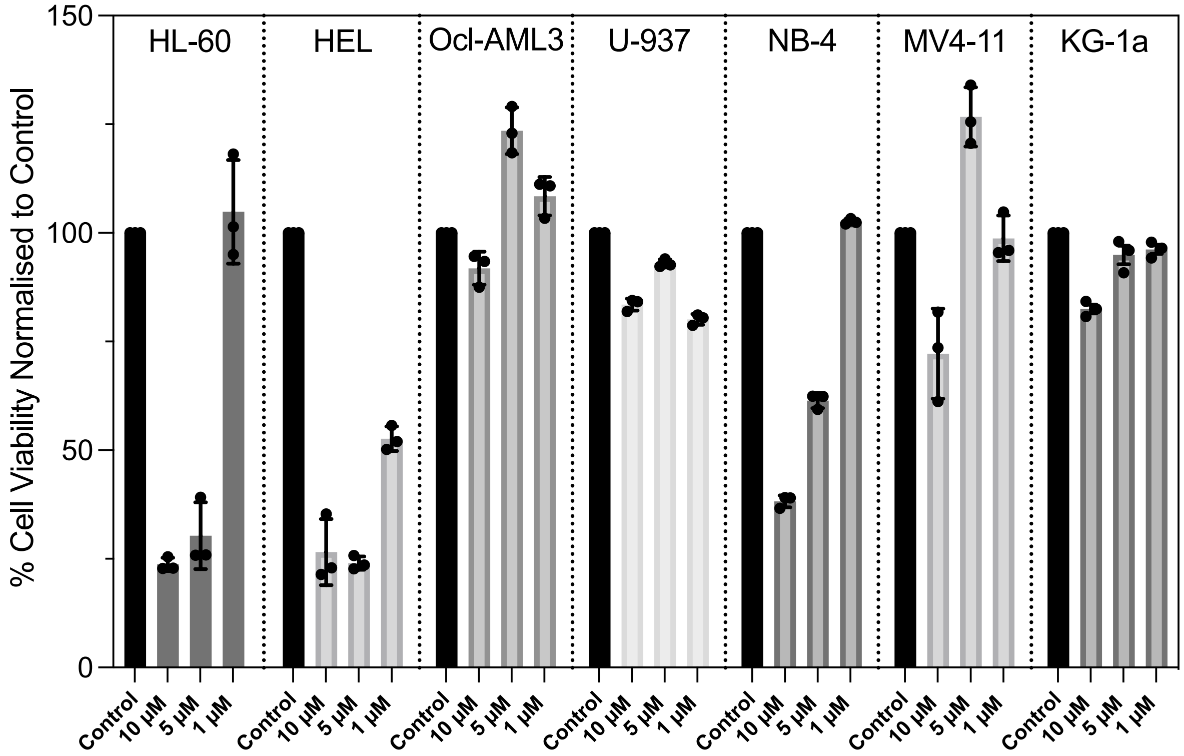

Figure 43. **Cell viability after treatment with β-lapachone, 1 measured by MTT assay. .** Data are presented as normalised mean values from one representative experiment ± SEM (n=3). This experiment was performed once.

CRISPR-Cas9 5-LO knockout (KO) in AML

The effect of CRISPR knockout of 5-LO on viability of HEL cells and their sensitivity to β-lapachone was tested.

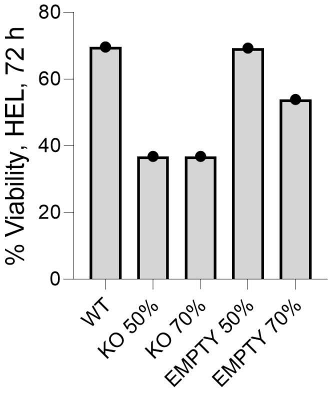

Figure 44. 5-LO knockout experiment using CRISPR-Cas9 KO in HEL. Viability of HEL cells after 72 h was lower upon active KO vector induction (50% or 70% vector at t = 0 h), suggesting decreased proliferation. Viability of cells containing the control vector was similar to wild-type for 50% vector samples or slightly lower than wild-type for 70 % vector samples. Viability of cells was assessed using FACS by propidium iodide staining. As β-lapachone triggers generalised necrosis viability of cells was conducted by analysis of propidium iodide-unstained cells as a % of total detected events. Data are n=1 and this experiment was performed once.

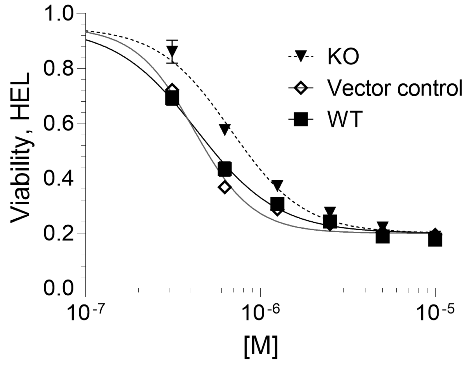

Figure 45. Viability of WT and 5-LO KO HEL upon treatment with β-lapachone, 1. HEL control or 5-LO–KO cells with 100 % gRNA vector expression were seeded in 96 well plates in complete RPMI medium and treated or not treated with β-lapachone as described. Viability was estimated after 72 h after plating by MTT assay. Cells were normalised to their untreated counterparts. CRISPR-Cas9 knockout (KO) of the 5-lipoxygenase gene leads to an IC_{50} change for **1** from 420 nM (vector control) to 680 nM (KO) in cell line HEL over 72 h incubation. Data displayed are mean values fit to 4PL model, with error bars representing ± SEM (n=3). This experiment was performed once.

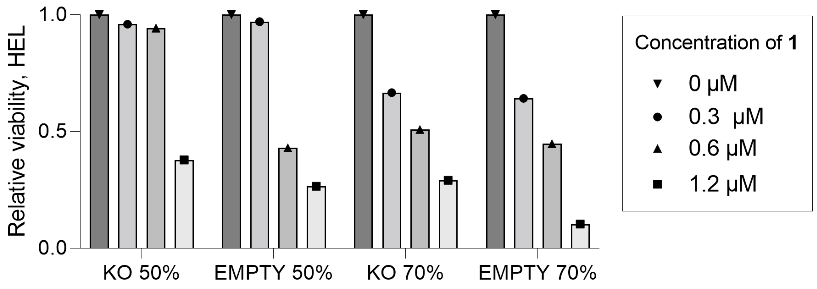

Figure 46. Additional 5-LO KO experiment assessing viability of WT and 5-LO KO HEL upon treatment with β-lapachone, 1. The graph shows relative viability of HEL cells after 72 h upon treatment with increasing concentrations of **1**. A survival effect is seen under higher concentrations of **1** in KO cells compared to comparable control vector cells. Data are n=1 and this experiment was performed once.

gRNA 1: GGATTCATACGACGTGACTG
gRNA 2: GTTGTCCTGAGGGATGGACG
gRNA 3: GAATCGAGAAGCGCAAGTAC

Figure 47. gRNA sequences used in 5-LO knockout. The three gRNAs used in the KO experiment were located on the 5-LO (ALOX-5) gene, exon 2.

Method: CRISPR knockout cell lines were generated as follows. gRNA sequences targeting areas of the 5-LO (ALOX-5) gene in were designed using Benchling software (benchling.com) and are described below. Knockout was performed according to the method of Tzelepis *et al.*.[5] Briefly, gRNAs were cloned into vector pKLV2-U6gRNA5(BbsI)-PGKpuro2ABFP-W (Addgene #67974) at cloning site BbsI. As the empty vector control the vector alone was used. HEL cells containing the Cas9 vector pKLV2-EF1a-Cas9Bsd-W (Addgene #68343) were transduced with lentiviruses carrying the gRNA containing or empty vector. Lentiviruses were produced as described previously.[6] Packaging plasmids, psPax2 (Addgene #12260) and pMD2.G (Addgene #12259) were used at the following mixing ratio: 6.26 μg lentiviral vector, 13.75 μg psPax2 and 2.97 μg pMD2.G per 10-cm dish. Transduction of HEL cells was performed in 6-well plates as follows: 1 x 10^6 cells and viral supernatant (500 μL) were mixed in 2 mL of culture medium supplemented with 2 μg Polybrene (Millipore), followed by spinfection (90 min, 850 g, 32 °C) and further incubated overnight at 37 °C. The medium was refreshed on the following day and cells were incubated for three days. After three days, cells containing the gRNA were selected with puromycin over a further three days. Following selection, % gRNA expression was analysed by BFP tag analysis. The three gRNA sequences performed equally well and produced equal knockout phenotype via BFP tag analysis. A mixture of the three gRNA expression vectors was used in the knockout experiment.

shRNA-mediated 5-LO knockdown (Kd) in AML

The effect of shRNA-mediated knockdown of 5-LO on proliferation of MOLM-13 and HEL cells was tested.

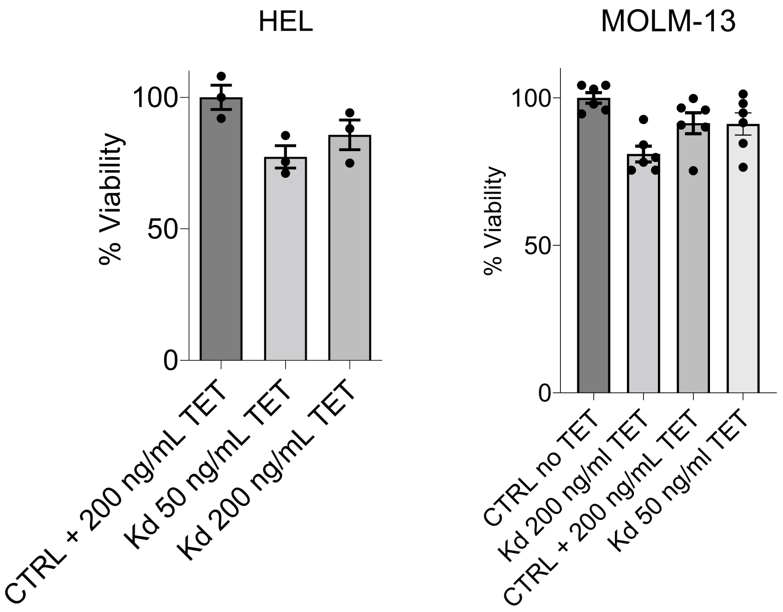

Figure 48. Effect of shRNA-mediated knockdown of 5-LO on the proliferation of AML cells. Data displayed are mean values with error bars representing ± SEM (n=3 or 6). This experiment was performed once.

Method: For virus production, 293T cells were transfected with the PLKO-TETon-Puro lentiviral vector expressing the shRNAs against the coding sequence of human *ALOX5* together with the packaging plasmids PAX2 and VSVg at a 1:1.5:0.5 ratio. Supernatant was harvested 48 and 72 h after transfection. 1 × 10^6 HEL or MOLM-13 cells and viral supernatant were mixed in 2 mL culture medium supplemented with 8 µg ml^{-1} polybrene (Millipore), followed by spinfection (60 min, 900*g*, 32 °C) and further incubated at 37 °C. 24 h after spinfection, the cells were replated in fresh medium containing 1 µg ml^{-1} of puromicin and kept in selection medium for 7 days. shRNA was induced by treatment with 200 ng ml^{-1} doxycycline for the indicated times. For viability assessment, cells with knockdown shRNA or non-active scrambled shRNA control were treated with different concentrations of knockdown inducer tetracycline for 96 h. Following this, cell viability was assessed by comparison of number of live cells divided by number of live cells for cell type (knockdown or control) not treated with tetracycline after 72 h. A decrease in cell number was seen upon knockdown of 5-LO (ALOX-5), suggesting that knockdown reduces proliferation of on AML cells and a cytostatic effect.

The effect of shRNA-mediated knockdown of 5-LO on toxicity of β-lapachone to MOLM-13 and HEL cells was tested.

a

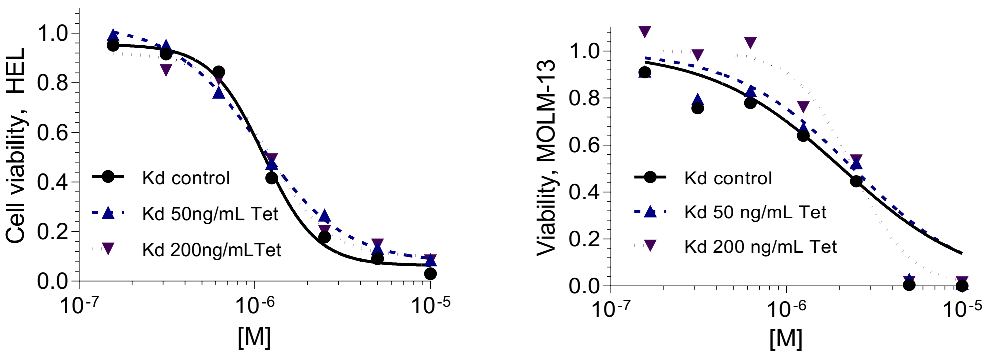

Figure 49. The effect of shRNA-mediated knockdown of 5-LO on toxicity of β-lapachone. **a,** IC_{50} change from 1.1 μM to 1.2 μM (Kd) was observed in HEL with Kd induction of 200 ng/mL tetracycline. An IC50 change from 2.1 μM to 2.4 μM (Kd) was observed in MOLM-13 with Kd induction of 200 ng/mL tetracycline. Data in (a) are mean values (n=6) fit to 4PL model. This experiment was performed once. Neither change was found to be significant.

Method: Toxicity of **1** following shRNA mediated knockdown of 5-LO in (i) HEL, (ii) MOLM-13. Kd induced with 50 ng/mL or 200 ng/mL tetracycline. HEL and MOLM-13 control or 5-LO–Kd cells (0.5×10^5 or 1.0×10^5; 4 days after tetracycline induction) were seeded in 0.5 mL complete RPMI medium, treated or not treated with **1** and live cells were counted 4 days after plating by Trypan-Blue exclusion using a Countess II instrument (ThermoFisher) according to the manufacturer's instructions. The number of tetracycline and beta-lapachone treated cells were normalized to their untreated counterparts. The knockdown (Kd) control contained a vector with a scrambled shRNA sequence.

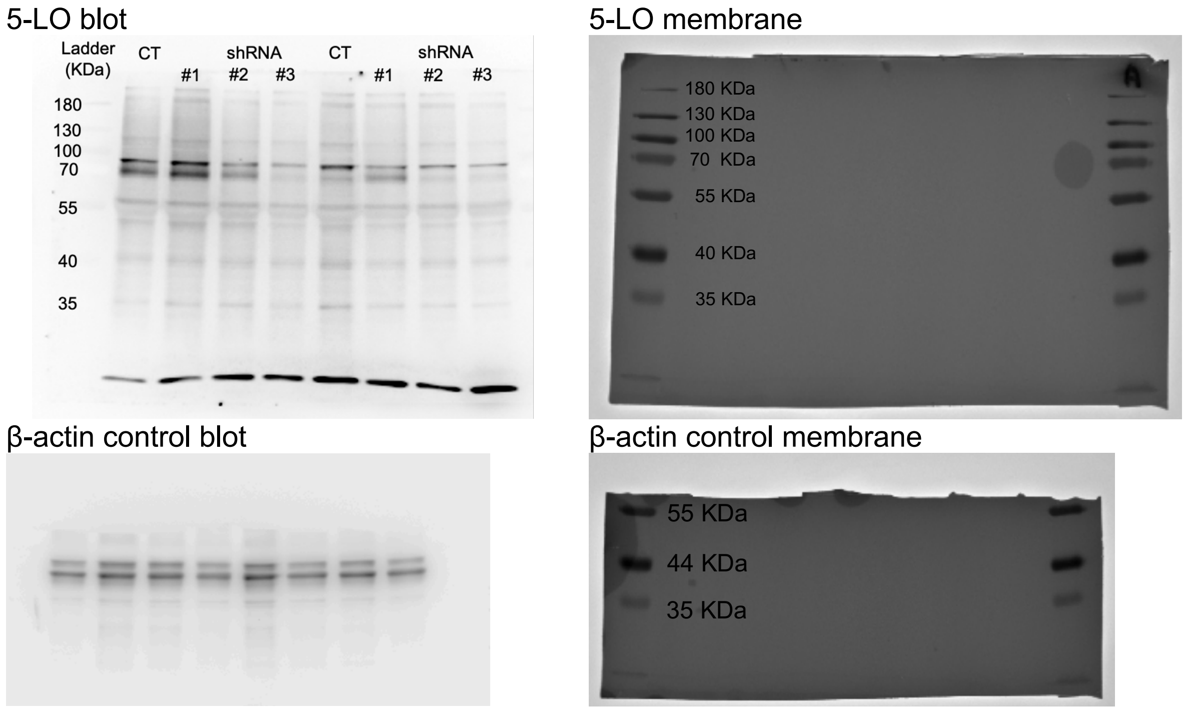

Figure 50. 5-LO Kd validation in HEL. shRNA #1 did not cause knockdown but shRNA #2 and #3 triggered a knockdown. shRNA #2 was chosen for the experiment. This experiment was performed once.

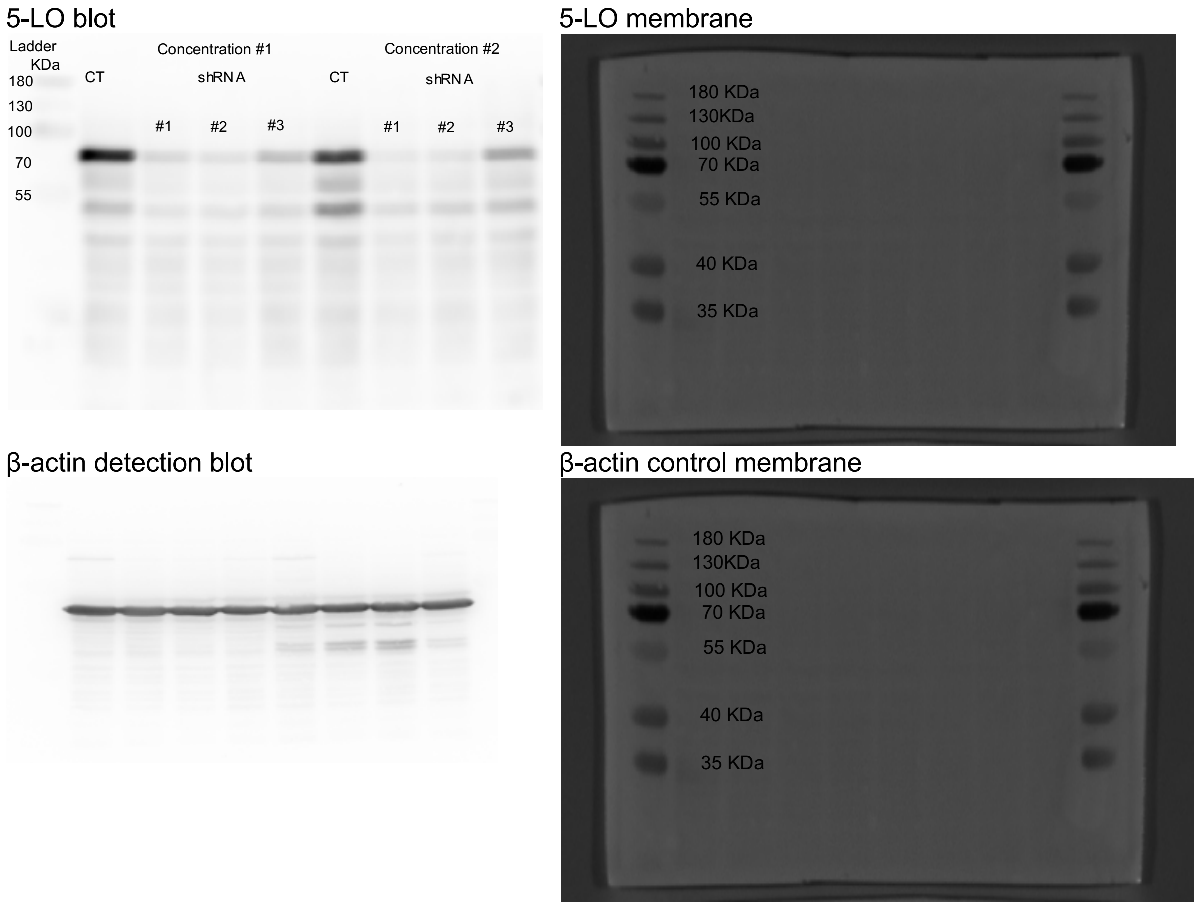

Figure 51. 5-LO Kd validation in MOLM13. shRNA #2 was chosen for the knockdown experiment. This experiment was performed once.

Sh ctrl: CAACAAGATGAAGAGCACCAACTCGAGTTGGTGCTCTTCATCTTGTTG

Sh1: CCTGTTCATCAACCGCTTCATCTCGAGATGAAGCGGTTGATGAACAGG

Sh2: CGGGAGATGAGAACCCTATTTCTCGAGAAATAGGGTTCTCATCTCCCG

Sh3: GACCACTGATAGATGTCTATTCTCGAGAATAGACATCTATCAGTGGTC

Figure 52. 5-LO shRNA sequences used.

6. Assays with dipeptide prodrugs

In-vitro cathepsin-B mediated cleavage of peptide prodrugs

Assays performed as described in Materials and Methods.

Cbz-Val-Cit-PAB-BL 26

a

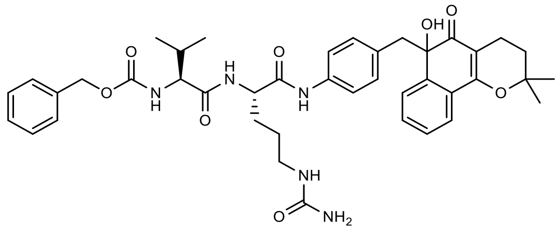

b **26** + cathepsin-B, 254 nm

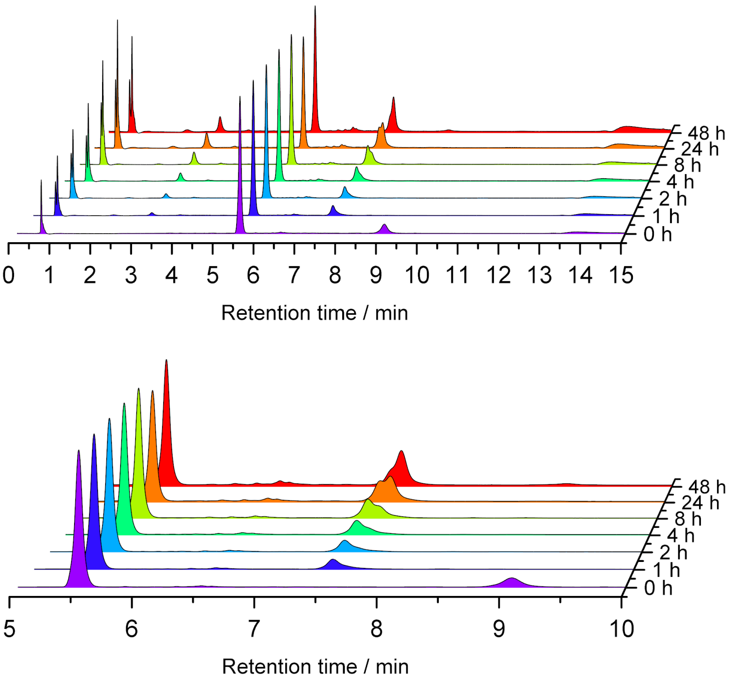

c **26**, no cathepsin-B, 254 nm

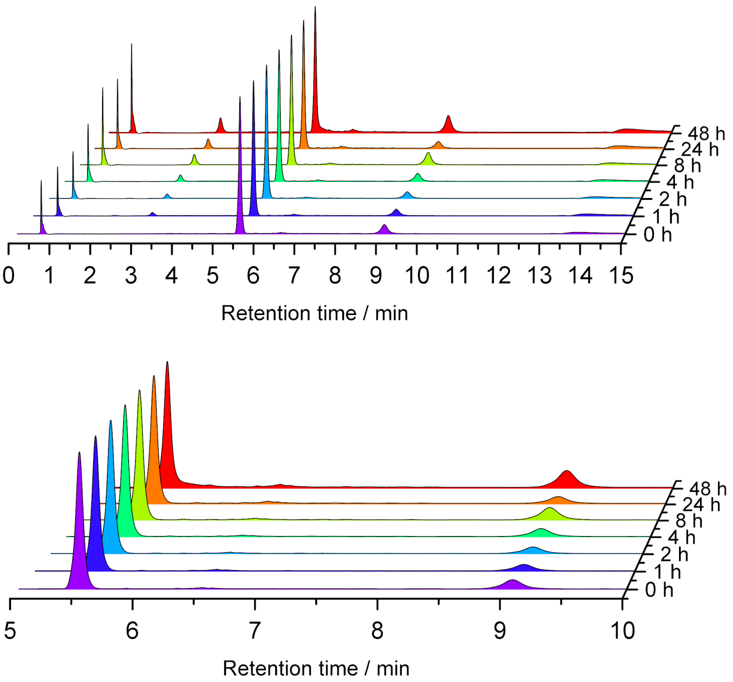

d **26** + cathepsin-B, 430 nm

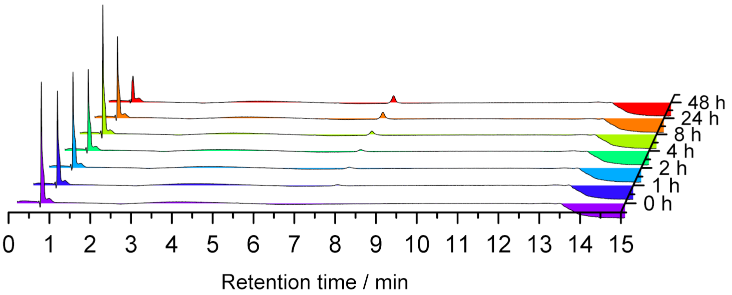

e **26**, no cathepsin-B, 430 nm

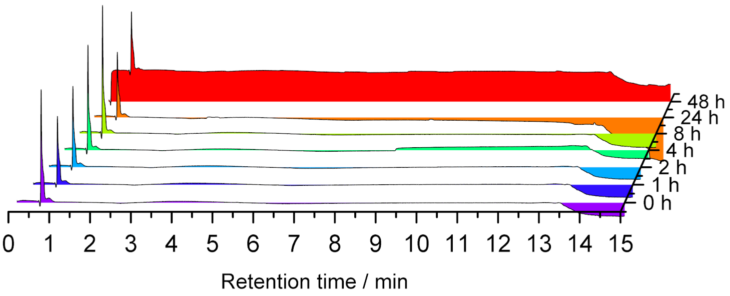

f

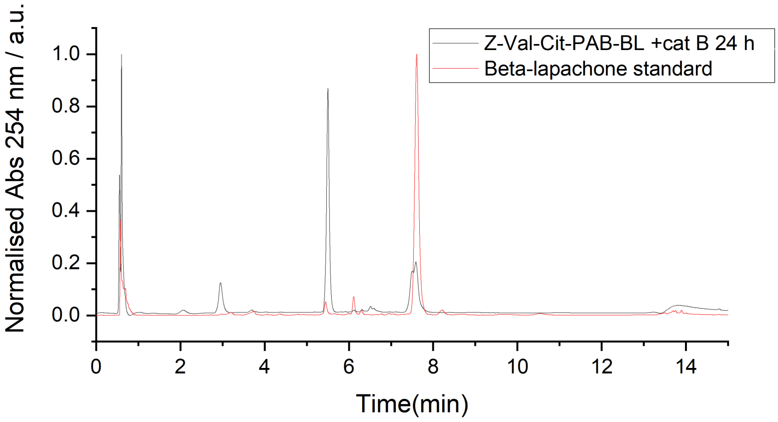

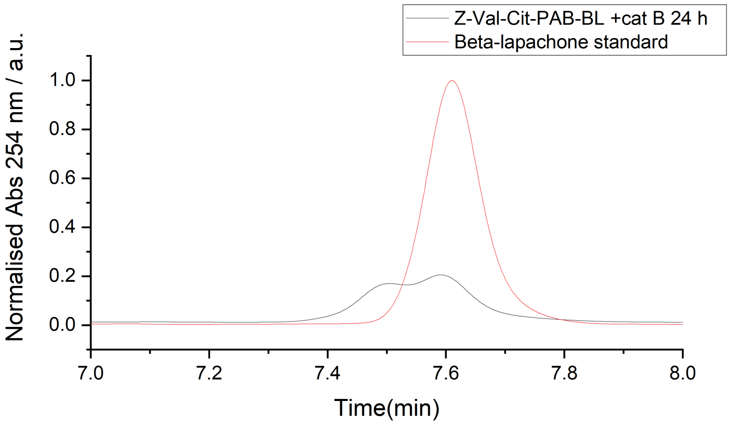

Figure 53. Cathepsin B cleavage of Z-Val-Cit-PAB-BL 26. **a**, Structure of prodrug tested. **b**, Overlay of HPLC traces of Cathepsin B treated sample recorded at 254 nm. Assignments: t = 5.5 min (acetophenone standard), t = 7.5 min (PAB-BL), t = 7.6 min (β-lapachone), t = 9.1 min (Cbz-Val-Cit-PAB-BL). **c**, Overlay of HPLC traces of control samples not treated with cathepsin B recorded at 254 nm. Assignments: t = 5.5 min (acetophenone standard), t = 9.1 min (Cbz-Val-Cit-PAB-BL). **d**, Overlay of HPLC traces of Cathepsin B treated sample recorded at 430 nm, un-normalised. Assignment: t = 7.6 min (β-lapachone). **e**, Overlay of HPLC traces of control samples not treated with cathepsin B recorded at 430 nm, un-normalised. **f**, Alignment of t = 24 h cathepsin B treated sample with a β-lapachone standard. A replicate experiment displayed similar results.

Cbz-Val-Ala-PAB-BL 27

a

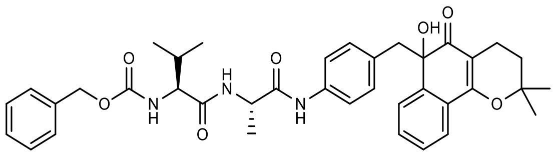

b **27** + cathepsin-B, 254 nm

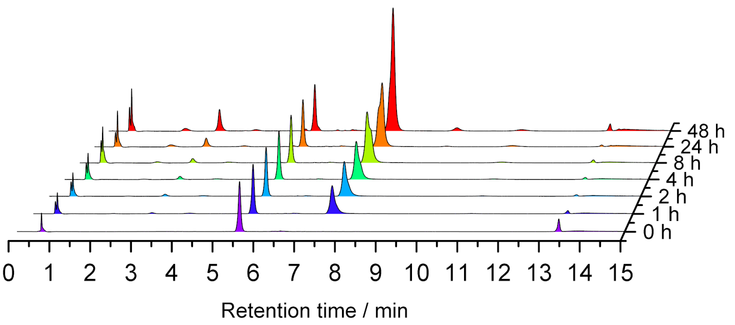

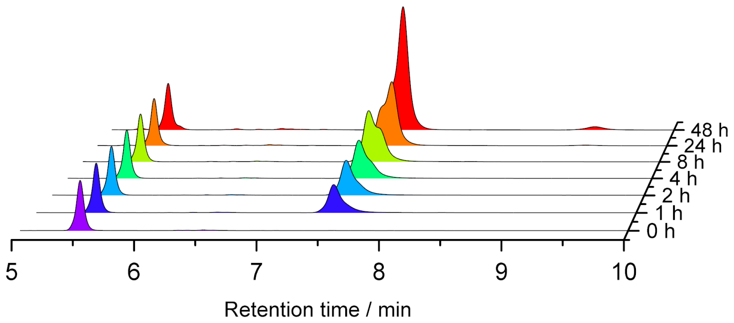

c **27**, no cathepsin-B, 254 nm

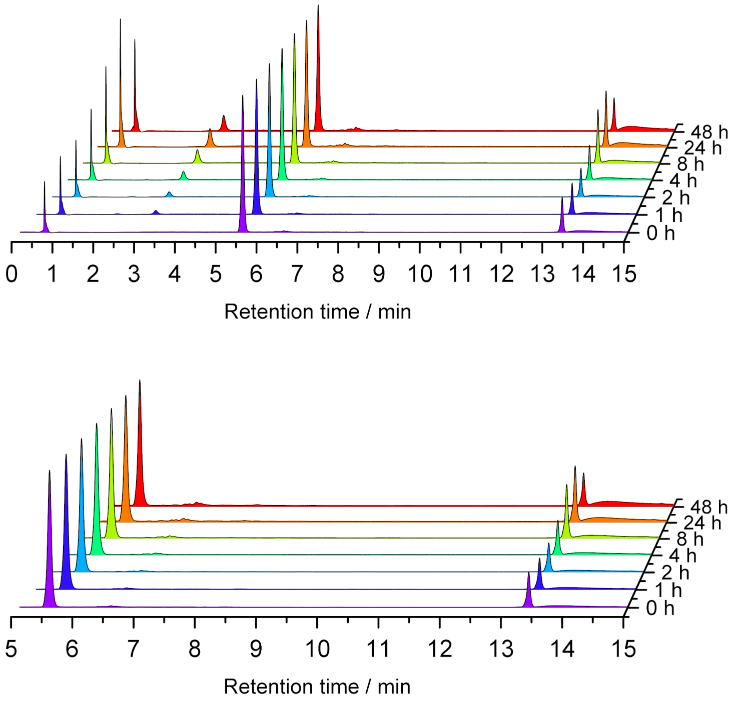

d **27** + cathepsin-B, 430 nm

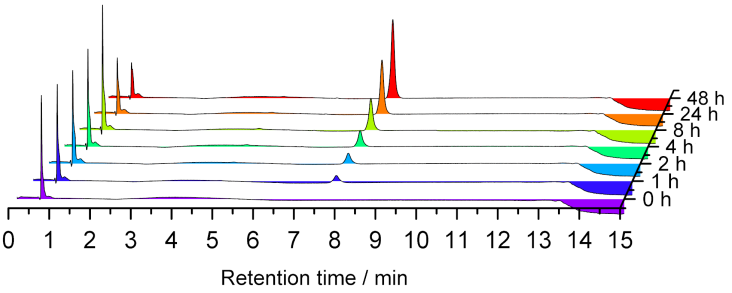

e **27**, no cathepsin-B, 430 nm

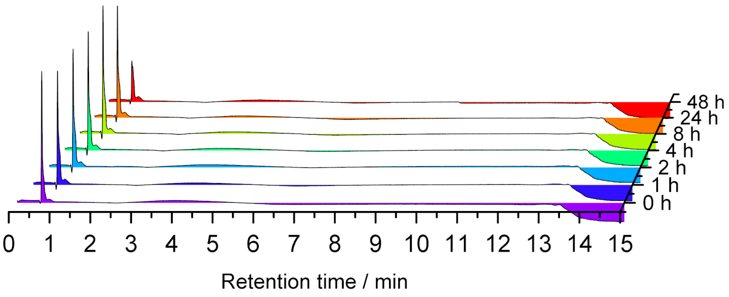

f

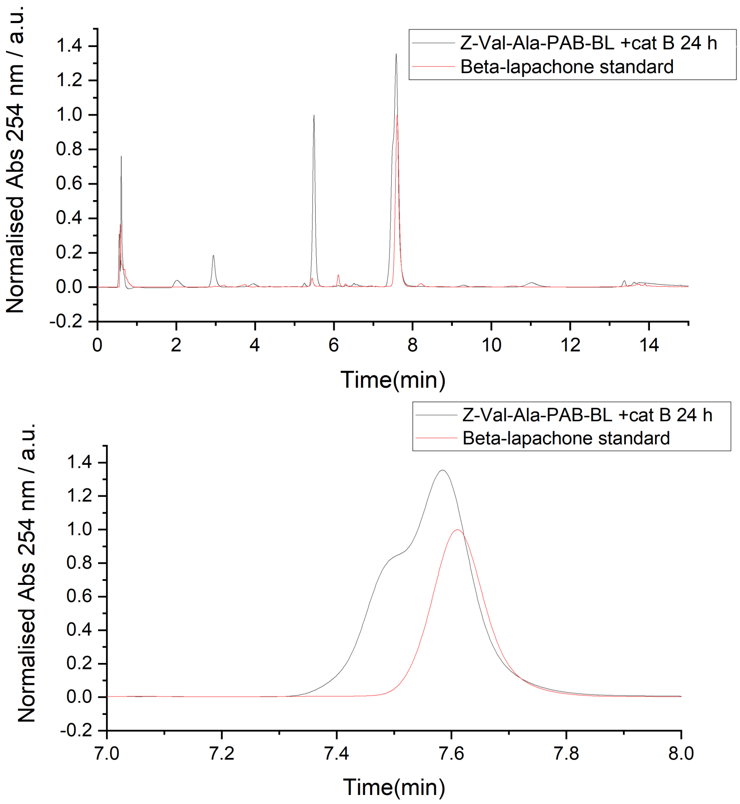

Figure 54. Cathepsin B cleavage of Z-Val-Ala-PAB-BL 27. a, Structure of prodrug tested. **b,** Overlay of HPLC traces of Cathepsin B treated sample recorded at 254 nm. Assignments: t = 5.5 min (acetophenone standard), t = 7.5 min (PAB-BL), t = 7.6 min (β-lapachone), t = 13.4 min (Cbz-Val-Ala-PAB-BL). **c,** Overlay of HPLC traces of control samples not treated with cathepsin B recorded at 254 nm. Assignments: t = 5.5 min (acetophenone standard), t = 13.4 min (Cbz-Val-Ala-PAB-BL). **d,** Overlay of HPLC traces of Cathepsin B treated sample recorded at 430 nm, un-normalised. Assignment: t = 7.6 min (β-lapachone). **e,** Overlay of HPLC traces of control samples not treated with cathepsin B recorded at 430 nm, un-normalised. **f,** Alignment of t = 24 h cathepsin B treated sample with a β-lapachone standard. A replicate experiment displayed similar results.

Cbz-Phe-Cit-PAB-BL 28

a

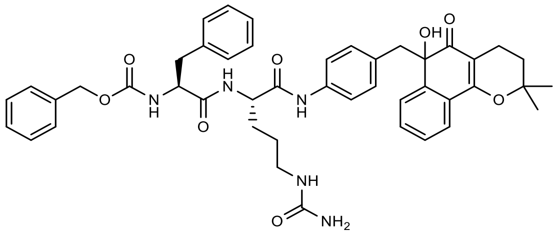

b **28** + cathepsin-B, 254 nm

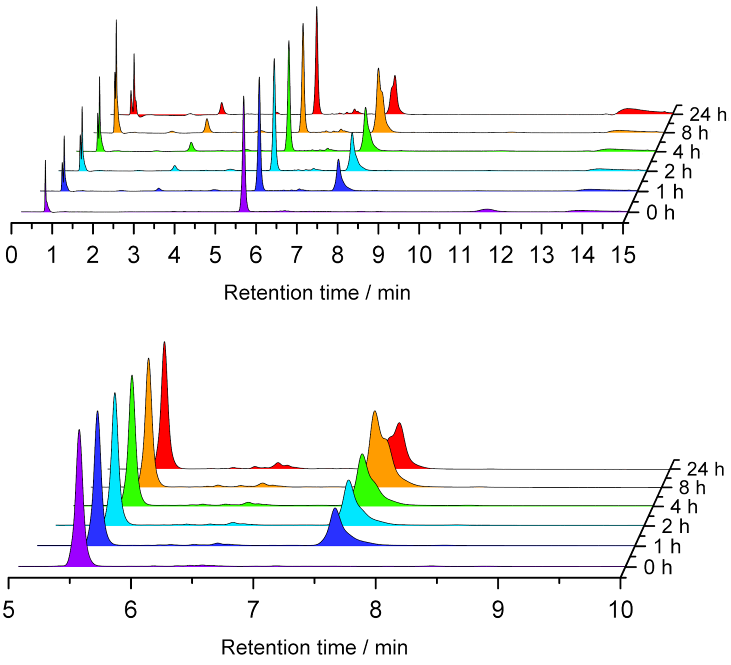

c **28**, no cathepsin-B, 254 nm

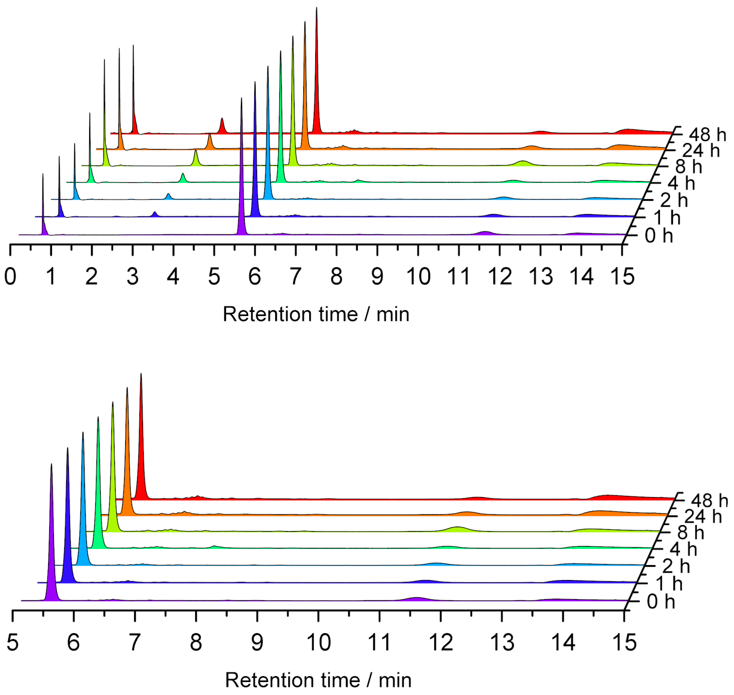

d **28** + cathepsin-B, 430 nm

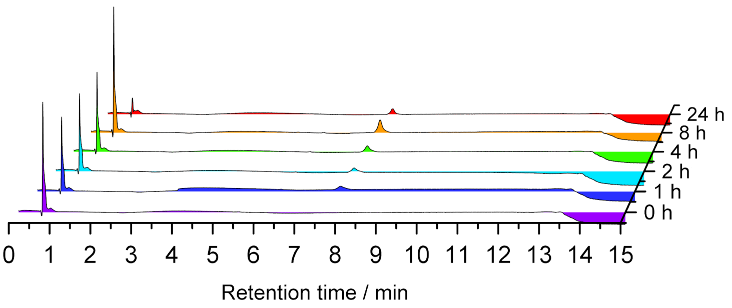

e **28**, no cathepsin-B, 430 nm

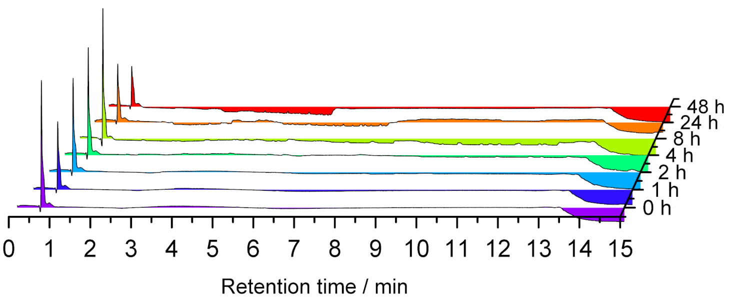

f

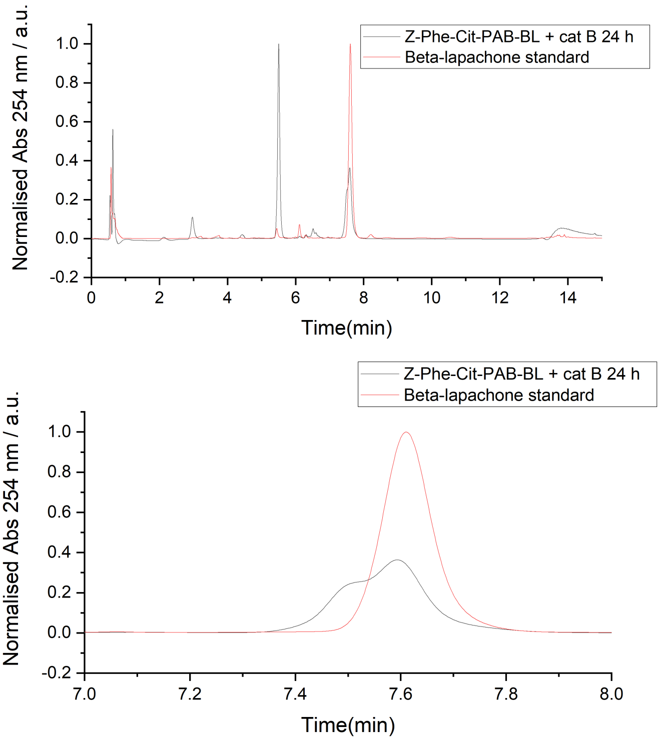

Figure 55. **Cathepsin B cleavage of Z-Phe-Cit-PAB-BL 28. a**, Structure of prodrug tested. **b**, Overlay of HPLC traces of Cathepsin B treated sample recorded at 254 nm. Assignments: t = 5.5 min (acetophenone standard), t = 7.5 min (PAB-BL), t = 7.6 min (β-lapachone), t = 11.5 min (Cbz-Phe-Cit-PAB-BL). **c**, Overlay of HPLC traces of control samples not treated with cathepsin B recorded at 254 nm. Assignments: t = 5.5 min (acetophenone standard), t = 11.7 min (Cbz-Phe-Cit-PAB-BL). **d**, Overlay of HPLC traces of Cathepsin B treated sample recorded at 430 nm, un-normalised. Assignment: t = 7.6 min (β-lapachone). **e**, Overlay of HPLC traces of control samples not treated with cathepsin B recorded at 430 nm, un-normalised. **f**, Alignment of t = 24 h cathepsin B treated sample with a β-lapachone standard. A replicate experiment displayed similar results.

NH_2-Val-Cit-PAB-BL (S7)

Method: Cathepsin B cleavage of **H_2N-Val-Cit-PAB-BL S7** was tested as follows: Cathepsin B (Abcam ab151914) was preactivated by dilution of enzyme stock (20 µL, 0.44 mg/mL, 37 kDa, 12 µM) 1:1 in activation buffer (20 µL, MES 25 mM pH 5 + 10 mM DTT) followed by incubation at 37 °C for 15 min. Following preactivation, activated cathepsin or cathepsin buffer only (5 µL) was added to a reaction of MES 20 mM pH 5 buffer (238.75 µL) containing NH_2-Val-Cit-PAB-BL (5 µL of a 25 mM stock in PBS) and internal standard acetophenone (1.25 µL of a 100 mM stock in DMF) resulting in a total reaction volume of 250 µL, at pH 5, with 0.5 % DMF, 500 µM NH_2-Val-Cit-PAB-BL and 0.12 µM of cathepsin B (4160 equiv. of substrate to enzyme). Three replicate reactions were made up for both enzyme treated and control tests. A 25 µL sample of each reaction was removed immediately at t = 0. Samples were then incubated with shaking (500 rpm) at 37 °C. Subsequent 25 µL samples were removed at specified timepoints. 20 µL of each sample was injected immediately for HPLC analysis (254 nm and 430 nm; instrument: ThermoFisher U3000; column: Phenomenex Kinetex C18, 5µm, 50 × 4.6mm, 100Å; solvent system A = H_2O, B = acetonitrile; flow = 1 mL / min; gradient: t = 0 – 1.0 min 0% B, t = 1.0 – 5.0 min 0 – 40% B, t = 5.0 – 12.0 min 40% B, t = 12.1 – 14.0 min 100% B, t = 14.1 – 15.0 min 0% B). Repeat samples were stored at 4 °C between HPLC injection. Displayed spectra are the mean of three replicates with standard error of the mean (dotted lines). Spectra at 254 nm are normalised to the height of the internal standard (acetophenone, t = 5.53 min). Peaks of PAB-BL (7.56 min) and β-lapachone (7.66 min) overlap but are distinguishable. Spectra at 430 nm are un-normalised. Appearance of β-lapachone (7.66 min) could also be clearly followed at 430 nm. Multiple small breakdown products are observed at 254 nm (unassigned). The starting material NH_2-Val-Cit-PAB-BL was not observed, likely due to its high polarity.

a NH_2-Val-Cit-PAB-BL **S7**

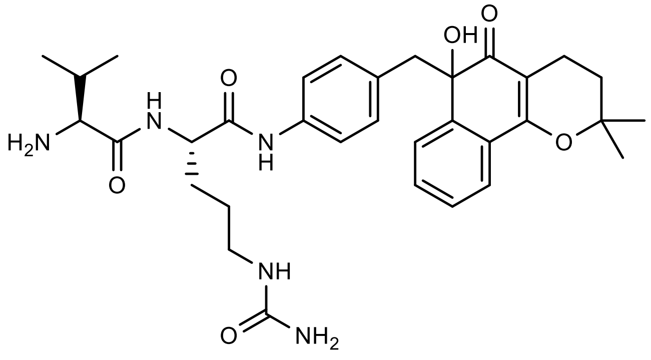

b NH$_2$-Val-Cit-PAB-BL + cathepsin-B, 254 nm

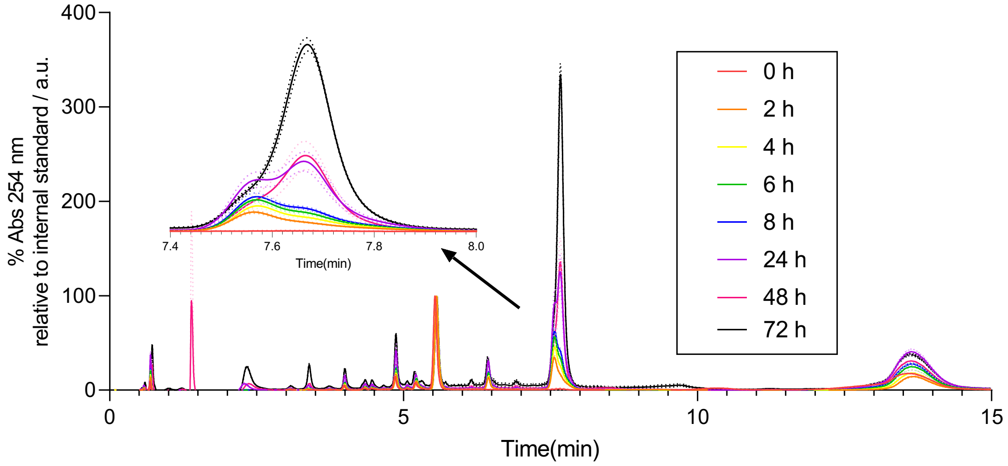

c NH$_2$-Val-Cit-PAB-BL + cathepsin-B, 430 nm

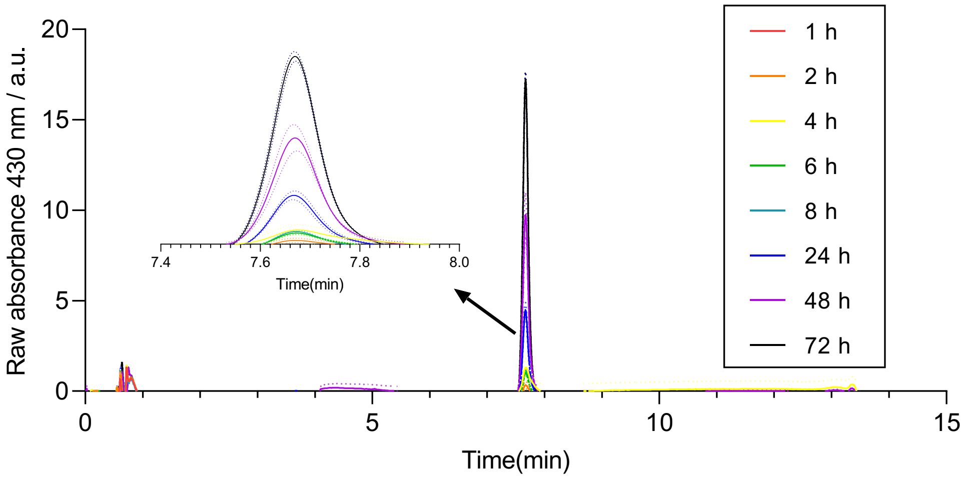

d NH2-Val-Cit-PAB-BL no cathepsin-B, 254 nm

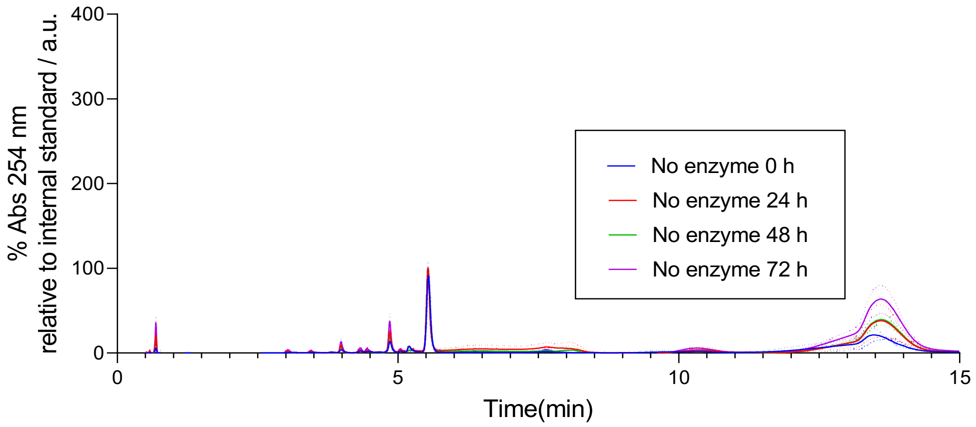

e NH2-Val-Cit-PAB-BL no cathepsin-B, 430 nm

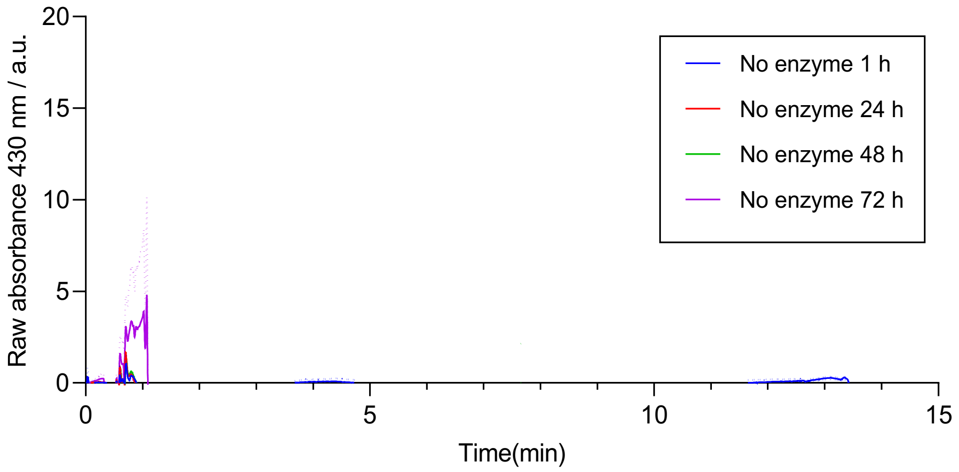

Figure 56. Cathepsin B cleavage of NH_2-Val-Cit-PAB-BL (S7). a, Structure of NH_2-Val-Cit-PAB-BL **S7**. **b,** Overlayed HPLC spectra of Cathepsin B treated sample measuring absorbance at 254 nm. Inset: zoom of peaks of overlapping intermediate PAB-BL **10** (7.56 min) and β-lapachone **1** (7.66 min). **c,** Overlayed HPLC spectra of Cathepsin B treated sample measuring absorbance at 430 nm, a maximum wavelength of β-lapachone absorbance. Inset: zoom of peak of β-lapachone 1 (7.66 min). The intermediate PAB-BL **10** does not absorb at this wavelength. **d,** Overlayed HPLC spectra of control sample with no added enzyme measuring absorbance at 254 nm. **e,** Overlayed HPLC spectra of control sample with no added enzyme measuring absorbance at 430 nm. This experiment was performed once and complements prodrug cathepsin-B experiments in **Supplementary Figures 53–55**.

Peptide prodrug toxicity to leukaemia cells

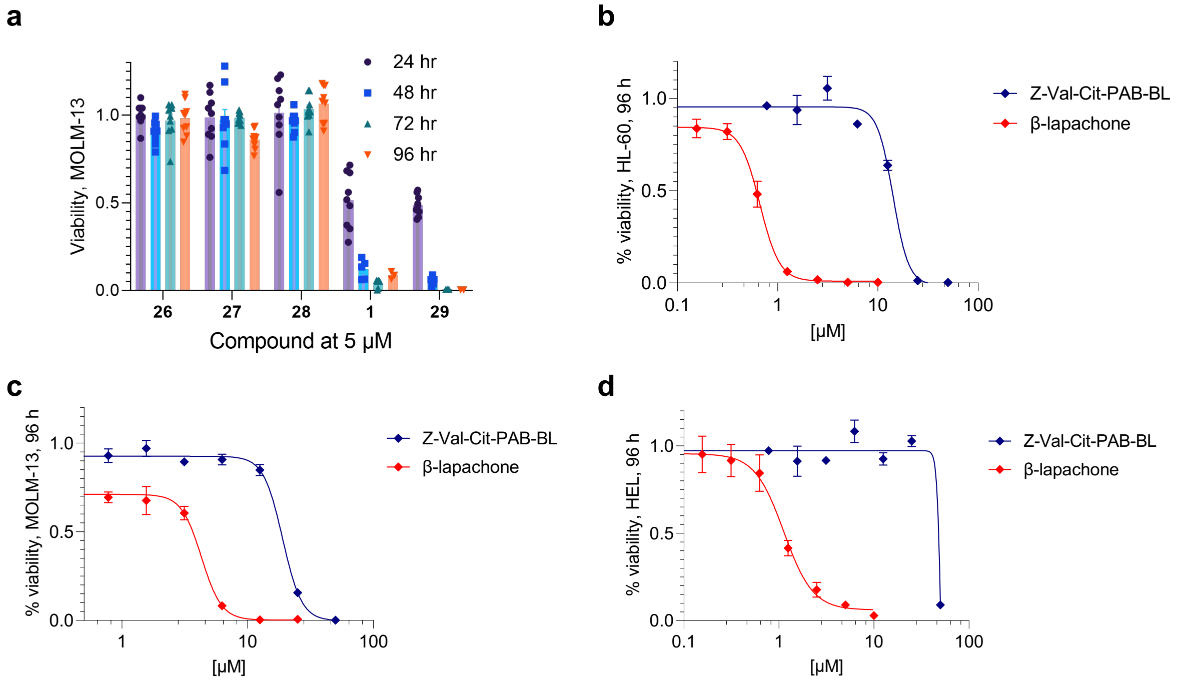

Figure 57. Toxicity of dipeptide prodrugs to leukaemia cell lines. a, Viability of leukaemia cell line MOLM-13 at 24 h timepoints following treatment with peptide prodrugs **26**, **27** and **28** at 5 µM compared to compounds **1** and **29.** Bar height represents mean cell viability (n=9). **b,** HL-60 (IC_{50} 13.8 µM for **26** vs. 0.62 µM for **1**). **c**, MOLM-13 (IC_{50} 19.8 µM for **26** vs. 2.2 µM for **1**). **d**, HEL (IC_{50} >25 µM for **26** vs. 1.1 µM for **1**). In **b**, **c** and **d**, data displayed are mean values fit to 4PL model, with error bars representing ± SEM (n=3). Viability was measured by CellTiter-Blue® assay. These experiments were performed once.

Peptide prodrug stability in human serum.

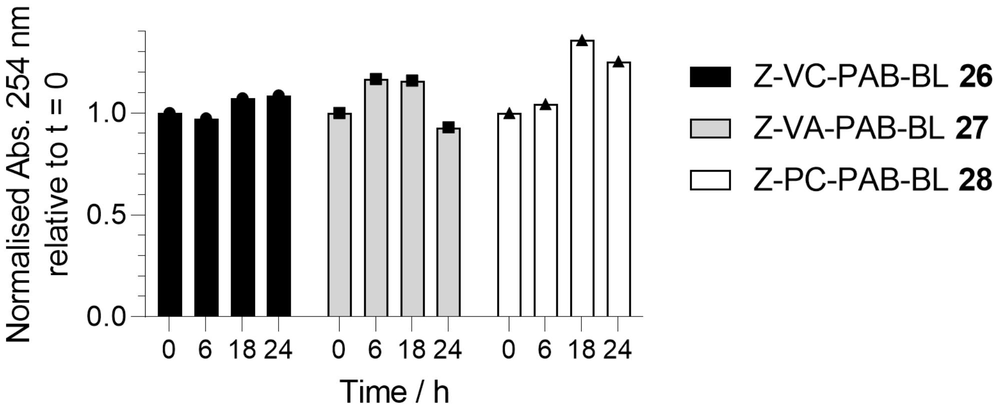

Figure 58. Peptide prodrug stability in human serum. Prodrugs appeared fully stable to incubation in human serum over 24 h. Graph displays normalised peak area. Data are n=1 and this experiment was performed once.

Method: To monitor stability of the prodrugs to serum, 10 µL of a 25 mM stock of prodrug in DMSO was added to 200 µL of human serum (Sigma H4522). 12.5 µL of a 10 mM stock of acetophenone internal standard was added. At the specified timepoints, 50 µL aliquots of the solution were removed and frozen at -20 °C. Upon completion of the experiment, proteins were precipitated from the aliquot samples by addition of 50 µL of cold acetonitrile. The samples were then centrifuged (14,000 rpm, 5 min) and the supernatant was taken. This process was repeated. The supernatant from the second centrifugation was analysed by HPLC (254 nm; instrument: ThermoFisher U3000; column: Phenomenex Kinetex C18, 5µm, 50×4.6 mm, 100 Å; solvent system A = H_2O, B = acetonitrile; flow = 1 mL / min; gradient: t = 0–1 min 0% B, t = 1–10 min 0–40% B, t = 10–16 min 40–50% B, t = 16.1 min 100% B, t = 16.1–18 min 100% B, t = 18.1–20 min 0% B). Values described represent peak area (mAU*min) divided by peak area of standard.

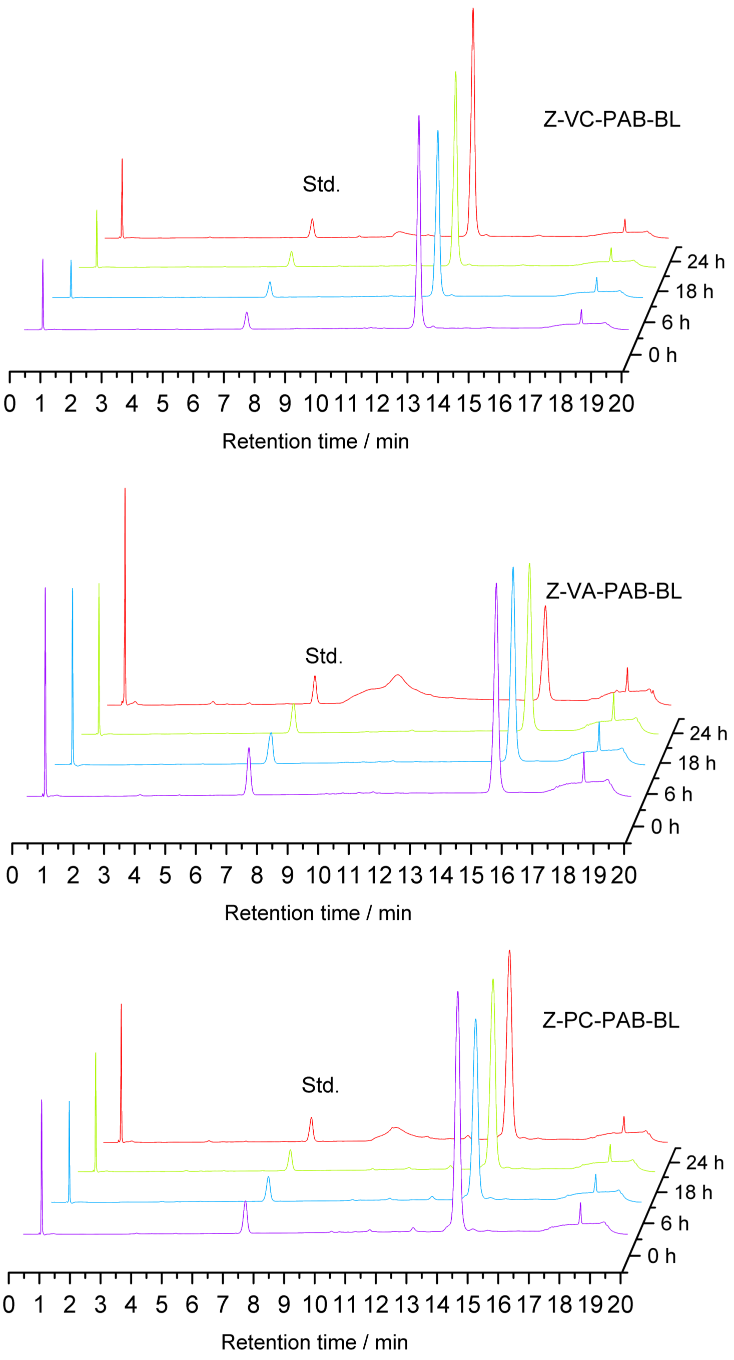

Figure 59. **Raw data HPLC traces for Figure 58**.

Toxicity of compounds following lysosomal deacidification or inhibition

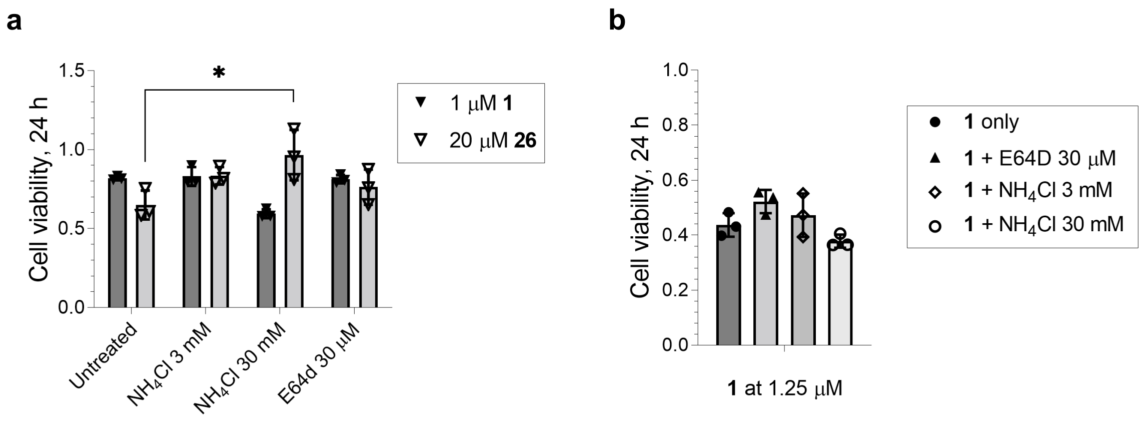

Figure 60. **a, Toxicity of compounds 1 and 26 following lysosome deacidification and inhibition in HL-60 over 24 h.** Significant difference marked: paired two-tailed t test for Z-Val-Cit-PAB-BL **26**, untreated vs. NH_4Cl 30 mM treated viability: p = 0.0199. **b, Effect of NH_4Cl and inhibitor E64d on toxicity of β-lapachone.** Viability of HL-60 cells treated with 1.25 μM β-lapachone **1** normalised to control cells treated with the same treatment (E64d, NH_4Cl, or no treatment), but without **1**. Viability following treatment with **1** with 30 mM NH_4Cl is lower than viability following treatment with **1** alone (Paired two-tailed t-test, p = 0.047 < 0.5). The experiments in **a** and **b** were repeated once with similar results obtained. Bar height displays mean values from one representative experiment with error bar displaying ± SEM (n=3).

7. Antibody and ADC QC characterisation data

Gem-HC-S442C

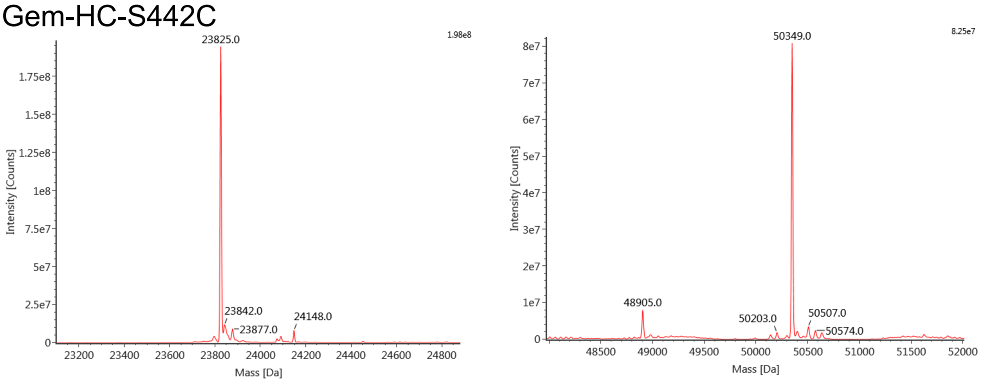

Gem-HC-239iC

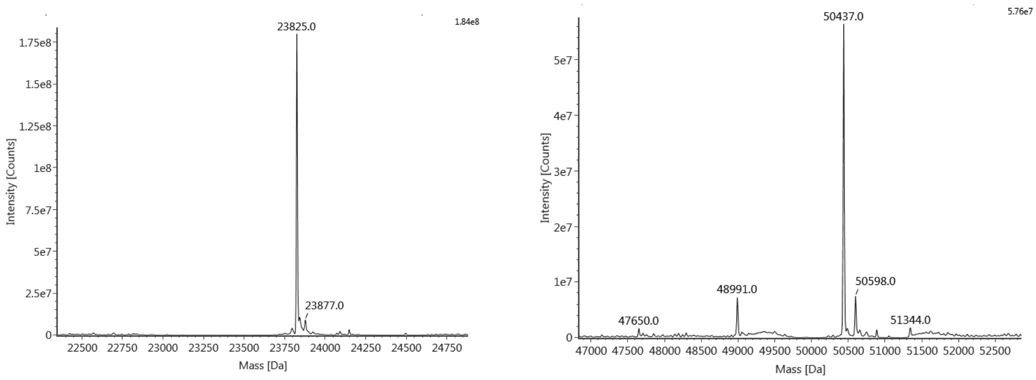

Gem-IgG1

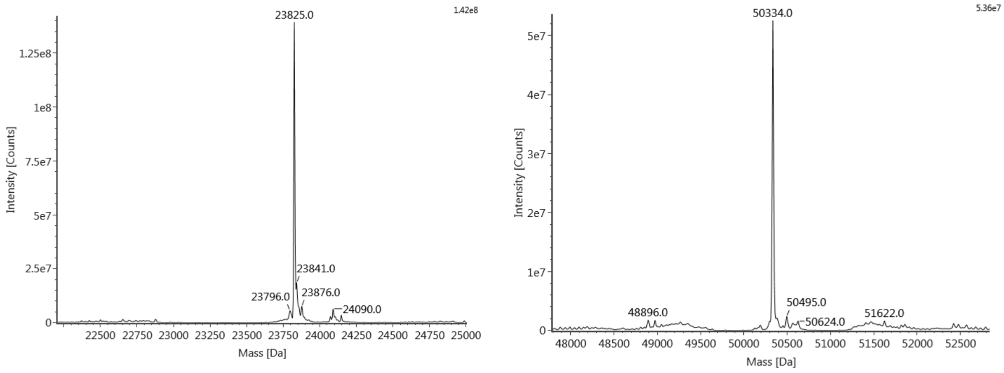

Gem-LC-V205-C

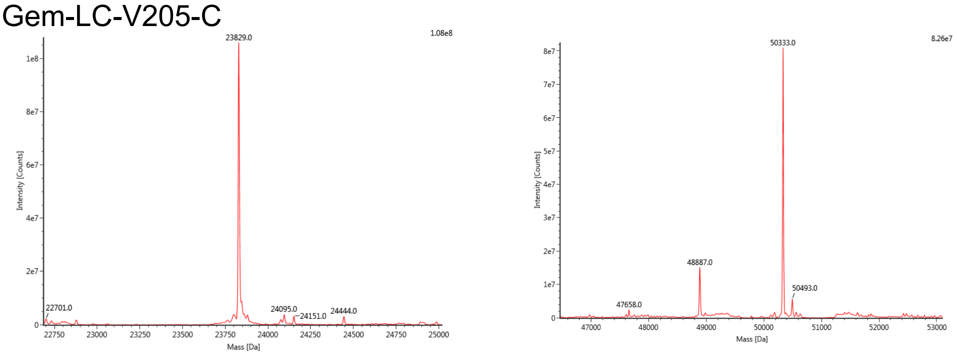

Figure 61. LCMS analysis of unmodified antibodies.

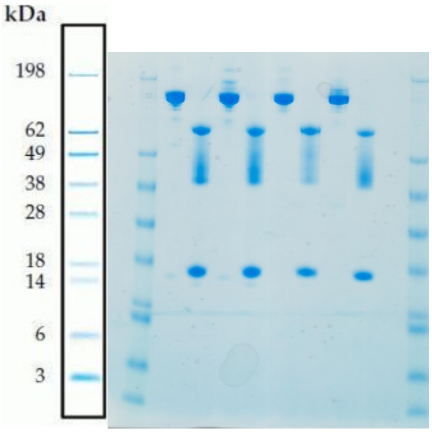

1. Ladder
2. Gem-IgG1 no TCEP
3. Gem-IgG1 with TCEP
4. Gem-IgG1-HC-S5442C no TCEP
5. Gem-IgG1-HC-S442C with TCEP
6. Gem-IgG1-HC-239iC no TCEP
7. Gem-IgG1-HC-239iC with TCEP
8. Gem-IgG1-LC-V205C no TCEP
9. Gem-IgG1-LC-V205C with TCEP
10. Ladder

Figure 62. **SDS-PAGE analysis of antibodies.** These results are representative and were replicated in repeat experiments.

Method: 4 uL of loading dye (NuPAGE™ LDS Sample Buffer (4X), cat # NP0007) was added to a sample volume of 6 uL and 6 uL of water. The loading dye either contained no (-) or 50 mM (+) Bond-Breaker™ TCEP Solution, Neutral pH. The samples were heated to 98 °C for ~ 4 min and quickly centrifuged. 4 uL of the sample was loaded on an Invitrogen NuPAGE 4-12% Bis-Tris Plus Gels (Invitrogen™, cat # NW04122BOX) and run at 160 V for 40 min with MES as running buffer (NuPAGE™ MES SDS Running Buffer (20X), Invitrogen™, cat # NP0002). 5 uL of SeeBlue™ Pre-stained Protein Standard was used as ladder (ThermoFisher Scientific, cat # LC5625). The gel was stained using InstantBlue™ Protein Stain (Expedeon, cat # ISB1L).

Construct	Chromatogram
Gem-IgG1	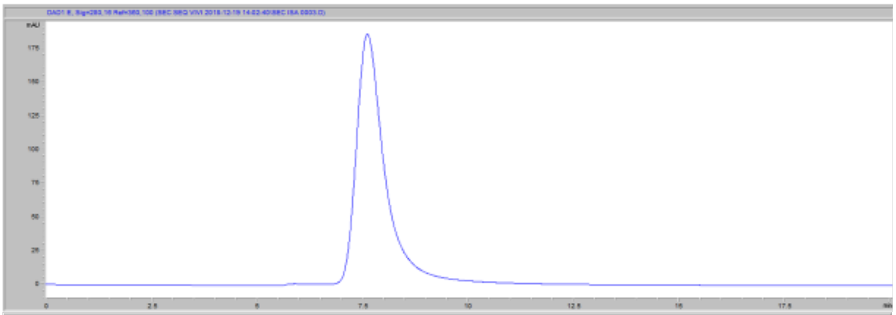
Gem-HC-S442C	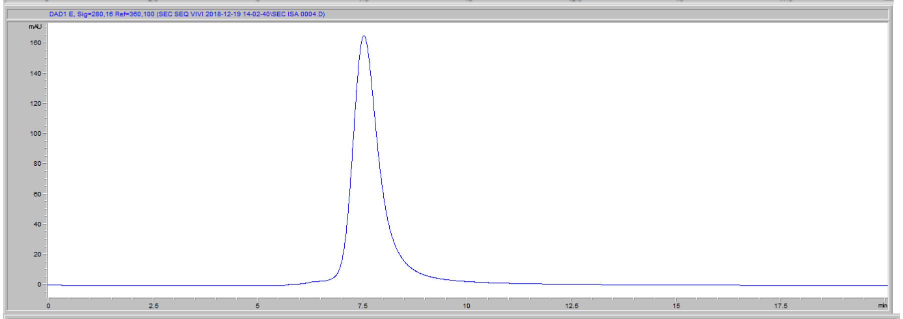
Gem-HC-239iC	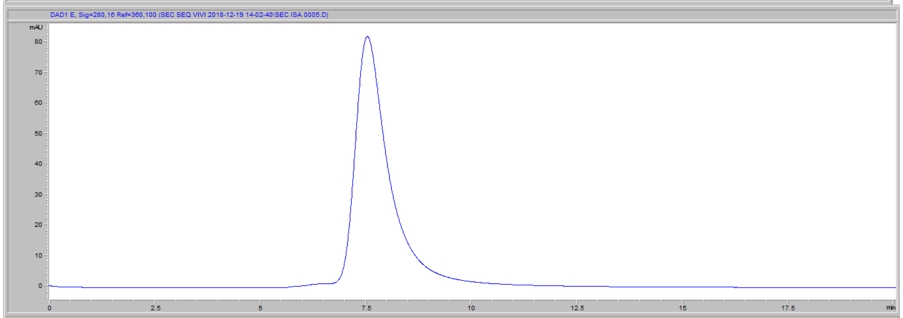
Gem-LC-V205C	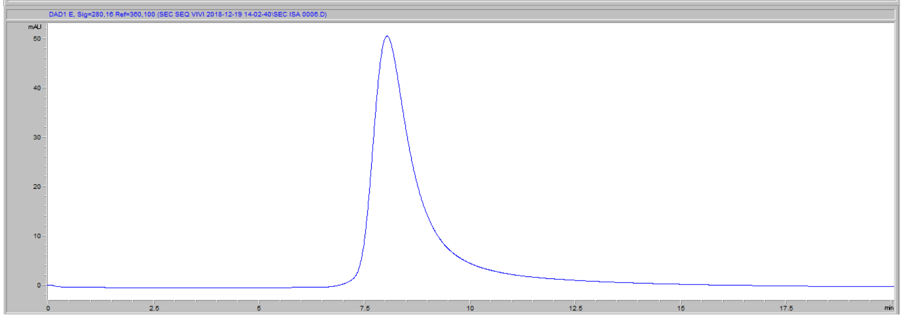

Figure 63. HP-SEC analysis of antibodies. Performed on Agilent 1100 Series HPLC Value System.

Binding of antibodies and ADCs to CD33 antigen

Method: Binding to CD33 was analysed using an Octet platform (Octet® RED384 System, ForteBio) based on bio-layer interferometry (BLI) technology. For the experiments, the kinetic buffer contained 0.02 % TWEEN20 and 0.1 % BSA in PBS. The antigen (CD33 Protein, Human, Recombinant, 27.4 kDa) was purchased from SinoBiological. To measure the kinetics of Gem-BL variants, 1.0 ug/mL of Gem-BL was loaded on the Protein A sensor tips for 120 s to achieve a binding intensity between 0.5-1.0 nm. A 2-fold serial dilution of CD33 (50 nM starting concentration) was used. The binding curves were fitted to a 1:1 global fit.

a Gem-IgG1

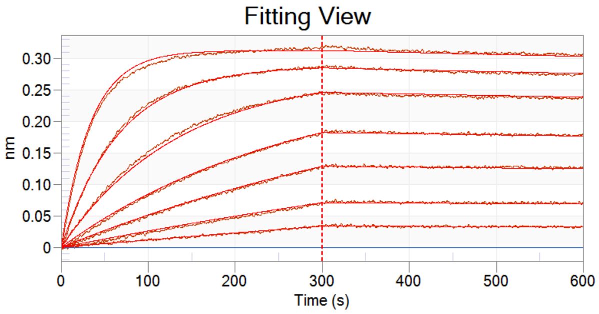

Loading Sample ID	K_D (M)	K_D Error	k_{on} (1/Ms)	k_{on} Error	k_{dis} (1/s)	k_{dis} Error	Full R^2
Gem-IgG1	1.76×10^{-10}	4.34×10^{-12}	5.89×10^{5}	1.33×10^{3}	1.03×10^{-4}	2.54×10^{-6}	0.9993

b Gem-HC-239iC

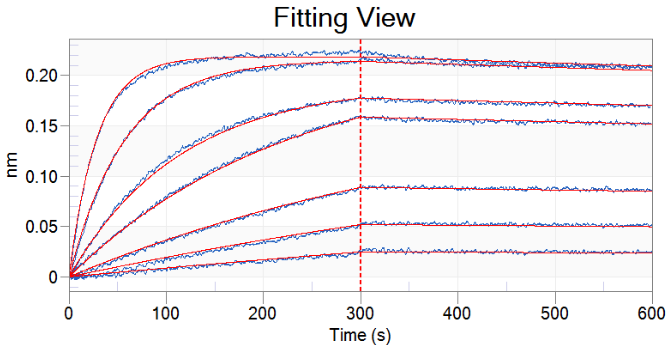

Loading Sample ID	K_D (M)	K_D Error	k_{on} (1/Ms)	k_{on} Error	k_{dis} (1/s)	k_{dis} Error	Full R^2
Gem-HC-239iC	2.10×10^{-10}	3.61×10^{-12}	6.90×10^{5}	1.52×10^{3}	1.45×10^{-4}	2.47×10^{-6}	0.9993

c Gem-HC-S442C

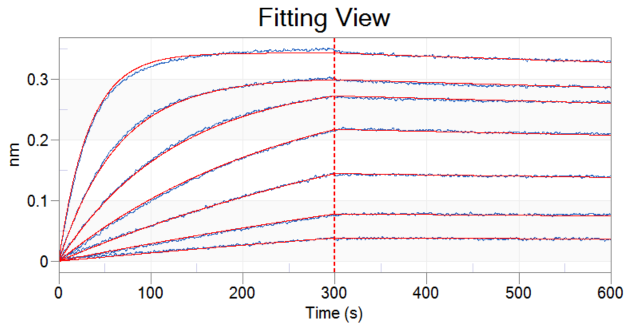

Loading Sample ID	K_D (M)	K_D Error	k_{on} (1/Ms)	k_{on} Error	k_{dis} (1/s)	k_{dis} Error	Full R^2
Gem-HC-S442C	2.45×10^{-10}	3.14×10^{-12}	6.15×10^{5}	1.05×10^{3}	1.50×10^{-4}	1.91×10^{-6}	0.9996

d Gem-LC-V205C

Two different affinities were measured for Gem-L-V205C, a frozen/thawed sample and a sample not frozen. The samples bind the antigen CD33 and in a very similar affinity range.

Never frozen

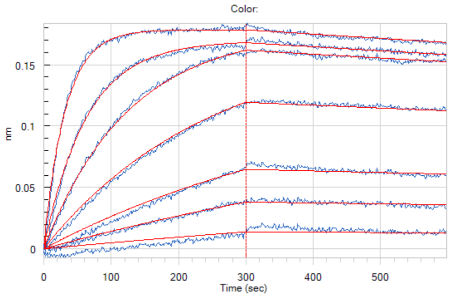

Freeze/thaw cycle

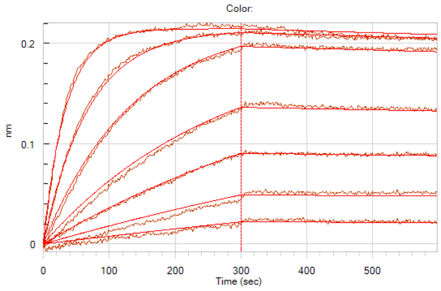

Loading Sample ID	K_D (M)	K_D Error	k_{on} (1/Ms)	k_{on} Error	k_{dis} (1/s)	k_{dis} Error	Full R^2
Never frozen	3.23×10^{-10}	6.48×10^{-12}	6.19×10^{5}	2.15×10^{3}	2.00×10^{-4}	3.95×10^{-6}	0.9984
Freeze/thaw cycle	1.57×10^{-10}	6.69×10^{-12}	5.78×10^{5}	1.98×10^{3}	9.08×10^{-5}	3.86×10^{-6}	0.9984

e Gem-HC-239iC-BL

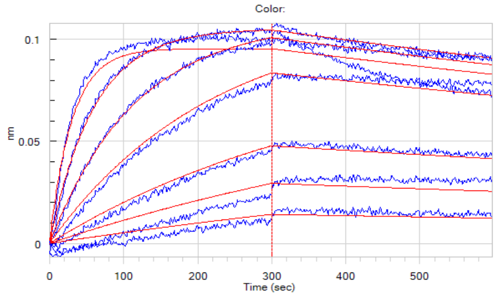

Loading Sample ID	K_D (M)	K_D Error	k_{on} (1/Ms)	k_{dis} (1/s)	Full R^2
Gem-HC-239iC-BL	9.47×10^{-10}	1.45×10^{-11}	6.51×10^{5}	6.17×10^{-4}	0.9923

f Gem-HC-S442C-BL

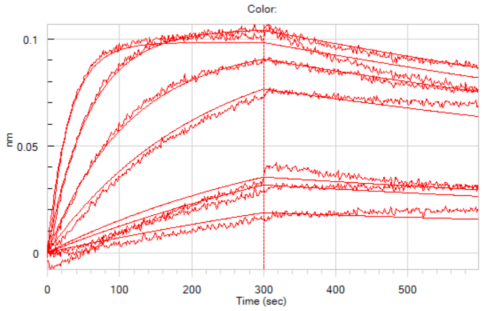

Loading Sample ID	K_D (M)	K_D Error	k_{on} (1/Ms)	k_{dis} (1/s)	Full R^2
Gem-HC-S442C-BL	7.31×10^{-10}	1.59×10^{-11}	6.49×10^{5}	4.74×10^{-4}	0.9897

g Gem-LC-V205C-BL

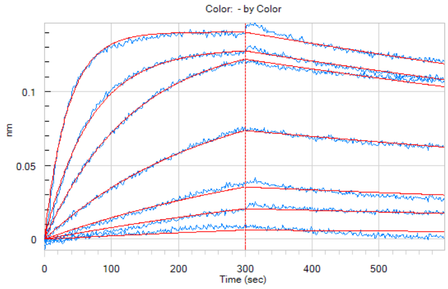

Loading Sample ID	K_D (M)	K_D Error	k_{on} (1/Ms)	k_{dis} (1/s)	Full R^2
Gem-LC-V205C-BL	1.00×10^{-9}	8.64×10^{-12}	5.55×10^{5}	5.56×10^{-4}	0.9984

Figure 64. Binding of antibodies and ADCs to CD33 antigen.

Antibody modification reactions

Antibody	Buffer	pH	Equiv./cyst	Time / h	Temp / °C	% conversion
Gem-LC-V205C	NaPi 20mM	8	40	6	25	>95
Gem-HC-S442C	NaPi 20mM	8	30	6	25	>95
Gem-HC-239iC	NaPi 20mM	8	20	6	25	>95
NIP228-HC-239iC	NaPi 20mM	8	40	24	25	>95

Figure 65. Summary of conditions used for antibody modification.

Reduced LC-MS spectra of antibodies and ADCs

Gem-LC-V205C

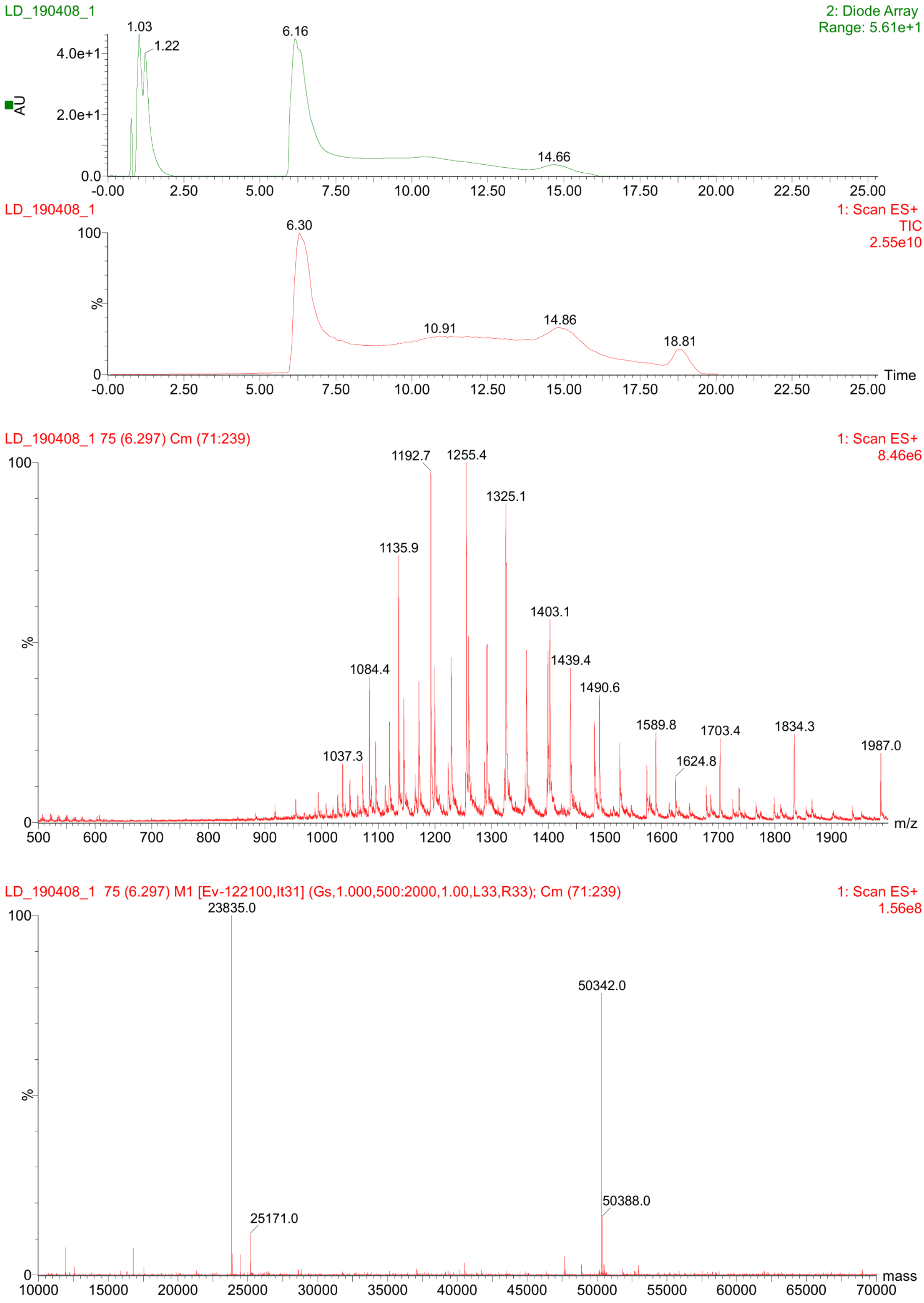

Figure 66. Reduced LCMS spectrum of Gem-LC-V205C. Exp. (LC) 23833 (HC) 50336. Obs. (LC) 23835 (HC) 50342.

Gem-HC-S442C

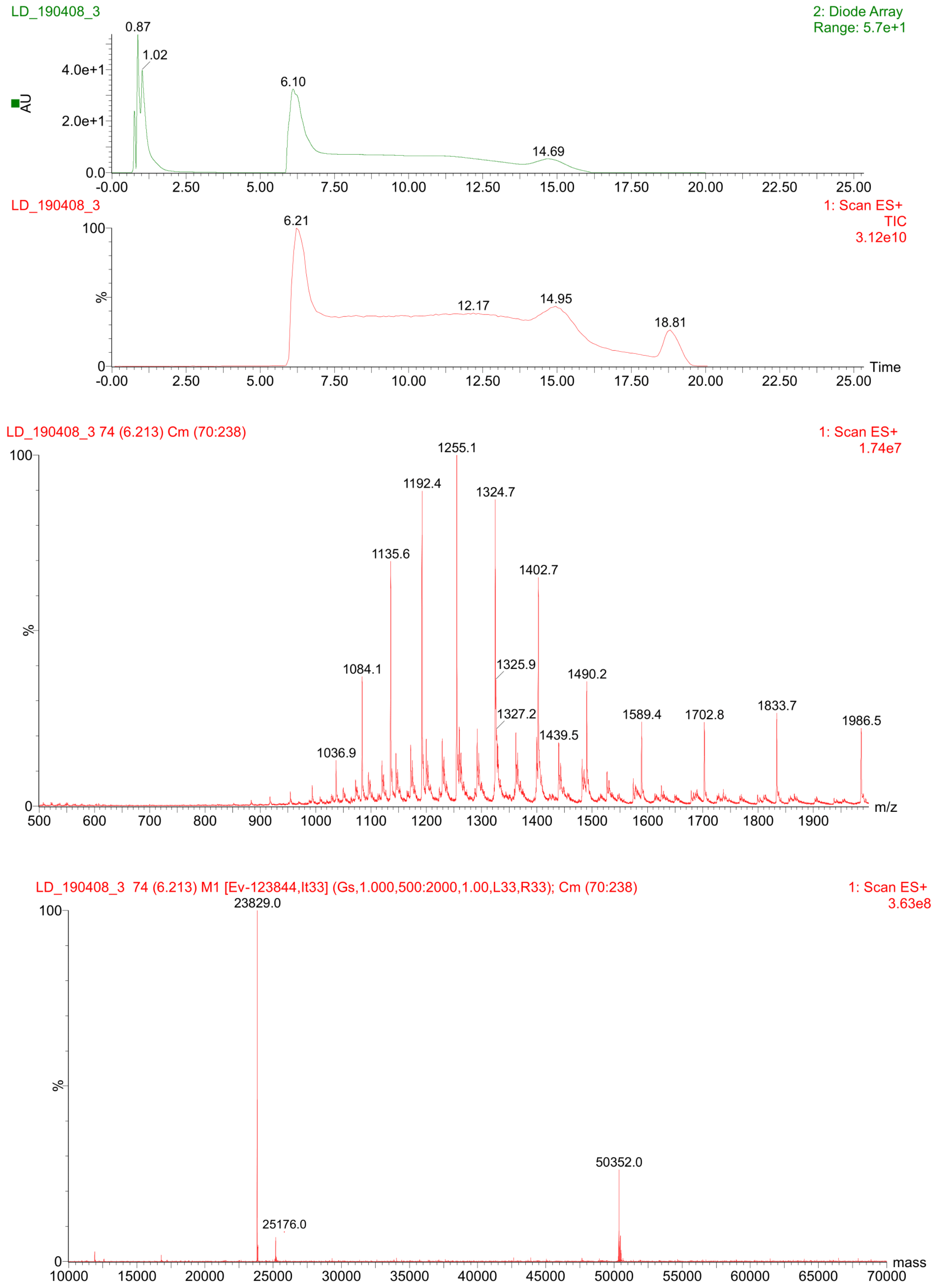

Figure 67. Reduced LCMS spectrum of Gem-HC-S442C. Exp. (LC) 23829 (HC) 50352. Obs. (LC) 23829 (HC) 50352.

Gem-HC-239iC

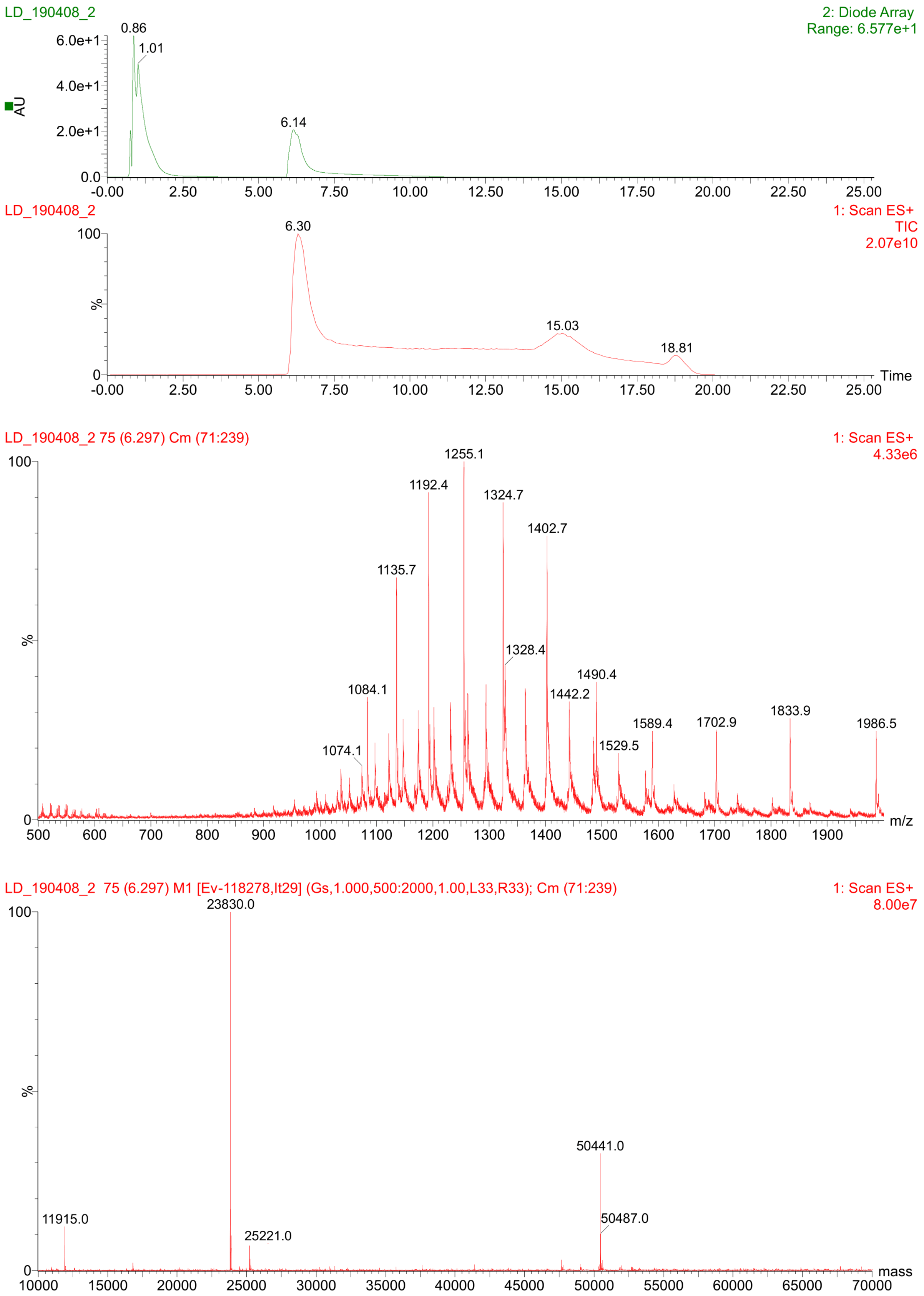

Figure 68. Reduced LCMS spectrum of Gem-HC-239iC. Exp. (LC) 23829 (HC) 50439. Obs. (LC) 23830 (HC) 50441**.**

NIP228-HC-239iC

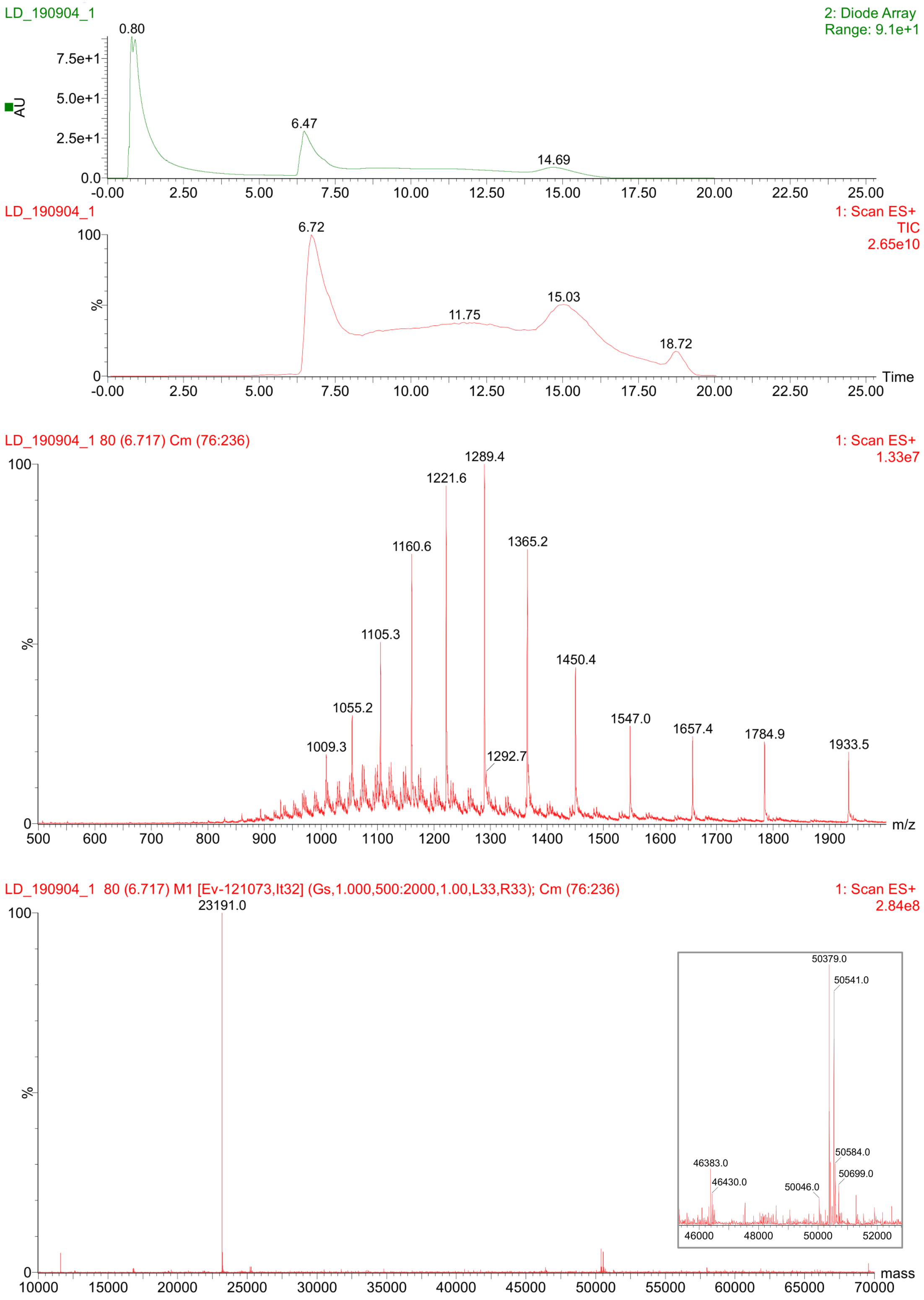

Figure 69. **Reduced LCMS spectrum of NIP228-HC-239iC.** Obs. (LC) 23191 (HC#1) 50379 (HC#2) 50541.

Gem-LC-V205C-BL

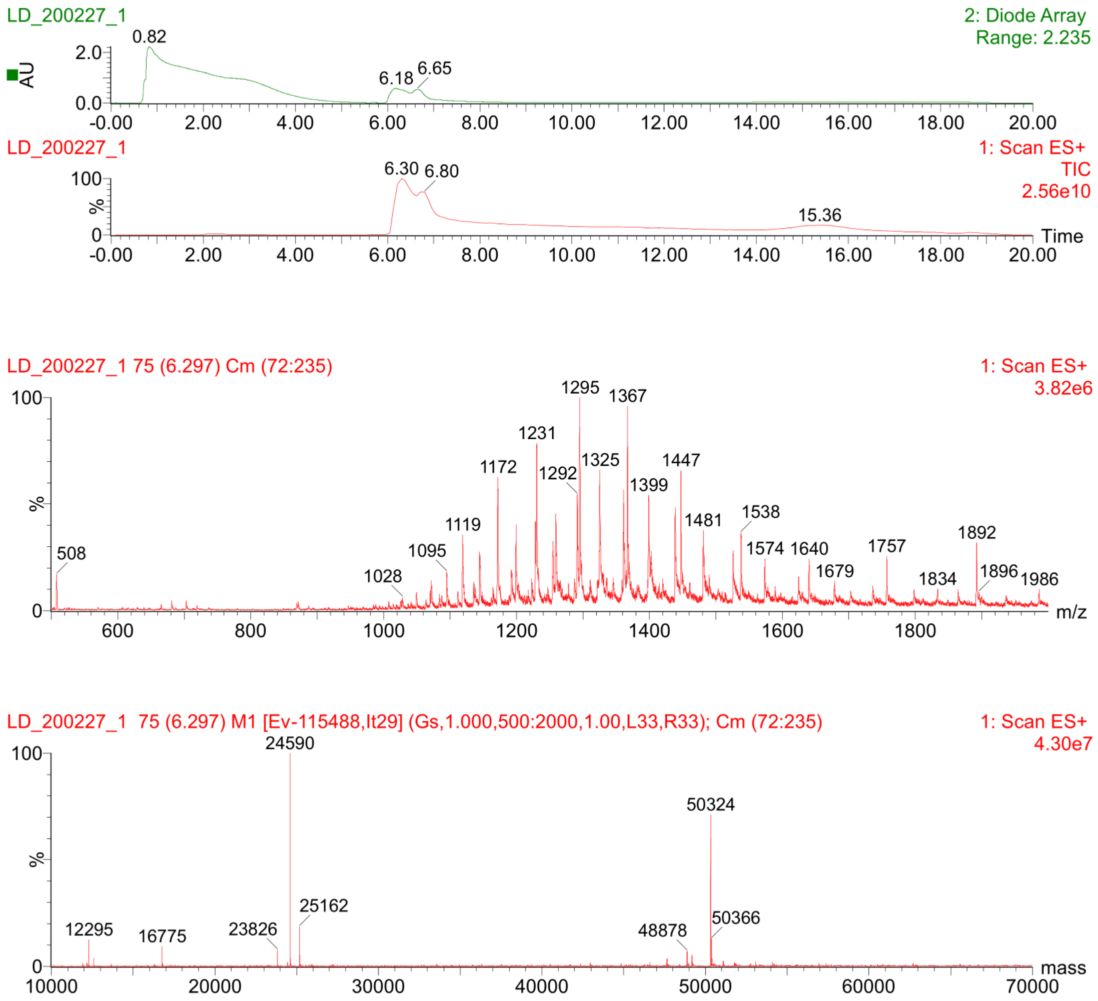

Figure 70. Reduced LCMS spectrum of Gem-LC-V205C-BL. Exp. 23833 (LC unmodified), 24596 (LC modified), 50336 (HC unmodified). Obs. 23826 (LC unmodified), 24590 (LC modified), 50324 (HC unmodified). Conversion estimate: > 95 %.

Gem-HC-442C-BL

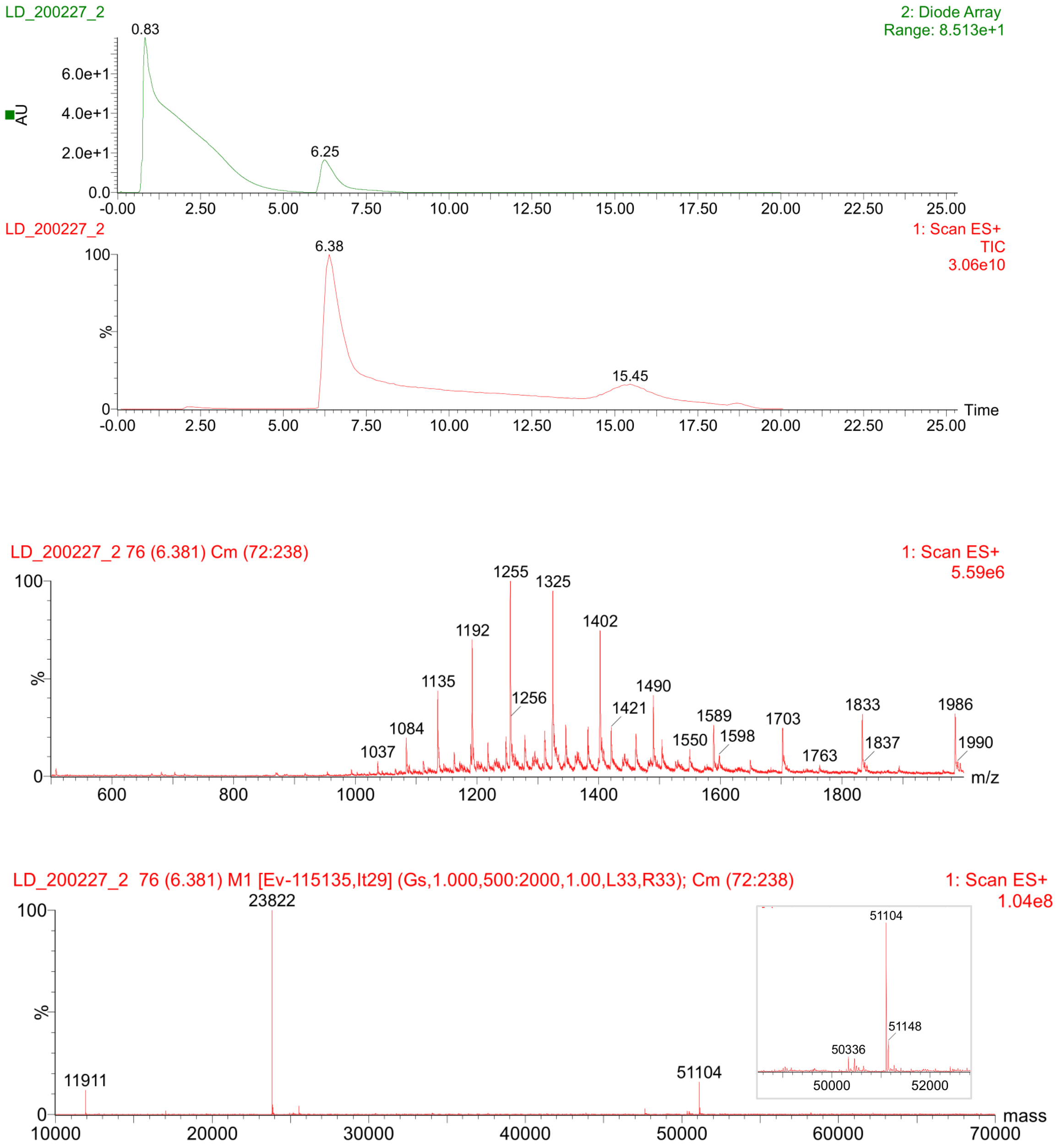

Figure 71. **Reduced LCMS spectrum of Gem-HC-S442C-BL**. Exp. 23829 (LC unmodified), 50352 (HC unmodified), 51115 (HC modified). Obs. 23822 (LC unmodified), 50336 (HC unmodified), 51104 (HC modified). Conversion estimate: > 95 %.

Gem-HC-239iC-BL

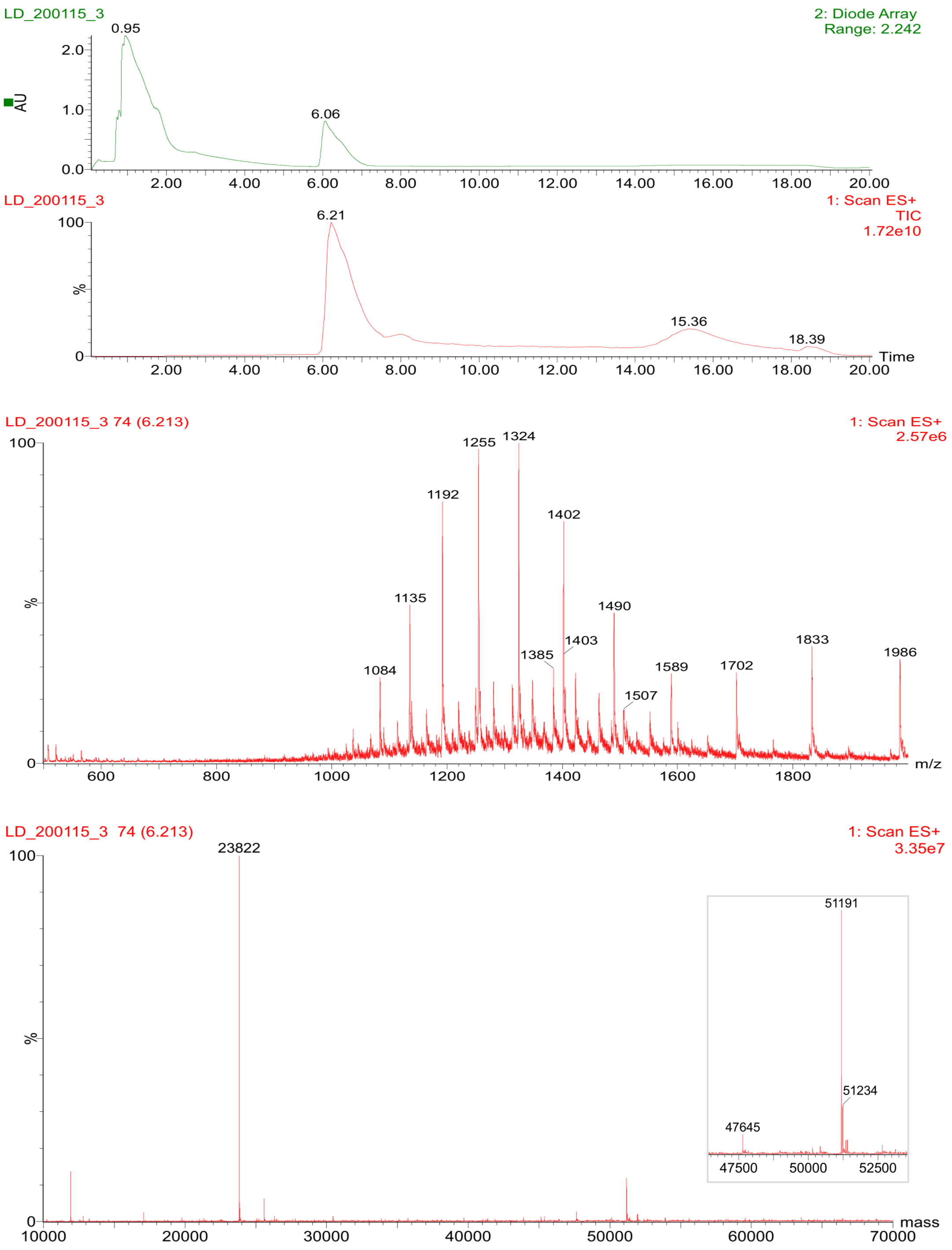

Figure 72. Reduced LCMS spectrum of Gem-HC-239iC-BL. Exp. 23829 (LC unmodified), 50439 (HC unmodified), 51202 (HC modified). Obs. 23822 (LC unmodified), 51191 (HC modified). Conversion estimate: > 95 %.

NIP228-HC-239iC-BL

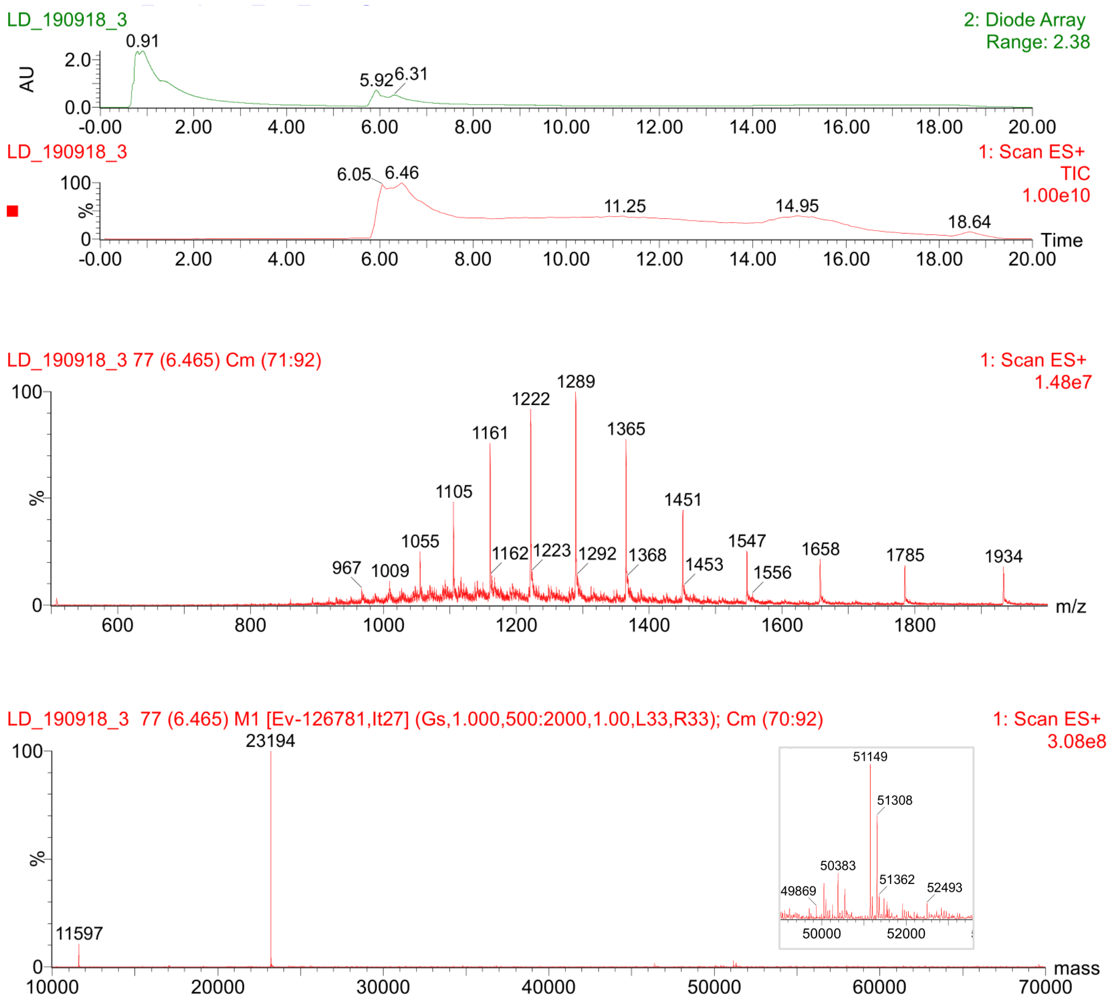

Figure 73. Reduced LCMS spectrum of NIP228-HC-239iC-BL. Exp. 23191 (LC), 50379 (HC#1 unmodified), 50541 (HC#2 unmodified), 51142 (HC#1 modified), 51304 (HC#2 modified). Obs. 23194 (LC), 51149 (HC#1 modified), 51308 (HC#2 modified). Conversion estimate: 77 %.

Native Mass Spectrometry of antibodies and ADCs

Gem-LC-V205C

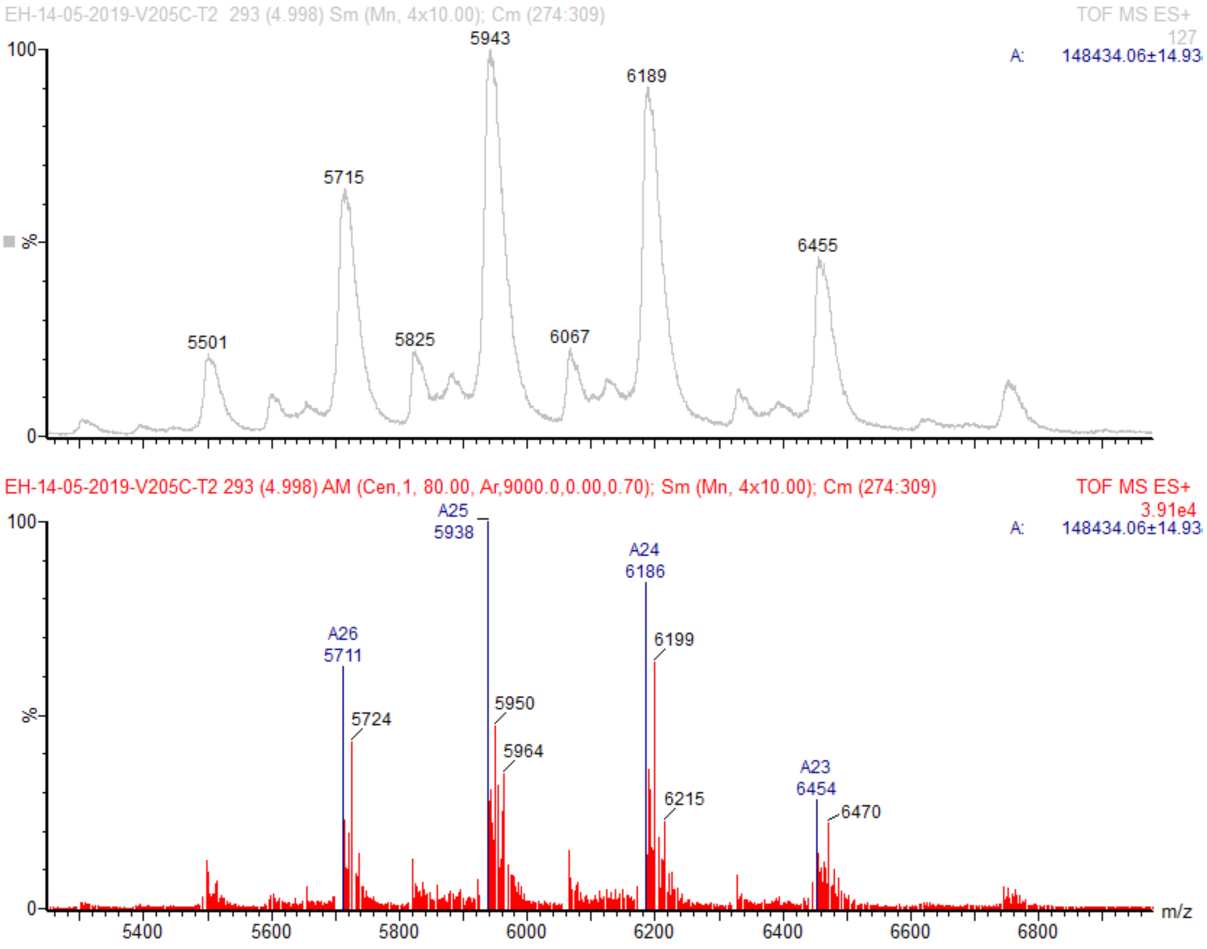

Figure 74. Native-MS spectrum of Gem-LC-V205C antibody. Obs.148434.06 ± 15. Exp.148338.

Gem-LC-V205C-BL

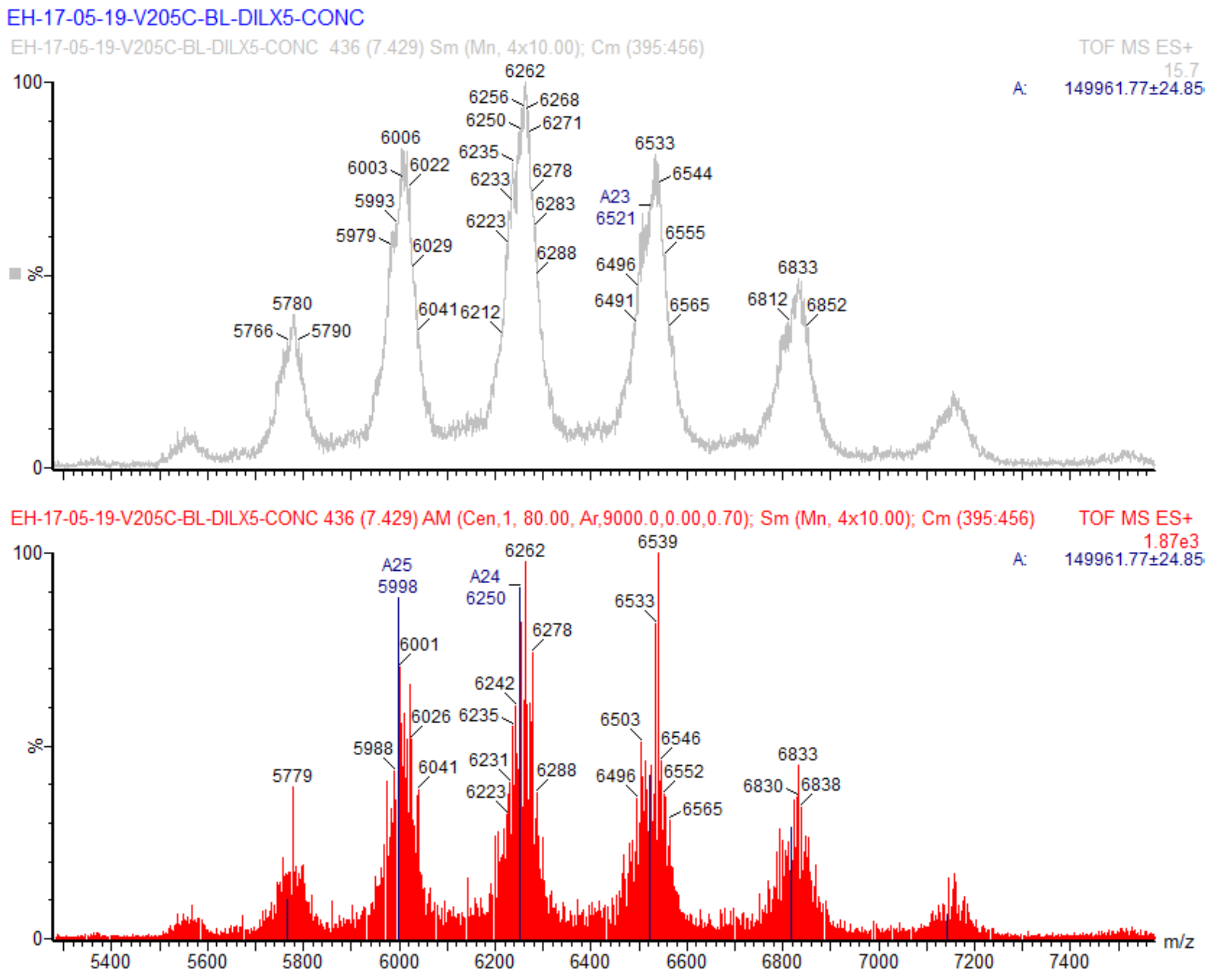

Figure 75. Native-MS spectrum of Gem-LC-V205C-BL ADC. Obs.149962 ± 25. Exp.149864. Exp. from addition (+1526) to obs. mass of unmodified antibody 149960**.**

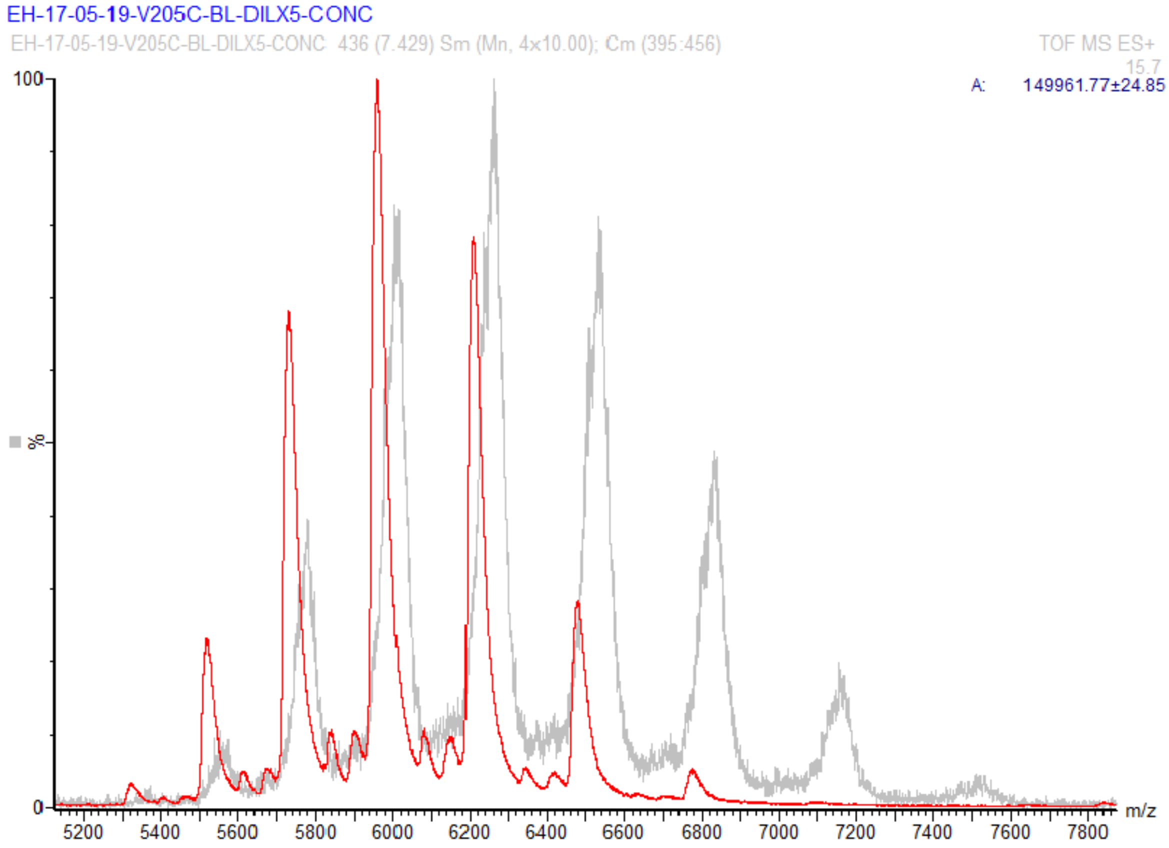

Figure 76. Comparison of Native-MS spectra of Gem-LC-V205C antibody (red line) and Gem-LC-V205C-BL ADC (grey line).

Gem-HC-S442C

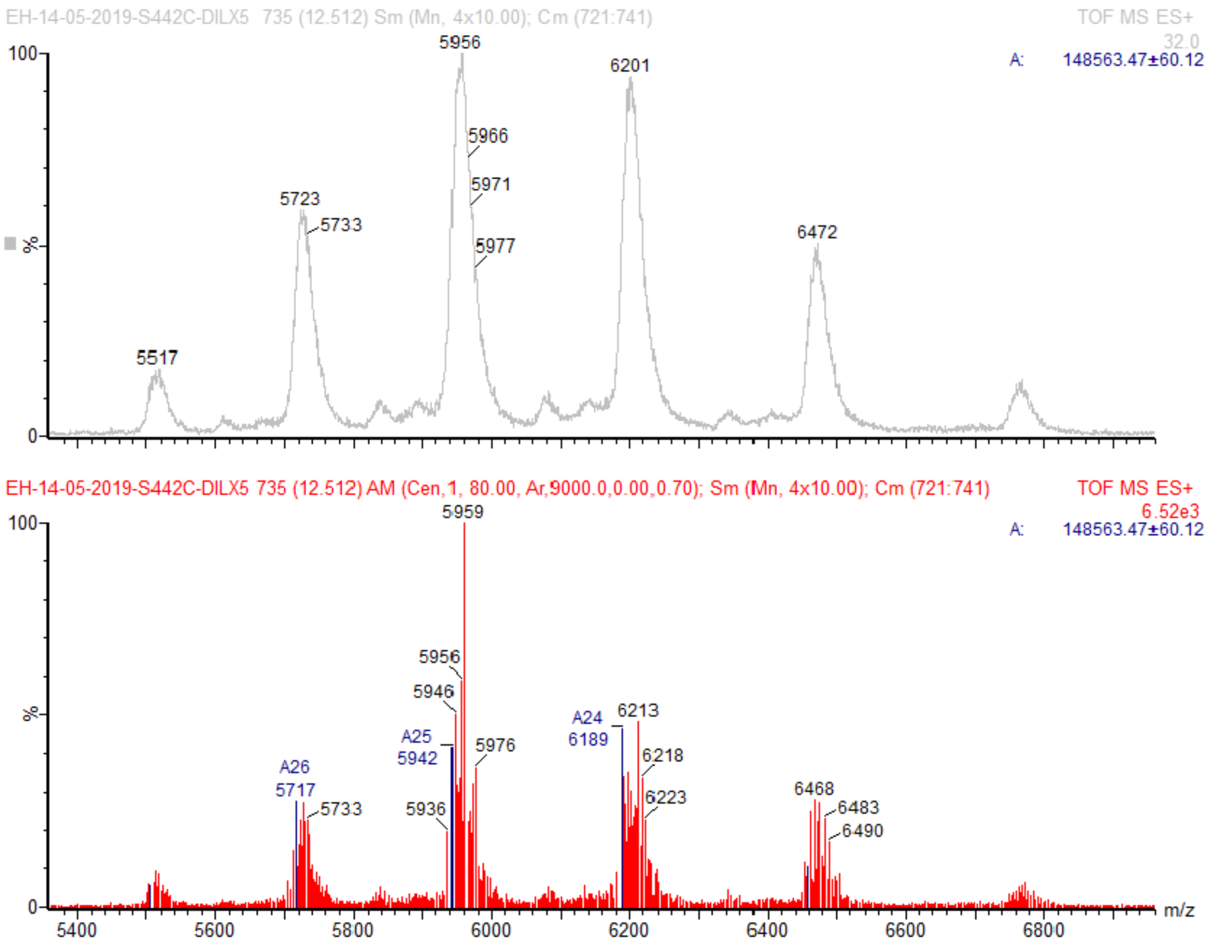

Figure 77. Native MS spectrum of Gem-HC-S442C. Obs.148563 ± 60. Exp.148362**.**

Gem-H-S442C-BL

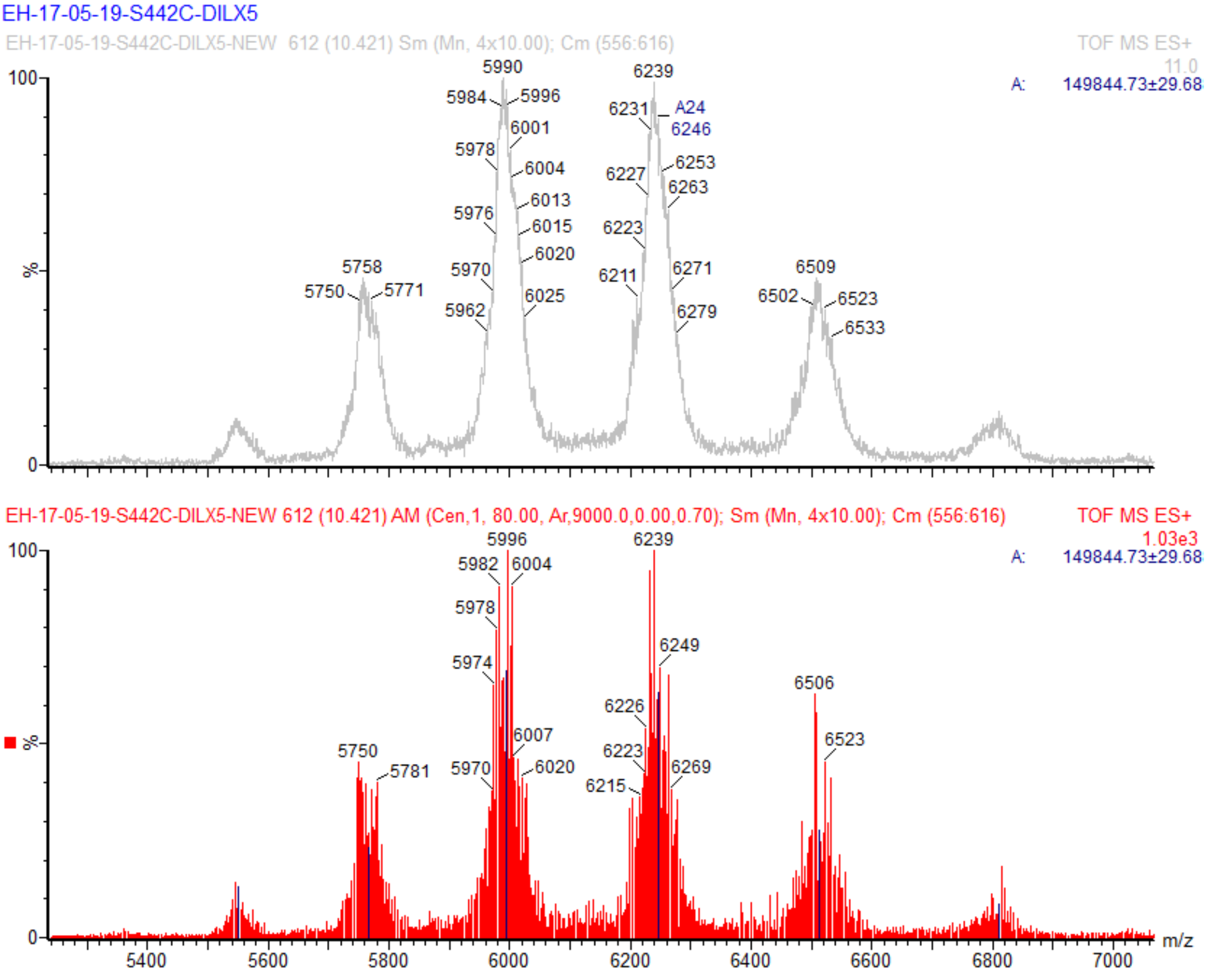

Figure 78. Native MS spectrum of Gem-HC-S442C-BL ADC. **Obs.149845 ± 30. Exp.148362. Exp. mass from addition (+ 1526) to obs. mass of unmodified antibody: 150089.**

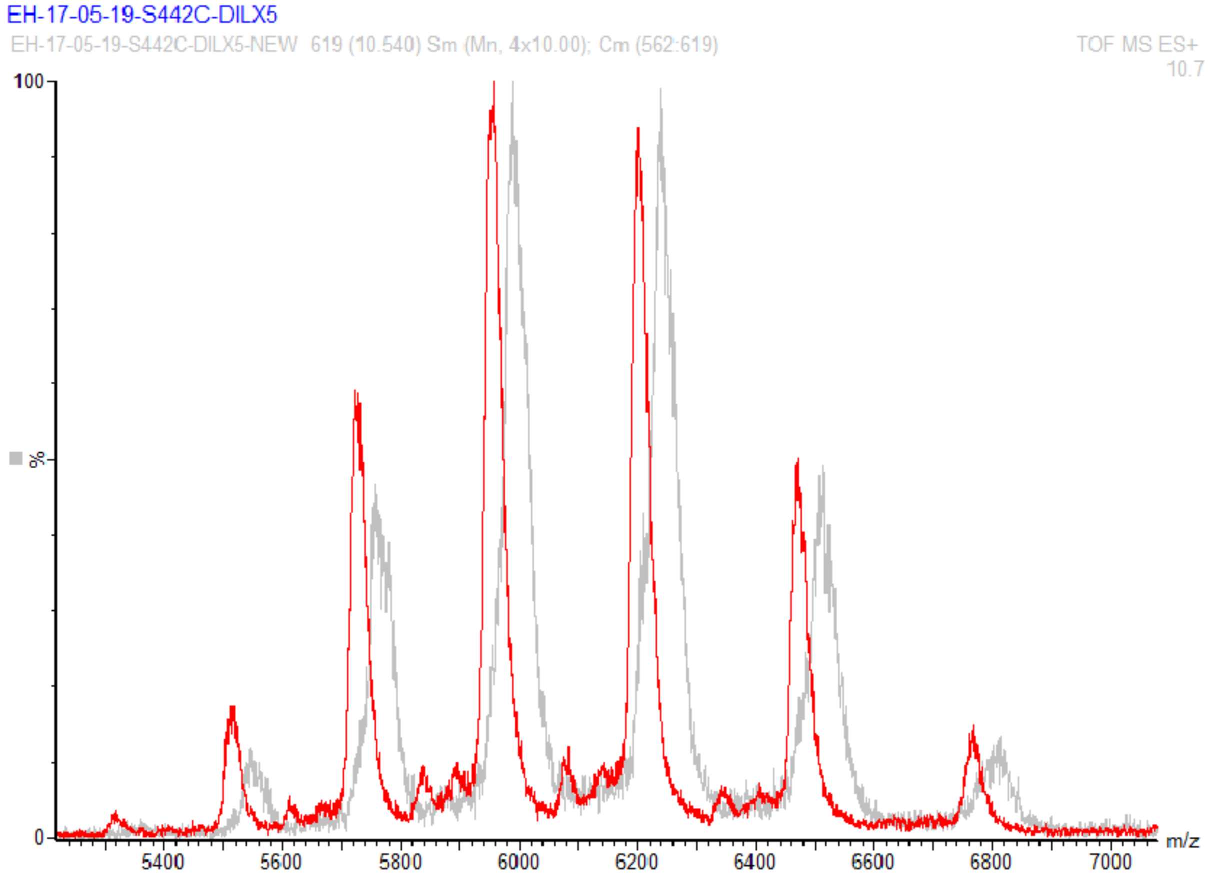

Figure 79. Comparison of native-MS spectra of Gem-HC-S442C antibody (red line) and Gem-HC-S442C-BL ADC (grey line).

Gem-HC-239iC

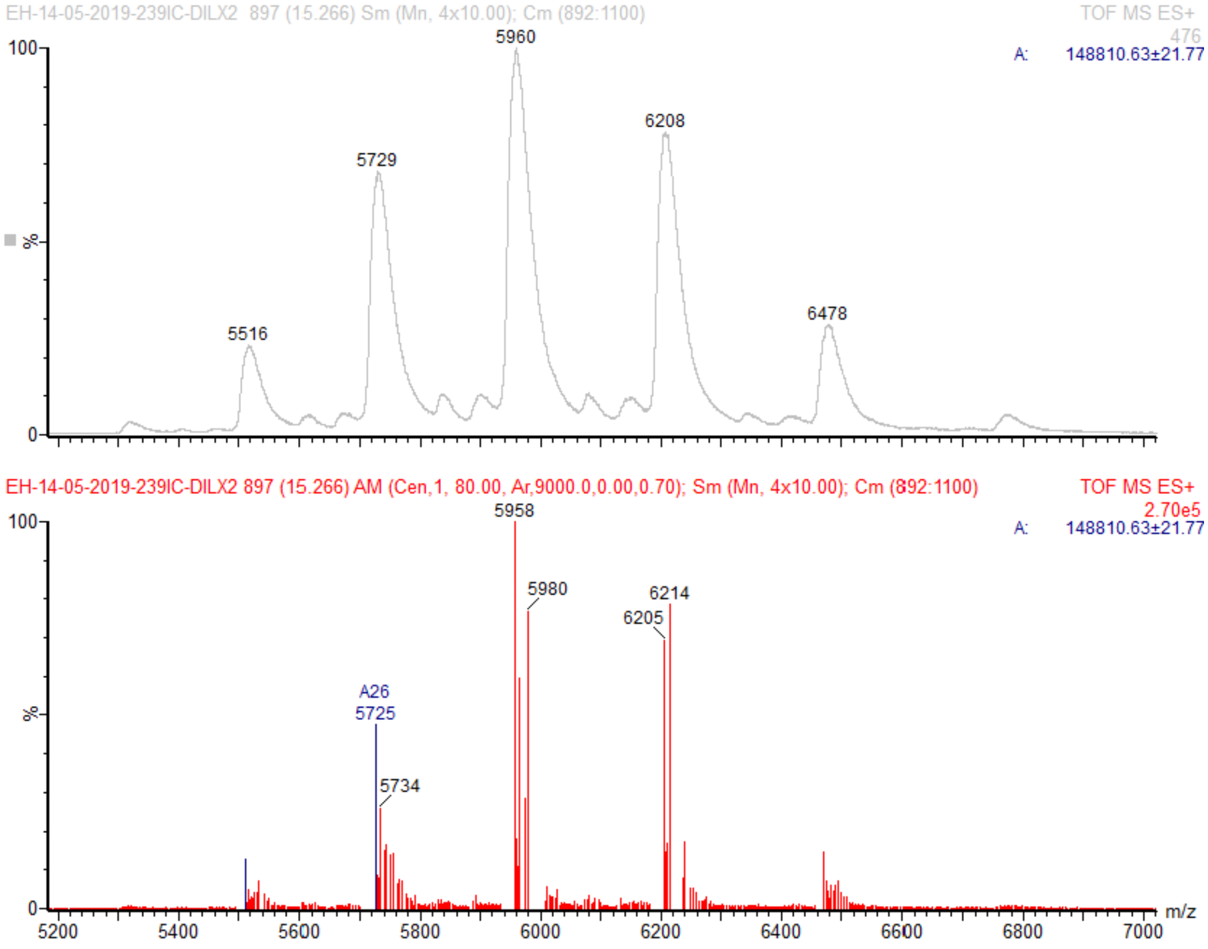

Figure 80. Native-MS spectrum of Gem-HC-239iC. Obs.148811 ± 22. Exp.148536.

Gem-HC-239iC-BL

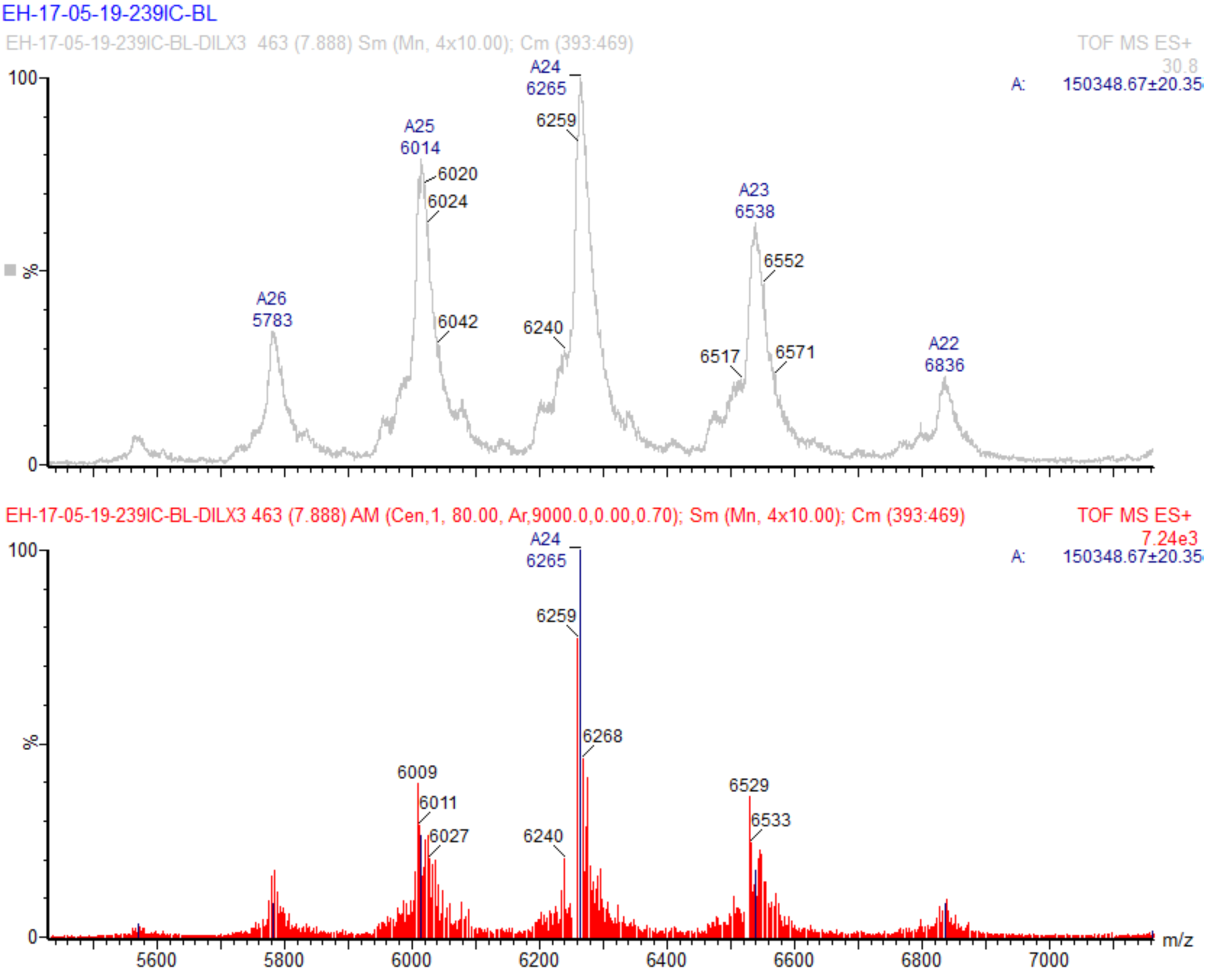

Figure 81. Native-MS spectrum of Gem-HC-239iC-BL ADC. Obs.150349 ± 20. Exp.150062. Exp. mass from addition (+ 1526) to obs. mass of unmodified antibody: 150336.

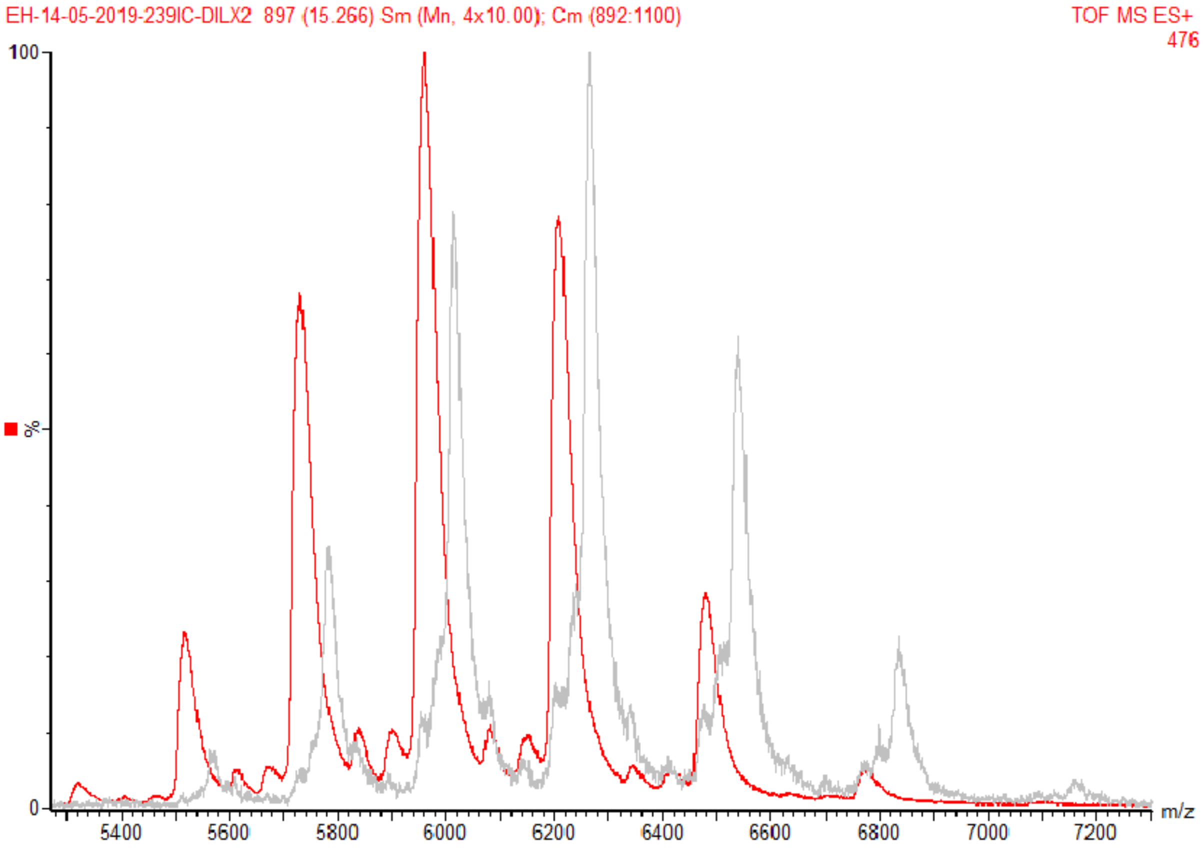

Figure 82. Comparison of Native-MS spectra of Gem-HC-239iC antibody (red line) and Gem-HC-239iC-BL ADC (grey line).

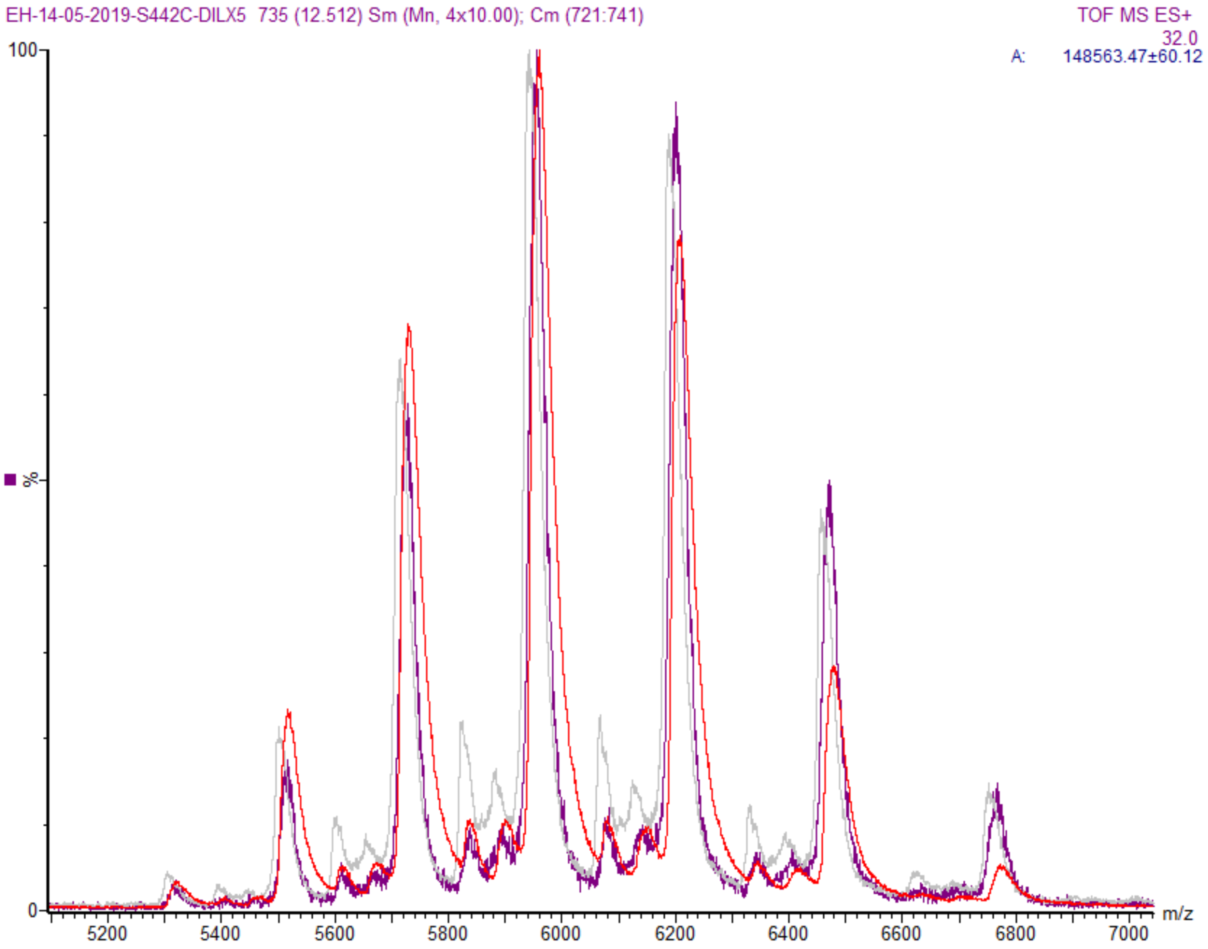

Figure 83. Comparison of Native-MS spectra of Gem-LC-V205C, Gem-HC-S442C and Gem-HC-239iC antibodies.

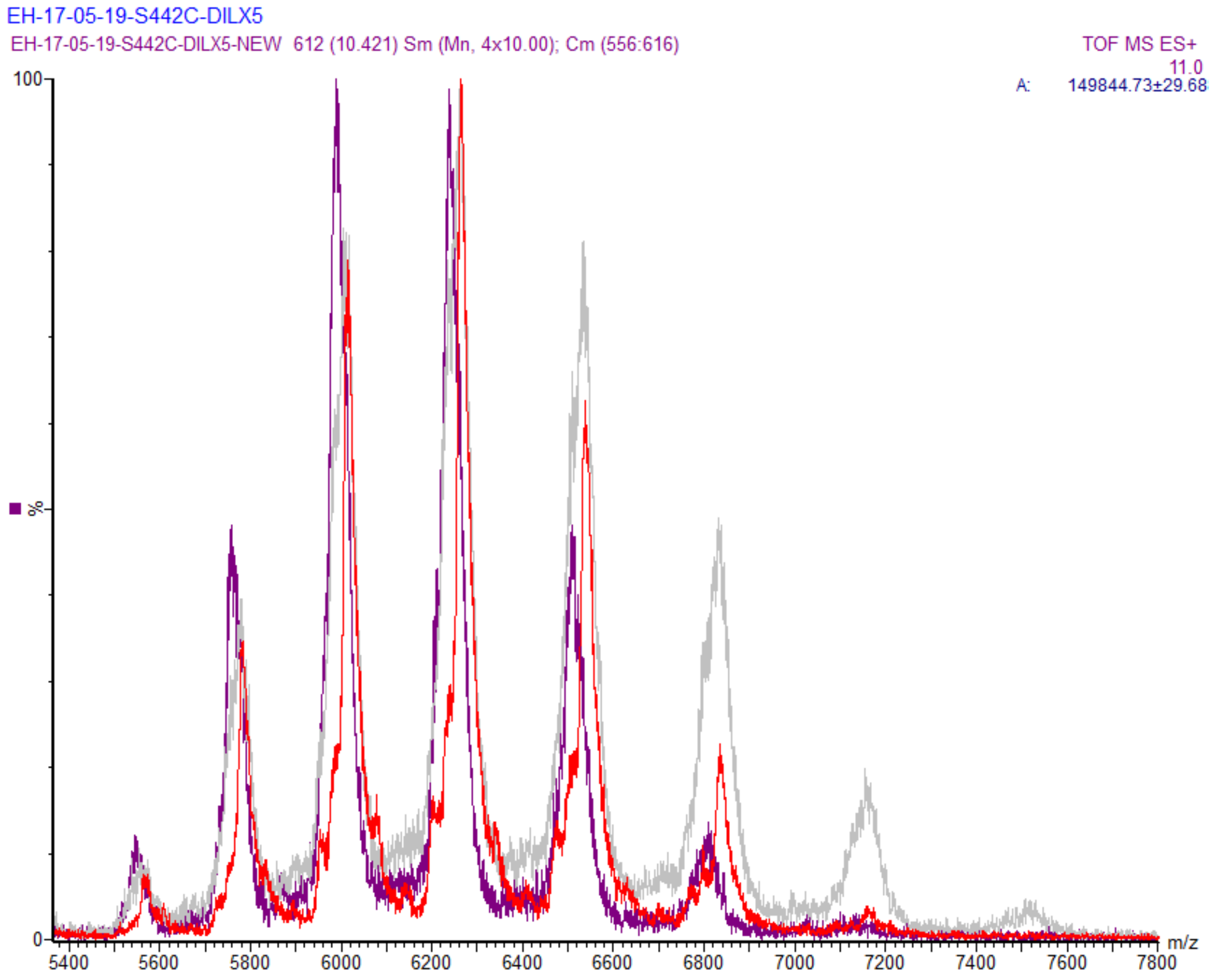

Figure 84. Comparison of Native-MS spectra of Gem-LC-V205C-BL, Gem-HC-S442C-BL and Gem-HC-239iC-BL ADCs.

Conjugate integrity of ADCs

Gem-LC-V205C-BL

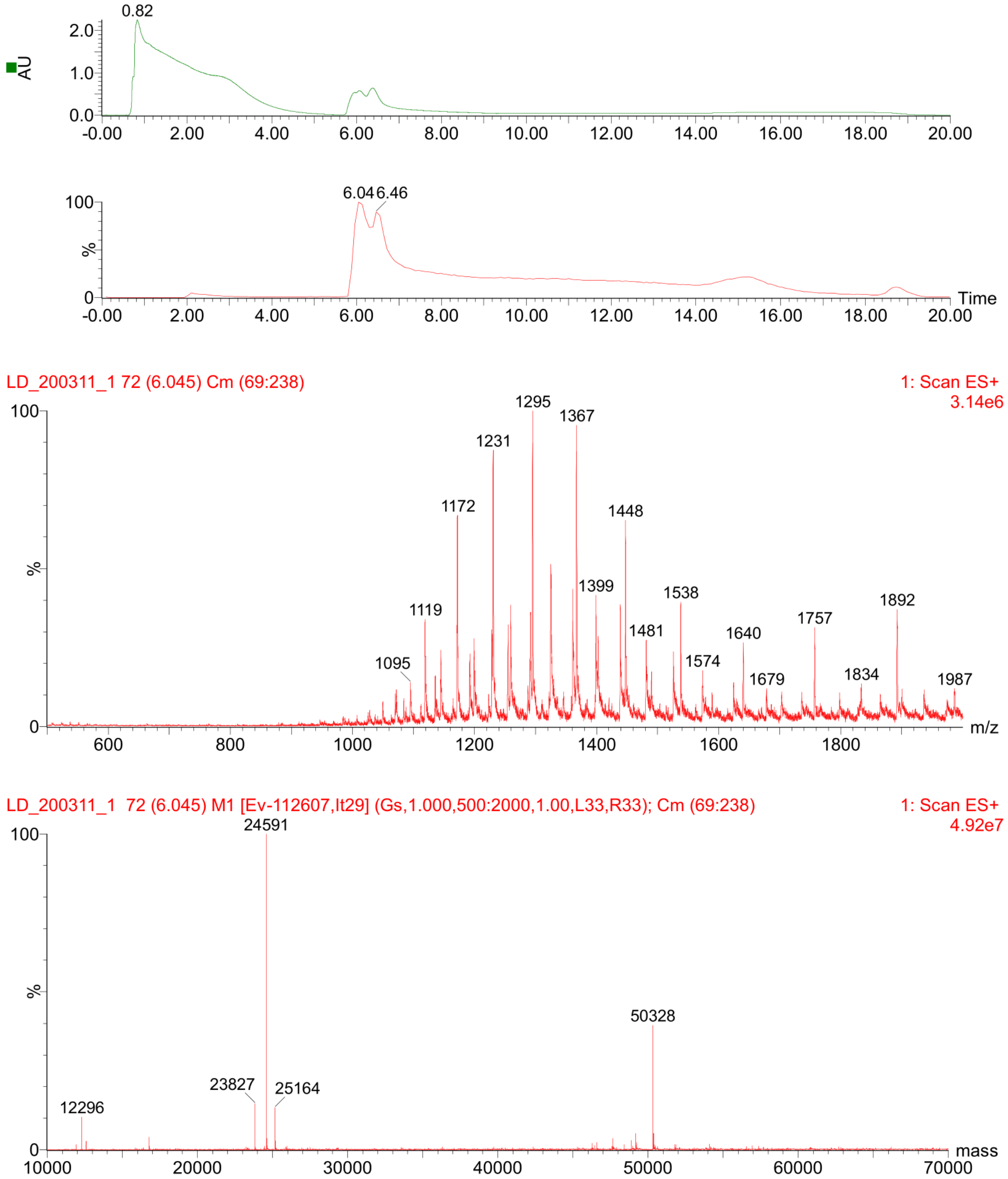

Figure 85. Gem-LC-V205C-BL following incubation in PBS pH 7.4, 37°C for 48 h. Exp. 23833 (LC unmodified), 24596 (LC modified), 50336 (HC unmodified). Obs. 23827 (LC), 24591 (LC modified), 25164 (artefact from heavy chain, 50328 / 2 = 25164), 50328 (HC unmodified). Conversion estimate > 87 %.

Gem-HC-S442C-BL

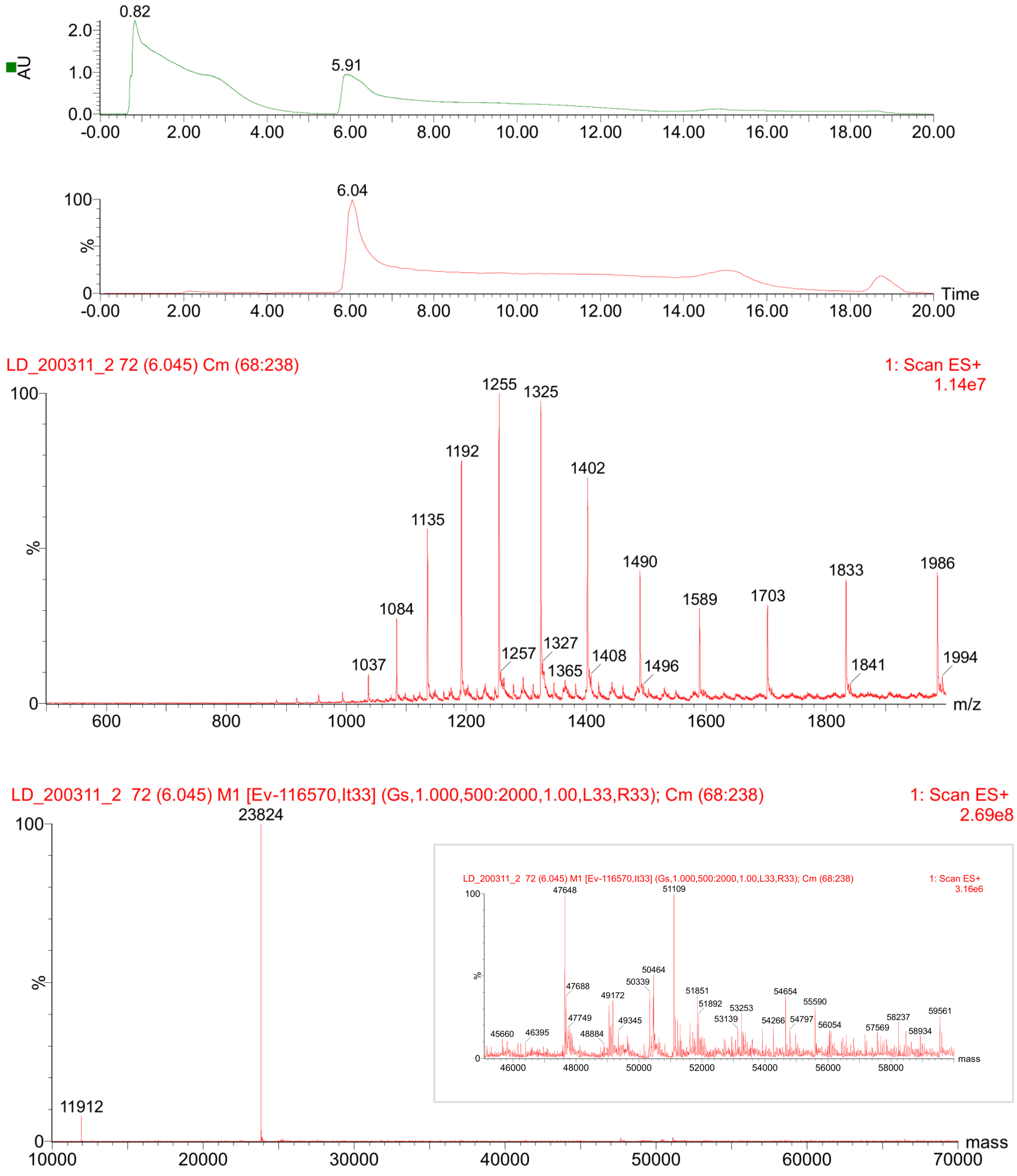

Figure 86. Gem-HC-S442C-BL following incubation in PBS pH 7.4, 37 °C for 48 h. Exp. 23829 (LC unmodified), 50352 (HC unmodified), 51115 (HC modified). Obs. 23824 (LC unmodified), 47648 (artefact from light chain, 23824 x 2 = 47648), 50339 (HC unmodified), 51109 (HC modified). Conversion estimate: 71%

Gem-HC-239iC-BL

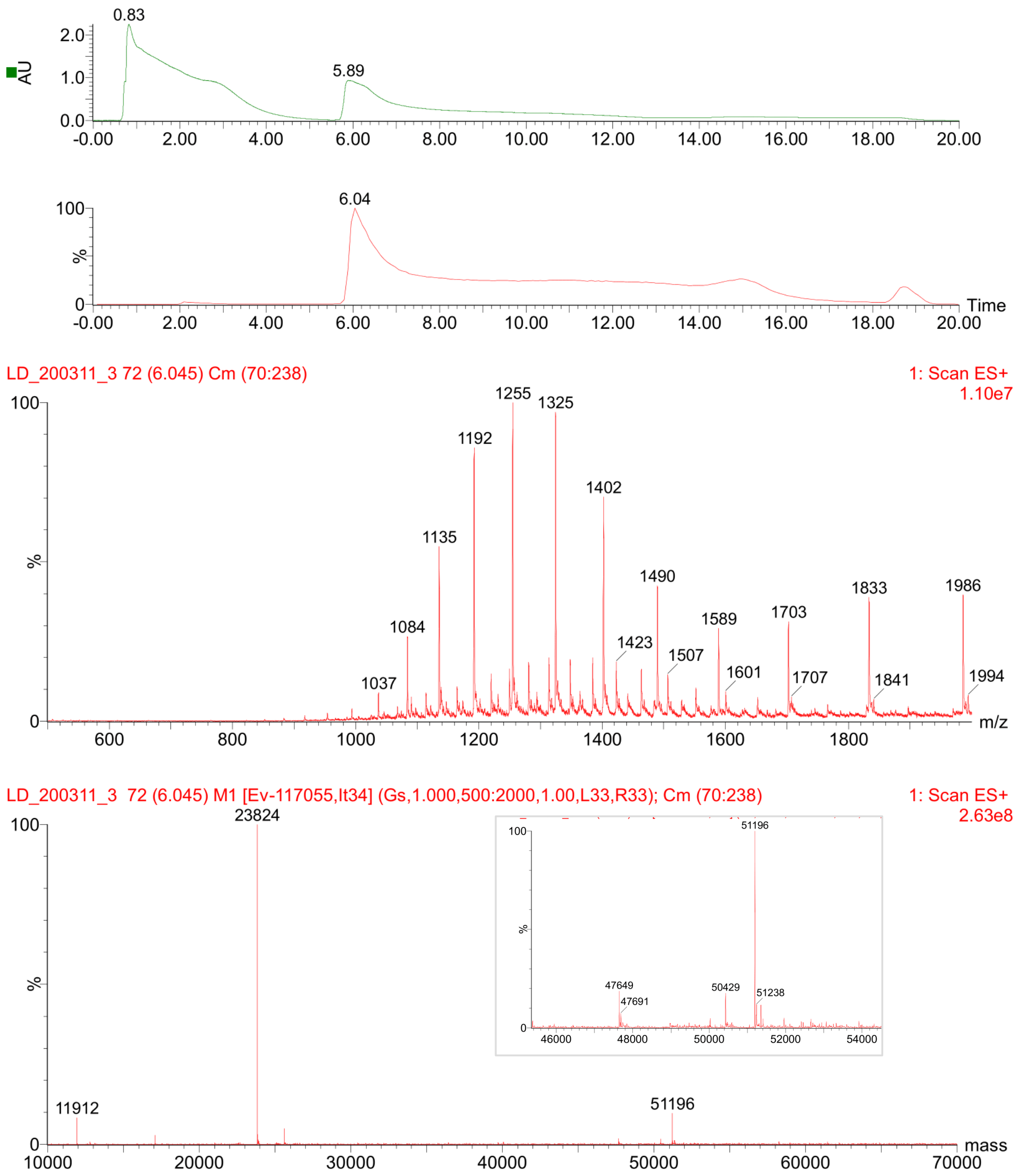

Figure 87. **Gem-HC-239iC-BL following incubation in PBS pH 7.4, 37°C for 48 h**. Exp. 23829 (LC unmodified), 50439 (HC unmodified), 51202 (HC modified). Obs. 23824 (LC unmodified), 47649 (artefact from light chain, 23824 x 2 = 47648), 50429 (HC unmodified), 51196 (HC modified). Conversion estimate: 83%.

Gem-LC-V205C-BL

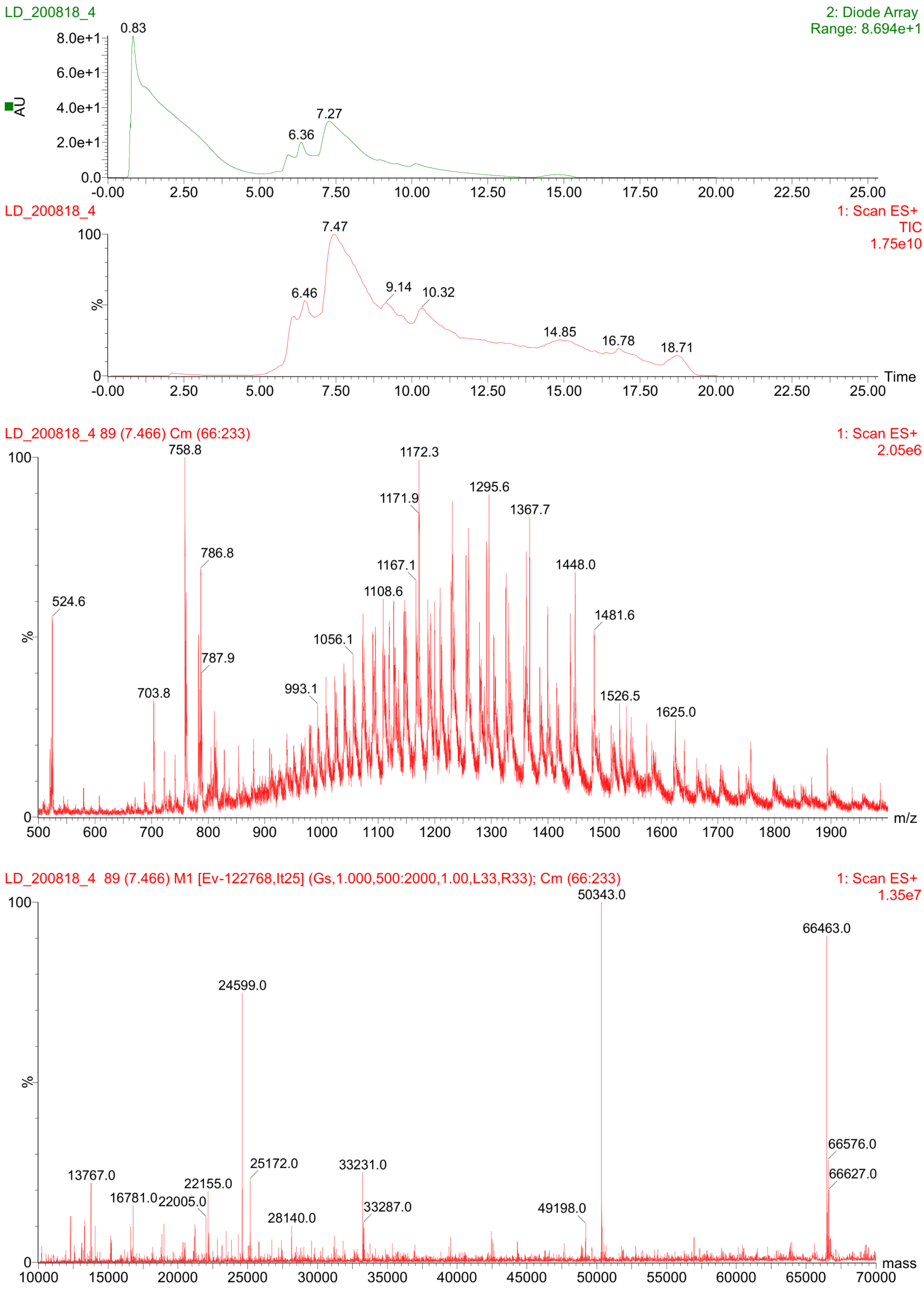

Figure 88. Gem-LC-V205C-BL conjugate integrity ADCs following incubation in 10% human serum at 37°C for 48 h. Exp. 23833 (LC unmodified), 24596 (LC modified), 50336 (HC unmodified). Obs. 24599 (LC modified), 50343 (HC unmodified). Conversion estimate >75%.

Gem-HC-S442C-BL

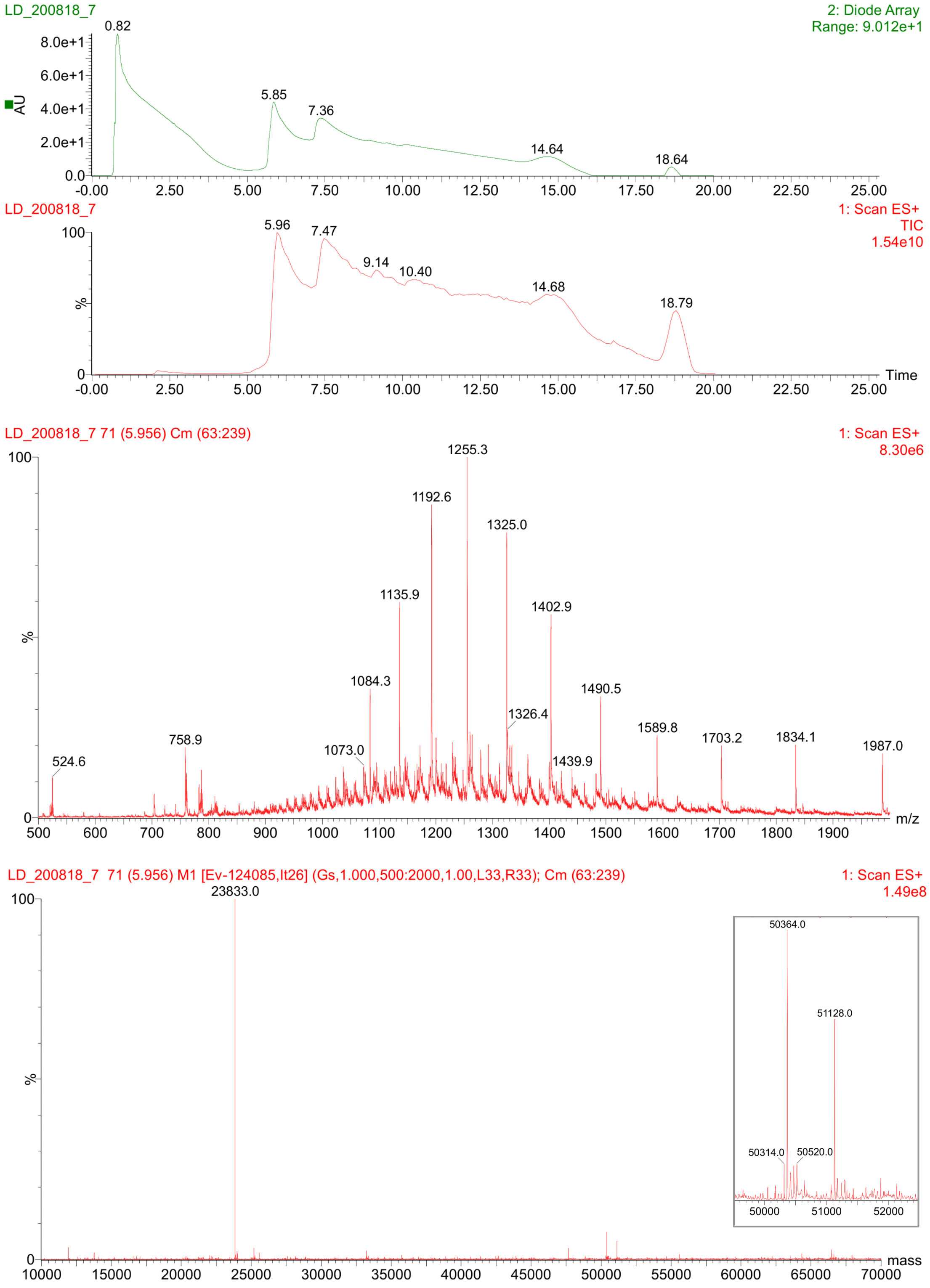

Figure 89. Gem-HC-S442C-BL conjugate integrity ADCs following incubation in 10% human serum at 37°C for 48 h. Exp. 23829 (LC unmodified), 50352 (HC unmodified), 51115 (HC modified). Obs. 23833 (LC unmodified), 50364 (HC unmodified), 51128 (HC modified). Conversion estimate: 41%.

Gem-HC-239iC-BL

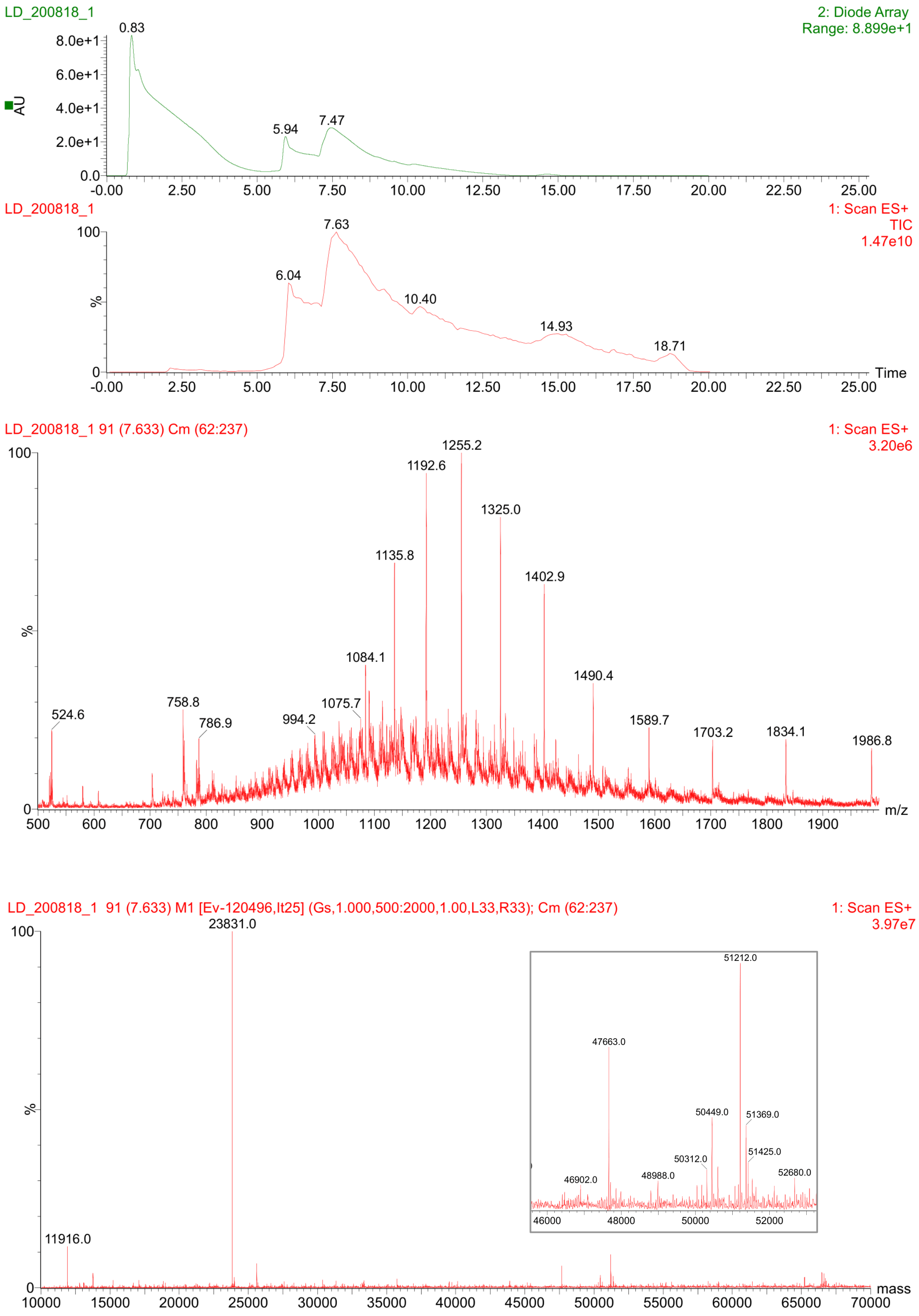

Figure 90. Gem-HC-239iC-BL conjugate integrity ADCs following incubation 10% human serum at 37°C for 48 h. Exp. 23829 (LC unmodified), 50439 (HC unmodified), 51202 (HC modified). Obs. 23831 (LC unmodified), 50449 (HC unmodified), 51212 (HC modified). Conversion estimate: 71%.

Gem-LC-V205C-BL

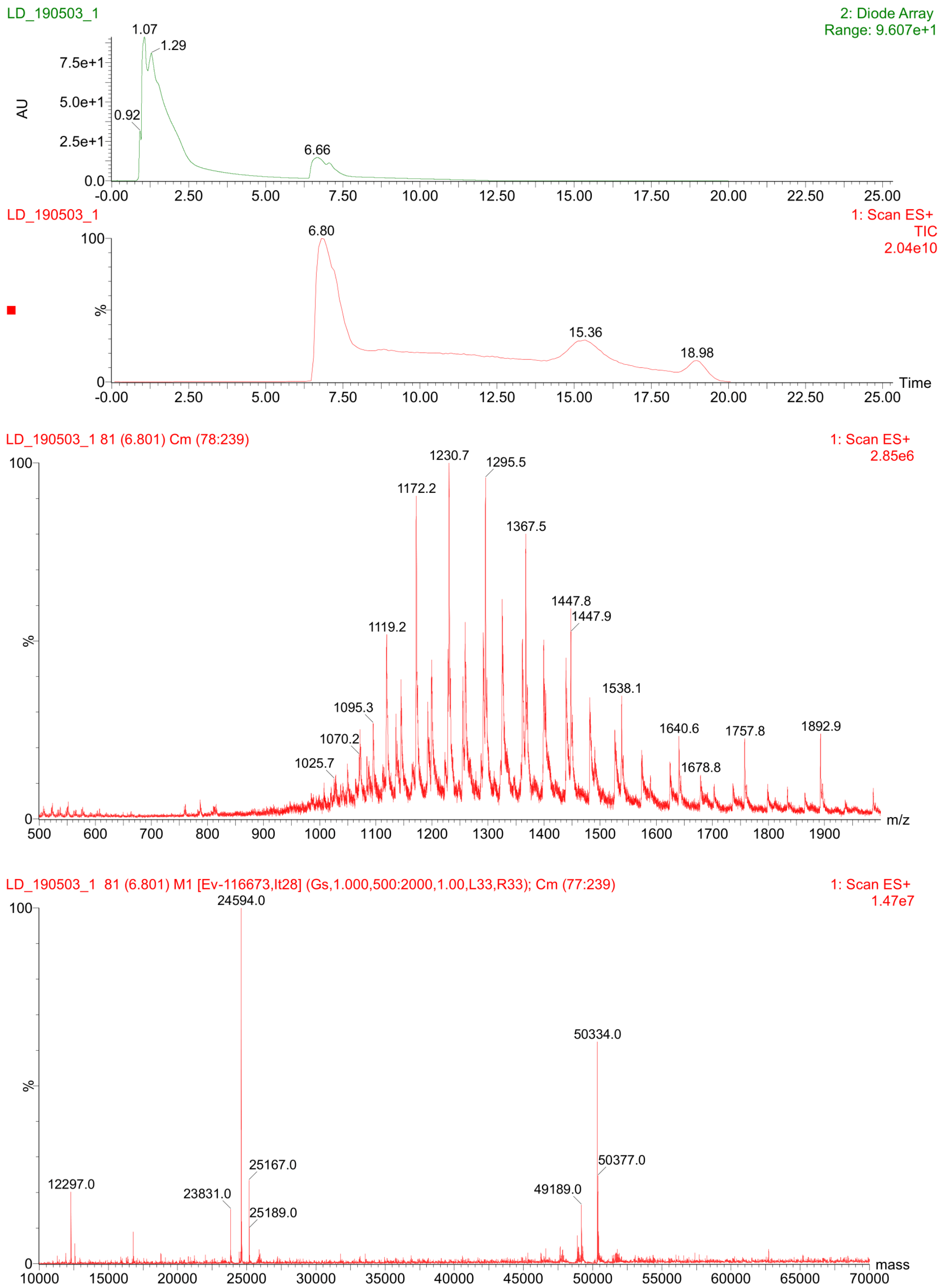

Figure 91. Reduced LCMS spectra of Gem-LC-V205C-BL prior to storage at 4 °C. Exp. 23833 (LC unmodified), 24596 (LC modified), 50336 (HC unmodified). Obs. 23831 (LC unmodified), 24594 (LC modified), 25167 (artefact from heavy chain, 50334 / 2 = 25167), 49189 (artefact from modified light chain, 24594 x 2 = 49188), 50334 (HC unmodified). Conversion estimate: 87%.

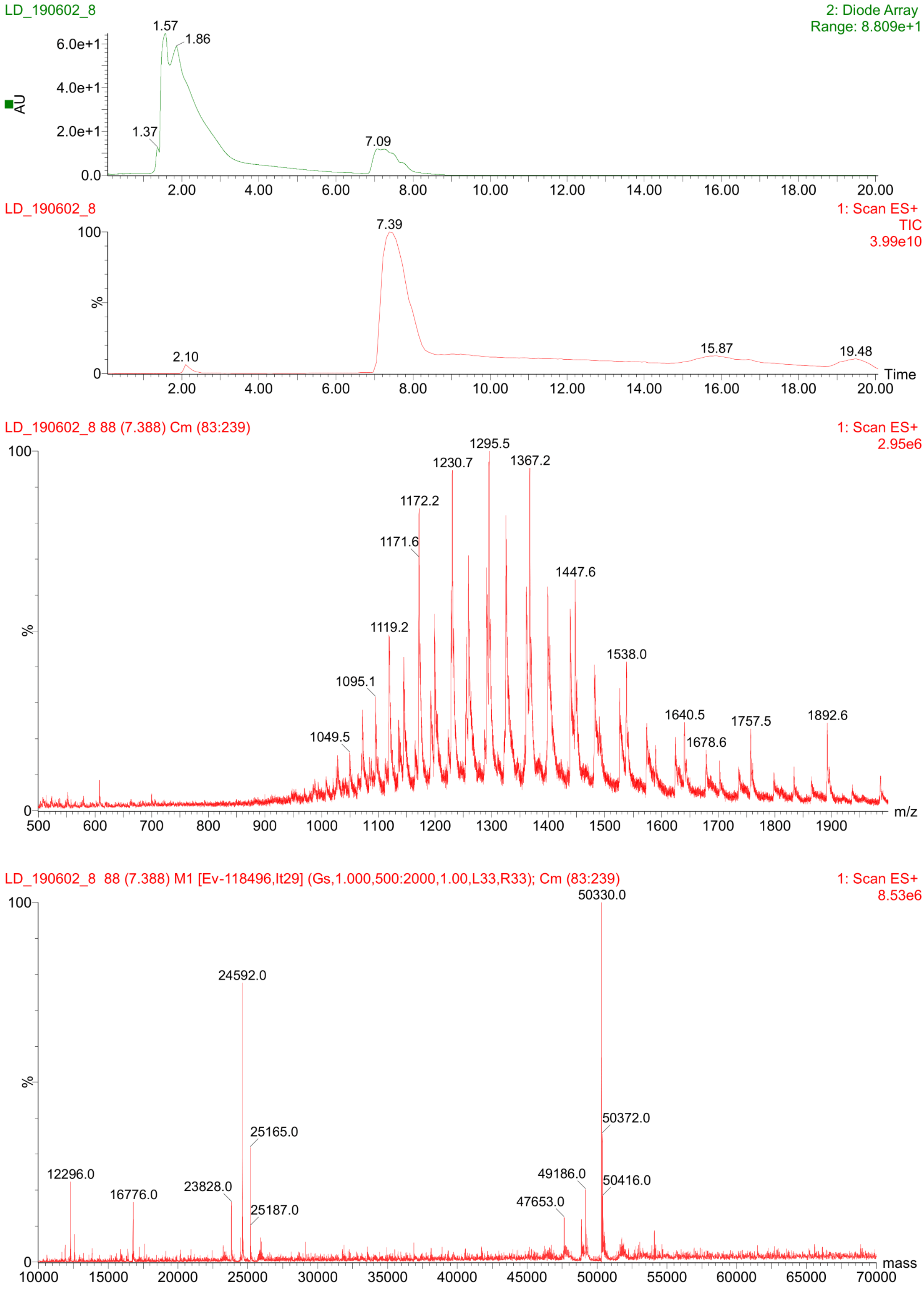

Figure 92. Gem-LC-V205C-BL after 4-weeks storage at 4 °C. Exp. 23833 (LC unmodified), 24596 (LC modified), 50336 (HC unmodified). Obs. 23828 (LC unmodified), 24592 (LC modified), 25165 (artefact from HC, 50330 / 2 = 25165), 47653 (artefact from unmodified LC, 47653 / 2 = 23827), 49186 (artefact from modified LC, 24592 x 2 = 49187), 50330 (HC unmodified). Conversion estimate: 81%.

Gem-HC-S442C-BL

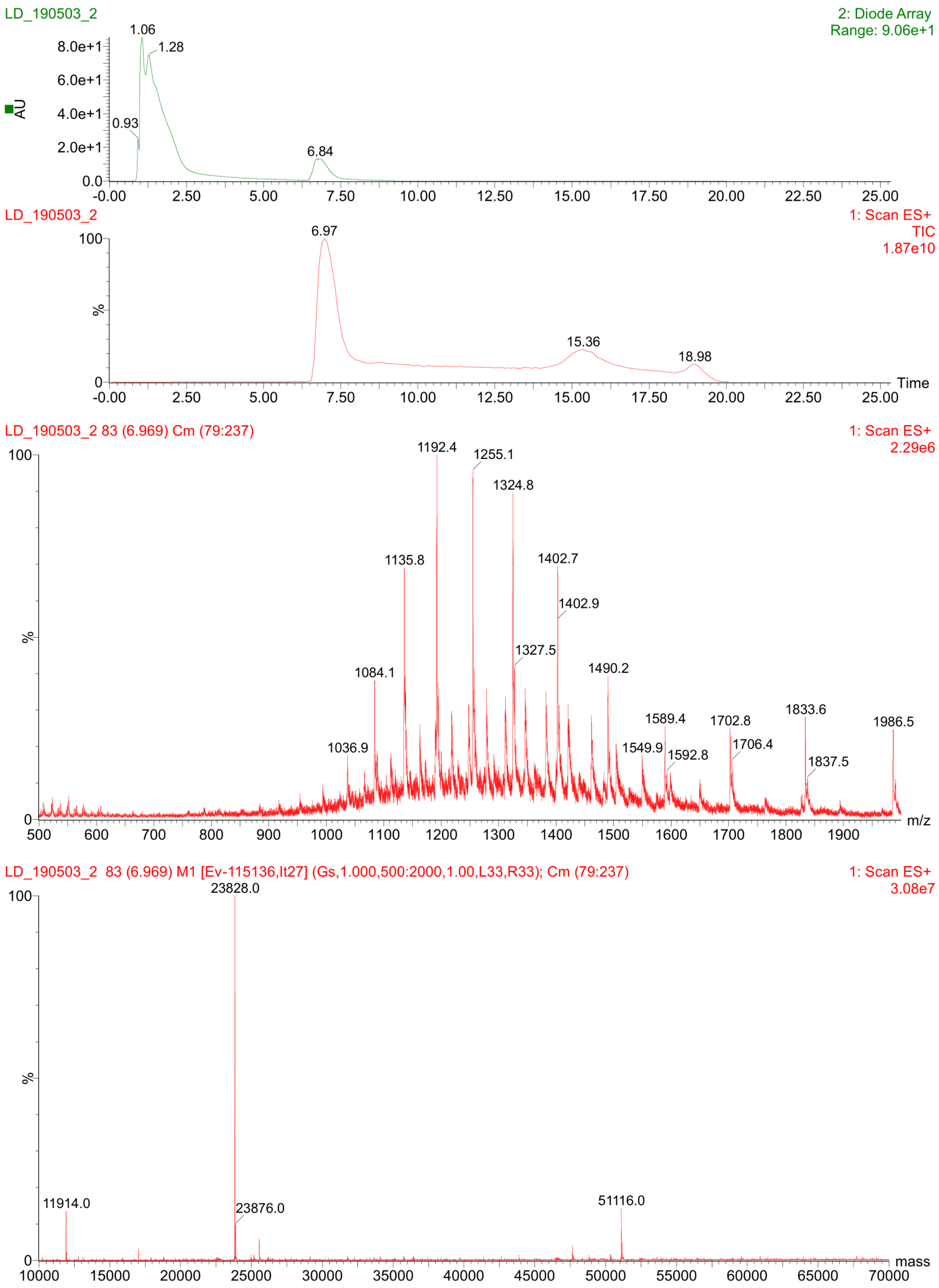

Figure 93. Reduced LCMS spectrum of Gem-HC-S442C-BL prior to storage at 4 °C. Exp. 23829 (LC unmodified), 51115 (HC modified). Obs. 23828 (LC unmodified), 51116 (HC modified). Conversion estimate: > 95%

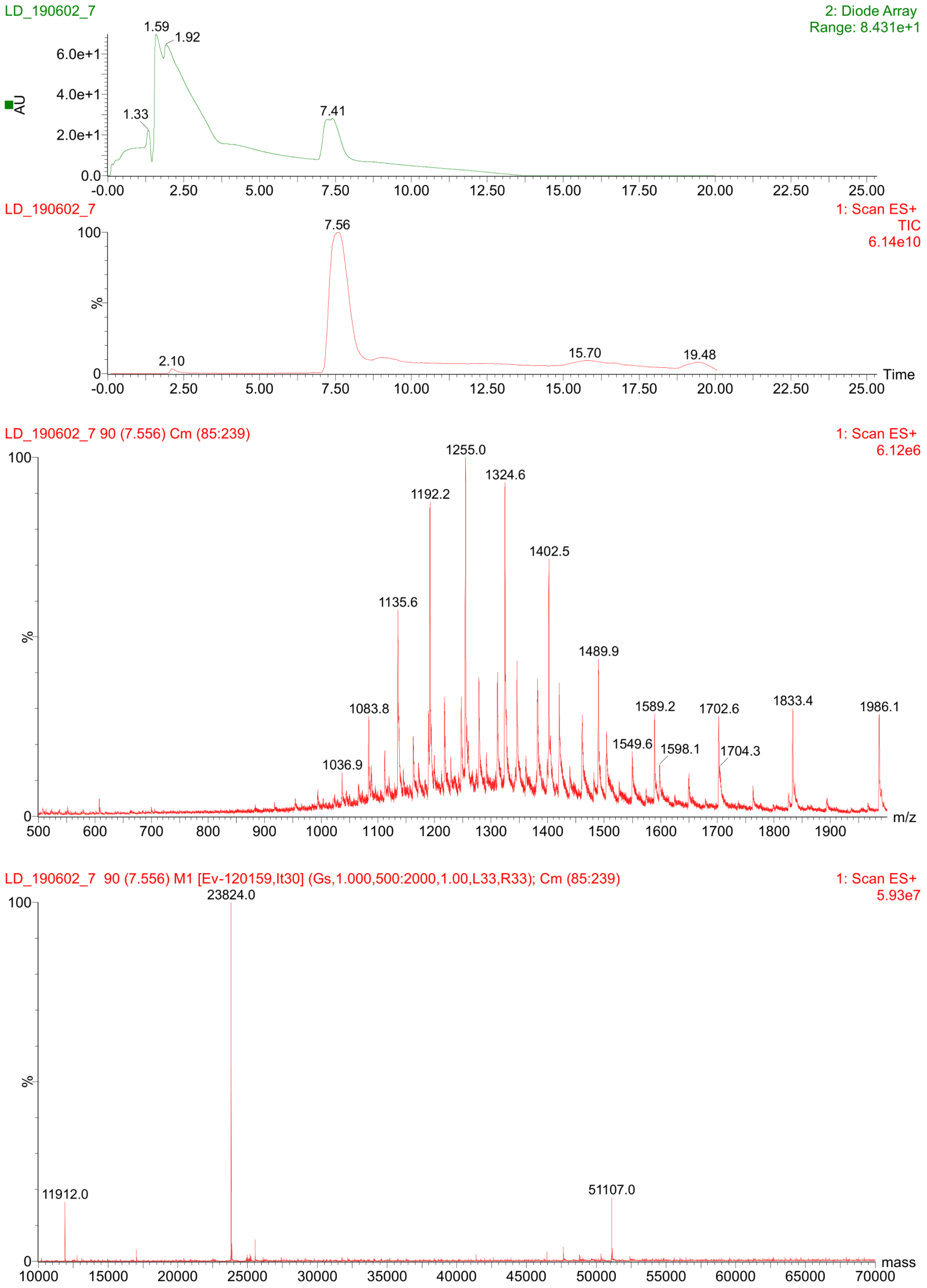

Figure 94. Reduced LCMS spectrum of Gem-HC-S442C-BL after 4-weeks storage at 4 °C. Exp. 23829 (LC unmodified), 51115 (HC modified). Obs. 23824 (LC unmodified), 51107 (HC modified). Conversion estimate: > 95%

Gem-HC-239iC-BL

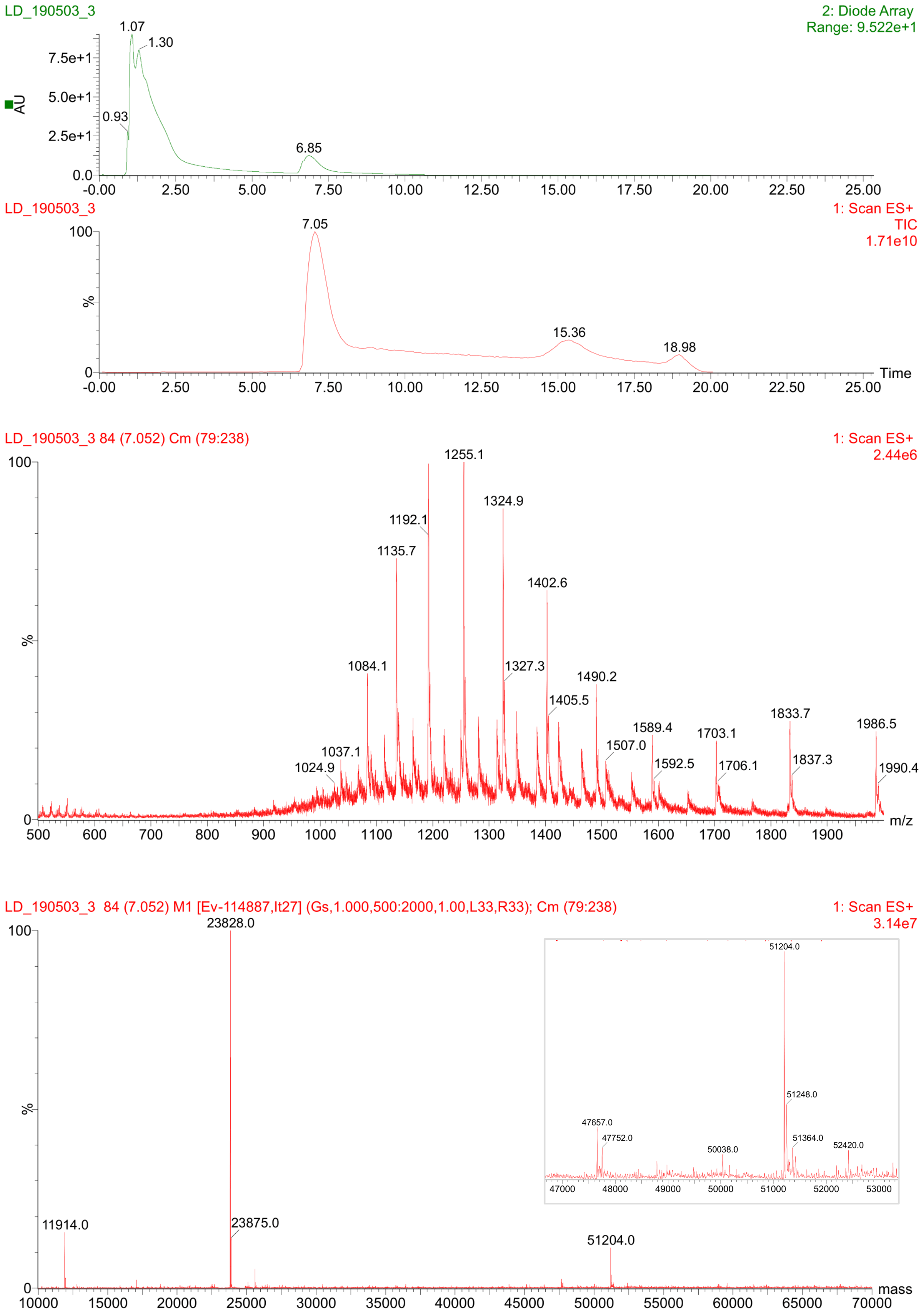

Figure 95. Reduced LCMS spectrum of Gem-HC-239iC-BL prior to storage at 4 °C. Exp. 23829 (LC unmodified), 51202 (HC modified). Obs. 23828 (LC unmodified), 51204 (HC modified). Conversion estimate: > 95%.

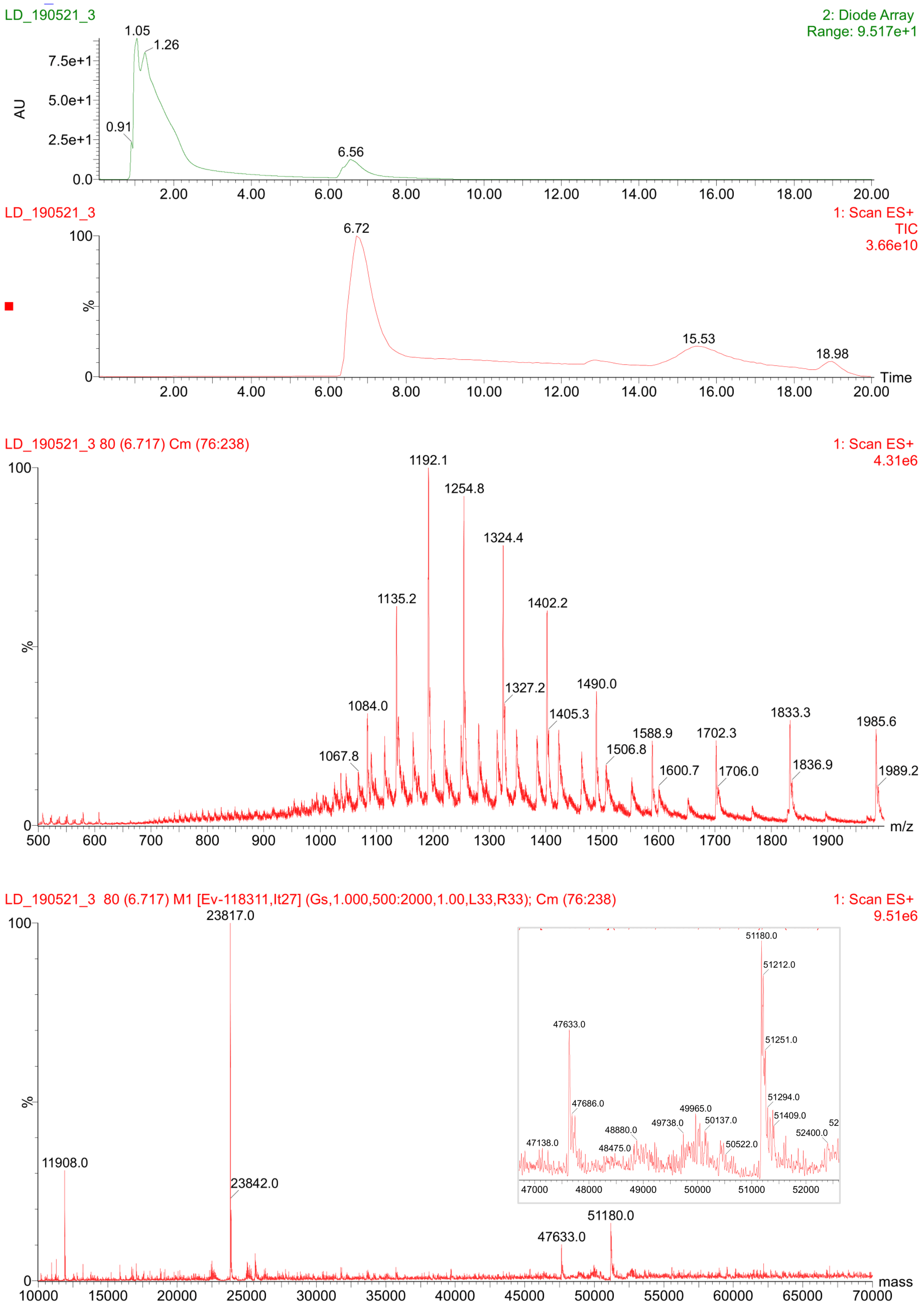

Figure 96. Reduced LCMS spectrum of Gem-HC-239iC-BL after 3-weeks storage at 4 °C. Exp. 23829 (LC unmodified), 51202 (HC modified). Obs. 23817 (LC unmodified), 51180 (HC modified). Conversion estimate: > 95%.

ADC Toxicity

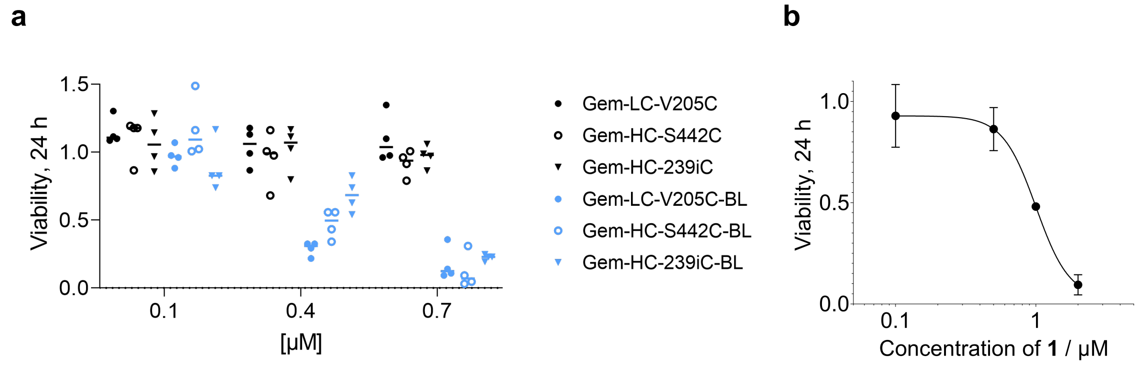

Figure 97. a, Viability of HL-60 cells treated with ADCs Gem-HC-239iC-BL, Gem-HC-S442C-BL and Gem-LC-V205C-BL, in comparison to the unconjugated parent antibody, after 24 h incubation. Line represents mean cell viability with error bar as ± SEM (n=3). This experiment was performed once due to limitations on antibody availability. **b,** Toxicity of β-lapachone 1 to HL-60 cells over 24 h, IC_{50} ~ 1 µM. Data are presented as mean values fit to 4-PL model with error bars representing ± SEM (n=3). In both **a** and **b**, cell viability was measured by counting of live cells in comparison to control untreated cells using the Trypan Blue exclusion method.

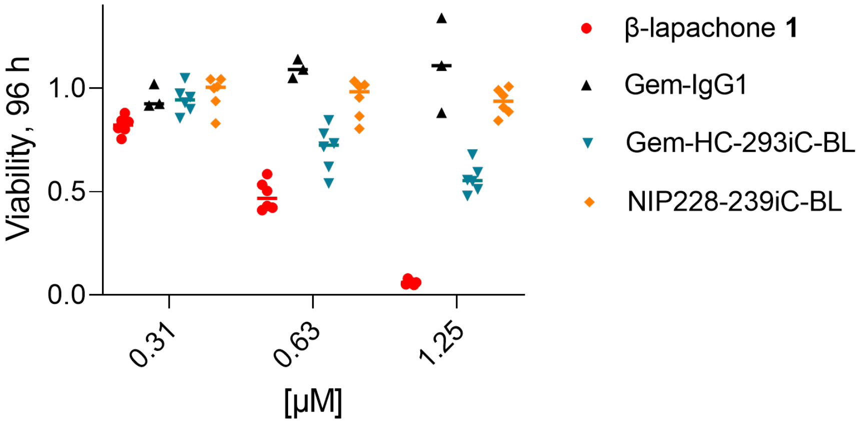

Figure 98. Toxicity of ADC Gem-HC-239iC-BL to HL-60 cells. a, Toxicity of ADC Gem-HC-239iC-BL, in comparison to non-internalising ADC control NIP228-HC-239iC-BL, β-lapachone, **1**, and unmodified native antibody control Gem-IgG1. Cell viability was measured after 96 h by counting of live cells in comparison to control untreated cells using the Trypan Blue exclusion method. Assay was performed in RPMI media + 5% human serum**.** Line represents mean cell viability with error bar as ± SEM (n=3 or 6). This experiment was performed once due to limitations on antibody availability. A repeat experiment using CellTiter-Glo® readout displayed some inconsistent results which we hypothesised was due to aggregation of the tested sample.

Morphology of HL-60 cells treated with ADCs

a Gem-HC-239iC 1 μM, 24 h

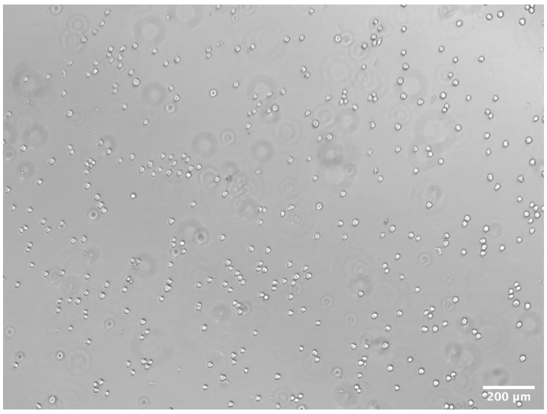

b Gem-HC-239iC-BL 0.7 μM 24 h

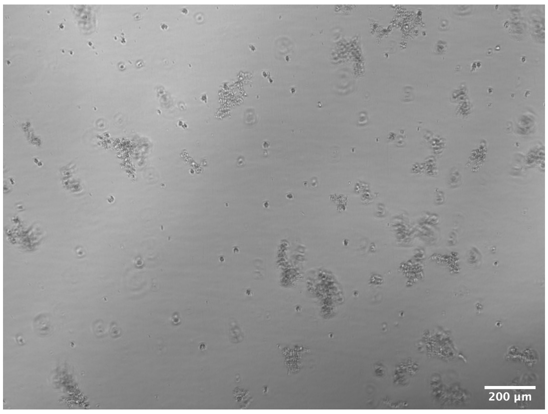

c Gem-HC-239iC-BL 0.4 μM, 24 h

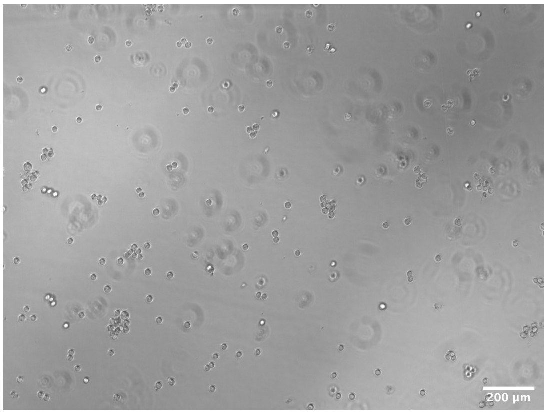

d Gem-HC-239iC-BL 0.1 μM, 24 h

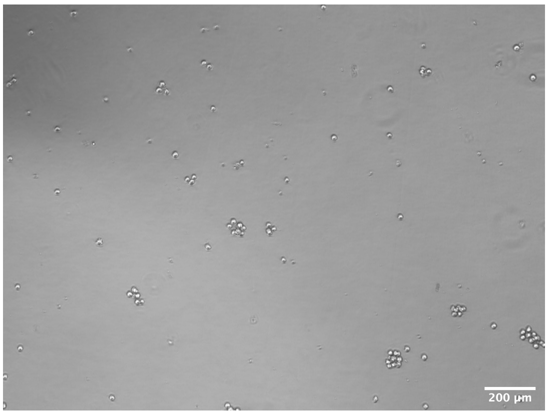

e β-lapachone 0.5 μM, 24 h

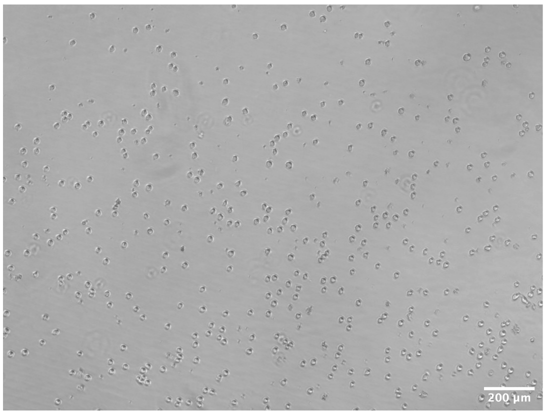

f β-lapachone 1 μM, 24 h,

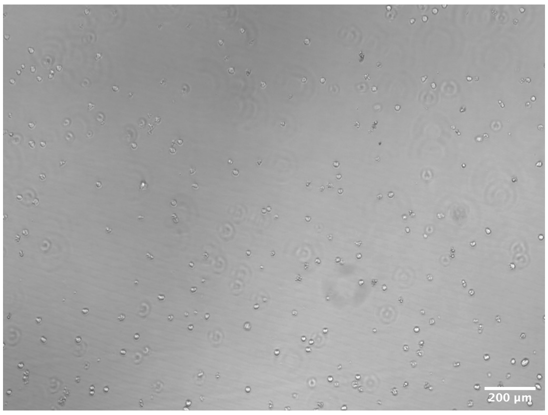

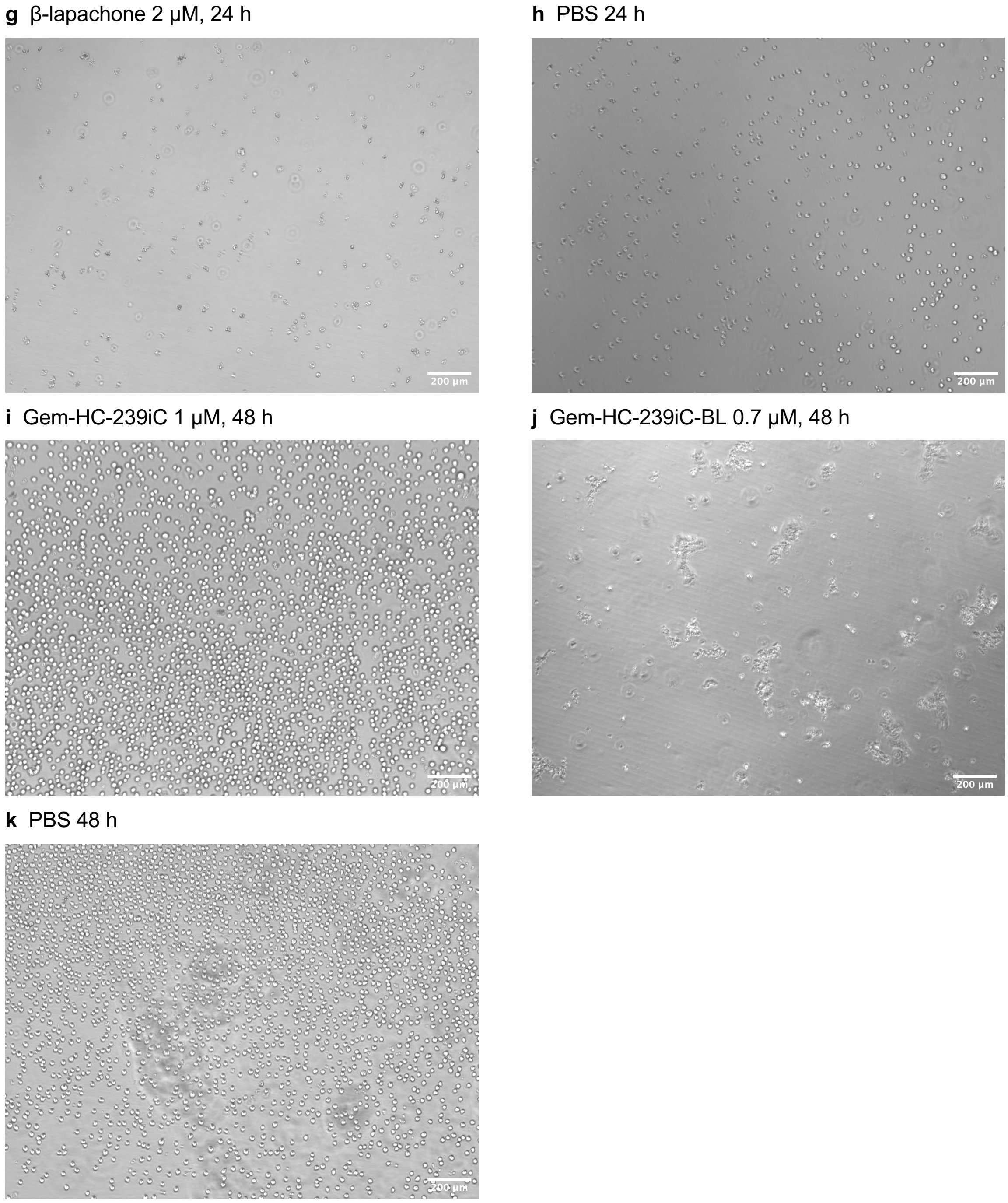

Figure 99. **Morphology of HL-60 cells treated with ADCs.** Images of HL-60 AML cells after treatment of with conjugate Gem-HC-239iC-BL, with unconjugated antibody Gem-HC-239iC, with β-lapachone or with PBS. Scale bars: White bar bottom right, 200 µm with 10x objective.

Histology data from *in vivo* experiment

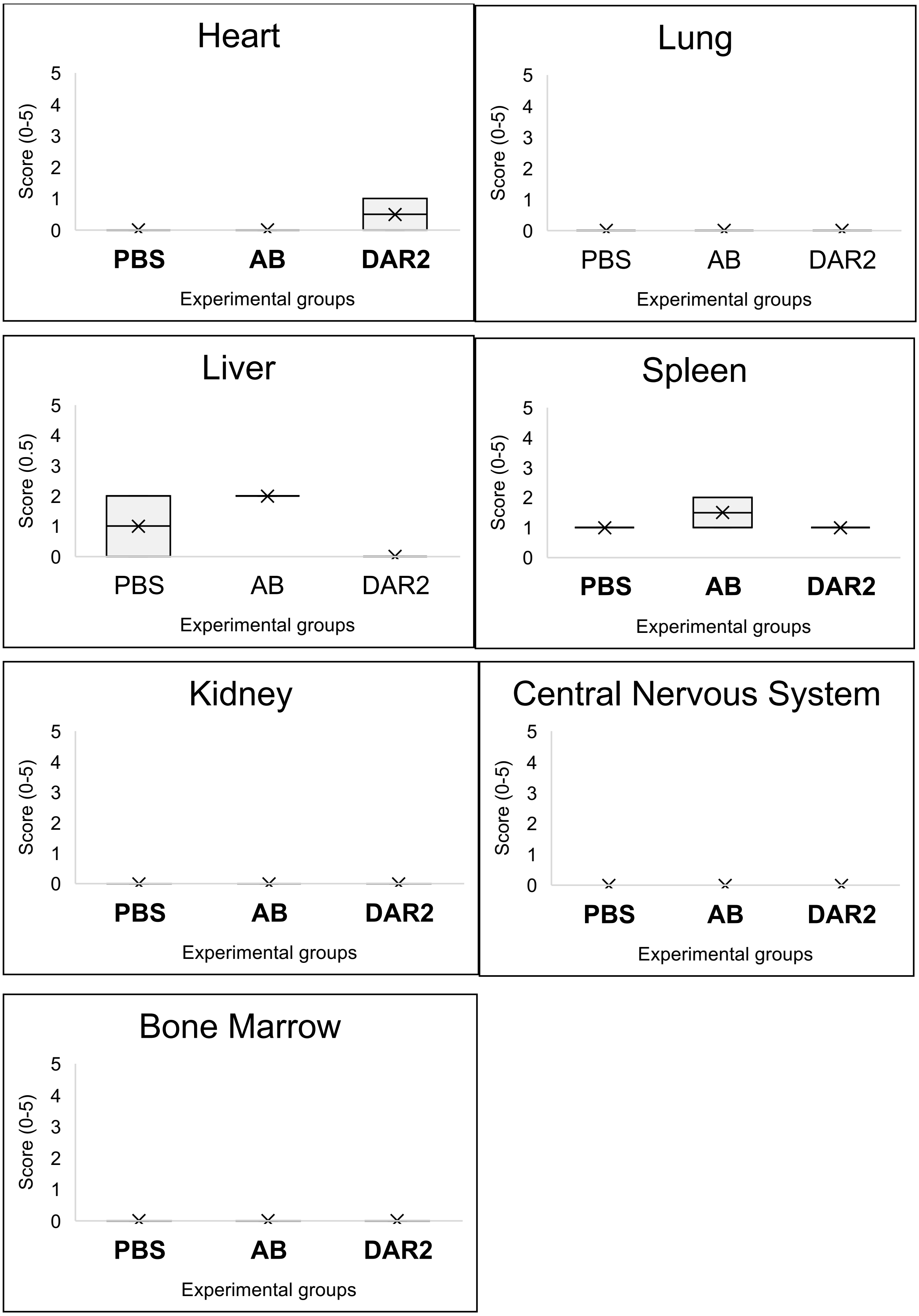

Figure 100. Histology summary.

Toxicologic analysis: No signs of toxicity or other significant changes were seen in the heart, lung, liver, kidney, spleen, bone marrow and central nervous system of mice treated with the test compounds. All of the observed lesions are generally considered background lesions* but should be diagnosed for completeness.

Oncology report: No metastasis was observed in any of the groups (PBS (control), AB (Gem-IgG1) and DAR-2 ADC (Gem-HC-239iC-BL).

*background lesion: incidental lesion with no relation to the testing compound such as mononuclear inflammatory cell infiltration and minimal foci of hepatic lipidosis in the liver; hemosiderosis in the spleen and clear vacuoles in the heart cardiomyocytes

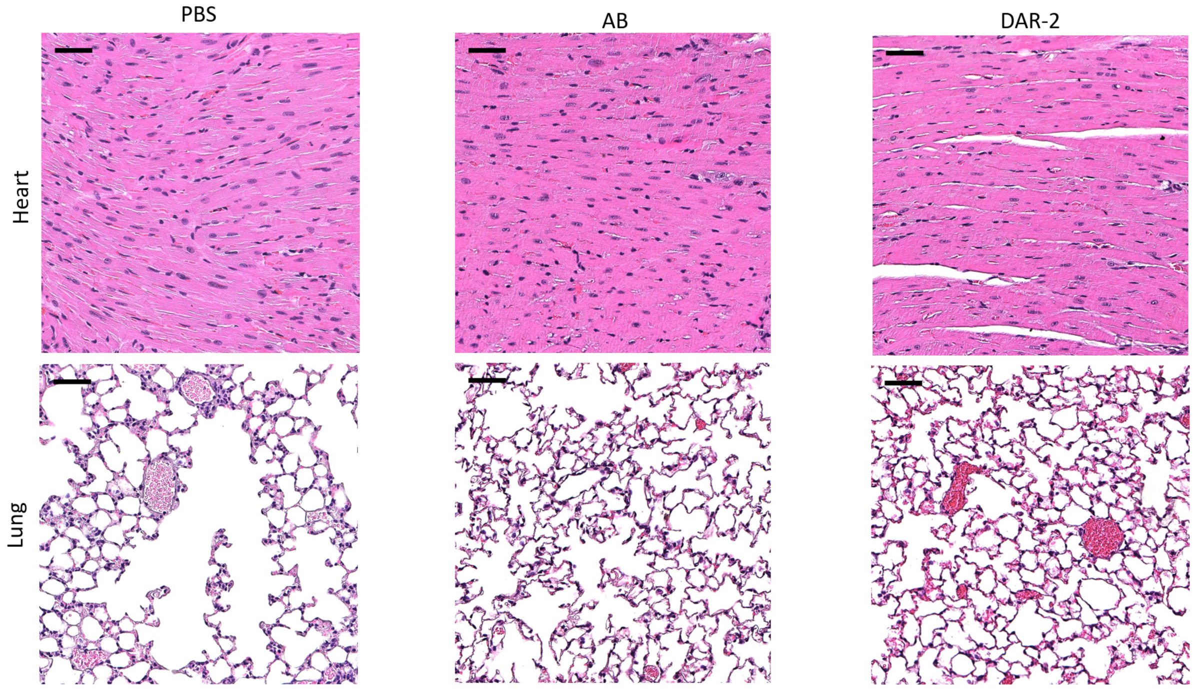
PBS
AB
DAR-2
Heart
Lung

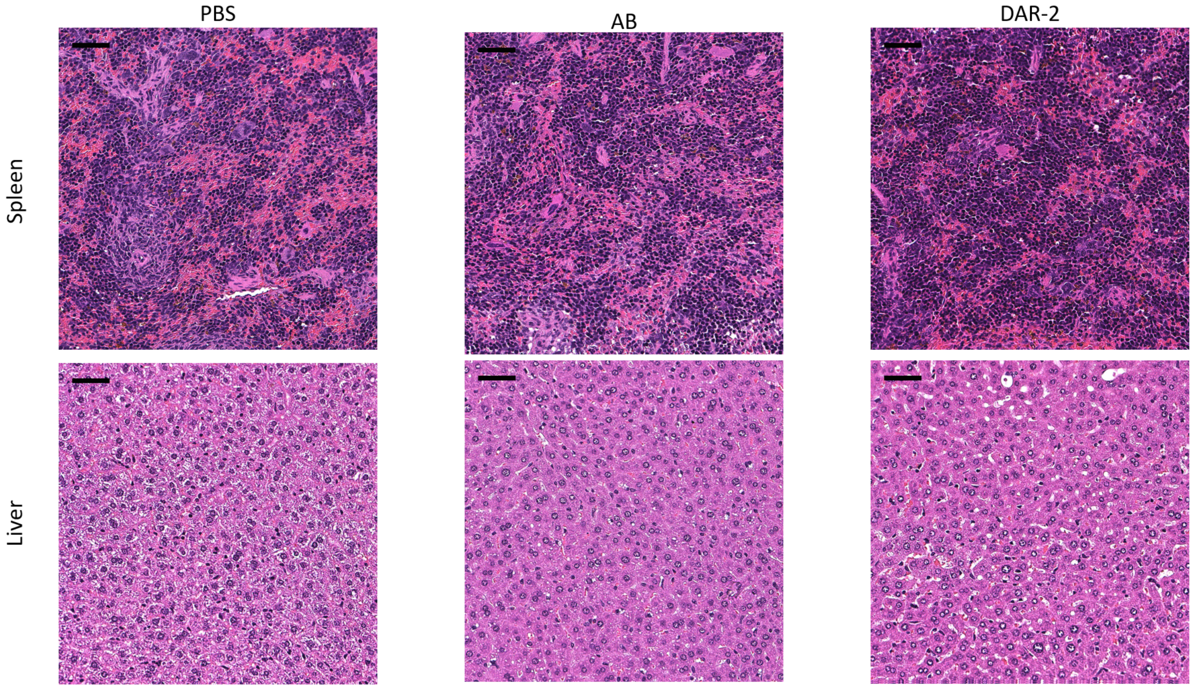
PBS
AB
DAR-2
Spleen
Liver

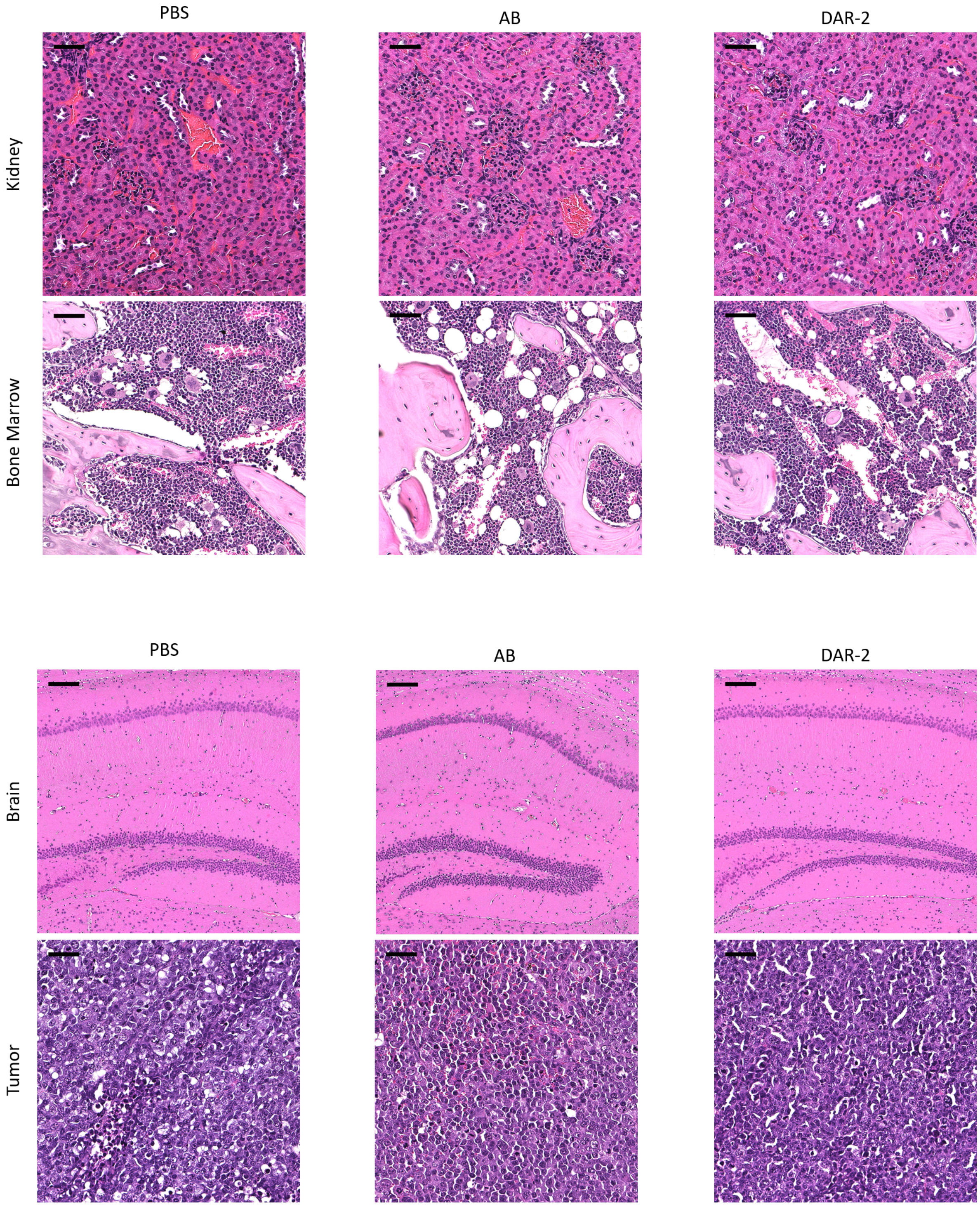

Figure 101. Histology images. Representative hematoxylin and eosin pictures for heart, lung, spleen, liver, kidney, bone marrow, brain and primary tumor.

HT Code	cassete ID	Animal ID	Experimental ID	Tissue/organ	Observations	Diagnosis	Other lesions (non-relevant)	Tumor	Score
HT409/20	1	PBS-1	PBS	Heart, Lung, thymus	No changes			**0**	**0**
HT409/20	2	PBS-1	PBS	Liver	No changes			**0**	**0**
HT409/20	3	PBS-1	PBS	Spleen and pancreas	Spleen: Brownish pigment accumulation, intracytoplasmatic, reticular cells, diffuse, minimal	Spleen: Hemossiderosis	Background lesion, non significant.	**0**	**1**
HT409/20	4	PBS-1	PBS	Kidneys and adrenal	No changes			**0**	**0**
HT409/20	5	PBS-1	PBS	Reproductive	No changes			**0**	**0**
HT409/20	6	PBS-1	PBS	Femur	No changes			**0**	**0**
HT409/20	7	PBS-1	PBS	Brain	No changes			**0**	**0**
HT409/20	8	PBS-1	PBS	Spinal Cord				**0**	**0**
HT409/20	9	PBS-1	PBS	Tumour	Tumour: Expanding the dermis and elevating the epidermis there is a well-demarcated, non encapsulated, mildly infiltrative, densely cellular mass. The mass is composed by poorly-differentiated neoplasic cells arranged in sheets and irregular nest, supported by scant amount of fibrovascular stroma. There are multifocal to coalescent areas of necrosis (20 to 25% of the mass). The neoplasic cells are polygonal to oval, with poorly-distinct cell borders, scant amount of amphophilic cytoplasm, often vacuolized, and oval to elongated nuclei, with vesicular chromatin and 3 to 4 nucleolus. Anisocytosis and anisokaryosis are high and the mitotic index is high (3 to 6 mitosis per high power field).			**NA**	**NA**
HT409/20	10	PBS-2	PBS	Heart, Lung, thymus	Heart and lung: No changes; Thymus: occasional apoptotic cells, diffuse, mild			**0**	**0**

HT409/20	11	PBS-2	PBS	Liver	Liver: micro-vesicular vacuoles, intracytplasmatic, hepatocyte, multifocal, mild	Liver: Lipidosis, mild	Background lesion, non significant.	**0**	**2**
HT409/20	12	PBS-2	PBS	Spleen and pancreas	Spleen: Brownish pigment accumulation, intracytoplasmatic, reticular cells, diffuse, minimal	Spleen: Hemossiderosis, minimal to mild	background lesion, non significant.	**0**	**1**
HT409/20	13	PBS-2	PBS	Kidneys and adrenal	No changes			**0**	**0**
HT409/20	14	PBS-2	PBS	Reproductive	Uterus: Endometric cystic hyperplasia, diffuse	Uterus: Endometric cystic hyperplasia	Background lesion, non significant.	**0**	**0**
HT409/20	15	PBS-2	PBS	Femur	No changes			**0**	**0**
HT409/20	16	PBS-2	PBS	Brain	No changes			**0**	**0**
HT409/20	17	PBS-2	PBS	Spinal Cord	No changes			**0**	**0**
HT409/20	18	PBS-2	PBS	Tumor	Tumour: Expanding the dermis and elevating the epidermis there is a well-demarcated, non encapsulated, mildly infiltrative, densely cellular mass. The mass is composed by poorly-differentiated neoplasic cells arranged in sheets and irregular nest, supported by scant amount of fibrovascular stroma. There are multifocal to coalescent areas of necrosis (30 to 35% of the mass). The neoplasic cells are polygonal to oval, with poorly-distinct cell borders, scant amount of amphophilic cytoplasm, often vacuolized, and oval to elongated nuclei, with vesicular chromatin and 3 to 4 nucleolus. Anisocytosis and anisokaryosis are high and the mitotic index is high (3 to 6 mitosis per high power field).			**NA**	**NA**
HT409/20	19	AB-1	AB	Heart, Lung, thymus	Heart and lung: No changes; Thymus: occasional apoptotic cells, diffuse, mild			**0**	**0**
HT409/20	20	AB-1	AB	Liver	Liver: micro-vesicular vacuoles, intracytoplasmatic, hepatocyte, multifocal, mild	Liver: Lipidosis, mild	Background lesion, non significant.	**0**	**2**

HT409/20	21	AB-1	AB	Spleen and pancreas	Spleen: Brownish pigment accumulation, intracytoplasmatic, reticular cells, diffuse, minimal	Spleen: Hemossiderosis, minimal to mild	Background lesion, non significant.	**0**	**1**
HT409/20	22	AB-1	AB	Kidneys and adrenal	No changes			**0**	**0**
HT409/20	23	AB-1	AB	Reproductive	No changes			**0**	**0**
HT409/20	24	AB-1	AB	Femur	No changes			**0**	**0**
HT409/20	25	AB-1	AB	Brain	No changes			**0**	**0**
HT409/20	26	AB-1	AB	Spinal Cord	No changes			**0**	**0**
HT409/20	27	AB-1	AB	Tumour	Tumour: Expanding the dermis and elevating the epidermis there is a well-demarcated, non encapsulated, mildly infiltrative, densely cellular mass. The mass is composed by poorly-differentiated neoplasic cells arranged in sheets and irregular nest, supported by scant amount of fibrovascular stroma. There are multifocal to coalescent areas of necrosis (50% of the mass). The neoplasic cells are polygonal to oval, with poorly-distinct cell borders, scant amount of amphophilic cytoplasm, often vacuolized, and oval to elongated nuclei, with vesicular chromatin and 3 to 4 nucleolus. Anisocytosis and anisokaryosis are high and the mitotic index is high (3 to 6 mitosis per high power field).			**NA**	**NA**
HT409/20	28	AB-2	AB	Heart, Lung, thymus	No changes			**0**	**0**
HT409/20	29	AB-2	AB	Liver	Liver: micro-vesicular vacuoles, intracytoplasmatic, hepatocyte, multifocal, mild	Liver: Lipidosis, mild	Background lesion, non significant.	**0**	**2**
HT409/20	30	AB-2	AB	Spleen and pancreas	Spleen: Brownish pigment accumulation, intracytoplasmatic, reticular cells, diffuse, mild	Spleen: Hemossiderosis, mild	Background lesion, non significant.	**0**	**2**
HT409/20	31	AB-2	AB	Kidneys and adrenal	No changes			**0**	**0**

HT409/20	32	AB-2	AB	Reproductive	No changes			**0**	**0**
HT409/20	33	AB-2	AB	Femur	No changes			**0**	**0**
HT409/20	34	AB-2	AB	Brain	No changes			**0**	**0**
HT409/20	35	AB-2	AB	Spinal Cord	No changes			**0**	**0**
HT409/20	36	AB-2	AB	Tumour	Tumour: Expanding the dermis and elevating the epidermis there is a well-demarcated, non encapsulated, mildly infiltrative, densely cellular mass. The mass is composed by poorly-differentiated neoplasic cells arranged in sheets and irregular nest, supported by scant amount of fibrovascular stroma. There are multifocal to coalescent areas of necrosis (30% of the mass). The neoplasic cells are polygonal to oval, with poorly-distinct cell borders, scant amount of amphophilic cytoplasm, often vacuolized, and oval to elongated nuclei, with vesicular chromatin and 3 to 4 nucleolus. Anisocytosis and anisokaryosis are high and the mitotic index is high (3 to 6 mitosis per high power field).			**NA**	**NA**
HT409/20	37	DAR2-1	DAR2	Heart, Lung, thymus	Heart: vacuolation, intracytoplasmatic, cardiomyocyte, focally extensive, minimal; Lung and thymus: No changes	Heart: Vacuolar degeneration, minimal	Background lesion, non significant.	**0**	**1**
HT409/20	38	DAR2-1	DAR2	Liver	No changes			**0**	**0**
HT409/20	39	DAR2-1	DAR2	Spleen and pancreas	Spleen: Brownish pigment accumulation, intracytoplasmatic, reticular cells, diffuse, minimal	Spleen: Hemossiderosis	Background lesion, non significant.	**0**	**1**
HT409/20	40	DAR2-1	DAR2	Kidneys and adrenal	No changes			**0**	**0**
HT409/20	41	DAR2-1	DAR2	Reproductive	No changes			**0**	**0**
HT409/20	42	DAR2-1	DAR2	Femur	No changes			**0**	**0**
HT409/20	43	DAR2-1	DAR2	Brain	No changes			**0**	**0**

HT409/20	44	DAR2-1	DAR2	Spinal Cord	No changes			**0**	**0**
HT409/20	45	DAR2-1	DAR2	Tumour	Tumour: Expanding the dermis and elevating the epidermis there is a well-demarcated, non encapsulated, mildly infiltrative, densely cellular mass. The mass is composed by poorly-differentiated neoplasic cells arranged in sheets and irregular nest, supported by scant amount of fibrovascular stroma. There are multifocal to coalescent areas of necrosis (30 to 35% of the mass). The neoplasic cells are polygonal to oval, with poorly-distinct cell borders, scant amount of amphophilic cytoplasm, often vacuolized, and oval to elongated nuclei, with vesicular chromatin and 3 to 4 nucleolus. Anisocytosis and anisokaryosis are high and the mitotic index is high (3 to 6 mitosis per high power field).			**NA**	**NA**
HT409/20	46	DAR2-2	DAR2	Heart, Lung, thymus	No changes			**0**	**0**
HT409/20	47	DAR2-2	DAR2	Liver	No changes			**0**	**0**
HT409/20	48	DAR2-2	DAR2	Spleen and pancreas	Spleen: Brownish pigment accumulation, intracytoplasmatic, reticular cells, diffuse, minimal	Spleen: Hemossiderosis	Background lesion, non significant.	**0**	**1**
HT409/20	49	DAR2-2	DAR2	Kidneys and adrenal	No changes			**0**	**0**
HT409/20	50	DAR2-2	DAR2	Reproductive	No changes			**0**	**0**
HT409/20	51	DAR2-2	DAR2	Femur	No changes			**0**	**0**
HT409/20	52	DAR2-2	DAR2	Brain	No changes			**0**	**0**
HT409/20	53	DAR2-2	DAR2	Spinal Cord	No changes			**0**	**0**

HT409/20	54	DAR2-2	DAR2	Tumour	Tumour: Expanding the dermis and elevating the epidermis there is a well-demarcated, non encapsulated, mildly infiltrative, densely cellular mass. The mass is composed by poorly-differentiated neoplasic cells arranged in sheets and irregular nest, supported by scant amount of fibrovascular stroma. There are multifocal to coalescent areas of necrosis (50% of the mass). The neoplasic cells are polygonal to oval, with poorly-distinct cell borders, scant amount of amphophilic cytoplasm, often vacuolized, and oval to elongated nuclei, with vesicular chromatin and 3 to 4 nucleolus. Anisocytosis and anisokaryosis are high and the mitotic index is high (3 to 6 mitosis per high power field).			**NA**	**NA**

Table 1. Histology data.

Quantitative LC-MS/MS analysis of β-lapachone in formol fixed xenograft mouse tumour and formol supernatant.

Four mouse xenograft tumour samples were provided in formol. Test compound **1** was provided with the samples and assumed to be 100% pure. Analysis was carried out as described in materials and methods. Analysis was conducted in a batch, consisting of calibration standards, quality control samples, blank control matrix samples, internal standard only samples and study samples. Calibration curves were constructed using Linear regression with a $1/x^2$ weighting. Precision and accuracy of plasma sample analysis were within the acceptance criteria of ±20%.

Sample ID	Concentration, ng/g
PBS Homogenate	*BLQ*
ADC Homogenate	0.327

Calibration range: 0.25-250 ng/g*

*Based on a homogenate concentration of 200 mg/mL

BLQ: Below the limit of quantitation

Table 2. Tissue homogenate samples.

Sample ID	Concentration, ng/mL
PBS	*BLQ*
ADC	*BLQ**

Calibration range: 0.05-50 ng/mL

BLQ: Below the limit of quantitation

*Small signal detected, but not quantifiable.

Table 3. Formol supernatant samples.

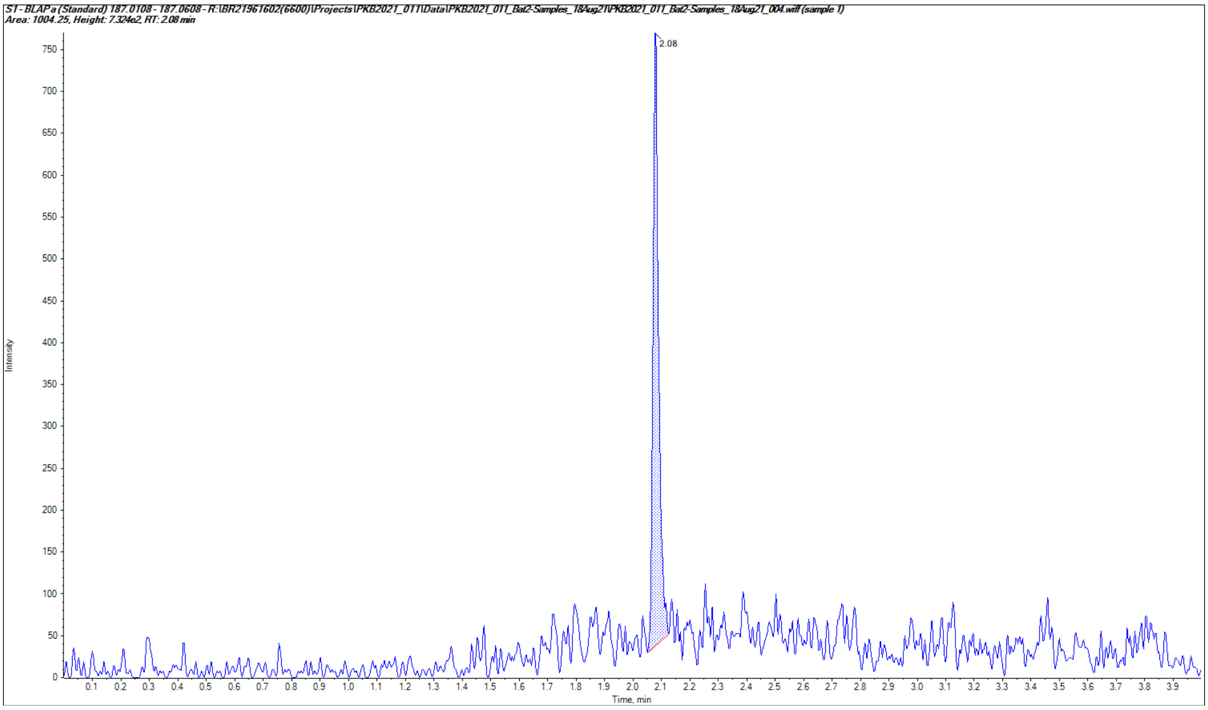

Figure 102. Lower Limit of Quantitation (LLOQ), 0.05 ng/mL or 0.25 ng/g (based on 200mg/mL homogenate).

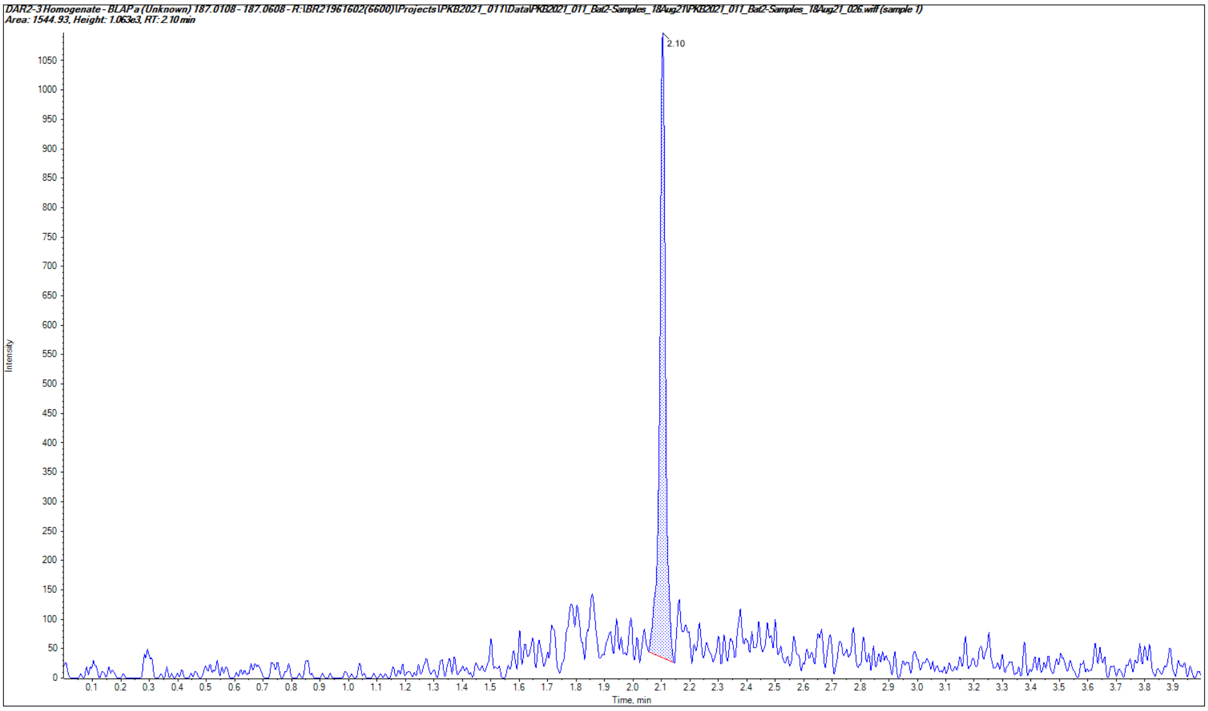

Figure 103. ADC 200 mg/mL Homogenate Sample, 0.327 ng/g.

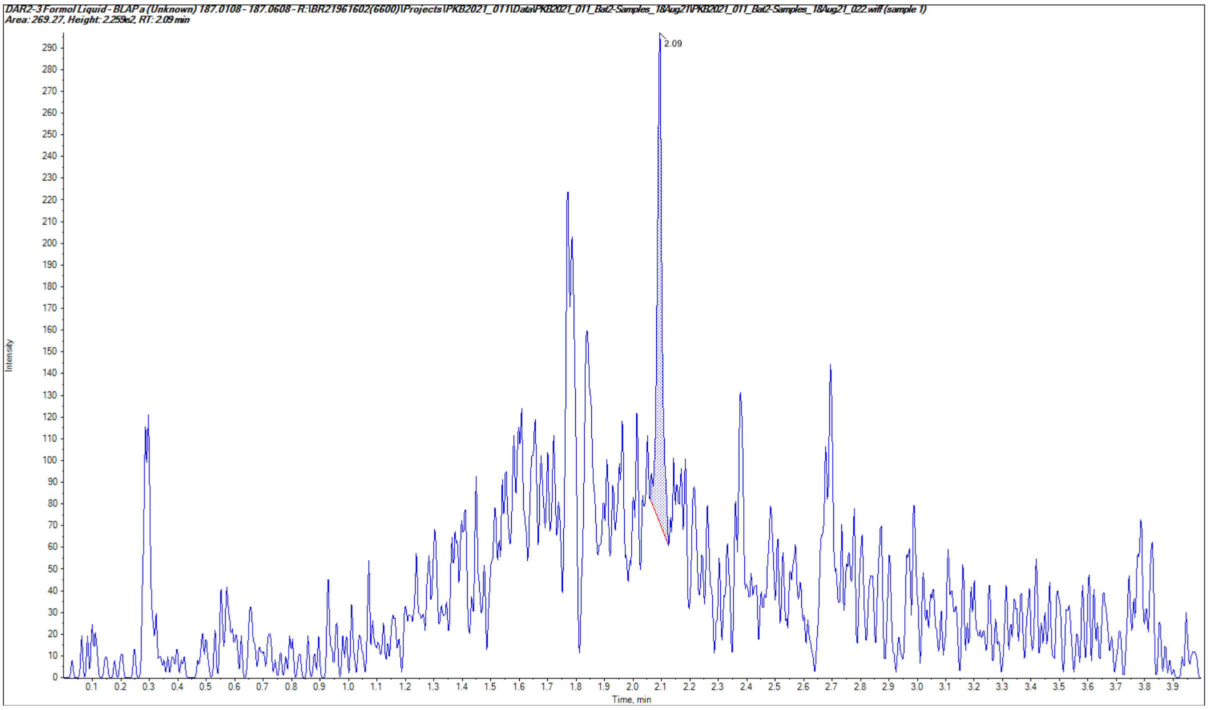

Figure 104. ADC Formol Supernatant Sample, BLQ.

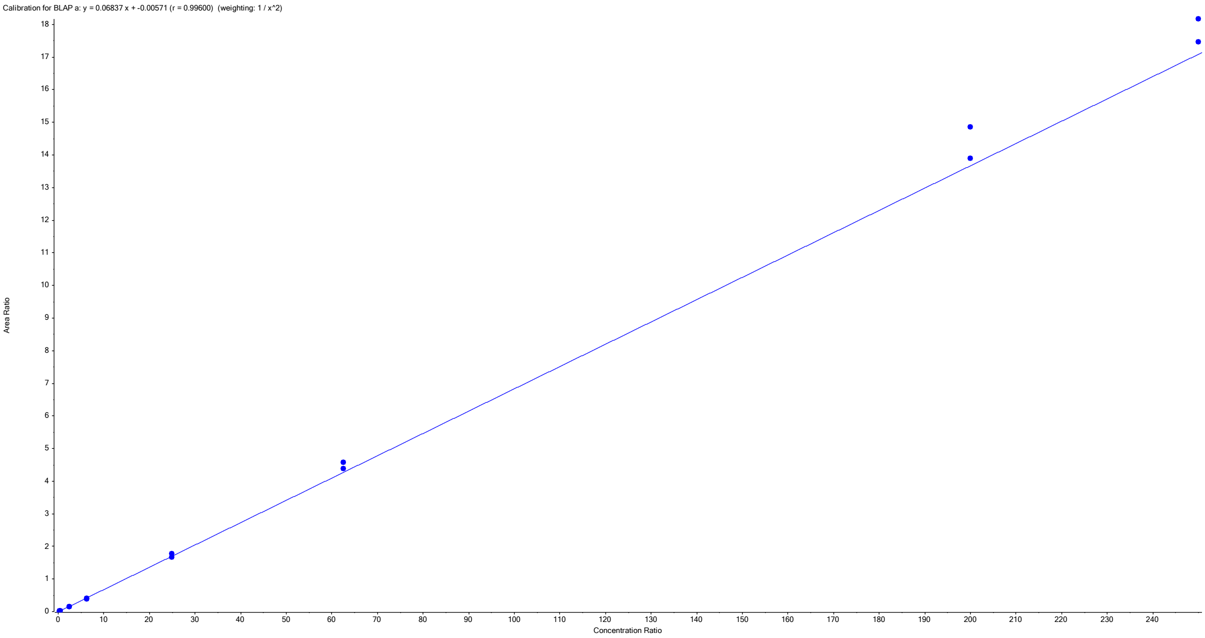

Figure 105. Linear calibration curve of β-lapachone.

8. *In-silico* modelling

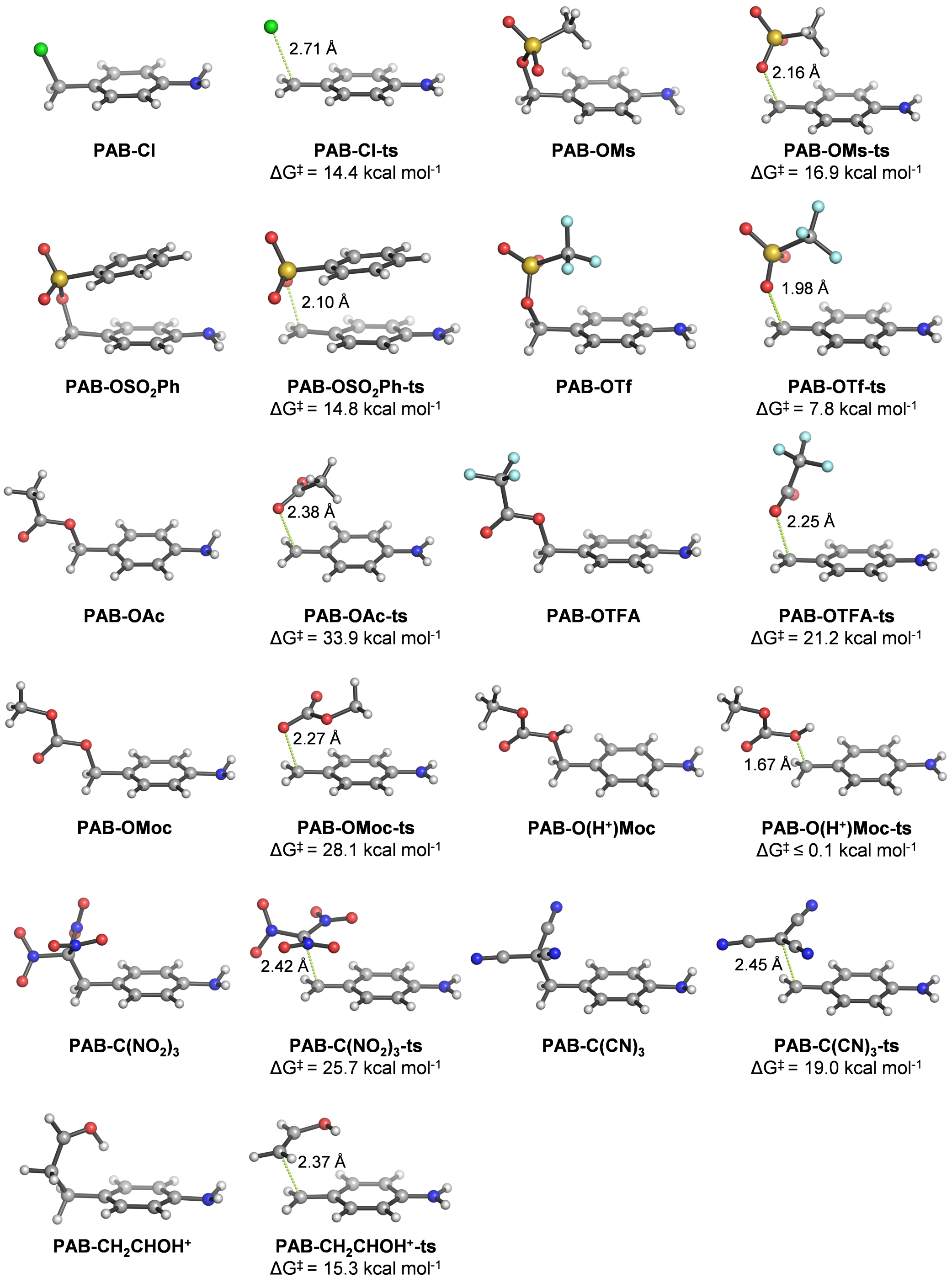

Figure 106. Lowest energy structures (reactants and transition states) for the fragmentation of neutral p-aminobenzyl (PAB) derivatives with leaving groups of different nature (halogens, activated alcohols, carbon-based) calculated with PCM(H_2O)/M06-2X/6-31+G(d,p). The neutral species of chlorides and activated alcohols are able to fragment with low to moderate activation barriers (7.8 – 16.9 kcal mol^{-1}). The neutral carbonate leaving group showed a much higher activation barrier (28.1 kcal mol^{-1}), which drastically drops upon protonation (≤ 0.1 kcal mol^{-1}). Neutral carbon-based leaving groups needed three electron-withdrawing

groups (CN or NO_2) or protonated carbonyl groups to fragment with moderate to high activation barriers (15.3 – 25.7 kcal mol^{-1}).

Derivation of Equations 1 and 2

The rate law for a typical first-order reaction (A → P) is:

$$rate = -\frac{d[A]}{dt} = \frac{d[P]}{dt} = k[A]$$

The integrated expression of this equation is:

$$[A]_t = [A]_0 \cdot e^{-kt} \qquad [P]_t = [A]_0 - [A]_0 \cdot e^{-kt}$$

where $[A]_0$ is the initial concentration of reagent A, and $[A]_t$ and $[P]_t$ are the concentrations of reagent A and products P at a certain time (t).

Consider that reagent A is partially protonated, and that it is the protonated species (AH^+) that is the only one that undergoes fragmentation to products (P):

$$A + H^+ \overset{K_a}{\rightleftharpoons} AH^+$$

$$AH^+ \xrightarrow{k_1} P$$

The rate of product formation is,

$$rate = k_1[AH^+] = k_1\frac{[A][H^+]}{K_a}$$

where:

$$K_a = k_1\frac{[A][H^+]}{[AH^+]}$$

In turn, the experimentally determined rate of product formation can be expressed in terms of the total concentration of A ($[A_T] = [A] + [AH^+]$):

$$rate = -\frac{d[A_T]}{dt} = \frac{d[P]}{dt} = k_{obs}[A_T]$$

Combining both equations, we can express the measured pseudo-first-order constant (k_{obs}) as a function of pH ($[H^+]$):

$$rate = k_{obs}[A_T] = k_1 \frac{[A][H^+]}{K_a}$$

$$k_{obs} = k_1 \frac{[A][H^+]}{K_a[A_T]}$$

$$k_{obs} = k_1 \frac{[A][H^+]}{K_a([A] + [AH^+])}$$

$$k_{obs} = k_1 \frac{[A][H^+]}{K_a\left([A] + \frac{[A][H^+]}{K_a}\right)}$$

$$k_{obs} = \frac{k_1[H^+]}{K_a + [H^+]}$$

An analogous derivation can be carried out for a partially deprotonated reagent (AH, A^-), which is the only one that fragments to products:

$$AH \underset{}{\overset{K_a}{\rightleftharpoons}} A^- + H^+$$

$$A^- \xrightarrow{k_2} P$$

$$k_{obs} = \frac{k_2 K_a}{K_a + [H^+]}$$

Suppose a single reagent A that reacts independently following a first-order reaction to afford different products:

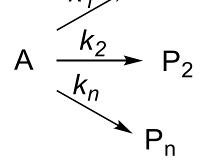

The rate of disappearance of A is a summation of the individual rates,

$$rate = -\frac{d[A]}{dt} = (k_1 + k_2 + \cdots + k_n)[A]$$

therefore $k_{obs} = k_1 + k_2 + \ldots + k_n$.

Similarly, consider that reagent AH is partially protonated and deprotonated, and that only those charged forms (AH^{2+}, A^-) react independently following first-order kinetics:

$$AH + H^+ \xrightleftharpoons{K_{a+}} AH_2^+ \xrightarrow{k_+} P$$

$$AH \xrightleftharpoons{K_{a-}} H^+ + A^- \xrightarrow{k_-} P$$

The rate of disappearance of A is a summation of the fragmentation rates from each reactive species:

$$rate = k_+[AH_2^+] + k_-[A^-] = k_+\frac{[AH][H^+]}{K_{a+}} + k_-\frac{[AH]K_{a-}}{[H^+]}$$

The experimentally determined rate of product formation is expressed in terms of the total concentration of AH ($[AH_T] = [AH] + [AH^+]+[A^-]$), and the measured pseudo-first-order constant (k_{obs}) can be expressed as a function of pH ($[H^+]$)

$$rate = k_{obs}[AH_T] = k_+\frac{[AH][H^+]}{K_{a+}} + k_-\frac{[AH]K_{a-}}{[H^+]}$$

$$k_{obs} = \frac{k_+\frac{[AH][H^+]}{K_{a+}} + k_-\frac{[AH]K_{a-}}{[H^+]}}{[A] + [AH^+] + [A^-]}$$

$$k_{obs} = \frac{k_+\frac{[A][H^+]}{K_{a+}} + k_-\frac{[A]K_{a-}}{[H^+]}}{[A] + \frac{[A][H^+]}{K_{a+}} + \frac{[A]K_{a-}}{[H^+]}}$$

$$k_{obs} = \frac{k_+\frac{[H^+]}{K_{a+}} + k_-\frac{K_{a-}}{[H^+]}}{1 + \frac{[H^+]}{K_{a+}} + \frac{K_{a-}}{[H^+]}}$$

In a generic form, it can be expressed as,

$$k_{obs} = \frac{\sum k_r [A_r]}{[A_T]}$$

where kr and [A_r] are the intrinsic rate constants and concentration of each reactive species, and [A_T] is the total concentration of A, expressed as the summation of the concentration of each species, either reactive or not.

Applying this generic equation to the considered species in equilibrium for protected (**20**, R = Ac) or unprotected phenanthrenequinone derivative (**7**, R = H), an expression for the theoretical reaction rate constant (k_{theo}) can be derived, which in turn can be related to the experimentally observed rate constant (k_{obs}):

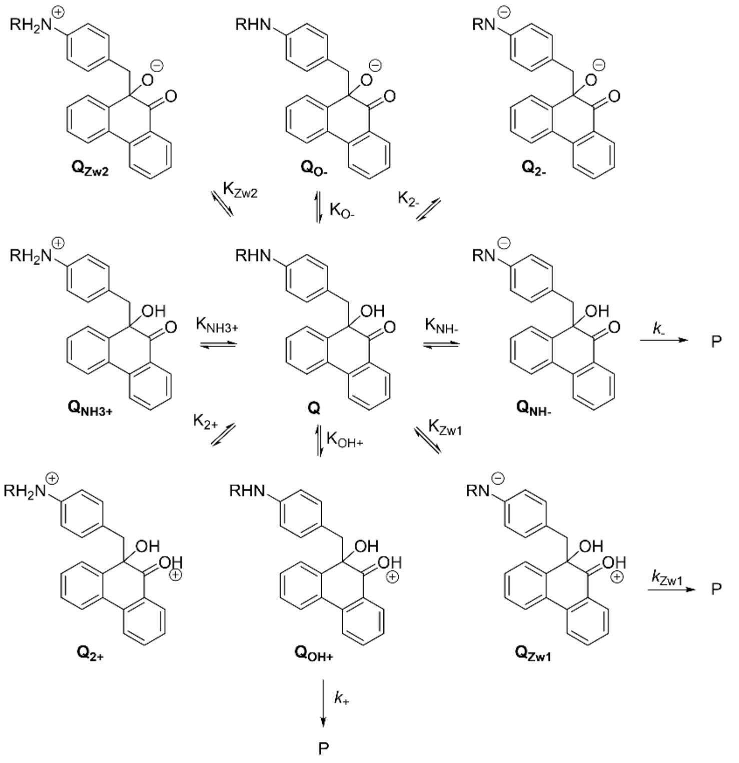

$$K_{NH3+} = \frac{[Q][H^+]}{[Q_{NH3+}]}$$

$$K_{OH+} = \frac{[Q][H^+]}{[Q_{OH+}]}$$

$$K_{NH-} = \frac{[Q_{NH-}][H^+]}{[Q]}$$

$$K_{O-} = \frac{[Q_{O-}][H^+]}{[Q]}$$

$$K_{Zw1} = \frac{[Q_{Zw1}]}{[Q]}$$

$$K_{Zw2} = \frac{[Q_{Zw2}]}{[Q]}$$

$$K_{2-} = \frac{[Q_{2-}][H^+]^2}{[Q]}$$

$$K_{2+} = \frac{[Q][H^+]^2}{[Q_{2+}]}$$

$$k_{obs} = \frac{\sum k_r [Q_r]}{[Q_T]} = \frac{\sum k_r [Q_r]}{\sum [Q_i]} \approx k_{theo} =$$

$$= \frac{k_+[Q_{OH+}] + k_-[Q_{NH-}] + k_{zw}[Q_{Zw1}]}{[Q] + [Q_{NH3+}] + [Q_{OH+}] + [Q_{NH-}] + [Q_{O-}] + [Q_{Zw1}] + [Q_{Zw2}] + [Q_{2-}] + [Q_{2+}]}$$

$k_{obs} \approx k_{theo}$

$$= \frac{k_{+}\frac{[H^{+}]}{K_{OH^{+}}}[Q] + k_{-}\frac{K_{NH^{-}}}{[H^{+}]}[Q] + k_{zw}K_{Zw1}[Q]}{[Q] + \frac{[H^{+}]}{K_{NH_3^{+}}}[Q] + \frac{[H^{+}]}{K_{OH^{+}}}[Q] + \frac{K_{NH^{-}}}{[H^{+}]}[Q] + \frac{K_{O^{-}}}{[H^{+}]}[Q] + K_{Zw1}[Q] + K_{Zw2}[Q] + \frac{K_{2-}}{[H^{+}]^2}[Q] + \frac{[H^{+}]^2}{K_{2+}}[Q]}$$

$$k_{obs} \approx k_{theo} = \frac{k_{+}\frac{[H^{+}]}{K_{OH^{+}}} + k_{-}\frac{K_{NH^{-}}}{[H^{+}]} + k_{zw}K_{Zw1}}{1 + \frac{[H^{+}]}{K_{NH_3^{+}}} + \frac{[H^{+}]}{K_{OH^{+}}} + \frac{K_{NH^{-}}}{[H^{+}]} + \frac{K_{O^{-}}}{[H^{+}]} + K_{Zw1} + K_{Zw2} + \frac{K_{2-}}{[H^{+}]^2} + \frac{[H^{+}]^2}{K_{2+}}}$$

Equation 1

A similar equation can be derived for β-lapachone and dunnione derivatives **10** and **18**, for which an additional equilibrium with a non-reactive form can be considered.

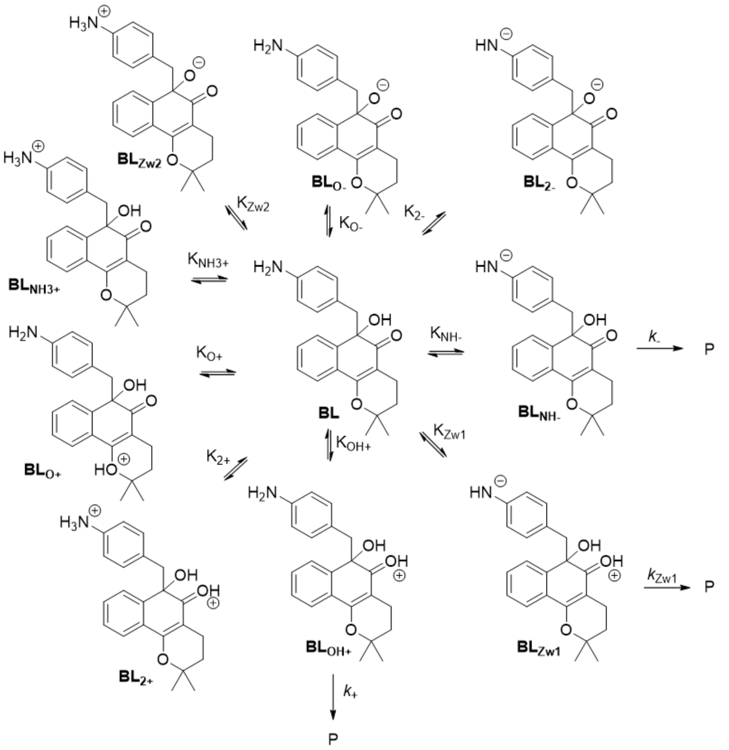

$$K_{NH3+} = \frac{[BL][H^{+}]}{[BL_{NH3+}]}$$

$$K_{OH+} = \frac{[BL][H^{+}]}{[BL_{OH+}]}$$

$$K_{NH-} = \frac{[BL_{NH-}][H^{+}]}{[BL]}$$

$$K_{O-} = \frac{[BL_{O-}][H^{+}]}{[BL]}$$

$$K_{Zw1} = \frac{[BL_{Zw1}]}{[BL]}$$

$$K_{Zw2} = \frac{[BL_{Zw2}]}{[BL]}$$

$$K_{2-} = \frac{[BL_{2-}][H^{+}]^2}{[BL]}$$

$$K_{2+} = \frac{[BL][H^{+}]^2}{[Q_{2+}]}$$

$$K_{O+} = \frac{[BL][H^{+}]^2}{[Q_{O+}]}$$

$$k_{obs} \approx k_{theo} = \frac{k_{+}\frac{[H^{+}]}{K_{OH^{+}}} + k_{-}\frac{K_{NH^{-}}}{[H^{+}]} + k_{zw}K_{Zw1}}{1 + \frac{[H^{+}]}{K_{NH_3^{+}}} + \frac{[H^{+}]}{K_{OH^{+}}} + \frac{K_{NH^{-}}}{[H^{+}]} + \frac{K_{O^{-}}}{[H^{+}]} + K_{Zw1} + K_{Zw2} + \frac{K_{2-}}{[H^{+}]^2} + \frac{[H^{+}]^2}{K_{2+}} + \frac{[H^{+}]}{K_{O^{+}}}}$$

Equation 2

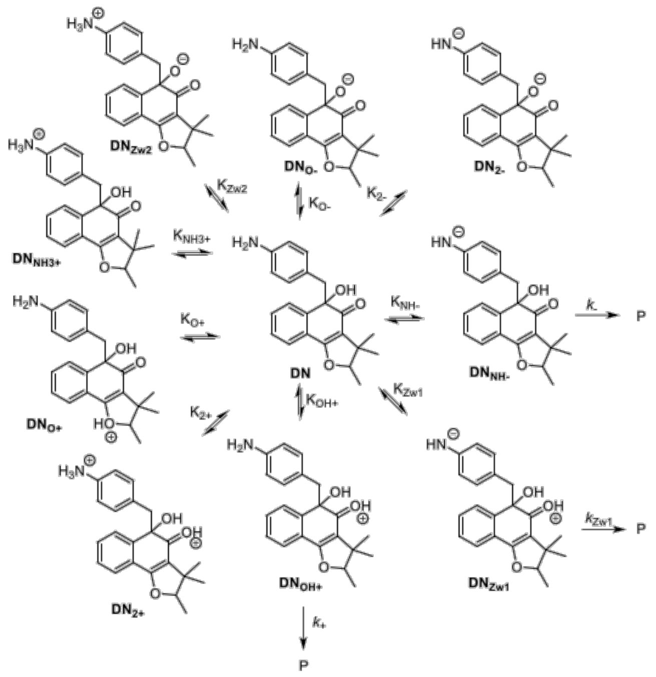

$$K_{NH3+} = \frac{[DN][H^+]}{[DN_{NH3+}]}$$

$$K_{OH+} = \frac{[DN][H^+]}{[DN_{OH+}]}$$

$$K_{NH-} = \frac{[DN_{NH-}][H^+]}{[DN]}$$

$$K_{O-} = \frac{[DN_{O-}][H^+]}{[DN]}$$

$$K_{Zw1} = \frac{[DN_{Zw1}]}{[DN]}$$

$$K_{Zw2} = \frac{[DN_{Zw2}]}{[DN]}$$

$$K_{2-} = \frac{[DN_{2-}][H^+]^2}{[DN]}$$

$$K_{2+} = \frac{[DN][H^+]^2}{[Q_{2+}]}$$

$$K_{O+} = \frac{[DN][H^+]^2}{[Q_{O+}]}$$

$$k_{obs} \approx k_{theo} = \frac{k_+ \frac{[H^+]}{K_{OH^+}} + k_- \frac{K_{NH^-}}{[H^+]} + k_{Zw} K_{Zw1}}{1 + \frac{[H^+]}{K_{NH_3^+}} + \frac{[H^+]}{K_{OH^+}} + \frac{K_{NH^-}}{[H^+]} + \frac{K_{O^-}}{[H^+]} + K_{Zw1} + K_{Zw2} + \frac{K_{2-}}{[H^+]^2} + \frac{[H^+]^2}{K_{2+}} + \frac{[H^+]}{K_{O^+}}}$$

Equation 2

Figure 107. Derivation of Equations 1 and 2.

	PCM(H_2O)/<br>M06-2X/<br>6-31+G(d,p)	SMD(H_2O)/<br>M06-2X/<br>6-31+G(d,p)	PCM(H_2O)/<br>ωB97x-D/<br>6-31+G(d,p)	PCM(H_2O)/<br>M06-2X/<br>6-311++G(2d,p)
**7_{NH-}-ts**	11.8	11.1	14.4	11.1
**7_{OH+}-ts**	6.5	6.8	9.3	6.1
**7_{Zw1}-ts**	≤ 0.1	≤ 0.1	0.6	≤ 0.1

Table 4. Activation energies ($\Delta G^{\ddagger}$ in kcal mol^{-1}) for the fragmentation of **7_{OH+}**, **7_{NH-}**, and **7_{Zw1}** calculated at four different theory levels combining different solvent models, density functionals and basis sets. Geometries and frequencies were calculated at a PCM(H_2O)/M06-2X/6-31+G(d,p) level, and the thermal and entropy corrections at 298 K were applied to single point energies calculated at each level.

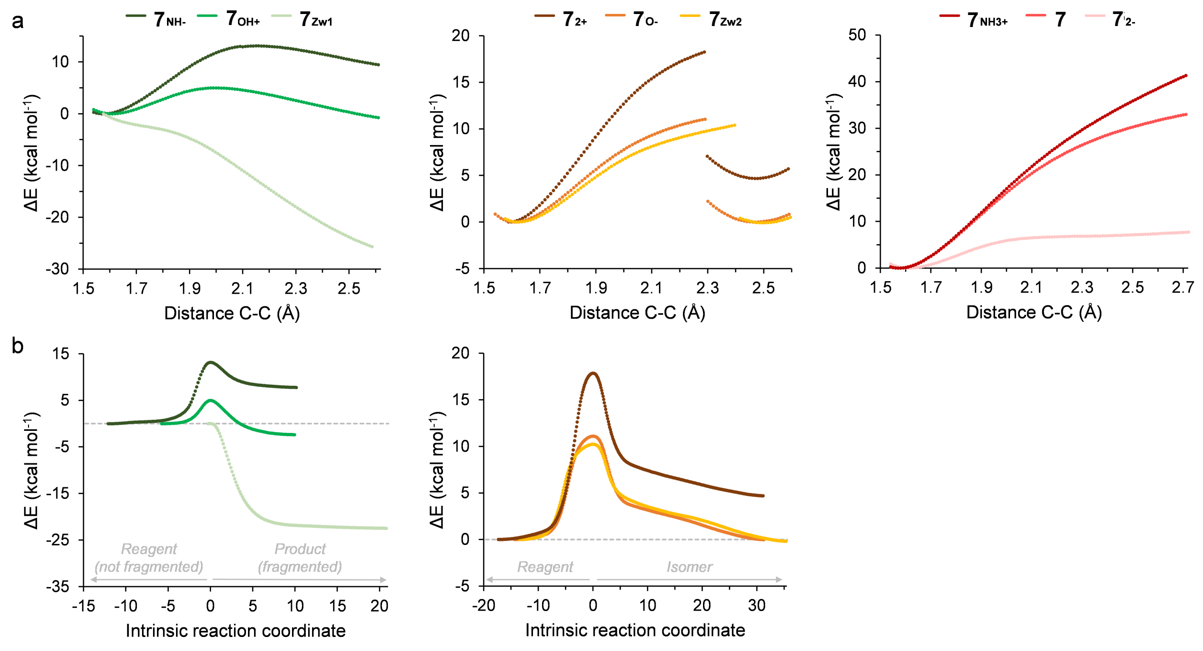

Figure 108. a, Potential energy surfaces (PES) calculated with PCM(H_2O)/M06-2X/6-31+G(d,p) for the fragmentation of different protomers of reagent **7** along the breaking C–C bond. All the scans started from the lowest energy ground state conformer for each species. A transition state corresponding to the fragmentation reaction was located for species $\mathbf{7_{NH-}}$ (dark green), $\mathbf{7_{OH+}}$ (green), and $\mathbf{7_{Zw1}}$ (light green). PES for species $\mathbf{7_{2+}}$ (brown), $\mathbf{7_{O-}}$ (orange), and $\mathbf{7_{Zw2}}$ (yellow) suggested an isomerization of the p-aminobenzyl fragment to the adjacent carbonyl group; the maximum points of these PES lead to α-ketol rearrangement transition states after re-optimization. No TS for either fragmentation or rearrangement were found for species $\mathbf{7_{NH3+}}$ (red), **7** (pink), and $\mathbf{7_{2-}}$ (pale pink). **b,** Intrinsic Reaction Coordinate (IRC) plots calculated with PCM(H_2O)/M06-2X/6-31+G(d,p) from the lowest energy fragmentation (left) and rearrangement (right) transition states. Gray dashed lines indicate the reactants ground state (ΔE = 0.0 kcal mol^{-1}).

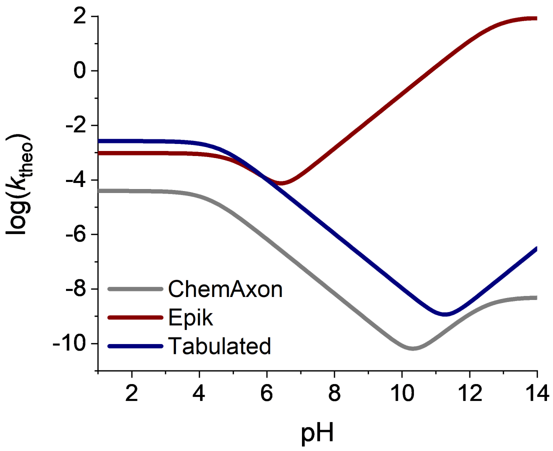

Equilibrium constants	ChemAxon	Epik	Tabulated
pK_{NH3+}	4.2	5.0	4.6
pK_{OH+}	-8.4	-6.2	-6.2
pK_{NH-}	25.3	15.3	25.0
pK_{O-}	12.5	12.8	17.0
pK_{Zw1}	33.7	21.5	31.2
pK_{Zw2}	8.3	7.8	12.4
pK_{2-}	37.8	28.2	42.0
pK_{2+}	-4.2	-1.2	-1.6

Figure 109. Comparison of the theoretical rate constants (shown as logarithmic) at different pH values for fragmentation of unprotected compound **7** using equation 1 and either tabulated (blue) or calculated (grey or red) equilibrium constants. Intrinsic reaction constants (k_+ = $1.68{\cdot}10^8$ s^{-1}; k_- = $2.93{\cdot}10^4$ s^{-1}; k_{zw} = $5.17{\cdot}10^{12}$ s^{-1}) were calculated from the activation barriers of the reactive species $\mathbf{7}_{\mathbf{NH-}}$, $\mathbf{7}_{\mathbf{OH+}}$, and $\mathbf{7}_{\mathbf{Zw1}}$.

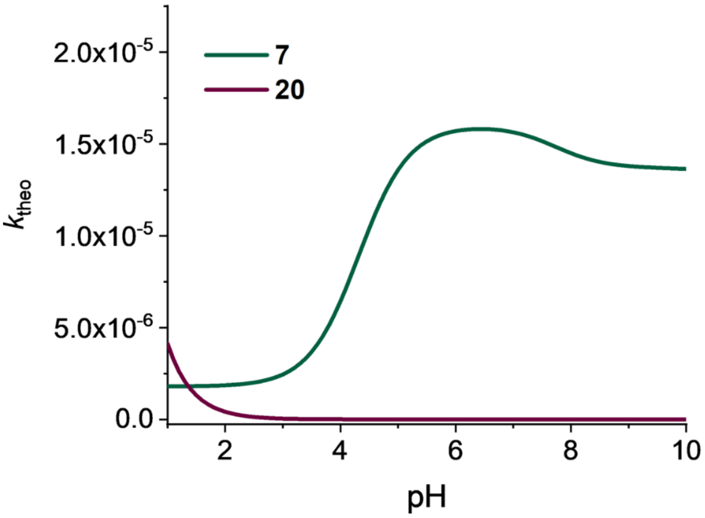

Fitting parameters	7	20
k_+ (s^{-1})	$1.68 \cdot 10^{8}$	$1.86 \cdot 10^{8}$
k_- (s^{-1})	$2.93 \cdot 10^{4}$	$2.16 \cdot 10^{-5}$
k_{Zw} (s^{-1})	$5.17 \cdot 10^{12}$	$4.36 \cdot 10^{9}$
K_{NH3+} (M)	$4.83 \cdot 10^{-5}$	*$3.55 \cdot 10^{4}$
K_{OH+} (M)	$4.51 \cdot 10^{9}$	$4.51 \cdot 10^{9}$
K_{NH-} (M)	$8.78 \cdot 10^{-18}$	*$4.36 \cdot 10^{-15}$
K_{O-} (M)	$1.88 \cdot 10^{-8}$	$1.88 \cdot 10^{-8}$
K_{Zw1}	$3.10 \cdot 10^{-18}$	$3.10 \cdot 10^{-18}$
K_{Zw2}	$8.18 \cdot 10^{-16}$	$8.18 \cdot 10^{-16}$
K_{2-} (M^2)	$8.78 \cdot 10^{-21}$	$8.78 \cdot 10^{-21}$
K_{2+} (M^2)	$1.35 \cdot 10^{15}$	$1.35 \cdot 10^{15}$

Figure 110. Comparison of theoretical rate constants (k_{theo}) at different pH values for fragmentation of unprotected compound **7** (green) and N-Ac-protected derivative **20** (red). Curve for compound **7** was obtained from the fitting of experimental rate constants to equation 1. Curve for compound **20** was generated using the intrinsic reaction constants calculated from the activation barriers of the reactive species $\mathbf{20}_{\mathbf{NH-}}$, $\mathbf{20}_{\mathbf{OH+}}$, and $\mathbf{20}_{\mathbf{Zw1}}$ and the experimentally derived equilibrium constants for compound **7**. Additionally, asterisks denote equilibrium constants involving the *para*-acetanilide group non directly attainable from experimental data and thus calculated using ChemAxon for the protonation of the acetamide carbonyl group (K_{NH3+}) and deprotonation of the acetamide NH group (K_{NH-}). From these data, elimination from *N*-protected **20** across the whole pH range is predicted to be exceedingly slow. In fact, at pH > 2, the elimination rate for protected derivative **20** is slowed down by 2–5 orders of magnitude.

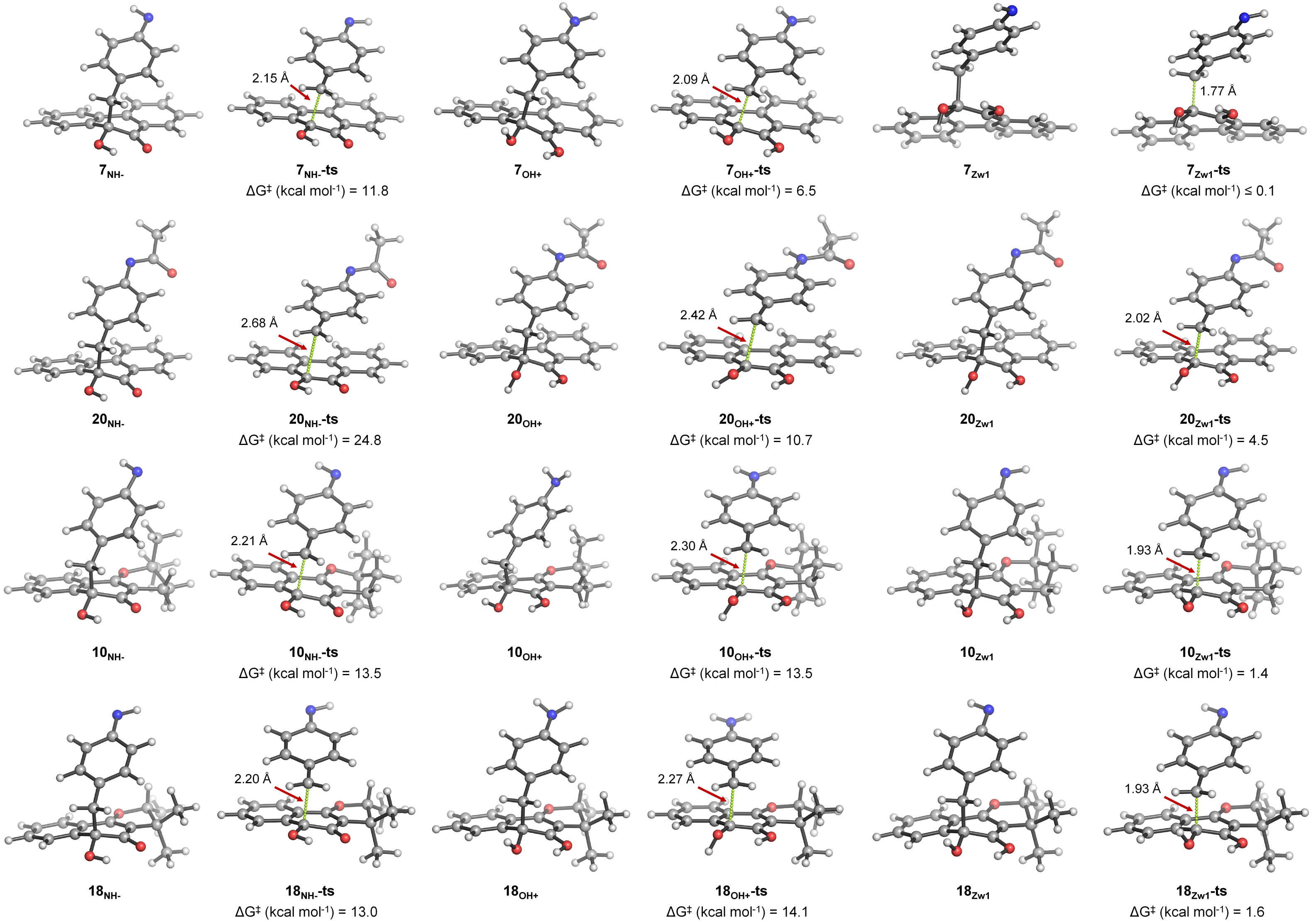

Figure 111. Lowest energy structures (reactants and transition states) for the elimination of reactive protomers of reagents **7**, **20**, **10** and **18** calculated with PCM(H_2O)/M06-2X/6-31+G(d,p).

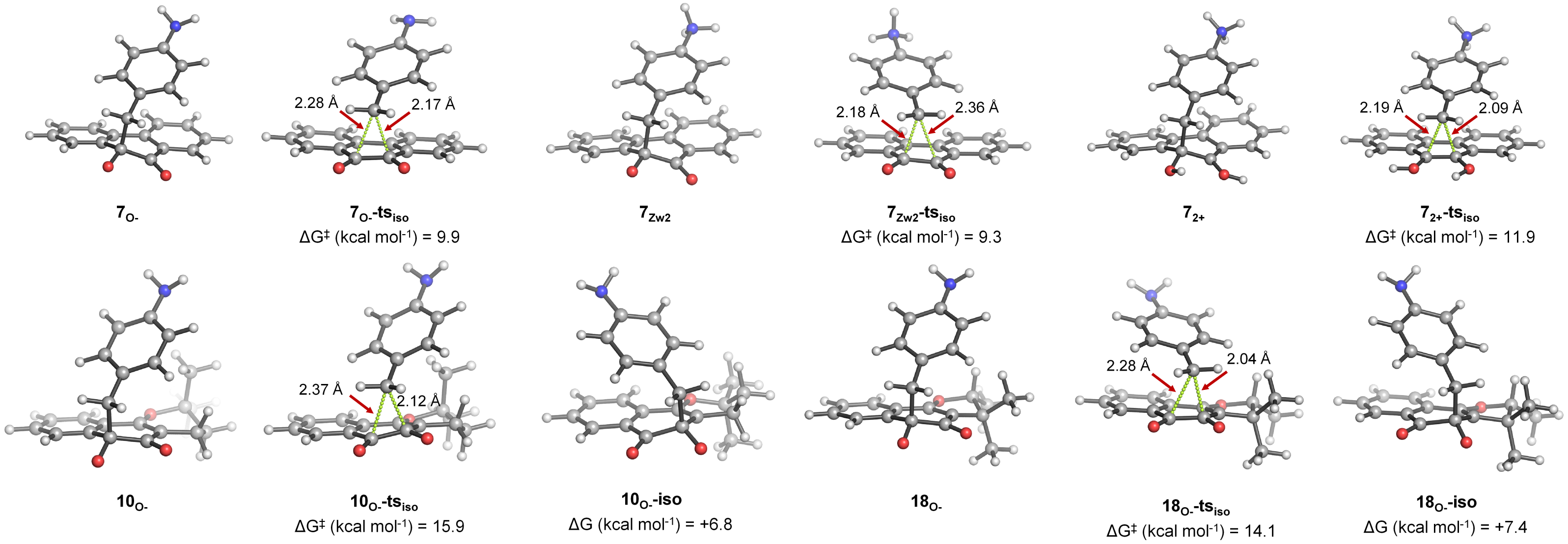

Figure 112. Lowest energy structures (reactants and transition states) for the isomerization (α-ketol rearrangement) of reactive protomers of reagents **7**, **10** and **18** calculated with PCM(H_2O)/M06-2X/6-31+G(d,p).

Table 5. Energies, entropies, and lowest frequencies for the lowest energy calculated structures (reactants and transition states) for the elimination and isomerization of reactive protomers of reagents **7**, **10**, **18** and **20** and PAB models calculated with PCM(H_2O)/M06-2X/6-31+G(d,p).

Structure	E_{elec} (Hartree)^a	E_{elec} + ZPE (Hartree)^a	H (Hartree)^a	S (cal mol^{-1} K^{-1})^b	G (Hartree)^{a,b}	Lowest freq. (cm^{-1})	# of imag. freq.
**7**$_{OH+}$	-1015.762710	-1015.418313	-1015.396804	141.9	-1015.464946	39.1	0
**7**$_{OH+}$**_ts**	-1015.749082	-1015.407490	-1015.385559	143.3	-1015.454575	-332.4	1
**7**$_{NH-}$	-1014.841881	-1014.524357	-1014.503469	140.0	-1014.570675	35.1	0
**7**$_{NH-}$**_ts**	-1014.820923	-1014.505550	-1014.484579	142.1	-1014.551901	-357.3	1
**7**$_{Zw1}$	-1015.256875	-1014.927197	-1014.905702	145.2	-1014.974078	22.9	0
**7**$_{Zw1}$**_ts**	-1015.256735	-1014.927648	-1014.906595	143.0	-1014.974264	-276.3	1
**20**$_{OH+}$	-1168.372172	-1167.988708	-1167.963853	160.3	-1168.038998	29.6	0
**20**$_{OH+}$**_ts**	-1168.350269	-1167.970694	-1167.945096	163.1	-1168.021964	-113.3	1
**20**$_{AcN-}$	-1167.474034	-1167.118025	-1167.093423	159.6	-1167.168313	25.3	0
**20**$_{AcN-}$**_ts**	-1167.429908	-1167.077791	-1167.052669	161.9	-1167.128829	-59.0	1
**20**$_{Zw1}$	-1167.885527	-1167.516527	-1167.491891	159.0	-1167.566748	29.9	0
**20**$_{Zw1}$**_ts**	-1167.875805	-1167.509292	-1167.484410	160.1	-1167.559642	-444.4	1
**10**$_{OH+}$	-1132.681201	-1132.258374	-1132.232884	159.5	-1132.308277	27.1	0
**10**$_{OH+}$**_ts**	-1132.656203	-1132.236464	-1132.210767	159.5	-1132.286707	-233.7	1
**10**$_{NH-}$	-1131.745299	-1131.349825	-1131.324997	156.7	-1131.399453	35.4	0
**10**$_{NH-}$**_ts**	-1131.721327	-1131.328097	-1131.303269	156.8	-1131.377911	-306.4	1
**10**$_{Zw1}$	-1132.175989	-1131.767139	-1131.742242	155.3	-1131.816660	40.1	0
**10**$_{Zw1}$**_ts**	-1132.171876	-1131.764843	-1131.739839	156.8	-1131.814507	-451.6	1
**18**$_{OH+}$	-1132.671031	-1132.249163	-1132.223219	161.5	-1132.299480	22.7	0
**18**$_{OH+}$**_ts**	-1132.645656	-1132.226516	-1132.200451	160.4	-1132.276995	-265.5	1
**18**$_{NH-}$	-1131.734141	-1131.339746	-1131.314325	160.7	-1131.389808	22.2	0
**18**$_{NH-}$**_ts**	-1131.711044	-1131.318767	-1131.293424	158.8	-1131.369077	-330.0	1
**18**$_{zw1}$	-1132.166182	-1131.758130	-1131.732758	158.2	-1131.808069	27.1	0
**18**$_{zw1}$**_ts**	-1132.161552	-1131.755351	-1131.729898	159.0	-1131.805482	-469.3	1
**7**$_{O-}$	-1014.852228	-1014.534700	-1014.513591	142.0	-1014.581112	29.6	0
**7**$_{O-}$**_ts**$_{iso}$	-1014.834706	-1014.519113	-1014.498162	141.5	-1014.565375	-139.5	1
**7**$_{Zw2}$	-1015.288335	-1014.955780	-1014.934478	142.8	-1015.002777	38.5	0
**7**$_{Zw2}$**_ts**$_{iso}$	-1015.271889	-1014.941295	-1014.920120	142.2	-1014.988033	-126.0	1
**7**$_{2+}$	-1016.182259	-1015.822571	-1015.800911	142.9	-1015.869493	37.1	0
**7**$_{2+}$**_ts**$_{iso}$	-1016.161269	-1015.803865	-1015.782187	143.5	-1015.850533	-214.4	1
**10**$_{O-}$	-1131.753895	-1131.358285	-1131.333311	157.4	-1131.407986	35.8	0
**10**$_{O-}$**_ts**$_{iso}$	-1131.726892	-1131.333018	-1131.308361	154.9	-1131.382580	-252.6	1
**10**$_{O-}$**_iso**	-1131.742691	-1131.347616	-1131.322459	160.7	-1131.397176	17.9	0
**18**$_{O-}$	-1131.743119	-1131.348714	-1131.323136	161.0	-1131.398897	26.2	0
**18**$_{O-}$**_ts**$_{iso}$	-1131.719247	-1131.326720	-1131.301577	156.0	-1131.376504	-143.7	1

**18_{O-}_iso**	-1131.731274	-1131.337061	-1131.311467	161.4	-1131.387114	19.0	0
**PAB-Cl**	–786.369672	–786.232282	–786.222999	91.3[c]	–786.265502[c]	47.0	0
**PAB-Cl-ts**	–786.345184	–786.209061	–786.199849	92.0[c]	–786.242502[c]	–89.6	1
**PAB-OMs**	–989.795266	–989.605031	–989.591543	111.2[c]	–989.642639[c]	44.0	0
**PAB-OMs-ts**	–989.764910	–989.577536	–989.563826	113.7[c]	–989.615782[c]	–263.0	1
**PAB-OSO_2Ph**	–1181.461713	–1181.218258	–1181.201581	129.8[c]	–1181.259163[c]	8.4	0
**PAB-OSO_2Ph-ts**	–1181.435545	–1181.194241	–1181.177658	126.5[c]	–1181.235515[c]	–299.5	1
**PAB-OTf**	–1287.415593	–1287.248798	–1287.233072	125.0[c]	–1287.288959[c]	18.0	0
**PAB-OTf-ts**	–1287.400517	–1287.236048	–1287.220224	125.6[c]	–1287.276601[c]	–344.7	1
**PAB-OAc**	–554.605299	–554.416132	–554.403375	110.6[c]	–554.453035[c]	24.5	0
**PAB-OAc-ts**	–554.547205	–554.361462	–554.348478	111.3[c]	–554.398991[c]	–112.2	1
**PAB-OTFA**	–852.243100	–852.076638	–852.062088	122.2[c]	–852.115844[c]	21.9	0
**PAB-OTFA-ts**	–852.206184	–852.042681	–852.028029	123.6[c]	–852.082001[c]	–194.6	1
**PAB-OMoc**	–629.804810	–629.609651	–629.596143	114.1[c]	–629.647649[c]	26.9	0
**PAB_OMoc-ts**	–629.756383	–629.564748	–629.551085	114.7[c]	–629.602802[c]	–212.3	1
**PAB-O(H^+)Moc**	–630.178597	–629.972916	–629.958396	118.1[c]	–630.011963[c]	34.2	0
**PAB-O(H^+)Moc-ts**	–630.178499	–629.973575	–629.959436	116.2[c]	–630.012504[c]	–189.2	1
**PAB-$C(NO_2)_3$**	–979.357157	–979.174076	–979.157205	131.4[c]	–979.215713[c]	20.5	0
**PAB-$C(NO_2)_3$-ts**	–979.312946	–979.132396	–979.115376	133.3[c]	–979.174809[c]	–210.1	1
**PAB-$C(CN)_3$**	–642.712649	–642.542142	–642.527346	118.5[c]	–642.581574[c]	27.9	0
**PAB-$C(CN)_3$-ts**	–642.679699	–642.511401	–642.496483	121.1[c]	–642.551269[c]	–236.6	1
**PAB-CH_2CHOH^+**	–479.779482	–479.582454	–479.570970	101.0[c]	–479.617801[c]	49.5	0
**PAB-CH_2CHOH^+-ts**	–479.752885	–479.557823	–479.546160	101.8[c]	–479.593409[c]	–238.6	1

[a]Energy values calculated with PCM(H_2O)/M06-2X/6-31+G(d,p). 1 Hartree = 627.51 kcal mol-1. [b]Thermal corrections at 310.15 K. [c]Thermal corrections at 298.15 K.

Table 6. Cartesian coordinates of the lowest-energy calculated structures.

Structure 7_{OH+}

C	-3.213266	-2.400024	0.586652
C	-3.078325	-1.232825	-0.162858
C	-2.012105	-0.373137	0.079661
C	-1.059835	-0.670760	1.067232
C	-1.212070	-1.842802	1.818334
C	-2.277736	-2.703561	1.575720
C	-1.799823	0.832526	-0.802542
C	0.052349	0.271316	1.308284
C	-0.037245	1.609886	0.833687
C	-1.080436	1.919016	-0.070951
C	0.940134	2.578197	1.149526
H	0.830619	3.587926	0.768004
C	2.048007	2.207493	1.883813
C	2.174593	0.875019	2.304587
C	1.192259	-0.074310	2.035705
H	-4.051313	-3.064352	0.404729
H	-3.809008	-0.985369	-0.926144
H	-0.508358	-2.081531	2.607952
H	-2.383752	-3.606241	2.167744
H	2.823168	2.929128	2.112853
H	3.058704	0.573683	2.857515
H	1.332678	-1.090809	2.383972
O	-1.347714	3.135760	-0.385493
O	-2.990089	1.403941	-1.290059
H	-3.147865	1.148146	-2.209810
C	-0.875325	0.434593	-2.046414
H	-1.504583	-0.274800	-2.597068
H	-0.763873	1.341225	-2.650955
C	0.456618	-0.177928	-1.734905
C	0.578306	-1.545109	-1.457598
C	1.611871	0.611140	-1.660421
C	1.795229	-2.100632	-1.083945
H	-0.299379	-2.185396	-1.519035
C	2.834715	0.070959	-1.288206
H	1.553282	1.673799	-1.888117
C	2.942727	-1.296375	-0.979585
H	1.863182	-3.162527	-0.865094
H	3.714224	0.705711	-1.227063
N	4.143041	-1.826970	-0.532000
H	4.975987	-1.313908	-0.787676
H	4.254909	-2.825671	-0.643333
H	-2.121330	3.146074	-0.994701

Structure 7_{OH+}-ts

C	-3.127697	-2.530758	0.499580
C	-3.141958	-1.265861	-0.065170
C	-2.092716	-0.369248	0.188178
C	-1.012137	-0.746927	1.012973
C	-1.025850	-2.034145	1.581403
C	-2.062716	-2.915394	1.325200
C	-2.046978	0.938159	-0.457893
C	0.068865	0.211207	1.268496
C	-0.068221	1.564349	0.860793
C	-1.186317	1.925987	0.054319
C	0.929768	2.518732	1.154411
H	0.789728	3.544406	0.830593
C	2.077348	2.133369	1.815032
C	2.246060	0.786291	2.177184
C	1.260268	-0.152552	1.918795
H	-3.946055	-3.216674	0.308778
H	-3.980334	-0.970504	-0.687897
H	-0.222703	-2.347484	2.238124
H	-2.053232	-3.902505	1.774834
H	2.854197	2.858036	2.031786
H	3.161637	0.472899	2.668799
H	1.431669	-1.182524	2.208790
O	-1.326477	3.188294	-0.319850
O	-3.144836	1.467123	-1.086151
H	-3.523289	0.869680	-1.745658
C	-0.778602	0.551648	-2.078677
H	-1.557202	-0.099503	-2.471537
H	-0.789273	1.568960	-2.464904
C	0.466041	-0.045880	-1.764614
C	0.572457	-1.445446	-1.543417
C	1.636740	0.742321	-1.592666
C	1.759632	-2.023537	-1.165918
H	-0.311983	-2.066807	-1.666633
C	2.830082	0.180271	-1.216254
H	1.580796	1.814130	-1.766646
C	2.915685	-1.218995	-0.979825
H	1.823641	-3.093724	-0.996585
H	3.714704	0.795494	-1.085366
N	4.080104	-1.774128	-0.592816
H	4.909535	-1.214128	-0.467462
H	4.158283	-2.766496	-0.431158
H	-2.148866	3.290747	-0.831722

Structure 7_{NH-}

C	-3.350168	-2.145016	0.719767
C	-3.070032	-1.135806	-0.197800
C	-1.994768	-0.270324	0.005043
C	-1.175866	-0.411985	1.137555
C	-1.476428	-1.425453	2.058880
C	-2.547430	-2.288806	1.851695
C	-1.664446	0.750615	-1.055150
C	-0.049394	0.528519	1.357976
C	0.031414	1.717535	0.607039
C	-0.923653	1.953075	-0.492163
C	1.029931	2.664633	0.850772
H	1.049547	3.566734	0.247132
C	1.993857	2.428023	1.821624
C	1.949203	1.234425	2.547220
C	0.939115	0.302040	2.325077
H	-4.191871	-2.810429	0.556460
H	-3.688205	-1.008356	-1.080760
H	-0.881858	-1.537903	2.958844
H	-2.760644	-3.064998	2.579561
H	2.782335	3.150811	2.001871
H	2.708888	1.027051	3.294372
H	0.938937	-0.614113	2.904759
O	-1.084711	3.045778	-1.019157
O	-2.850702	1.190179	-1.684892
H	-2.669951	2.085708	-2.012796
C	-0.746019	0.098359	-2.158140
H	-1.391298	-0.663657	-2.611684
H	-0.586502	0.883903	-2.908444
C	0.562750	-0.492785	-1.718098
C	0.657040	-1.801831	-1.221023
C	1.754156	0.253257	-1.741599
C	1.854096	-2.337951	-0.766580
H	-0.242909	-2.417534	-1.183826
C	2.961054	-0.258432	-1.294563
H	1.726472	1.276396	-2.120142
C	3.083953	-1.591847	-0.771503
H	1.875040	-3.361948	-0.395466
H	3.857774	0.356904	-1.334064
N	4.271432	-2.052335	-0.348624
H	4.147324	-3.011350	-0.019228

Structure 7_{NH-}-ts

C	-2.960725	-2.574593	0.699065
C	-3.042318	-1.370458	0.020116
C	-2.060779	-0.376722	0.206686
C	-0.969797	-0.613679	1.076990
C	-0.919002	-1.842167	1.761600
C	-1.891709	-2.812392	1.577787
C	-2.081748	0.841611	-0.560229
C	0.039803	0.435901	1.252340
C	-0.166558	1.721712	0.693780
C	-1.311190	1.981762	-0.180204
C	0.768494	2.748362	0.909334
H	0.570928	3.719058	0.464921
C	1.919649	2.516192	1.640317
C	2.153860	1.233732	2.161002
C	1.229249	0.218900	1.975601
H	-3.726045	-3.331416	0.555615
H	-3.863753	-1.173041	-0.661305
H	-0.108187	-2.043866	2.453410
H	-1.827692	-3.751327	2.118354
H	2.645436	3.308883	1.792190
H	3.066492	1.031172	2.713767
H	1.446261	-0.762426	2.383545
O	-1.567049	3.109844	-0.687052
O	-3.197995	1.104871	-1.317230
H	-3.130013	2.053081	-1.532902
C	-0.713268	0.262688	-2.120453
H	-1.508885	-0.408650	-2.434983
H	-0.743154	1.256501	-2.565370
C	0.522641	-0.282846	-1.751217
C	0.666219	-1.667362	-1.405980
C	1.697981	0.529749	-1.629803
C	1.854265	-2.195279	-0.992848
H	-0.215336	-2.304969	-1.471575
C	2.893357	0.022024	-1.217966
H	1.618078	1.587804	-1.876167
C	3.051269	-1.381680	-0.860383
H	1.935643	-3.248716	-0.737512
H	3.765856	0.667464	-1.136887
N	4.173562	-1.925812	-0.449390
H	4.901632	-1.209325	-0.418332

Structure 7_{Zw1}

C	3.267643	-3.108632	-0.255742
C	1.999079	-2.701475	0.138949
C	1.684028	-1.341892	0.198934
C	2.634715	-0.368619	-0.164071
C	3.911976	-0.801643	-0.558868
C	4.225665	-2.152751	-0.604386
C	0.295895	-0.928323	0.560057
C	2.286111	1.061219	-0.085816
C	1.070658	1.466683	0.529021
C	0.177921	0.459325	0.987279
C	0.737629	2.831327	0.675671
H	-0.199646	3.098152	1.152087
C	1.593120	3.800635	0.194581
C	2.785647	3.412172	-0.436423
C	3.125697	2.072856	-0.573179
H	3.511475	-4.165192	-0.289166
H	1.246053	-3.435433	0.409266
H	4.677568	-0.080432	-0.819811
H	5.220046	-2.462652	-0.907441
H	1.342828	4.850905	0.291268
H	3.456394	4.169997	-0.827886
H	4.052692	1.821295	-1.073357
O	-0.899120	0.792057	1.629174
O	-0.402128	-1.792959	1.423177
H	0.148368	-1.987144	2.197144
C	-0.625731	-0.978938	-0.845739
H	-0.092493	-0.356139	-1.568464
H	-0.485407	-2.035542	-1.092063
C	-2.036690	-0.620045	-0.676840
C	-2.514825	0.681852	-0.982326
C	-2.990809	-1.554986	-0.204052
C	-3.836197	1.027532	-0.843153
H	-1.806844	1.424815	-1.350783
C	-4.317413	-1.227574	-0.048682
H	-2.657141	-2.562907	0.035209
C	-4.827362	0.092510	-0.357147
H	-4.171764	2.029414	-1.098186
H	-5.021289	-1.977063	0.307615
N	-6.089521	0.468550	-0.229837
H	-1.428624	-0.012799	1.813652
H	-6.636136	-0.319311	0.122557

Structure 7_{Zw1}-ts

C	3.309917	-3.072247	-0.269711
C	2.046050	-2.685986	0.154842
C	1.709802	-1.329694	0.222869
C	2.636709	-0.340384	-0.163506
C	3.911436	-0.755332	-0.589028
C	4.244962	-2.100378	-0.641458
C	0.336736	-0.933763	0.616892
C	2.264715	1.080955	-0.078751
C	1.045759	1.461396	0.543636
C	0.177593	0.436471	1.018735
C	0.685809	2.819511	0.683973
H	-0.253455	3.069369	1.165633
C	1.518595	3.804814	0.195003
C	2.717261	3.441181	-0.440052
C	3.082236	2.109445	-0.573908
H	3.569888	-4.124761	-0.310118
H	1.310880	-3.431684	0.441493
H	4.658728	-0.022568	-0.870181
H	5.236333	-2.395141	-0.968677
H	1.245981	4.849901	0.289743
H	3.370199	4.211844	-0.836503
H	4.011375	1.874251	-1.078383
O	-0.922322	0.760811	1.642124
O	-0.382418	-1.826868	1.424092
H	0.143649	-2.042280	2.209355
C	-0.627663	-0.976024	-0.869226
H	-0.072461	-0.333170	-1.554932
H	-0.448392	-2.032865	-1.078714
C	-2.021424	-0.638448	-0.691012
C	-2.511529	0.670501	-0.964157
C	-2.971001	-1.596716	-0.232777
C	-3.833334	0.999156	-0.819737
H	-1.808008	1.426732	-1.312945
C	-4.294501	-1.285887	-0.070160
H	-2.623997	-2.604825	-0.015669
C	-4.816208	0.037618	-0.353219
H	-4.173497	2.005553	-1.053914
H	-5.000357	-2.038877	0.270235
N	-6.098226	0.289858	-0.186555
H	-6.282771	1.263072	-0.437006
H	-1.430116	-0.052236	1.841238

Structure 20_{OH+}

C	3.419340	2.878061	0.535675
C	3.485650	1.685608	-0.182369
C	2.594657	0.654079	0.101282

C	1.601860	0.811622	1.082201
C	1.552190	2.013226	1.799671
C	2.453150	3.037725	1.528564
C	2.605570	-0.594700	-0.741366
C	0.647029	-0.285951	1.338438
C	0.929052	-1.602303	0.876336
C	2.012382	-1.766186	-0.016787
C	0.096021	-2.697715	1.194238
H	0.350793	-3.684433	0.822114
C	-1.058178	-2.483103	1.918384
C	-1.375281	-1.178849	2.327482
C	-0.535909	-0.101463	2.056235
H	4.121447	3.676557	0.321607
H	4.227418	1.560162	-0.965407
H	0.817339	2.151093	2.584653
H	2.402423	3.960498	2.096221
H	-1.725204	-3.305440	2.148445
H	-2.297397	-1.001470	2.871994
H	-0.823604	0.886646	2.395014
O	2.430769	-2.937965	-0.339821
O	3.864697	-0.966611	-1.250121
H	4.551534	-0.835253	-0.577818
C	1.708911	-0.374912	-2.038527
H	2.253574	0.406530	-2.576783
H	1.769412	-1.297863	-2.623781
C	0.283145	0.020384	-1.783260
C	-0.063117	1.355750	-1.545862
C	-0.724685	-0.944526	-1.703782
C	-1.359493	1.702279	-1.190989
H	0.695541	2.132011	-1.614180
C	-2.030068	-0.617940	-1.347229
H	-0.486701	-1.986459	-1.908636
C	-2.349579	0.717265	-1.066180
H	-1.606095	2.741869	-0.994042
H	-2.787082	-1.385400	-1.277226
N	-3.634987	1.142616	-0.684123
C	-4.678047	0.382639	-0.229644
H	-3.770943	2.144818	-0.665635
O	-4.631556	-0.839822	-0.142758
C	-5.901556	1.162696	0.191944
H	-6.788145	0.628666	-0.151565
H	-5.927807	1.196811	1.284639
H	-5.916993	2.183191	-0.192545
H	3.195843	-2.857780	-0.953338

Structure 20_{OH+}-ts

C	-3.186472	3.073296	-0.391884
C	-3.503230	1.801146	0.044495
C	-2.671522	0.710082	-0.278953
C	-1.480620	0.914390	-1.017145
C	-1.192758	2.221517	-1.459799
C	-2.028122	3.280956	-1.157537
C	-2.977171	-0.620057	0.188342
C	-0.587264	-0.216120	-1.276840
C	-0.970612	-1.525720	-0.888682
C	-2.201102	-1.708136	-0.181258
C	-0.128574	-2.629806	-1.140468
H	-0.452480	-3.618376	-0.833495
C	1.095263	-2.443007	-1.751047
C	1.499972	-1.147087	-2.109861
C	0.673374	-0.058750	-1.883069
H	-3.832220	3.907856	-0.140899
H	-4.391193	1.655032	0.651823
H	-0.301249	2.413013	-2.045171
H	-1.782004	4.277182	-1.509238
H	1.748097	-3.289084	-1.936357
H	2.471230	-0.991887	-2.569005
H	1.023967	0.926602	-2.166956
O	-2.560584	-2.952180	0.160249
O	-4.119979	-0.919126	0.871822
H	-4.872379	-0.395066	0.563248
C	-1.576087	-0.582883	2.166216
H	-2.337444	0.147302	2.424057
H	-1.789842	-1.622690	2.400446
C	-0.277643	-0.178800	1.875976
C	0.036681	1.197958	1.671068
C	0.754423	-1.141083	1.671857
C	1.291165	1.573989	1.268181
H	-0.735652	1.948059	1.822266
C	2.013782	-0.775541	1.264733
H	0.531273	-2.191157	1.839853
C	2.290731	0.594906	1.030927
H	1.520631	2.621795	1.101721
H	2.780407	-1.517176	1.101976
N	3.509811	1.052976	0.585610
C	4.628549	0.325657	0.187731
H	3.592564	2.059353	0.495640
O	4.661929	-0.890564	0.204887
C	5.777378	1.187482	-0.267749
H	6.618179	0.544210	-0.519257
H	5.486154	1.770125	-1.146147
H	6.069357	1.884912	0.521731
H	-3.447846	-2.932199	0.557417

Structure 20_{AcN-}

C	-3.539063	-2.727973	0.736706
C	-3.495473	-1.654158	-0.148700
C	-2.602134	-0.602260	0.055415
C	-1.728910	-0.617221	1.155634
C	-1.792655	-1.698714	2.045631
C	-2.682450	-2.747534	1.837675
C	-2.520988	0.499667	-0.974313
C	-0.788689	0.510847	1.371712
C	-0.960237	1.715431	0.661905
C	-1.991267	1.802900	-0.388766
C	-0.138982	2.821945	0.897485
H	-0.311307	3.729052	0.326571
C	0.897320	2.736885	1.817349
C	1.101391	1.536441	2.503265
C	0.266779	0.442474	2.290733
H	-4.239715	-3.540216	0.572260
H	-4.158742	-1.622070	-1.007297
H	-1.153864	-1.722337	2.921503
H	-2.712954	-3.573769	2.540551
H	1.550929	3.585160	1.989909
H	1.920561	1.449999	3.210250
H	0.459655	-0.473731	2.837270
O	-2.379974	2.860714	-0.863289
O	-3.798076	0.729062	-1.530841
H	-3.808617	1.655275	-1.821646
C	-1.559860	0.075528	-2.143516
H	-2.064793	-0.778332	-2.608910
H	-1.571458	0.903687	-2.862539
C	-0.146010	-0.280115	-1.767319
C	0.187349	-1.563902	-1.316546
C	0.880171	0.668241	-1.820942
C	1.483235	-1.871802	-0.920966
H	-0.584138	-2.331202	-1.269486
C	2.181409	0.375169	-1.417216
H	0.653544	1.673236	-2.176554
C	2.523026	-0.914387	-0.944285
H	1.725698	-2.876126	-0.583237
H	2.946401	1.139251	-1.460310
N	3.792250	-1.353122	-0.576452

C	4.714699	-0.496509	-0.141842
O	4.599273	0.743029	0.073298
C	6.073640	-1.119246	0.157511
H	6.839063	-0.625495	-0.449975
H	6.331288	-0.941912	1.206672
H	6.089091	-2.191294	-0.042860

Structure 20_{AcN-}-ts

C	3.153754	3.057317	0.634029
C	3.518020	1.866429	0.040632
C	2.754925	0.688699	0.250954
C	1.588992	0.743465	1.066010
C	1.256471	1.975797	1.667817
C	2.014301	3.113967	1.461759
C	3.101328	-0.531660	-0.386738
C	0.794140	-0.466690	1.237582
C	1.218989	-1.683038	0.637761
C	2.416087	-1.741442	-0.194403
C	0.448331	-2.852269	0.800862
H	0.800910	-3.760489	0.322022
C	-0.719946	-2.840388	1.537604
C	-1.150805	-1.639373	2.128805
C	-0.409615	-0.481665	1.977529
H	3.747653	3.951045	0.466247
H	4.395430	1.811034	-0.595884
H	0.379471	2.046926	2.302343
H	1.729459	4.048090	1.935288
H	-1.306386	-3.746810	1.654212
H	-2.073066	-1.615119	2.701499
H	-0.776661	0.430589	2.435509
O	2.828465	-2.810164	-0.774570
O	4.213721	-0.579661	-1.185089
H	4.256266	-1.515399	-1.461322
C	1.272560	-0.124014	-2.296182
H	2.020764	0.649305	-2.440693
H	1.510004	-1.123696	-2.650206
C	0.036081	0.176834	-1.806555
C	-0.295740	1.508541	-1.350404
C	-0.991001	-0.834451	-1.679116
C	-1.522951	1.797551	-0.852548
H	0.472700	2.277077	-1.408041
C	-2.220413	-0.558396	-1.178239
H	-0.751112	-1.844288	-2.004643
C	-2.560692	0.788882	-0.743928
H	-1.773019	2.798356	-0.514353
H	-2.968314	-1.336023	-1.091450
N	-3.748044	1.190451	-0.333669
C	-4.770165	0.316091	-0.035724
O	-4.630861	-0.795259	0.476815
C	-6.146447	0.881851	-0.305697
H	-6.912333	0.144284	-0.066779
H	-6.296551	1.780623	0.298756
H	-6.226719	1.179782	-1.354955

Structure 20_{Zw1}

C	-3.425466	-2.864445	0.565154
C	-3.483573	-1.688358	-0.179839
C	-2.594516	-0.651233	0.090127
C	-1.610917	-0.788820	1.082770
C	-1.569335	-1.973961	1.827751
C	-2.468954	-3.003164	1.570966
C	-2.585790	0.577239	-0.776643
C	-0.660765	0.315367	1.323025
C	-0.946891	1.622888	0.838845
C	-2.016990	1.763883	-0.072002
C	-0.122535	2.727378	1.149118
H	-0.379475	3.707070	0.760252
C	1.024686	2.530189	1.887992
C	1.347614	1.232686	2.316090
C	0.517724	0.147229	2.052523
H	-4.126193	-3.667112	0.362016
H	-4.216773	-1.580283	-0.973475
H	-0.841097	-2.094843	2.621810
H	-2.425152	-3.913281	2.159385
H	1.684827	3.359563	2.113051
H	2.266787	1.068767	2.869767
H	0.808885	-0.835194	2.404634
O	-2.427368	2.930105	-0.436811
O	-3.826853	0.928271	-1.342908
H	-4.536565	0.812514	-0.692182
C	-1.634849	0.324360	-2.044070
H	-2.176779	-0.464839	-2.574699
H	-1.684411	1.238064	-2.645618
C	-0.221917	-0.071256	-1.751623
C	0.122622	-1.404970	-1.483384
C	0.797403	0.885505	-1.676776
C	1.416305	-1.748566	-1.123659
H	-0.641789	-2.178438	-1.542743
C	2.096662	0.555902	-1.305230
H	0.564522	1.926674	-1.899403
C	2.447394	-0.784357	-0.998184
H	1.666339	-2.786222	-0.919950
H	2.851861	1.326172	-1.237945
N	3.698375	-1.265513	-0.644359
C	4.668783	-0.447683	-0.227348
O	4.618463	0.796994	-0.033120
C	5.995963	-1.135435	0.070636
H	6.782445	-0.682633	-0.541431
H	6.264514	-0.964717	1.118130
H	5.958104	-2.207957	-0.122783
H	-3.178566	2.830073	-1.063021

Structure 20_{Zw1}-ts

C	3.205050	3.022500	0.568747
C	3.436405	1.820550	-0.081907
C	2.622527	0.710607	0.185859
C	1.539373	0.813095	1.081890
C	1.332227	2.039759	1.738341
C	2.152197	3.128143	1.487321
C	2.807365	-0.544576	-0.536603
C	0.670233	-0.350021	1.292916
C	1.051966	-1.627120	0.800802
C	2.203434	-1.716426	-0.030513
C	0.260207	-2.770482	1.045556
H	0.585372	-3.729423	0.656432
C	-0.924838	-2.650044	1.739279
C	-1.336170	-1.382732	2.187244
C	-0.552727	-0.259206	1.977651
H	3.840818	3.878044	0.366841
H	4.242294	1.738334	-0.804545
H	0.526959	2.144606	2.456455
H	1.975708	4.064813	2.005334
H	-1.547574	-3.519673	1.917241
H	-2.283761	-1.279049	2.706354
H	-0.910502	0.701054	2.329984
O	2.598997	-2.899625	-0.466725
O	3.954208	-0.757562	-1.282096
H	4.740768	-0.547012	-0.756035
C	1.536914	-0.321560	-2.085421
H	2.194679	0.457896	-2.464777
H	1.701970	-1.301440	-2.531589
C	0.205025	0.039990	-1.748551
C	-0.147410	1.389488	-1.469912
C	-0.816322	-0.938539	-1.626358

C	-1.422383	1.721891	-1.090710
H	0.616012	2.161455	-1.552515
C	-2.096724	-0.618450	-1.237743
H	-0.571682	-1.975680	-1.850005
C	-2.453349	0.738449	-0.939120
H	-1.685590	2.754991	-0.884896
H	-2.847752	-1.389798	-1.147654
N	-3.676079	1.210378	-0.586821
C	-4.684799	0.389998	-0.208722
O	-4.629211	-0.835429	0.020863
C	-6.017613	1.091204	-0.000420
H	-6.797022	0.554256	-0.548075
H	-6.280238	1.051008	1.061684
H	-5.988750	2.131952	-0.322738
H	3.405365	-2.787166	-1.003839

Structure 10_{OH+}

C	-3.492913	-0.496475	-2.382533
C	-3.422584	-0.071291	-1.057310
C	-2.267370	-0.297052	-0.315486
C	-1.181058	-0.952435	-0.909791
C	-1.247499	-1.365906	-2.247364
C	-2.403612	-1.135452	-2.980507
C	-2.126258	0.271850	1.072891
C	0.010959	-1.224585	-0.104203
C	0.058501	-1.013439	1.288434
C	-1.036509	-0.407964	1.859592
C	1.218826	-1.503564	2.113619
H	0.841277	-1.956669	3.033720
C	2.024908	-2.521322	1.311413
C	2.298888	-2.030281	-0.105786
H	-4.400413	-0.326011	-2.952050
H	-4.271057	0.431462	-0.603880
H	-0.396887	-1.865463	-2.696463
H	-2.461237	-1.456330	-4.014609
H	2.982320	-2.725182	1.797447
O	-1.100828	-0.292207	3.163305
O	-3.301406	0.152528	1.848125
H	-3.827304	0.962847	1.798587
C	-1.734326	1.799365	0.971853
H	-2.544092	2.260529	0.394505
H	-1.778646	2.192946	1.993353
C	-0.396920	2.107848	0.358972
C	-0.209557	2.136659	-1.027737
C	0.711295	2.366203	1.174851
C	1.039497	2.394538	-1.582811
H	-1.054313	1.955936	-1.689586
C	1.961244	2.640572	0.634221
H	0.595219	2.357832	2.256836
C	2.146837	2.650799	-0.758363
H	1.160886	2.406303	-2.662391
H	2.805605	2.839075	1.288657
N	3.409629	2.843874	-1.301731
H	4.066786	3.342876	-0.717007
H	3.430992	3.165354	-2.260311
H	-1.986428	0.038507	3.413874
O	0.997790	-1.754380	-0.760321
H	1.475754	-3.467490	1.250255
H	1.842278	-0.652373	2.412663
C	2.924438	-3.107830	-0.971569
H	3.032701	-2.755418	-2.000309
H	3.915404	-3.350075	-0.579504
H	2.312492	-4.013234	-0.964763
C	3.084382	-0.727561	-0.156361
H	3.228531	-0.412471	-1.193072
H	2.567606	0.076228	0.375307
H	4.063667	-0.879897	0.305842

Structure 10_{OH+}-ts

C	2.977380	1.390561	2.249628
C	3.169372	0.408830	1.296585
C	2.065793	-0.304407	0.784130
C	0.762376	0.016219	1.232641
C	0.589385	1.018064	2.211534
C	1.682207	1.694323	2.714179
C	2.201599	-1.281580	-0.254192
C	-0.366563	-0.695393	0.678683
C	-0.214760	-1.733227	-0.228542
C	1.092026	-2.049744	-0.639275
C	-1.399128	-2.476269	-0.788445
C	-2.678653	-1.681044	-0.539269
C	-2.731892	-1.120771	0.881002
H	3.831588	1.933139	2.641451
H	4.167248	0.189627	0.929977
H	-0.413260	1.251871	2.551296
H	1.545129	2.465743	3.464463
O	1.252955	-3.064079	-1.501541
O	3.433137	-1.717672	-0.697210
H	3.961514	-2.057665	0.040382
C	1.868390	0.140868	-2.025315
H	2.901062	0.412980	-1.826375
H	1.709235	-0.671933	-2.729606
C	0.844108	1.046579	-1.762204
C	1.089955	2.233023	-1.003043
C	-0.498898	0.792368	-2.178213
C	0.081667	3.095895	-0.679213
H	2.104184	2.439663	-0.670017
C	-1.518564	1.651670	-1.869650
H	-0.703889	-0.103256	-2.760635
C	-1.254960	2.820131	-1.097173
H	0.279379	3.990138	-0.097088
H	-2.533131	1.457049	-2.203134
N	-2.248391	3.654940	-0.770956
H	-3.199790	3.476174	-1.057299
H	-2.074774	4.486859	-0.226148
H	2.204265	-3.190014	-1.660556
O	-1.561857	-0.278476	1.110690
C	-2.719996	-2.217318	1.941951
H	-2.776986	-1.773416	2.939172
H	-3.580380	-2.877585	1.800882
H	-1.808953	-2.819840	1.883152
C	-3.912691	-0.182000	1.059394
H	-3.899048	0.260739	2.059029
H	-3.875372	0.620233	0.316273
H	-4.847173	-0.735370	0.934358
H	-3.560136	-2.307051	-0.703613
H	-2.737068	-0.841135	-1.239279
H	-1.255055	-2.637390	-1.861527
H	-1.462655	-3.473000	-0.335157

Structure 10_{NH-}

C	-3.517927	-0.783910	-2.237241
C	-3.415737	-0.255917	-0.951527
C	-2.236951	-0.399119	-0.220836
C	-1.152102	-1.081731	-0.785461
C	-1.251791	-1.599770	-2.082530
C	-2.430711	-1.449616	-2.806169
C	-2.095337	0.269883	1.119682
C	0.075733	-1.269128	0.009990
C	0.179467	-0.918017	1.325040
C	-0.946876	-0.277618	1.965077
C	1.401983	-1.242412	2.135049
H	1.095777	-1.605158	3.120427
C	2.236508	-2.294650	1.410693

C	2.383252	-1.960066	-0.073218
H	-4.441627	-0.671755	-2.796121
H	-4.252875	0.273144	-0.506270
H	-0.404222	-2.120005	-2.513883
H	-2.503070	-1.854163	-3.810475
H	3.233466	-2.381729	1.852637
O	-1.003439	-0.081778	3.182351
O	-3.307519	0.131756	1.840533
H	-3.078584	0.286106	2.771352
C	-1.815398	1.800571	0.916373
H	-2.673461	2.174442	0.344704
H	-1.870406	2.237164	1.921925
C	-0.511633	2.168904	0.265015
C	-0.336400	2.162016	-1.126829
C	0.619200	2.492660	1.035317
C	0.884632	2.457234	-1.720432
H	-1.185671	1.913898	-1.765315
C	1.844830	2.796058	0.465420
H	0.528058	2.503798	2.122464
C	2.053781	2.793816	-0.955473
H	0.967477	2.442659	-2.806661
H	2.691475	3.042741	1.103450
N	3.258288	3.092546	-1.469499
H	3.198794	3.031938	-2.487593
O	1.055214	-1.873634	-0.671977
H	1.750174	-3.273964	1.493681
H	1.985255	-0.327523	2.308430
C	3.071126	-3.083705	-0.830504
H	3.093428	-2.861963	-1.901091
H	4.099797	-3.190213	-0.475559
H	2.546144	-4.030299	-0.675209
C	3.075979	-0.621962	-0.308171
H	3.192116	-0.446398	-1.381743
H	2.503502	0.211270	0.111566
H	4.068615	-0.633702	0.152947

Structure 10$_{NH-}$-ts

C	3.128252	1.220615	2.303981
C	3.296570	0.412002	1.195413
C	2.193376	-0.265829	0.629219
C	0.908378	-0.095254	1.205380
C	0.761370	0.731477	2.337630
C	1.852320	1.385034	2.880899
C	2.292445	-1.023312	-0.573696
C	-0.208882	-0.809131	0.626358
C	-0.064762	-1.696837	-0.413009
C	1.231416	-1.881704	-1.013568
C	-1.243631	-2.431551	-0.988182
C	-2.546953	-1.773400	-0.543361
C	-2.517744	-1.425240	0.945197
H	3.984828	1.731348	2.734153
H	4.276426	0.286526	0.744936
H	-0.225398	0.852759	2.771878
H	1.727867	2.021268	3.751411
O	1.454132	-2.694107	-1.967237
O	3.535434	-1.298810	-1.091273
H	3.363279	-1.983717	-1.764692
C	1.700565	0.611463	-1.942787
H	2.685806	1.011930	-1.718928
H	1.654773	-0.091370	-2.772895
C	0.571144	1.353483	-1.603209
C	0.642276	2.460987	-0.692498
C	-0.733014	1.030395	-2.113877
C	-0.454022	3.195066	-0.351381
H	1.612771	2.705326	-0.260810
C	-1.839583	1.754099	-1.791116
H	-0.821356	0.175852	-2.784756
C	-1.776769	2.893256	-0.885224
H	-0.363807	4.029853	0.340590
H	-2.814434	1.495766	-2.196934
N	-2.867931	3.561889	-0.605453
H	-2.642204	4.319018	0.042933
O	-1.404480	-0.536649	1.210027
C	-2.351739	-2.662591	1.825652
H	-2.374897	-2.376449	2.880964
H	-3.164494	-3.370298	1.636215
H	-1.401254	-3.164992	1.624290
C	-3.749361	-0.627286	1.342962
H	-3.682010	-0.319942	2.390676
H	-3.838036	0.266668	0.717797
H	-4.648083	-1.237231	1.215087
H	-1.218194	-3.488530	-0.689986
H	-1.164447	-2.428836	-2.081022
H	-2.696950	-0.841892	-1.100489
H	-3.405276	-2.424210	-0.739082

Structure 10$_{Zw1}$

C	3.216436	1.488997	2.188878
C	3.336320	0.721989	1.030656
C	2.230332	0.033031	0.543989
C	1.001254	0.120901	1.213468
C	0.879459	0.903813	2.368997
C	1.988816	1.586495	2.851773
C	2.234298	-0.650497	-0.783396
C	-0.121836	-0.663246	0.703634
C	0.057180	-1.732573	-0.201078
C	1.270679	-1.783847	-0.841415
C	-1.072694	-2.677692	-0.511111
C	-2.401877	-1.962090	-0.274559
C	-2.452065	-1.259173	1.077848
H	4.082794	2.015337	2.575959
H	4.285501	0.655917	0.508631
H	-0.076939	0.965780	2.876091
H	1.903048	2.191181	3.748170
O	1.529983	-2.753171	-1.701072
O	3.494364	-1.130623	-1.190776
H	3.906504	-0.514780	-1.812588
C	1.714978	0.436902	-1.887678
H	2.545598	1.153243	-1.910287
H	1.700300	-0.114501	-2.834761
C	0.429543	1.124886	-1.623017
C	0.363532	2.303281	-0.840605
C	-0.790432	0.654732	-2.156468
C	-0.823009	2.947466	-0.578350
H	1.286321	2.702208	-0.417550
C	-1.991863	1.286187	-1.915780
H	-0.779943	-0.243862	-2.775683
C	-2.086836	2.473089	-1.095779
H	-0.832840	3.845731	0.034185
H	-2.908252	0.888280	-2.349267
N	-3.211942	3.115648	-0.811756
H	-3.990724	2.632479	-1.263733
H	2.450486	-2.649969	-2.010735
O	-1.276079	-0.378503	1.238657
C	-2.399800	-2.227277	2.253874
H	-2.418154	-1.675200	3.196796
H	-3.268823	-2.889808	2.217350
H	-1.494275	-2.840098	2.227602
C	-3.635202	-0.311920	1.167578
H	-3.634974	0.210284	2.128160
H	-3.589798	0.425455	0.360111
H	-4.563439	-0.882910	1.080070
H	-0.995353	-3.579967	0.105900
H	-1.000363	-3.001400	-1.552903

H	-2.559121	-1.214003	-1.056852
H	-3.233387	-2.671279	-0.315602

Structure 10_{Zw1}-ts

C	3.165699	1.531893	2.173867
C	3.323326	0.679419	1.091697
C	2.223620	-0.038539	0.602352
C	0.961354	0.118329	1.207247
C	0.811942	0.995838	2.298487
C	1.905668	1.695800	2.774950
C	2.282407	-0.821099	-0.625861
C	-0.151134	-0.661542	0.701648
C	0.024164	-1.711743	-0.205273
C	1.277766	-1.831890	-0.790295
C	-1.120413	-2.607866	-0.599435
C	-2.446865	-1.912098	-0.302292
C	-2.463710	-1.270108	1.082444
H	4.021874	2.076423	2.558886
H	4.294520	0.557477	0.622550
H	-0.165336	1.113470	2.753141
H	1.792330	2.369997	3.617324
O	1.489152	-2.801804	-1.682929
O	3.525826	-1.239201	-1.093470
H	3.929660	-0.568201	-1.662050
C	1.695825	0.464477	-1.936900
H	2.596372	1.074682	-1.841509
H	1.712892	-0.198739	-2.803113
C	0.473399	1.137617	-1.665179
C	0.433638	2.323120	-0.862448
C	-0.776265	0.673378	-2.180800
C	-0.728973	2.982350	-0.593959
H	1.370429	2.695275	-0.447303
C	-1.951907	1.321962	-1.932113
H	-0.778961	-0.226644	-2.796410
C	-2.009386	2.522959	-1.110882
H	-0.729774	3.878849	0.020040
H	-2.884518	0.947259	-2.349643
N	-3.105250	3.184531	-0.827804
H	-3.901983	2.721610	-1.270190
H	2.421481	-2.762336	-1.961637
O	-1.330465	-0.352581	1.221589
C	-2.334786	-2.294442	2.205412
H	-2.357645	-1.792225	3.176000
H	-3.166227	-3.003186	2.156012
H	-1.397904	-2.854039	2.127308
C	-3.684507	-0.383829	1.260476
H	-3.658376	0.110709	2.235389
H	-3.712831	0.378891	0.475775
H	-4.591991	-0.990561	1.199908
H	-1.049793	-3.563134	-0.065536
H	-1.048842	-2.843242	-1.665454
H	-2.623382	-1.126907	-1.043036
H	-3.276534	-2.622387	-0.362196

Structure 18_{OH+}

C	3.928289	-0.010020	1.650370
C	3.474049	-0.518670	0.432480
C	2.110499	-0.677550	0.215580
C	1.212869	-0.318290	1.236130
C	1.665459	0.205880	2.452750
C	3.030030	0.361200	2.655270
C	1.581769	-1.080250	-1.144510
C	-0.186891	-0.561250	0.978520
C	-0.692261	-1.277510	-0.116790
C	0.187399	-1.665820	-1.085940
C	-2.155621	-1.579009	0.143840
C	-2.436801	-0.497739	1.223060
H	4.994929	0.100070	1.815560
H	4.185989	-0.794020	-0.338960
H	0.948660	0.479440	3.219790
H	3.398160	0.763390	3.592320
O	-0.173381	-2.450950	-2.072830
O	2.384229	-2.045380	-1.788350
H	2.983179	-1.627090	-2.422340
C	1.502549	0.215630	-2.054310
H	2.541870	0.551120	-2.149890
H	1.159469	-0.119290	-3.039680
C	0.634340	1.328080	-1.539220
C	1.114870	2.263600	-0.615140
C	-0.702830	1.433910	-1.938740
C	0.289770	3.249540	-0.087840
H	2.153210	2.216210	-0.292280
C	-1.541280	2.413921	-1.421550
H	-1.098680	0.731830	-2.670350
C	-1.057420	3.335550	-0.477650
H	0.685310	3.961010	0.631430
H	-2.576580	2.472031	-1.746750
N	-1.903430	4.274811	0.094940
H	-2.724630	4.514211	-0.444520
H	-1.452370	5.089910	0.488360
O	-1.109491	-0.204950	1.821850
C	-3.067061	-1.373319	-1.067240
H	-2.838021	-2.104729	-1.846020
H	-4.112211	-1.507749	-0.771020
H	-2.952111	-0.369149	-1.483680
C	-2.288891	-3.011479	0.682400
H	-3.331031	-3.236119	0.927560
H	-1.957211	-3.718159	-0.083250
H	-1.677491	-3.164749	1.576830
C	-3.398761	-0.830729	2.333730
H	-3.504001	0.021681	3.007380
H	-4.379691	-1.048239	1.901940
H	-3.063911	-1.699579	2.903770
H	-2.715370	0.440321	0.728820
H	0.621209	-2.699840	-2.586070

Structure 18_{OH+}_ts

C	2.933918	-1.695654	1.920579
C	2.032827	-2.335683	1.087359
C	0.821058	-1.707011	0.740819
C	0.564830	-0.403101	1.241759
C	1.497720	0.239778	2.078999
C	2.670330	-0.405433	2.419349
C	-0.105232	-2.287410	-0.193131
C	-0.670180	0.201280	0.857269
C	-1.630650	-0.415679	0.082349
C	-1.376652	-1.699829	-0.398411
C	-2.869539	0.457423	0.054429
C	-2.224958	1.797602	0.511129
H	3.861478	-2.193005	2.185009
H	2.261746	-3.319223	0.689159
H	1.280052	1.239068	2.442629
H	3.393860	0.082786	3.063239
O	-2.304653	-2.344728	-1.113261
O	0.033006	-3.589790	-0.633451
H	0.030975	-4.204160	0.116029
C	0.693609	-1.343701	-2.092671
C	1.281270	-0.113252	-1.792551
C	2.618730	-0.047833	-1.294281
C	0.540901	1.104519	-1.862231
C	3.162621	1.129466	-0.863431
H	3.207779	-0.959644	-1.254151
C	1.073603	2.293668	-1.435081
H	-0.465199	1.079330	-2.271741

C	2.392213	2.330277	-0.903191
H	4.177791	1.165015	-0.480981
H	0.501084	3.213909	-1.494261
N	2.913374	3.483066	-0.458961
O	-1.025298	1.424741	1.264349
C	-3.518439	0.611374	-1.322031
H	-3.968570	-0.331706	-1.640651
H	-4.307568	1.369665	-1.279261
H	-2.790489	0.918503	-2.078531
C	-3.891430	-0.090026	1.063109
H	-4.781819	0.545555	1.097829
H	-4.199421	-1.094946	0.760059
H	-3.465530	-0.151226	2.069309
C	-3.062177	2.728623	1.353799
H	-2.499616	3.636792	1.580619
H	-3.962116	3.013254	0.801069
H	-3.359947	2.253053	2.290859
H	-1.854107	2.321852	-0.380311
H	-1.976154	-3.237418	-1.319151
H	3.851374	3.526685	-0.089401
H	2.383785	4.341717	-0.488231
H	1.308248	-2.238352	-2.143601
H	-0.251931	-1.377140	-2.627531

Structure 18_{NH-}

C	3.985930	-0.295507	1.551339
C	3.490120	-0.529428	0.267929
C	2.120680	-0.675868	0.058889
C	1.253000	-0.587449	1.159259
C	1.745430	-0.342649	2.444289
C	3.115300	-0.193258	2.638529
C	1.570040	-0.802679	-1.343241
C	-0.162660	-0.803900	0.898989
C	-0.700869	-1.189880	-0.287441
C	0.147711	-1.380610	-1.424561
C	-2.163049	-1.528121	-0.074521
C	-2.380740	-0.782421	1.271279
H	5.055360	-0.187927	1.702549
H	4.167350	-0.597277	-0.577951
H	1.053120	-0.273929	3.277209
H	3.504870	-0.002868	3.633059
O	-0.192069	-1.915200	-2.484621
O	2.435911	-1.614438	-2.114541
H	1.895891	-1.948769	-2.849571
C	1.522160	0.625961	-2.001621
H	2.567339	0.957802	-2.022461
H	1.207500	0.454581	-3.039301
C	0.632359	1.641561	-1.342251
C	1.046148	2.386231	-0.223861
C	-0.692671	1.831480	-1.761181
C	0.195218	3.252110	0.445419
H	2.069068	2.270441	0.137449
C	-1.562922	2.693029	-1.109151
H	-1.057071	1.268639	-2.622371
C	-1.171242	3.450639	0.048389
H	0.558857	3.811311	1.305269
H	-2.583302	2.802598	-1.475561
N	-1.967703	4.288219	0.731779
H	-2.889323	4.288188	0.291019
O	-1.054690	-0.703151	1.892569
C	-3.100499	-0.979092	-1.150801
H	-2.910749	-1.476002	-2.105991
H	-4.144489	-1.160583	-0.870951
H	-2.953630	0.096748	-1.282321
C	-2.327058	-3.050671	0.047979
H	-3.371138	-3.319042	0.241179
H	-2.018298	-3.524271	-0.888401
H	-1.708108	-3.454101	0.855889
C	-3.350229	-1.380172	2.261879
H	-3.421250	-0.747912	3.149799
H	-4.343589	-1.444373	1.807949
H	-3.037869	-2.382132	2.565249
H	-2.652790	0.257818	1.045299

Structure 18_{NH-}_ts

C	2.903662	-2.112307	1.803410
C	2.036962	-2.595648	0.839100
C	0.853522	-1.893159	0.523740
C	0.585911	-0.675849	1.210410
C	1.482440	-0.195898	2.183970
C	2.631971	-0.904617	2.479570
C	-0.014898	-2.282050	-0.543870
C	-0.642850	-0.022470	0.880860
C	-1.578300	-0.510681	0.010740
C	-1.341119	-1.724341	-0.705330
C	-2.818670	0.356148	0.099160
C	-2.187112	1.612968	0.758340
H	3.805572	-2.668766	2.041140
H	2.255453	-3.520208	0.313430
H	1.258029	0.737731	2.691600
H	3.324370	-0.535157	3.229140
O	-2.154048	-2.293292	-1.497190
O	0.167273	-3.514020	-1.127240
H	-0.654957	-3.659500	-1.633030
C	0.879581	-1.005369	-2.089870
C	1.330530	0.249131	-1.674050
C	2.625180	0.418533	-1.075430
C	0.516779	1.426351	-1.770650
C	3.071538	1.627483	-0.635810
H	3.258800	-0.461247	-0.971590
C	0.942637	2.646181	-1.334980
H	-0.471821	1.326850	-2.215500
C	2.255977	2.829852	-0.730840
H	4.057888	1.729124	-0.190570
H	0.298887	3.518780	-1.427970
N	2.723856	3.973003	-0.291300
O	-1.018731	1.135319	1.485010
C	-3.451611	0.711067	-1.246640
H	-3.867010	-0.184003	-1.715690
H	-4.260101	1.438216	-1.106720
H	-2.715861	1.145718	-1.930000
C	-3.858490	-0.332973	0.996910
H	-4.759240	0.281446	1.105310
H	-4.146049	-1.288454	0.547890
H	-3.450970	-0.532223	1.993440
C	-3.051662	2.423577	1.695680
H	-2.497713	3.288918	2.067780
H	-3.935393	2.787787	1.162910
H	-3.379232	1.823347	2.548070
H	-1.795772	2.252549	-0.045810
H	1.597362	-1.815398	-2.191330
H	-0.000439	-1.078610	-2.725980
H	2.023386	4.702462	-0.438560

Structure 18_{Zw1}

C	3.896640	0.107715	1.654280
C	3.453791	-0.591145	0.530960
C	2.089471	-0.721747	0.294570
C	1.179380	-0.136239	1.192580
C	1.622579	0.580212	2.311420
C	2.986919	0.700424	2.537500
C	1.555482	-1.285818	-0.989960
C	-0.217229	-0.363881	0.915480
C	-0.707508	-1.313011	0.004120

C	0.186293	-1.879140	-0.857460
C	-2.181648	-1.526304	0.289650
C	-2.472140	-0.217054	1.075380
H	4.961800	0.195367	1.841960
H	4.167022	-1.034534	-0.156390
H	0.899328	1.027931	2.985030
H	3.347858	1.249265	3.400320
O	-0.156066	-2.826991	-1.710680
O	2.385733	-2.262057	-1.574770
H	2.908823	-1.878066	-2.292310
C	1.413410	-0.038238	-2.028700
H	2.458970	0.248523	-2.196210
H	1.021701	-0.488519	-2.948310
C	0.602358	1.125441	-1.595860
C	1.150197	2.181271	-0.835120
C	-0.764632	1.240348	-1.939750
C	0.393875	3.254740	-0.414990
H	2.204547	2.137743	-0.558390
C	-1.541673	2.303277	-1.536920
H	-1.213001	0.457448	-2.551870
C	-1.012645	3.377338	-0.729410
H	0.858804	4.043401	0.173680
H	-2.590693	2.352316	-1.820850
N	-1.799356	4.374447	-0.341940
H	-1.249207	5.030088	0.215760
O	-1.159010	0.206378	1.617480
C	-3.067578	-1.605495	-0.954600
H	-2.832306	-2.500685	-1.535370
H	-4.119868	-1.659566	-0.656930
H	-2.934439	-0.727835	-1.592400
C	-2.349256	-2.790564	1.146550
H	-3.399176	-2.940615	1.415500
H	-2.013865	-3.661133	0.575710
H	-1.756516	-2.736363	2.064870
C	-3.459140	-0.276825	2.213520
H	-3.575481	0.711964	2.661610
H	-4.432459	-0.593347	1.827800
H	-3.139199	-0.982605	2.982960
H	-2.736291	0.573966	0.365780
H	0.652205	-3.136349	-2.164010

Structure 18_{Zw1}_ts

C	3.902410	0.153804	1.607121
C	3.456282	-0.643778	0.564381
C	2.080143	-0.798423	0.340891
C	1.170551	-0.122376	1.184161
C	1.632968	0.696046	2.232641
C	2.992167	0.831701	2.439911
C	1.550685	-1.484935	-0.835779
C	-0.218749	-0.349751	0.928321
C	-0.711245	-1.304013	0.044441
C	0.186947	-1.951159	-0.778609
C	-2.195145	-1.479598	0.305411
C	-2.469509	-0.154629	1.072331
H	4.968359	0.257868	1.782361
H	4.165834	-1.155305	-0.078279
H	0.914086	1.211173	2.861501
H	3.360395	1.458182	3.245191
O	-0.212290	-2.892201	-1.636739
O	2.377339	-2.389862	-1.497229
H	2.900607	-1.951450	-2.183489
C	1.389520	-0.010725	-2.078579
H	2.467810	0.151079	-2.132309
H	0.993592	-0.587237	-2.916289
C	0.618796	1.100962	-1.647539
C	1.185512	2.159454	-0.871039
C	-0.768844	1.222517	-1.979159
C	0.448029	3.228872	-0.452369
H	2.240163	2.095978	-0.602079
C	-1.523738	2.285575	-1.579679
H	-1.218221	0.438836	-2.587649
C	-0.968002	3.359217	-0.770979
H	0.912036	4.016193	0.137621
H	-2.574238	2.355091	-1.850719
N	-1.734675	4.351974	-0.389249
H	-1.183818	5.009386	0.166261
O	-1.170461	0.258426	1.626351
C	-3.071704	-1.561101	-0.945249
H	-2.848271	-2.468040	-1.512339
H	-4.127694	-1.590955	-0.655979
H	-2.918687	-0.695240	-1.594469
C	-2.397970	-2.731339	1.174101
H	-3.454490	-2.862322	1.428011
H	-2.066647	-3.614517	0.620001
H	-1.819530	-2.673517	2.101371
C	-3.480899	-0.188162	2.192141
H	-3.591333	0.807417	2.627331
H	-4.452748	-0.496896	1.796081
H	-3.182487	-0.887201	2.976611
H	-2.725572	0.622530	0.343881
H	0.577652	-3.264728	-2.068159

Structure 7_{O-}

C	-3.351357	-2.174806	0.678179
C	-3.097006	-1.137077	-0.214220
C	-2.036679	-0.249659	-0.014374
C	-1.216623	-0.405278	1.117548
C	-1.494147	-1.440196	2.025862
C	-2.543970	-2.325695	1.807677
C	-1.798938	0.813852	-1.100440
C	-0.101692	0.543662	1.348339
C	-0.029336	1.727721	0.592012
C	-1.003483	1.992849	-0.511695
C	0.977919	2.667328	0.833601
H	0.995622	3.569006	0.229286
C	1.947658	2.434932	1.800369
C	1.903016	1.247092	2.535651
C	0.889631	0.319184	2.316668
H	-4.180070	-2.855165	0.504384
H	-3.720835	-0.977045	-1.087960
H	-0.895835	-1.553816	2.923912
H	-2.738750	-3.119153	2.522612
H	2.738364	3.158068	1.972345
H	2.662922	1.041758	3.283493
H	0.888000	-0.595624	2.898977
O	-1.090362	3.119337	-0.981720
O	-2.916246	1.180552	-1.749213
C	-0.779797	0.153972	-2.159086
H	-1.401823	-0.617292	-2.626284
H	-0.606733	0.941456	-2.902015
C	0.526268	-0.436645	-1.702449
C	0.601257	-1.743962	-1.206043
C	1.714759	0.302718	-1.735093
C	1.798392	-2.285859	-0.744855
H	-0.302034	-2.350389	-1.171715
C	2.920610	-0.222652	-1.279739
H	1.695454	1.321177	-2.119444
C	2.978111	-1.528257	-0.770200
H	1.823755	-3.302950	-0.361459
H	3.825146	0.379541	-1.314156
N	4.171238	-2.040539	-0.246264
H	5.010070	-1.623444	-0.629751
H	4.233384	-3.050798	-0.257197

Structure 7_{O^-}-ts$_{iso}$

C	-2.686284	-2.875870	0.574738
C	-2.894436	-1.689924	-0.112790
C	-2.087149	-0.566405	0.120689
C	-1.049621	-0.630335	1.072464
C	-0.864282	-1.837726	1.772432
C	-1.662892	-2.945112	1.528546
C	-2.378458	0.669143	-0.664046
C	-0.191729	0.547566	1.295322
C	-0.397023	1.711207	0.524116
C	-1.452229	1.809147	-0.528091
C	0.416818	2.831956	0.732957
H	0.230610	3.709622	0.122841
C	1.425616	2.821983	1.686890
C	1.630734	1.673803	2.458504
C	0.829203	0.556311	2.262596
H	-3.310959	-3.741705	0.377674
H	-3.683833	-1.601901	-0.852262
H	-0.073745	-1.925591	2.509135
H	-1.489434	-3.864769	2.078858
H	2.047924	3.699534	1.834887
H	2.411610	1.650981	3.212465
H	1.006881	-0.318548	2.878138
O	-1.707026	2.933637	-1.053219
O	-3.416801	0.738610	-1.357779
C	-0.562464	0.526369	-2.036545
H	-1.288488	-0.148253	-2.486394
H	-0.487645	1.473533	-2.566044
C	0.701759	-0.091778	-1.657507
C	0.791646	-1.457279	-1.330279
C	1.881836	0.669404	-1.538715
C	1.985562	-2.033499	-0.910286
H	-0.101393	-2.076109	-1.396754
C	3.079999	0.104660	-1.124847
H	1.849638	1.730030	-1.777484
C	3.149494	-1.259602	-0.794706
H	2.021086	-3.092219	-0.664960
H	3.974689	0.717844	-1.049752
N	4.367228	-1.843783	-0.431978
H	5.035036	-1.203292	-0.021750
H	4.281432	-2.684765	0.124570

Structure 7_{Zw2}

C	-3.299398	-2.302159	0.558373
C	-3.131989	-1.138266	-0.188144
C	-2.059777	-0.275608	0.047031
C	-1.136109	-0.588467	1.060920
C	-1.324010	-1.751189	1.824275
C	-2.390139	-2.608766	1.572706
C	-1.914355	0.948190	-0.875947
C	0.000426	0.327096	1.318154
C	-0.033231	1.633057	0.795198
C	-1.126451	2.048330	-0.137084
C	0.994586	2.538562	1.073268
H	0.929993	3.538066	0.653967
C	2.091899	2.150156	1.832288
C	2.155022	0.842029	2.319457
C	1.119660	-0.054638	2.071813
H	-4.139888	-2.961256	0.361362
H	-3.833904	-0.860561	-0.967849
H	-0.642032	-1.986745	2.635094
H	-2.517442	-3.503453	2.174234
H	2.899316	2.848100	2.028881
H	3.017056	0.517437	2.894519
H	1.199817	-1.066444	2.455210
O	-1.318746	3.233459	-0.365194
O	-3.062490	1.371137	-1.413093
C	-0.927406	0.491443	-2.080785
H	-1.560568	-0.207985	-2.634518
H	-0.792916	1.391831	-2.689302
C	0.395792	-0.141961	-1.766433
C	0.480164	-1.509705	-1.468572
C	1.565082	0.625158	-1.698190
C	1.678892	-2.088809	-1.064601
H	-0.413546	-2.125076	-1.528412
C	2.774958	0.068287	-1.294844
H	1.524197	1.684866	-1.935705
C	2.802546	-1.279748	-0.970829
H	1.730142	-3.145168	-0.818581
H	3.669706	0.679642	-1.225602
N	4.070561	-1.878694	-0.510518
H	4.798856	-1.167178	-0.396835
H	3.961041	-2.343944	0.396682
H	4.429155	-2.575990	-1.171527

Structure 7_{Zw2}-ts$_{iso}$

C	1.658215	-2.544640	1.739501
C	0.566461	-2.703574	0.894858
C	-0.316237	-1.646750	0.646628
C	-0.109088	-0.399634	1.273346
C	1.000630	-0.255307	2.122201
C	1.877480	-1.308737	2.352643
C	-1.431260	-1.888915	-0.320531
C	-1.046006	0.711666	1.013849
C	-2.142285	0.518583	0.149862
C	-2.432489	-0.800786	-0.484157
C	-3.019160	1.578067	-0.124939
H	-3.852654	1.387769	-0.793236
C	-2.824266	2.829835	0.438127
C	-1.744918	3.027904	1.308024
C	-0.876265	1.984322	1.590897
H	2.336662	-3.373300	1.918571
H	0.374323	-3.649000	0.397971
H	1.193793	0.691093	2.614688
H	2.728121	-1.165838	3.011934
H	-3.502504	3.646289	0.211066
H	-1.581993	3.999857	1.763352
H	-0.046292	2.173825	2.261795
O	-3.500856	-0.987171	-1.086176
O	-1.684678	-3.062924	-0.703427
C	-0.641009	-0.771340	-2.018008
H	-0.555232	-1.761887	-2.456034
H	-1.410007	-0.157050	-2.480950
C	0.603367	-0.093364	-1.716756
C	1.798820	-0.822572	-1.526229
C	0.644757	1.297796	-1.489618
C	2.965176	-0.207016	-1.099236
H	1.797387	-1.894467	-1.699780
C	1.807524	1.929804	-1.064256
H	-0.259131	1.885854	-1.626130
C	2.947127	1.163749	-0.862829
H	3.870842	-0.786993	-0.946464
H	1.816971	3.001242	-0.885616
N	4.189959	1.829722	-0.426019
H	4.760081	2.140955	-1.220100
H	3.992435	2.654973	0.147971
H	4.772307	1.204854	0.139660

Structure 7_{2+}

C	-3.187748	-2.433841	0.542830
C	-3.053930	-1.266290	-0.205658
C	-2.018756	-0.378754	0.074443
C	-1.092822	-0.656856	1.090598

C	-1.245773	-1.829513	1.842348
C	-2.284541	-2.712140	1.568871
C	-1.835651	0.840907	-0.797784
C	0.012516	0.287008	1.340743
C	-0.055161	1.618910	0.838396
C	-1.104923	1.928213	-0.064676
C	0.960127	2.561305	1.123808
H	0.925836	3.565232	0.711197
C	2.059069	2.183604	1.865268
C	2.149397	0.862605	2.328473
C	1.144615	-0.066445	2.078590
H	-4.001647	-3.119058	0.331294
H	-3.754369	-1.035087	-1.000525
H	-0.564805	-2.051413	2.656093
H	-2.392664	-3.613755	2.161937
H	2.855563	2.890620	2.063038
H	3.024943	0.553896	2.890512
H	1.261426	-1.078101	2.448121
O	-1.431973	3.113545	-0.443810
O	-3.083321	1.273091	-1.253004
H	-2.979100	1.967823	-1.919258
C	-0.928927	0.468522	-2.056061
H	-1.562542	-0.230643	-2.609485
H	-0.814871	1.378633	-2.653997
C	0.411515	-0.143647	-1.753258
C	0.517318	-1.510998	-1.474672
C	1.560822	0.651782	-1.693838
C	1.734289	-2.070264	-1.096822
H	-0.362306	-2.145740	-1.535169
C	2.786534	0.111520	-1.315873
H	1.504151	1.710276	-1.933416
C	2.843247	-1.240805	-1.012593
H	1.806127	-3.128498	-0.867832
H	3.670758	0.738014	-1.257004
N	4.124733	-1.812383	-0.560441
H	4.923217	-1.271912	-0.908627
H	4.248926	-2.776862	-0.885593
H	-0.955206	3.836317	0.003172
H	4.188483	-1.825082	0.464339

Structure 7_{2+}-ts$_{iso}$

C	2.405515	3.066884	0.669473
C	2.720081	1.896850	0.005015
C	1.962208	0.732468	0.223077
C	0.861135	0.738419	1.113250
C	0.572896	1.941128	1.781246
C	1.327310	3.081836	1.564106
C	2.285313	-0.494756	-0.473081
C	0.054952	-0.476921	1.311317
C	0.393594	-1.667622	0.633647
C	1.497003	-1.655403	-0.311576
C	-0.349710	-2.842175	0.807784
H	-0.060936	-3.738872	0.270520
C	-1.449296	-2.841999	1.651266
C	-1.808405	-1.666013	2.319390
C	-1.066869	-0.504855	2.156499
H	2.989618	3.963297	0.496242
H	3.552324	1.906858	-0.692106
H	-0.255438	1.994032	2.476167
H	1.076276	3.995632	2.091889
H	-2.029817	-3.747732	1.787243
H	-2.673526	-1.657711	2.973894
H	-1.375373	0.384925	2.691103
O	1.940251	-2.839797	-0.743072
O	3.410164	-0.696857	-1.148666
H	3.942881	0.100365	-1.284149
C	0.735677	-0.690205	-2.005452
H	1.462184	-0.061599	-2.515631
H	0.700965	-1.698681	-2.412585
C	-0.547478	-0.068061	-1.695580
C	-0.648218	1.322680	-1.537035
C	-1.688017	-0.867435	-1.506641
C	-1.853141	1.906216	-1.161291
H	0.220440	1.953481	-1.701786
C	-2.897173	-0.297811	-1.133768
H	-1.625957	-1.941403	-1.650089
C	-2.948759	1.079794	-0.956718
H	-1.927276	2.980224	-1.028087
H	-3.775654	-0.916424	-0.982361
N	-4.218970	1.690169	-0.525171
H	-5.017074	1.073009	-0.707407
H	-4.403897	2.573456	-1.012918
H	2.792989	-2.750764	-1.201055
H	-4.214957	1.891834	0.481658

Structure 10_{O-}

C	-3.542660	-0.659674	-2.219331
C	-3.431699	-0.203138	-0.908657
C	-2.260605	-0.393277	-0.170615
C	-1.196555	-1.078453	-0.773140
C	-1.303951	-1.542328	-2.093617
C	-2.471203	-1.329312	-2.817919
C	-2.183578	0.213167	1.235013
C	0.026411	-1.319776	0.007850
C	0.147848	-0.991893	1.322065
C	-0.950188	-0.309750	2.010933
C	1.385518	-1.336394	2.102775
H	1.096522	-1.719690	3.085641
C	2.216742	-2.368891	1.347042
C	2.330816	-2.008019	-0.133552
H	-4.461294	-0.497114	-2.775788
H	-4.256761	0.309635	-0.423945
H	-0.467982	-2.067858	-2.542015
H	-2.549524	-1.687720	-3.839611
H	3.222145	-2.457049	1.770473
O	-0.852735	-0.080165	3.218712
O	-3.343256	0.093859	1.919224
C	-1.841023	1.758290	1.026983
H	-2.695263	2.156632	0.467826
H	-1.871560	2.172837	2.040361
C	-0.543535	2.125945	0.357605
C	-0.406130	2.134937	-1.035992
C	0.590999	2.448161	1.113434
C	0.805726	2.444370	-1.650193
H	-1.266888	1.891234	-1.656230
C	1.807007	2.766767	0.515168
H	0.519501	2.448455	2.199414
C	1.932143	2.767767	-0.881143
H	0.881994	2.441441	-2.734840
H	2.669753	3.011670	1.130070
N	3.168859	3.017880	-1.488843
H	3.795704	3.591841	-0.938963
H	3.105434	3.363092	-2.438351
O	0.996077	-1.930496	-0.702981
H	1.739615	-3.353799	1.419884
H	1.969899	-0.424619	2.293272
C	3.024662	-3.106882	-0.921928
H	3.024067	-2.867078	-1.989069
H	4.061244	-3.204321	-0.587520
H	2.516734	-4.064111	-0.774398
C	3.005350	-0.656906	-0.353683
H	3.095295	-0.454530	-1.425074
H	2.428842	0.154945	0.099477
H	4.007249	-0.662907	0.087054

Structure $10_{O\text{-}}$_ts$_{iso}$

C	3.652903	-0.489311	-2.388288
C	3.579361	-0.738433	-1.023528
C	2.549265	-0.190783	-0.249380
C	1.589018	0.640772	-0.858014
C	1.678290	0.899104	-2.236817
C	2.696637	0.335949	-2.994669
C	2.510737	-0.468039	1.216541
C	0.520363	1.207740	-0.042508
C	0.356015	0.868316	1.255991
C	1.270550	-0.095955	1.942772
C	-0.744763	1.481088	2.072077
H	-0.359266	1.712022	3.068732
C	-1.282365	2.734664	1.386997
C	-1.510749	2.496436	-0.106911
H	4.447274	-0.930554	-2.982290
H	4.313252	-1.367232	-0.528778
H	0.935714	1.538786	-2.700738
H	2.750230	0.537297	-4.060296
H	-2.220536	3.064740	1.845121
O	1.215163	-0.159570	3.223180
O	3.482096	-1.005269	1.778683
C	0.770649	-1.940856	1.220296
H	1.556208	-2.443451	0.657251
H	0.747306	-2.272223	2.256376
C	-0.522110	-1.961235	0.529794
C	-0.605247	-1.939588	-0.874135
C	-1.735822	-2.009769	1.239284
C	-1.828678	-1.975946	-1.535229
H	0.313533	-1.906247	-1.456644
C	-2.963567	-2.055538	0.588257
H	-1.710104	-2.033424	2.326685
C	-3.029213	-2.037472	-0.813279
H	-1.859371	-1.958038	-2.622002
H	-3.883774	-2.102288	1.165548
N	-4.264908	-2.008013	-1.471489
H	-5.026195	-2.423889	-0.949577
H	-4.242307	-2.363695	-2.419053
O	-0.258809	2.112094	-0.717534
H	-0.558170	3.552163	1.492369
H	-1.546960	0.742417	2.220731
C	-1.917617	3.779821	-0.813654
H	-1.994864	3.611009	-1.891852
H	-2.890649	4.116509	-0.444268
H	-1.181536	4.568497	-0.632405
C	-2.520909	1.380600	-0.357383
H	-2.717023	1.283383	-1.429853
H	-2.137824	0.424537	0.007416
H	-3.464668	1.598348	0.154035

Structure $10_{O\text{-}}$_iso

C	-2.282859	0.349649	3.028907
C	-1.930097	1.367174	2.148449
C	-0.818845	1.230923	1.313807
C	-0.023905	0.073135	1.378222
C	-0.374284	-0.942070	2.279771
C	-1.499717	-0.806886	3.088779
C	-0.476715	2.321563	0.352995
C	1.157319	-0.039202	0.513287
C	1.426010	0.844323	-0.463924
C	0.520565	2.036852	-0.791644
C	2.617239	0.648473	-1.358755
H	3.381858	1.398863	-1.122055
C	3.176314	-0.765940	-1.224348
C	3.247382	-1.193070	0.242944
H	-3.158707	0.450875	3.661756
H	-2.520693	2.275406	2.077353
H	0.240021	-1.833361	2.338056
H	-1.765900	-1.606260	3.773923
H	2.525959	-1.478291	-1.747547
C	-0.453859	1.563477	-1.978017
H	-1.060612	2.448337	-2.202045
H	0.218358	1.394741	-2.826485
C	-1.325624	0.360335	-1.745116
C	-2.632193	0.483849	-1.258158
C	-0.837897	-0.937313	-1.940808
C	-3.411093	-0.629226	-0.953558
H	-3.045852	1.477719	-1.094924
C	-1.601971	-2.062409	-1.642509
H	0.169846	-1.070482	-2.329206
C	-2.901769	-1.923019	-1.134635
H	-4.419533	-0.499605	-0.568372
H	-1.193076	-3.057173	-1.802080
N	-3.651258	-3.046530	-0.763518
H	-3.401611	-3.895965	-1.254139
H	-4.653199	-2.903434	-0.779015
O	1.920134	-1.147580	0.805575
H	4.173018	-0.842481	-1.671536
H	2.327108	0.848168	-2.396855
C	4.156376	-0.277732	1.063713
H	4.222864	-0.644109	2.092219
H	5.162157	-0.260987	0.632541
H	3.771625	0.745382	1.084825
C	3.682452	-2.644575	0.373887
H	3.648760	-2.957249	1.421773
H	3.023177	-3.294747	-0.208672
H	4.706235	-2.763722	0.007783
O	-1.045137	3.402752	0.440356
O	1.216257	3.125358	-1.183734

Structure $18_{O\text{-}}$

C	3.997650	-0.484831	1.464054
C	3.474346	-0.656754	0.183430
C	2.099261	-0.781313	-0.025146
C	1.262216	-0.750041	1.102976
C	1.778278	-0.582757	2.394667
C	3.149552	-0.442415	2.575655
C	1.581838	-0.873648	-1.475284
C	-0.158689	-0.927077	0.860029
C	-0.726160	-1.178293	-0.342247
C	0.080004	-1.288343	-1.542593
C	-2.199033	-1.479403	-0.129277
C	-2.364579	-0.868965	1.289319
H	5.070733	-0.385060	1.599982
H	4.127167	-0.701066	-0.683037
H	1.099536	-0.563358	3.242004
H	3.557863	-0.308317	3.572275
O	-0.416999	-1.637487	-2.616212
O	2.377727	-1.627961	-2.261757
C	1.563130	0.632904	-2.013167
H	2.616195	0.935790	-2.004993
H	1.241953	0.540465	-3.056702
C	0.708900	1.638495	-1.288327
C	1.152368	2.278476	-0.123967
C	-0.590756	1.928385	-1.719787
C	0.333149	3.148379	0.591451
H	2.159318	2.082417	0.240068
C	-1.421816	2.798155	-1.018941
H	-0.968221	1.448632	-2.621171
C	-0.972202	3.417489	0.155605
H	0.704479	3.626071	1.494835
H	-2.428574	2.998602	-1.377831
N	-1.824802	4.237508	0.905770

H	-2.563115	4.673888	0.367983
H	-1.347553	4.919844	1.481369
O	-1.032881	-0.899388	1.887930
C	-3.141227	-0.791755	-1.118159
H	-3.002604	-1.204297	-2.120280
H	-4.184440	-0.947820	-0.818682
H	-2.946989	0.284241	-1.157264
C	-2.422792	-2.999172	-0.157779
H	-3.473863	-3.248091	0.025939
H	-2.144709	-3.385583	-1.142432
H	-1.808870	-3.505167	0.594543
C	-3.343748	-1.527418	2.232212
H	-3.377798	-0.987124	3.181275
H	-4.345970	-1.510342	1.793448
H	-3.064980	-2.565632	2.427761
H	-2.606872	0.197446	1.172494

Structure 18$_{O-}$_ts$_{iso}$

C	-2.822273	1.648179	2.006288
C	-1.915824	2.294287	1.174554
C	-0.722910	1.672215	0.787741
C	-0.440252	0.378936	1.287026
C	-1.356842	-0.269934	2.128281
C	-2.543523	0.358760	2.480070
C	0.205282	2.387562	-0.133339
C	0.808495	-0.213969	0.883205
C	1.667752	0.358657	0.019082
C	1.411665	1.655856	-0.623681
C	2.910076	-0.506192	-0.072681
C	2.355487	-1.812428	0.568091
H	-1.127166	-1.267522	2.490603
H	-3.254952	-0.149054	3.124512
O	2.328160	2.255144	-1.273506
O	-0.012709	3.578747	-0.440406
C	-0.026562	1.023504	-2.051503
H	-0.372556	1.988883	-2.417749
H	0.844914	0.652137	-2.586405
C	-1.044243	0.029168	-1.753768
C	-2.385471	0.385826	-1.498552
C	-0.712255	-1.328462	-1.592153
C	-3.324419	-0.544630	-1.083991
H	-2.683732	1.425811	-1.612495
C	-1.646408	-2.272999	-1.176384
H	0.306890	-1.647108	-1.796392
C	-2.966011	-1.892401	-0.902359
H	-4.347051	-0.233826	-0.883563
H	-1.351685	-3.312857	-1.056340
N	-3.895307	-2.815712	-0.408242
H	-3.700313	-3.778325	-0.653360
H	-4.861038	-2.582072	-0.601826
O	1.235182	-1.412549	1.398984
H	-3.750075	2.138882	2.284572
H	-2.121714	3.289209	0.791871
C	3.395056	-0.773883	-1.498668
H	3.748259	0.154853	-1.952112
H	4.216536	-1.500567	-1.490638
H	2.590728	-1.176487	-2.123259
C	4.038336	0.139835	0.746016
H	4.948836	-0.469654	0.718384
H	4.262270	1.123058	0.322995
H	3.741302	0.273683	1.791447
C	3.311798	-2.647265	1.389321
H	2.809273	-3.544629	1.759032
H	4.157769	-2.960802	0.769760
H	3.694226	-2.082485	2.243194
H	1.929630	-2.425600	-0.240681

Structure 18$_{O-}$_iso

C	2.623755	-2.369839	1.790654
C	1.989030	-2.487397	0.555722
C	0.819271	-1.775533	0.289302
C	0.266359	-0.948550	1.287135
C	0.895243	-0.843568	2.532472
C	2.072470	-1.547888	2.777535
C	0.149852	-1.908327	-1.050644
C	-0.966088	-0.252216	0.959937
C	-1.552719	-0.249728	-0.241360
C	-0.983115	-0.924101	-1.477797
C	-2.888749	0.465656	-0.103111
C	-2.627665	1.226315	1.225547
H	3.542829	-2.913116	1.985450
H	2.400049	-3.120900	-0.224486
H	0.458614	-0.209850	3.297670
H	2.562441	-1.456993	3.742206
O	-1.917125	-1.496898	-2.264664
O	0.546616	-2.763420	-1.829567
C	-0.168661	0.181081	-2.303521
H	0.178450	-0.355000	-3.194344
H	-0.924457	0.907167	-2.618839
C	0.986504	0.865012	-1.621744
C	2.293550	0.377225	-1.744109
C	0.798514	1.979117	-0.794601
C	3.361030	0.952873	-1.060449
H	2.479183	-0.485459	-2.381665
C	1.853808	2.567645	-0.102352
H	-0.200058	2.395207	-0.684002
C	3.154014	2.056142	-0.220324
H	4.362958	0.546102	-1.173233
H	1.673873	3.430883	0.533677
N	4.208485	2.598700	0.524662
H	4.080995	3.568993	0.783257
H	5.124696	2.451241	0.120395
O	-1.643224	0.439688	1.941785
C	-3.232046	1.439987	-1.229997
H	-3.325065	0.895667	-2.173736
H	-4.183849	1.942505	-1.019759
H	-2.462241	2.209720	-1.347470
C	-4.004770	-0.584253	0.023318
H	-4.983631	-0.106051	0.147362
H	-4.006967	-1.192208	-0.884191
H	-3.828955	-1.241397	0.882081
C	-3.811619	1.488128	2.128653
H	-3.498453	2.052407	3.010857
H	-4.563403	2.078481	1.595427
H	-4.270497	0.552036	2.456418
H	-2.133861	2.178021	0.972257

Structure PAB-Cl

C	1.972081	0.000777	0.841289
H	2.275766	-0.890820	1.387703
H	2.275690	0.893342	1.386161
C	0.516261	0.000459	0.517734
C	-0.182147	-1.200606	0.353796
C	-0.182498	1.201204	0.353027
C	-1.535307	-1.208094	0.039462
H	0.341484	-2.146044	0.473847
C	-1.535673	1.208091	0.038703
H	0.340846	2.146877	0.472476
C	-2.234164	-0.000153	-0.123452
H	-2.062323	-2.150834	-0.077278
H	-2.062969	2.150600	-0.078626
N	-3.600360	-0.000438	-0.374145
H	-3.967761	0.838640	-0.802537
H	-3.967525	-0.839935	-0.801920

17	3.006941	-0.000519	-0.674363

Structure PAB-Cl-ts

C	-1.631203	-0.000006	1.238889
H	-2.130431	0.925186	1.500266
H	-2.130428	-0.925201	1.500260
C	-0.345615	-0.000002	0.761037
C	0.343718	1.231412	0.492194
C	0.343718	-1.231415	0.492188
C	1.616004	1.239627	0.010165
H	-0.171470	2.168671	0.681415
C	1.616004	-1.239629	0.010158
H	-0.171470	-2.168673	0.681406
C	2.288842	0.000000	-0.242364
H	2.135893	2.170396	-0.189618
H	2.135890	-2.170397	-0.189631
N	3.531802	0.000003	-0.709571
H	4.025008	-0.862996	-0.892189
H	4.025003	0.863007	-0.892183
17	-3.401730	0.000003	-0.812136

Structure PAB-OMs

C	0.741096	1.712246	0.428584
H	1.092018	1.698663	1.461793
H	0.741021	2.736191	0.053556
C	-0.594195	1.049841	0.284824
C	-1.119566	0.258837	1.310227
C	-1.316212	1.158470	-0.909878
C	-2.328115	-0.411197	1.152563
H	-0.568531	0.155176	2.241362
C	-2.519524	0.489491	-1.083257
H	-0.924277	1.768993	-1.720014
C	-3.045787	-0.308034	-0.050285
H	-2.720925	-1.023133	1.959453
H	-3.065294	0.581200	-2.018005
N	-4.220026	-1.021341	-0.239177
H	-4.841823	-0.667395	-0.953582
H	-4.712715	-1.292531	0.601028
O	1.766099	1.080975	-0.410828
S	2.261884	-0.400867	-0.041151
O	2.097606	-0.610579	1.392008
O	3.588905	-0.506606	-0.627843
C	1.157573	-1.465212	-0.935044
H	1.198471	-1.180041	-1.986051
H	1.526590	-2.481714	-0.791935
H	0.153004	-1.349117	-0.528059

Structure PAB-OMs-ts

C	0.369588	1.965738	-0.515516
H	0.566952	2.809716	0.134415
H	0.941153	1.894838	-1.433261
C	-0.764110	1.191063	-0.340381
C	-1.589846	1.346803	0.816581
C	-1.113908	0.180774	-1.290534
C	-2.689883	0.562748	1.011951
H	-1.326927	2.103835	1.549975
C	-2.215869	-0.605896	-1.111846
H	-0.474060	0.040607	-2.156063
C	-3.032663	-0.434690	0.047258
H	-3.314472	0.683184	1.890654
H	-2.482082	-1.367952	-1.836591
N	-4.109075	-1.201283	0.230157
H	-4.367806	-1.911267	-0.439875
H	-4.698257	-1.093271	1.043181
O	2.059030	1.082818	0.491004
S	2.341506	-0.340638	0.074333
O	3.722487	-0.736997	0.417937
O	1.968813	-0.573390	-1.338426
C	1.255292	-1.348669	1.067625
H	0.224812	-1.059793	0.852697
H	1.423941	-2.392612	0.802070
H	1.491931	-1.174758	2.117416

Structure PAB-OSO$_2$Ph

C	-0.872964	-2.265254	0.444605
H	-1.310705	-2.179109	1.441715
H	-0.941568	-3.298899	0.104326
C	0.523650	-1.731589	0.381440
C	1.011881	-0.868694	1.366141
C	1.335401	-2.007826	-0.723677
C	2.261464	-0.274450	1.244698
H	0.396489	-0.640057	2.233675
C	2.585595	-1.419620	-0.859572
H	0.975409	-2.680374	-1.498974
C	3.062402	-0.531426	0.120228
H	2.618154	0.407088	2.011987
H	3.200139	-1.636715	-1.728811
N	4.273132	0.126115	-0.051049
H	4.935323	-0.324511	-0.668373
H	4.709917	0.470873	0.793304
O	-1.752217	-1.565828	-0.505963
S	-2.417694	-0.171815	-0.085337
O	-2.875597	-0.253712	1.295266
O	-3.381189	0.084943	-1.144349
C	-1.094482	1.005668	-0.175135
C	-0.939021	1.912141	0.868716
C	-0.254607	0.995961	-1.287715
C	0.099243	2.839636	0.793086
H	-1.603370	1.878505	1.725439
C	0.785006	1.917957	-1.340991
H	-0.393088	0.264926	-2.078259
C	0.959358	2.837786	-0.303819
H	0.240199	3.553786	1.597207
H	1.463931	1.913413	-2.187202
H	1.774811	3.552342	-0.350982

Structure PAB-OSO$_2$Ph-ts

C	-0.629499	-2.553165	0.331931
H	-1.279711	-2.483469	1.197335
H	-0.835947	-3.341619	-0.382191
C	0.596496	-1.902407	0.323610
C	0.928099	-0.957887	1.342174
C	1.516004	-2.085933	-0.752191
C	2.090257	-0.241722	1.293050
H	0.226113	-0.802351	2.157526
C	2.681926	-1.376733	-0.816060
H	1.271669	-2.798559	-1.535142
C	2.988317	-0.421629	0.199766
H	2.332702	0.485393	2.061151
H	3.376575	-1.514763	-1.638049
N	4.108445	0.304038	0.124120
H	4.756342	0.192000	-0.641953
H	4.337639	0.987002	0.831496
O	-2.017028	-1.411120	-0.763372
S	-2.461404	-0.146177	-0.068766
O	-2.678356	-0.361503	1.375479
O	-3.596463	0.475149	-0.777472
C	-1.073989	0.974568	-0.201668
C	-0.891978	1.936893	0.790075
C	-0.203991	0.876456	-1.285241
C	0.188582	2.811613	0.696804
H	-1.573809	1.979978	1.633429
C	0.877770	1.751548	-1.366531
H	-0.355431	0.105313	-2.034044

C	1.074948	2.715754	-0.377105
H	0.345430	3.558539	1.468208
H	1.574752	1.669755	-2.194945
H	1.924156	3.389003	-0.440169

Structure PAB-OTf

C	0.233327	-2.129110	-0.513125
H	0.177865	-2.765086	-1.396196
H	0.502208	-2.724185	0.359964
C	-0.998105	-1.316133	-0.295249
C	-1.605971	-0.647019	-1.364750
C	-1.551386	-1.179517	0.980827
C	-2.724501	0.148070	-1.165913
H	-1.192804	-0.745842	-2.365908
C	-2.679140	-0.396390	1.191288
H	-1.088861	-1.687523	1.823030
C	-3.279997	0.285246	0.119961
H	-3.183276	0.665153	-2.003774
H	-3.098683	-0.301112	2.188620
N	-4.365050	1.119827	0.331749
H	-4.910618	0.938076	1.163193
H	-4.940308	1.315368	-0.476227
O	1.405947	-1.280017	-0.854970
S	2.078029	-0.378840	0.250458
O	3.463432	-0.208544	-0.122016
O	1.686525	-0.794515	1.579557
C	1.295641	1.283301	-0.066095
9	1.180877	1.478704	-1.372584
9	0.106668	1.368349	0.504528
9	2.096337	2.208694	0.449778

Structure PAB-OTf-ts

C	0.190889	-2.302330	0.333519
H	0.071778	-2.831807	1.271776
H	-0.263505	-2.754016	-0.541491
C	1.252596	-1.404955	0.171116
C	1.996179	-0.946227	1.293559
C	1.592292	-0.903216	-1.115940
C	3.019134	-0.048158	1.147197
H	1.741751	-1.318079	2.282331
C	2.618935	-0.011586	-1.279690
H	1.017123	-1.235331	-1.974851
C	3.354362	0.441808	-0.148138
H	3.585308	0.296421	2.006283
H	2.880674	0.361929	-2.264224
N	4.356762	1.319651	-0.299862
H	4.607886	1.674474	-1.210499
H	4.890311	1.647751	0.491212
O	-1.468465	-1.321101	0.798142
S	-2.106185	-0.454092	-0.253689
O	-3.559035	-0.386670	-0.133857
O	-1.542928	-0.657758	-1.586651
C	-1.535798	1.233566	0.236837
9	-1.967685	1.532969	1.461644
9	-0.205988	1.300563	0.226403
9	-2.014974	2.139512	-0.616303

Structure PAB-OAc

C	-1.094887	0.001111	-0.929775
H	-1.366726	-0.886566	-1.507214
H	-1.366845	0.890395	-1.504665
C	0.355884	0.000674	-0.557336
C	1.046977	-1.199368	-0.364891
C	1.046927	1.200241	-0.361889
C	2.384742	-1.207586	0.012998
H	0.530724	-2.144728	-0.513857
C	2.384692	1.207561	0.016073
H	0.530639	2.145953	-0.508494
C	3.074341	-0.000240	0.211045
H	2.906043	-2.150545	0.152344
H	2.905923	2.150190	0.157899
N	4.429095	-0.000615	0.527169
H	4.769393	0.836996	0.980580
H	4.769330	-0.839024	0.979152
O	-1.872986	-0.000712	0.292995
C	-3.204110	-0.000036	0.145267
O	-3.747540	0.001919	-0.940544
C	-3.910871	-0.002249	1.471275
H	-3.615120	0.878747	2.045323
H	-4.987402	-0.000422	1.311686
H	-3.617586	-0.886992	2.040844

Structure PAB-OAc-ts

C	1.146755	1.776653	-0.588459
H	1.451936	2.587457	0.062866
H	1.761486	1.564252	-1.454418
C	-0.041026	1.122013	-0.397934
C	-0.888047	1.422921	0.724683
C	-0.449201	0.072704	-1.292621
C	-2.051146	0.749069	0.931172
H	-0.582120	2.205693	1.412756
C	-1.611553	-0.608884	-1.097208
H	0.199548	-0.172603	-2.127024
C	-2.445183	-0.287035	0.020715
H	-2.692033	0.977869	1.776010
H	-1.920297	-1.398377	-1.773779
N	-3.582236	-0.944872	0.217550
H	-3.881691	-1.677617	-0.410911
H	-4.185398	-0.729724	0.999288
O	2.861014	0.526289	0.497706
C	2.446217	-0.636635	0.194037
O	2.425389	-1.115620	-0.963604
C	1.835996	-1.460113	1.324886
H	0.794755	-1.129585	1.440033
H	1.833730	-2.526157	1.092229
H	2.347637	-1.276613	2.271663

Structure PAB-OTFA

C	-0.001831	1.386742	-0.000471
H	0.190813	1.988286	0.890788
H	0.190869	1.987596	-0.892185
C	-1.370801	0.789481	-0.000279
C	-2.020303	0.489332	1.201236
C	-2.020067	0.488045	-1.201602
C	-3.279646	-0.096902	1.208738
H	-1.533928	0.718228	2.146263
C	-3.279406	-0.098196	-1.208724
H	-1.533505	0.715920	-2.146779
C	-3.928666	-0.401576	0.000106
H	-3.772228	-0.319039	2.151083
H	-3.771803	-0.321343	-2.150925
N	-5.212018	-0.929981	0.000257
H	-5.485035	-1.422902	-0.839288
H	-5.485208	-1.422000	0.840273
O	0.969186	0.289711	-0.000026
C	2.234837	0.641787	-0.000142
O	2.693518	1.753936	-0.000574
C	3.125676	-0.620635	0.000231
9	4.412666	-0.285613	0.000243
9	2.883955	-1.370368	-1.081749
9	2.883797	-1.369854	1.082530

Structure PAB-OTFA-ts

C	0.333266	2.387927	0.016407
H	0.178397	3.001563	-0.863153
H	-0.142222	2.699269	0.938453
C	1.264611	1.374253	0.016974
C	1.958735	1.008211	-1.182676
C	1.529268	0.622348	1.208792
C	2.852908	-0.019564	-1.196441
H	1.760286	1.567521	-2.092448
C	2.422944	-0.407295	1.210094
H	0.990811	0.884263	2.114139
C	3.104599	-0.758766	0.003291
H	3.380312	-0.293486	-2.104011
H	2.625212	-0.974291	2.112546
N	3.970972	-1.769588	-0.005820
H	4.158147	-2.307627	0.828435
H	4.459096	-2.036104	-0.849103
O	-1.626638	1.424833	-0.542162
C	-1.865965	0.581815	0.357991
O	-1.595114	0.600657	1.563276
C	-2.537405	-0.717505	-0.177960
9	-3.102924	-1.455399	0.787640
9	-1.609407	-1.495883	-0.777275
9	-3.488847	-0.469437	-1.093906

Structure PAB-OMoc

C	-0.598211	0.204021	1.030506
H	-0.838579	1.188607	1.439634
H	-0.844477	-0.559596	1.772569
C	0.830239	0.116343	0.591837
C	1.517227	1.253058	0.155428
C	1.501341	-1.110265	0.577688
C	2.833425	1.173496	-0.284015
H	1.015180	2.217535	0.160054
C	2.817035	-1.205015	0.140438
H	0.986773	-2.006822	0.914929
C	3.503509	-0.060908	-0.298989
H	3.353594	2.068354	-0.614075
H	3.324505	-2.165610	0.141079
N	4.838884	-0.137981	-0.676967
H	5.152324	-1.048537	-0.985886
H	5.163353	0.601973	-1.285141
O	-1.435977	-0.015529	-0.135429
C	-2.745090	0.019605	0.092908
O	-3.272203	0.215629	1.166800
O	-3.385037	-0.195232	-1.053558
C	-4.817393	-0.189626	-0.964602
H	-5.167352	0.780270	-0.608498
H	-5.170116	-0.375961	-1.976115
H	-5.154140	-0.977541	-0.289463

Structure PAB_OMoc-ts

C	1.161555	-2.061912	-0.255313
H	1.549607	-2.338904	-1.227782
H	1.646396	-2.485571	0.615840
C	-0.028113	-1.380044	-0.138752
C	-0.713363	-0.890819	-1.300172
C	-0.586532	-1.082277	1.148491
C	-1.873535	-0.185208	-1.193647
H	-0.286389	-1.092020	-2.278249
C	-1.748047	-0.379086	1.270626
H	-0.066247	-1.431370	2.035490
C	-2.422408	0.088639	0.099360
H	-2.390775	0.181357	-2.073949
H	-2.170981	-0.159108	2.244940
N	-3.554050	0.781001	0.211633
H	-3.958908	0.982723	1.114991
H	-4.045619	1.119824	-0.603283
O	2.861045	-0.553711	-0.212629
C	2.233282	0.460183	0.209839
O	2.112601	0.862444	1.376020
O	1.580060	1.138834	-0.797171
C	0.743431	2.215173	-0.394146
H	1.323392	3.005697	0.088878
H	0.279413	2.595265	-1.304930
H	-0.028799	1.866663	0.299157

Structure PAB-O(H^+)Moc

C	0.472029	0.928317	0.758650
H	0.831430	0.633353	1.742271
H	0.732434	1.951464	0.500861
C	-0.892550	0.510543	0.431078
C	-1.508805	-0.534841	1.139728
C	-1.610685	1.149528	-0.594478
C	-2.797209	-0.933336	0.836799
H	-0.969846	-1.031368	1.942392
C	-2.897543	0.757427	-0.908591
H	-1.150208	1.965360	-1.145253
C	-3.516033	-0.294043	-0.196428
H	-3.266594	-1.737019	1.395311
H	-3.443876	1.259138	-1.701065
N	-4.774712	-0.710156	-0.526111
H	-5.276293	-1.298611	0.122513
H	-5.351372	-0.110953	-1.098019
O	1.455077	0.129648	-0.214944
C	2.854531	0.153850	0.005353
O	3.323914	1.007584	0.684934
O	3.375142	-0.838856	-0.646987
C	4.821292	-0.961369	-0.549073
H	5.094497	-1.098234	0.496190
H	5.064064	-1.838917	-1.139676
H	5.280968	-0.065037	-0.962771
H	1.094566	-0.701537	-0.592240

Structure PAB-O(H^+)Moc-ts

C	0.434974	0.952450	0.781481
H	0.831875	0.624008	1.738902
H	0.714979	1.966315	0.511267
C	-0.907448	0.526135	0.449208
C	-1.504475	-0.556726	1.125506
C	-1.643837	1.186236	-0.555170
C	-2.783378	-0.967583	0.815511
H	-0.951406	-1.070265	1.907632
C	-2.922696	0.784953	-0.876096
H	-1.197806	2.026114	-1.081129
C	-3.519385	-0.304870	-0.196137
H	-3.237407	-1.798079	1.346408
H	-3.483156	1.303419	-1.647435
N	-4.769523	-0.716770	-0.517069
H	-5.335360	-0.192412	-1.165938
H	-5.233698	-1.424397	0.030792
O	1.473965	0.142559	-0.252856
C	2.857702	0.157943	-0.020100
O	3.336053	1.023495	0.642917
O	3.390843	-0.856382	-0.636590
C	4.833192	-0.973121	-0.514053
H	5.093814	-1.078045	0.538237
H	5.087598	-1.867289	-1.074421
H	5.299689	-0.089190	-0.946859
H	1.122759	-0.692679	-0.626632

Structure PAB-C$(NO_2)_3$

C	-0.363598	-0.122939	-1.258691
H	-0.585146	-1.052831	-1.788528

H	-0.598430	0.703380	-1.936052
C	1.077537	-0.072254	-0.812713
C	1.806843	-1.253546	-0.638912
C	1.728550	1.147004	-0.598543
C	3.137415	-1.224768	-0.245745
H	1.329475	-2.214452	-0.815303
C	3.059169	1.187920	-0.200520
H	1.197616	2.082172	-0.754571
C	3.784673	0.000417	-0.010688
H	3.686394	-2.153719	-0.121778
H	3.547611	2.145347	-0.044558
N	5.128061	0.037315	0.329488
H	5.445162	0.879671	0.789974
H	5.499085	-0.799929	0.757927
C	-1.395991	-0.023997	-0.138244
N	-1.273859	1.266647	0.669665
O	-1.319339	1.199572	1.872452
O	-1.176519	2.263650	-0.009863
N	-2.830022	0.004187	-0.686133
O	-3.645130	0.606917	-0.025547
O	-3.015901	-0.594198	-1.716317
N	-1.344565	-1.199480	0.825944
O	-2.301556	-1.943670	0.834643
O	-0.327385	-1.304440	1.465191

Structure PAB-C(NO_2)$_3$-ts

C	-0.106617	0.050532	1.676372
H	-0.561410	0.985453	1.992507
H	-0.581763	-0.867383	2.012756
C	1.139830	0.029981	1.103793
C	1.824577	1.251539	0.766623
C	1.795778	-1.217976	0.805327
C	3.060515	1.233282	0.202231
H	1.334952	2.197181	0.978516
C	3.033688	-1.248904	0.245822
H	1.282773	-2.145342	1.045784
C	3.701381	-0.020194	-0.071950
H	3.578749	2.153534	-0.045150
H	3.529140	-2.189208	0.029876
N	4.910690	-0.041834	-0.615383
H	5.384411	-0.912785	-0.813301
H	5.400100	0.812782	-0.843524
C	-1.656804	0.020091	-0.184997
N	-0.958531	-0.978079	-0.877852
O	-0.026319	-0.681293	-1.612188
O	-1.269882	-2.141923	-0.597211
N	-2.975496	-0.404336	0.317626
O	-3.780959	-0.822566	-0.490552
O	-3.169289	-0.292521	1.512355
N	-1.616722	1.380458	-0.536001
O	-2.601447	2.055359	-0.215766
O	-0.607183	1.843220	-1.051077

Structure PAB-C(CN)$_3$

C	0.982491	-0.000086	-1.210768
H	1.238874	0.886512	-1.795783
H	1.238813	-0.886836	-1.795590
C	-0.462411	-0.000028	-0.798994
C	-1.151598	1.201306	-0.596952
C	-1.151634	-1.201338	-0.596919
C	-2.480445	1.207708	-0.193179
H	-0.644694	2.148892	-0.762604
C	-2.480484	-1.207699	-0.193149
H	-0.644750	-2.148940	-0.762544
C	-3.165717	0.000017	0.021426
H	-2.998483	2.151131	-0.047455
H	-2.998557	-2.151101	-0.047414
N	-4.508478	0.000038	0.369477
H	-4.847682	-0.839829	0.818857
H	-4.847688	0.839968	0.818737
C	1.963924	-0.000002	0.028761
C	1.739133	1.204085	0.858706
N	1.545011	2.156566	1.479900
C	3.363610	-0.000282	-0.450688
N	4.437782	-0.000465	-0.871299
C	1.738764	-1.203773	0.859060
N	1.544310	-2.156032	1.480491

Structure PAB-C(CN)$_3$-ts

C	-0.765533	-0.003600	1.509198
H	-1.239409	0.922692	1.820140
H	-1.238344	-0.930896	1.818879
C	0.519774	-0.002434	1.010298
C	1.197669	1.228828	0.719084
C	1.200459	-1.232393	0.720256
C	2.454836	1.235945	0.192804
H	0.691007	2.166130	0.929029
C	2.458255	-1.237297	0.195142
H	0.695872	-2.170599	0.931033
C	3.119610	-0.000187	-0.082570
H	2.966532	2.168332	-0.020815
H	2.972233	-2.168808	-0.016773
N	4.350916	0.001144	-0.590349
H	4.839877	-0.861172	-0.784659
H	4.837345	0.864469	-0.786493
C	-2.277263	0.000547	-0.424249
C	-1.804196	1.234735	-0.938048
N	-1.358967	2.239529	-1.317779
C	-3.516974	-0.036226	0.265743
N	-4.507428	-0.066418	0.873512
C	-1.756309	-1.194724	-0.981198
N	-1.271247	-2.167015	-1.395256

Structure PAB-CH_2CHOH^+

C	0.399059	-0.479323	1.268367
C	-0.346927	0.514290	0.619581
C	0.283124	1.238671	-0.405573
C	1.593074	0.974996	-0.773445
C	2.333180	-0.034505	-0.126978
C	1.715553	-0.755790	0.904201
H	-0.053430	-1.046145	2.079173
H	-0.258684	2.028143	-0.921235
H	2.059166	1.551302	-1.567485
H	2.267784	-1.532511	1.424590
H	3.937775	0.029921	-1.389970
N	3.656516	-0.261323	-0.463730
H	4.034116	-1.163306	-0.207390
C	-1.786020	0.789281	1.008740
H	-2.063942	0.155828	1.856092
H	-1.901596	1.825477	1.338377
C	-2.796231	0.566587	-0.124600
H	-3.832301	0.709396	0.203502
H	-2.677145	1.298990	-0.943791
C	-2.751021	-0.723081	-0.821260
O	-1.795452	-1.539554	-0.779396
H	-3.575405	-1.040205	-1.460051
H	-1.031083	-1.217955	-0.224744

Structure PAB-CH_2CHOH^+-ts

C	0.307862	-0.507436	1.162551
C	-0.270758	0.692084	0.638553
C	0.498666	1.452694	-0.299426
C	1.754814	1.065990	-0.664128
C	2.325830	-0.122848	-0.110643

C	1.568432	-0.901501	0.813923
H	-0.270775	-1.104262	1.861958
H	0.068776	2.358395	-0.718288
H	2.336073	1.650926	-1.368901
H	2.003341	-1.806293	1.224610
H	3.980521	-1.332366	-0.079704
N	3.557302	-0.498247	-0.460744
H	4.104512	0.042458	-1.115053
C	-1.540244	1.101463	1.022032
H	-2.053965	0.606013	1.838756
H	-1.907554	2.084382	0.744840
C	-3.112007	0.422008	-0.618238
H	-4.008191	0.865313	-0.202119
H	-2.592844	0.955772	-1.408413
C	-2.816914	-0.863293	-0.317563
O	-1.773680	-1.548615	-0.784396
H	-3.378019	-1.433274	0.416756
H	-1.287630	-1.045375	-1.456429

9. *C*-benzylation of *ortho*-quinones

Sodium dithionite is a well-known reductant able to convert NAD to NADH, as well as reducing aromatic compounds[7] and quinones.[8,9] The alkylation reaction requires basic media and protic solvents, most commonly water. Either the dithionite intermediate or the carbanion/enolate generated after deprotonation of the reduced intermediate with KOH, would undergo nucleophilic attack to the benzyl bromide electrophile. The initial alkylation reaction does not work in the absence of base, which we believe is necessary to deprotonate the hydroquinone. The di-O-alkylation adducts of quinones, despite being fully aromatic (formally they are hydroquinone ethers), have been reported with alkyl halides. However, the mono-*O*-benzyl derivatives are reported to be unstable. Mono-*O*-alkyl phenanthrenequinone derivatives have been reported to undergo thermal interconversion to the corresponding *C*-alkylation products.[10,11] A radical mechanism for the transformation, occurring in the presence of light and a second quinone molecule, was proposed by Shurygina et al.[10,11] It is also possible to propose a non-radical mechanism, which could occur under the highly basic reaction conditions (**Scheme S9**). To our knowledge, this rearrangement reaction has not been studied in detail.

Additionally, the two possible *C*-alkylation products (alpha-hydroxy ketones) can interconvert under basic conditions or thermal activation through the α-ketol rearrangement[12] (**Scheme S10**). In fact, we could locate transition states for such process, as shown by the Intrinsic Reaction Coordinate calculations shown in **Scheme S10** and **Figures S108, S112.** While such process is thermoneutral for phenanthrenequinone (reactants and products are the same species), for β-lapachone a clear thermodynamic preference of ca. 7 kcal/mol was calculated for the isomer alkylated at C-6. The observed regioselective reductive benzylation could be either kinetically or, more likely, thermodynamically controlled.

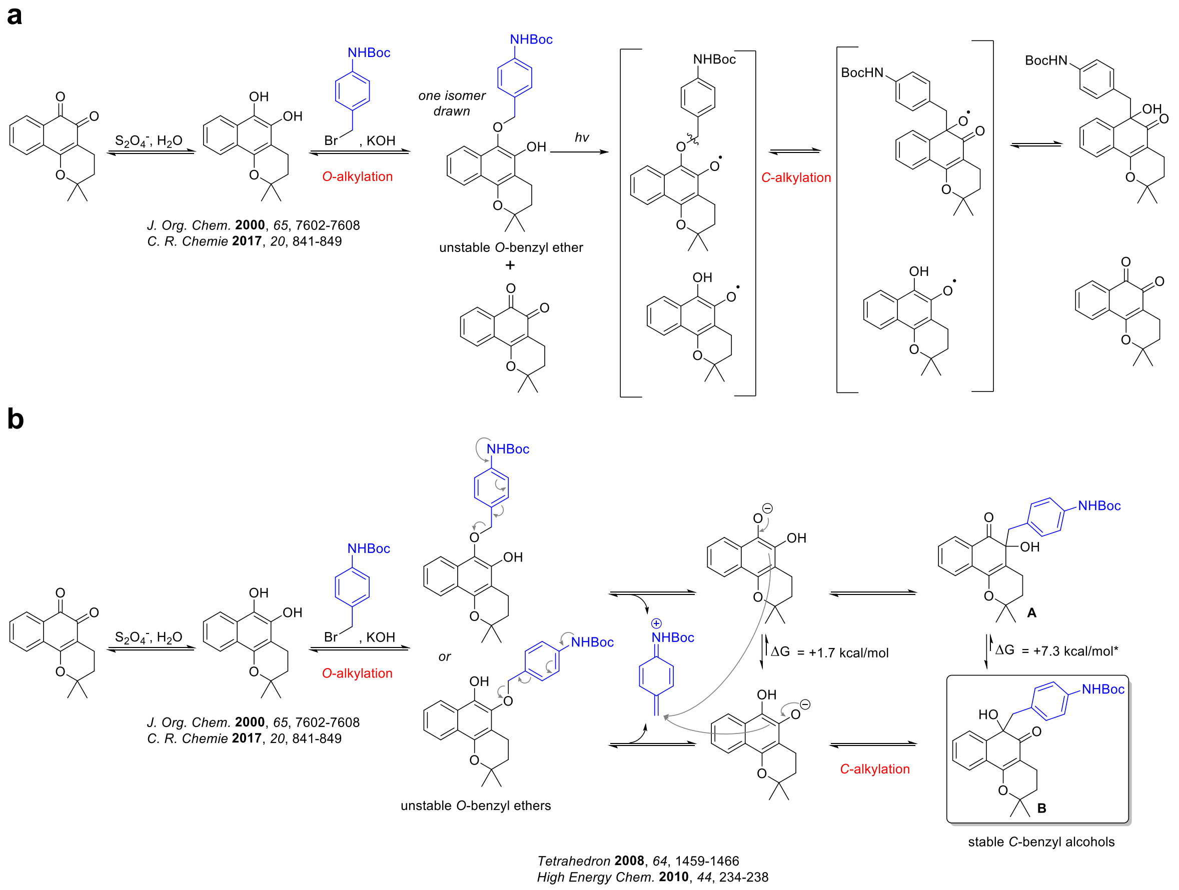

Scheme 9. Suggested mechanisms for *C*-benzylation of β-lapachone 1. a, radical-mediated mechanism. **b,** non-radical mediated mechanism.

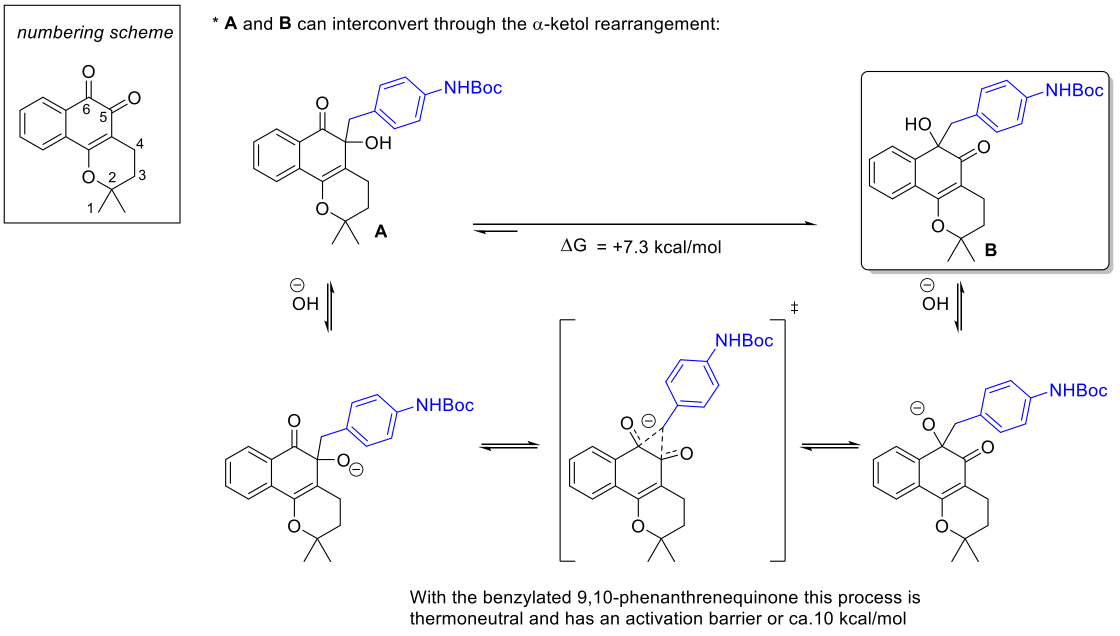

Scheme 10. α-ketol rearrangement of *ortho*-quinone β-lapachone.

10. ^{1}H and ^{13}C NMR Spectra

^{1}H NMR (400 MHz, $CDCl_3$) of β-lapachone **1**

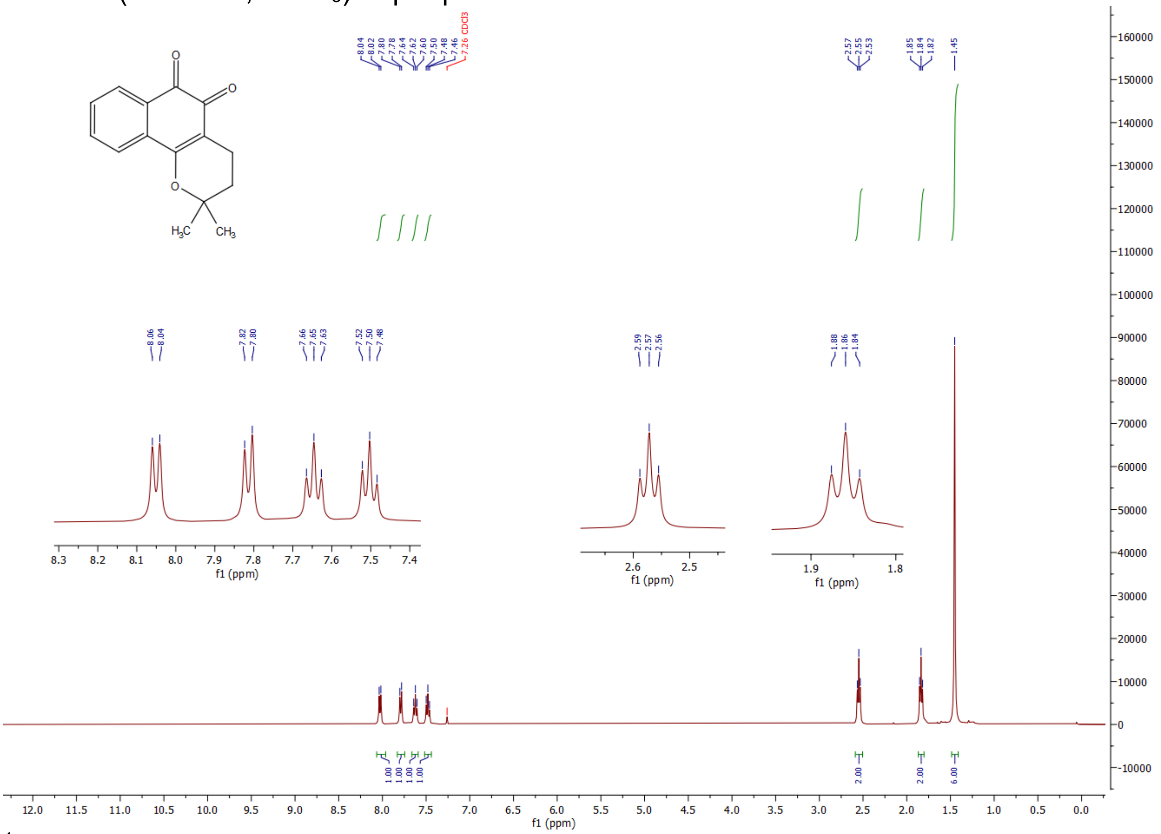

^{1}H NMR (400 MHz, MeOD) of β-lapachone **1**

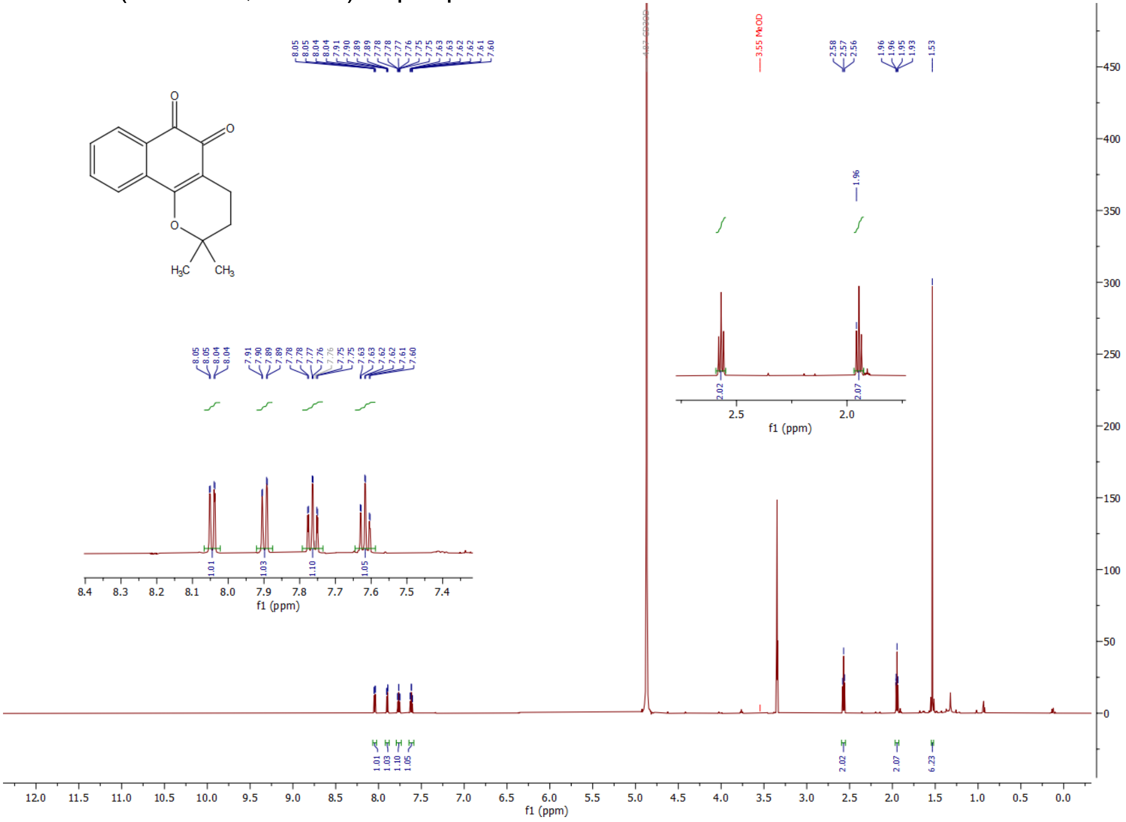

^{13}C NMR (100 MHz, $CDCl_3$) of β-lapachone **1**

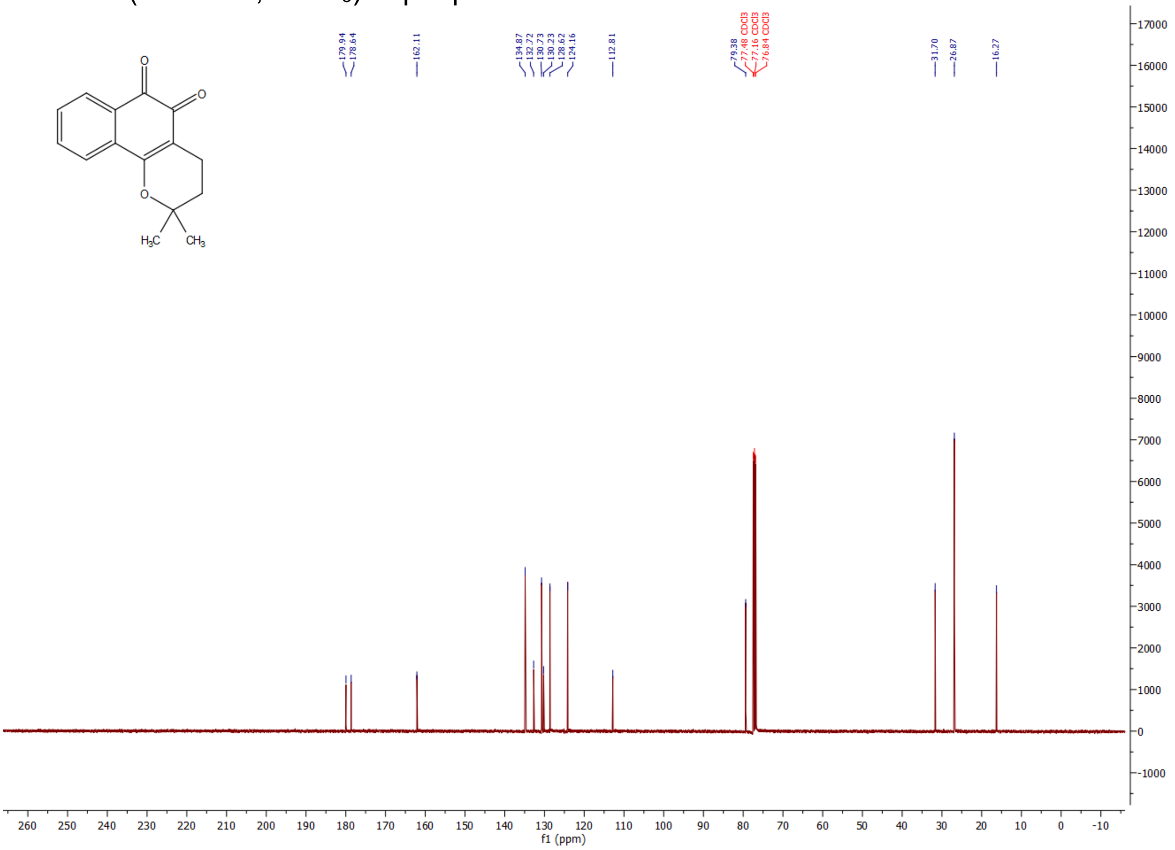

^{1}H NMR (400 MHz, MeOD) of 9,10-phenanthrenequinone **4**

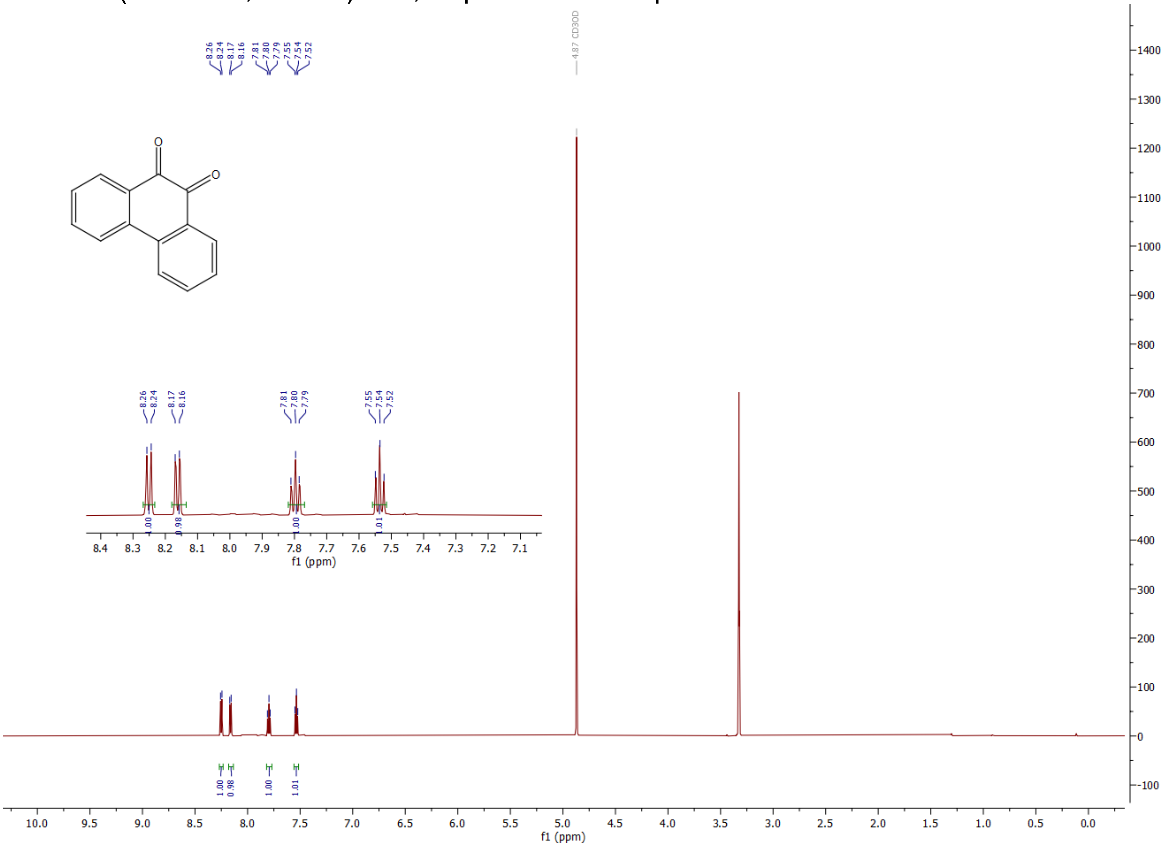

^{1}H NMR (400 MHz, CDCl3) of 9,10-phenanthrenequinone **4**

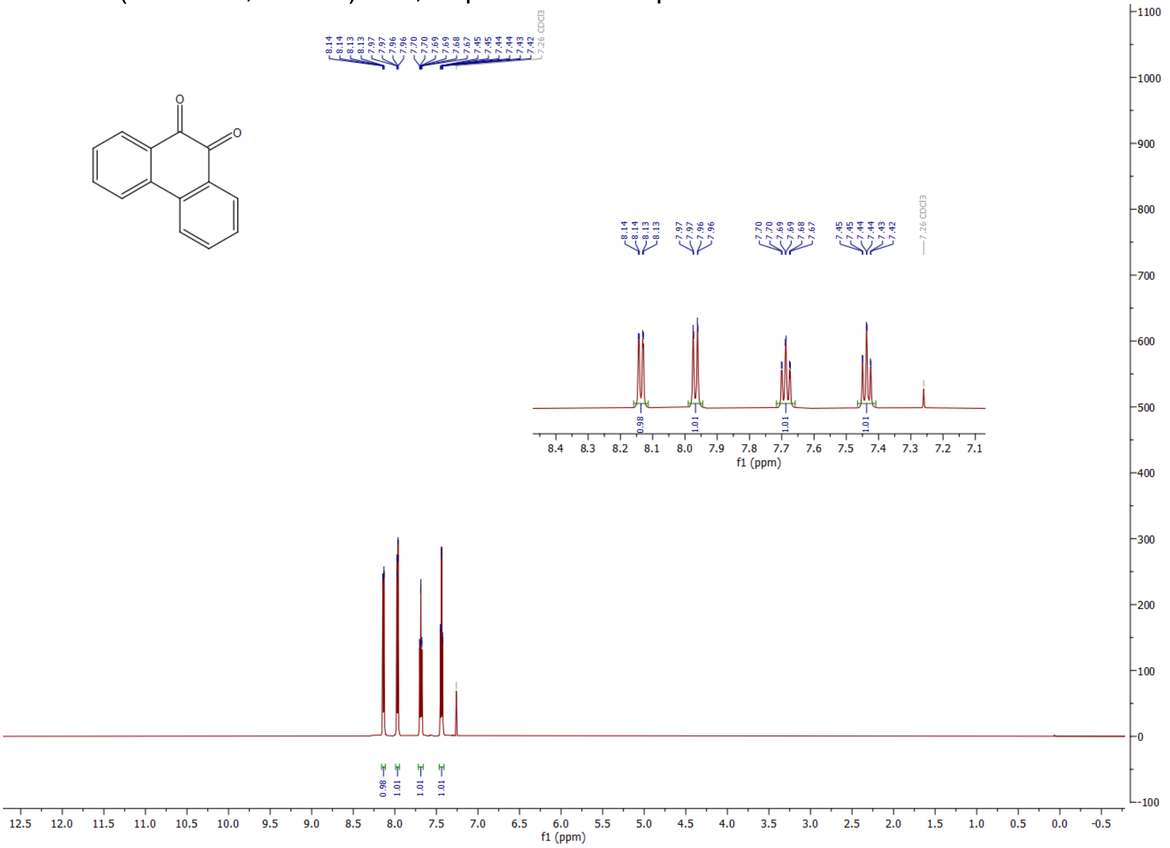

^{1}H NMR (400 MHz, $CDCl_3$) of dunnione **12**

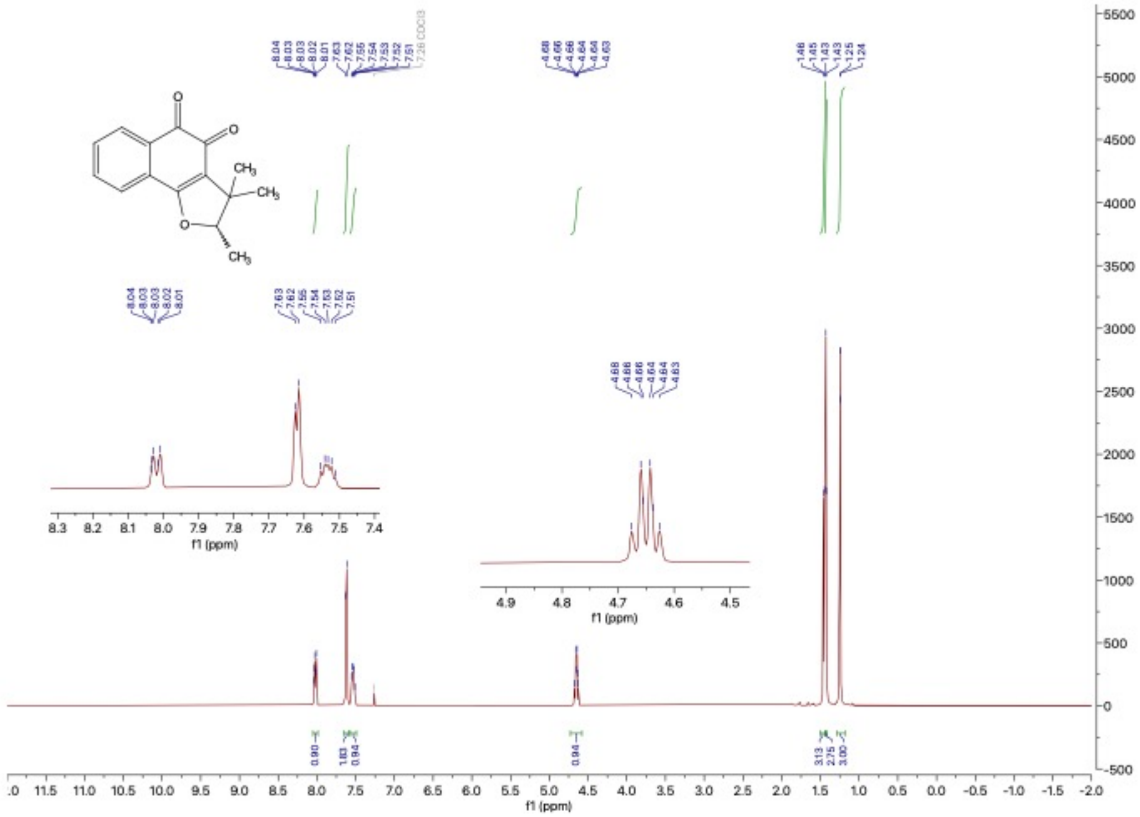

^{13}C NMR (101 MHz, $CDCl_3$) of dunnione **12**

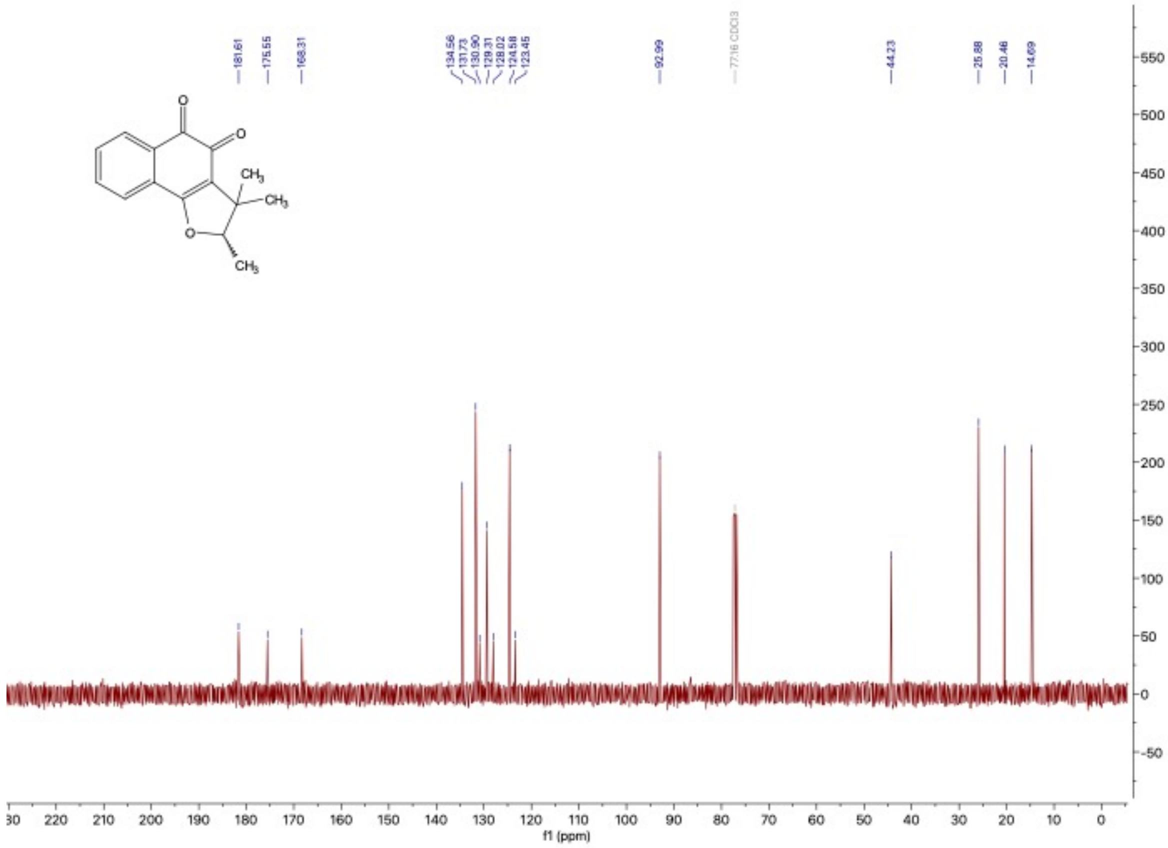

^{1}H NMR (400 MHz, $CDCl_3$) of *tert*-butyl (4-(hydroxymethyl)phenyl)carbamate **S1**

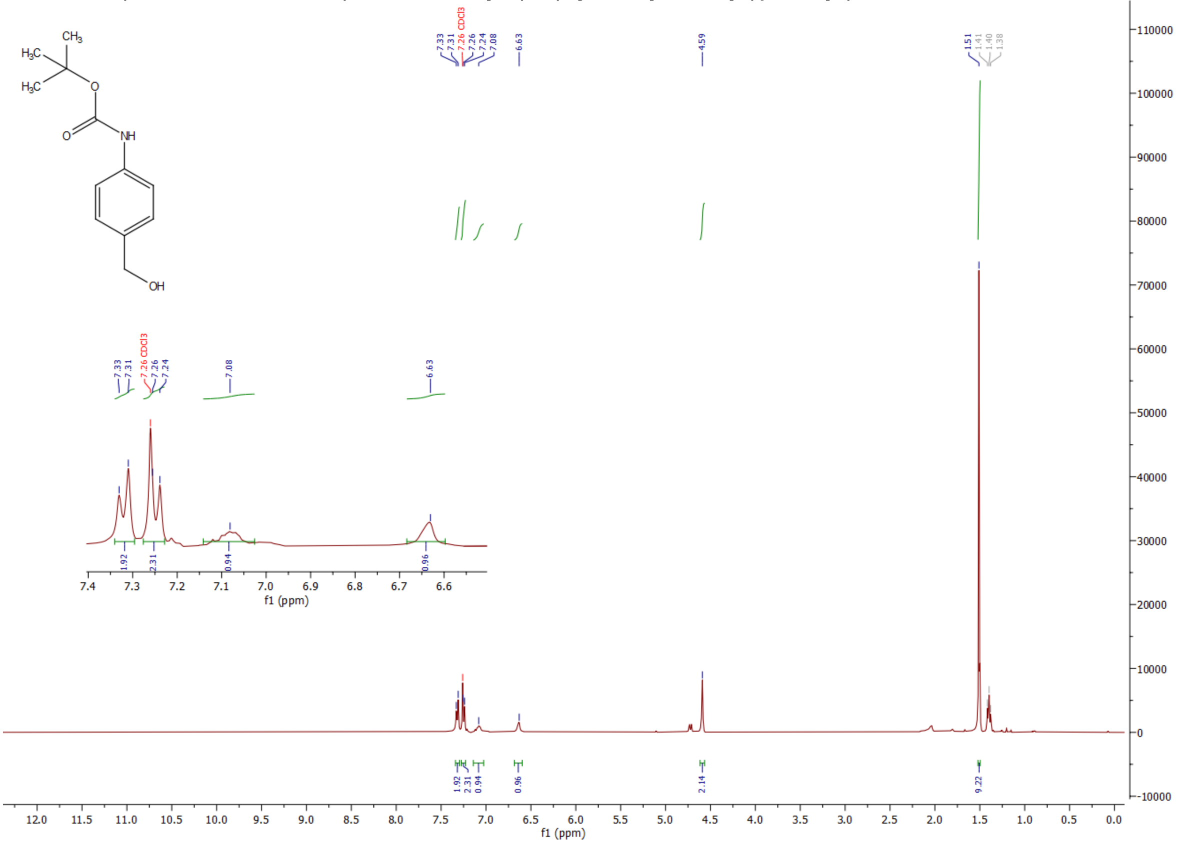

^{13}C NMR (100 MHz, $CDCl_3$) of *tert*-butyl (4-(hydroxymethyl)phenyl)carbamate **S1**

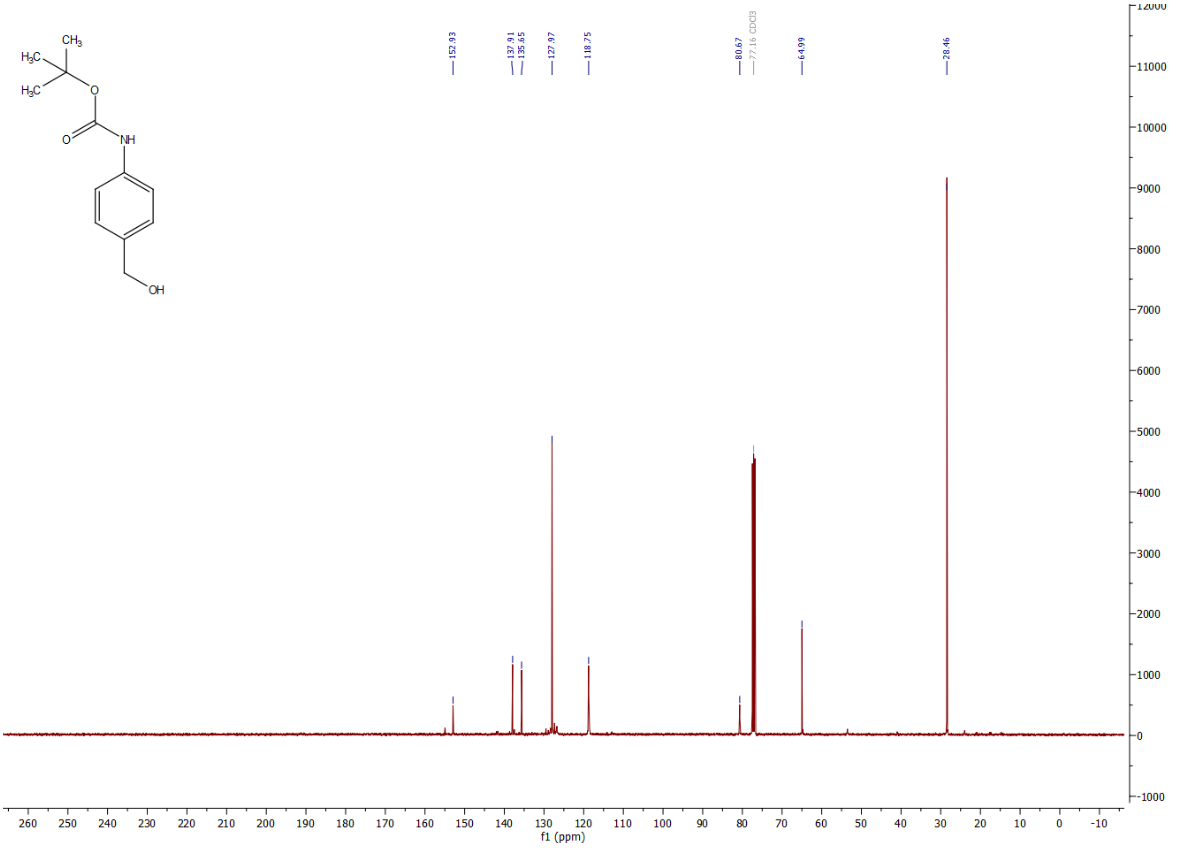

^{1}H NMR (400 MHz, $CDCl_3$) of *tert*-butyl (4-(bromomethyl)phenyl)carbamate **5**

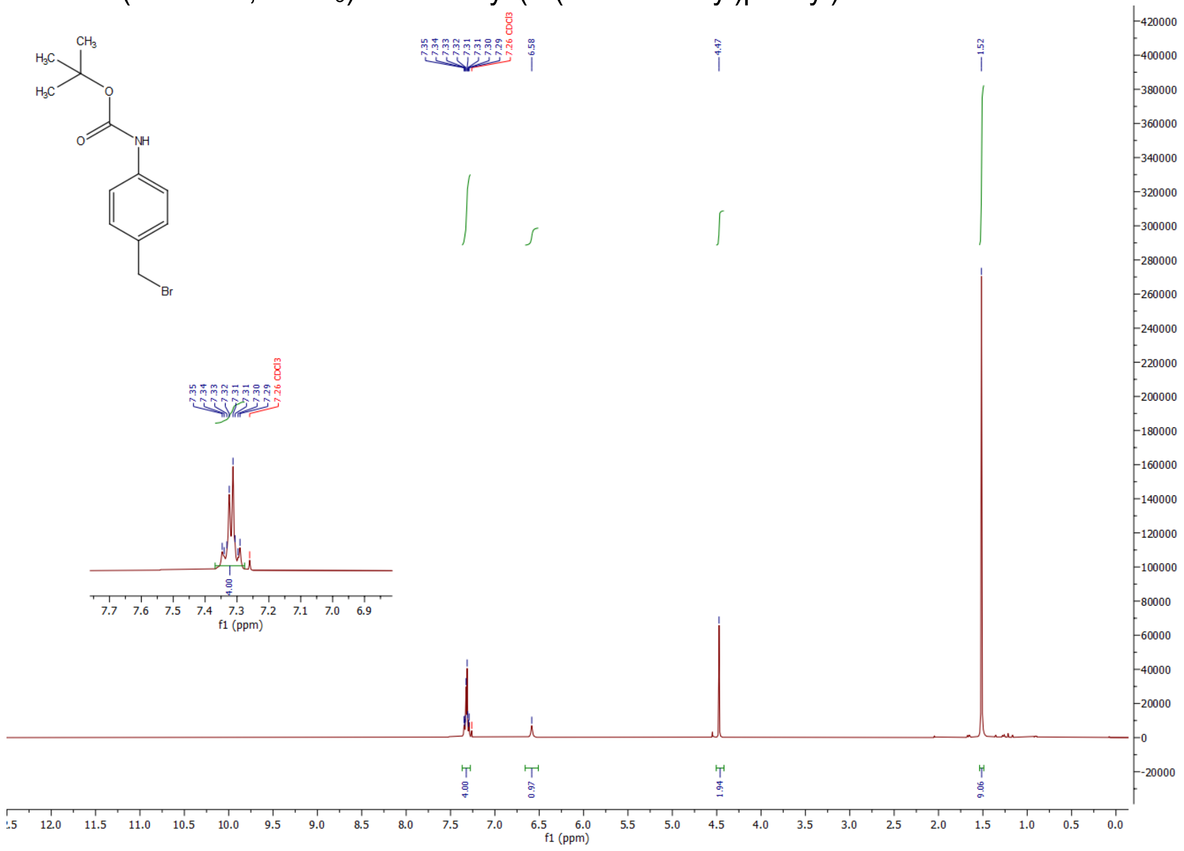

^{13}C NMR (100 MHz, $CDCl_3$) of *tert*-butyl (4-(bromomethyl)phenyl)carbamate **5**

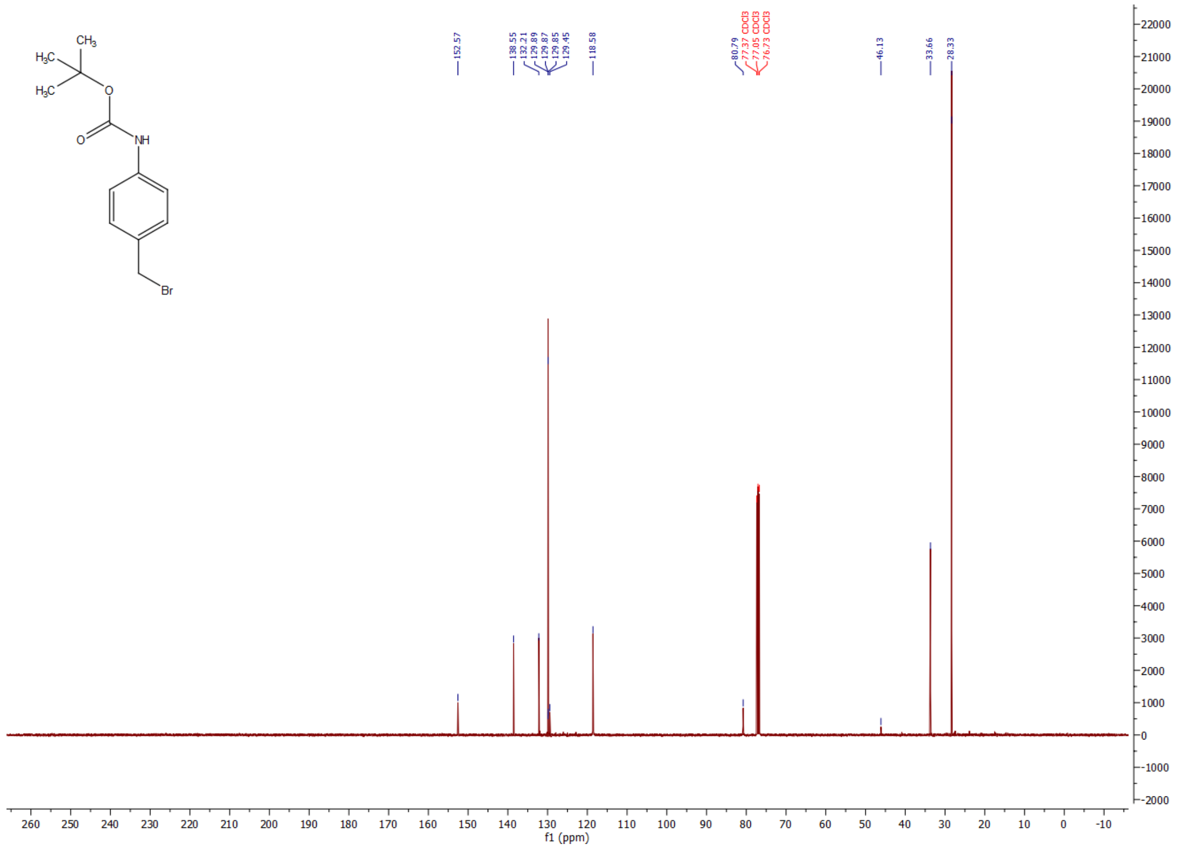

^{1}H NMR (600 MHz, $CDCl_3$) of Boc-*para*-aminobenzyl phenanthrene-ketol **6**

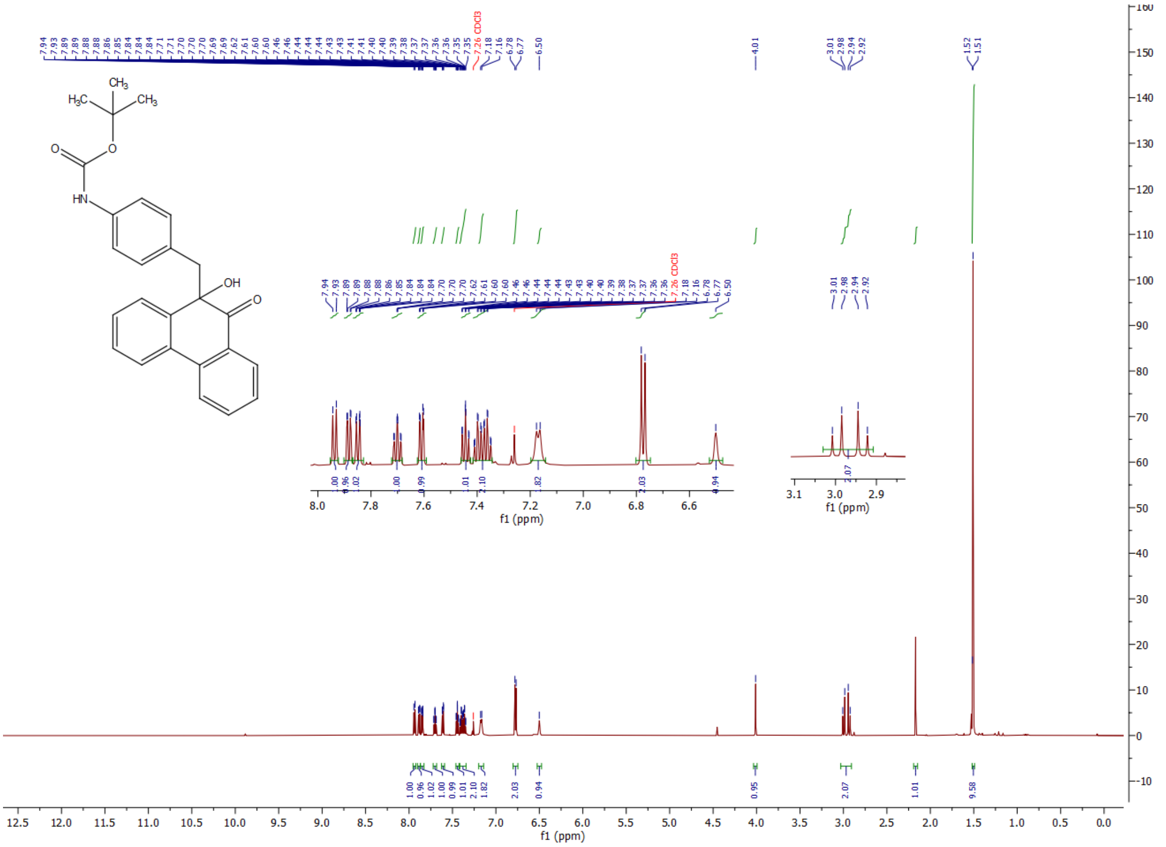

^{13}C NMR (151 MHz, $CDCl_3$) of Boc-*para*-aminobenzyl phenanthrene-ketol **6**

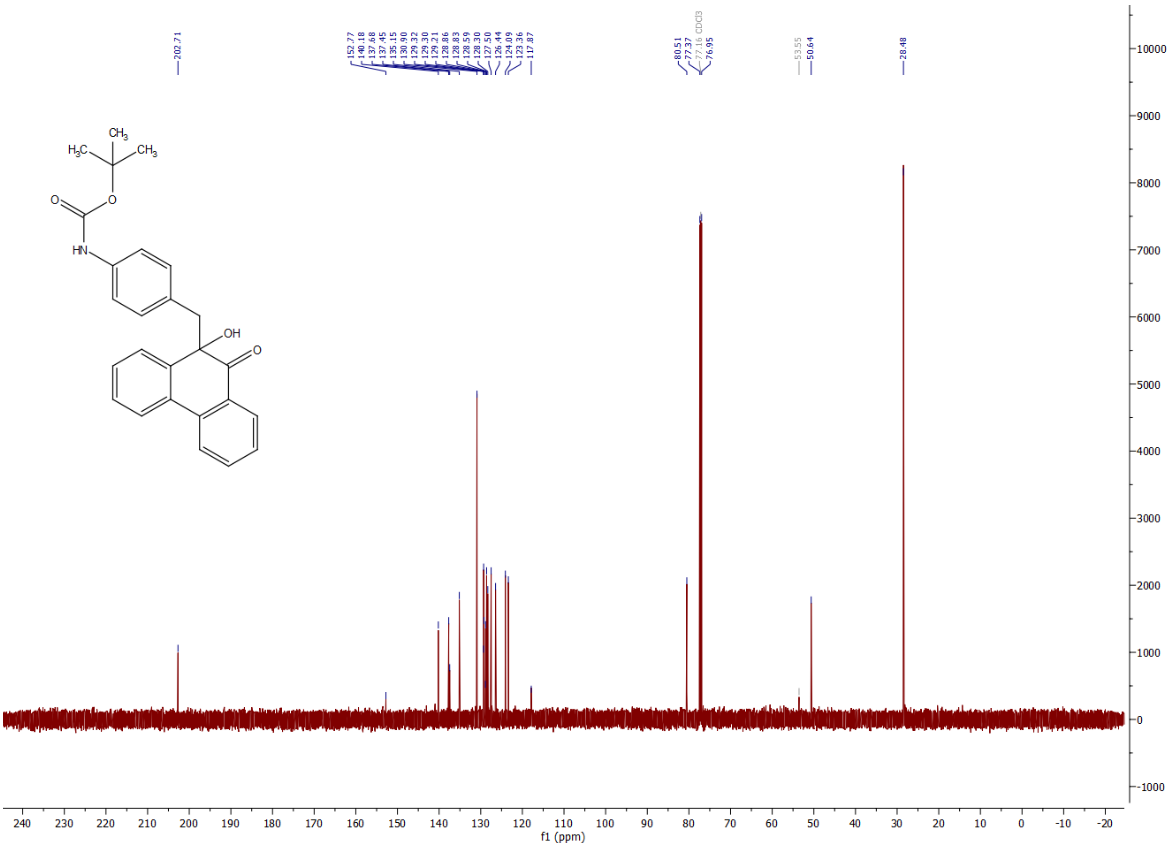

^{1}H NMR (600 MHz, MeOD) of *para*-aminobenzyl phenanthrene-ketol **7**

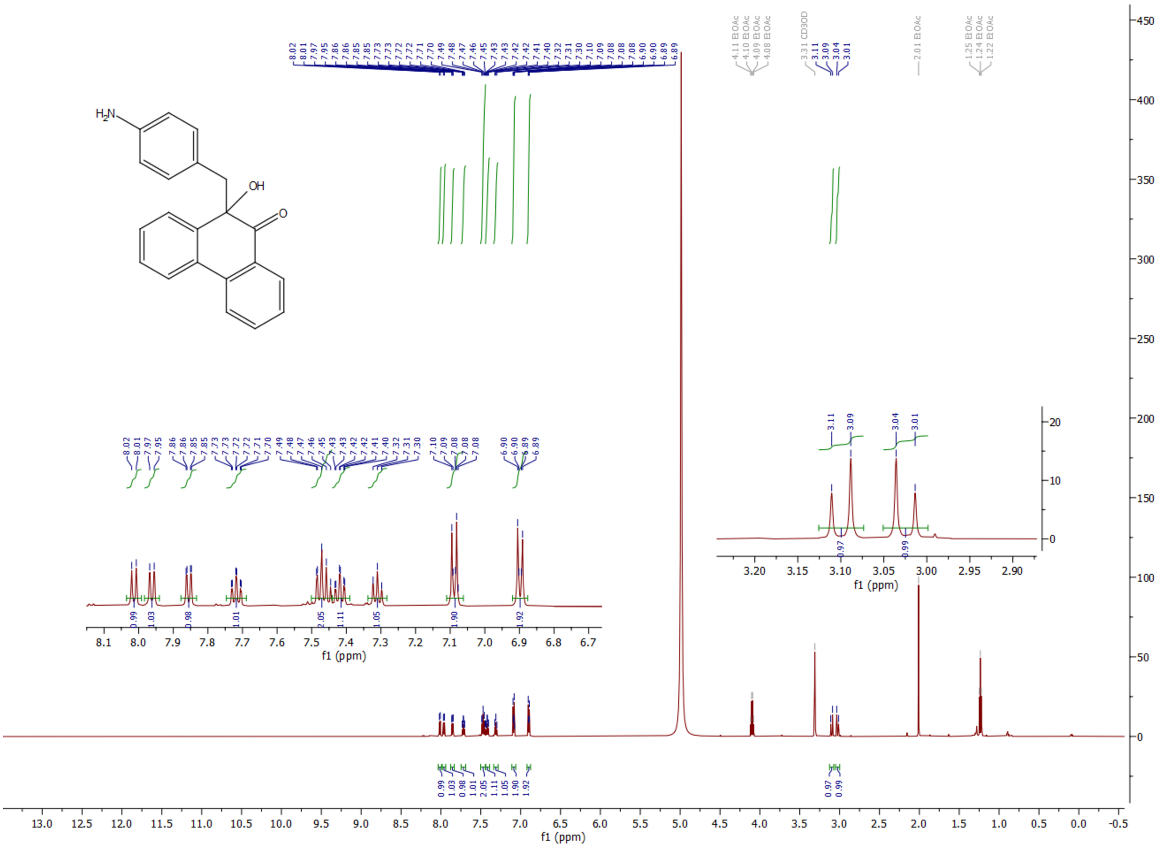

^{1}H NMR (400 MHz, $CDCl_3$) of 9,10-phenanthrenediol **8**

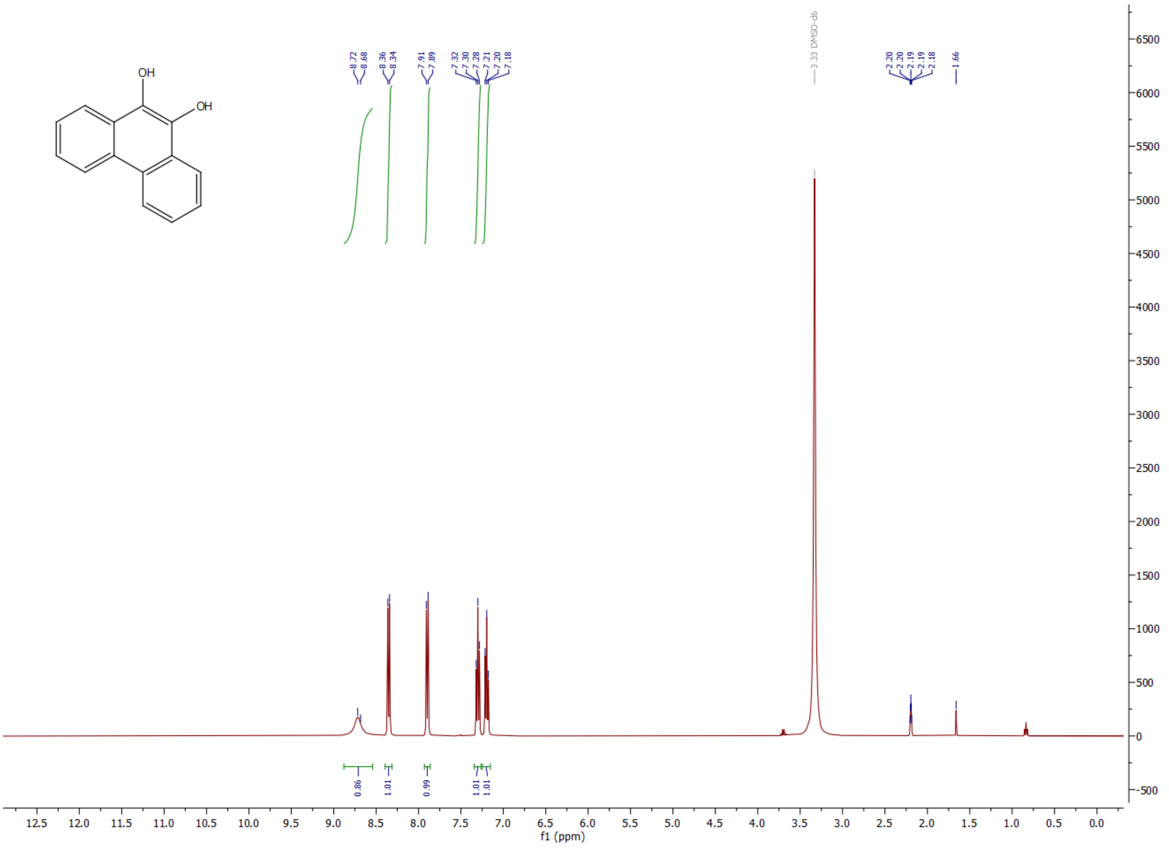

^{13}C NMR (100 MHz, $CDCl_3$) of 9,10-phenanthrenediol **8**

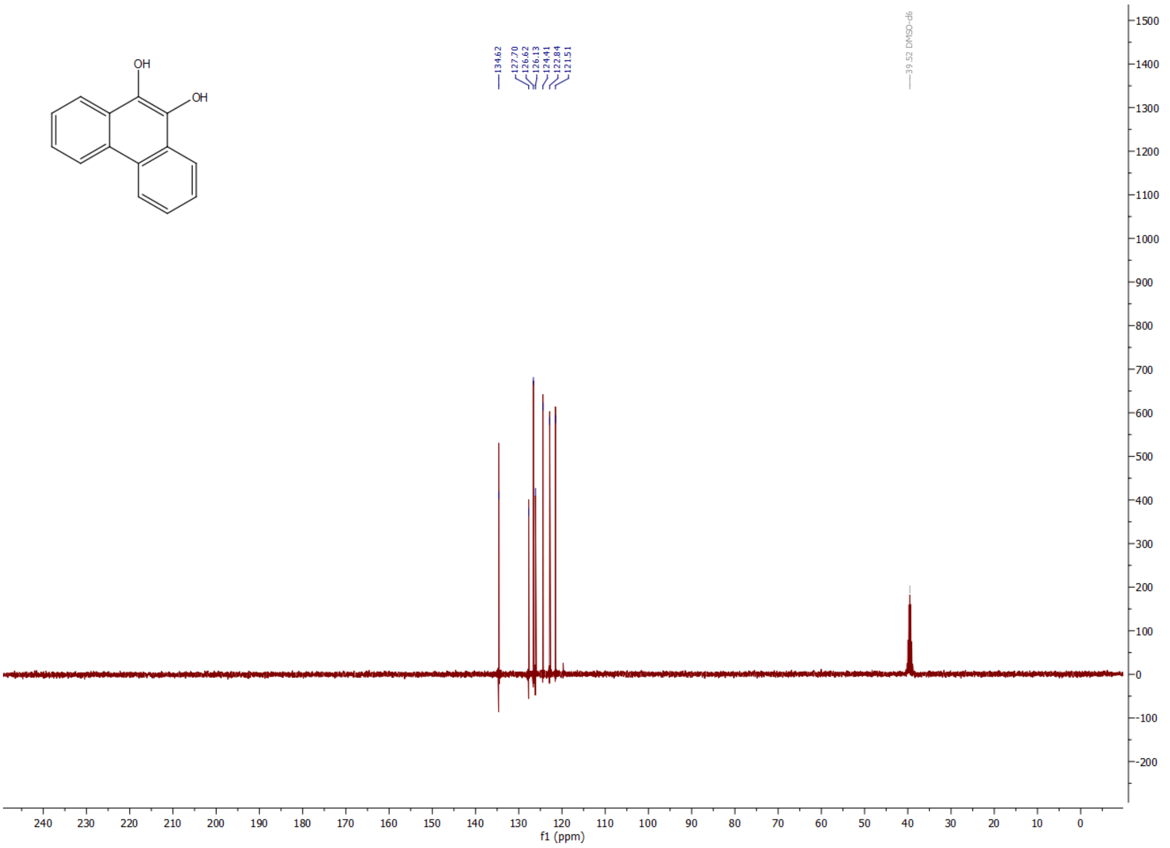

^{1}H NMR (400 MHz, $CDCl_3$) of Boc-*para*-aminobenzyl β-lapa-ketol **9**

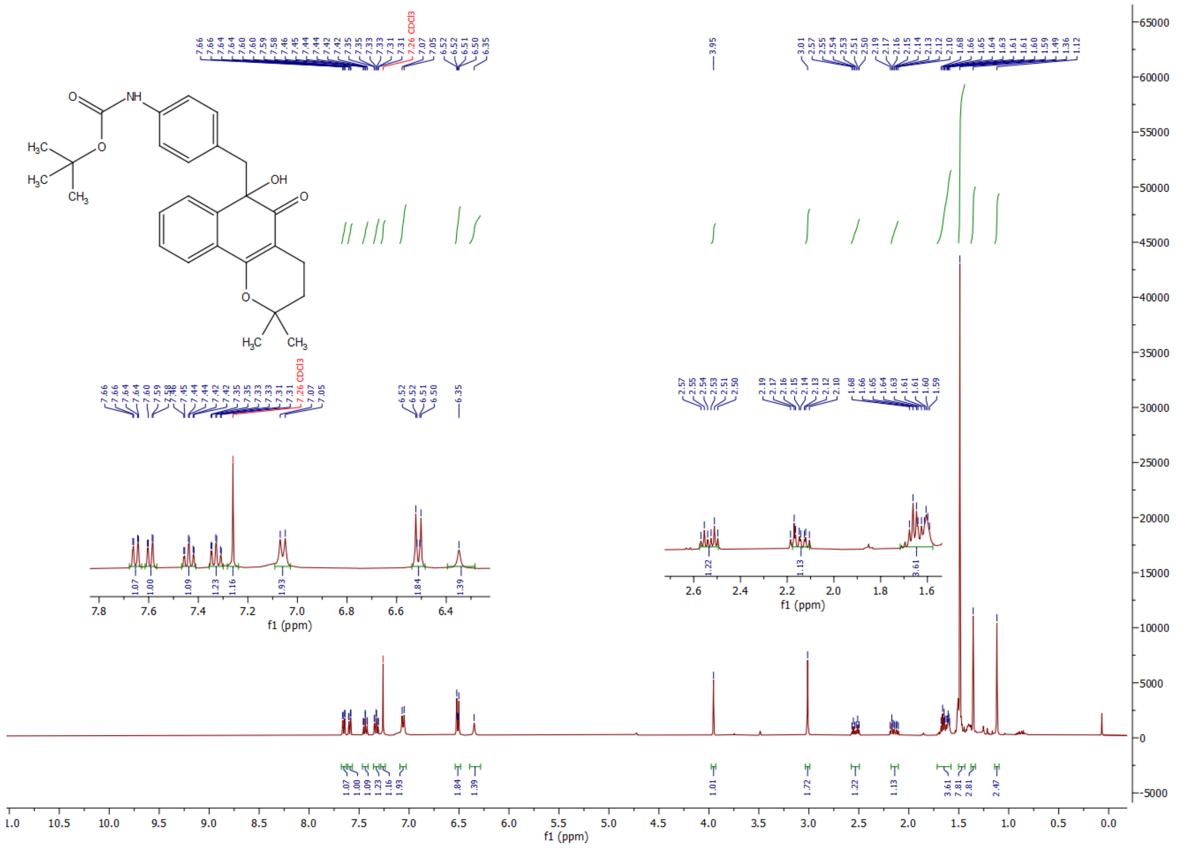

^{13}C NMR (100 MHz, $CDCl_3$) of Boc-*para*-aminobenzyl β-lapa-ketol **9**

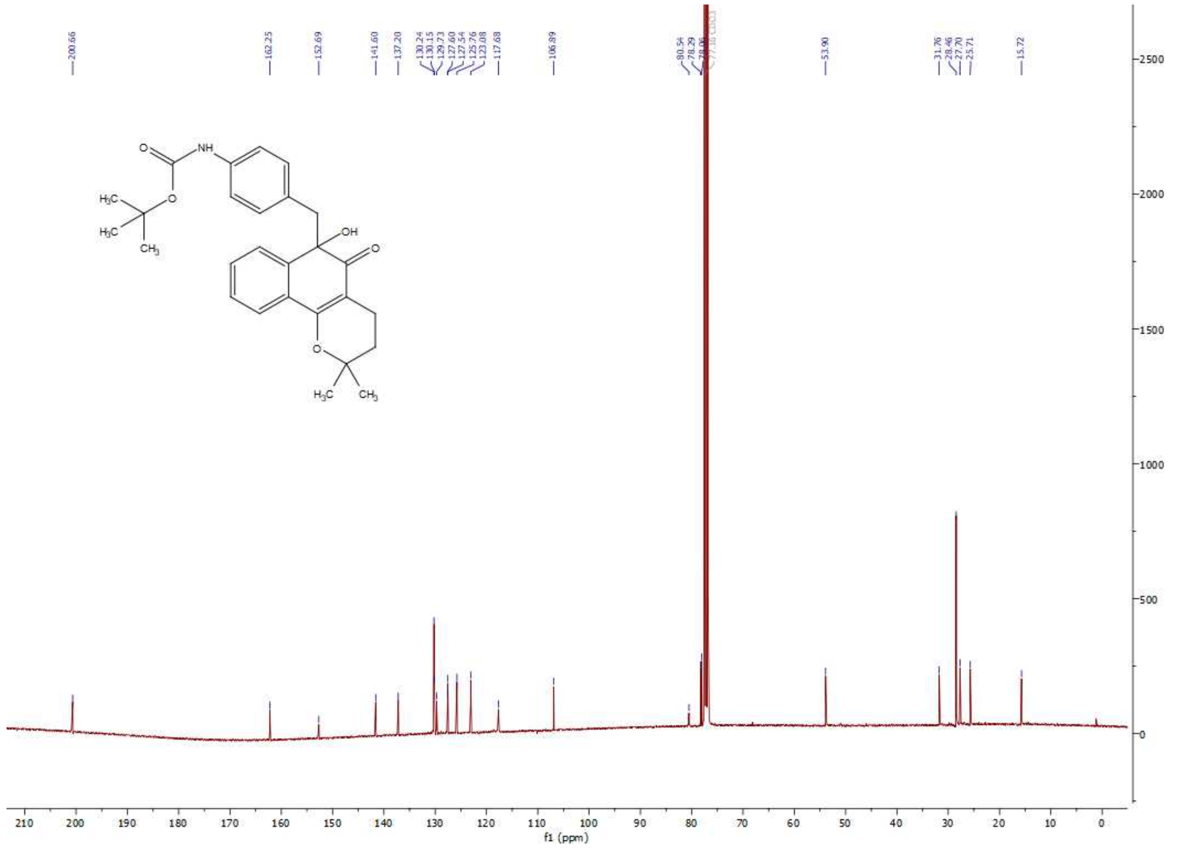

^{1}H NMR (600 MHz, MeOD) of *para*-aminobenzyl β-lapa-ketol **10**

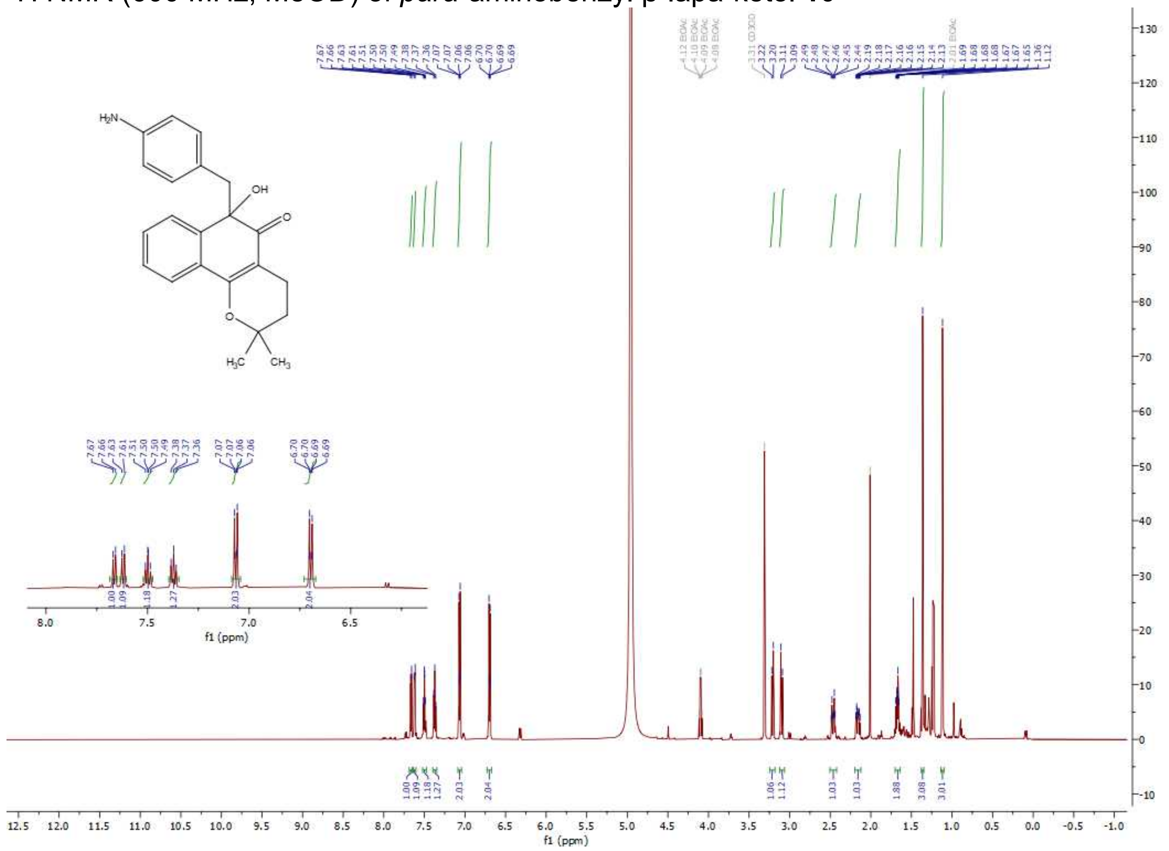

^{1}H NMR (500 MHz, $CDCl_3$) of Boc-*para*-aminobenzyl dunni-ketol **15**

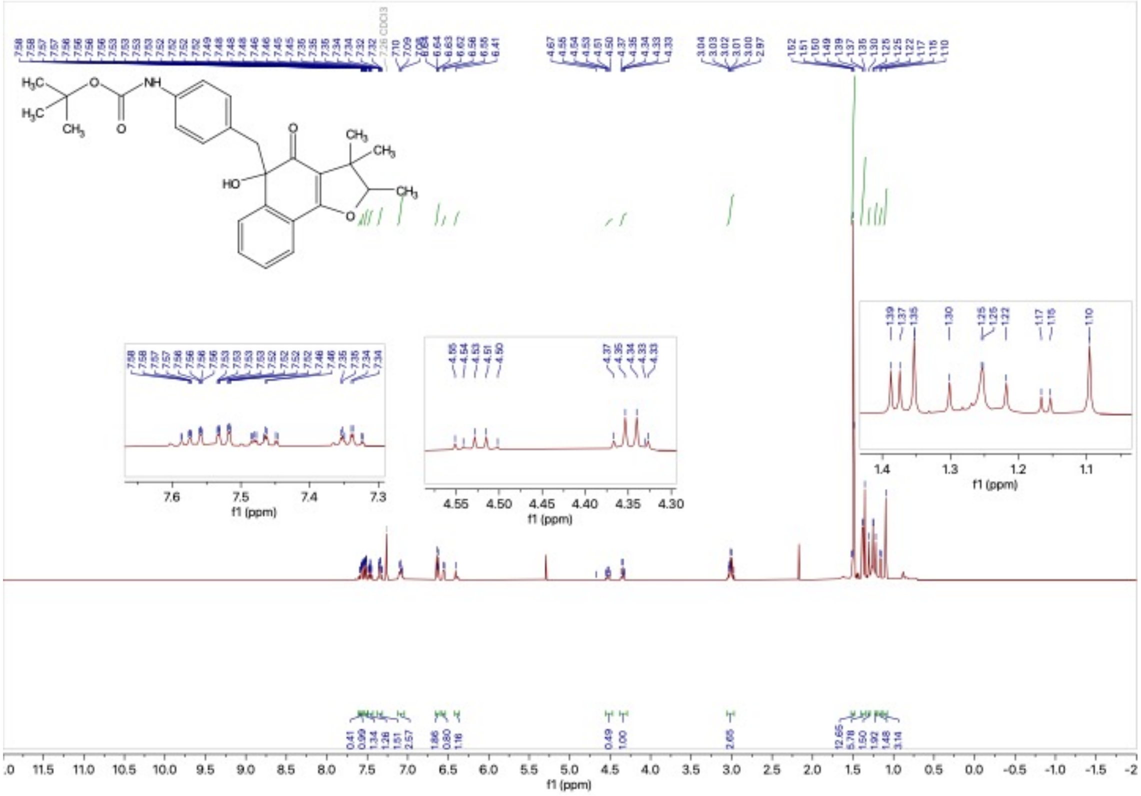

^{13}C NMR (126 MHz, $CDCl_3$) of Boc-*para*-aminobenzyl dunni-ketol **15**

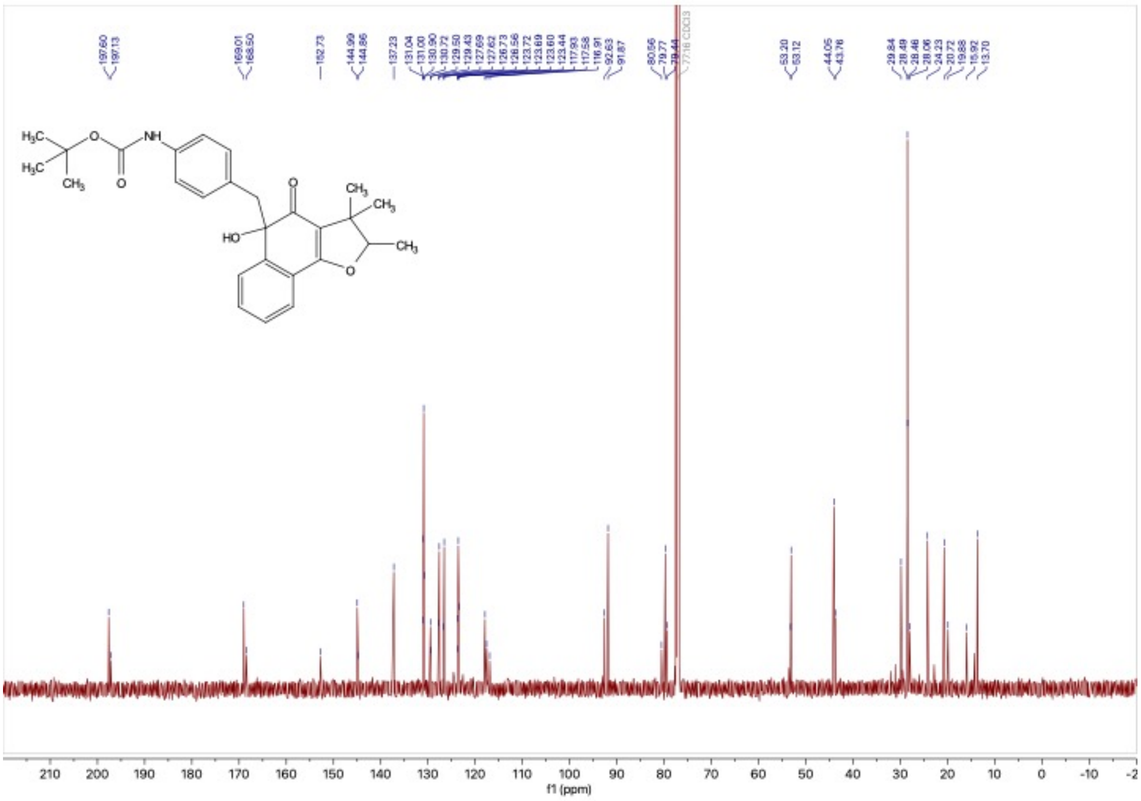

^{1}H NMR (400 MHz, $CDCl_3$) of *O*-acetyl-3-hydroxy-β-lapachone **S2**

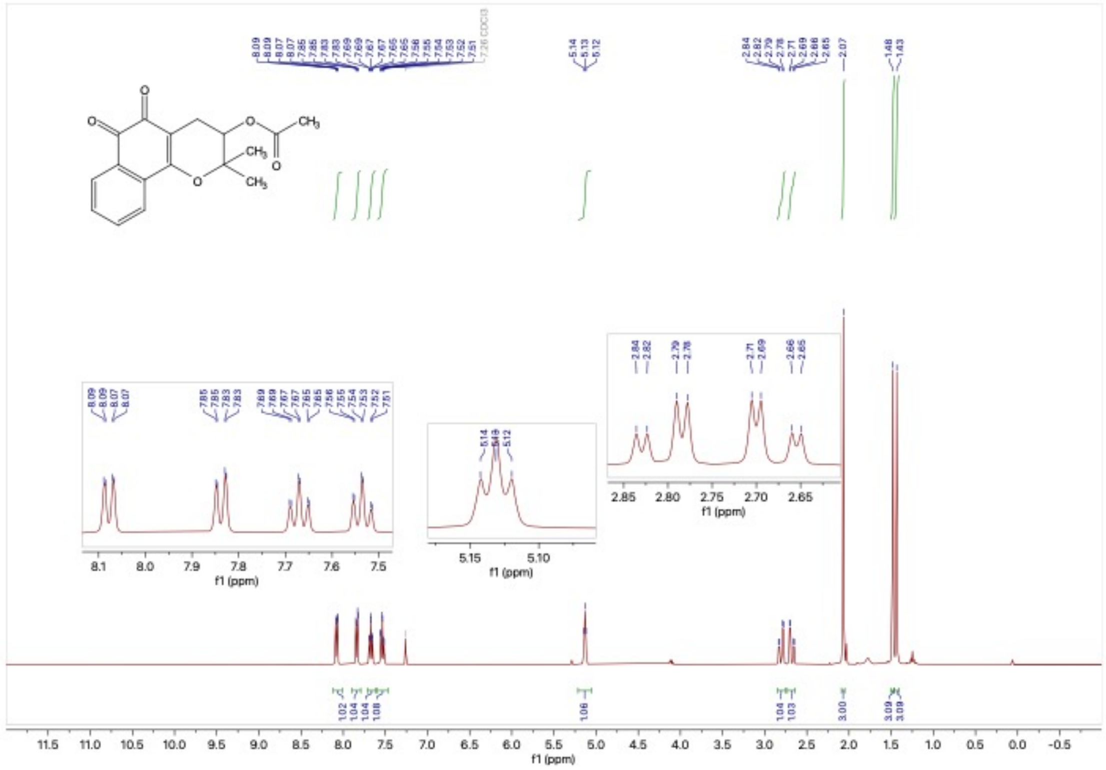

^{13}C NMR (101 MHz, $CDCl_3$) of *O*-acetyl-3-hydroxy-β-lapachone **S2**

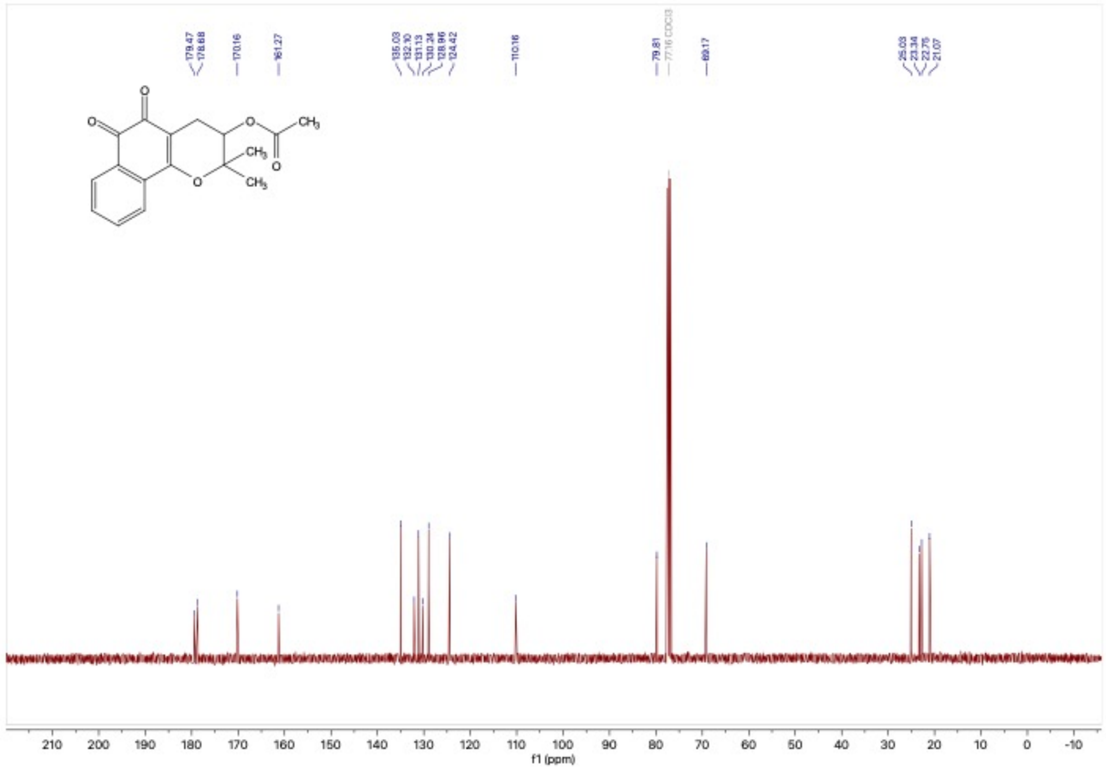

^{1}H NMR (500 MHz, $CDCl_3$) of Boc-*para*-aminobenzyl *O*-acetyl-β-lapa-ketol **S3**

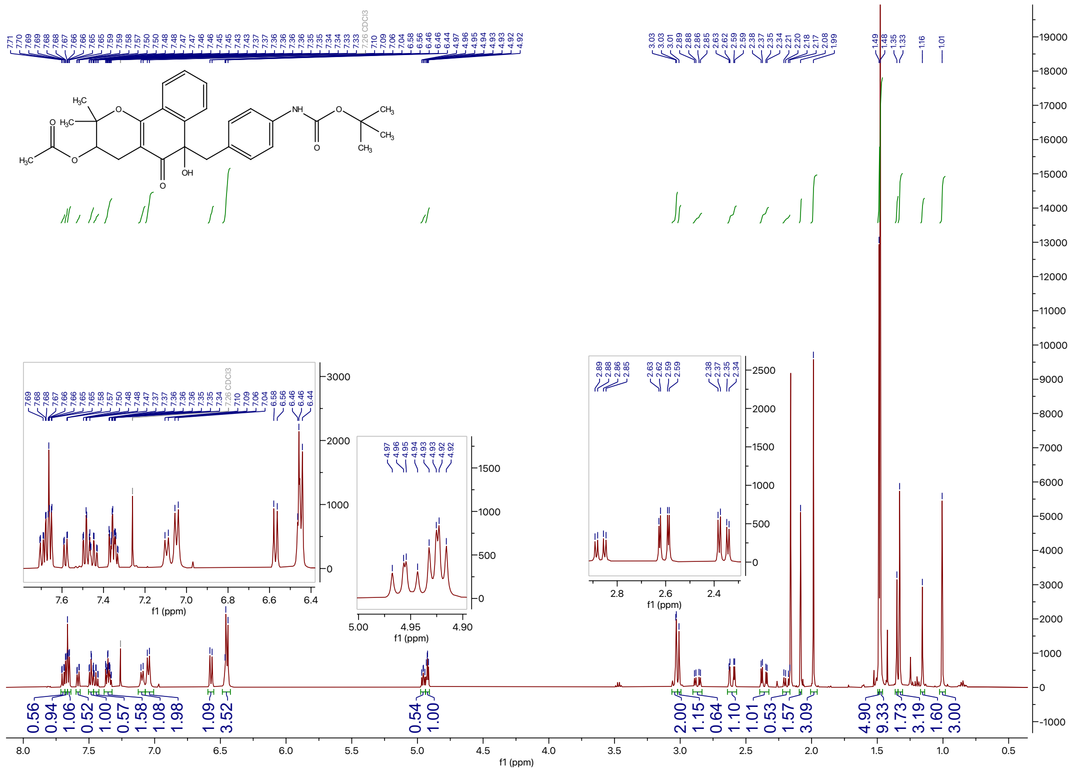

^{13}C NMR (126 MHz, $CDCl_3$) of Boc-*para*-aminobenzyl *O*-acetyl-β-lapa-ketol **S3**

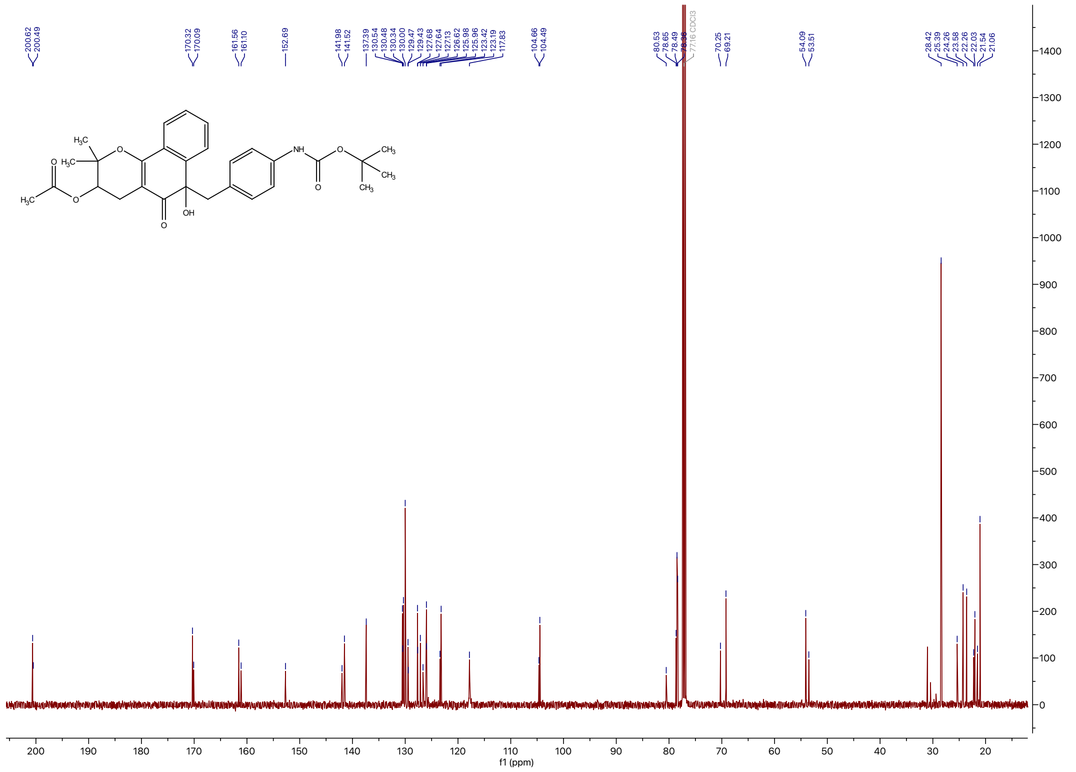

^{1}H NMR (500 MHz, $CDCl_3$) of Boc-*para*-aminobenzyl 3-hydroxy-β-lapa-ketol **14**

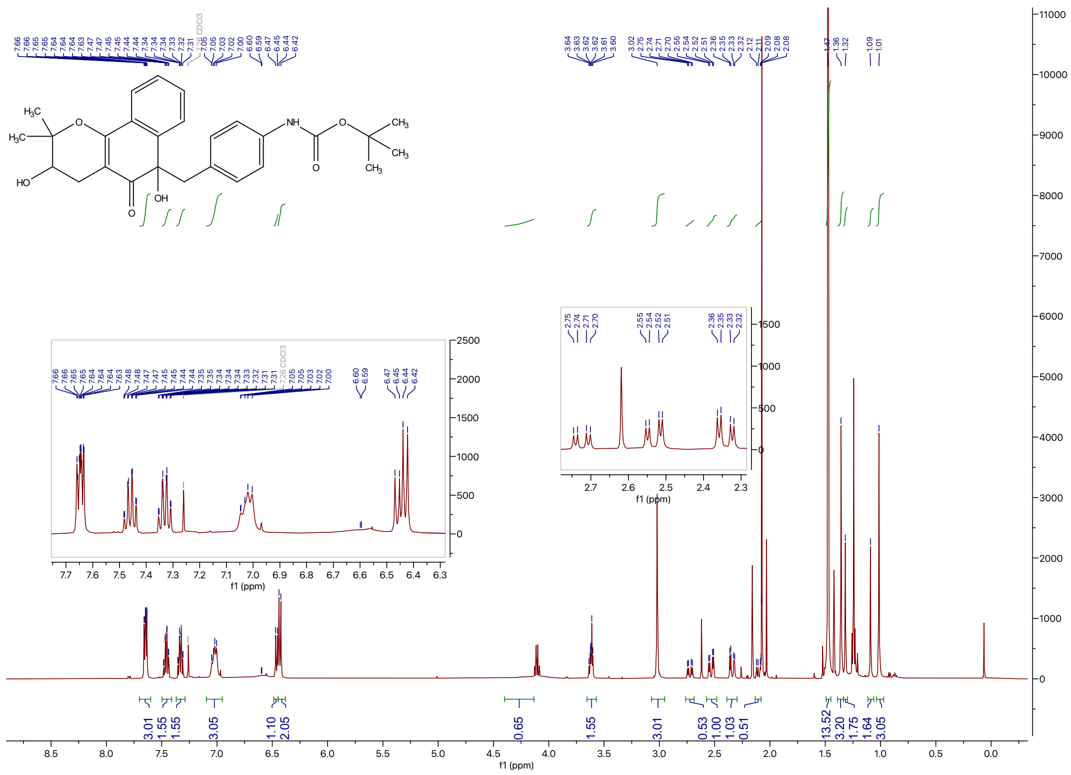

^{13}C NMR (126 MHz, $CDCl_3$) of Boc-*para*-aminobenzyl 3-hydroxy-β-lapa-ketol **14**

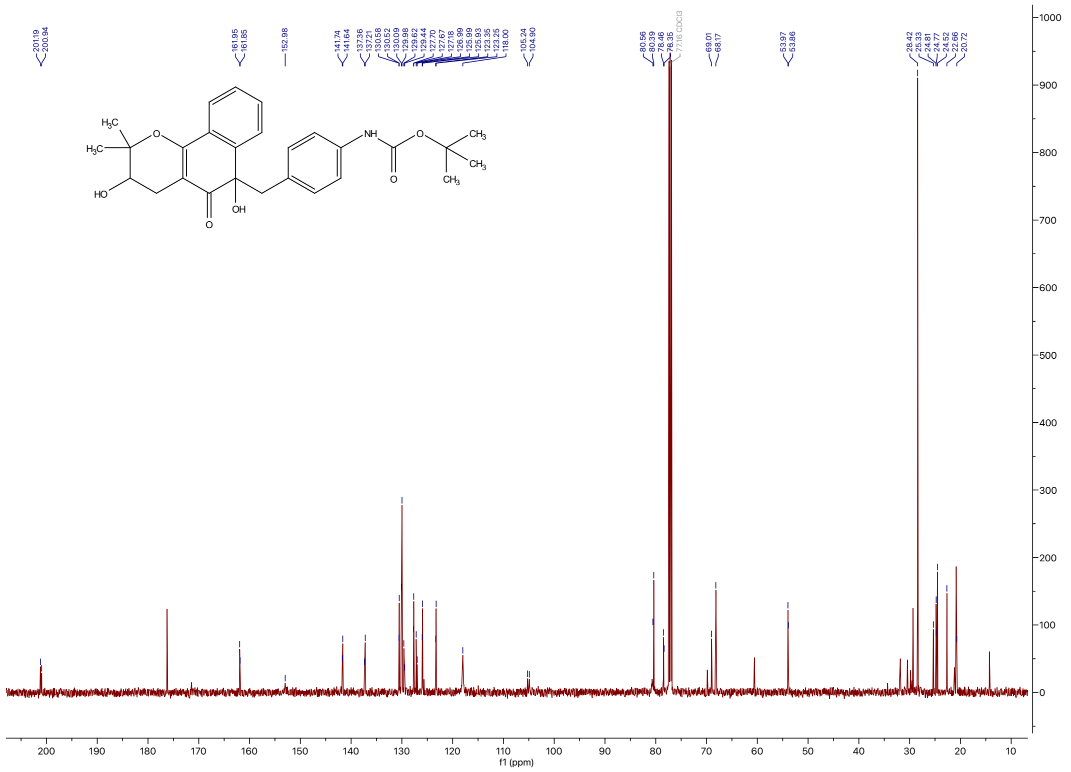

^{1}H NMR (500 MHz, $CDCl_3$) of Boc-*para*-aminobenzyl cryptotanshi-ketol **16**

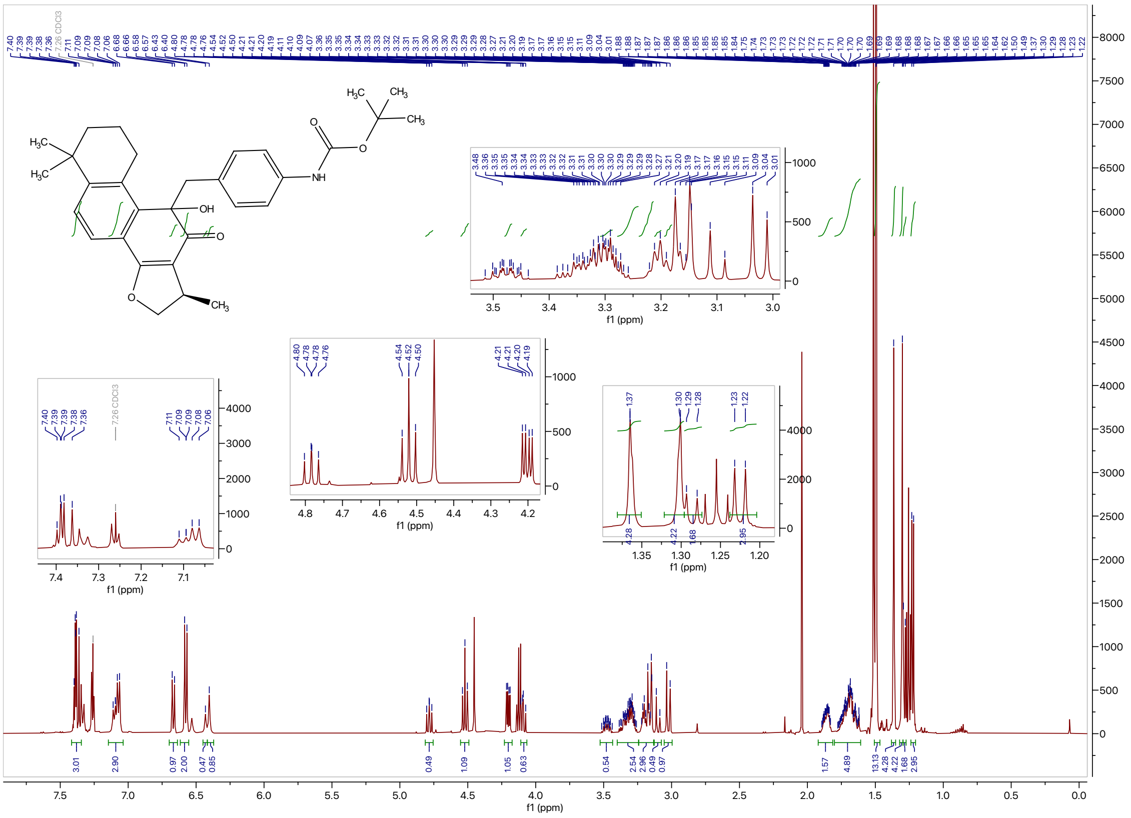

^{13}C NMR (126 MHz, $CDCl_3$) of Boc-*para*-aminobenzyl cryptotanshi-ketol **16**

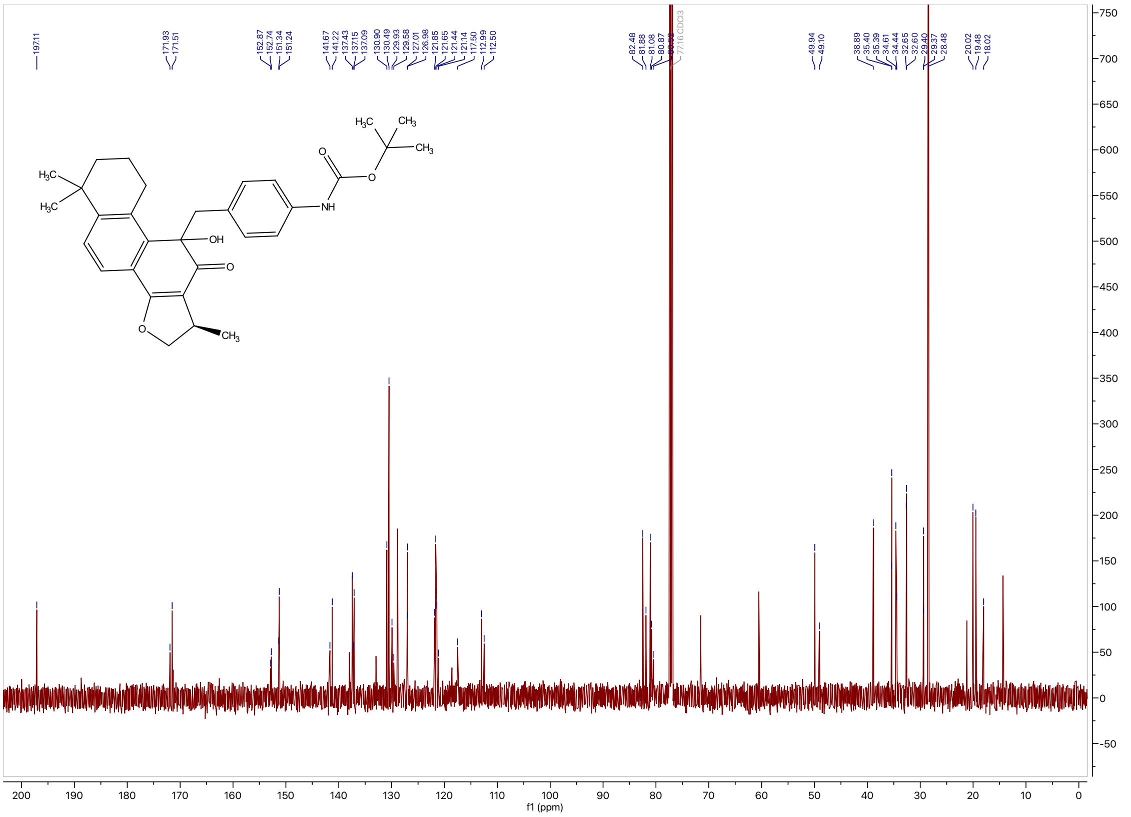

^{1}H NMR (400 MHz, $CDCl_3$) of 2-phenyl-acetyl-*para*-aminobenzyl phenanthrene-ketol **21**

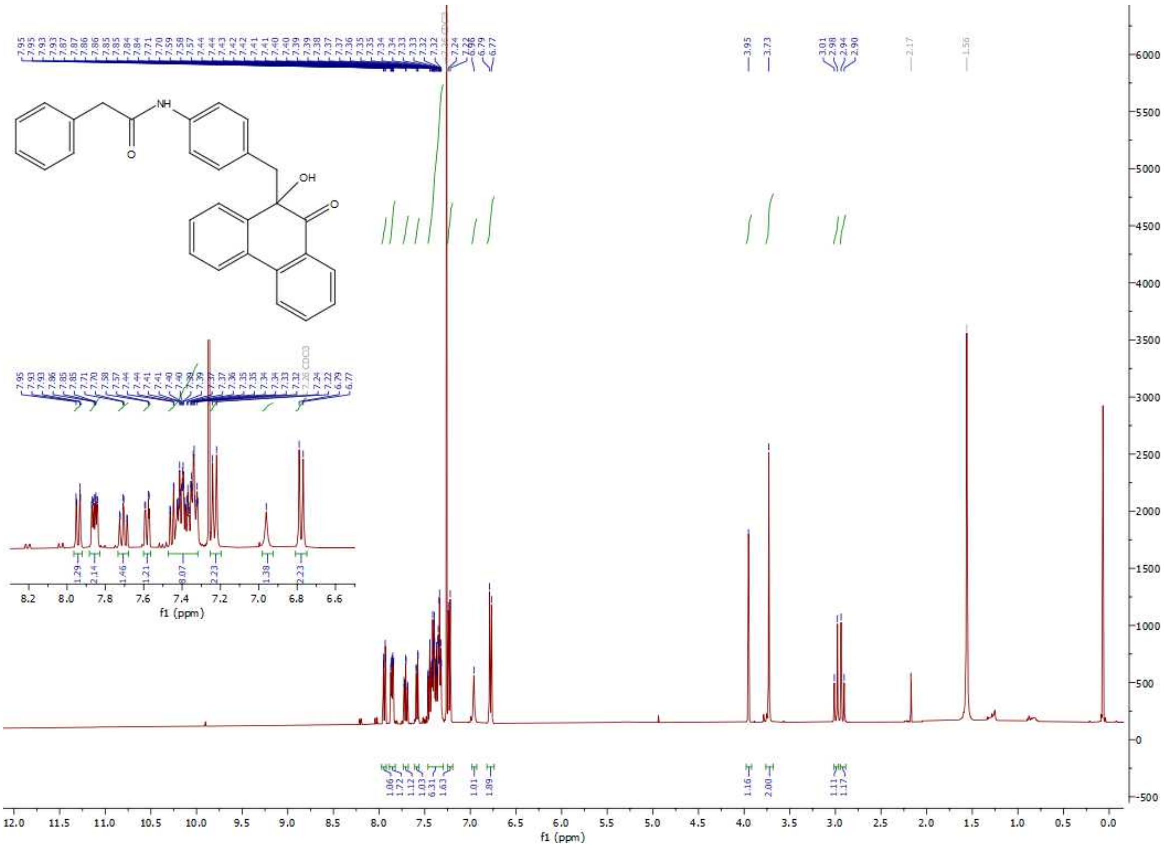

^{13}C NMR (100 MHz, $CDCl_3$) of 2-phenyl-acetyl-*para*-aminobenzyl phenanthrene-ketol **21**

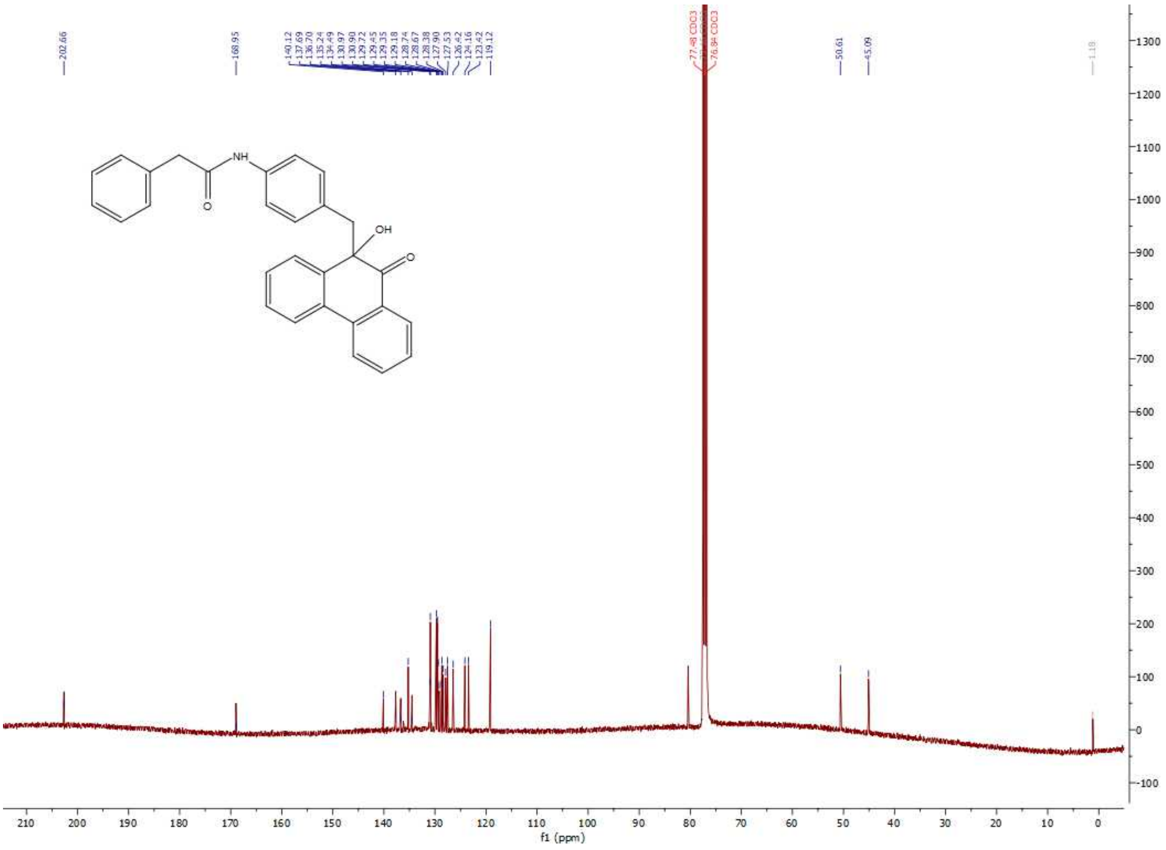

^{1}H NMR (500 MHz, $CDCl_3$) of 2-phenyl-acetyl-*para*-aminobenzyl β-lapa-ketol **22**

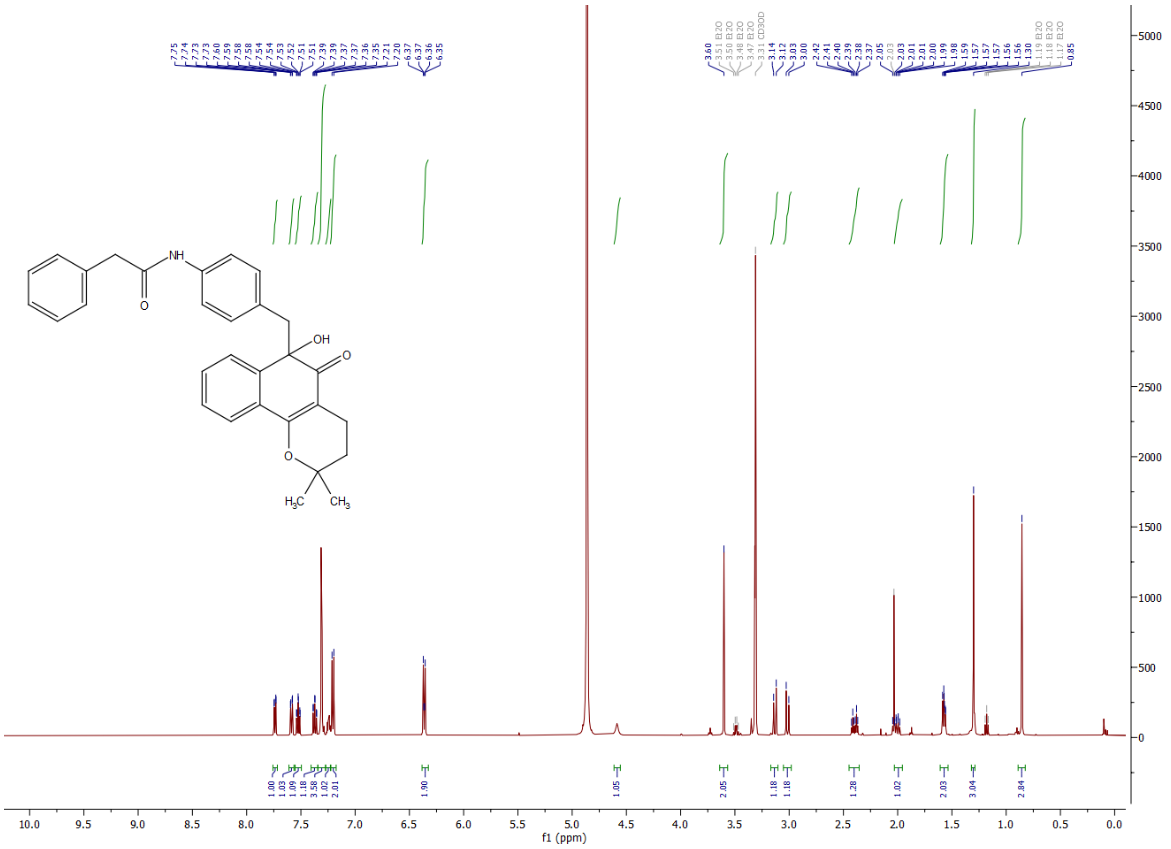

^{13}C NMR (126 MHz, $CDCl_3$) of 2-phenyl-acetyl-*para*-aminobenzyl β-lapa-ketol **22**

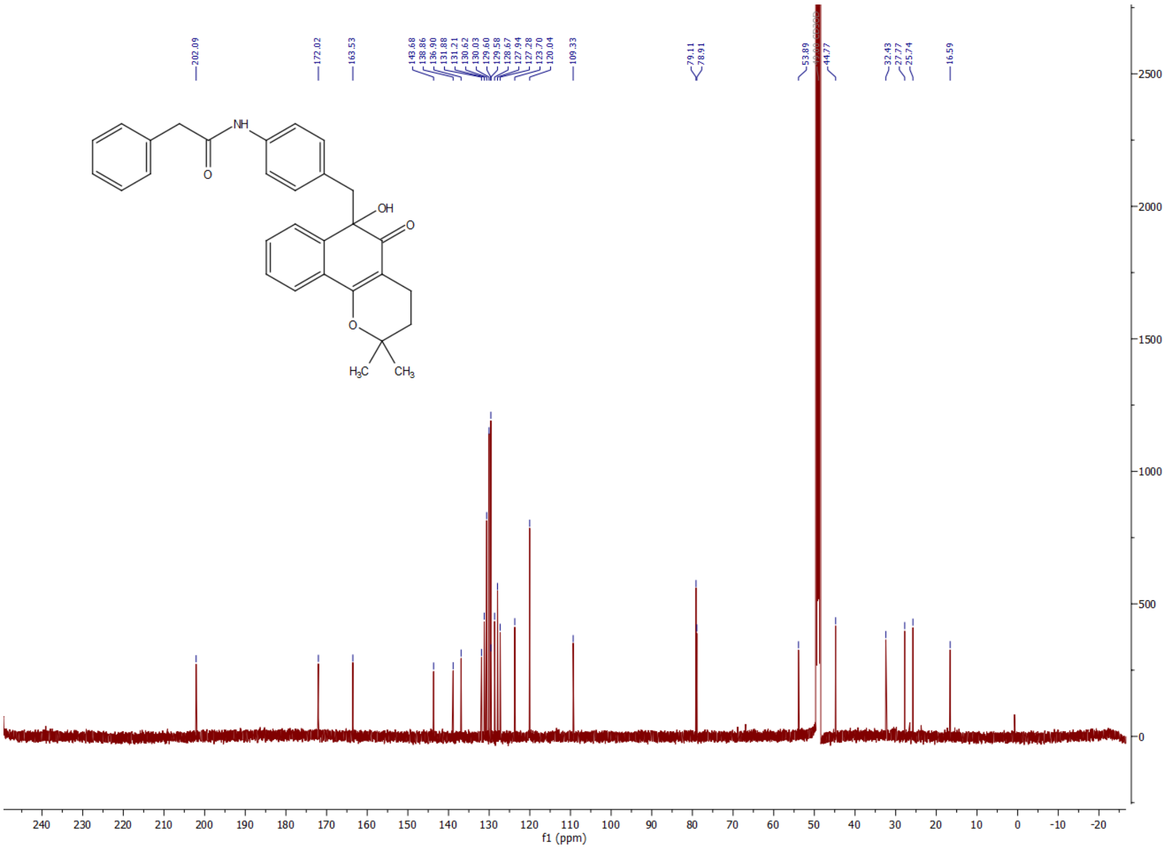

^{1}H NMR (500 MHz, $CDCl_3$) of 2-phenyl-acetyl-*para*-aminobenzyl dunni-ketol **23**

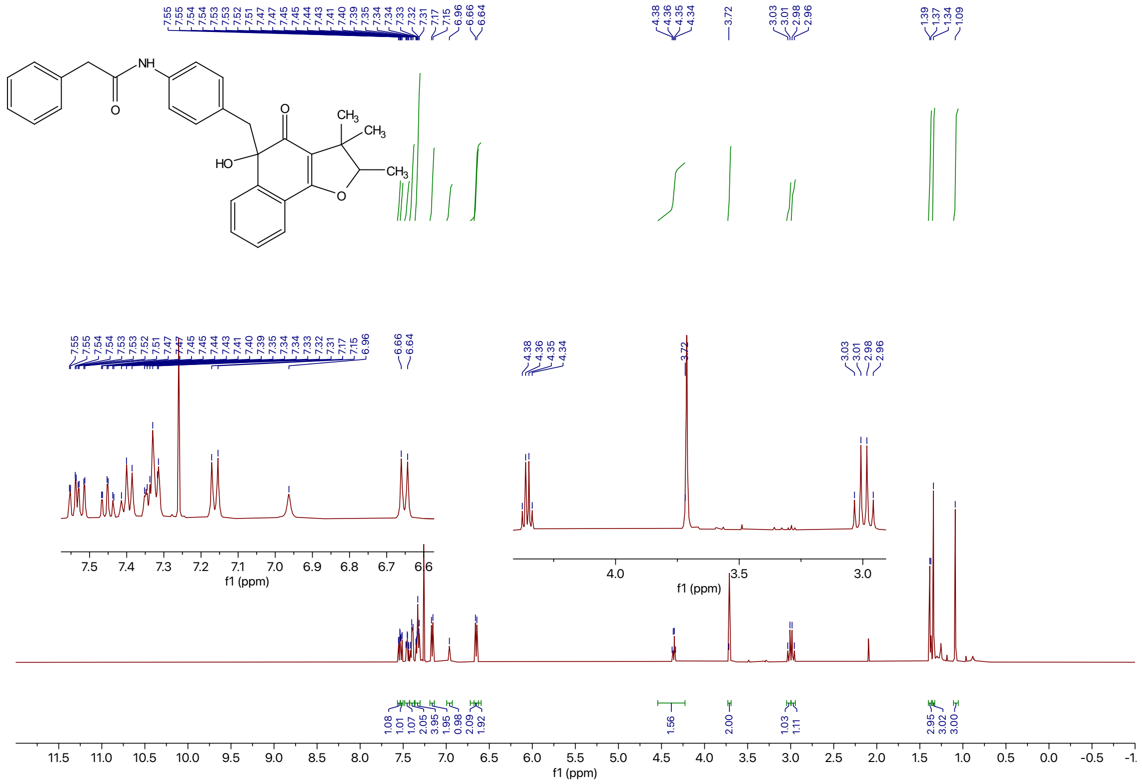

^{13}C NMR (126 MHz, $CDCl_3$) of 2-phenyl-acetyl-*para*-aminobenzyl dunni-ketol **23**

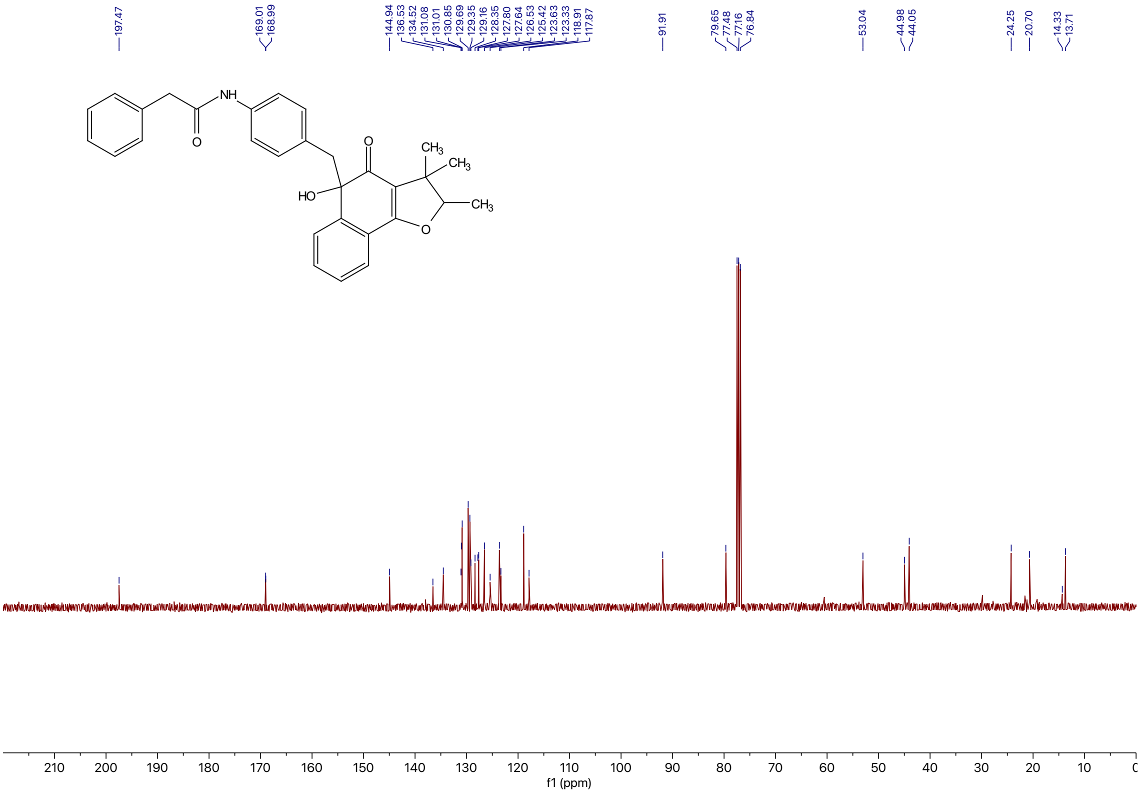

^{1}H NMR (400 MHz, $CDCl_3$) of benzyl β-lapa-ketol **24**

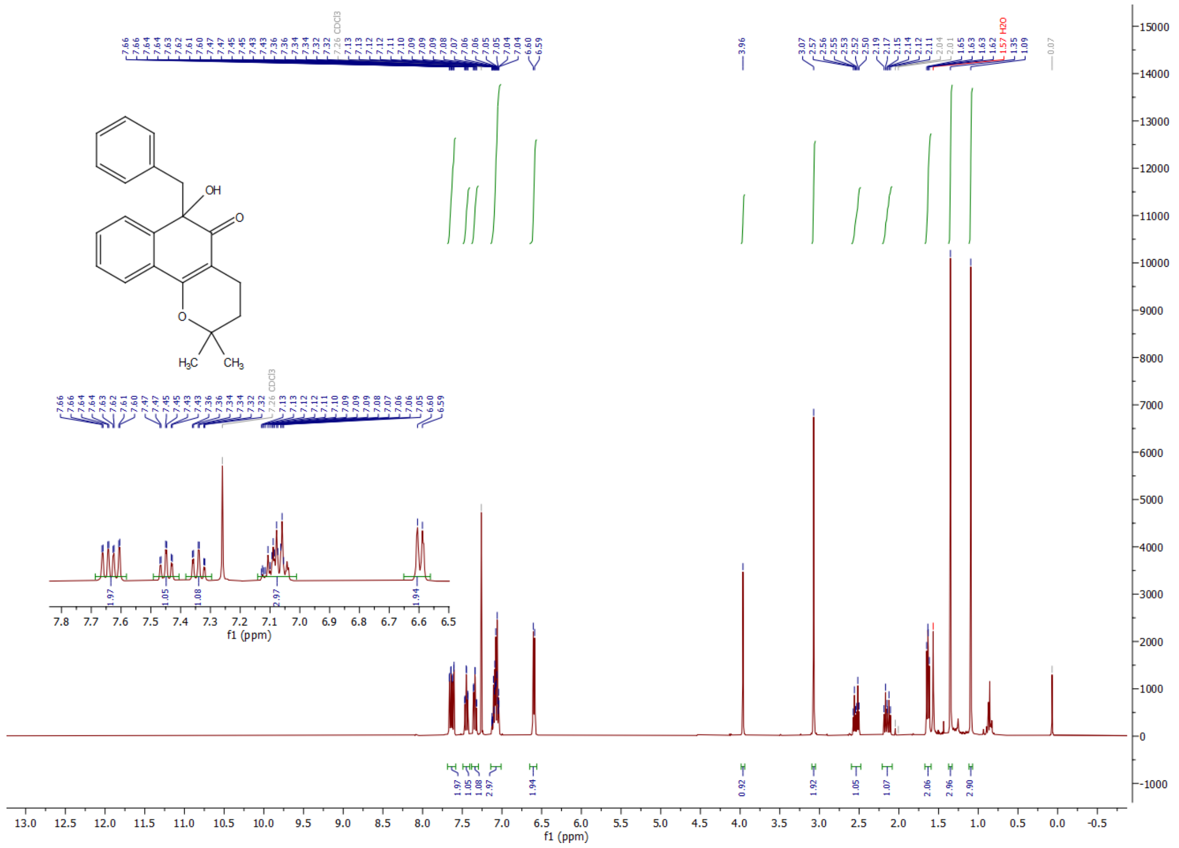

^{13}C NMR (100 MHz, $CDCl_3$) of benzyl β-lapa-ketol **24**

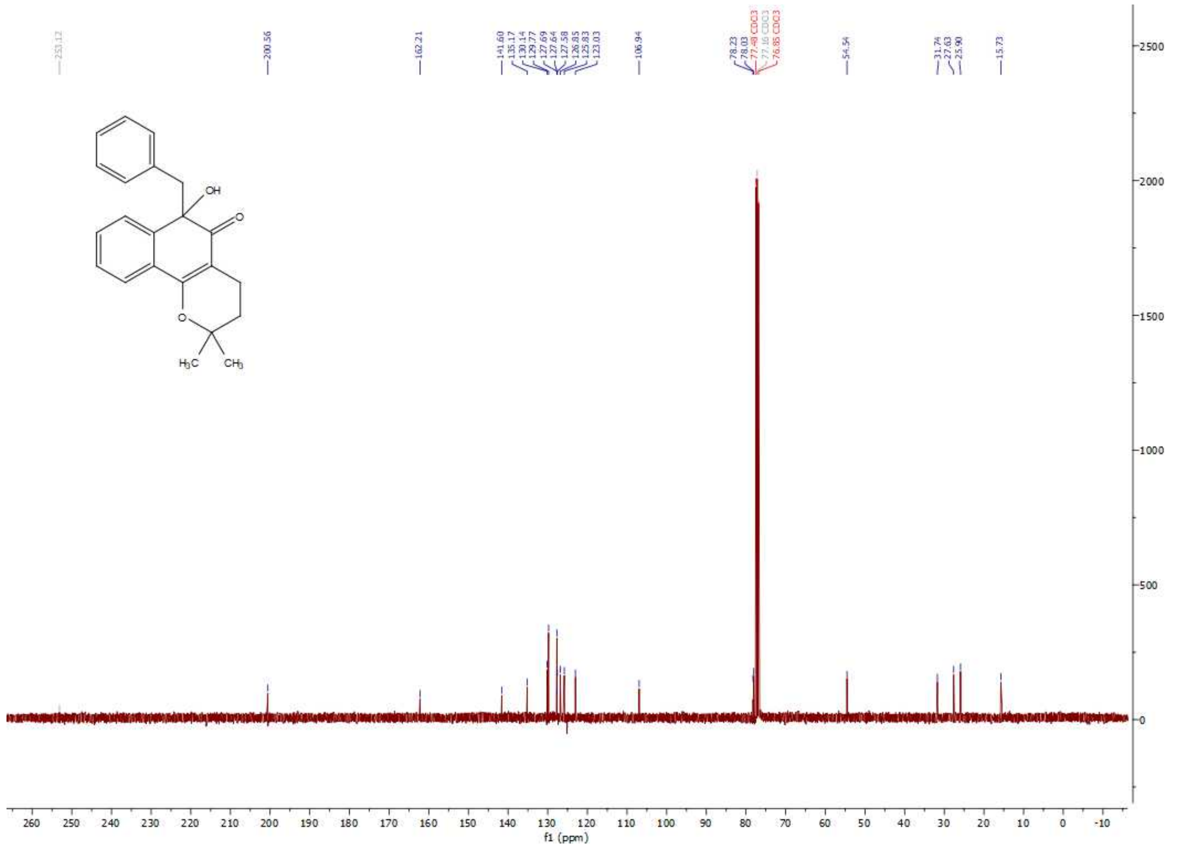

^{1}H NMR (400 MHz, $CDCl_3$) of β-lapa-hydro-1,4-dioxine **25**

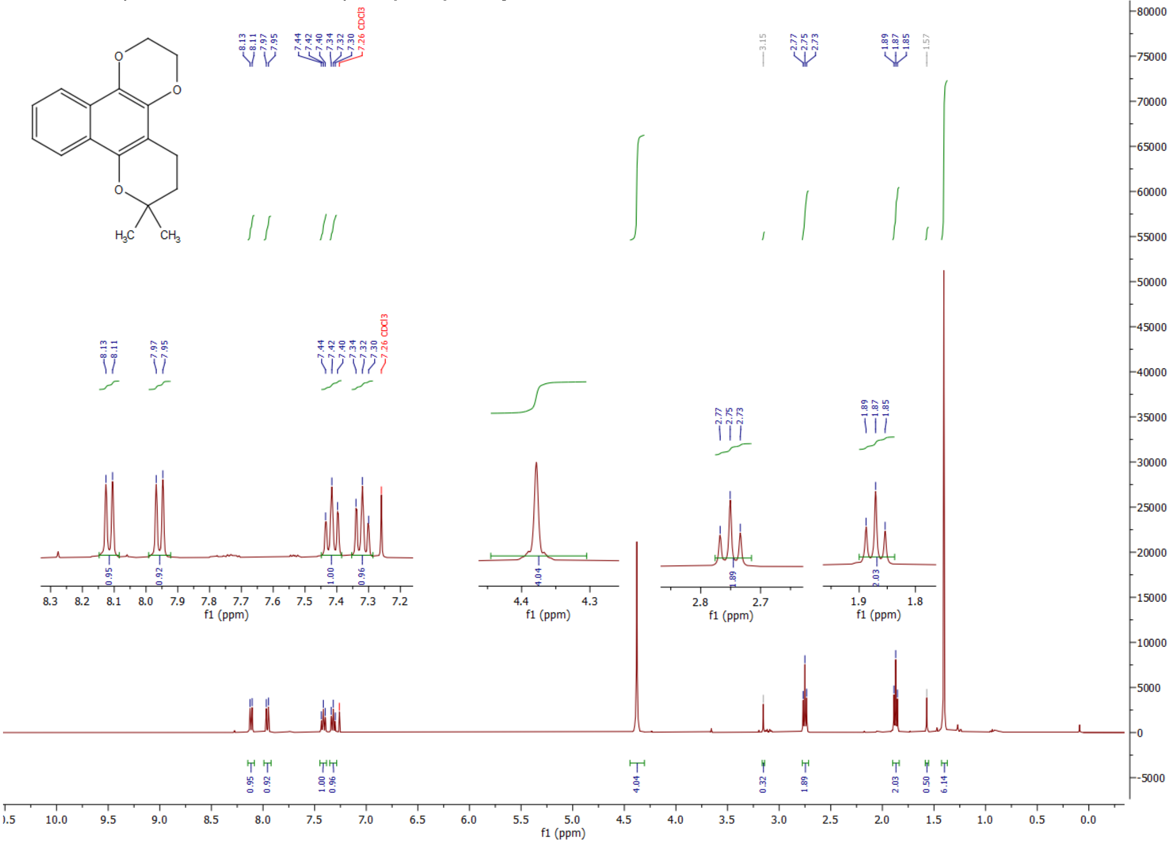

^{13}C NMR (100 MHz, $CDCl_3$) of β-lapa-hydro-1,4-dioxine **25**

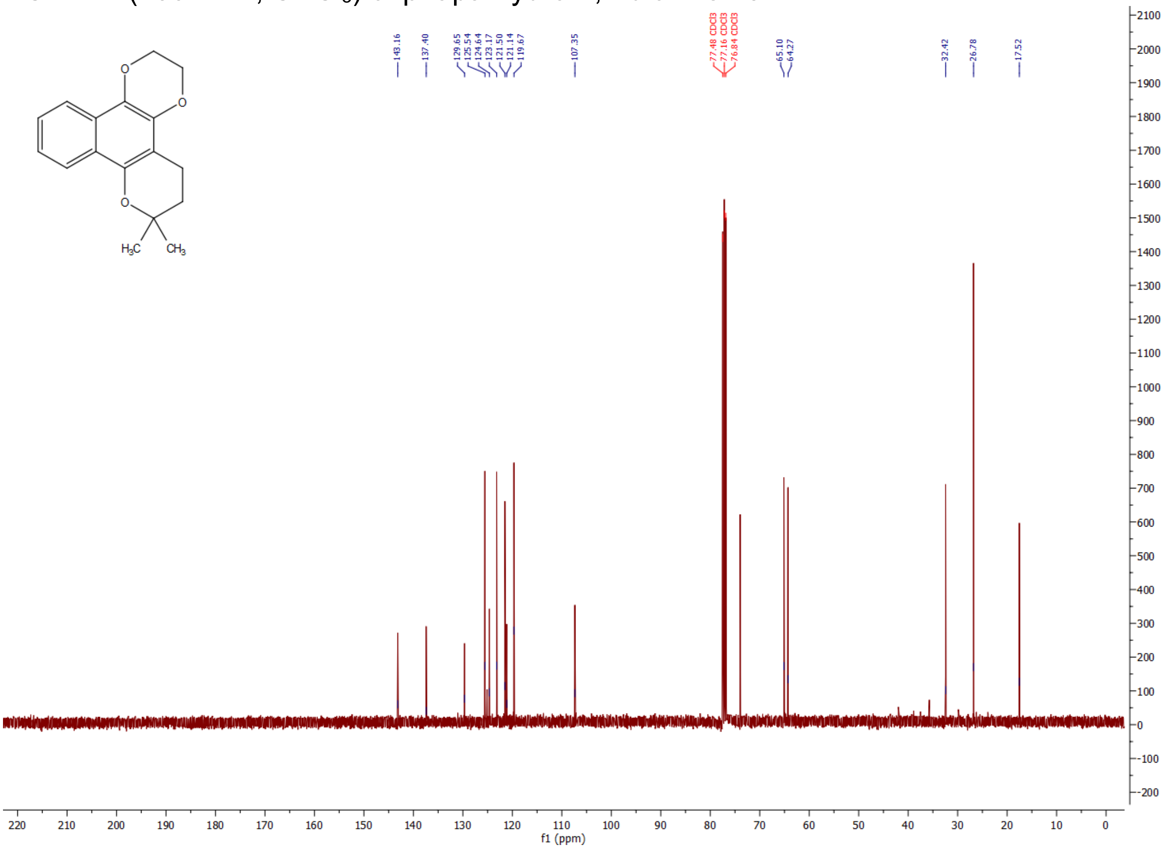

^{1}H NMR (400 MHz, DMSO-d6) of Boc-L-citrulline

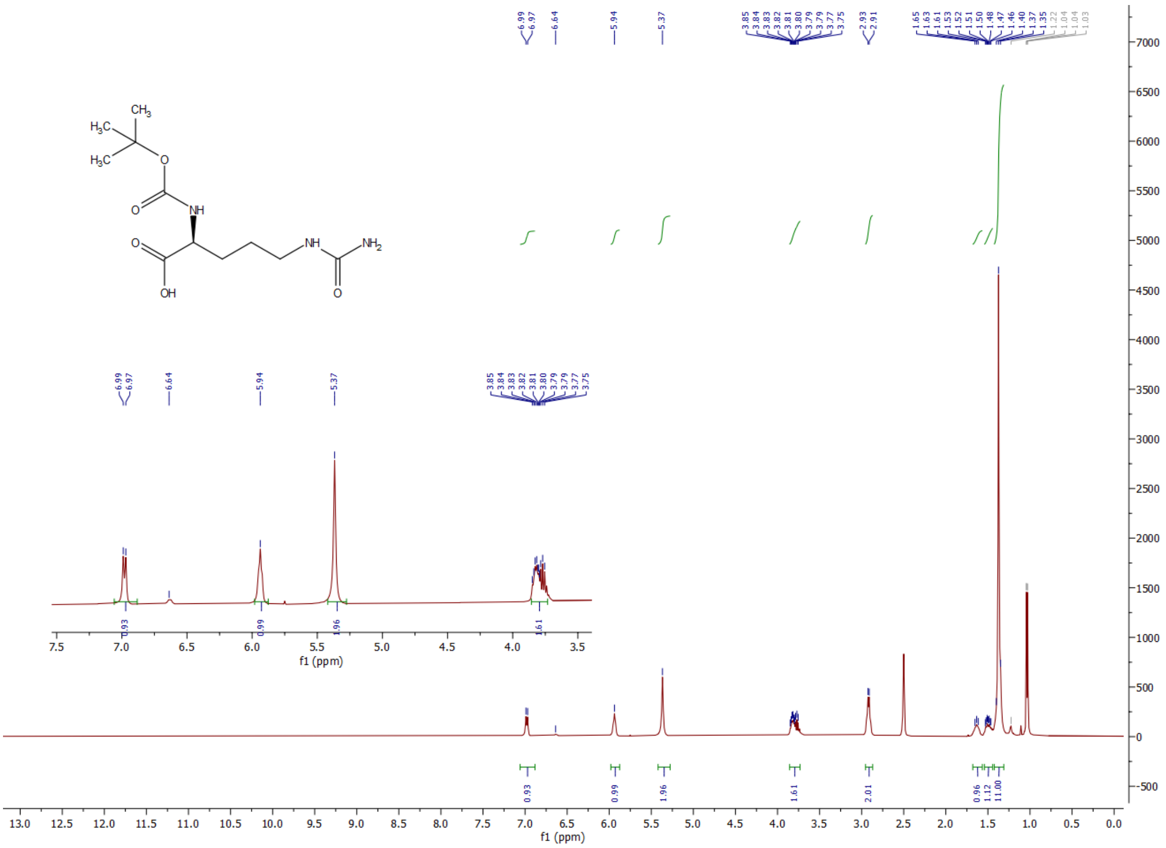

^{13}C NMR (400 MHz, DMSO-d6) of Boc-L-citrulline

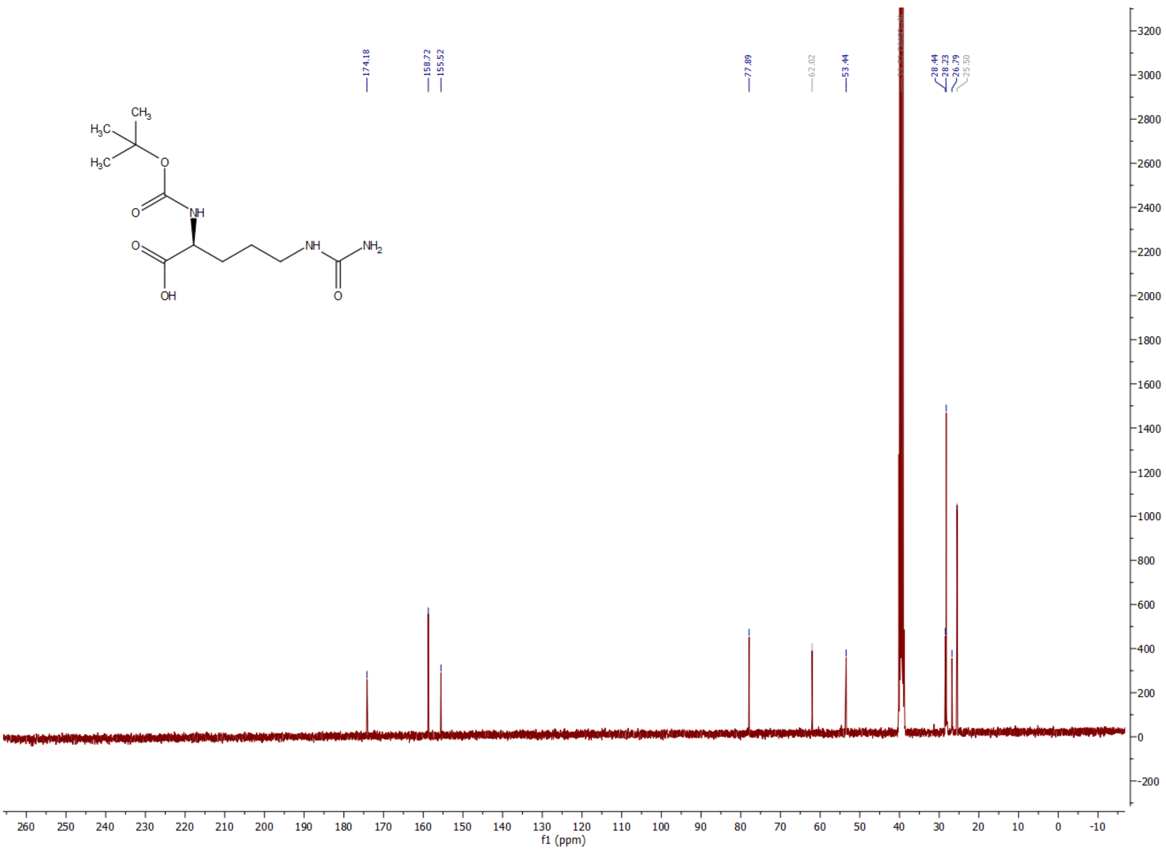

^{1}H NMR (400 MHz, $CDCl_3$) of perfluorophenyl ((benzyloxy)carbonyl)-L-valinate

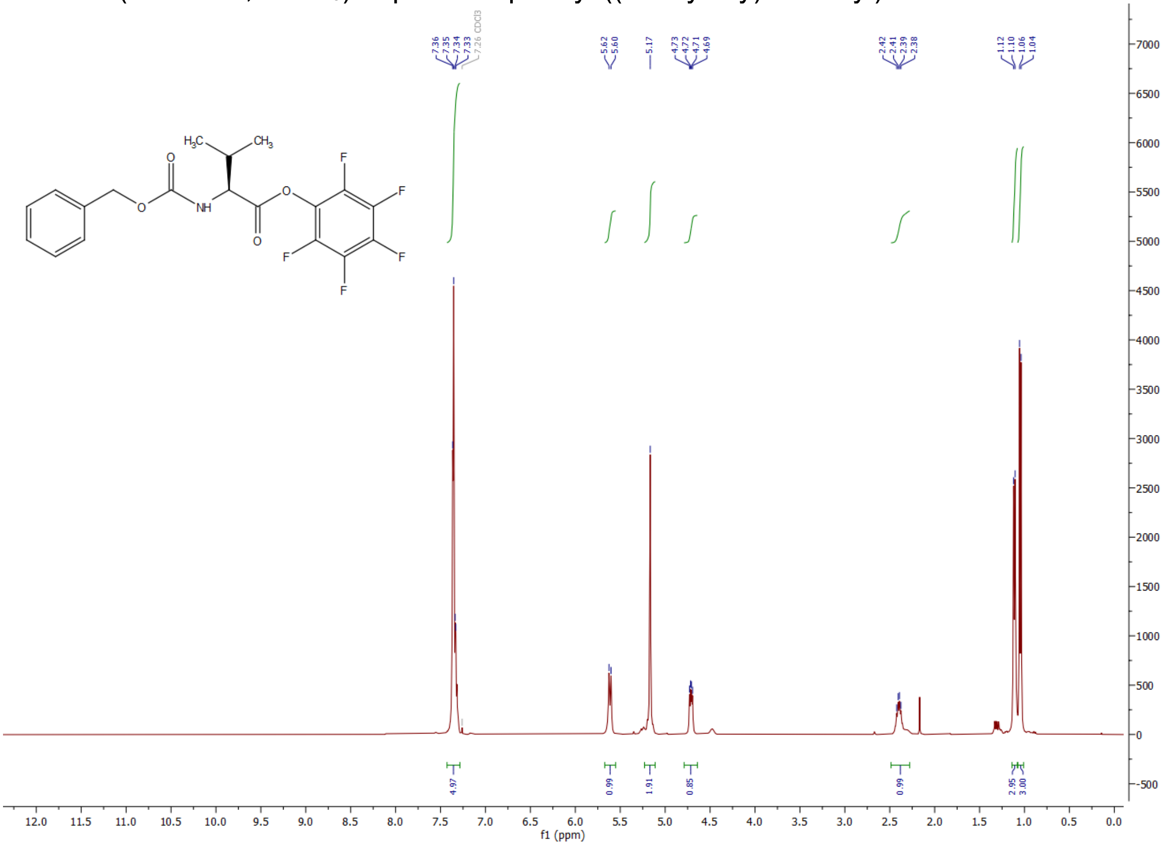

^{13}C NMR (100 MHz, $CDCl_3$) of perfluorophenyl ((benzyloxy)carbonyl)-L-valinate

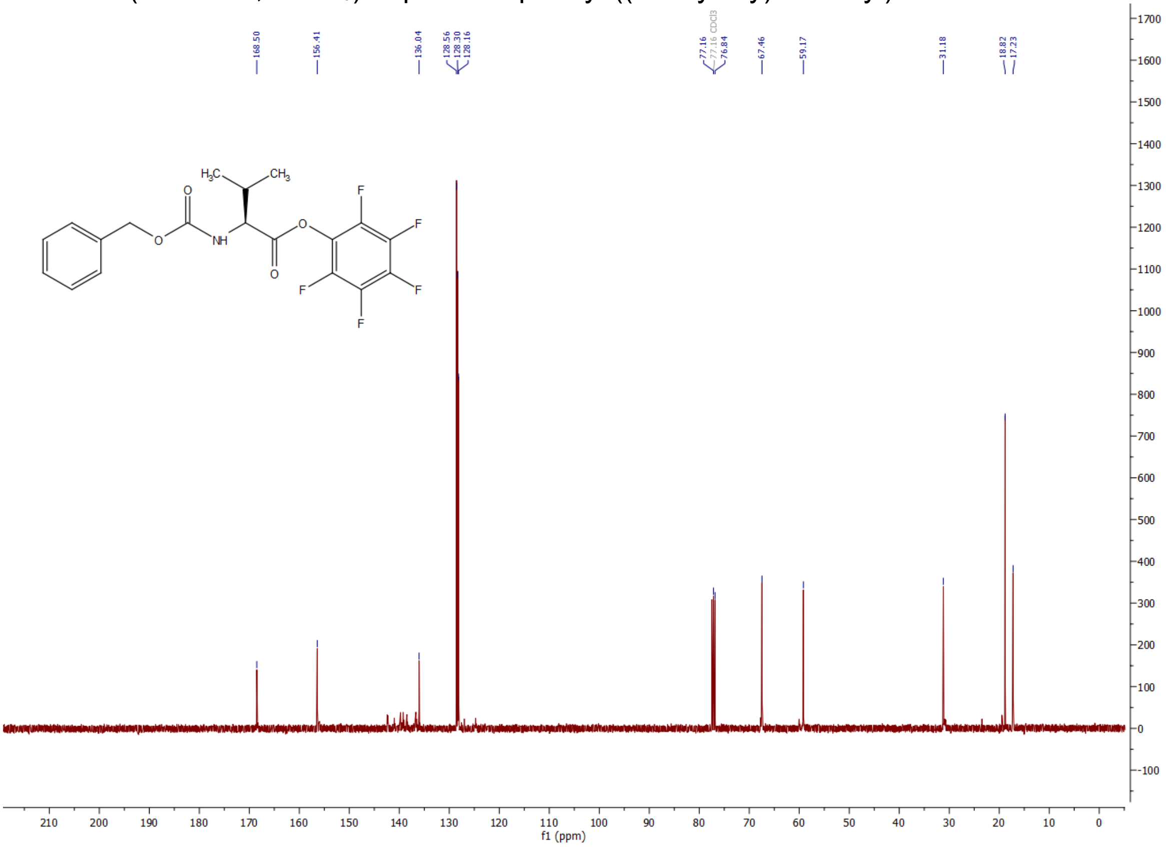

^{1}H NMR (400 MHz, $CDCl_3$) of perfluorophenyl ((benzyloxy)carbonyl)-L-phenylalaninate

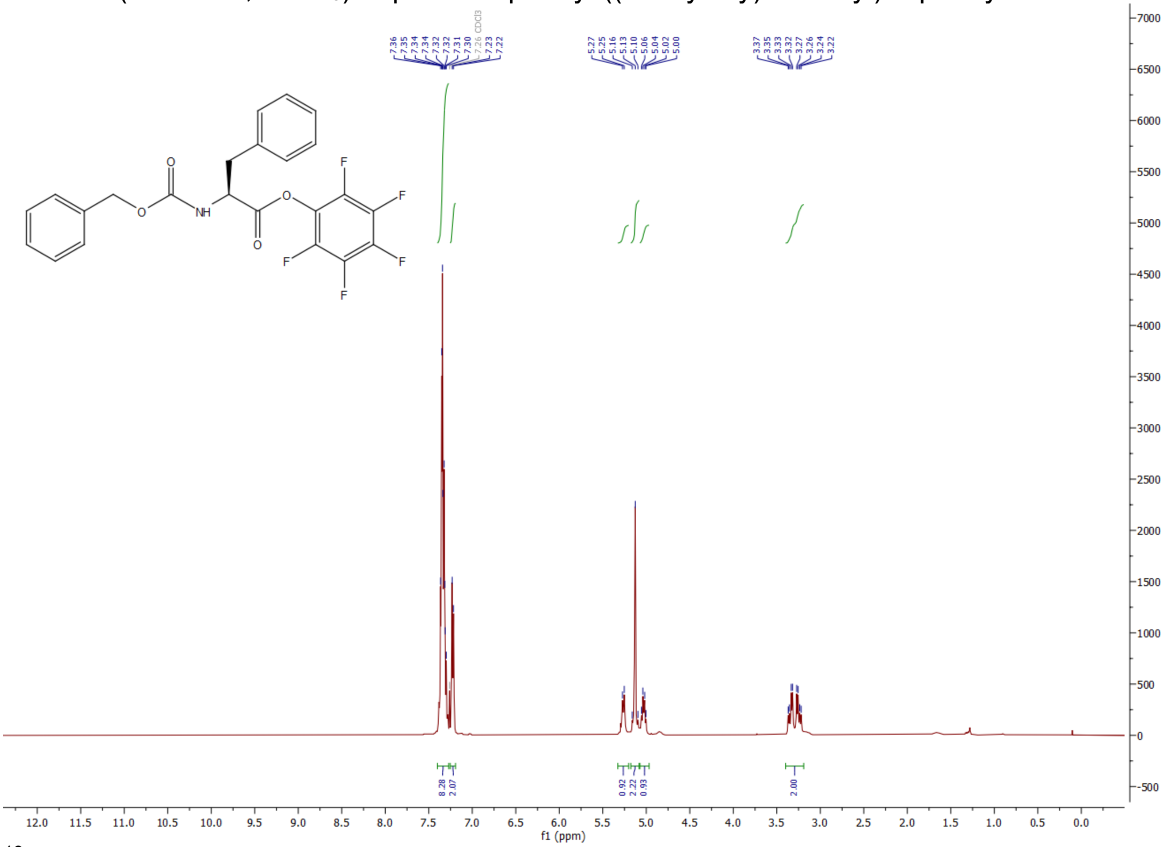

^{13}C NMR (100 MHz, $CDCl_3$) of perfluorophenyl ((benzyloxy)carbonyl)-L-phenylalaninate

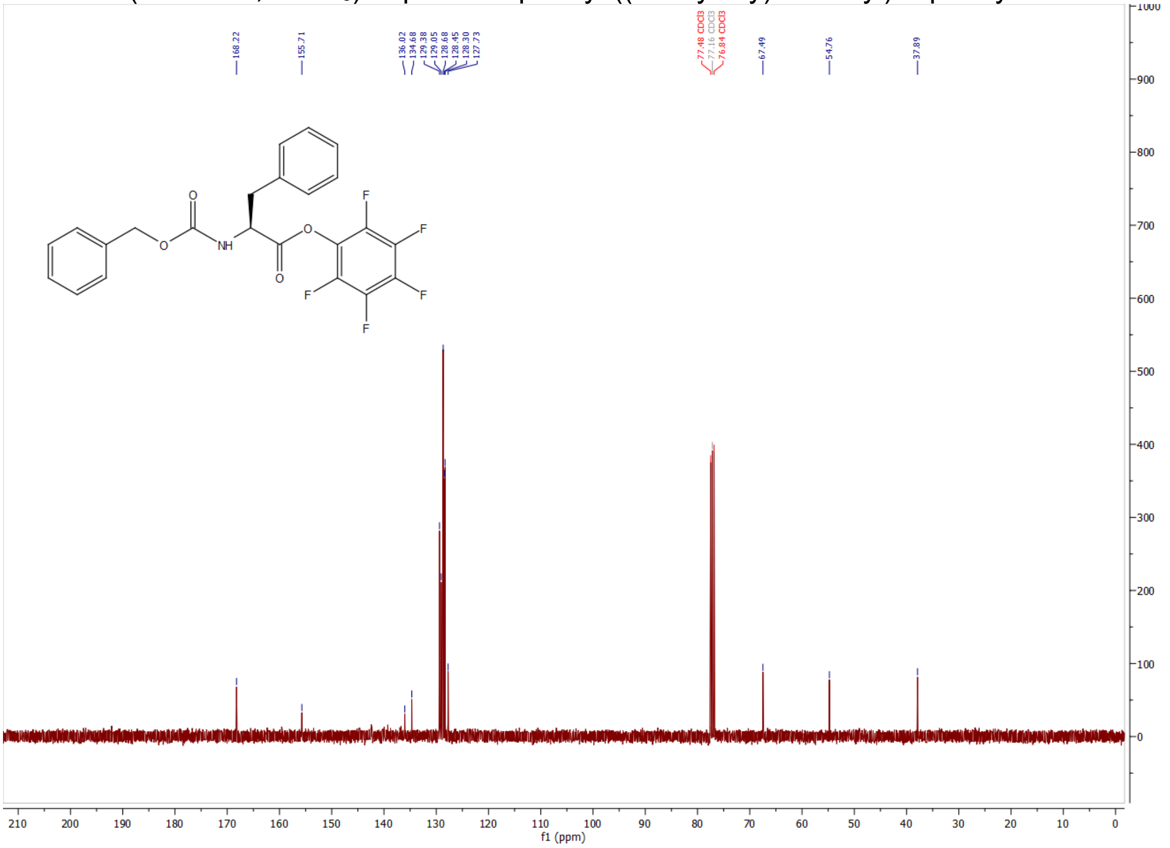

^{1}H NMR (600 MHz, $CDCl_3$) of perfluorophenyl (((9H-fluoren-9-yl)methoxy)carbonyl)-L-valinate

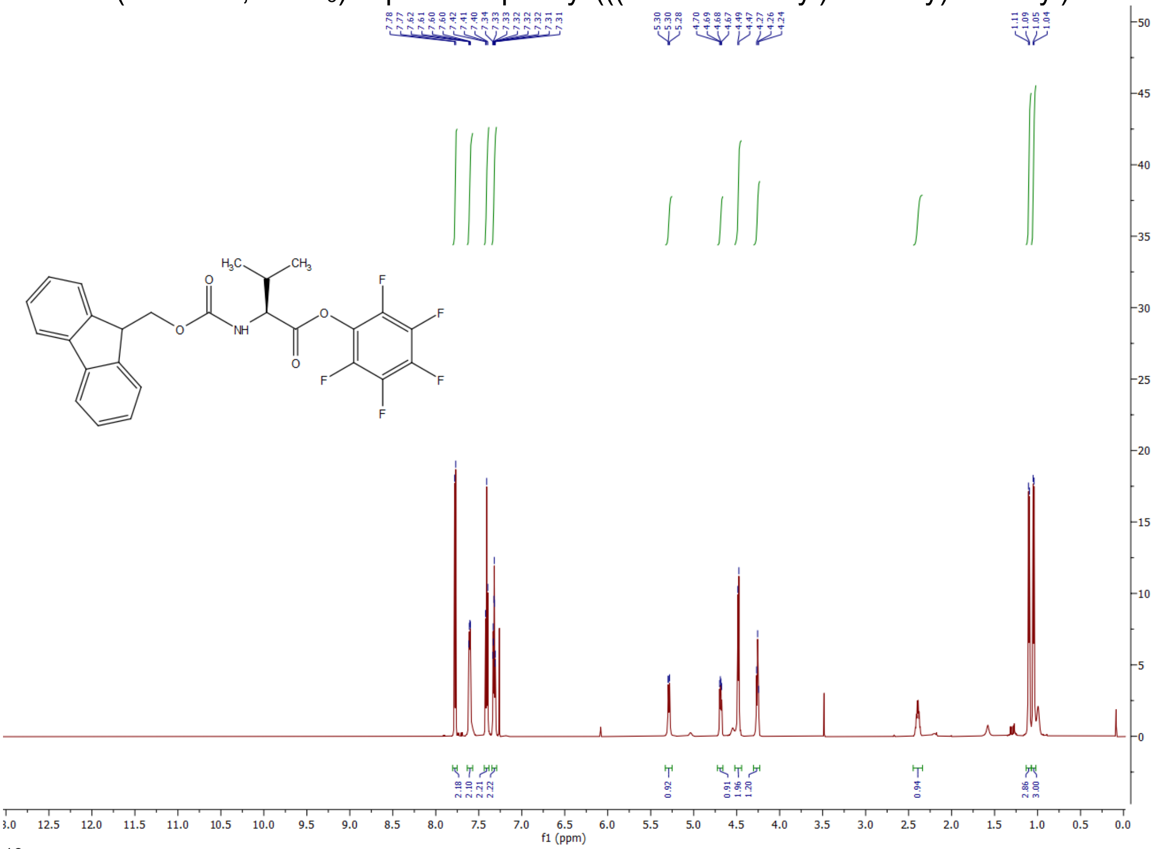

^{13}C NMR (151 MHz, $CDCl_3$) of perfluorophenyl (((9H-fluoren-9-yl)methoxy)carbonyl)-L-valinate

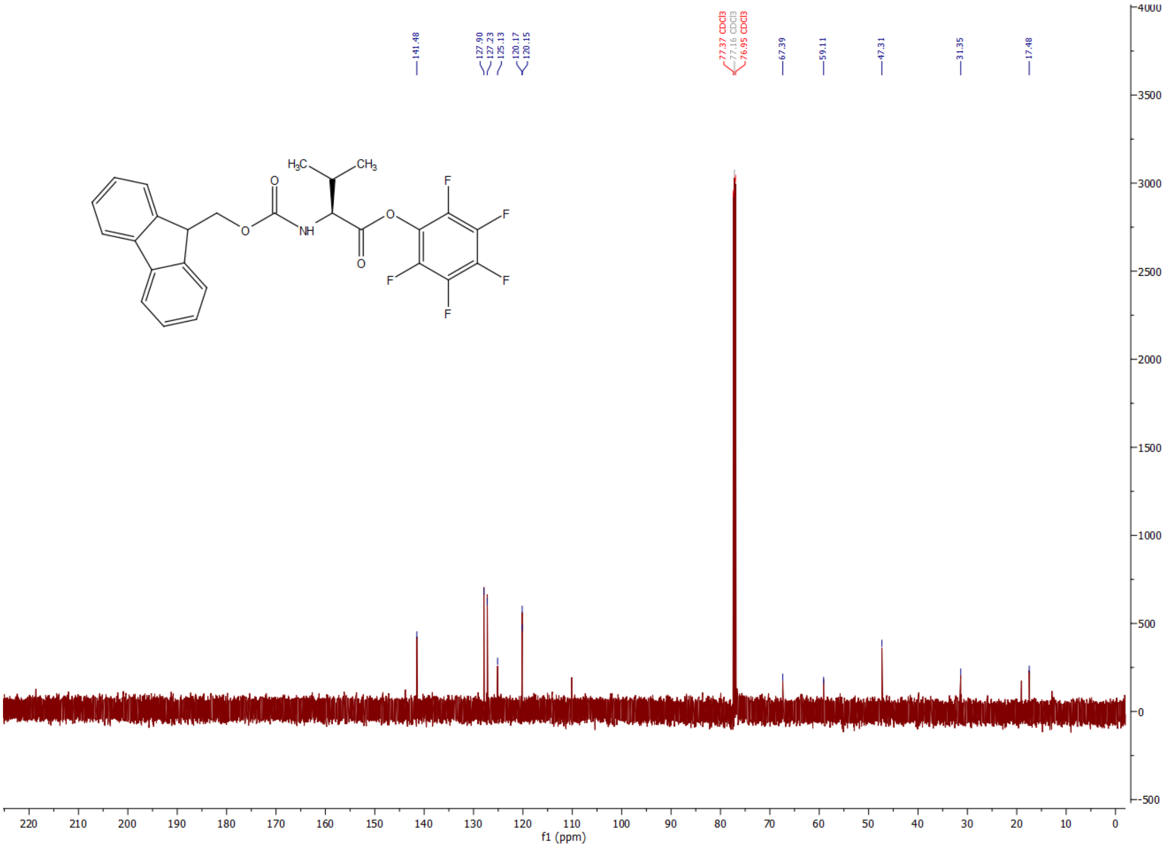

^{1}H NMR (600 MHz, $CDCl_3$) of perfluorophenyl (*E*)-4-oxo-4-phenylbut-2-enoate

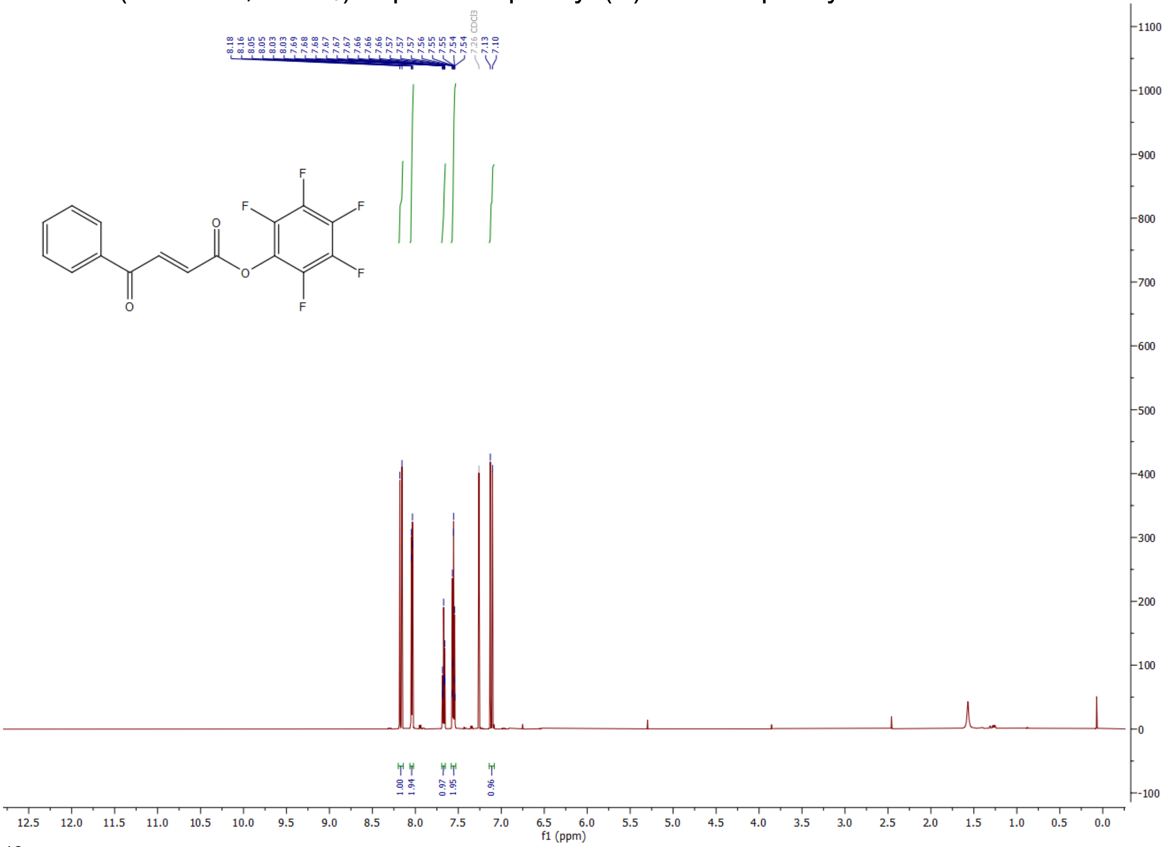

^{13}C NMR (151 MHz, $CDCl_3$) of perfluorophenyl (*E*)-4-oxo-4-phenylbut-2-enoate

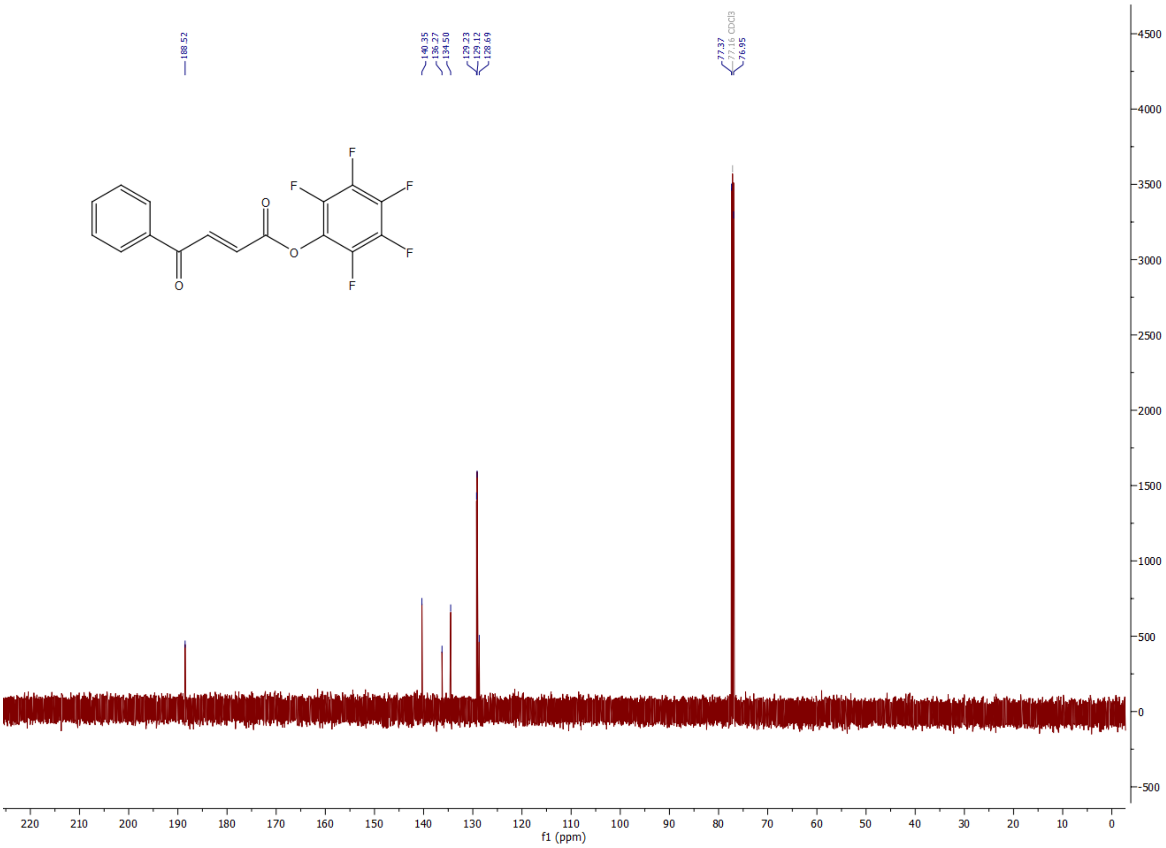

^{1}H NMR (500 MHz, DMSO-d$_6$) of Boc-L-Cit-*para*-aminobenzyl β-lapa-ketol **S4**

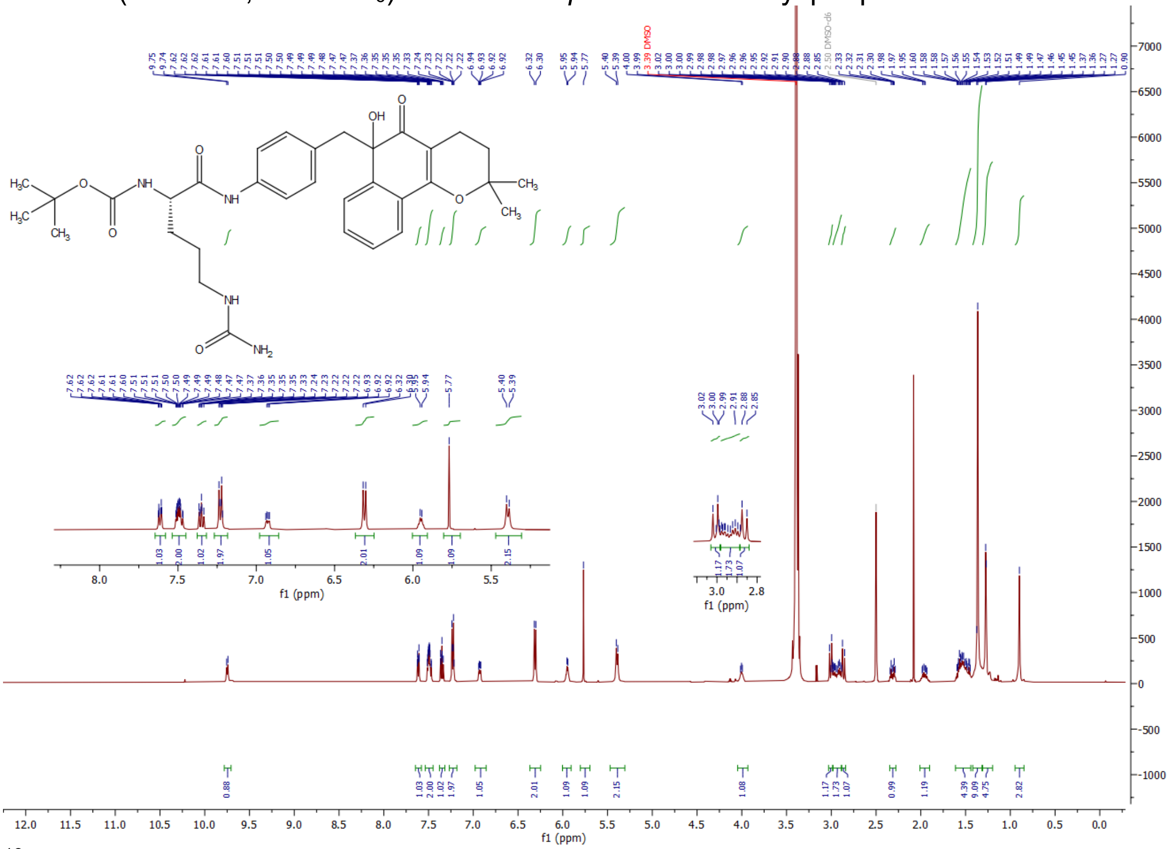

^{13}C NMR (126 MHz, DMSO-d$_6$) of Boc-L-Cit-*para*-aminobenzyl β-lapa-ketol **S4**

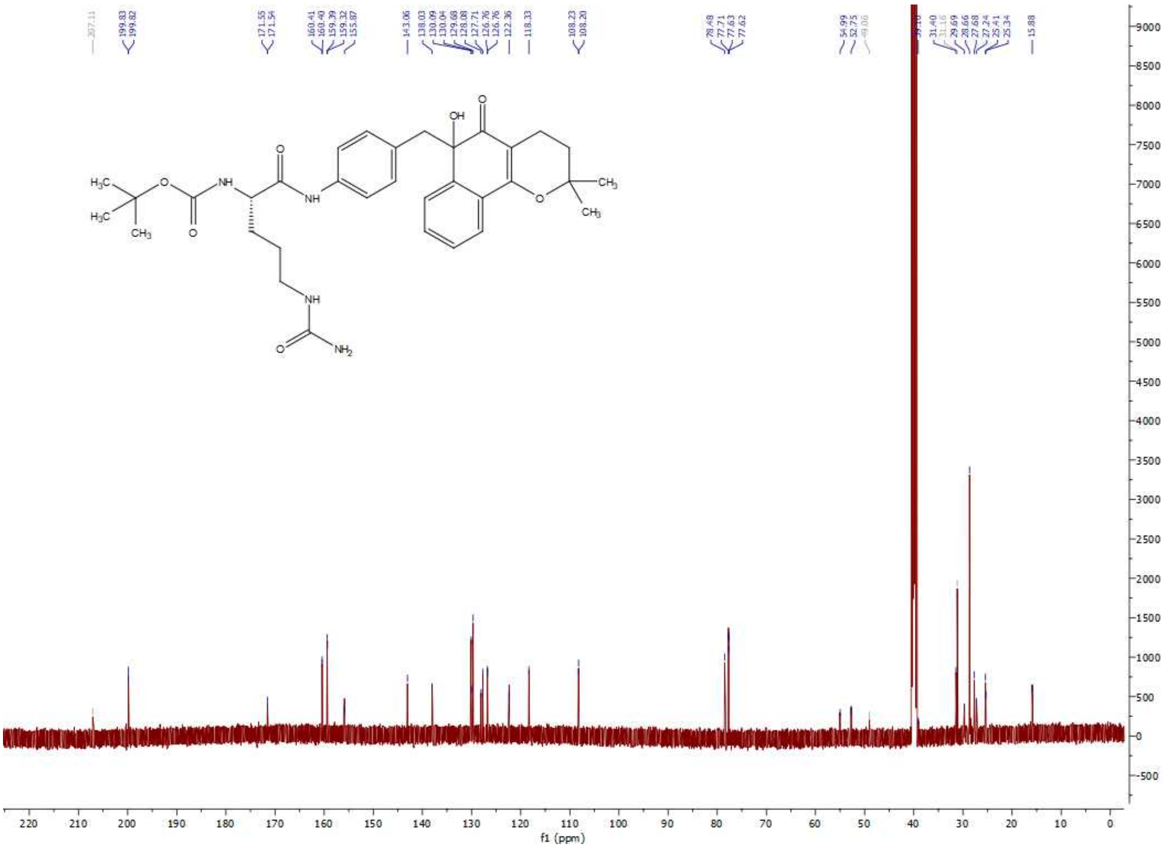

^{1}H NMR (400 MHz, MeOD) of Boc-L-Ala-*para*-aminobenzyl β-lapa-ketol **S5**

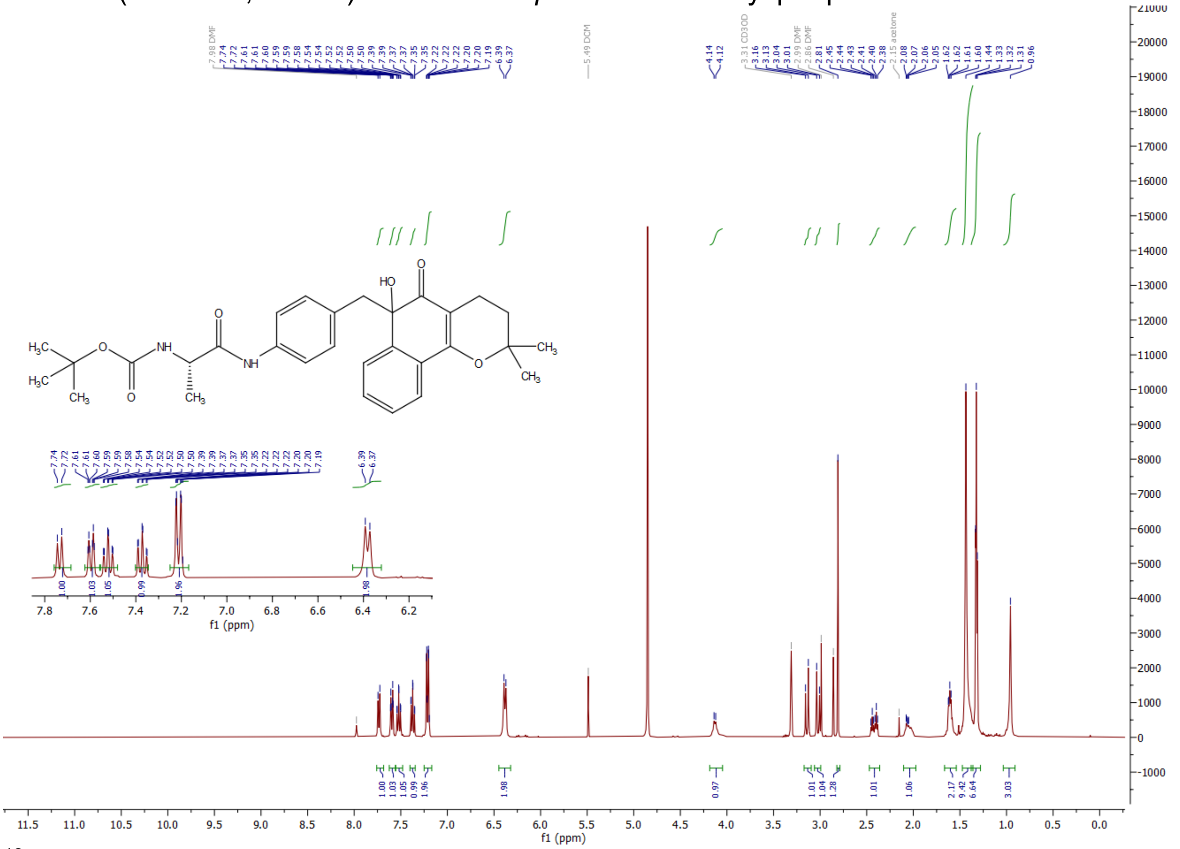

^{13}C NMR (100 MHz, MeOD) of Boc-L-Ala-*para*-aminobenzyl β-lapa-ketol **S5**

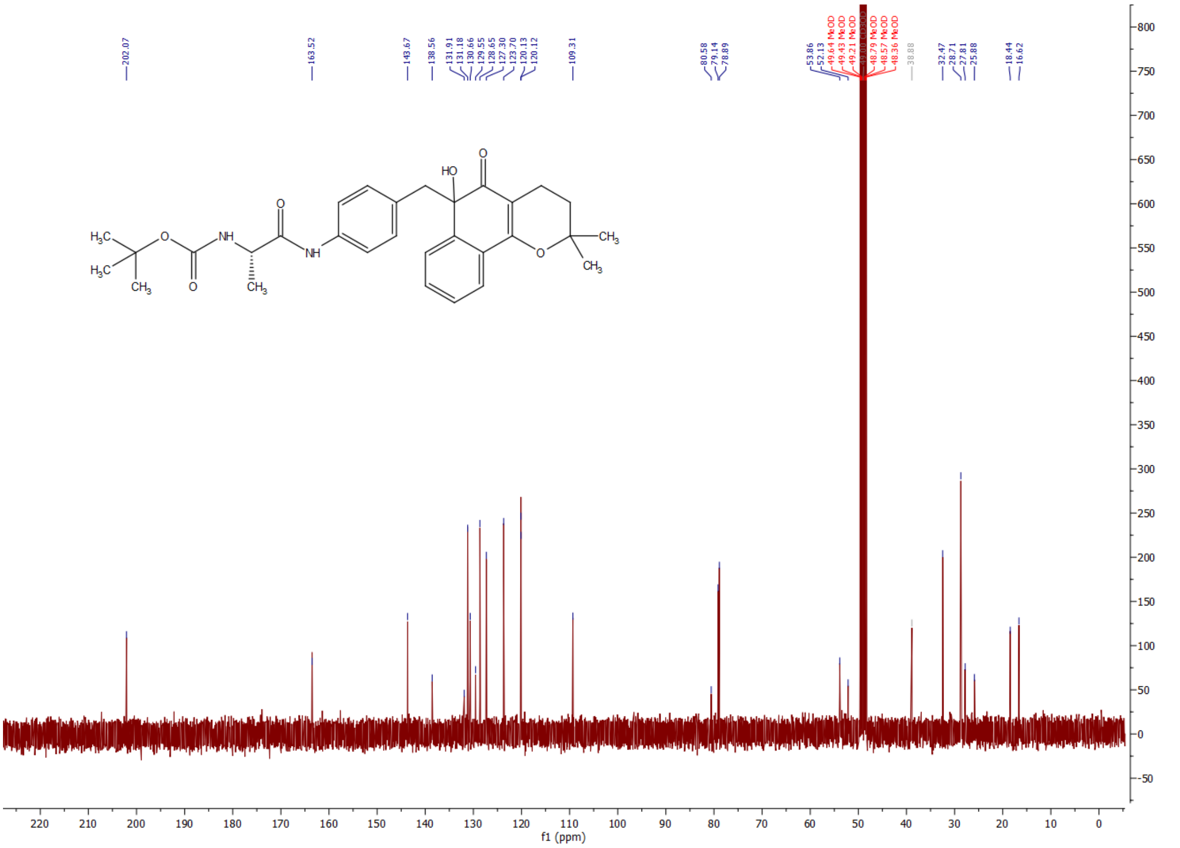

^{1}H NMR (400 MHz, MeOD) of Cbz-Val-Cit-*para*-aminobenzyl β-lapa-ketol **26**

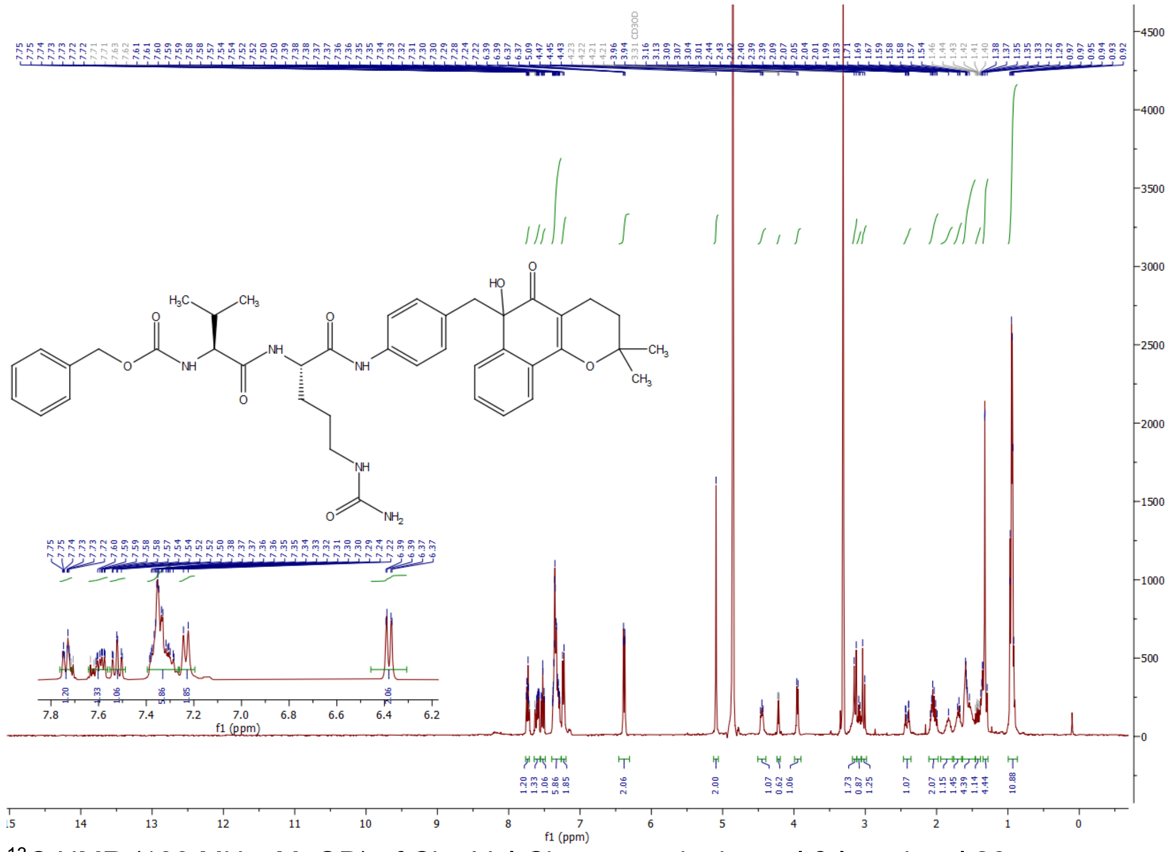

^{13}C NMR (100 MHz, MeOD) of Cbz-Val-Cit-*para*-aminobenzyl β-lapa-ketol **26**

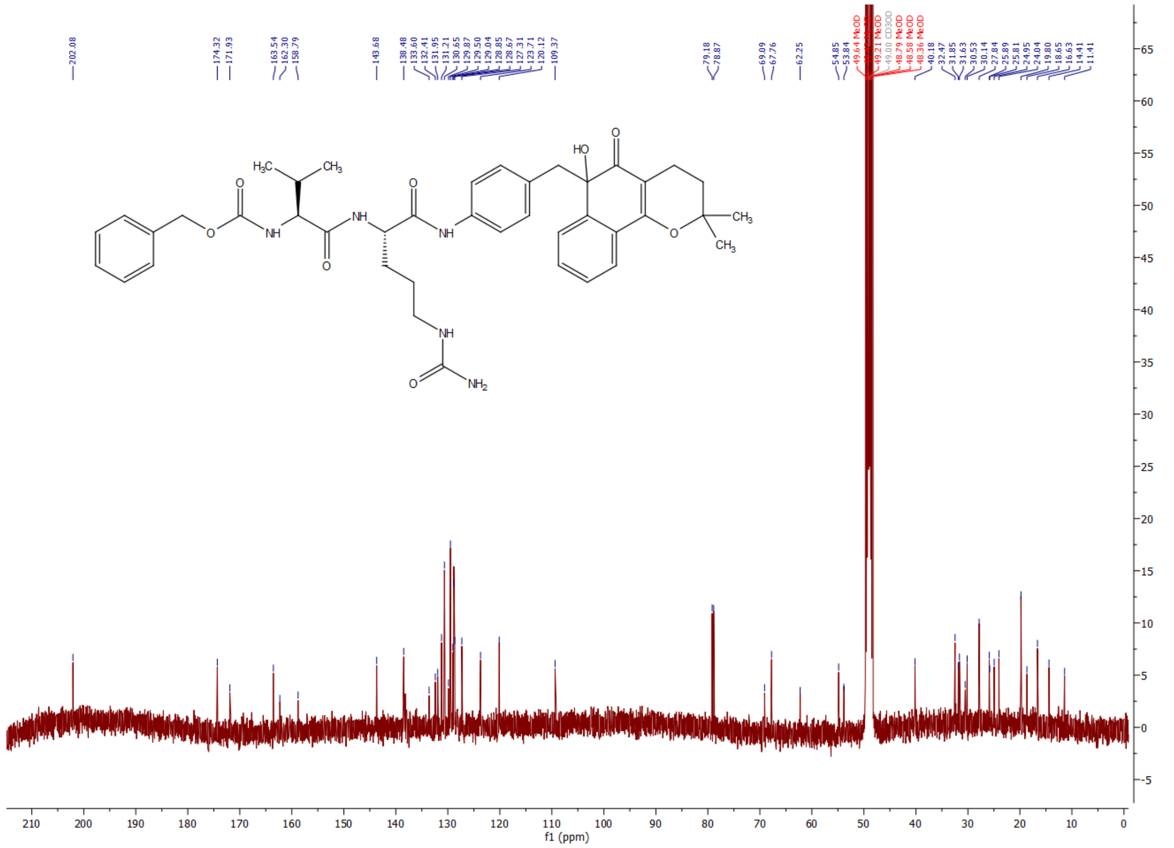

^{1}H NMR (600 MHz, MeOD) of Cbz-Val-Ala-*para*-aminobenzyl β-lapa-ketol **27**

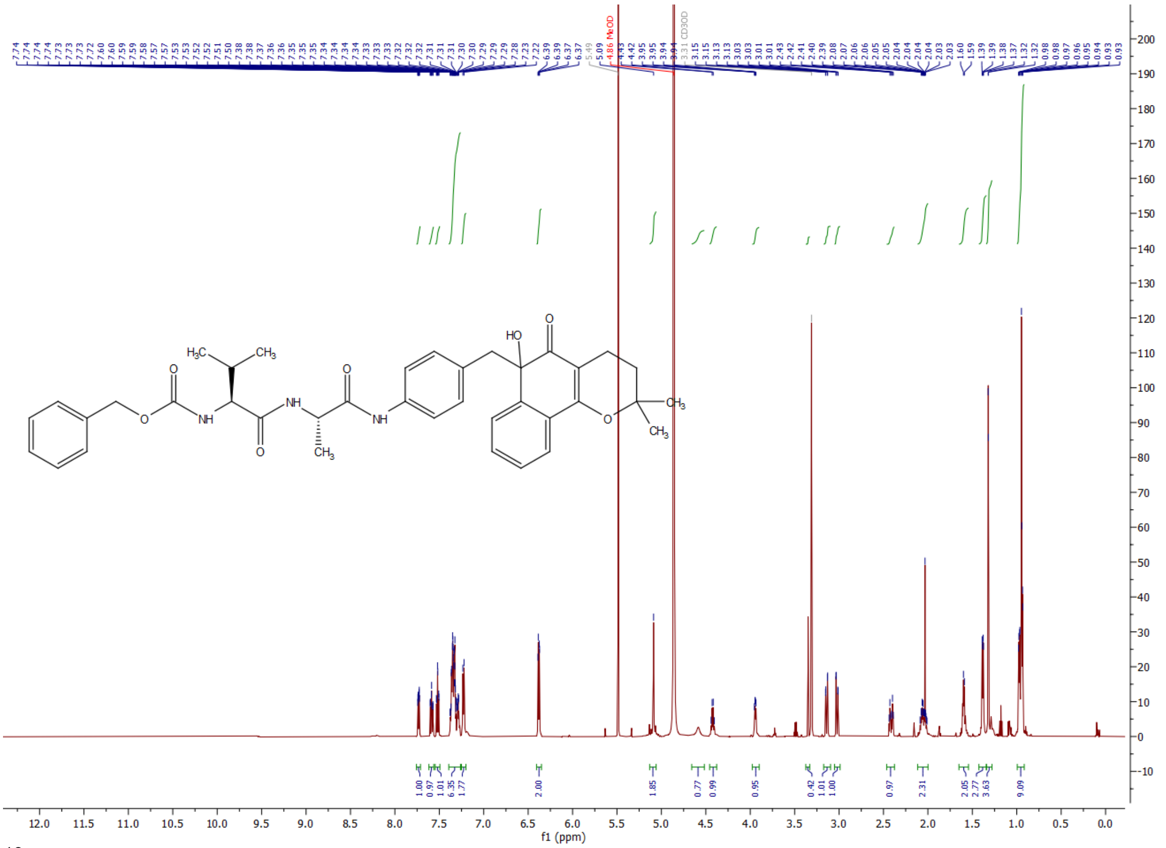

^{13}C NMR (126 MHz, MeOD) of Cbz-Val-Ala-*para*-aminobenzyl β-lapa-ketol **27**

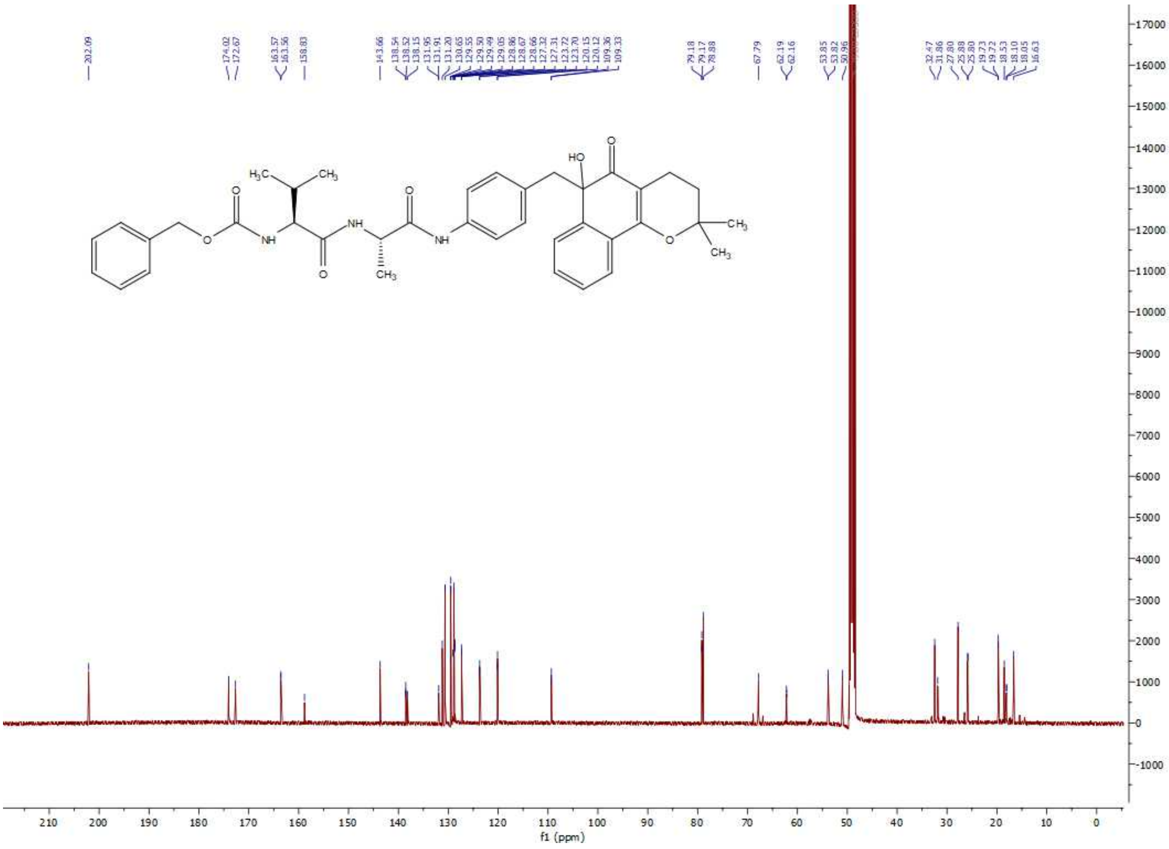

^{1}H NMR (400 MHz, MeOD) of Cbz-Phe-Cit-*para*-aminobenzyl β-lapa-ketol **28**

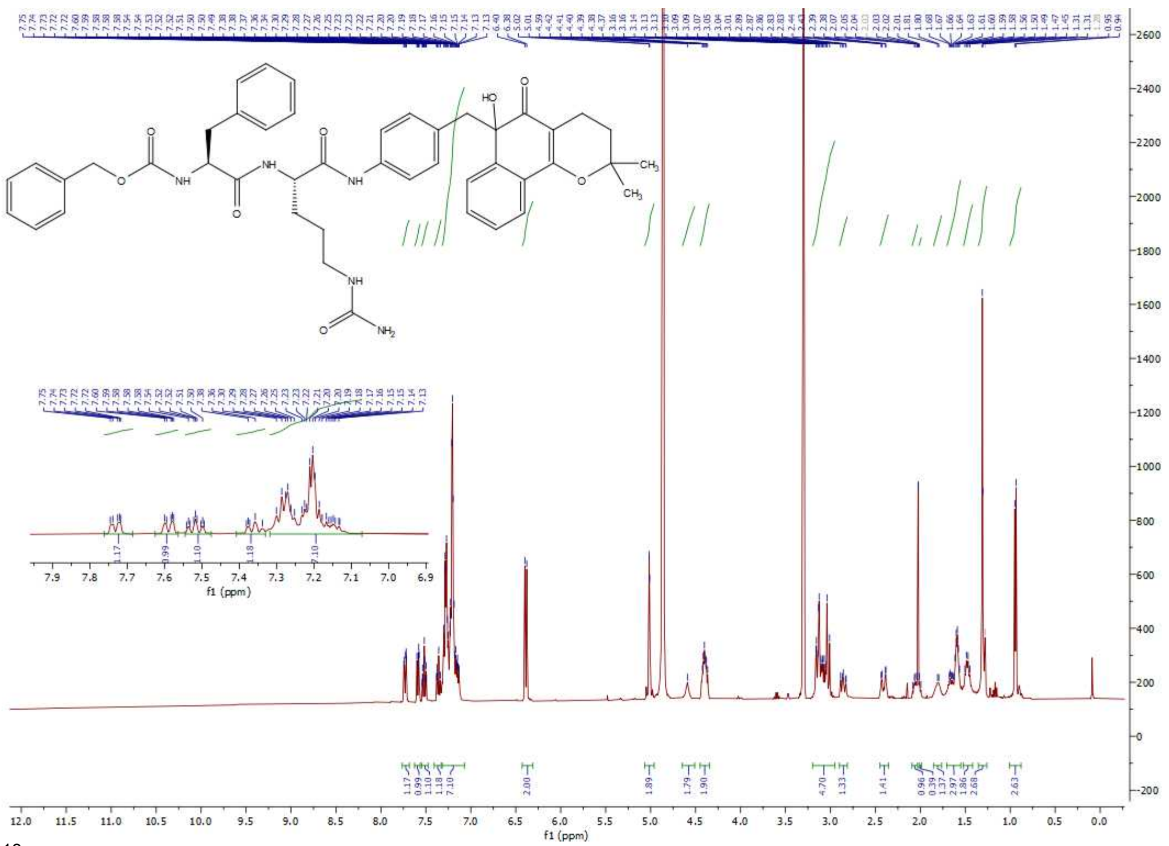

^{13}C NMR (100 MHz, MeOD) of Cbz-Phe-Cit-*para*-aminobenzyl β-lapa-ketol **28**

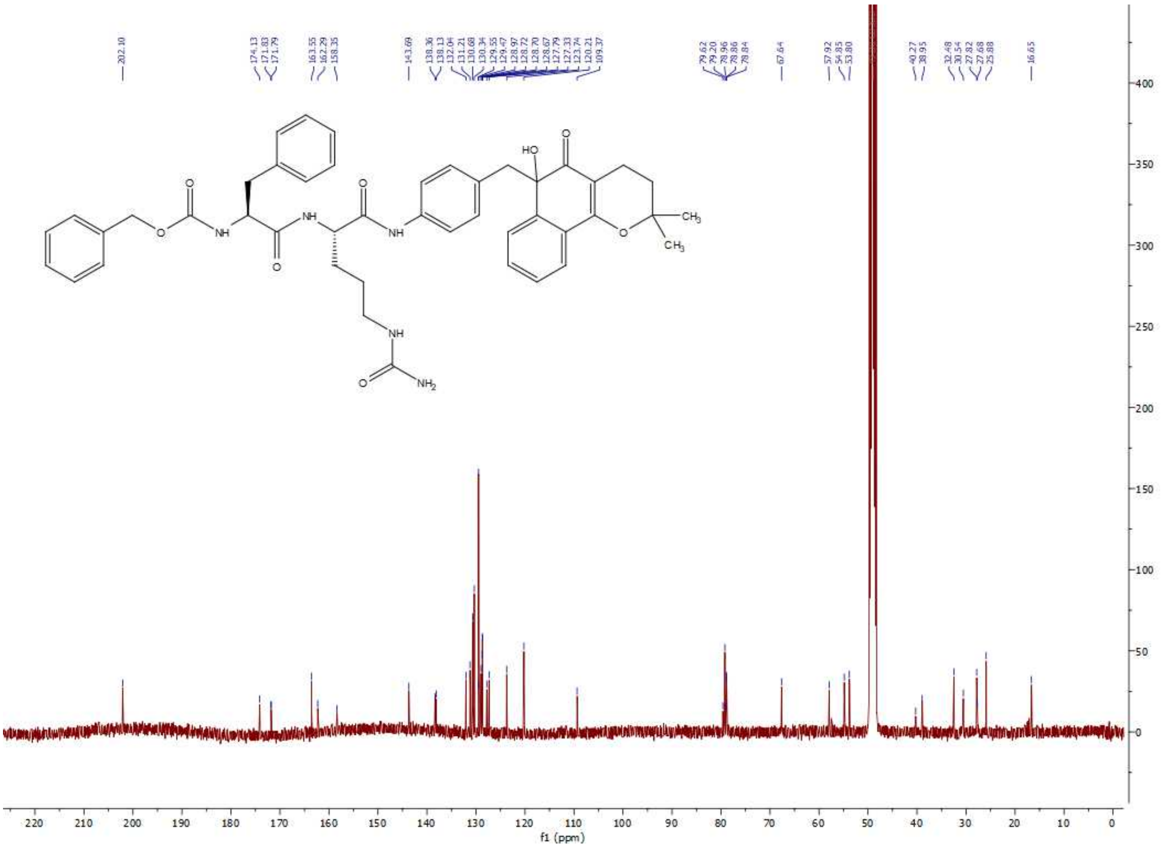

^{1}H NMR (400 MHz, CDCl3) of acetyl-β-lapa-hydroquinone **29**

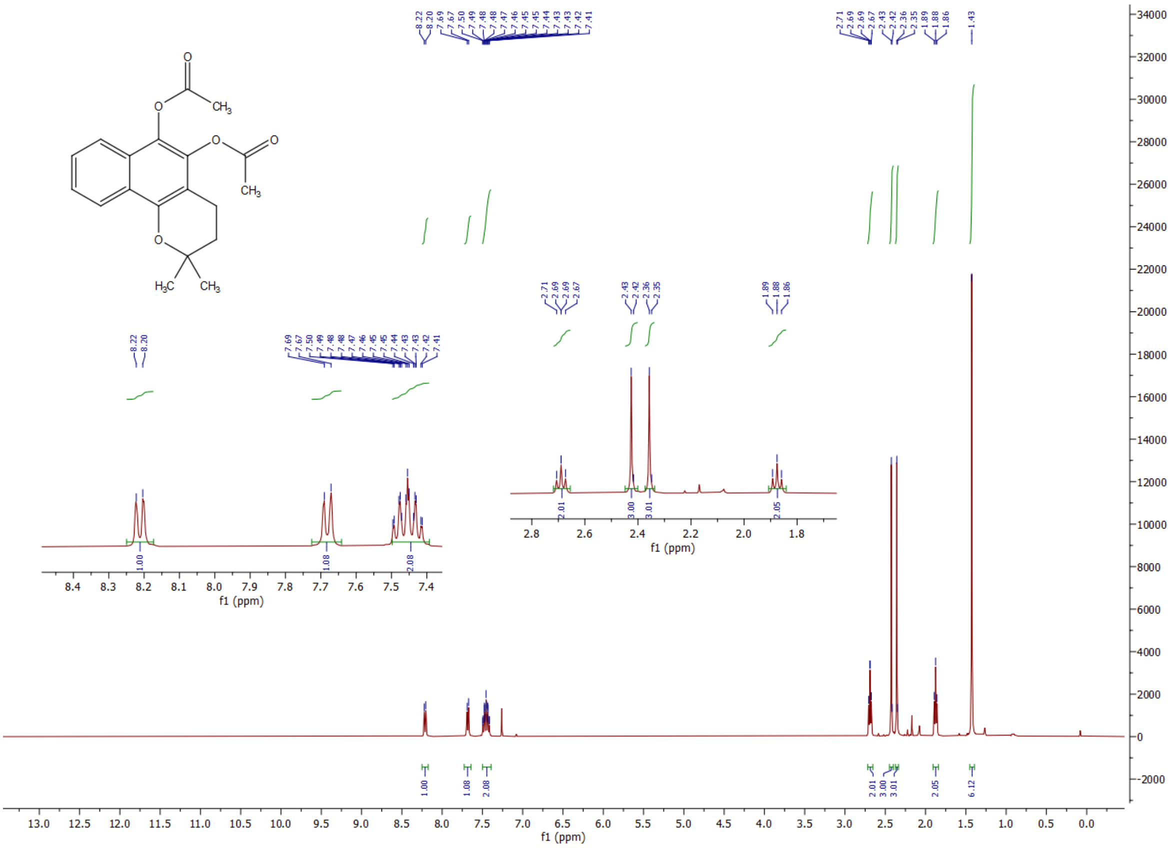

^{13}C NMR (100 MHz, CDCl3) of acetyl-β-lapa-hydroquinone **29**

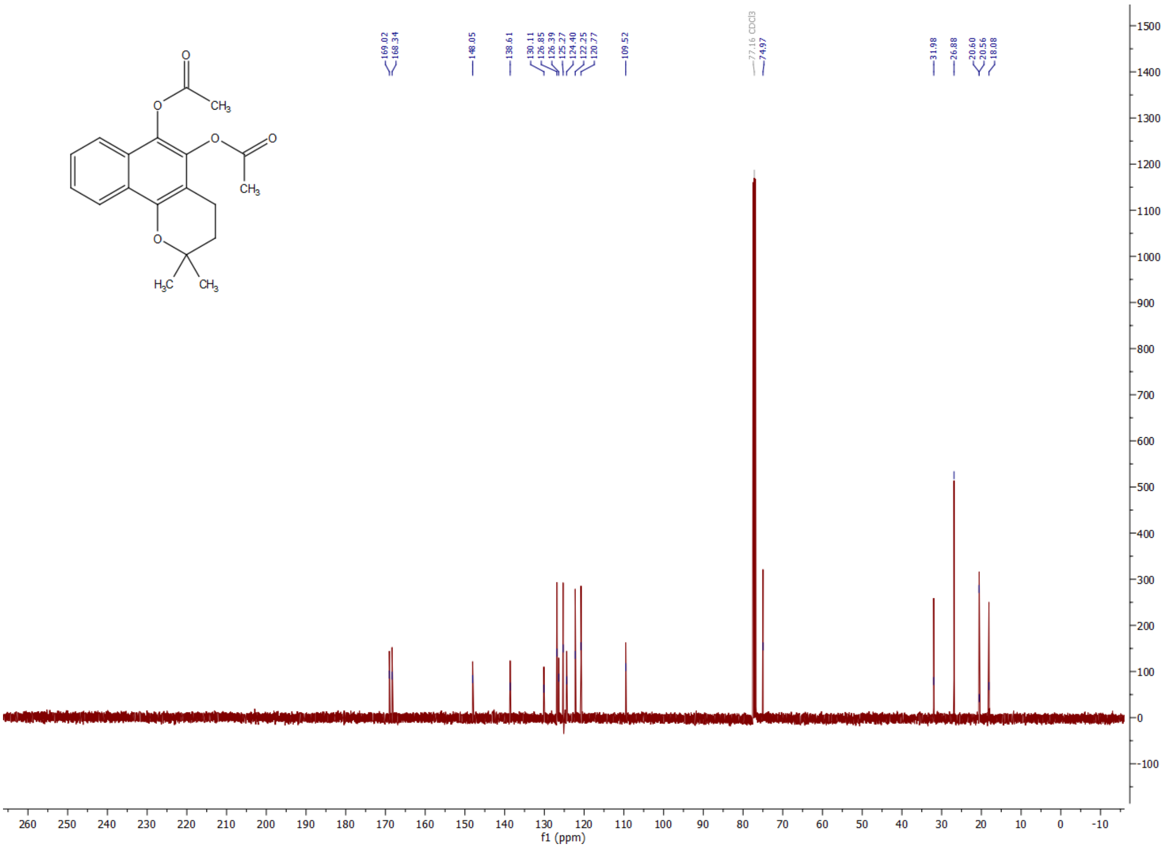

^{1}H NMR (600 MHz, MeOD) of Fmoc-Val-Cit *para*-aminobenzyl β-lapa-ketol **S6**

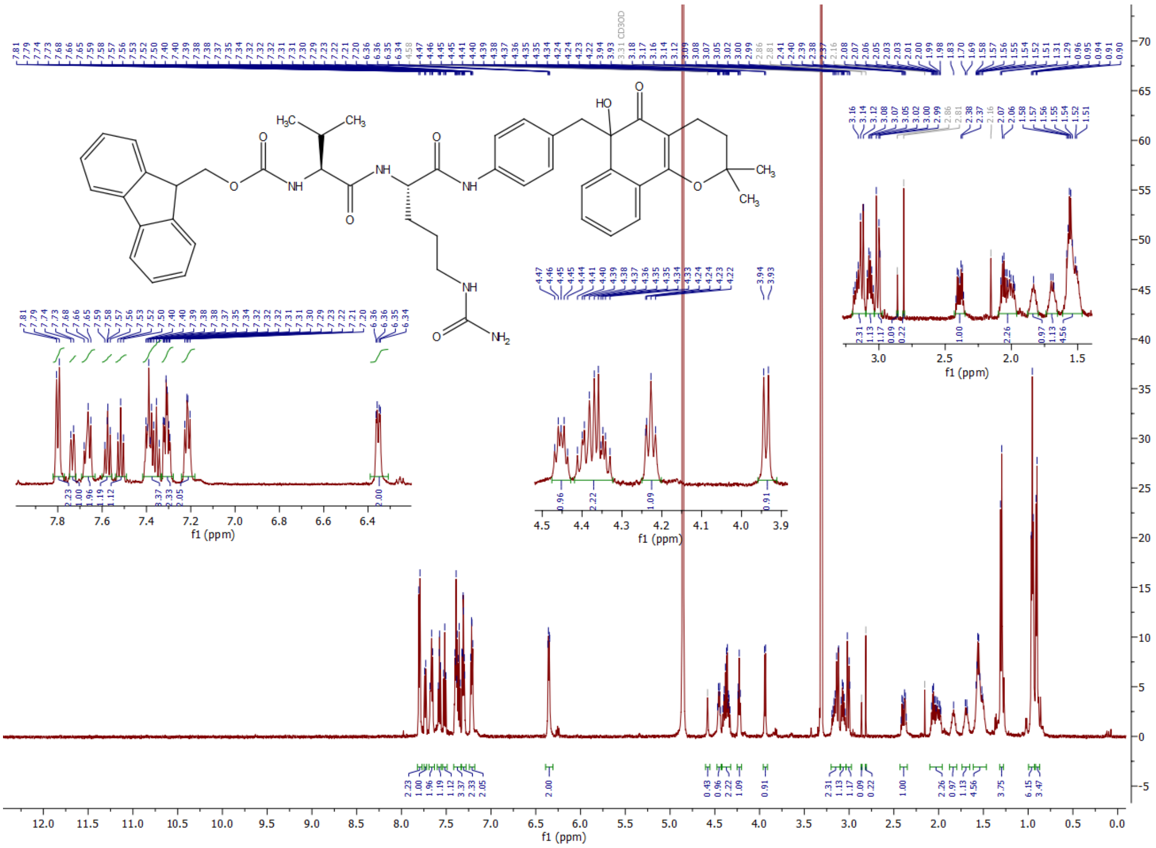

^{13}C NMR (126 MHz, MeOD) of Fmoc-Val-Cit-*para*-aminobenzyl β-lapa-ketol **S6**

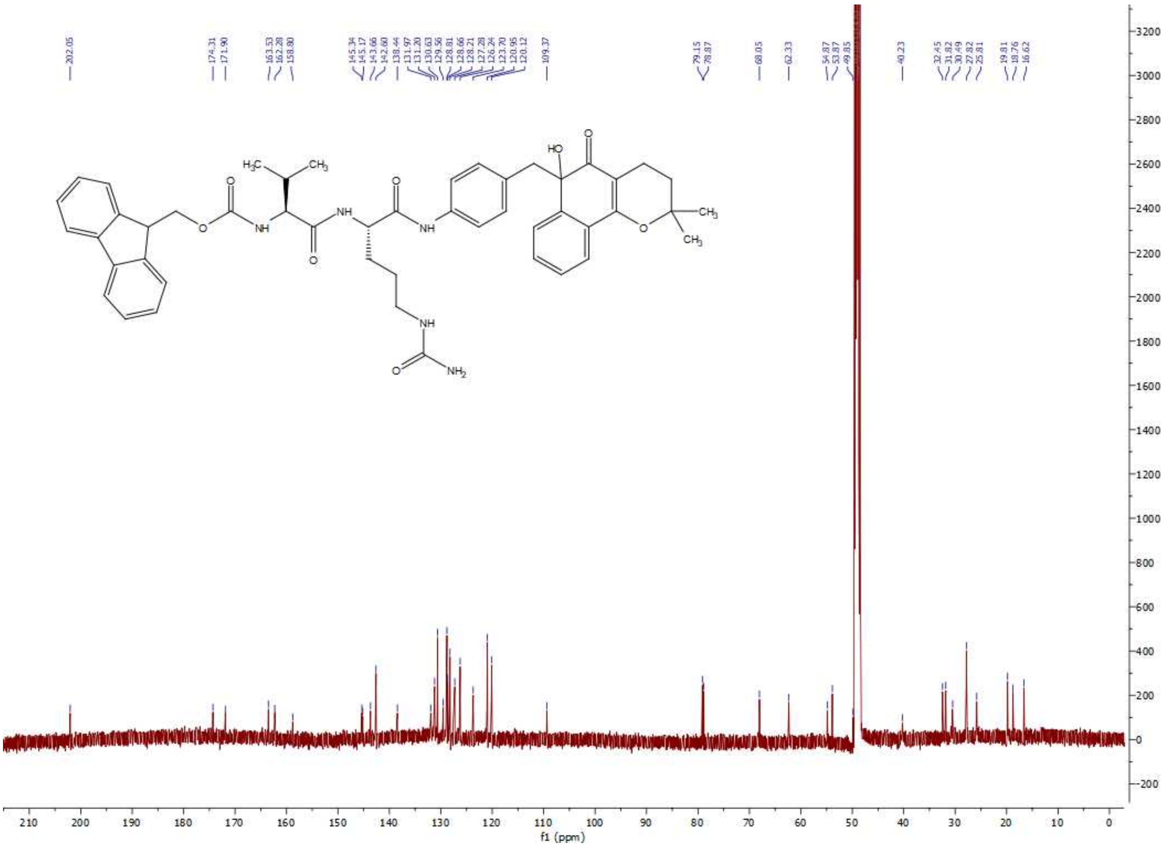

^{1}H NMR (600 MHz, MeOD) of NH_2-Val-Cit-*para*-aminobenzyl β-lapa-ketol **S7**

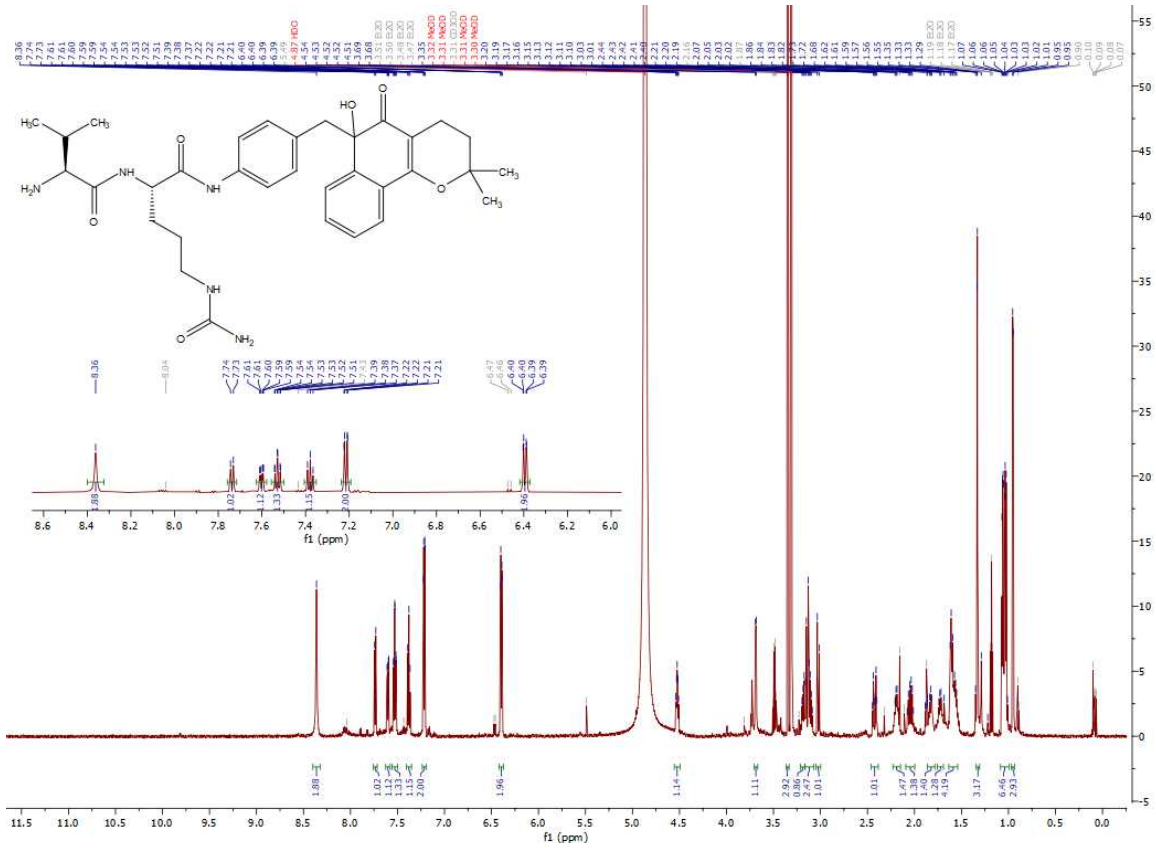

^{1}H NMR (500 MHz, MeOD) of BAA-Val-Cit-*para*-aminobenzyl β-lapa-ketol **30**

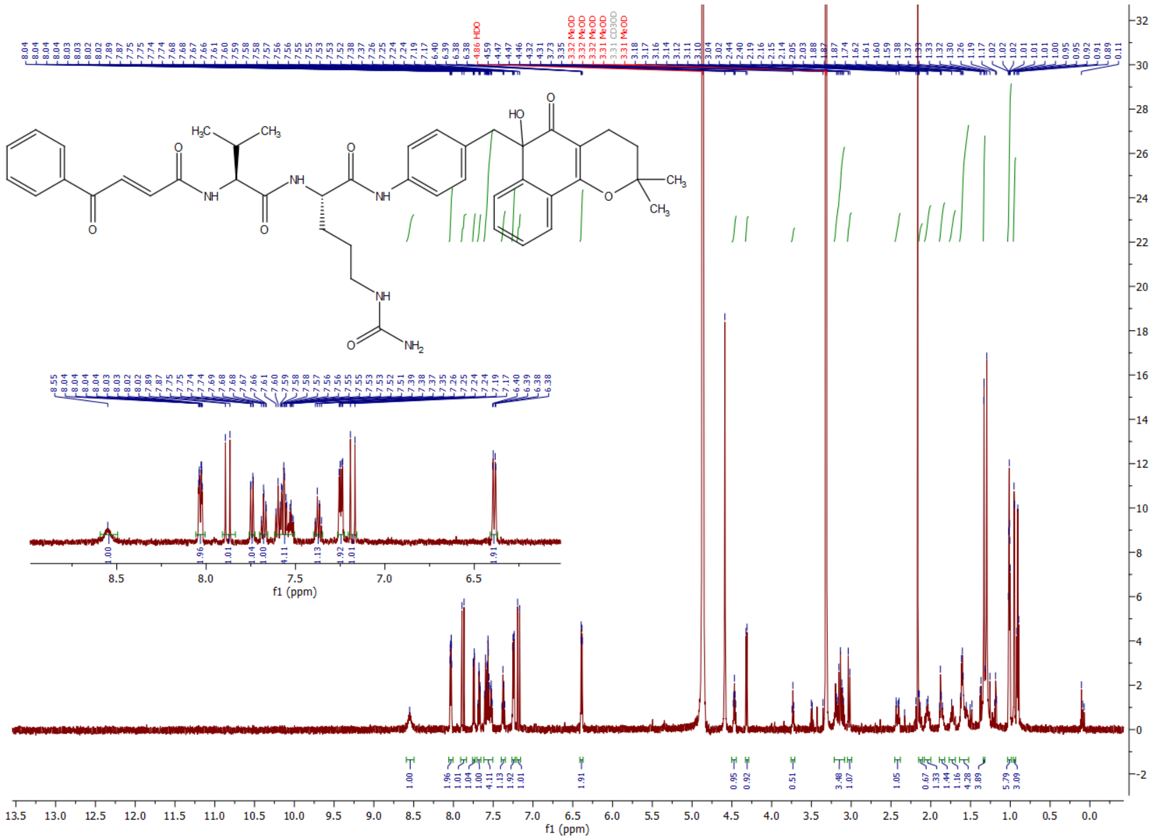

^{13}C NMR (126 MHz, MeOD) of BAA-Val-Cit-*para*-aminobenzyl β-lapa-ketol **30**

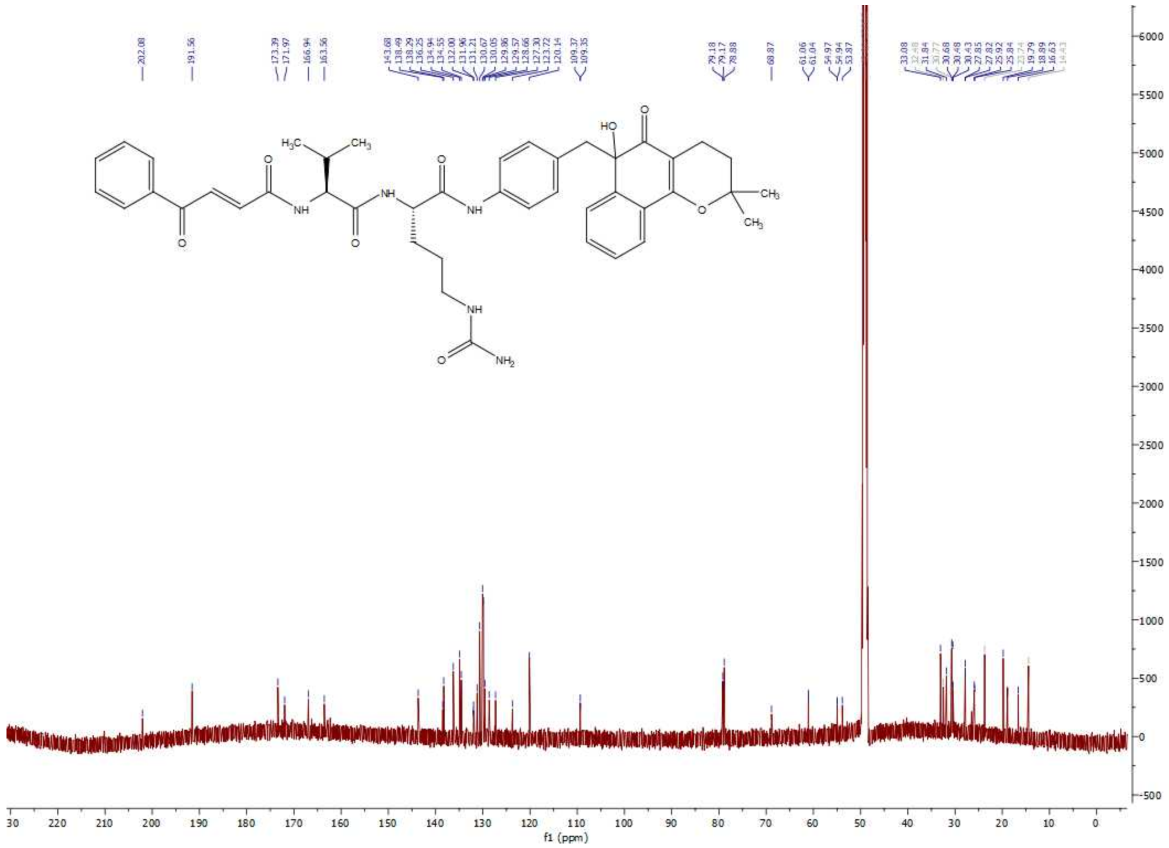

11. References

1. Inagaki, R., Ninomiya, M., Tanaka, K. & Koketsu, M. Synthesis, Characterization, and Antileukemic Properties of Naphthoquinone Derivatives of Lawsone. *ChemMedChem* **10**, 1413–1423 (2015).
2. Bian, J. *et al.* Lewis acid mediated highly regioselective intramolecular cyclization for the synthesis of β-lapachone. *Tetrahedron Letters* **55**, 1475–1478 (2014).
3. Bian, J. *et al.* Synthesis and evaluation of (±)-dunnione and its ortho-quinone analogues as substrates for NAD(P)H:quinone oxidoreductase 1 (NQO1). *Bioorganic & Medicinal Chemistry Letters* **25**, 1244–1248 (2015).
4. Bian, J. *et al.* NbCl5 mediated biomimetic cascade reaction: efficient and scalable one-pot synthesis of dunnione and nor-β-lapachone. *Tetrahedron Letters* **56**, 397–400 (2015).
5. Tzelepis, K. *et al.* A CRISPR Dropout Screen Identifies Genetic Vulnerabilities and Therapeutic Targets in Acute Myeloid Leukemia. *Cell Rep* **17**, 1193–1205 (2016).
6. Koike-Yusa, H., Li, Y., Tan, E.-P., Velasco-Herrera, M. D. C. & Yusa, K. Genome-wide recessive genetic screening in mammalian cells with a lentiviral CRISPR-guide RNA library. *Nat Biotechnol* **32**, 267–273 (2014).
7. Caughey, W. S. & Schellenberg, K. A. Characterization of an Intermediate in the Dithionite Reduction of a Diphosphopyridine Nucleotide Model as a 1,4-Addition Product by Nuclear Magnetic Resonance Spectroscopy1. *J. Org. Chem.* **31**, 1978–1982 (1966).
8. Paruch, K. *et al.* First Friedel−Crafts Diacylation of a Phenanthrene as the Basis for an Efficient Synthesis of Nonracemic [7]Helicenes. *J. Org. Chem.* **65**, 7602–7608 (2000).
9. Guédouar, H., Aloui, F., Beltifa, A., Ben Mansour, H. & Ben Hassine, B. Synthesis and characterization of phenanthrene derivatives with anticancer property against human colon and epithelial cancer cell lines. *Comptes Rendus Chimie* **20**, 841–849 (2017).

10. Shurygina, M. P., Kurskii, Yu. A., Chesnokov, S. A. & Abakumov, G. A. Products of photoreduction of 9,10-phenanthrenequinone in the presence of N,N-dimethylanilines and polymethylbenzenes. *Tetrahedron* **64**, 1459–1466 (2008).
11. Shurygina, M. P., Kurskii, Yu. A., Druzhkov, N. O., Chesnokov, S. A. & Abakumov, G. A. Products and mechanisms of photochemical transformations of o-quinones. *High Energy Chem* **44**, 234–238 (2010).
12. Paquette, L. A. & Hofferberth, J. E. The α-Hydroxy Ketone (α-Ketol) and Related Rearrangements †. in *Organic Reactions* 477–567 (American Cancer Society, 2004). doi:10.1002/0471264180.or062.03.
