# Supplementary material for: Controlled masking and targeted release of redox-cycling ortho-quinones via a C–C bond-cleaving 1,6-elimination
Source: Nat Chem. 2022 Jun 27;14(7):754–65. doi: 10.1038/s41557-022-00964-7 (PMC9252919; doi:10.1038/s41557-022-00964-7)
Supplement: Supplementary file 2 — Reporting Summary [file 41557_2022_964_MOESM2_ESM.pdf]

nature research

Corresponding author(s): Gonçalo Bernardes & Gonzalo Jiménez-Osés

Last updated by author(s): 16/3/2022

Reporting Summary

Nature Research wishes to improve the reproducibility of the work that we publish. This form provides structure for consistency and transparency in reporting. For further information on Nature Research policies, see our Editorial Policies and the Editorial Policy Checklist.

Statistics

For all statistical analyses, confirm that the following items are present in the figure legend, table legend, main text, or Methods section.

n/a	Confirmed	
☐	☒	The exact sample size (n) for each experimental group/condition, given as a discrete number and unit of measurement
☐	☒	A statement on whether measurements were taken from distinct samples or whether the same sample was measured repeatedly
☐	☒	The statistical test(s) used AND whether they are one- or two-sided<br>*Only common tests should be described solely by name; describe more complex techniques in the Methods section.*
☐	☒	A description of all covariates tested
☐	☒	A description of any assumptions or corrections, such as tests of normality and adjustment for multiple comparisons
☐	☒	A full description of the statistical parameters including central tendency (e.g. means) or other basic estimates (e.g. regression coefficient) AND variation (e.g. standard deviation) or associated estimates of uncertainty (e.g. confidence intervals)
☐	☒	For null hypothesis testing, the test statistic (e.g. F, t, r) with confidence intervals, effect sizes, degrees of freedom and P value noted<br>*Give P values as exact values whenever suitable.*
☐	☒	For Bayesian analysis, information on the choice of priors and Markov chain Monte Carlo settings
☐	☒	For hierarchical and complex designs, identification of the appropriate level for tests and full reporting of outcomes
☒	☐	Estimates of effect sizes (e.g. Cohen's d, Pearson's r), indicating how they were calculated

Our web collection on statistics for biologists contains articles on many of the points above.

Software and code

Policy information about availability of computer code

Data collection	Quantum Mechanical transition state geometries, energies and vibrational frequencies were computed with Gaussian 16 Rev C01 (www.gaussian.com).
Data analysis	Computed molecular structures were depicted using Open-Source PyMol 2.3 (https://pymol.org). Theoretical kinetic and equilibrium constants derived from fitting of experimental and computed data were calculated using Microcal Origin Pro 2020b. Mestrenova version 14.2.0 was used for NMR analysis. The MaxEnt algorithm in MassLynx version 4.1 was used for mass spectrometry analysis. Analysis of data was also performed with GraphPad Prism version 8.0 and Origin Pro 2020.

For manuscripts utilizing custom algorithms or software that are central to the research but not yet described in published literature, software must be made available to editors and reviewers. We strongly encourage code deposition in a community repository (e.g. GitHub). See the Nature Research guidelines for submitting code & software for further information.

Data

Policy information about availability of data

All manuscripts must include a data availability statement. This statement should provide the following information, where applicable:

- Accession codes, unique identifiers, or web links for publicly available datasets
- A list of figures that have associated raw data
- A description of any restrictions on data availability

Supplementary Information is available for this paper and details experiments described within the manuscript in greater detail in addition to describing synthetic procedures and characterisation data. Source data of HPLC traces used to calculate the kinetics of PAB-PhQ, PAB-BL and PAB-DN elimination in aqueous solution are provided with the manuscript. All computed geometries, energies and fitting data can be accessed through the Zenodo repository (DOI: 10.5281/zenodo.6325898). No restrictions on data availability apply.

Field-specific reporting

Please select the one below that is the best fit for your research. If you are not sure, read the appropriate sections before making your selection.

☒ Life sciences ☐ Behavioural & social sciences ☐ Ecological, evolutionary & environmental sciences

For a reference copy of the document with all sections, see nature.com/documents/nr-reporting-summary-flat.pdf

Life sciences study design

All studies must disclose on these points even when the disclosure is negative.

Sample size	HPLC assays and in vitro biological experiments contained a minimum of n=3 replicates at each sampling point to allow for calculation of mean result value and an estimation of the standard error of the result value. For the pH-dependent elimination rate analysis experiments performed by HPLC, for some time-points fewer than n=3 replicates are available due to HPLC instrument errors. In the in vivo study, n=5 animals were tested for each test condition.
Data exclusions	No data exclusions apply.
Replication	The number of times each experiment was repeated with similar results is detailed in the figure caption for each experiment described within the Supplementary Information.
Randomization	Randomization was not applicable to this study, as the focus of this study was proof-of-concept of a protection chemistry.
Blinding	Blinding was not applicable to this study. It was not possible within the conditions of the research laboratories this work was performed in to blind the scientists performing the experiments to the samples they were testing.

Reporting for specific materials, systems and methods

We require information from authors about some types of materials, experimental systems and methods used in many studies. Here, indicate whether each material, system or method listed is relevant to your study. If you are not sure if a list item applies to your research, read the appropriate section before selecting a response.

Materials & experimental systems

n/a	Involved in the study	
☐	☒	Antibodies
☐	☒	Eukaryotic cell lines
☒	☐	Palaeontology and archaeology
☐	☒	Animals and other organisms
☒	☐	Human research participants
☒	☐	Clinical data
☒	☐	Dual use research of concern

Methods

n/a	Involved in the study	
☒	☐	ChIP-seq
☒	☐	Flow cytometry
☒	☐	MRI-based neuroimaging

Antibodies

Antibodies used	Gem-IgG1, Gem-LC-V205C, Gem-HC-S442C and Gem-HC-239iC are IgG1 antibodies expressed at AstraZeneca, Cambridge containing the variable region originally found in gemtuzumab, and hence the antibodies bind to CD33. Gem-LC-V205C, Gem-HC-S442C and Gem-HC-239iC are identical to Gem-IgG1 with the specified cysteine mutations on the light chain (LC) or heavy chain (HC) respectively. Control antibody NIP228-HC-239iC was also expressed at AstraZeneca. NIP228-HC-239iC does not bind to CD33 and contains an heavy chain cysteine muation in an identical position to Gem-HC-239iC.
Validation	QC data available for the antibodies used in this work is described within the Supplementary Information.

Eukaryotic cell lines

Policy information about cell lines

Cell line source(s)	HeLa, SKBr3 and HCT-116 were purchased from ATCC. HL-60 was donated from the group of Prof. Bruno Silva-Santos, iMM Lisbon. MCF-7 was donated from the group of Dr. Sérgio Almeida, iMM Lisbon. MOLM-13 was donated from the group of Prof. Tony Kouzarides, Gurdon Institute, University of Cambridge. HEL was either donated from the group of Prof. Bruno Silva-Santos from iMM, Lisbon (in vivo studies), from the group of Dr. Isaia Barbieri, Department of Pathology, University of Cambridge (in vitro ADC studies and shRNA 5-LO Kd study), or donated from the group of Prof. George Vassiliou, Wellcome-MRC Cambridge Stem Cell Institute, Department of Haematology, University of Cambridge (CRISPR 5-LO KO study).

Authentication	Cell lines were not authenticated.
Mycoplasma contamination	All cell lines were tested and confirmed to have no mycoplasma contamination.
Commonly misidentified lines (See ICLAC register)	No commonly misidentified cell lines were used in the study.

Animals and other organisms

Policy information about studies involving animals; ARRIVE guidelines recommended for reporting animal research

Laboratory animals	8 week old female NOD-SCID mice.
Wild animals	No wild animals were used in this study.
Field-collected samples	No field collected samples were used in this study.
Ethics oversight	Instituto de Medicina Molecular João Lobo Antunes & Direção-Geral de Alimentação e Veterinária, Portugal

Note that full information on the approval of the study protocol must also be provided in the manuscript.
